# Clinical and genetic landscape of optic atrophy in 826 families: insights from 50 nuclear genes

**DOI:** 10.1093/brain/awae324

**Published:** 2024-10-18

**Authors:** Yuxi Zheng, Panfeng Wang, Shiqiang Li, Yuxi Long, Yi Jiang, Dongwei Guo, Xiaoyun Jia, Mengchu Liu, Yiyan Zeng, Xueshan Xiao, J Fielding Hejtmancik, Qingjiong Zhang, Wenmin Sun

**Affiliations:** State Key Laboratory of Ophthalmology, Zhongshan Ophthalmic Center, Sun Yat-sen University, Guangdong Provincial Key Laboratory of Ophthalmology and Visual Science, Guangzhou 510060, China; State Key Laboratory of Ophthalmology, Zhongshan Ophthalmic Center, Sun Yat-sen University, Guangdong Provincial Key Laboratory of Ophthalmology and Visual Science, Guangzhou 510060, China; State Key Laboratory of Ophthalmology, Zhongshan Ophthalmic Center, Sun Yat-sen University, Guangdong Provincial Key Laboratory of Ophthalmology and Visual Science, Guangzhou 510060, China; State Key Laboratory of Ophthalmology, Zhongshan Ophthalmic Center, Sun Yat-sen University, Guangdong Provincial Key Laboratory of Ophthalmology and Visual Science, Guangzhou 510060, China; State Key Laboratory of Ophthalmology, Zhongshan Ophthalmic Center, Sun Yat-sen University, Guangdong Provincial Key Laboratory of Ophthalmology and Visual Science, Guangzhou 510060, China; State Key Laboratory of Ophthalmology, Zhongshan Ophthalmic Center, Sun Yat-sen University, Guangdong Provincial Key Laboratory of Ophthalmology and Visual Science, Guangzhou 510060, China; State Key Laboratory of Ophthalmology, Zhongshan Ophthalmic Center, Sun Yat-sen University, Guangdong Provincial Key Laboratory of Ophthalmology and Visual Science, Guangzhou 510060, China; State Key Laboratory of Ophthalmology, Zhongshan Ophthalmic Center, Sun Yat-sen University, Guangdong Provincial Key Laboratory of Ophthalmology and Visual Science, Guangzhou 510060, China; State Key Laboratory of Ophthalmology, Zhongshan Ophthalmic Center, Sun Yat-sen University, Guangdong Provincial Key Laboratory of Ophthalmology and Visual Science, Guangzhou 510060, China; State Key Laboratory of Ophthalmology, Zhongshan Ophthalmic Center, Sun Yat-sen University, Guangdong Provincial Key Laboratory of Ophthalmology and Visual Science, Guangzhou 510060, China; Ophthalmic Molecular Genetics Section, Ophthalmic Genetics and Visual Function Branch, National Eye Institute, National Institutes of Health, Bethesda, MD 20892, USA; State Key Laboratory of Ophthalmology, Zhongshan Ophthalmic Center, Sun Yat-sen University, Guangdong Provincial Key Laboratory of Ophthalmology and Visual Science, Guangzhou 510060, China; State Key Laboratory of Ophthalmology, Zhongshan Ophthalmic Center, Sun Yat-sen University, Guangdong Provincial Key Laboratory of Ophthalmology and Visual Science, Guangzhou 510060, China

**Keywords:** mitochondrial, hereditary optic neuropathy, Leber’s hereditary optic neuropathy, dominant optic atrophy

## Abstract

Hereditary optic neuropathies (HON) comprise a group of diseases caused by genetic defects in either the mitochondrial or nuclear genomes. The increasing availability of genetic testing has expanded the genetic and phenotypic spectrum of HON more broadly than previously recognized. The genetic and phenotypic landscape of HON is attributed to 50 nuclear genes, so we genetically analysed patients with suspected HON from a group of 4776 index cases following our previous study on 1516 probands with Leber’s HON (LHON) who had mitochondrial DNA variants.

Exome sequencing was performed in 473 probands diagnosed with nuclear gene-related HON (nHON) and 353 cases with unsolved LHON. Sequencing and variant interpretation of the 50 nuclear genes indicated that the diagnostic yield of exome sequencing for nHON was 31.50% (149/473), while it was markedly lower [1.42% (5/353)] for LHON patients without primary mtDNA mutations. The top five genes implicated in nHON in our in-house cohort were *OPA1*, *WFS1*, *FDXR*, *ACO2* and *AFG3L2*, which accounted for 82.46% of probands. Although *OPA1* was the most prevalent nHON-causative gene in both our cohort (53.25%) and a literature review (37.09%), the predominance of *OPA1*, *WFS1* and *FDXR* differed significantly between our in-house cohort and the literature review (*P*-adjusted < 0.001). Fundus changes in nHON could be stratified into three categories: the most common was optic atrophy at examination (78.79%); the rarest was LHON-like optic atrophy (3.64%); and optic atrophy with concurrent retinal degeneration (17.57%), an independent risk factor for visual prognosis in nHON, occurred at an intermediate frequency. A systematic genotype-phenotype analysis highlighted different genetic contributions for ocular, extraocular neurological and extraocular non-neurological phenotypes. In addition, systemic variant analysis at the individual gene level suggested a revised interpretation of the pathogenicity of a *WFS1* heterozygous truncation variant.

This study provides a panoramic view of the genetic and phenotypic profiles of HON in a real-world study and the literature. The categories of nHON fundus phenotypes will benefit future studies on the molecular mechanisms underlying HON and targeted therapies. In addition to routine ophthalmic examinations, careful examination of extraocular symptoms and meaningful genetic counselling are warranted for patients with nHON.

## Teaser

Zheng *et al.* categorize the funduscopic manifestations of nuclear gene-related hereditary optic neuropathies into three distinct patterns: optic atrophy at the time of examination, Leber’s hereditary optic neuropathy-like presentations and optic atrophy with retinal degeneration.

## Introduction

**Figure 1:**
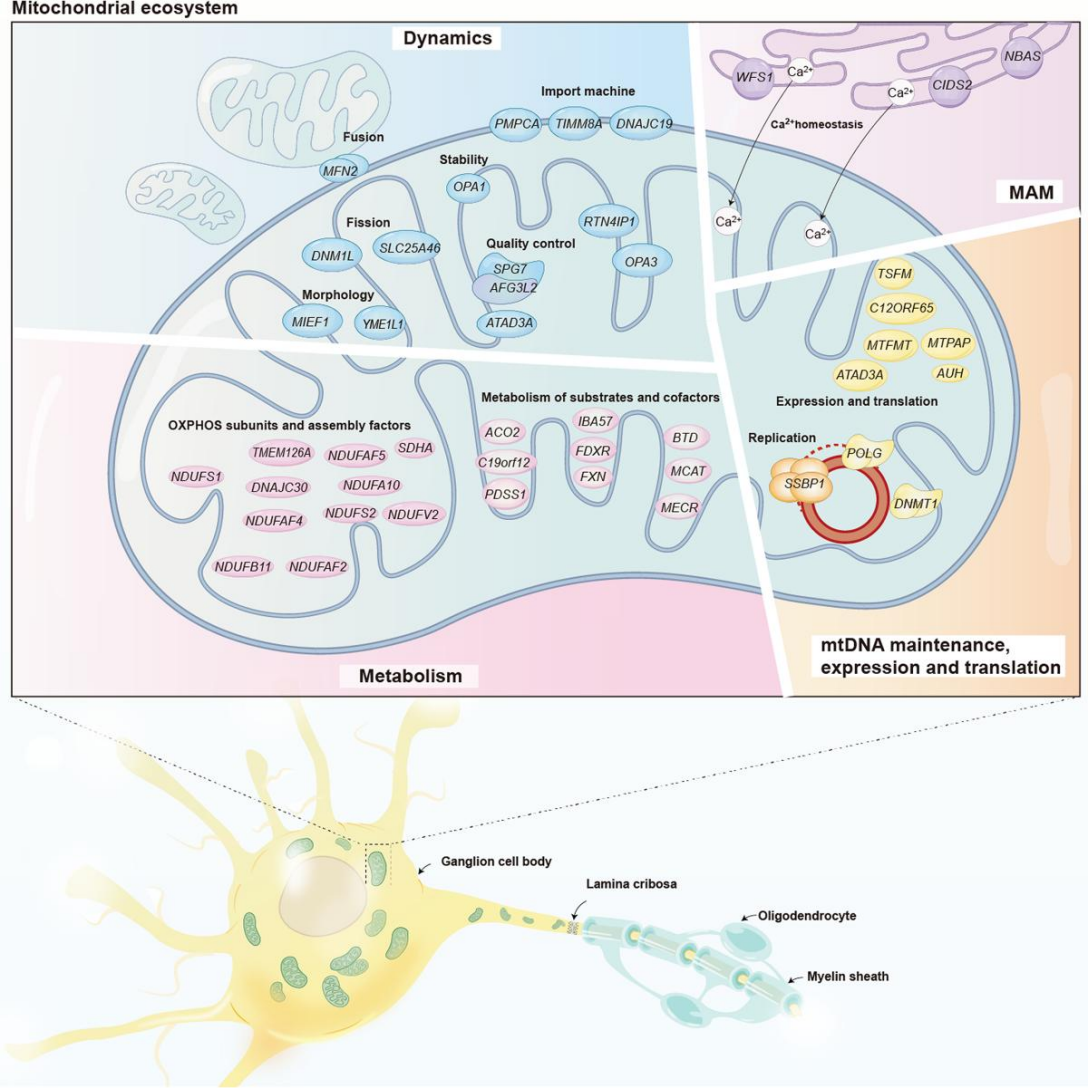
**Mitochondrial ecosystem contributing to retinal ganglion cell degeneration.** Schematic demonstrating the localization and function of hereditary optic neuropathy (HON)-related gene encoded proteins. Mitochondrial disease genes divided into four subsets according to their function: (i) mitochondrial membrane dynamics; (ii) mitochondrial-associated membrane networks (MAM); (iii) metabolism; and (iv) mtDNA maintenance. Given the multiple roles of these genes, broad categories were used to ensure appropriate assignment. The mitochondrial ecosystem displays a delicate balance, and perturbations in one component propagate detrimental effects across upstream and downstream cellular cascades. The illustration was created in Adobe Illustrator.

Hereditary optic neuropathies (HON) are characterized by a preferential loss of retinal ganglion cells due to genetic variants in either nuclear (nDNA) or mitochondrial (mtDNA) genes and exhibit both genetic and clinical heterogeneity.^1-3^ HON resulting from mtDNA or nDNA variants often presents with different clinical manifestations, including variations in onset age, optic disc pathology, disease progression, visual prognosis and extraocular organ involvement.^4,5^ The molecular pathogenesis and natural history of Leber’s hereditary optic neuropathy (LHON) are relatively well understood. It is clinically characterized by a painless, subacute loss of central vision, typically between the ages of 15 and 35, with a marked sex bias and incomplete penetrance.^5,6^ Distinct from LHON, nuclear gene-related HON (nHON) has a more complex clinical presentation. nHON—mainly caused by mutations in the *OPA1* gene—is classically associated with an insidious onset during the first decades of life with no sex predilection and a better visual prognosis.^7-9^ However, the increased availability of genetic testing in clinical ophthalmological practice has revealed a broader genetic and phenotypic spectrum of nHON than previously appreciated. Variants in at least 50 nuclear genes have been reported to cause either isolated or syndromic nHON. Variants in most nuclear genes frequently disrupt mitochondrial function, either directly or indirectly (Fig. 1). These impacts on mitochondrial function span a spectrum from disturbances in oxidative phosphorylation or contiguous metabolic pathways to disrupted mitochondrial dynamic homeostasis between organelle fusion and fission and could extend even to include interactions with the endoplasmic reticulum. These pathogenic mechanisms are interwoven within the cell, in which individual variants may disturb a complex mitochondrial ecosystem (Fig. 1). The phenotypic spectrum of nHON has also been expanded. Variants in some nuclear genes, including *DNAJC30*, *MCAT*, *MECR*, *NDUFA12*, *NDUFAF5* and *NDUFS2*, have recently been identified in patients with typical LHON-like phenotypes manifesting hallmarks similar to classical LHON.^10-14^ In addition, variants in several genes, including *ACO2*, *FDXR*, *SSBP1* and *RTN4IP1*, have been identified in patients showing optic atrophy plus retinal degeneration at varying levels of severity.^15-17^ In addition, a considerable proportion of patients with nHON have been reported to develop extraocular symptoms, including abnormalities in the central nervous, endocrine, cardiovascular and muscular systems.^3^ These observations underscore the manifestation of optic atrophy as either an isolated condition or part of a multisystem degenerative process with high variability in clinical presentation. The HON spectrum, as reported in the literature and real-world practice, remains unclear. Recent studies have delineated the genetic profiles of HON in French and Italian populations, providing meaningful and important information regarding the genetic spectrum in clinical practice.^18,19^ However, a panoramic and systematic view of the genetic landscape of HON, including ocular manifestations, genotype-phenotype correlations and the impact of other factors on visual prognosis across different genes, remains limited in East-Asian populations. Such a holistic perspective is crucial to developing a nuanced understanding of HON within an ophthalmological context.

Herein, the clinical and genetic landscape of HON was elucidated by systematically evaluating our in-house cohort as well as carrying out an extensive review of the literature. Variants in *OPA1* was found to contribute the most to nHON in our in-house data as well as different ethnic groups reported in the literature. Compared with non-East Asians, our in-house nHON patients had higher proportions of *OPA1* and *FDXR* mutations but lower proportions of *WFS1* mutations. By systematically categorizing the fundus changes in nHON, three distinct fundus phenotypes were delineated: optic atrophy at the time of examination; LHON-like; and optic atrophy plus retinal degeneration. The visual prognosis for each group was estimated. The systematic genotype-phenotype insight into both ocular and extraocular features based on literature review may be useful not only to ophthalmologists and neurologists but also otologists, endocrinologists, osteologists, etc. In the literature, variants of four of the top five genes identified from our in-house nHON cohort are indicated to cause ophthalmic dysfunction prior to neurological symptoms. Thus, the meticulous clinical characterization of nHON is imperative for precise genetic counselling, personalized therapeutic intervention and efficacy assessments of individual patients.

## Materials and methods

### Subjects

The institutional review board of Zhongshan Ophthalmic Center approved this study. Patients with suspected HON and their available family members were recruited from the Pediatric and Genetic Clinic, Zhongshan Ophthalmic Center, Guangzhou, China. Clinical data and peripheral venous blood samples were collected after written informed consent forms were signed, with the process conducted in compliance with the ethical principles of the Declaration of Helsinki. Genomic DNA was extracted from the peripheral lymphocytes of patients and their available family members according to our previously described method.^20^

### Variant detection and confirmation

Next-generation sequencing was conducted in genomic DNA samples from a total of 826 probands with HON, including 473 patients with a clinical diagnosis of nHON and 353 patients with clinically suspected LHON with the exclusion of four primary variants. Of the 473 patients suspected to have nHON, targeted exome sequencing or whole-exome sequencing (WES) and Sanger sequencing for the four primary LHON variants was performed for 265 patients, while WES and entire mitochondrial DNA sequencing was carried out for 208 patients. WES and entire mitochondrial DNA sequencing were performed for all 353 patients with LHON. After the exclusion of 64 mitochondrial variants with confirmed pathogenicity based on the Mitomap database, a list of 50 causative nuclear genes was selected through a comprehensive evaluation based on prior research (Supplementary Table 1).^3,21^ Variants in the 50 HON-related nuclear genes were obtained from exome sequencing data from the 826 probands as well as in-house controls (Supplementary Fig. 1). The procedures for targeted exome sequencing and WES, along with multistep bioinformatics analyses, were implemented as detailed previously (Supplementary Fig. 1).^22^ The allele frequency of each variant was retrieved from gnomAD (https://gnomad.broadinstitute.org/, access date: April 2023). The potential effects of missense variants were evaluated using five *in silico* tools—REVEL, CADD, PROVEAN, SIFT and Poly-phen2—via the dbNSFP database (version 4, accessed April 2023) (Supplementary Table 2). The Rare Disease Data Center (https://rddc.tsinghua-gd.org/, accessed August 2023), Human Splicing Finder (https://hsf.genomnis.com/, accessed August 2023) and SpliceAI (https://spliceailookup.broadinstitute.org/, accessed August 2023) were used to predict the possible effects of splicing changes (Supplementary Table 2). Co-segregation analysis of the variants was performed for available family members after Sanger sequencing. The variants were evaluated according to the American College of Medical Genetics and Genomics (ACMG) and Association for Molecular Pathology standards (Supplementary Table 2). Variant nomenclature followed the guidelines of the Human Genome Variation Society (https://www.hgvs.org/mutnomen/).

### Clinical assessment

Detailed clinical data from patients with pathogenic variants in 50 nuclear genes, including sex, age at onset, best corrected visual acuity (BCVA), initial symptoms and family histories, were collected for subsequent assessments. Details about the timing and pattern of visual loss were collected from the clinical records or interviews with patients or their guardians (Supplementary Table 3). Detailed ophthalmologic examinations were performed, including direct ophthalmoscopy, BCVA, optical coherence tomography (OCT), fundus photography, fundus autofluorescence, wide-field scanning laser ophthalmoscopy and electroretinography.

#### Fundus phenotype classification

This classification incorporated a multifaceted assessment based on initial symptoms, progressive changes in the optic disc (such as whether its appearance transitions from oedema to temporal pallor, before culminating in complete optic atrophy) and comprehensive ophthalmologic evaluations, including direct ophthalmoscopy, fundus photography, OCT, electroretinography and autofluorescence imaging. In our classification scheme, the phenotype was classified as LHON-like if at least one of the following criteria were met: (i) presence of optic disc oedema according to either fundus photographs or medical records; or (ii) appearance of optic disc pallor with a well-documented history of acute or subacute vision loss and previously normal visual baseline. The phenotype was defined as optic atrophy plus retinal degeneration when the following fundus changes were observed: tapetoretinal degeneration, attenuated retina vessels or silver wire changes, peripheral retinal pigmentation, or abnormal hyperautofluorescent or hypoautofluorescent appearances of the fundus on autofluorescence imaging. Finally, the classification of optic atrophy refers to patients with a pale optic disc with insidious onset but without signs of LHON-like or retinal degeneration phenotypes at the time of examination, knowing that optic atrophy might later progress to show retinal degeneration or syndromic features. Classification into fundus categories was carried out by a senior ophthalmologist with clinical expertise in genetic eye diseases.

### Statistical analysis and visualization

Statistical analysis and visualization were performed in the R environment (v.4.1.2) and RStudio. Snellen visual acuity measurements were converted to the logarithm of the minimum angle of resolution (logMAR) equivalents for analysis proposes. The better eye from each participant was selected for analysis. Blindness was defined as a BCVA < 0.05 on Snellen testing. Exposure time to blindness was analysed with a Cox regression. Exposure time was considered to be the age of the patient at the initial visit, and the genetic mutation was considered to be the exposure. Variables, including genotypes and fundus characteristics, were entered into the multivariate cox regression analysis. The prevalence of category variables (presented as numbers and percentages) between groups was analysed with Fisher’s exact test. The Bonferroni test was applied to correct for multiple comparisons. Differences between the age of onset of the ocular and neurological symptoms were analysed with paired non-parametric tests (Wilcoxon matched-pairs test). Differences were considered statistically significant at *P* < 0.05.

### Literature review

A meticulous review of genotypes and phenotypes in nHON was performed based on a search conducted on 1 October 2023 of the Human Gene Mutation Database, Web of Science, Google and PubMed. Specific search terms were employed, including ‘mitochondrial disease,’ ‘optic atrophy,’ ‘hereditary optic neuropathy,’ ‘syndromic optic atrophy,’ ‘dominant optic atrophy,’ ‘autosomal recessive optic atrophy’ and the individual gene names in the nHON list. Exclusion criteria included: (i) articles not in English; (ii) review articles reiterating previously published cases; (iii) articles lacking the details of individual cases (e.g. a study showing only the number of patients in a large cohort of optic neuropathy or other neurological disorder without specific clinical data or causative variants); and (iv) interventional clinical trials. Two authors independently conducted a literature review of all potentially relevant publications and extracted data from the full text of eligible studies. Any discrepancies were resolved through discussion with a third senior author (Q.Z.). Data were collected and organized using a commonly employed spreadsheet (Supplementary Table 4). For each selected article, the following data were extracted: PubMed ID; patient ID; sex; age at onset of first manifestation; age at vision loss onset; age at visit; any reported systematic complications; BCVA; inheritance pattern; genetic variant; and variant type. Reported cases were characterized using Human Phenotype Ontology (HPO) terminology (https://hpo.jax.org/, access date: March 2024), which offers a standardized lexicon for phenotypic abnormalities in human genetic diseases. The subcategories of HPO terms were grouped into broader categories for analytical clarity. For example, nystagmus HP:0000639, strabismus HP:0000486, ophthalmoplegia HP:0000602 and slow saccadic eye movements HP:0000514 were grouped into abnormality of eye movement HP:0000496. Duplicated descriptions of cases that were reported in more than one article were excluded based on the patient ID, genetic variant, age at onset of first manifestation, age at vision loss onset and names of authors.

## Results

### The genetic spectrum of hereditary optic neuropathies from the in-house dataset and literature review

**Figure 2:**
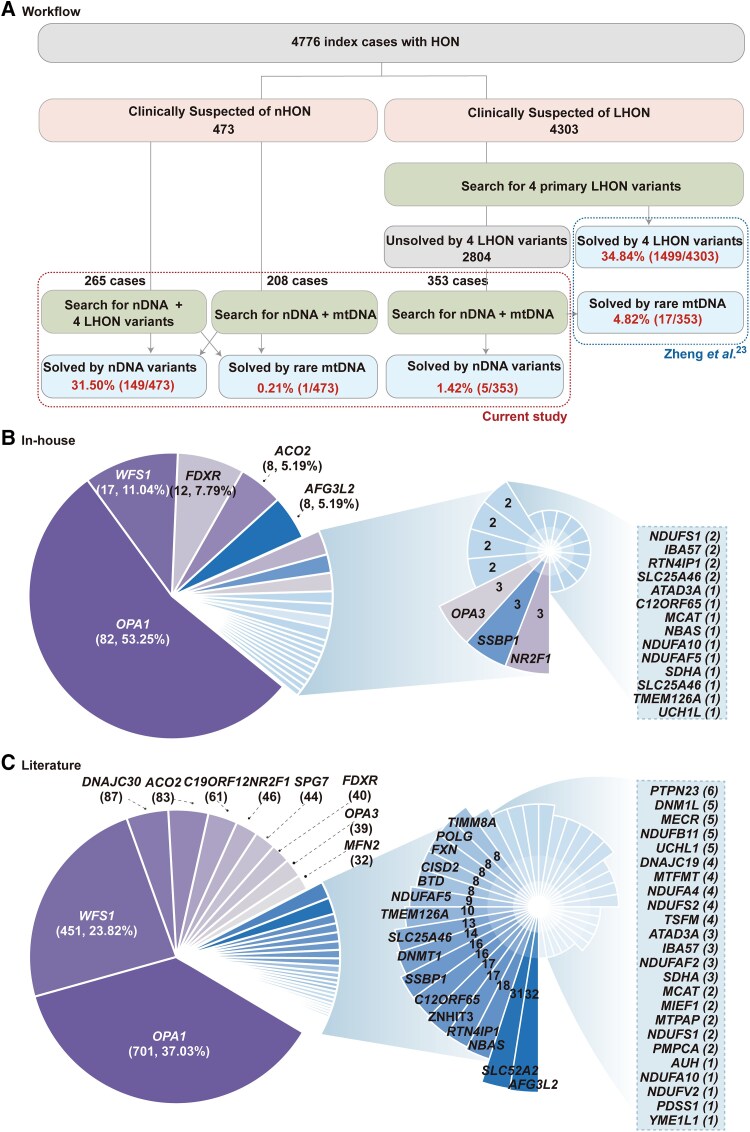
**Study workflow and frequency distribution of hereditary optic neuropathy-related nuclear genes**. (**A**) Overview of the workflow for identifying hereditary optic neuropathy (HON)-related nuclear gene variants. *Left*: Next-generation sequencing was conducted in genomic DNA samples from 473 patients with clinically suspected nuclear gene-related HON (nHON). Whole-exome sequencing (WES) and sequence analyses of four primary Leber’s HON (LHON) variants (m.11778G>A, m. 14484T>C, m.3460G>A and m.3635G>A) were carried out for 265 of the 473 patients, while WES and entire mitochondrial DNA sequencing analyses were carried out for 208 of the patients. None of the four primary LHON variants were identified in the cohort of 473 patients; one patient with optic atrophy plus mitochondrial encephalopathy carried a mitochondrial variant of proven pathogenicity, m.3243A>G. *Right*: The methodology for identifying causative genetic variants in 353 patients initially suspected of having LHON but lacking the four predominant mtDNA mutations. Comprehensive genomic analyses involving whole-exome and mitochondrial sequencing showed that 17 of these patients did indeed carry mitochondrial variants clearly associated with LHON (from Zheng *et al*.^23^). (**B**) The prevalence of in-house families harbouring mutations in HON-related nuclear genes. The pie chart displays the distribution of the five most frequently encountered genes in the families, while the expanded pie chart illustrates the remaining genetic landscape. (**C**) Distribution of optic atrophy pedigrees harbouring HON-related nuclear gene mutations in the literature. The colours representing genes in **B** correspond with those in **C**. The contributions of each gene are indicated as percentages, with only those greater than 5% displayed.

A total of 4776 in-house patients clinically suspected of having HON, 473 with nHON and 4303 with LHON, were referred for genetic diagnosis. To gain a comprehensive understanding of the genetic and clinical features of nHON, 36 families in our previous studies were included for statistical analysis (Supplementary Table 3). Among the 473 patients in the nHON cohort, none of the four primary LHON mtDNA variants were detected; however, one patient with optic atrophy accompanied by mitochondrial encephalopathy was found to carry a pathogenic mitochondrial variant, m.3243A>G. The diagnostic yield for nHON was 31.50% (149/473) for nuclear genes and 0.21% (1/473) for mitochondrial genes. For the 4303 probands clinically suspected of having LHON, the diagnostic yield from the screenings of the four primary LHON mtDNA variants was 34.84% (1499/4303).^23^ Of the remaining 2804 in-house patients, 353 were selected to undergo WES following evaluation of the quality of the genomic DNA samples and integrity of the clinical data. A nuclear gene diagnosis yield of 1.42% (5/353) and mitochondrial gene diagnosis yield of 4.82% (17/353) was achieved in these patients, who were negative in the initial screenings of the four primary LHON mtDNA genes (Fig. 2A).

In total, 162 variants were defined as causing disease phenotypes, including 111 pathogenic variants, 39 likely pathogenic variants and 12 variants of unknown significance (VUS) according to the ACMG criteria and guidelines. All of the 12 VUS were considered to cause recessive phenotypes in combination with a second pathogenic or likely pathogenic variant. In addition, 32 VUS in genes associated with dominant phenotypes were classified as having uncertain effects on phenotype (Supplementary Table 2). The 154 families with nDNA variants included 82 in *OPA1* (53.25%), 17 in *WFS1* (11.04%), 12 in *FDXR* (7.79%), 8 in *ACO2* (5.19%), 8 in *AFG3L2* (5.19%), 3 in *NR2F1* (1.95%), 3 in *OPA3* (1.95%), 3 in *SSBP1* (1.95%), 2 in *NDUFS1* (1.30%), 2 in *IBA57* (1.30%), 2 in *RTN4IP1* (1.30%) and 2 in *SLC25A46* (1.30%), plus variants in 10 other genes found in single cases (Fig. 2B). The top five genes, namely *OPA1*, *WFS1*, *FDXR*, *ACO2* and *AFG3L2*, accounted for 82.47% (127/154) of solved cases in the in-house cohort.

From the literature, pathogenic variants of the HON nuclear genes have been identified in at least 5057 families to date (Supplementary Fig. 3A). After excluding 2790 families that lacked a documented ocular phenotype, the remaining 2267 families were further analysed (Supplementary Fig. 2), the individual gene contributions from which are illustrated in Supplementary Fig. 3B. Among these families, 367 exhibited no recorded optic nerve phenotype and seven had a documented normal optic nerve (Supplementary Fig. 2). The remaining 1893 families presented with reported optic nerve atrophy, with the top 10 implicated nuclear genes being *OPA1* (701, 37.03%), *WFS1* (451, 23.82%), *DNAJC30* (87, 4.60%), *ACO2* (83, 4.38%), *C19orf12* (61, 3.22%), *NR2F1* (46, 2.43%), *SPG7* (44, 2.32%), *FDXR* (40, 2.11%), *OPA3* (39, 2.06%) and *MFN2* (32, 1.69%) (Fig. 2C). *OPA1*, *WFS1, FDXR, NR2F1* and *OPA3* were in the top 10 list of genes contributing to nHON from our in-house data, the non-East-Asian population and East-Asian population reported in the literature (Supplementary Table 5). However, the proportions of *OPA1*, *WFS1*, *FDXR* and *NDUFS1* differed significantly between the in-house cohort and non-East Asians (*P*-adjusted < 0.001) (Supplementary Table 5). As for real-world practice, seven of the top 10 genes were the same in our in-house cohort and the French and Italian cohorts (Supplementary Table 5). From the literature, among the cases of optic atrophy caused by *OPA1*, 84.31% (591/701) presented as non-syndromic optic atrophy (involving visual symptoms only), a rate that surpasses that of *ACO2* (71.08%) but falls below *DNAJC30* (which exhibits complete prevalence, 100%). Regarding the in-house cohort, 85.06% (131/154) of families manifested non-syndromic optic atrophy, with families harbouring *OPA1* variants representing the largest fraction (80 families) compared with other genes.

### Fundus characteristics of the in-house nHON cohort

**Figure 3:**
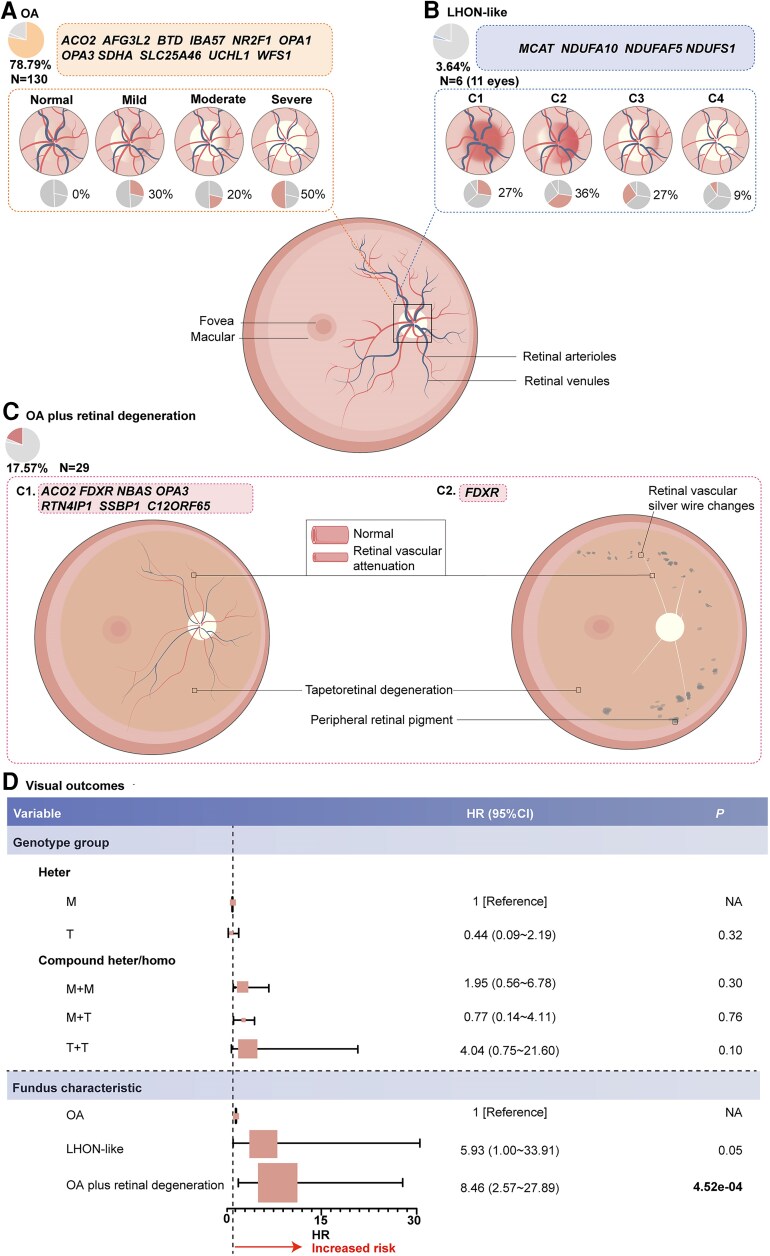
**Ocular fundus manifestations and visual prognosis associated with hereditary optic neuropathy-related nuclear genes.** The fundus characteristics caused by hereditary optic neuropathy (HON)-related nuclear genes fall into three categories—optic atrophy (OA), Leber’s hereditary optic neuropathy (LHON)-like and OA plus retinal degeneration (OA+RD), which accounted for 78.79% (orange), 3.64% (blue) and 17.57% (red) of in-house patients, respectively. (**A**) OA is characterized by a pale optic disc, which can graded as mild, moderate or severe. The mild phase is characterized by temporal pallor of the optic disc with the superior, inferior and nasal quadrants uninvolved. The moderate phase shows noticeable thinning of the retinal nerve fibre layer (RNFL) in the superior or inferior quadrant and temporal pallor of the optic disc, with the nasal quadrant remaining unaffected. The severe phase features a clear optic disc boundary and 360° pallor of the optic disc. The pie charts below the optic disc schema indicate the contributions of the corresponding grades. (**B**) The four categories of LHON-like optic disc characteristics. C1: 360° optic disc oedema accompanied by peripapillary microangiopathy; C2: blurred optic disc boundary in ether the superior or inferior quadrant with less than 90° temporal pallor; C3: more than 90° temporal pallor, sparing the nasal quadrant; and C4: clear optic nerve boundary with a 360° pale optic disk. The pie charts below the optic disc schema indicate the contributions of the corresponding grades in 11 eyes from six LHON-like patients. (**C**) For OA+RD, two sub-categories are recognized, one featuring retinal vascular attenuation and tapetoretinal degeneration and the other attenuated retinal silver-like retinal vessels, pigment deposits in the mid-peripheral retina and tapetoretinal degeneration. (**D**) Forest plot of multivariable hazard ratios for blindness. The genotype groups and fundus manifestations were entered into a multivariate Cox regression analysis to determine the characteristics correlating with blindness in patients possessing HON-related nuclear gene variants. Patients with a best corrected visual acuity (BCVA) < 0.05 on Snellen testing were considered blind. Hazard ratios (HR) and 95% confidence intervals (CI) were computed and visualized using the forestplot package in R. M = missense/in-frame deletion; NA = not applicable; OA = optic atrophy at the time of examination; T = truncation.

The fundus characteristics caused by HON-related nuclear genes in the nHON cohort fell into three categories: isolated optic atrophy at the time of examination (78.79% of patients); LHON-like (3.64% of patients); and optic atrophy plus retinal degeneration (17.57% of patients). Optic disc alterations in isolated optic atrophy were divided into three categories: mild, moderate and severe (Fig. 3A). The mild category is characterized by temporal pallor of the optic disc, with pink optic discs in the superior, inferior and nasal quadrants. The moderate category includes noticeable thinning of the RNFL in the superior or inferior quadrant, temporal pallor of the optic disc and an unaffected nasal quadrant (Fig. 3A). The severe phase features a clear optic disc boundary and 360° pallor of the optic disc. The OCT data indicated progressive decline in sectoral RNFL thickness with increasing severity of the optic neuropathy, particularly obvious in the superior and inferior sectors, which largely supported the categorization according to fundus characteristics (Supplementary Fig. 4). In our previous study, LHON-like optic disc characteristics were classified into four categories: manifestation of peripapillary microangiopathy and telangiectasis (C1); followed by temporal optic nerve pallor less than 90° (C2); progression to over 90° pallor, sparing the nasal quadrant (C3); and finally, a distinct optic nerve boundary with a completely pale optic disk (C4) (Fig. 3B).^23^

In optic atrophy plus retinal degeneration, two sub-categories exist. One is marked by retinal vascular attenuation as well as tapetoretinal degeneration. These are different from the changes seen in typical retinitis pigmentosa, which include waxy pale optic discs, attenuated retinal vessels and peripheral degeneration with bone spicule pigmentation. The second category features attenuated retinal silver-like vessels coexisting with pigment deposits in the mid-peripheral retina in addition to tapetoretinal degeneration (Fig. 3C).

The OCT data revealed a progressive decline in RNFL thickness with increasing severity of optical atrophy, which was particularly pronounced in the superior and inferior sectors (Supplementary Fig. 4A). In the four categories of LHON-like phenotypes, the OCT data demonstrated that the superior and inferior sectors were thickened in C1, and as the condition progressed to C2, a thinning of the temporal sector RNFL became apparent. This thinning further propagated into the temporal and inferior quadrants in C3, while diffuse RNFL thinning was evident across all quadrants in C4 (Supplementary Fig. 4B). For patients with optic atrophy plus retinal degeneration, the OCT data showed severe and diffuse RNFL thinning in all quadrants (Supplementary Fig. 4C).

When comparing the BCVA among the optic atrophy, LHON-like and optic atrophy plus retinal degeneration groups, the latter manifested a worse BCVA than the optic atrophy and LHON-like groups, although the difference in BCVA between the optic atrophy plus retinal degeneration and LHON-like groups did not reach statistical significance (Supplementary Fig. 5). To analyse the correlation of the genotype groups and fundus characteristics with overall visual survival in patients with nHON, a multivariate cox regression analysis was performed among patients with HON-related nuclear gene variants in the in-house cohort. Multivariate analysis substantiated the proposal that the optic atrophy plus retinal degeneration phenotype (hazard ratio = 8.46; 95% confidence interval = 2.57, 27.89; *P* = 4.52 × 10^−4^) is significantly independently associated with worse visual acuity outcome (Fig. 3D).

### Characteristics of optic atrophy

**Figure 4:**
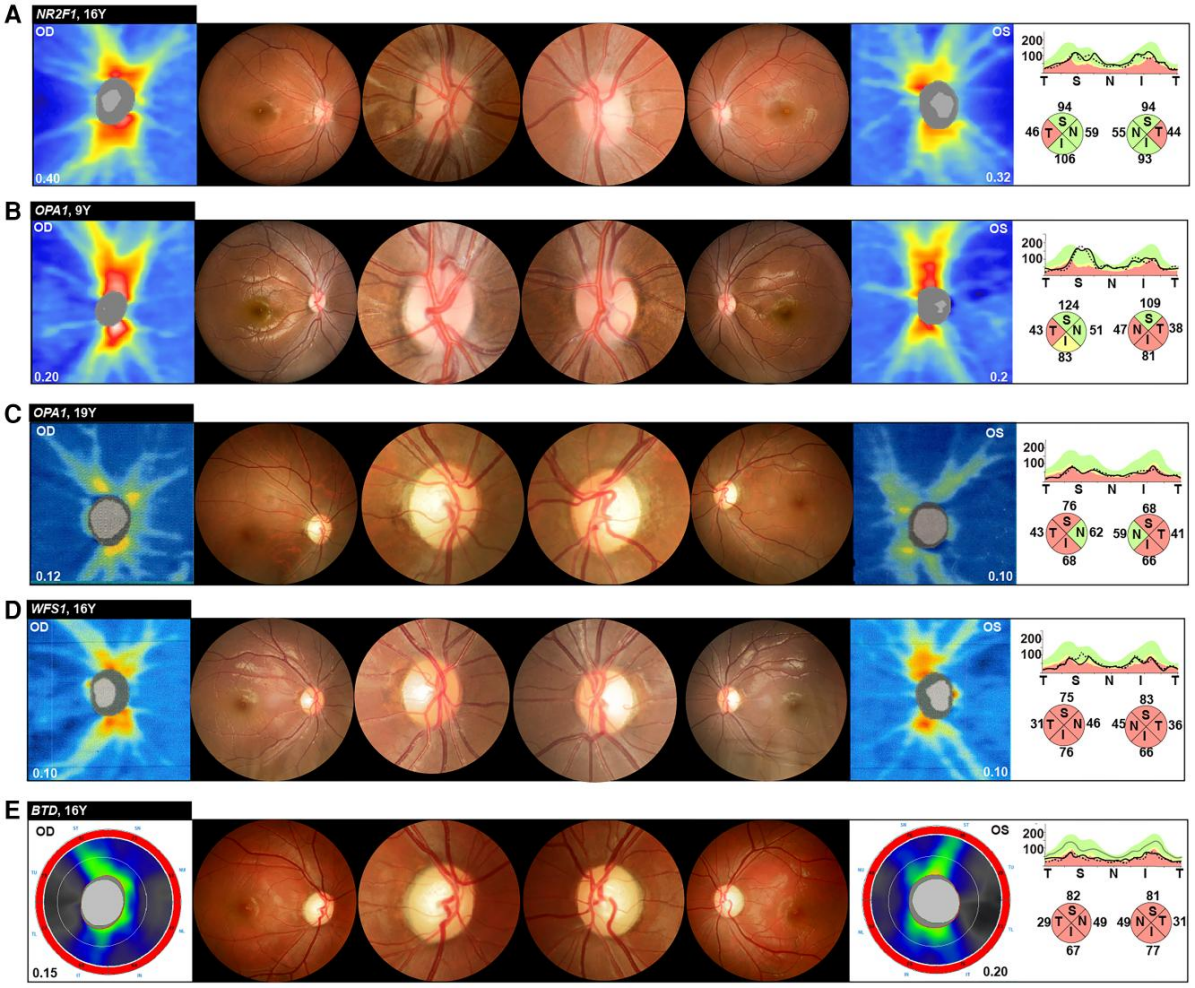
**Fundus and optical coherence tomography findings in isolated optic atrophy. A**–**E** each show the clinical data from an individual patient, highlighting the optic nerve head photographs captured with a fundus camera and the optical coherence tomography (OCT) results. Annotations in the *top left* detail the causative gene and patient’s age at examination. These images illustrate varying degrees of optic disc atrophy across different genes and individuals. The OCT heat map indicates the thickness of the peripapillary retinal nerve fibre layer (RNFL). The line graphs of RNFL-thickness (*right*) were extracted from data for the temporal, superior, nasal, inferior, temporal (T-S-N-I-T) quadrants based on a cube scan of the optic disc. The green shading indicates RNFL-thickness within normal limits, yellow denotes borderline thickness and red signifies RNFL thinning. The optic nerve heads in **A** and **B** exhibit temporal pallor as seen on the OCT RNFL-thickness evaluation, with predominant thinning observed in the temporal aspects of the nerve head. The optic nerve head in **C** displays temporal pallor exceeding 90°, sparing the nasal quadrant. The optic nerve heads in **D** and **E** show severe optic disc pallor. In particular, **E** shows marked excavation and severe pallor of the optic nerve head, highlighting extensive atrophic changes.

Patients with optic atrophy typically present with slowly progressive, bilateral central vision loss. The characteristics of the optic nerve head in optic atrophy can range from mild to severe (Fig. 4). Optic disc pallor increases as the disease progress, particularly in the temporal region. OCT revealed mild temporal thinning to general diffuse thinning of the RNFL in the papillomacular bundle in patients (Fig. 4 and Supplementary Fig. 4).

### Characteristics of families with LHON-like phenotypes

A total of five families exhibiting LHON-like fundus phenotypes were identified in our in-house cohort. One had the p.Leu135Ser/p.364_370del mutation in *NDUFA10*, one had the p.Met279Arg/p.Met279Arg mutation in *NDUFAF5*, two had p.Arg158His/p.Asp138Ala and p.His66Leu/p.Ser457Ilefs*7 mutations in *NDUFS1* and the fifth had a p.Leu81Arg/p.Tyr230* mutation in *MCAT* (Supplementary Fig. 6). Patients from these five families reported subacute painless onset of vision loss; the two patients carrying variants in *NDUFAF5* and one with *NDUFS1* variants also complained of sequential vision loss in both eyes within 2 months.

**Figure 5:**
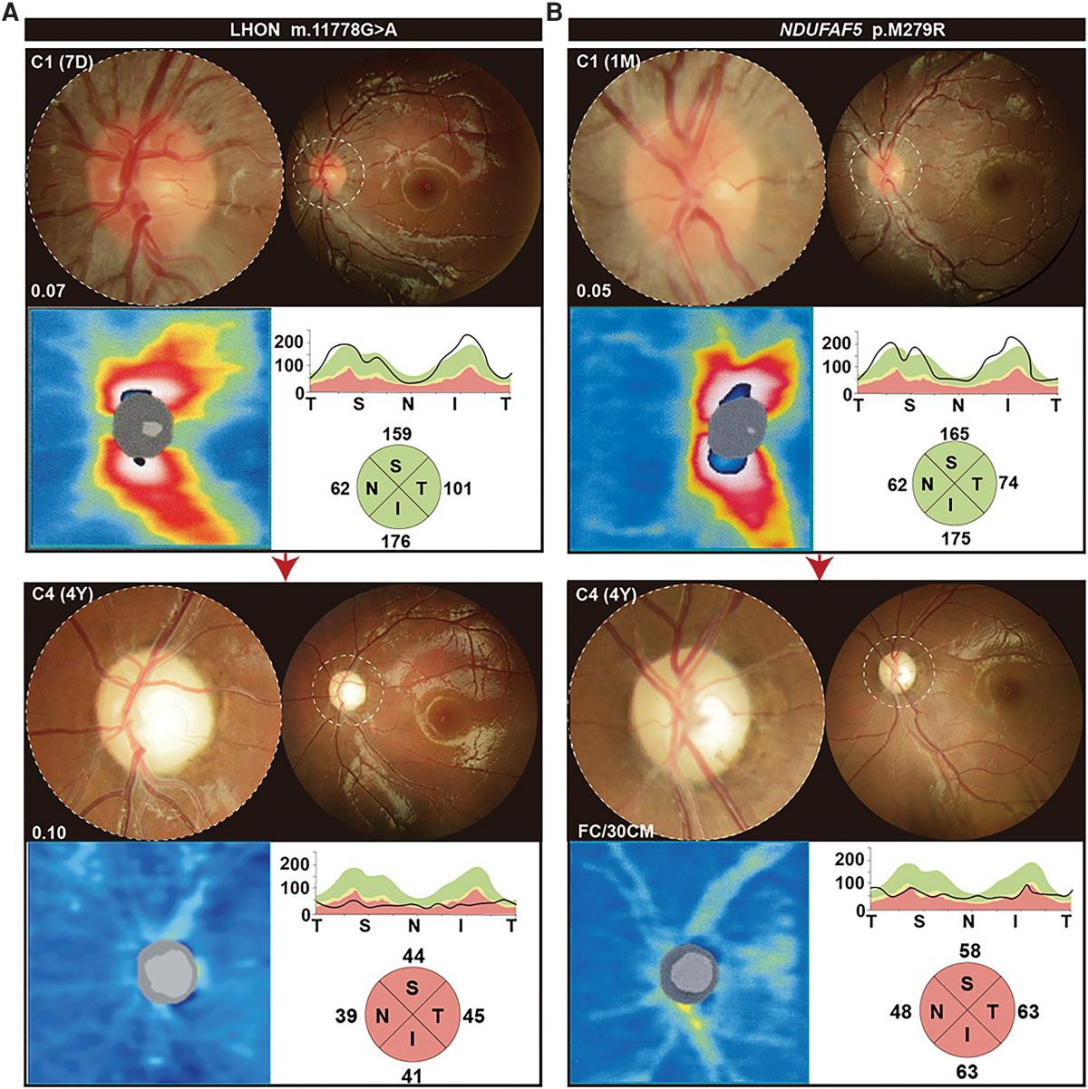
**Optic disc conversion in nuclear gene phenocopying Leber’s hereditary optic neuropathy.** (**A**) The optic disc in a patient with category C1 Leber’s hereditary optic neuropathy (LHON) manifests 360° optic disc oedema accompanied by peripapillary microangiopathy. Four years later, the optic disc was categorized as C4, showing a clear optic nerve boundary with a 360° pale optic disk. (**B**) The optic disc in a patient harbouring biallelic mutations in *NDUFAF5* underwent the same category conversion as in LHON.

The elder brother of two siblings carrying biallelic variants in *NDUFAF5* was first referred to the ophthalmic centre due to subacute painless loss of central vision sequentially affecting each eye within a few weeks. Fundus examination in this patient revealed optic disc oedema with peripapillary telangiectatic microangiopathy and retinal vascular tortuosity which was indistinguishable from typical LHON fundus phenotypes (Fig. 5). Ten months later, the younger sibling exhibited similar symptoms of central vision loss and exhibited LHON-like alterations in the optic disc. Their electroretinograms were normal, but with decreased amplitudes and delayed peaks in the visual evoked potentials. Although LHON was first suggested as a diagnosis, negative Sanger sequencing for the primary mtDNA variants led to WES, with the identification of a homozygous missense mutation in *NDUFAF5* (p.Met279Arg) in both brothers. Four years later, both exhibited pale optic disc and diffuse thinning of the RNFL. The progression of alterations in their optic discs, from early to advanced stages over 4 years, matched the C1 to C4 categorization delineated in our prior research (Figs 3B and 5B).^23^

### Fundus characteristics in optic atrophy plus retinal degeneration

Fundus changes in patients with variants in seven genes exhibited optic atrophy plus retinal degeneration in our in-house cohort, including *ACO2*, *FDXR*, *NBAS*, *OPA3*, *RTN4IP1*, *SSBP1* and *C12ORF65*.

#### ACO2

**Figure 6:**
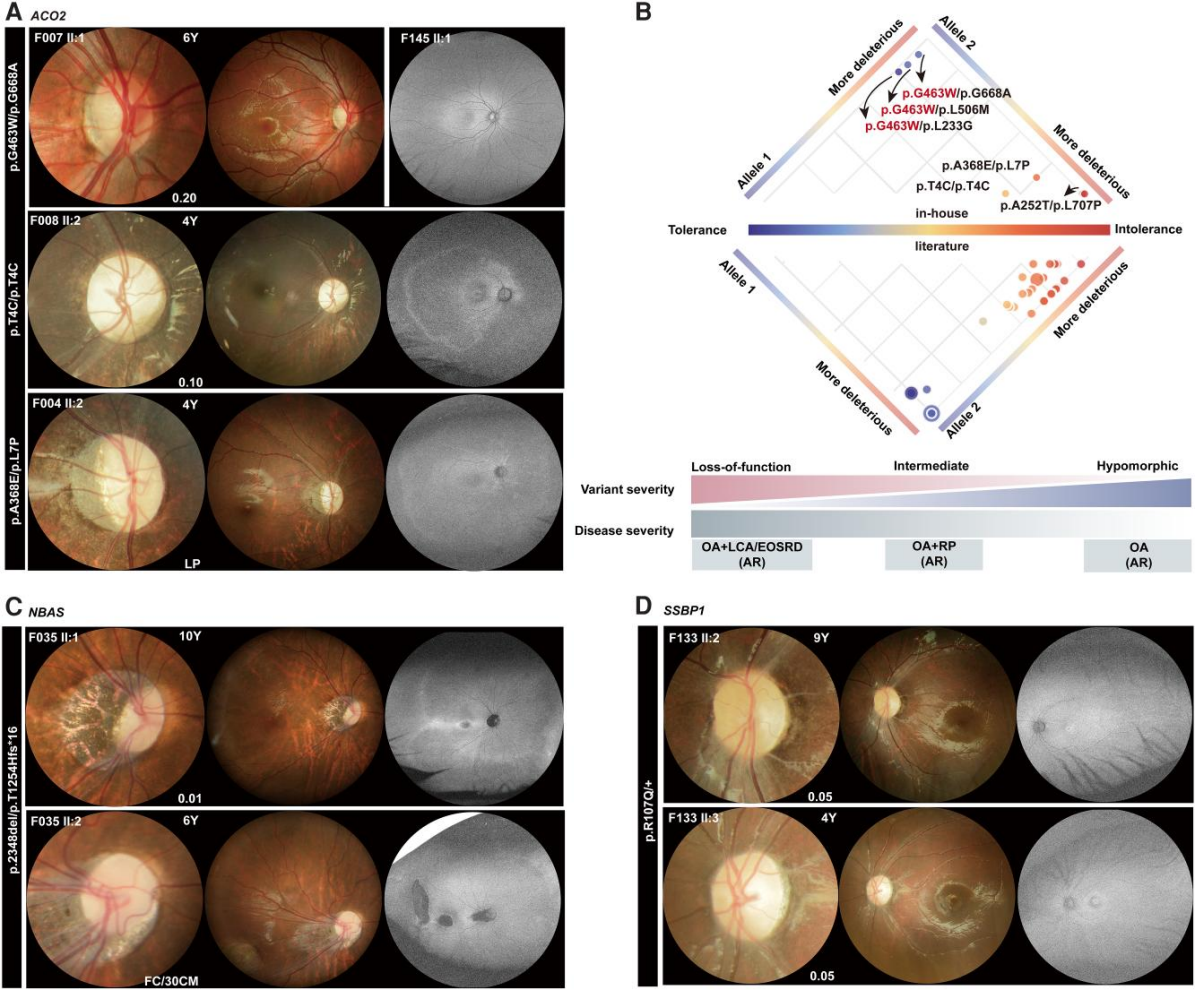
**Fundus characteristic in optic atrophy plus retinal degeneration.** (**A**) Optic nerve head, fundus and fundus autofluorescence (FAF) in three patients with *ACO2* mutations. The fundus images of these three individuals showed an increased degree of attenuated vessels and tessellated fundus from *top* to *bottom*. The FAF of F007:II1 is provided in Supplementary Fig. 7, and because his eyelashes could lead to misinterpretation, the FAF of another patient (F145 II:1) with optic atrophy but a normal retina was used as a control. (**B**) Heat map based on the deleteriousness of each allele from low to high. The colour of each filled circle, from cool to warm, indicates the predicted severity of the phenotype based on the predicted pathogenicity of the biallelic alleles from mild to severe. This heat map was primarily used to demonstrate that the hypomorphic variant was associated with mild forms of *ACO2*-related retinopathy, indicating that the phenotypic severity of *ACO2*-related fundus characteristics reconciles with the prediction heat map. It has been indicated that high pathogenicity variants could cause early-onset severe retinal dystrophy (EOSRD) or Leber congenital amaurosis (LCA) phenotypes, while hypomorphic variants are implicated in optic atrophy (OA). (**C**) Optic nerve head, fundus and FAF of patients with *NBAS* mutations. Patient F035:II-2 exhbits temporal dragging of the optic disc, accompanied by retinal traction. FAF shows demarcated hypo-autofluorescence. The colour image of the fundus of patient F035:II-1 shows a tessellated fundus and tilted optic disc with peripapillary atrophy. (**D**) Optic nerve head, fundus and FAF of patients with *SSBP1* mutations. The colour images of F133-II:2 and F133-II:3 show an atrophic optic nerve head, and the fundus images show attenuated vessels and pigmentary changes. The FAF of F133-II:2 shows an area of hypo-autofluorescence in the mid-peripheral retina and increased autofluorescence around the vessels. F133-II:3 shows doughnut-like hyper-autofluorescence in the fovea. FC = finger count; LP = light perception.

Among the nine patients with biallelic *ACO2* variants, four (F002:II-1,F004:II-2, F008:II-1, F008:II-2) from three families had severe retinopathy, including retinitis pigmentosa and Leber congenital amaurosis; the remaining five patients (F001:II-1,F003:II-2, F005:II-1, F006:II-1, F007:II-1), three of which harbour the same hypomorphic p.Gly463Trp variant (Fig. 6A and B), each from a different family, showed optic atrophy. These findings suggested that a mild form of *ACO2*-associated optic atrophy was associated with this presumably hypomorphic allele, with a high allele frequency, regardless of any potential pathogenic missense variant in the other allele in trans (Fig. 6B). The BCVA of *ACO2*-associated isolated optic atrophy at first examination ranged from 0.10 to 0.6, with 40% (2/5) demonstrating a BCVA better than 0.3; these patients had a median onset age of 11 years, which was significantly later than the 1.5 years in patients with *ACO2*-associated Leber congenital amaurosis/retinitis pigmentosa (*P* = 0.015).

The fundus changes of *ACO2*-associated optic atrophy included pallor of the optic disc (either temporal or complete) with thinning of the RNFL (either temporal or diffuse) and mild tessellated fundus changes around (Fig. 6A). The wide-field fundus autofluorescence of optic atrophy in F007-II:1 with an *ACO2* variant appeared normal at the initial visit (Supplementary Fig. 8). The fundi of *ACO2*-associated retinal degeneration patients featured complete pale optic discs, attenuated vessels, tessellated fundus changes and a dull retinal appearance (Fig. 6A). Fundus autofluorescence in patients with *ACO2*-associated retinal degenerations showed various degrees of change. The fundus autofluorescence of F008-II:1 exhibited diffuse hypoautofluorescence with a ring-like hyperautofluorescence around the macula, whereas his sibling (F008-II:2), as well as F004-II:2, showed a ‘sand-beach-like’ hyper-autofluorescence arc in the mid-peripheral retina and around the vessel arcades (Fig. 6A and Supplementary Fig. 7). During the 1-year follow-up visits, neither *ACO2*-associated optic atrophy in F007-II:1 nor optic atrophy plus retinal degeneration in F004-II:2 exhibited obvious progression (Supplementary Fig. 7). However, the visual acuity of patient F004-II:2 had deteriorated from light perception to no light perception after 1 year.

The phenotypic spectrum of *ACO2* could be categorized into three distinct forms: optic atrophy, optic atrophy plus retinal pigmentosa and optic atrophy plus early-onset severe retinal dystrophy (EOSRD) or Leber congenital amaurosis (Fig. 6B). The hypomorphic c.1387G>T/p.(Gly463Trp) allele, which has a high population frequency, particularly in East Asia (0.55%, 110/19950, based on gnomAD), was responsible for the optic atrophy in four unrelated families. F008:II-1 and F008:II-2 carrying intermediate pathogenic variants were associated with optic atrophy plus retinal pigmentosa, while F004:II2 and F002:II1, carrying higher pathogenic variants, were associated with optic atrophy plus EOSRD/Leber congenital amaurosis (Fig. 6A and B). The *ACO2* variants pathogenicity scores from low to high correlated with phenotypes spanning from optic atrophy to severe retinal degeneration (Fig. 6B). These findings provide novel insight into molecular mechanisms by which this hypomorphic variant results in a mild phenotype.

#### NBAS

A boy (F035:II-2) presented with poor visual acuity and nystagmus in both eyes since birth, along with systemic symptoms such as abnormal liver function, short stature [1.52 m (−3.6 SD)] and a progeroid appearance; his elder sister experienced similar symptoms. Both siblings had biallelic *NBAS* mutations. When he was referred to the ophthalmic centre at 6 years old, fundus examination revealed tilted optic discs, diffuse optic atrophy, straightened vessels and notable tessellated fundus with additional findings of temporal dragging of the optic disc and macular dystrophy on OCT (Fig. 6C and Supplementary Fig. 9). The fundus of his elder sister also manifested optic atrophy; her optic discs showed a highly myopic appearance with tilted optic discs and the presence of advanced peripapillary atrophy (Fig. 6C and Supplementary Fig. 9).

#### SSBP1

All six patients from the three families harbouring pathogenic variants of *SSBP1* showed optic atrophy plus retinal degeneration—the relatively later-stage of the *SSBP1* chronological progression—at their initial visits at a median age of 5 years (Fig. 6D and Supplementary Fig. 10), and it seemed that the retinal degeneration of these patients was earlier than that in the previous report.^24^ Although diffuse pale optic disc and attenuated vessels were common features in these patients with *SSBP1* variants, available fundus autofluorescence from five patients showed variable changes (Fig. 6D and Supplementary Fig. 10). Increased autofluorescence in the macular region was present in individuals F133-II:2 and F133-II:3 on visits at ages 5 and 4 years, respectively, but absent in individuals F133-II:1 and F133-I:2. These patterns were similar to those at 5-year follow-up assessments in F133-I:2, F133-II:1 and F133-II:2 and 1-year follow-up in F133-II:3 (Fig. 6D and Supplementary Fig. 10). The reason for the earlier retinal degeneration changes for those with *SSBP1* variants in our cohort is not clear, but environmental factors as well as genetic background might contribute to the difference. Further studies with a larger cohort of individuals from different genetic backgrounds will help to clarify this.

#### Other genes

The carriers of *OPA3*, *RTN4IP1*, *FDXR* and *C12orf65* mutations in our cohort also manifested optical atrophy plus retinal degeneration. The shared fundus features of *OPA3*, *RTN4IP1* and *C12orf65* mutation carriers included pale, excavated optic disc, attenuated vessels, tapetoretinal degeneration and reduced electroretinogram responses. The fundus examination of the *OPA3* patients also showed non-uniform macular pigments. Complete pale optic disc, silver wiring of retinal vessels and salt-and-pepper retinal degeneration, mostly in the mid-peripheral area, were observed in our in-house patients carrying *FDXR* mutations.^15,16^

### Ocular phenotype spectrum in the literature

A total of 2267 reported families had documented ocular phenotypes, with *OPA1* (705, 31.10%), *WFS1* (459, 20.25%), *POLG* (459, 20.25%), *SPG7* (132, 5.82%) and *ACO2* (87, 3.84%) being the predominant genes (Supplementary Fig. 3). The reported ocular phenotypes varied from optic atrophy to retinal degeneration, ptosis, saccadic eye movement, nystagmus, strabismus, cataracts and colour vision defects.

**Figure 7:**
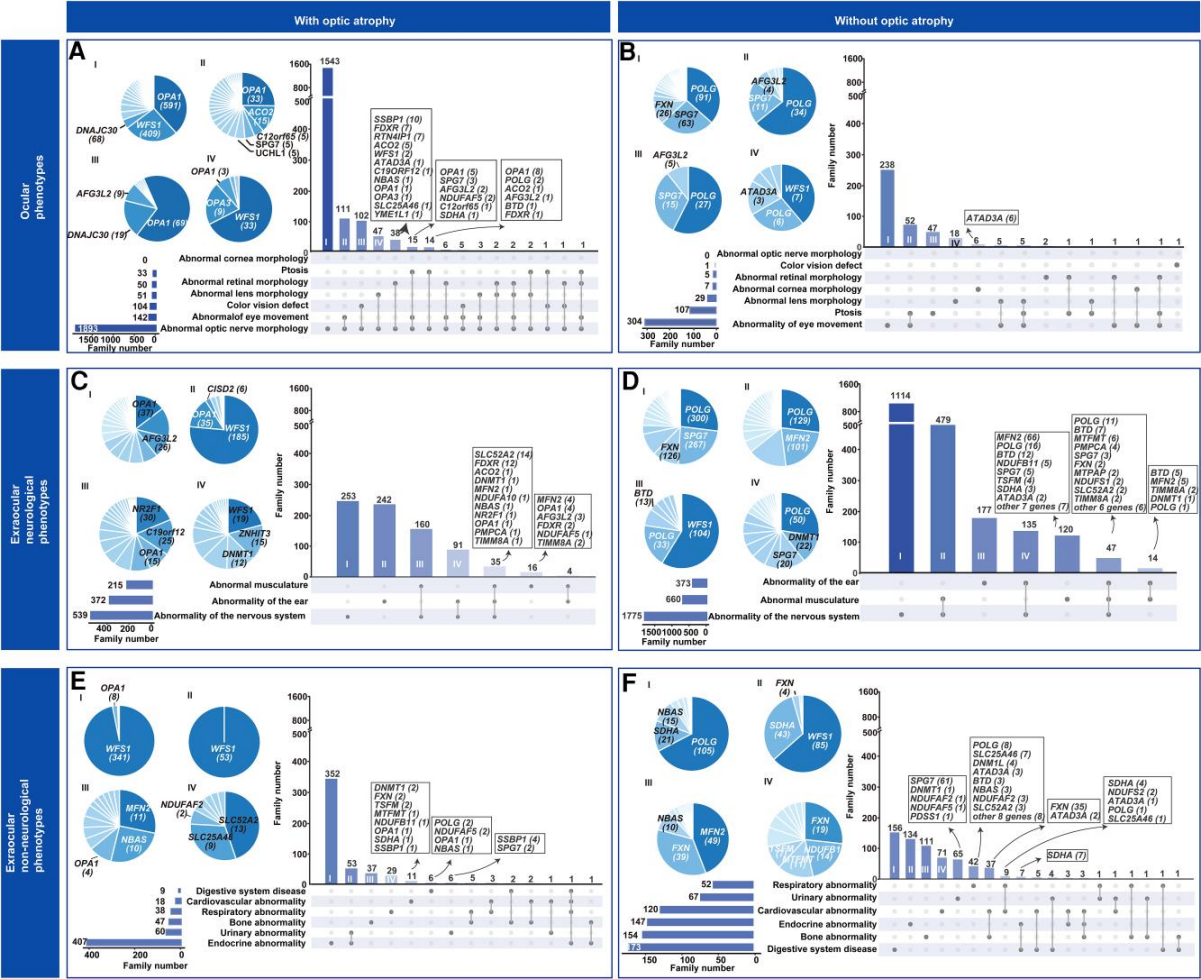
**Modified Venn diagram of the intricate intersection between ocular and extra-ocular phenotypes in patients with or without optic atrophy**. (**A**) Distribution of ocular phenotypes in patients presenting with optic atrophy. (**B**) Distribution of extra-ocular neurological phenotypes in patients presenting with optic atrophy. (**C**) Distribution of extra-ocular non-neurological phenotypes in patients manifesting optic atrophy. (**D**) Distribution of ocular phenotypes associated with gene variants for which the main clinical feature is not optic nerve atrophy. (**E**) Distribution of extra-ocular neurological phenotypes associated with gene variants for which the main clinical feature is not optic nerve atrophy. (**F**) Distribution of extra-ocular non-neurological phenotypes associated with gene variants for which the main clinical feature is not optic nerve atrophy. The pie charts and boxed gene lists in **A**–**F** show the abundances of the genes associated with the phenotype; I–IV on the pie charts (*left*) correspond to I–IV on the bar graphs (*right*). Vertical bars illustrate the number of overlaps between phenotypes in the datasets (*bottom*), which are further specified by solid black circles; the grey lines indicate overlaps between multiple phenotypes. The total number of families displaying each phenotype is indicated to the *left* of the matrix.

To elucidate the spectrum of genetically related ocular phenotypes, with or without optic nerve atrophy as a major clinical feature, a modified Venn diagram was used to visualize the intricate intersection of ocular phenotypic relationships in these two groups of patients (Fig. 7). Abnormal eye movements were the most prevalent concomitant ocular phenotype in both patient groups, irrespective of the presence of optic nerve atrophy. Specifically, abnormal eye movements, such as nystagmus, strabismus and external ophthalmoplegia, were documented in 19.85% of families with ocular involvement (Fig. 7 and Supplementary Fig. 2). Of the 50 genes associated with optic nerve atrophy, 43 have been documented to contribute to abnormal eye movements in at least one affected pedigree, and the most predominant of these were *POLG* and *SPG7.* Patients with biallelic variants in *POLG* showed a predominant phenotype of external ophthalmoplegia but rarely optic atrophy (Supplementary Fig. 11).^25,26^ For patients with biallelic variants of *SPG7*, progressive weakness and spasticity of the lower limbs was a typical sign, and optic atrophy was present in only a subgroup of these patients (Supplementary Fig. 11).^27,28^ These two genes were among the top five contributors of total families and ocular-involved families (Supplementary Fig. 3A and B), but these were uncommonly found in the literature on optic atrophy and not found at all in our in-house cohort with a predominant phenotype of optic atrophy.

Colour vision defects are frequent in patients with optic atrophy but rare in those without it; and retinal dystrophy contributes minimally to both groups, representing 2.47% of all ocular-involved families (Fig. 7). The predominant genes for retinal dystrophy are *FDXR*, *SSBP1*, *ACO2* and *RTN4IP1* (Supplementary Fig. 3C). Retinal dystrophy with attenuated, silver-like retinal vessels was found to be a unique clinical characteristic of *FDXR* mutation carriers, with most patients displaying a hyper-autofluorescence ring around the fovea. Heterozygous mutations in *SSBP1* could lead to retinal vessel attenuation, abnormal appearance of the fovea and variable pigmentary retinal changes.

Ptosis occurred in 1.74% of patients with, and 28.88% of those without, optic atrophy, mainly those showing variants in *POLG*, *SPG7* and *AFG3L2*. Abnormal lens morphology—mainly cataracts, was present in 8% of patients with or without optic atrophy and was mainly contributed by Wolfram syndrome or isolated cataracts caused by *WFS1* variants. Colour vision defects were most frequently linked to *OPA1* (69 families), followed by *DNAJC30* (34 families) (Fig. 7). Corneal opacity, reported in seven families, was mainly contributed by variants in *ATAD3A* (Fig. 7).

### Extra-ocular neurological and non-neurological phenotype spectrum in the literature

**Figure 8:**
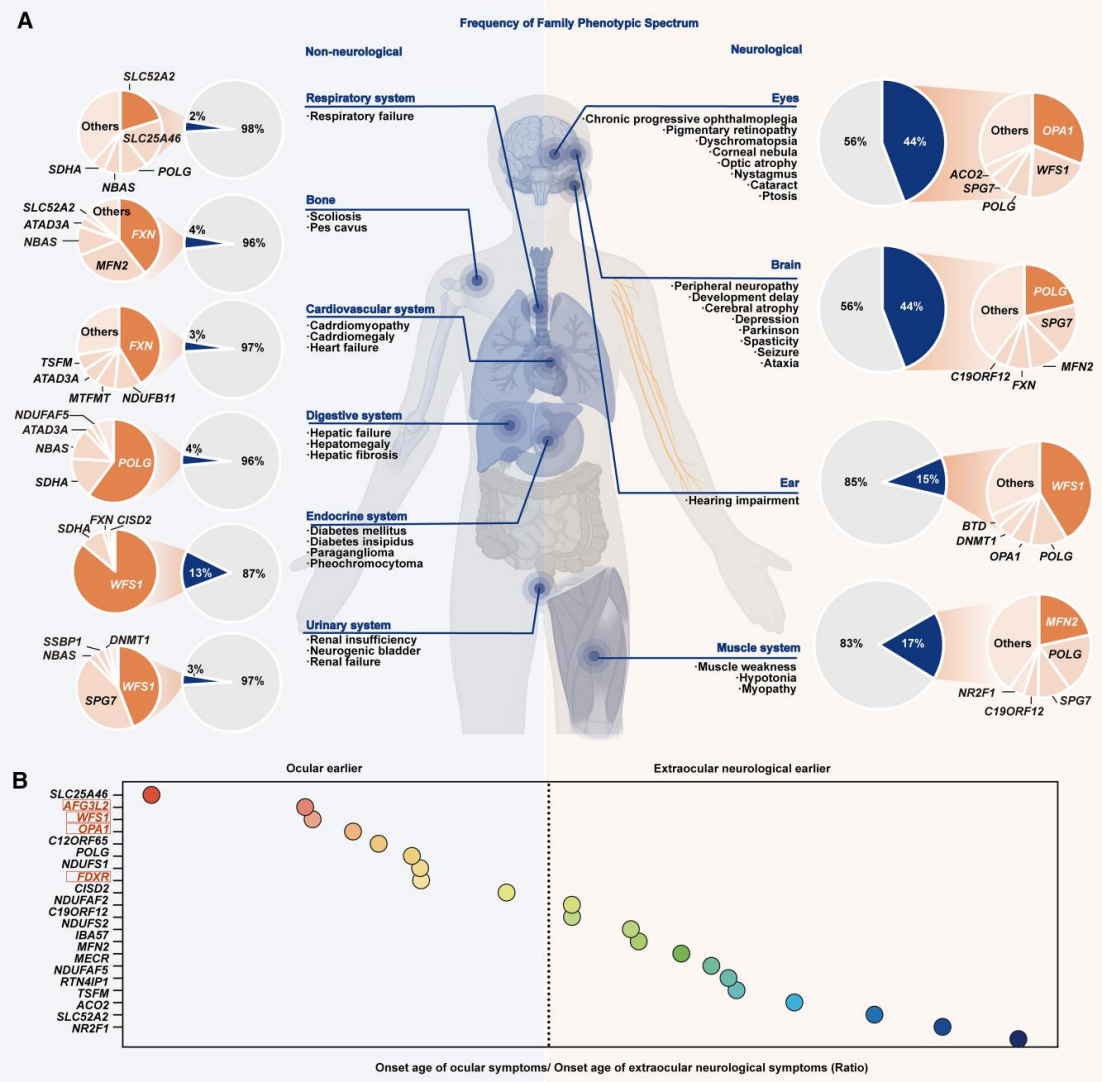
**Frequency distribution of the phenotypic spectrum of hereditary optic neuropathy-related nuclear genes in literature and comparison of age at onset of ocular symptoms and neurological symptom age of onset for each gene.** (**A**) Non-neurological groups on the *left* and neurological groups on the *right*. The grey shaded segments of the pie charts represent the phenotypes that are either normal or unidentified across all reported families, while the percentage of the associated phenotype is indicated by the blue shaded segments. In the expanded pie charts, the dark orange segments note the genes most linked to the phenotype, highlighting their prevalence. The central portion of the human body illustration was created and authorized by Biorender.com. (**B**) The gene names in red are among the top five genes associated with the in-house nuclear gene-related hereditary optic neuropathy (nHON) cohort. The boxed gene names indicate statistical significance between the onset age of ocular symptoms and onset age of neurological symptoms reported in the literature.

Since mitochondria are essential in all human cells, mitochondrial dysfunction and oxidative stress can precipitate a range of neurological and non-neurological manifestations (Figs 7 and 8). In this study, HPO terms were used to record the phenotypes of patients reported in the literature.

#### Neurological presentations

Abnormalities of the nervous system, including neurodevelopmental abnormality (HP:0012759), ataxia (HP:0001251), seizure (HP:0001250), peripheral neuropathy (HP:0009830) etc., were present in 44% of the families reported, and the top five contributing genes were *POLG*, *SPG7*, *MFN2*, *FXN* and *C19ORF12* (Fig. 8). Seizure is a major manifestation in children with *POLG* mutations,^29-31^ while parkinsonism is more commonly noted in affected adults (Fig. 8).^32,33^*SPG7* mutations can result in spasticity paraplegia associated with ataxia, subclinical myopathy or ophthalmoplegia.^34,35^ Mutations in *MFN2* are associated with Charcot-Marie-Tooth disease, mainly manifesting as peripheral neuropathy, distal muscle weakness and atrophy, which can be complicated by optic atrophy.^36^ Homozygous (GAA)_n_ expansion or compound heterozygous *FXN* mutations cause Friedreich’s ataxia, the most prevalent hereditary ataxia overall (Figs 7 and 8).

Hearing impairment, which affected nearly 15% of the reported families, was mainly observed in Wolfram syndrome and autosomal dominant hearing impairment caused by *WFS1* variants, followed by variants of *POLG*, *OPA1*, *DNMT1* and *SLC25A46*. Muscle system abnormalities, including muscle weakness and hypotonia, were noted in nearly 17% of families and were predominantly associated with *MFN2*, *POLG*, *SPG7*, *C19ORF12* and *NR2F1* (Figs 7 and 8).

For patients whose reports detailed the onset ages of ocular and neurological symptoms, the age of onset of ocular symptoms and neurological disorders by gene were compared. (Supplementary Fig. 12). Ocular symptoms preceded neurological symptoms in syndromic HON patients with *SLC25A46* (*P* = 0.02)*, WFS1* (*P* = 4.90 × 10^−7^), *OPA1* (*P* = 3.01 × 10^−5^) and *POLG* (*P* = 2.00 × 10^−4^) variants, whereas neurological symptoms appeared earlier in patients with *NR2F1* (*P* = 0.03) and *ACO2* (*P* = 0.03) variants (Supplementary Fig. 12). From the literature review, patients harbouring variants of the top four genes associated with the in-house nHON cohort, including *OPA1*, *WFS1*, *FDXR* and *AFG3L2*, exhibited ophthalmic symptoms prior to neurological symptoms and were most frequently seen in our Pediatric and Genetic Eye Clinic. Active surveillance of extraocular neurological symptoms should be carried out in these during follow-up. No specific variant of each gene was detected in association with isolated optic atrophy or syndromic optic atrophy in these patients. (Fig. 8).

### Non-neurological features

Management of syndromic-optic atrophy is complicated by the recognition that multiple organs, such as the liver, lung, heart and kidneys, can be involved, which may show up as typical or atypical clinical presentations. Most of the studies reported organ-involvement associated with one gene, but there are limited investigations on the distribution of disease-related genes associated with specific organs.

The non-neurological phenotypic spectrum in syndromic optic atrophy includes abnormalities involving the endocrine, respiratory, skeletal, cardiovascular, digestive and urinary systems. Regarding extraocular non-neurological abnormalities, endocrine disorders primarily due to *WFS1* mutations are the most common in patients with optic atrophy, while digestive abnormalities, such as liver failure (primarily linked to *POLG* and *NBAS* mutations) and gastrointestinal stromal tumours (primarily linked to *SDHA* mutations), are predominant in patients without optic atrophy. (Fig. 7 and 8)

## Discussion

The neural retina stands at the forefront of neurology and ophthalmology, shaping the future of numerous rare neurogenetic diseases. Comprehensive insights from an ophthalmic perspective are relatively limited, particularly in the genetic and clinical landscape of HON in real-world practice and genotype-phenotype correlations. Here, the genetic and clinical spectra of HON from both macro and micro perspectives were systematically analysed utilizing both our in-house dataset and an extensive literature review.

*OPA1* and *WFS1* were the top two genes contributing most to nHON in our in-house cohort and reported literature, and were evident in the reports of real-world practice in French and Italian HON cohorts (Supplementary Table 4).^18,19^ However, the proportion of patients associated with *OPA1* and *WFS1* variants differed between the in-house cohort and literature review, which might be attributed to the variant interpretation of these two genes in different studies (Supplementary Table 5). The predominant inheritance pattern of *WFS1* and *OPA1* varied significantly (*P* < 0.0001). In the in-house HON families with *OPA1* variants, autosomal dominant patterns were found in 77 of 82 (93.90%) families and autosomal recessive patterns in 5 of 82 (6.10%), while for *WFS1*, autosomal dominant patterns were found in 2 of 15 (13.33%) pedigrees and autosomal recessive patterns in 15 of 17 (88.24%). The distribution of *OPA1* and *WFS1* variants in the in-house data, the gnomAD database and available data from published literature are presented in Supplementary Figs 13 and 14, respectively. In-house *OPA1* pathogenic/likely pathogenic missense variants leading to an autosomal dominant inheritance pattern were predicted to be pathogenic in at least three of five *in silico* prediction tools (Supplementary Fig. 13). Thirty-five of 38 (92.11%) truncations classified as pathogenic/likely pathogenic in *OPA1* were associated with autosomal dominant patterns (Supplementary Fig. 13), while no heterozygous truncation associated with autosomal dominant patterns was detected in *WFS1* (Supplementary Fig. 14). The heterozygous truncation variant of *WFS1* was not considered to be dominant pathogenic for Wolfram syndrome 1 for the following reasons: (i) heterozygous truncation variants were also present in healthy individuals (father of F152:II-1 and father of F147:II-1) and in-house healthy normal controls; (ii) truncating variants of *WFS1* were frequently seen in the gnomAD database but were not clustered in patients with HON (Supplementary Fig. 9); and (iii) the Loss Intolerance probability (pLI) for *WFS1* was zero. Previous studies of *WFS1* have indicated that deep intronic variants and copy number variants could explain unsolved cases with a monoallelic variant of *WFS1*, which underscores the need to complete full sequencing of *WFS1* and whole-genome sequencing in unsolved cases.^37^

No pathogenic variants in *DNAJC30* were detected in our cohort. It has been suggested that the homozygous variant (p.Tyr51Cys) is responsible for up to 27% of genetically diagnosed LHON families in the founder population of Eastern Europe and up to 5% of families in non-founder populations.^38,39^ This suggests a gradient influenced by proximity to the founder event’s geographic location.^38^ Therefore, possible ethnicity-specific differences in *DNAJC30* might exist between East Asians and Eastern Europeans.

Previous studies have indicated that variants in *SSBP1*, *ACO2*, *RTN4IP1*, *NBAS*, *OPA3* and *FDXR* could manifest a concurrent occurrence of retinal degeneration in the fundus along with optic atrophy.^15,16,40-45^ Biallelic mutations in nuclear genes such as *DNAJC30*, *NDUFS2*, *NDUFAF5*, *NDUFA12*, *MECR* and *MCAT* have been identified in unsolved cases with typical LHON-like phenotypes.^10-14^ However, the systematic summary of the fundus characteristics of nHON is limited. To the best of our knowledge, this is the first study to summarize and characterize fundus phenotypes associated with nHON and propose three fundus categories. Nearly 63.64% (7/11) of eyes from six nHON patients with LHON-like fundus characteristics were in the C1/C2 category at their first visit, an obvious distinction from the optic atrophy phenotype. Two distinct patterns of vision loss were also noticed in our patient records for those with non-syndromic optic atrophy: (i) insidious onset; and (ii) acute or subacute onset. *OPA1*-associated HON showed insidious onset of visual impairment. Of the 87 patients with *OPA1* variants in our cohort, 64 (73.56%) were identified as having impaired vision during routine examinations, and a presumed onset age was defined by either the earliest time of medical documentation or the self-reported time of visual loss noticed. Unclear onset information was obtained from the remaining 23 patients (Supplementary Table 3). Therefore, the age of onset for *OPA1*-associated HON is more likely the age at diagnosis, due to the insidious nature of disease onset. However, the age of onset for this disease is still used to be consistent with previously published literature. The median onset age of visual impairment in patients with *OPA1* variants was 6.00 (1.75, 9.00) years in our in-house cohort, which was significantly earlier than that in the overall literature on *OPA1*-related age of disease onset but was not significantly different from that in large, published cohorts (Supplementary Fig. 15). Most cases of visual impairment caused by *WFS1* variants are detected through eye examinations conducted after the diagnosis of diabetes mellitus or diabetic ketoacidosis in adolescents and genetic testing that confirms the presence of pathogenic variants in the *WFS1* gene. In-house patients presented acute or subacute vision loss, predominantly those patients with variants in *MCAT*, *NDUFAF5*, *NDUFS1* and *NDUFA10*, making it difficult to differentiate LHON. Most of the suspected HON cases could be solved by thorough clinical assessment, including a careful evaluation of family history, knowledge of environmental exposure, pattern of onset, ophthalmic characteristics, other organ involvement and, eventually, genetic screening.

In our cohort, 29 patients with optic atrophy plus retinal degeneration were classified according to fundus changes with or without electroretinography data. Of the 29 patients, electroretinography data were available for 15. Although fundus changes associated with retinal degeneration were present in all 15 patients, they showed variable electroretinography results: 10 patients had a severely reduced to undetectable waveform of both rod and cone responses; four patients indicated moderately reduced rod and cone responses; and one patient exhibited a normal rod response with a moderately reduced cone response (Supplementary Table 3). It has been long maintained that the hallmark of LHON and *OPA1* variants is retinal ganglion cell degeneration, with normal full-field electroretinography.^46,47^ However, previous studies have revealed the average amplitude of oscillatory potentials, the amplitude of maximal response and single flash photopic electroretinography in *OPA1* patients were smaller than those in controls, with the average amplitude of oscillatory potentials approximately half the average amplitude seen in controls.^48,49^ Additionally, five of seven LHON individuals had full-field electroretinography abnormalities; two of these patients exhibited significantly reduced amplitudes in photopic response and lower amplitudes in the scotopic response, despite their outer retinal layers (such as the inner segment, outer segment and retinal pigment epithelium) showing no detectable structural changes on spectral-domain OCT.^49^ In fact, it is not unusual that the electroretinography results do not correspond to the fundus changes associated with retinal degeneration. In our previous studies, 14 patients carrying *ABCA4* variants with yellowish flecks at the posterior fundus and a patient carrying *NPHP1* variants with diffused tapetoretinal degeneration and arteriolar attenuation exhibited relatively normal electroretinography responses.^50,51^ The inconsistent results between fundus appearance and electroretinography response may be due to the limited power of electroretinography to detect changes confined to the macular region, isolated area or far peripheral retinal regions. Therefore, electroretinography was not conducted for all the patients in our cohort. Based on previous genotype-related fundus manifestations, the categorization of fundus manifestations according to direct fundoscopy, fundus photography or autofluorescence imaging in this study remains a relatively reliable and straightforward method for categorizing HON with or without retinal degeneration.

In our cohort of patients with a clinical diagnosis of suspected HON caused by nuclear gene mutations, the diagnostic rate of nHON was 31.50%, which aligns closely with international findings, where rates were reported to be 27%^19^ and 36.9%.^18^ Looking forwards, our research aims to incorporate comprehensive mitochondrial sequencing for those patients with a negative nuclear gene diagnosis and four primary LHON variants, particularly to rule out cases of childhood-onset LHON in children whose parents may not recognize early visual impairment, yet who exhibit complete optic nerve atrophy at clinical presentation. The low percentage of nuclear genes in patients with suspected LHON-like disease suggests that the number of nuclear genes that can cause the LHON-like phenotype is relatively small and that the LHON-like phenotype is more likely to be the result of a combination of mitochondrial DNA mutations and the environment.

Establishing the genetic diagnosis of mitochondrial-related eye disease can be difficult because mitochondrial constituents are encoded by both nuclear and mitochondrial genes. Since March 2024, whole mitochondrial genome sequencing with WES or whole-genome sequencing have been combined in our routine clinical practice for the diagnosis of genetic eye diseases. It is believed that pooled WES + mitochondrial DNA sequencing and whole-genome + mitochondrial DNA sequencing data will advance the understanding of the pathogenicity and regulatory functions of mitochondrial and nuclear mutations not only in retinal disease but also in cataract, corneal and muscle diseases, among others.

Recently, the most common point mutation, G11778A of LHON, has been reported to have a high prevalence (about 2/1000) in healthy populations, suggesting that this LHON-associated variant has a very low penetrance (the prevalence of LHON is around 3/100 000).^5^ However, significant enrichment of this variant has been observed in 84.5% of our in-house patients with LHON.^23^ In our attempts to explore possible incomplete penetrance in nHON-associated variants, we encountered a few family members harbouring pathogenic variants in *OPA1* who did not have signs and symptoms of optic atrophy, in whom incomplete penetrance is a possible explanation. On the other hand, the following problems have also been observed: (i) some parents harbouring pathogenic *OPA1* variants claimed that they were normal, however, careful ocular examination revealed mild-to-moderate reduced visual acuity, localized atrophy of the optic disc and thinning RNFLs in the atrophic region on OCT scan; (ii) it is possible that the interpretation of some variants in genes causing both dominant and recessive phenotypes is inaccurate, for example, heterozygous truncating mutations in *WFS1* have been reported to cause nHON but the pathogenicity of such variants might be questionable due to normal phenotype in carrier parents, extremely high frequency in general population (pLI = 0) and presence in a subset of controls and related controls. Additional studies are needed to clarify whether this is the result of incomplete inheritance or due to inaccurate interpretation of the variants.

Education of primary care physicians, ophthalmologists, patients and patient families is especially important because of the insidious onset of vision loss in most nHON cases. Specifically, knowledge of the pertinent ocular signs and symptoms (e.g. nystagmus, strabismus and cataract) and extraocular symptoms (e.g. hearing loss, ataxia, seizure, developmental delay) is paramount. Extraocular symptoms are often not fully identified at the initial ophthalmological visit and should be discussed with the patients and families. Furthermore, non-syndromic optic atrophy should be followed-up regularly for timely detection of any new extraocular complications.

There are limitations to our study. The extraocular neurological examinations of our in-house cohort might be insufficient. The top four genes associated with nHON in our in-house cohort correlated with the earlier appearance of ophthalmic symptoms over neurological symptoms based on the literature review, which is the reason these patients were most often received in the Pediatric and Genetic Eye Clinic. As certain extraocular neurological manifestations are often subtle or absent in early childhood, typically becoming more apparent during adolescence, genetic testing in young children may serve as an early warning marker for these conditions, facilitating timely intervention and management. Nevertheless, the progression and transition of these clinical manifestations require further longitudinal observation to fully understand their developmental trajectory. A multidisciplinary, collaborative and inter-institutional approach is warranted to make rapid progress in the diagnosis and treatment of nHON.

To date, no definitive treatments for nHON have been established, although studies on the use of idebenone, gene modifying therapies and stem cell therapy in HON-nDNA are ongoing.^52-55^ Diagnosis of hereditary optic atrophy is challenging for ophthalmologists, especially when combined with retinal dystrophy, as the optic nerve may have variable degrees of pallor. This study augments our understanding of the genetic aetiology of optic atrophy, its ocular manifestations and the corresponding visual prognoses in the context of genetic defects, which is essential to disease recognition and expediting diagnosis, targeted treatment, and providing genetic and prognostic counselling. Knowledge about the disease course will ensure appropriate monitoring of the effects of defect-specific therapies, the next frontier of HON research.

## Supplementary Material

awae324_Supplementary_Data

## Data Availability

Additional data reported in this paper are available upon reasonable request.
