# Supplementary material for: Clinical and genetic landscape of optic atrophy in 826 families: insights from 50 nuclear genes
Source: Brain. 2024 Oct 18;148(5):1604–20. doi: 10.1093/brain/awae324 (PMC12073998; doi:10.1093/brain/awae324)
Supplement: awae324_Supplementary_Data [file awae324_supplementary_data.zip › brain-2024-01629-File010-v2.pdf]

**Figure S1**

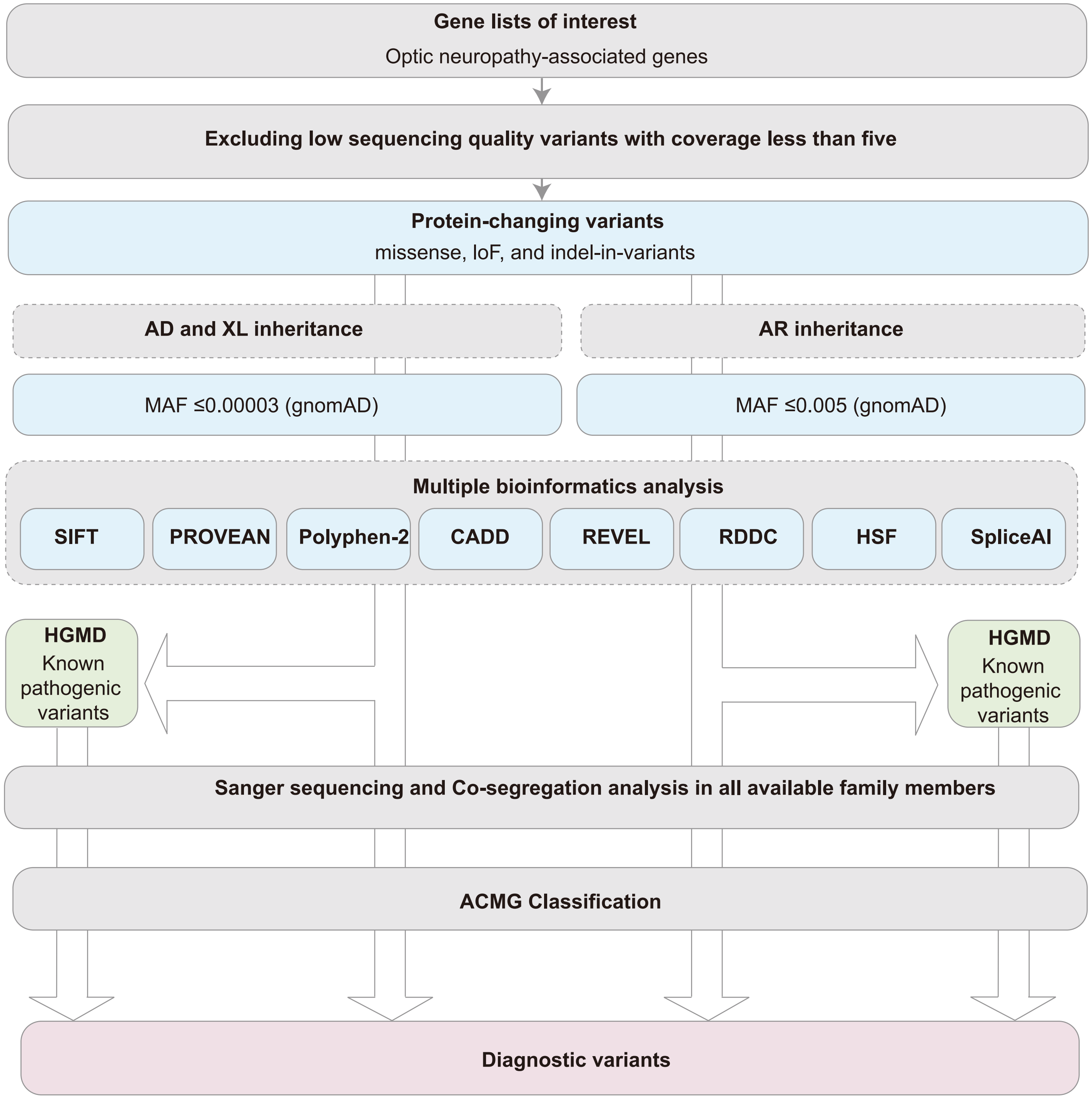


**Supplementary Figure 1. Workflow overview of multistep bioinformatics analysis in this study identifying HON-related nuclear gene variants.** As the prevalence of dominant optic atrophy was 1 in 35000 in North England (PMID 21112411), the threshold for the potential pathogenic variants in dominant inheritance was expected to be less than 0.00003, and in recessive inheritance was expected to be less than 0.005.

**Figure S2**

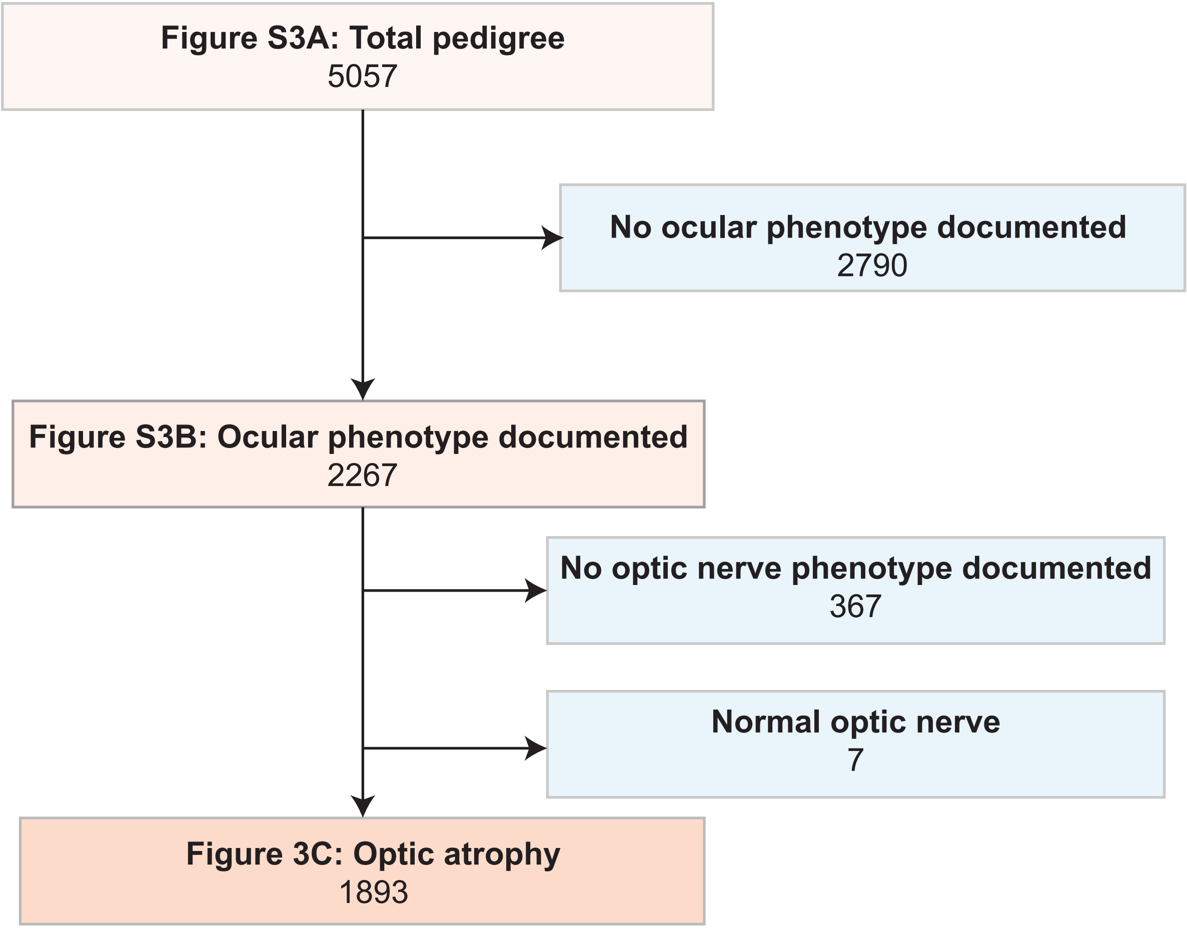


**Supplementary Figure 2. Schematic Overview of Literature Review Workflow to to Identify Pedigrees relevant to Optic Atrophy.** To date, pathogenic variants associated with hereditary optic neuropathy (HON) in nuclear genes have been identified in 5,057 families. Upon exclusion of 2,790 families lacking documented ocular phenotypes, the remaining 2,267 families were analyzed for optic nerve characteristics. Within this cohort, 367 families had no recorded optic nerve phenotypes, 7 families displayed a normal optic nerve upon evaluation, and 1,893 families were documented to have optic nerve atrophy.

# Figure S3

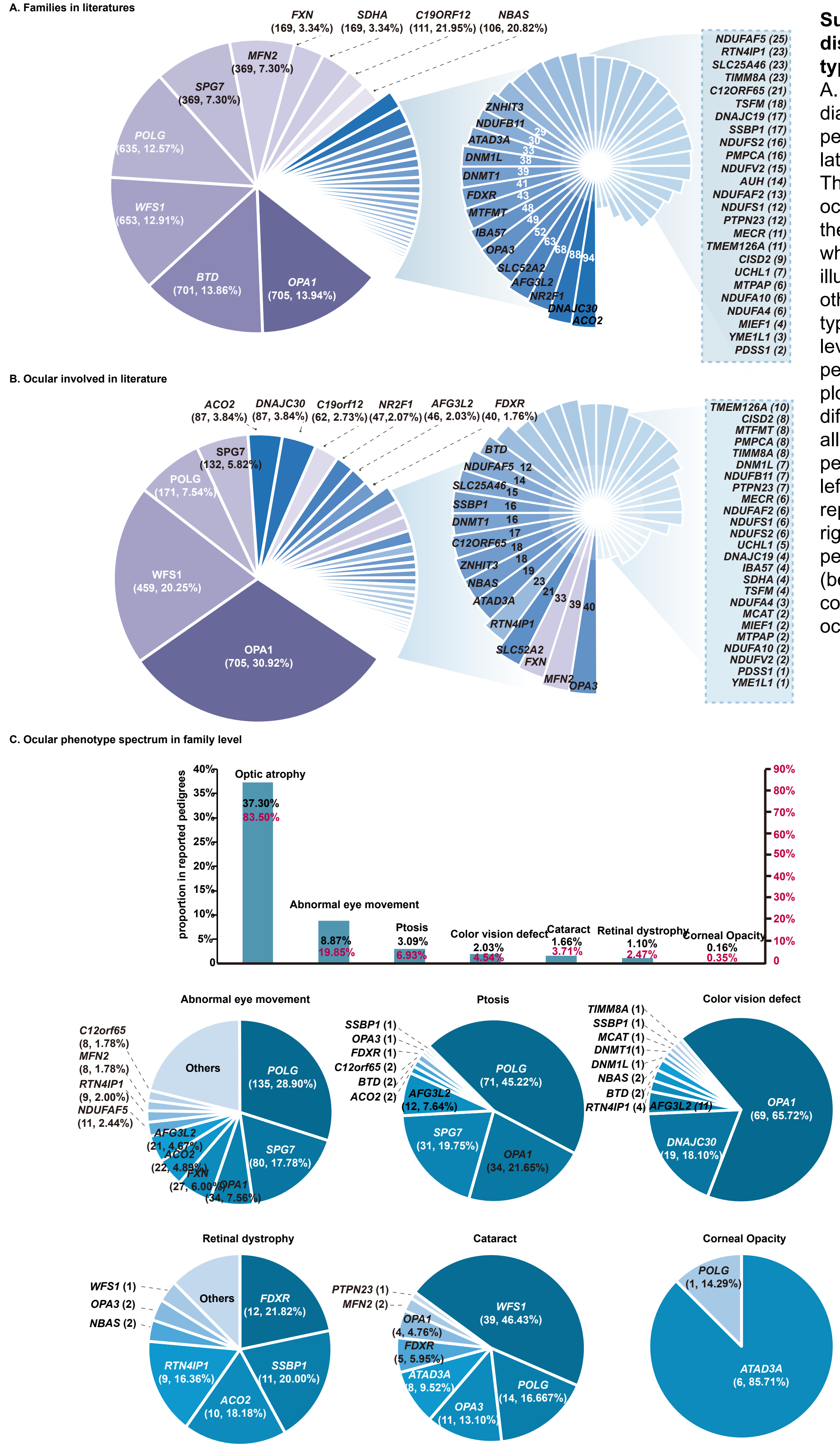


**Supplementary Figure 3. The distribution of ocular phenotype reported in the literature.** A. The pie chart and the rose diagram display all the reported pedigrees caused by HON-related nuclear gene mutations B. The pie chart displays the ocular phenotype involved in the ten most common genes, while the rose diagram illustrates the distribution of others. C. The ocular phenotype spectrum at the family level shows the reported pedigrees with nHON. The bar plot(top) shows the frequency of different ocular phenotypes in all reported and ocular-involved pedigrees. The Y-axis on the left is for proportion in all reported pedigrees, while the right is for all ocular-involved pedigrees. The pie chart (below) shows causative genes' contribution to each type of ocular phenotype.

Figure S4

A. Sectoral pRNFL thickness in patients manifested OA

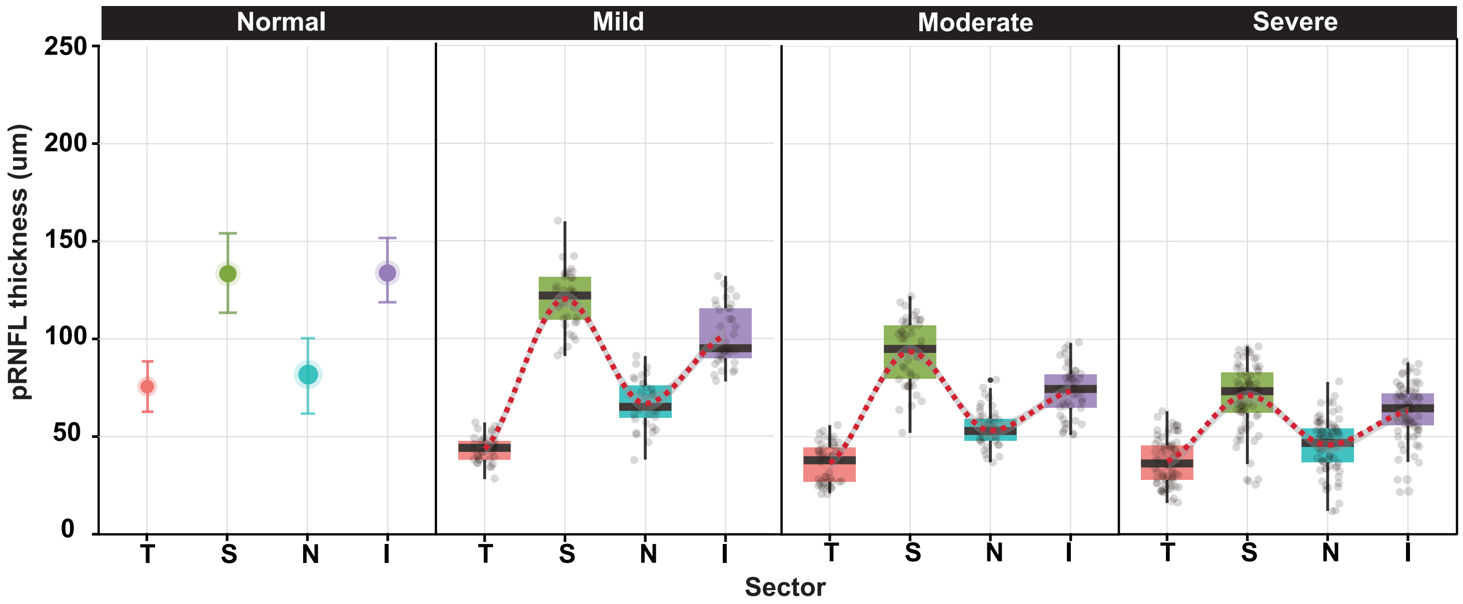


B. Sectoral pRNFL thickness in patients manifested LHON-like

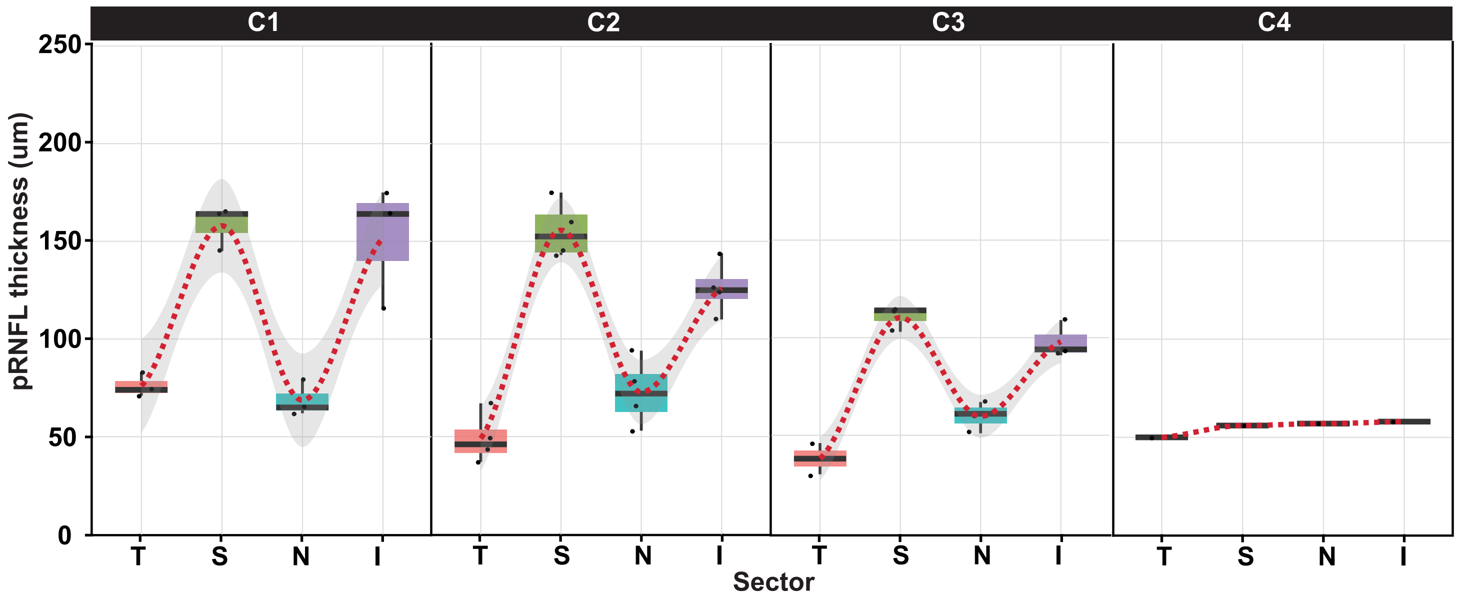


C. Sectoral pRNFL thickness in patients manifested OA+RD

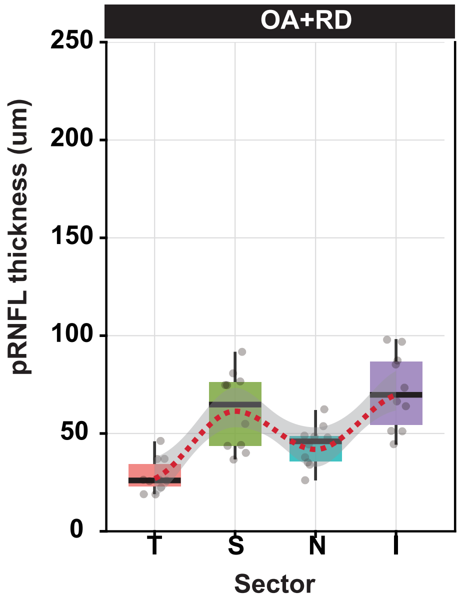


**Supplementary Figure 4.** A. Association between the sectoral peripapillary retinal nerve fiber layer thickness and stage of OA phases. Superior and inferior sectoral retinal nerve fiber layer thickness was most reduced in the severe phase, followed by the moderate phase, especially when compared with mild phases B. Association between the sectoral peripapillary retinal nerve fiber layer thickness and stage of LHON-like. C. Sectoral peripapillary retinal nerve fiber layer thickness in OA+RD. Black bars indicate median values, Error bars, from minimum to maximum. I, Inferior; N, Nasal; S, Superior; T, Temporal.

Figure S5

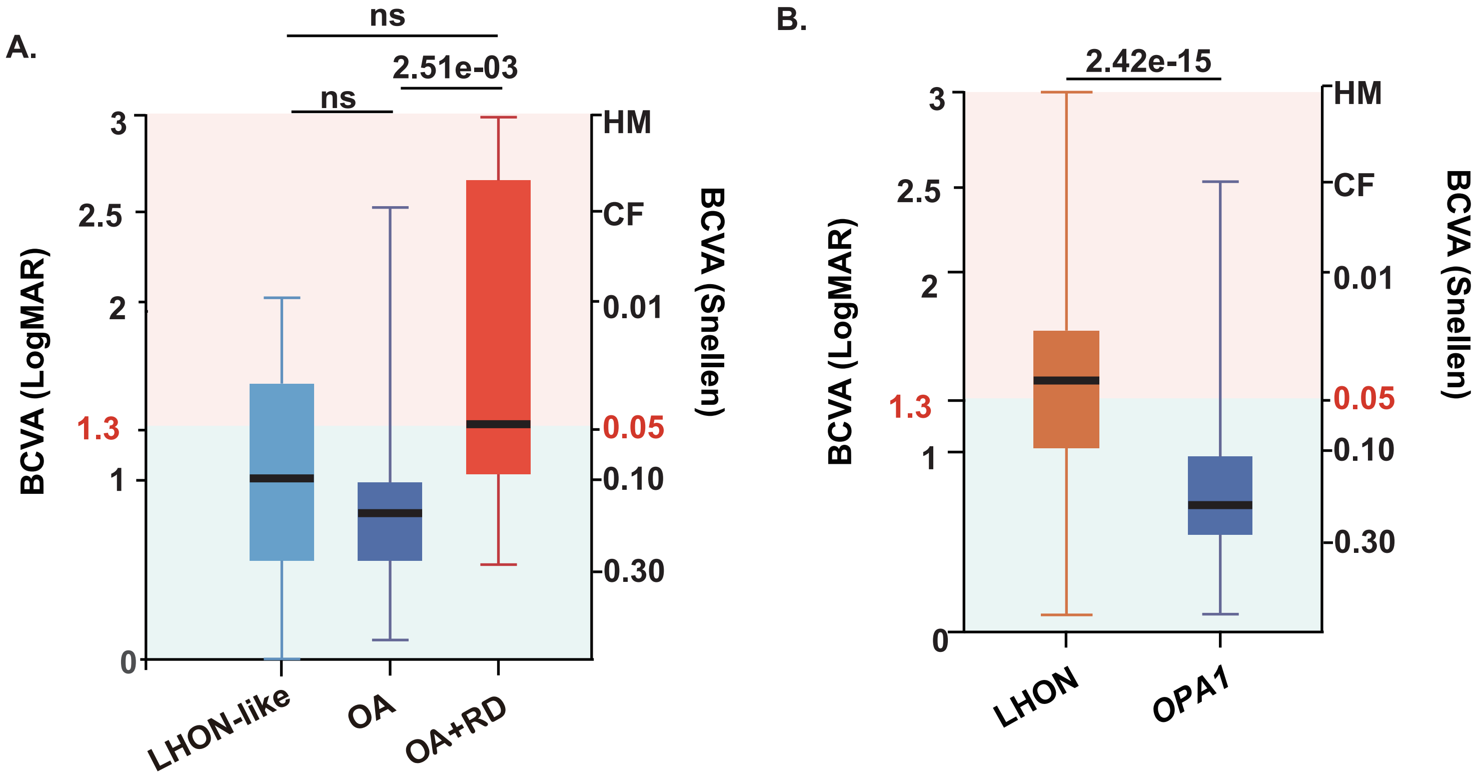


**Supplementary Figure** 5. A. The comparison of BCVA among LHON-like, OA, and OA plus retinal degeneration. The Kruskal–Wallis test with Dunn post hoc tests (R package FSA) was adopted to compare BCVA in different groups. ****, adjusted-p=2.51e-03. B. The comparison of BCVA between LHON and OPA1. The box plots indicating 25th, 50th and 75th percentiles were generated by R package ggplot2. ****, p=2.42e-15. Count finger was converted to 2.5 in LogMAR; Hand motion was converted to 3 in LogMAR; BCVA, Best-corrected visual acuity; CF, Count finger; HM, Hand motion; LogMAR, logarithm of the minimum angle of resolution; An adjusted p-value less than 0.05 was considered statistically significant. Four asterisks indicated an adjusted p-value less than 0.0001.

Figure S6

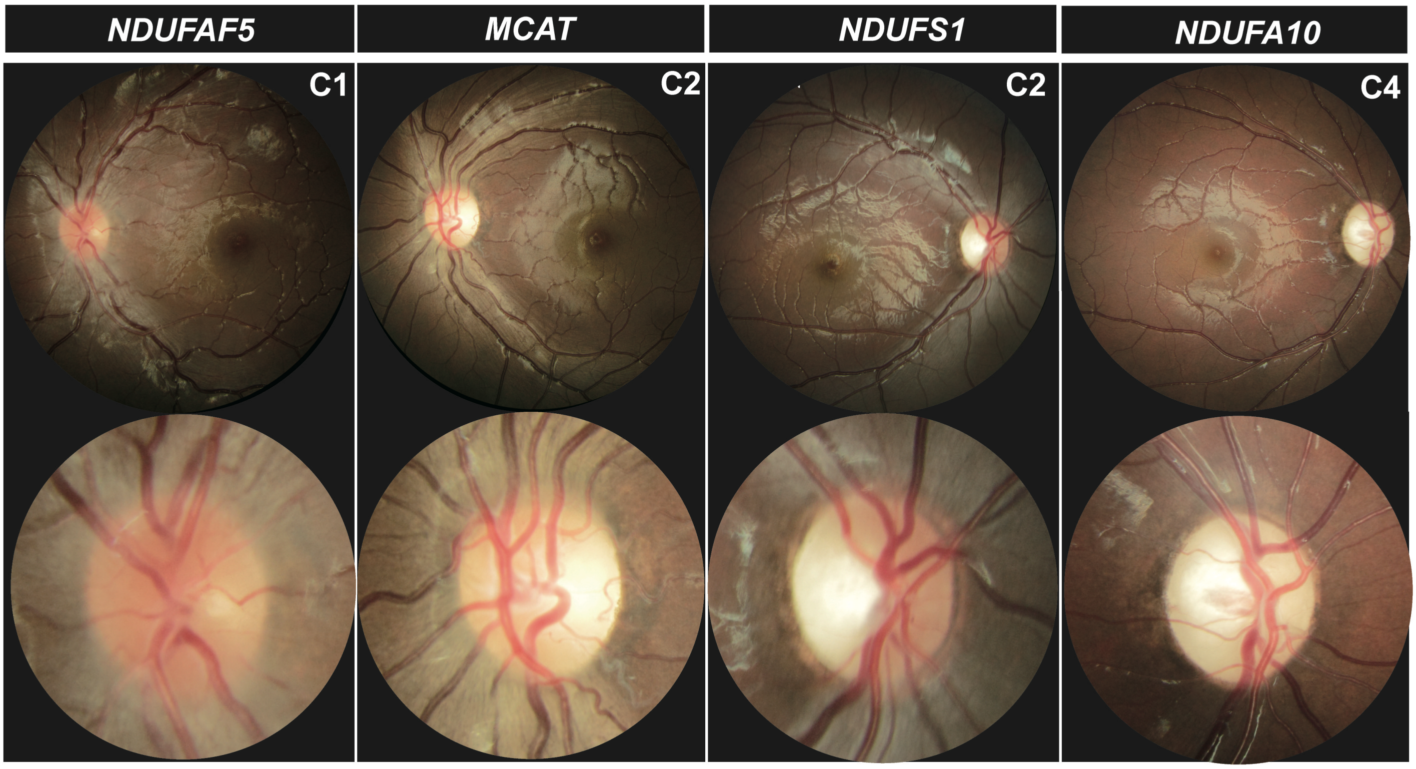


**Supplementary Figure 6. Fundus images in patients with LHON-like phenotypes**

Figure S7

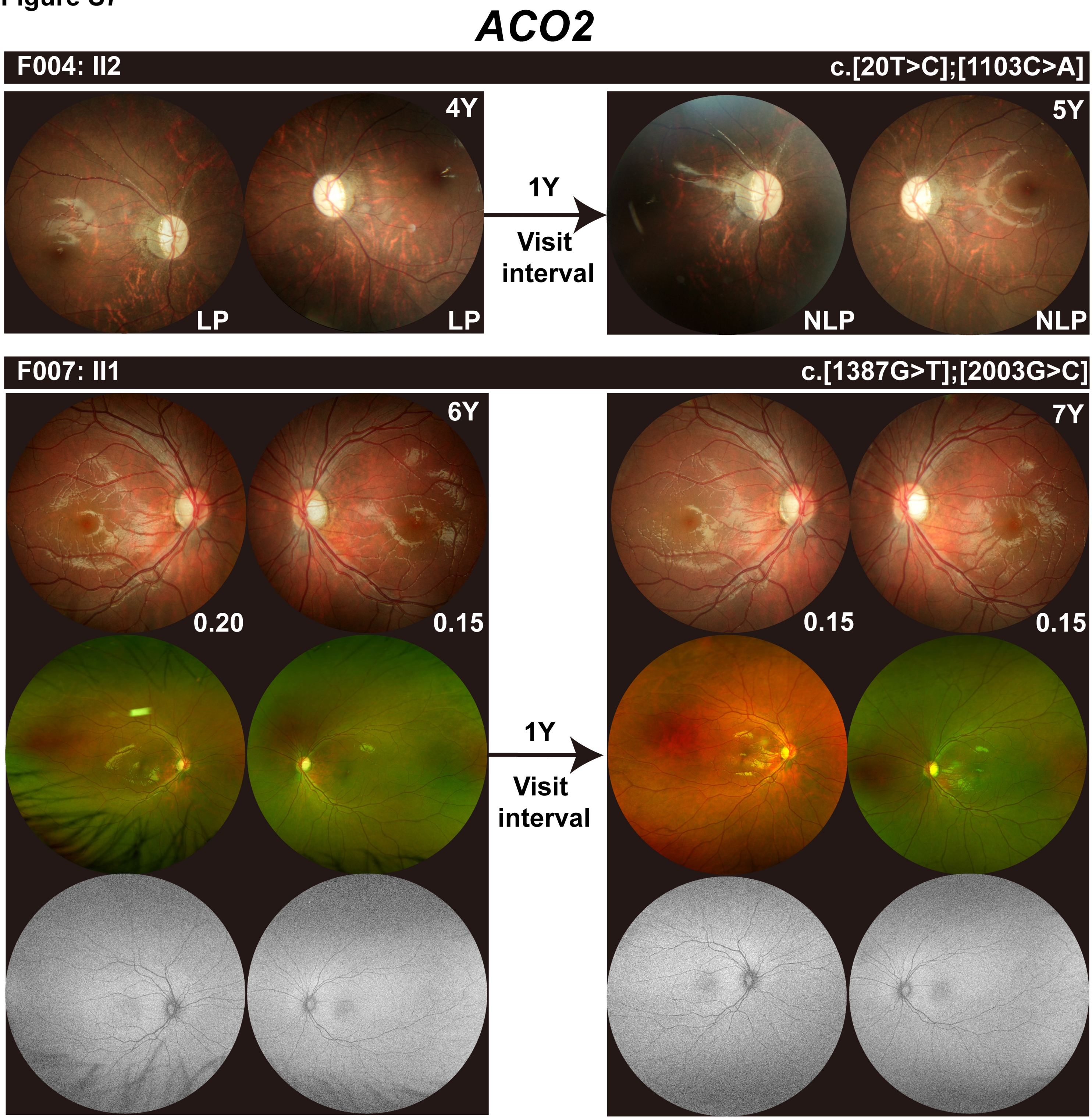


**Supplementary Figure 7. The follow-up of two patients with ACO2 variants.** The top left and bottom right corners of the fundus photographs show the patient's age and visual acuity at the time of the respective examinations. LP, light perception; NLP, no light perception.

Figure S8

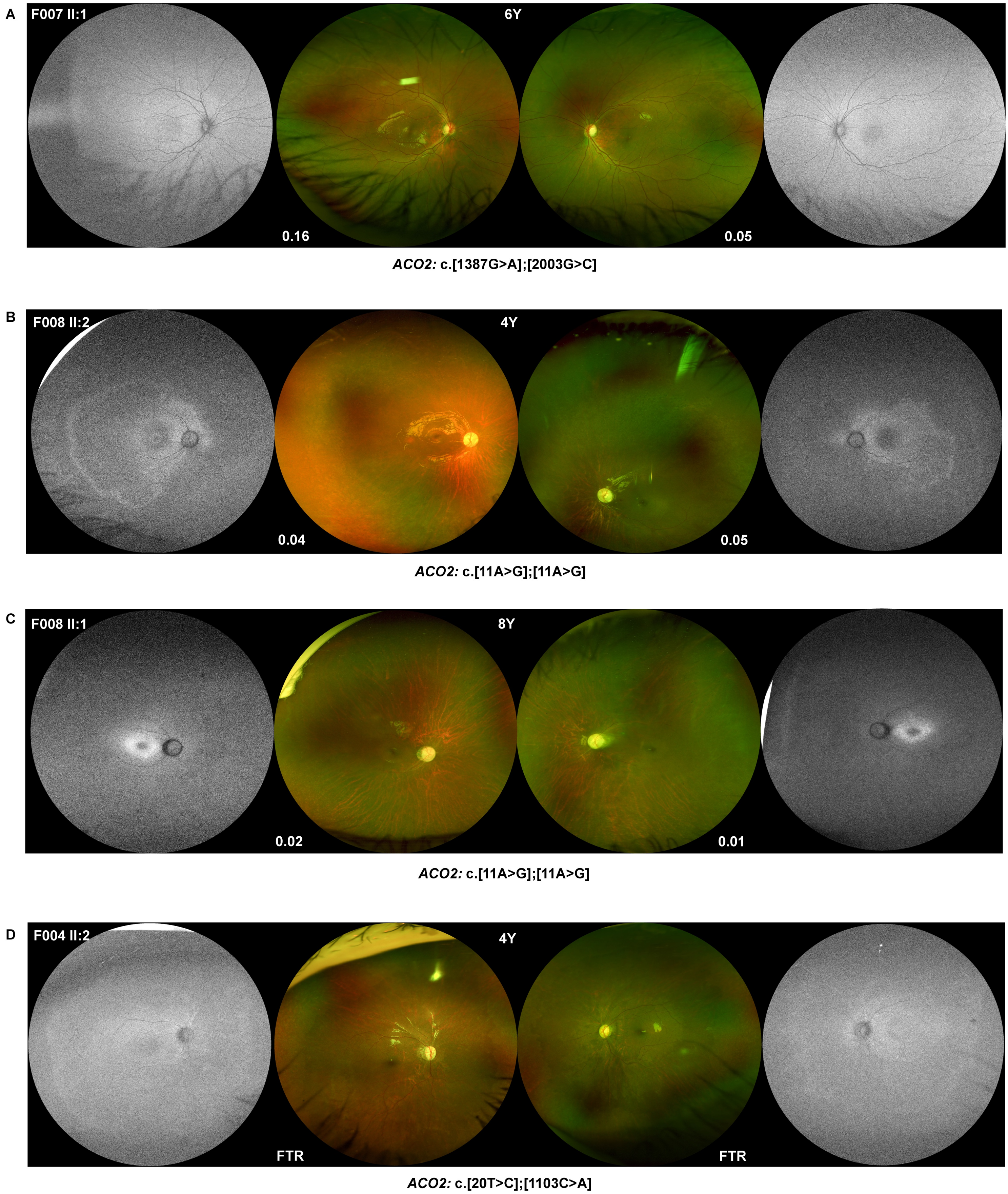


**Supplementary Figure 8. Multimodal images in patients with pathogenic variants in ACO2.**

Figure S9

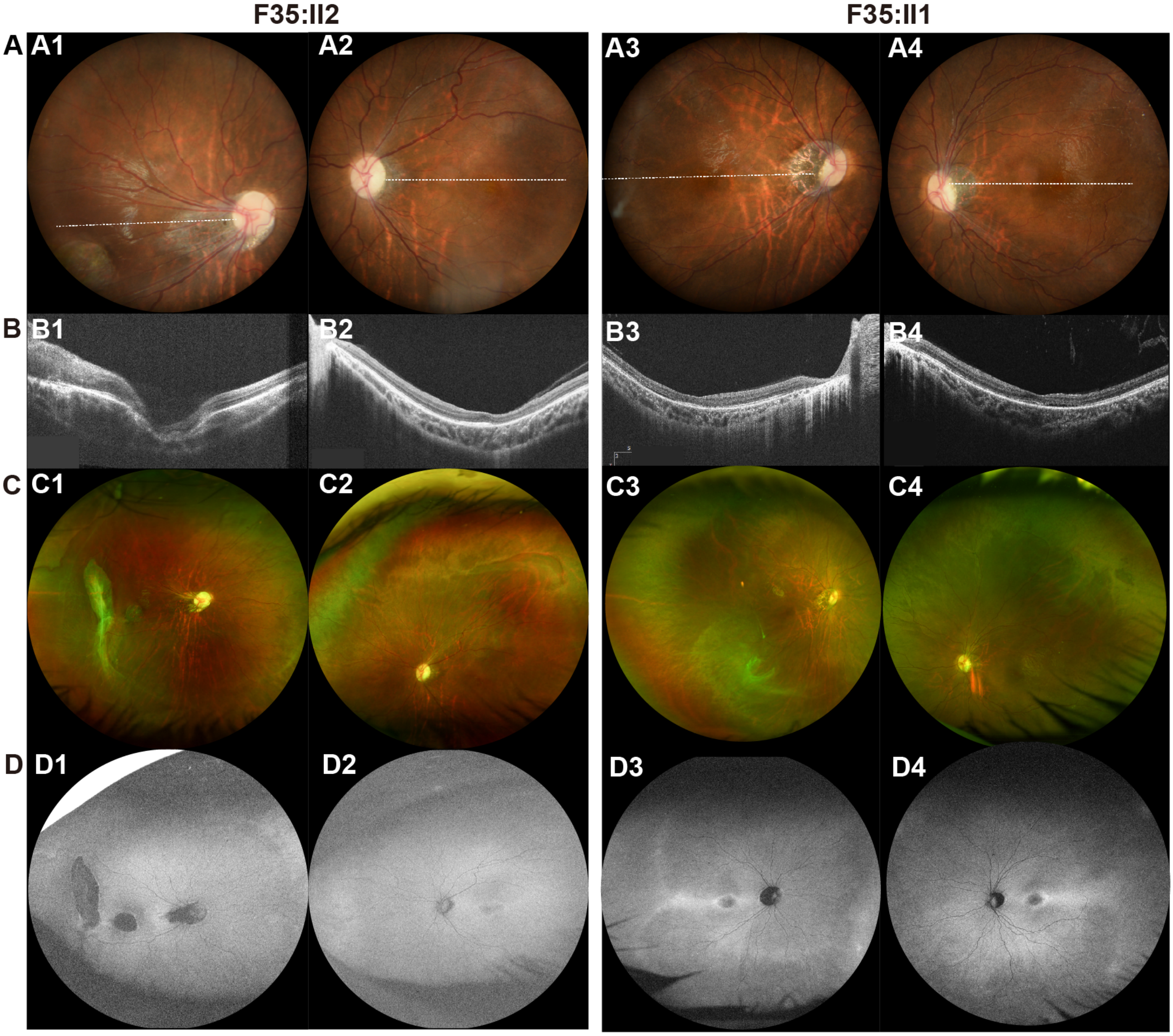


**Supplementary Figure 9. Multimodal images in patients with pathogenic variants in *NBAS.*** Both patients have tessellated fundus, and patient F035:II-2 oculus dexter showed temporal dragging of the optic disc, accompanied by geographic atrophy. FAF showed hypo autofluorescence in the area of geographic atrophy. The colour fundus of patient F035:II-1 shows a tessellated fundus and titled optic disc with peripapillary atrophy in both eyes.

Figure S10

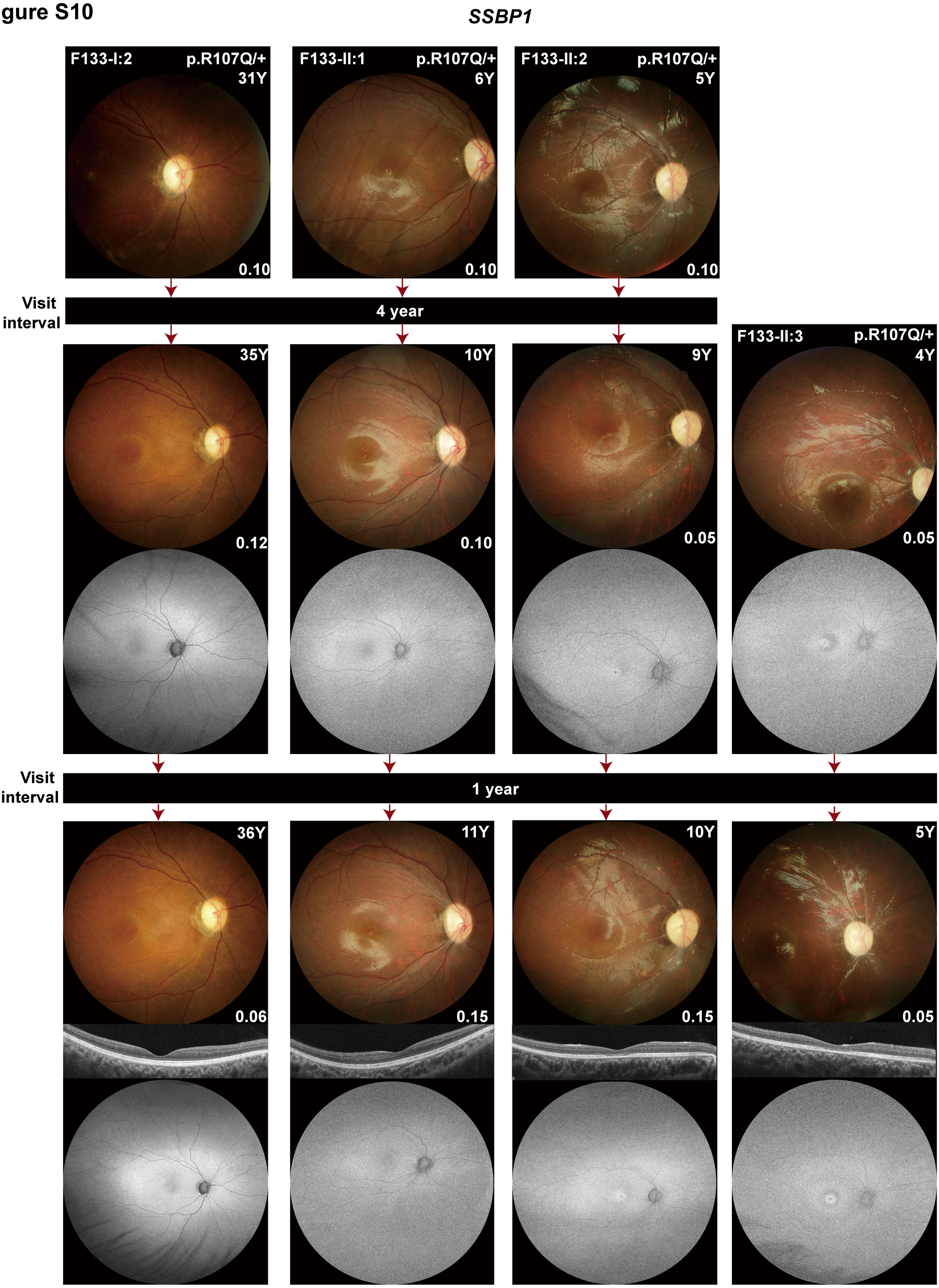


**Supplementary Figure 10. The follow-up of four patients with *SSBP1* variants.** A five-year follow-up of three patients with *SSBP1* variants showed common features including diffuse pale optic discs and attenuated vessels. FAF of these patients showed various changes. The FAF of F133-II:2 and F133-II:3 showed an increased autofluorescence in the macular region, which was absent in F133-II:1 and F133-I:2. The top right and bottom right corners of the fundus photographs show the exam age and visual acuity at the time of the respective examinations.

**Figure S11**

A. Ocular phenotypes in *POLG*

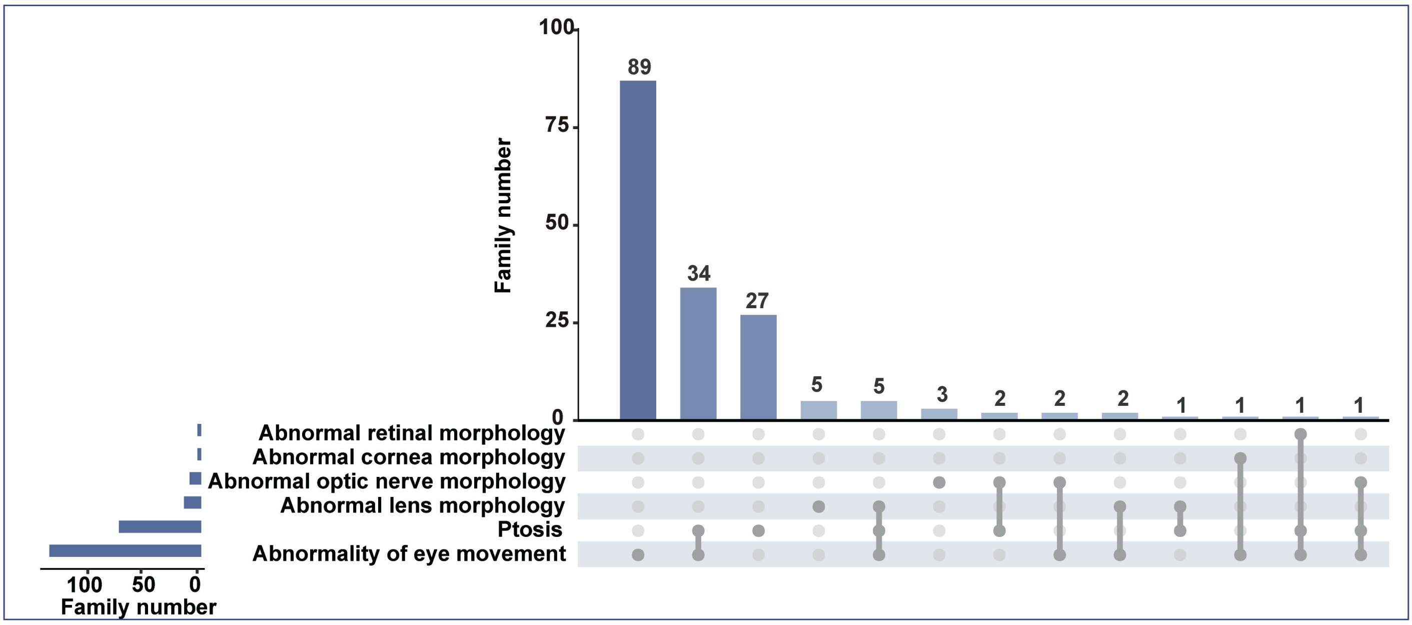


B. Ocular phenotypes in *SPG7*

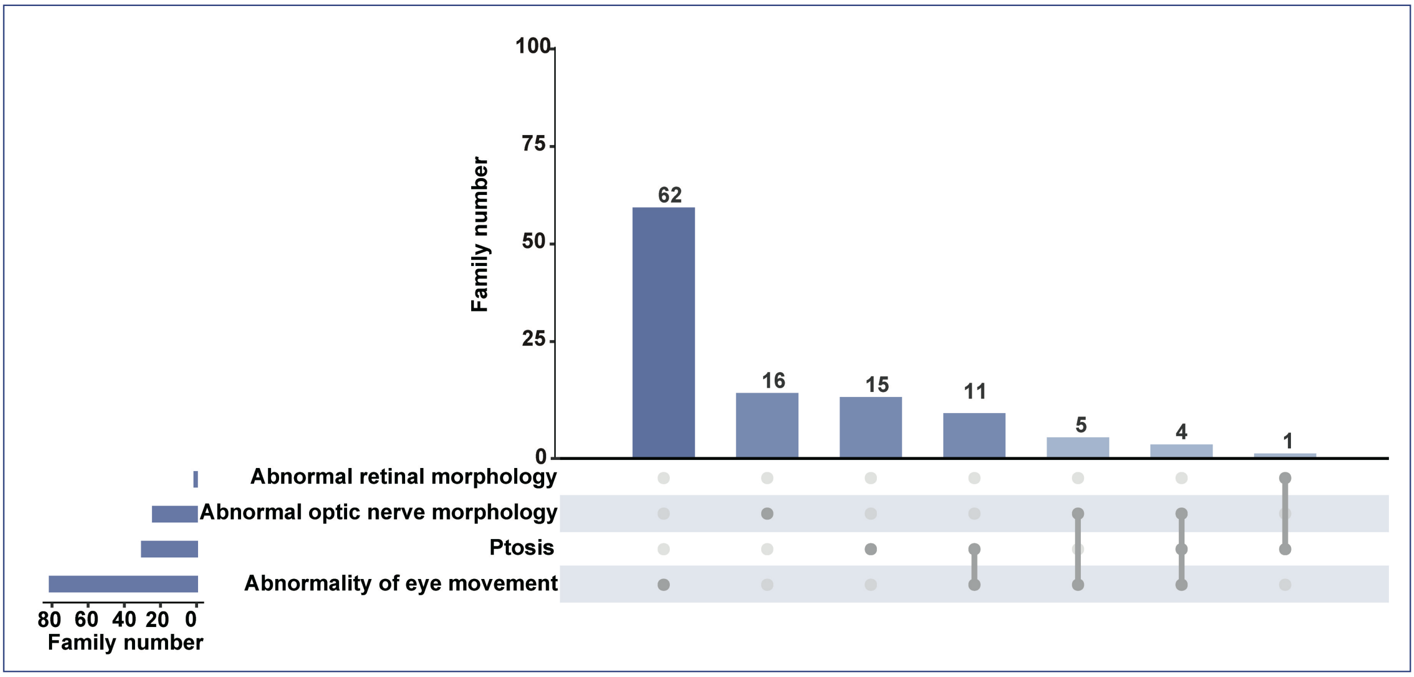


**Supplementary Figure 11**. A. Distribution of different ocular phenotypes in *POLG*. B. Distribution of different ocular phenotypes in *SPG7*. Vertical bars: Overlap between phenotypes in the datasets below, which are further specified by solid black circles; gray lines connecting the black circles indicate overlaps between multiple different phenotypes. The total number of each phenotype is indicated in the left of the matrix. Source data are provided as a Source Data file.

Figure S12

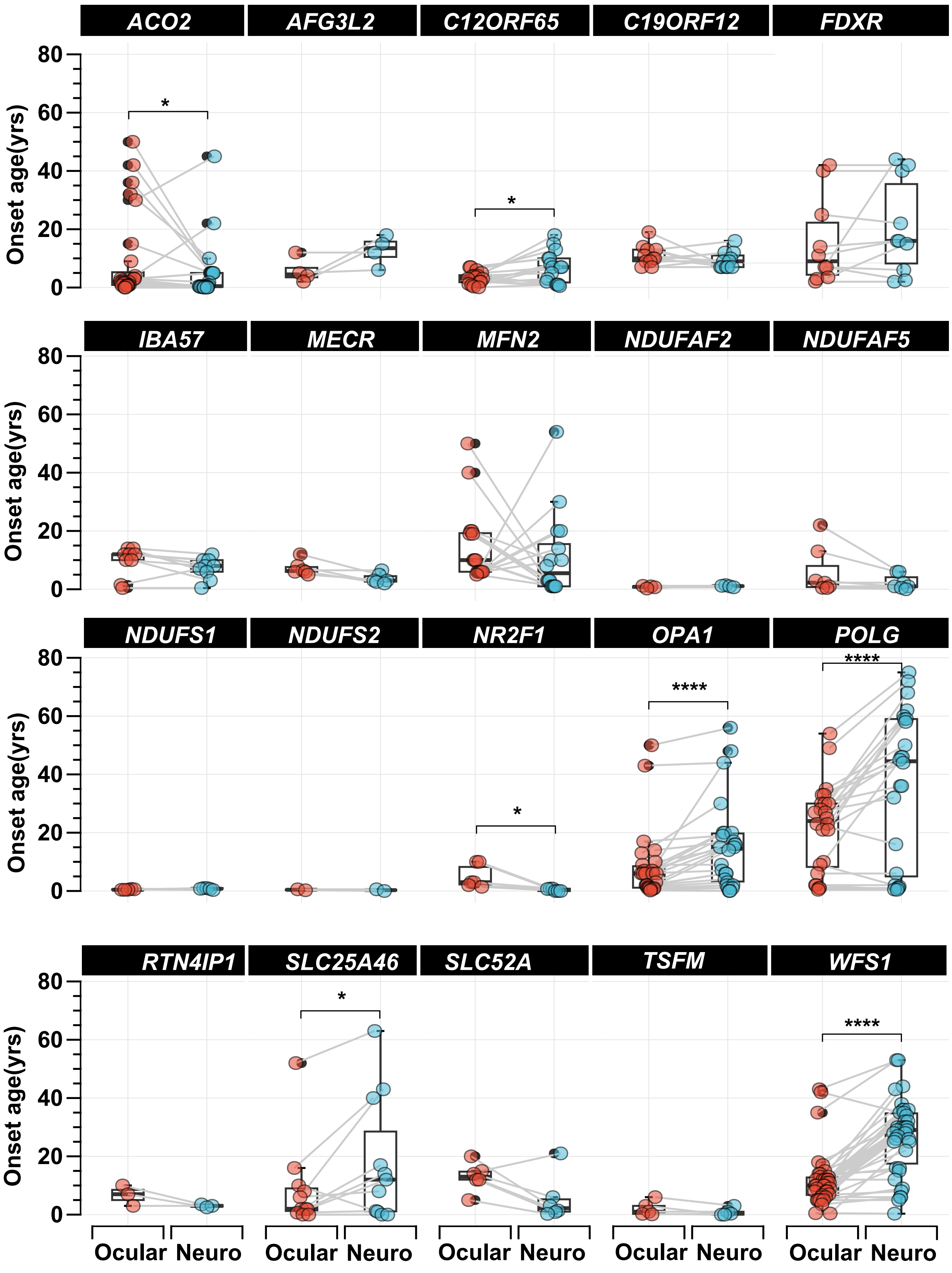


**Supplementary Figure 12. The comparison of onset age between ocular symptoms and neurological symtoms in individual gene.**

# Figure S13

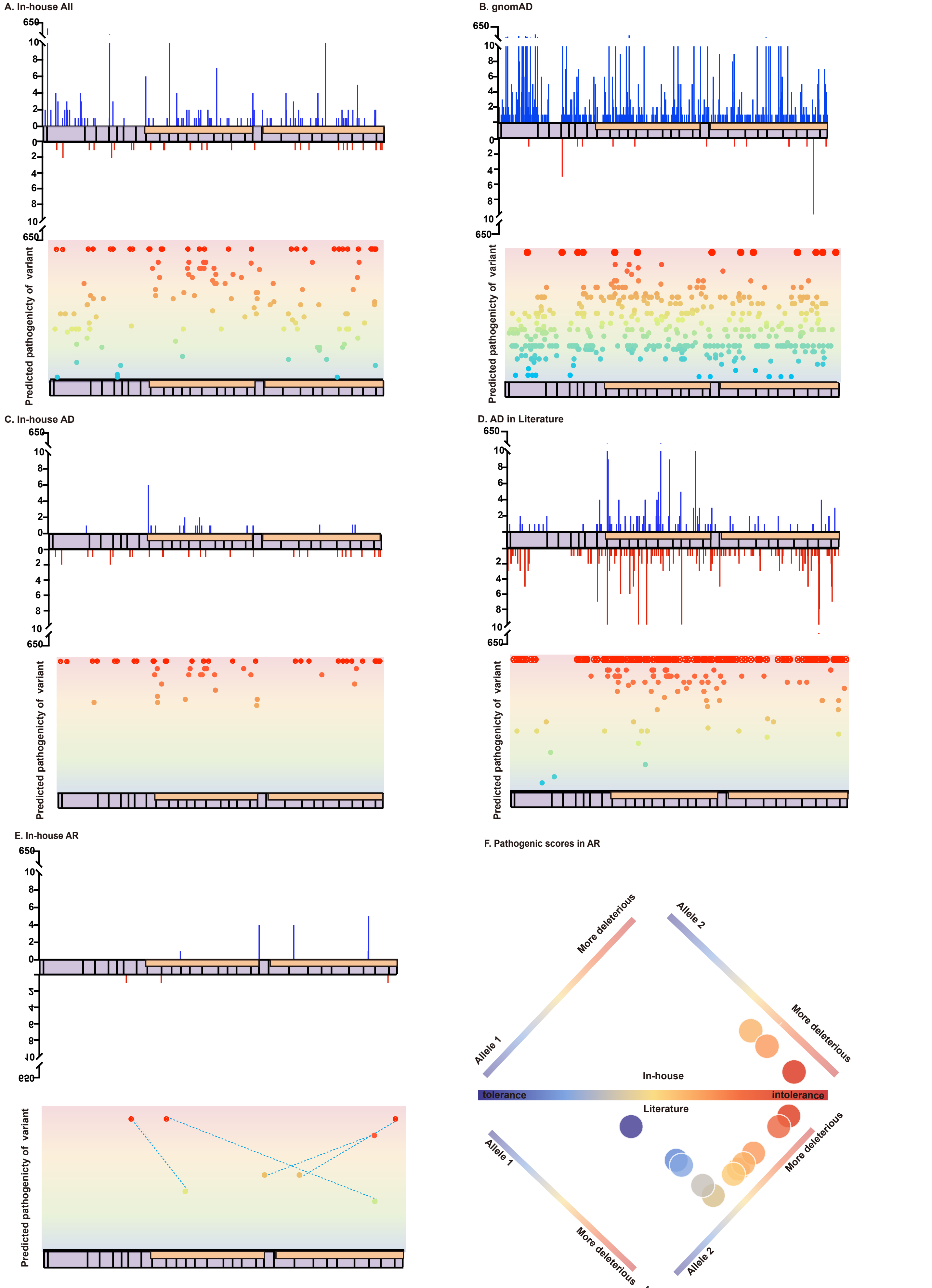


**Supplementary Figure 13. The contribution of the *OPA1* gene in the in-house data sets, gnomAD and published literature.** The blue and red lines in A), B), C) and D) represent missense and truncation in *OPA1*, respectively. The heatmap in F) was based on the ordering of the deleteriousness of each allele in *OPA1* from low to high. The color of each circle, from cool to warm, indicates the predicted severity of phenotype based on the predicted pathogenicity of biallelic alleles from mild to severe. The size of each circle indicated the number of pedigrees harboring the variants.

## Figure S14

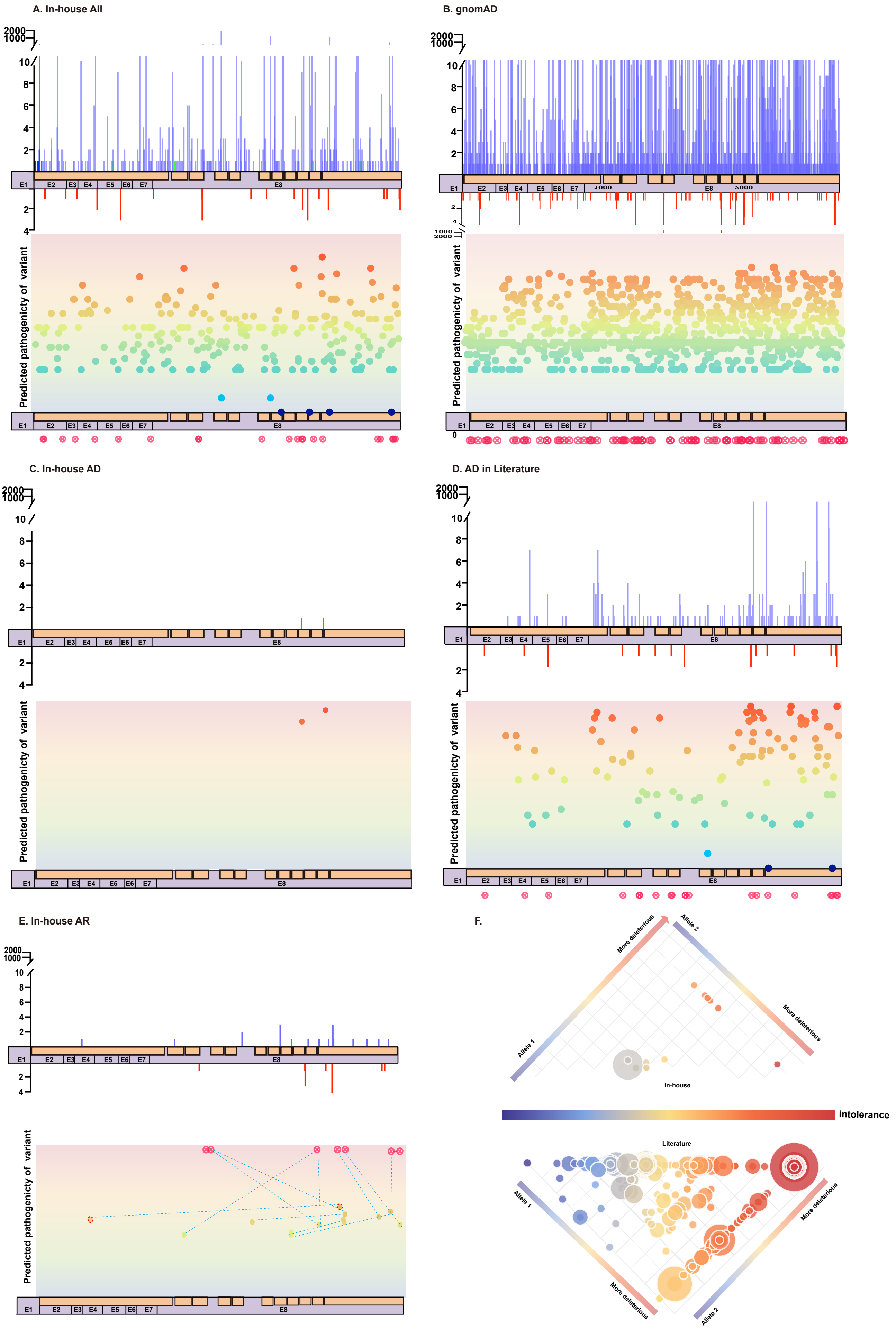


**Supplementary Figure 14. The contribution of the *WFS1* gene in the in-house data sets, gnomAD and published literature.** The blue and red lines in A), B), C) and D) represent missense and truncation, respectively. The heatmap in F) was based on the ordering of the deleteriousness of each allele in *WFS1* from low to high. The color of each circle, from cool to warm, indicates the predicted severity of phenotype based on the predicted pathogenicity of biallelic alleles from mild to severe. The size of each circle indicated the number of pedigrees harboring the variants.

**Figure 15**

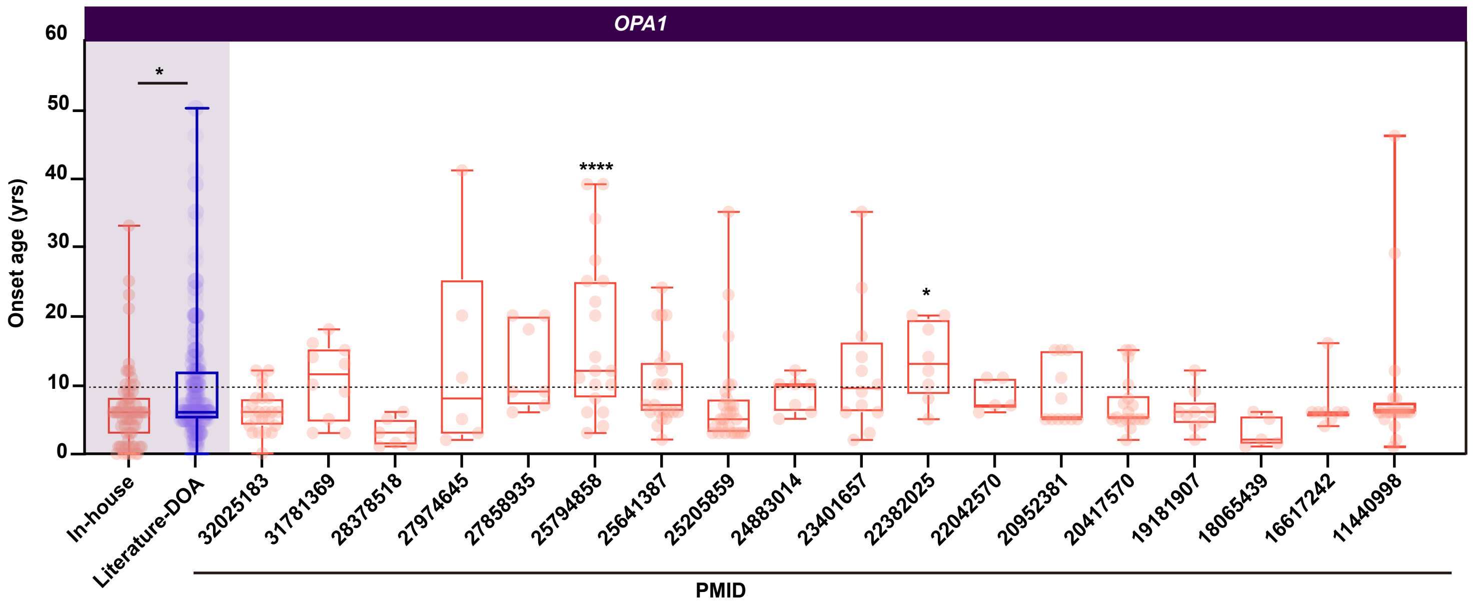


**Supplementary Figure 15. The age of onset of visual impairment in *OPA1* patients in our in-house data and literature reports.** The literature scan included all reports including the onset age of vision loss in patients with *OPA1*-DOA. Single published cohorts of *OPA1*-DOA were included if the onset ages were available from at least three patients. Differences in characteristics between groups were calculated using the Kruskal–Wallis test with Dunn post hoc tests (R package FSA).
