# Supplementary material for: Clinical and genetic landscape of optic atrophy in 826 families: insights from 50 nuclear genes
Source: Brain. 2024 Oct 18;148(5):1604–20. doi: 10.1093/brain/awae324 (PMC12073998; doi:10.1093/brain/awae324)
Supplement: awae324_Supplementary_Data [file awae324_supplementary_data.zip › brain-2024-01629-File014.pdf]

Table S4. Literature review of 50 HON-related nuclear genes.

Patient ID	Gene name	PMID	Family ID by Gene	Patient ID by Gene	Family ID in literatures	Patients ID in literatures	Ethicity	Ethicity Group	Transcipt Number	Variants 1	Effect 1	Variants 2	Effect 2	Hereditary mode	Genotype	State	Sex	Clinical diagnosis	Age of ocular sympotom onset	Age of neurologic onset
**AP0001**	***ACO2***	22405087	F001	P001	Family A	IV-3	Arabian	Non-EA	NM_001098.2	c.336C>G	p.Ser112Arg	c.336C>G	p.Ser112Arg	AR	M+M	homo	Male	Infantile Cerebellar-Retinal Degen	2.00	0.42
**AP0002**	***ACO2***	22405087	F001	P002	Family A	IV-5	Arabian	Non-EA	NM_001098.2	c.336C>G	p.Ser112Arg	c.336C>G	p.Ser112Arg	AR	M+M	homo	Female	Infantile Cerebellar-Retinal Degen	3.00	0.50
**AP0003**	***ACO2***	22405087	F001	P003	Family A	V-2	Arabian	Non-EA	NM_001098.2	c.336C>G	p.Ser112Arg	c.336C>G	p.Ser112Arg	AR	M+M	homo	Female	Infantile Cerebellar-Retinal Degen	1.50	0.17
**AP0004**	***ACO2***	22405087	F001	P004	Family A	V-5	Arabian	Non-EA	NM_001098.2	c.336C>G	p.Ser112Arg	c.336C>G	p.Ser112Arg	AR	M+M	homo	Female	Infantile Cerebellar-Retinal Degen	0.75	0.33
**AP0005**	***ACO2***	22405087	F001	P005	Family A	V-7	Arabian	Non-EA	NM_001098.2	c.336C>G	p.Ser112Arg	c.336C>G	p.Ser112Arg	AR	M+M	homo	Male	Infantile Cerebellar-Retinal Degen	2.50	0.25
**AP0006**	***ACO2***	22405087	F002	P006	Family B	III-1	Arabian	Non-EA	NM_001098.2	c.336C>G	p.Ser112Arg	c.336C>G	p.Ser112Arg	AR	M+M	homo	Female	Infantile Cerebellar-Retinal Degen	2.00	0.25
**AP0007**	***ACO2***	22405087	F002	P007	Family B	III-2	Arabian	Non-EA	NM_001098.2	c.336C>G	p.Ser112Arg	c.336C>G	p.Ser112Arg	AR	M+M	homo	Female	Infantile Cerebellar-Retinal Degen	1.50	0.42
**AP0008**	***ACO2***	22405087	F002	P008	Family B	III-3	Arabian	Non-EA	NM_001098.2	c.336C>G	p.Ser112Arg	c.336C>G	p.Ser112Arg	AR	M+M	homo	Female	Infantile Cerebellar-Retinal Degen	1.50	0.42
**AP0009**	***ACO2***	25351951	F003	P009	/	patient 2	French	Non-EA	NM_001098.2	c.220C>G	p.Leu74Val	c.1981G>A	p.Gly661Arg	AR	M+M	compound I	Male	Isolated optic neuropathy	3.00	/
**AP0010**	***ACO2***	25351951	F003	P010	/	patient 1	French	Non-EA	NM_001098.2	c.220C>G	p.Leu74Val	c.1981G>A	p.Gly661Arg	AR	M+M	compound I	Male	Isolated optic neuropathy	5.00	/
**AP0011**	***ACO2***	25351951	F004	P011	/	patient 3	Algerian	Non-EA	NM_001098.2	c.776G>A	p.Gly259Asp	c.776G>A	p.Gly259Asp.	AR	M+M	homo	Male	Syndromic optic neuropathy	0.00	0.00
**AP0012**	***ACO2***	25351951	F005	P012	/	patient 4	Algerian	Non-EA	NM_001098.2	c.776G>A	p.Gly259Asp	c.776G>A	p.Gly259Asp.	AR	M+M	homo	Male	Syndromic optic neuropathy	0.00	0.00
**AP0013**	***ACO2***	25351951	F006	P013	/	patient 5	Algerian	Non-EA	NM_001098.2	c.2208G>C	p.Lys736Asn	c.2325_2328d	p.Lys776Asnfs	AR	M+T	compound I	Female	Syndromic optic neuropathy	/	0.42
**AP0014**	***ACO2***	26992325	F007	P014	/	/	Afro-Caribbean an	Non-EA	NM_001098.2	c.2135C >T	p.Pro712Leu	c.2135C>T	p.Pro712Leu	AR	M+M	homo	Male	Mitochondrial aconitase deficiency	Not mention	0.50
**AP0015**	***ACO2***	28463998	F008	P015	/	F4-P1	Caucasian	Non-EA	NM_001098.2	c.1859G>A	p.Gly620Asp/	c.2048G>T	p.Gly683Val	AR	M+M	compound I	Female	Syndromic optic neuropathy	Not mention	Not mention
**AP0016**	***ACO2***	28463998	F009	P016	/	F3-P1	Arabian	Non-EA	NM_001098.2	c.336C>G	p.Ser112Arg	c.336C>G	p.Ser112Arg	AR	M+M	homo	Female	Syndromic optic neuropathy	Not mention	Not mention
**AP0017**	***ACO2***	28463998	F009	P017	/	F3-P2	Arabian	Non-EA	NM_001098.2	c.336C>G	p.Ser112Arg	c.336C>G	p.Ser112Arg	AR	M+M	homo	Female	Syndromic optic neuropathy	Not mention	Not mention
**AP0018**	***ACO2***	28545339	F010	P018	/	/	Mixed European	Non-EA	NM_001098.2	c.1091T>C	p.Val364Ala	c.2328_2331d	p.Lys776Asnfs	AR	M+T	compound I	Male	development delay	Not mention	1.25
**AP0019**	***ACO2***	29564393	F011	P019	/	/	Caucasian	Non-EA	NM_001098.2	c.2135C>T	p.Pro712Leu	c.940+5G>C	p.?	AR	M+T	compound I	Female	optic atrophy with spastic parapleg	Not mention	3.00
**AP0020**	***ACO2***	29577077	F012	P020	/	IV-1	Arab- Bedouin	Non-EA	NM_001098.2	c.1240T>G	p.Phe414Val	c.1240T>G	p.Phe414Val	AR	M+M	homo	Male	spastic paraplegia	/	0.25
**AP0021**	***ACO2***	29577077	F012	P021	/	IV-3	Arab	Non-EA	NM_001098.2	c.1240T>G	p.Phe414Val	c.1240T>G	p.Phe414Val	AR	M+M	homo	Female	spastic paraplegia	/	1.00
**AP0022**	***ACO2***	30118607	F013	P022	/	Sibling 1	Australian	Non-EA	NM_001098.2	c.220C>G	p.Leu74Val	c.2208+1dup	p.?	AR	M+T	compound I	Male	Isolated optic neuropathy	2.00	Never
**AP0023**	***ACO2***	30118607	F014	P023	/	Sibling 2	Australian	Non-EA	NM_001098.2	c.220C>G	p.Leu74Val	c.2208+1dup	p.?	AR	M+T	compound I	Male	Isolated optic neuropathy	8.00	Never
**AP0024**	***ACO2***	30552426	F015	P024	/	LOVD176985	Caucasian	Non-EA	NM_001098.2	c.1859G>A	p.Gly620Asp	c.2048G>T	p.Gly683Val	AR	M+M	compound I	Female	early-onset epileptic encephalopat	Not mention	0.58
**AP0025**	***ACO2***	30689204	F016	P025	/	IS-9	Arabian	Non-EA	NM_001098.2	c.336C>G	p.Ser112Arg	c.336C>G	p.Ser112Arg	AR	M+M	homo	Female	Syndromic optic neuropathy	0.75	0.25
**AP0026**	***ACO2***	30689204	F016	P026	/	IS-10	Arabian	Non-EA	NM_001098.2	c.336C>G	p.Ser112Arg	c.336C>G	p.Ser112Arg	AR	M+M	homo	Female	neurometabolic syndrome	0.58	0.33
**AP0027**	***ACO2***	30689204	F017	P027	/	A-1	Hipanic/Caucasian	Non-EA	NM_001098.2	c.260C>T	p.Ser87Leu	c.685-1_685d	p.?	AR	M+T	compound I	Female	neurometabolic syndrome	0.67	0.58
**AP0028**	***ACO2***	30689204	F018	P028	/	A-2	Caucasian	Non-EA	NM_001098.2	c.1181G>A	p.Gly394Glu	c.1722G>A	p.Trp574*	AR	M+T	compound I	Male	neurometabolic syndrome	0.83	0.00
**AP0029**	***ACO2***	30689204	F019	P029	/	A-3	Caucasian	Non-EA	NM_001098.2	c.172C>T	p.Arg58*	c.590A>G	p.Asn197Ser	AR	M+T	compound I	Male	neurometabolic syndrome	2.00	0.06
**AP0030**	***ACO2***	30689204	F020	P030	/	E-2	Africa/Caucasian	Non-EA	NM_001098.2	c.1787A>G	p.His596Arg	c.2050C>T	p.Arg684Trp.	AR	M+M	compound I	Female	neurometabolic syndrome	/	0.17
**AP0031**	***ACO2***	30689204	F021	P031	/	E-3	Africa/Caucasian	Non-EA	NM_001098.2	c.1787A>G	p.His596Arg	c.2050C>T	p.Arg684Trp.	AR	M+M	compound I	Male	neurometabolic syndrome	/	3.00
**AP0032**	***ACO2***	31106992	F022	P032	/	/	Japanese	EA	NM_001098.2	c.1534G > A	p.Asp512Asn	c.1997G>C	p.Gly666Ala	AR	M+M	compound I	Female	Cerebellar atrophy	0.90	0.90
**AP0033**	***ACO2***	31231135	F023	P034	/	/	Caucasian	Non-EA	NM_001098.2	c.85C>T	p.Arg29Trp.	c.283C>T	p.Arg95Cys	AR	M+M	compound I	/	None	Not mention	Not mention
[illegible]	[illegible]	[illegible]	[illegible]	[illegible]	[illegible]	[illegible]	[illegible]	[illegible]	[illegible]	[illegible]	[illegible]	[illegible]	[illegible]	[illegible]	[illegible]	[illegible]	[illegible]	[illegible]	[illegible]	[illegible]

Patient ID	Abnormality of the nervous system HP:0000707	Abnormal optic nerve morphology HP:0000587	Abnormal retinal morphology HP:0000479	Ptosis HP:0000508	Abnormality of eye movement HP:0000496	Abnormal cornea morphology HP:0000481	Abnormal lens morphology HP:0000517	Color vision defect HP:0000551	Abnormality of the ear	Abnormality of the musculature	Abnormality of the endocrine system	Abnormality of the cardiovascular system	Abnormal respiratory system physiology	Abnormality of the genital system	Abnormality of the urinary system	Abnormality of the digestive system	Abnormality of the bone	Age at present visit	Alive	Age at BCVA test	(OD.BCVA at last visit(snellen.	(OS.BCVA at last visit(snellen.	Fundus at present	(OD.OCT-thickness-TEMPORAL	(OS.OCT-thickness	MRI
**AP0001**	Ataxia HP:00012	Optic atrophy HP	/	/	Strabismus HP:0	/	/	/	/	Hypotonia HP:00	/	/	/	/	/	/	/	18.0	YES	/	/	/	/	/	/	/
**AP0002**	Ataxia HP:00012	Optic atrophy HP	Retinal dystrophy	/	Strabismus HP:0	/	/	/	/	Hypotonia HP:00	/	/	/	/	/	/	/	13.0	YES	/	/	/	/	/	/	/
**AP0003**	Ataxia HP:00012	Optic atrophy HP	Retinal dystrophy	/	Nystagmus HP:0	/	/	/	Sensorineural he	Hypotonia HP:00	/	/	/	/	/	/	/	7.0	YES	/	/	/	/	/	/	/
**AP0004**	Ataxia HP:00012	Optic atrophy HP	Retinal dystrophy	/	Nystagmus HP:0	/	/	/	Sensorineural he	Hypotonia HP:00	/	/	/	/	/	/	/	3.0	YES	/	/	/	/	/	/	/
**AP0005**	Ataxia HP:00012	Optic atrophy HP	/	/	Strabismus HP:0	/	/	/	/	Hypotonia HP:00	/	/	/	/	/	/	/	0.5	YES	/	/	/	/	/	/	/
**AP0006**	Ataxia HP:00012	Optic atrophy HP	Retinal dystrophy	/	Nystagmus HP:0	/	/	/	/	Hypotonia HP:00	/	/	/	/	/	/	/	9.0	YES	/	/	/	/	/	/	/
**AP0007**	Ataxia HP:00012	Optic atrophy HP	Retinal dystrophy	/	Nystagmus HP:0	/	/	/	/	/	/	/	/	/	/	/	/	8.0	YES	/	/	/	/	/	/	/
**AP0008**	Ataxia HP:00012	Optic atrophy HP	Retinal dystrophy	/	Nystagmus HP:0	/	/	/	/	/	/	/	/	/	/	/	/	2.0	YES	/	/	/	/	/	/	/
**AP0009**	/	Optic atrophy HP	/	/	/	/	/	/	/	/	/	/	/	/	/	/	/	36.0	YES	20	0.1	0.1	TP	/	/	/
**AP0010**	/	Optic atrophy HP	/	/	/	/	/	/	/	/	/	/	/	/	/	/	/	41.0	YES	20	0.1	0.1	TP	/	/	/
**AP0011**	Abnormality of th	Optic atrophy HP	/	/	/	/	/	/	/	Hypotonia HP:00	/	/	/	/	/	/	/	0.2	NO	/	/	/	DPOD	/	/	/
**AP0012**	Abnormality of th	Optic atrophy HP	Retinal dystrophy	/	Abnormality of ey	/	/	/	/	Hypotonia HP:00	/	Bradycardia HP:0	Apnea HP:00021	/	/	/	/	0.2	NO	/	/	/	DPOD	/	/	/
**AP0013**	Cerebellar atropl	Optic atrophy HP	Retinal dystrophy	/	Nystagmus HP:0	/	/	/	/	Hypotonia HP:00	/	/	/	/	/	/	/	4.0	YES	/	/	/	DPOD	/	/	/
**AP0014**	Ataxia HP:00012	/	/	Ptosis HP:00050	Abnormal saccad	/	/	/	Sensorineural he	Hypotonia HP:00	/	/	/	/	/	/	/	3.0	YES	/	/	/	/	/	/	/
**AP0015**	Ataxia HP:00012	Optic atrophy HP	/	/	Strabismus HP:0	/	/	/	/	Hypotonia HP:00	/	/	/	/	/	/	/	3.8	NO	/	/	/	/	/	/	/
**AP0016**	Ataxia HP:00012	Optic atrophy HP	/	/	Strabismus HP:0	/	/	/	Sensorineural he	/	/	/	/	/	/	/	/	17.0	YES	/	/	/	/	/	/	/
**AP0017**	Neurodevelopme	Optic atrophy HP	/	/	Strabismus HP:0	/	/	/	/	/	/	/	/	/	/	/	/	14.0	YES	/	/	/	/	/	/	/
**AP0018**	Neurodevelopme	Optic atrophy HP	/	/	Abnormality of ey	/	/	/	/	/	/	/	/	/	/	/	/	18.0	YES	/	/	/	/	/	/	/
**AP0019**	Neurodevelopme	Optic atrophy HP	/	/	/	/	/	/	/	/	/	/	/	/	/	/	/	56.0	YES	/	/	/	/	/	/	/
**AP0020**	Neurodevelopme	/	/	/	/	/	/	/	/	/	/	/	/	/	/	/	/	18.0	YES	/	/	/	/	/	/	/
**AP0021**	Neurodevelopme	/	/	/	/	/	/	/	/	/	/	/	/	/	/	/	/	28.0	YES	/	/	/	/	/	/	/
**AP0022**	/	Optic atrophy HP	/	/	Abnormality of ey	/	/	/	/	/	/	/	/	/	/	/	/	5.0	YES	5	0.05	0.05	/	/	/	/
**AP0023**	/	Optic atrophy HP	/	/	Abnormality of ey	/	/	/	/	/	/	/	/	/	/	/	/	9.0	YES	9	0.25	0.16	/	/	/	/
**AP0024**	Spasticity HP:00	/	/	/	/	/	/	/	/	Hypotonia HP:00	/	/	/	/	/	/	/	1.8	YES	/	/	/	/	/	/	/
**AP0025**	Neurodevelopme	Optic atrophy HP	/	/	Strabismus HP:0	/	/	/	/	Hypotonia HP:00	/	/	/	/	/	/	/	19.0	YES	/	/	/	DPOD	/	/	/
**AP0026**	Neurodevelopme	Optic atrophy HP	/	/	Strabismus HP:0	/	/	/	/	Hypotonia HP:00	/	/	/	/	/	/	/	16.0	YES	/	/	/	DPOD	/	/	/
**AP0027**	Neurodevelopme	Optic atrophy HP	/	/	Strabismus HP:0	/	/	/	/	Hypotonia HP:00	/	/	/	/	/	/	/	8.0	YES	/	/	/	DPOD	/	/	/
**AP0028**	Neurodevelopme	Optic atrophy HP	/	/	Strabismus HP:0	/	/	/	/	Hypotonia HP:00	/	/	/	/	/	/	/	14.0	NO	/	/	/	/	/	/	/
**AP0029**	Neurodevelopme	Optic atrophy HP	/	/	Strabismus HP:0	/	/	/	/	Hypotonia HP:00	/	/	/	/	/	/	/	5.0	YES	/	/	/	/	/	/	/
**AP0030**	Neurodevelopme	Optic atrophy HP	/	/	Strabismus HP:0	/	/	/	/	Hypotonia HP:00	/	/	/	/	/	/	/	8.0	YES	/	/	/	/	/	/	/
**AP0031**	Neurodevelopme	Optic atrophy HP	/	/	Strabismus HP:0	/	/	/	/	Hypotonia HP:00	/	/	/	/	/	/	/	5.0	YES	/	/	/	/	/	/	/
**AP0032**	Cerebellar atropl	Optic atrophy HP	/	/	Nystagmus HP:0	/	/	/	/	/	/	/	/	/	/	/	/	5.0	NO	/	/	/	/	/	/	/
**AP0033**	/	/	/	/	/	/	/	/	/	/	/	/	/	/	/	/	/	[illegible]	[illegible]	[illegible]	[illegible]	[illegible]	[illegible]	[illegible]	[illegible]	[illegible]
[illegible]	[illegible]	[illegible]	[illegible]	[illegible]	[illegible]	[illegible]	[illegible]	[illegible]	[illegible]	[illegible]	[illegible]	[illegible]	[illegible]	[illegible]	[illegible]	[illegible]	[illegible]	[illegible]	[illegible]	[illegible]	[illegible]	[illegible]	[illegible]	[illegible]	[illegible]	[illegible]

**AP0481**	***BTD***	29359854	F172	P176	/	P7	Chinese	EA	NM_000060.4	c.395T>G	p.Met132Trp	c.637delC	p.His213Thrfs*	AR	M+T	compound I	Male	Biotinidase deficiency
**AP0482**	***BTD***	29359854	F173	P177	/	P8	Chinese	EA	NM_000060.4	c.235C>T	p.Arg79Cys	c.235C>T	p.Arg79Cys	AR	M+M	homo	Female	Biotinidase deficiency
**AP0483**	***BTD***	29353266	F174	P178	/	1	Turkish	Non-EA	NM_000060.4	c.956C>T	p.Ser319Phe	c.1330G>C	p.Asp444His	AR	M+M	compound I	/	Biotinidase deficiency
**AP0484**	***BTD***	29353266	F175	P179	/	2	Turkish	Non-EA	NM_000060.4	c.1330G>C	p.Asp444His	c.592_594del(	p.Leu498Phef	AR	M+T	compound I	/	Biotinidase deficiency
**AP0485**	***BTD***	29353266	F176	P180	/	3	Turkish	Non-EA	NM_000060.4	c.1330G>C	p.Asp444His	c.1324del	p.Val442Serfs*	AR	M+T	compound I	/	Biotinidase deficiency
**AP0486**	***BTD***	29353266	F177	P181	/	4	Turkish	Non-EA	NM_000060.4	c.1330G>C	p.Asp444His	c.1324del	p.Val442Serfs*	AR	M+T	compound I	/	Biotinidase deficiency
**AP0487**	***BTD***	29353266	F178	P182	/	5	Turkish	Non-EA	NM_000060.4	c.1330G>C	p.Asp444His	c.1324del	p.Val442Serfs*	AR	M+T	compound I	/	Biotinidase deficiency
**AP0488**	***BTD***	29353266	F179	P183	/	6	Turkish	Non-EA	NM_000060.4	c.1330G>C	p.Asp444His	c.1324del	p.Val442Serfs*	AR	M+T	compound I	/	Biotinidase deficiency
**AP0489**	***BTD***	29353266	F180	P184	/	7	Turkish	Non-EA	NM_000060.4	c.1330G>C	p.Asp444His	c.1324del	p.Val442Serfs*	AR	M+T	compound I	/	Biotinidase deficiency
**AP0490**	***BTD***	29353266	F181	P185	/	8	Turkish	Non-EA	NM_000060.4	c.1330G>C	p.Asp444His	c.1324del	p.Val442Serfs*	AR	M+T	compound I	/	Biotinidase deficiency
**AP0491**	***BTD***	29353266	F182	P186	/	9	Turkish	Non-EA	NM_000060.4	c.419G>A	p.Trp140*	c.419G>A	p.Trp140*	AR	T+T	homo	/	Biotinidase deficiency
**AP0492**	***BTD***	29353266	F183	P187	/	10	Turkish	Non-EA	NM_000060.4	c.419G>A	p.Trp140*	c.470G>A	p.Arg157His	AR	M+T	compound I	/	Biotinidase deficiency
**AP0493**	***BTD***	29353266	F184	P188	/	11	Turkish	Non-EA	NM_000060.4	c.1330G>C	p.Asp444His	c.192_193ins(	p.L69Hfs*24	AR	M+T	compound I	/	Biotinidase deficiency
**AP0494**	***BTD***	29353266	F185	P189	/	12	Turkish	Non-EA	NM_000060.4	c.1330G>C	p.Asp444His	c.192_193ins(	p.L69Hfs*24	AR	M+T	compound I	/	Biotinidase deficiency
**AP0495**	***BTD***	29353266	F186	P190	/	13	Turkish	Non-EA	NM_000060.4	c.1330G>C	p.Asp444His	c.192_193ins(	p.L69Hfs*24	AR	M+T	compound I	/	Biotinidase deficiency
**AP0496**	***BTD***	29353266	F187	P191	/	14	Turkish	Non-EA	NM_000060.4	c.1330G>C	p.Asp444His	c.1340A>G	p.His447Arg	AR	M+M	compound I	/	Biotinidase deficiency
**AP0497**	***BTD***	28971021	F188	P192	/	2	Caucasian	Non-EA	NM_000060.4	c.1330G>C	p.Asp444His	c.631C>T	p.Arg211Cys	AR	M+M	compound I	Female	Biotinidase deficiency
**AP0498**	***BTD***	28971021	F189	P193	/	3	Caucasian	Non-EA	NM_000060.4	c.1368A>C	p.Gln456His	c.557G>A	p.Cys186Tyr	AR	M+M	compound I	Female	Biotinidase deficiency
**AP0499**	***BTD***	28971021	F190	P194	/	4	Caucasian	Non-EA	NM_000060.4	c.1368A>C	p.Gln456His	c.654G>C	p.Glu218Asp	AR	M+M	compound I	Female	Biotinidase deficiency
**AP0500**	***BTD***	28971021	F191	P195	/	6	Caucasian	Non-EA	NM_000060.4	c.1330G>C	p.Asp444His	c.1330G>C	p.Asp444His	AR	M+M	homo	Male	Biotinidase deficiency
**AP0501**	***BTD***	28971021	F192	P196	/	7	Caucasian	Non-EA	NM_000060.4	c.1368A>C	p.Gln456His	c.654G>C	p.Glu218Asp	AR	M+M	compound I	Male	Biotinidase deficiency
**AP0502**	***BTD***	28971021	F193	P197	/	8	Caucasian	Non-EA	NM_000060.4	c.1368A>C	p.Gln456His	c.100G>A	p.Gly34Ser	AR	M+M	compound I	Female	Biotinidase deficiency
**AP0503**	***BTD***	28971021	F194	P198	/	9	Caucasian	Non-EA	NM_000060.4	c.1368A>C	p.Gln456His	c.100G>A	p.Gly34Ser	AR	M+M	compound I	Male	Biotinidase deficiency
**AP0504**	***BTD***	28971021	F195	P199	/	10	Caucasian	Non-EA	NM_000060.4	c.1330G>C	p.Asp444His	c.1330G>C	p.Asp444His	AR	M+M	homo	Male	Biotinidase deficiency
**AP0505**	***BTD***	28971021	F196	P200	/	11	Caucasian	Non-EA	NM_000060.4	c.734G>A	p.Cys245Tyr	c.1368A>C	p.Gln456His	AR	M+M	compound I	Male	Biotinidase deficiency
**AP0506**	***BTD***	28971021	F197	P201	/	12	Caucasian	Non-EA	NM_000060.4	c.1137G>A	p.Met379Ile	c.1368A>C	p.Gln456His	AR	M+M	compound I	Male	Biotinidase deficiency
**AP0507**	***BTD***	28971021	F198	P202	/	13	Caucasian	Non-EA	NM_000060.4	c.734G>A	p.Cys245Tyr	c.1368A>C	p.Gln456His	AR	M+M	compound I	Male	Biotinidase deficiency
**AP0508**	***BTD***	28649532	F199	P203	/	patient 1	Caucasian	Non-EA	NM_000060.4	c.98_104delinsTCC	p.Cys33Phefs*36	c.98_104delin	p.Cys33Phefs*	AR	T+T	homo		Biotinidase deficiency
**AP0509**	***BTD***	28649532	F200	P204	/	patient 2	Caucasian	Non-EA	NM_000060.4	c.1316C>A	p.Ala439Asp	c.1413T>C	p.=	AR	M+T	compound heter		Biotinidase deficiency
**AP0510**	***BTD***	28649532	F201	P205	/	patient 3	Caucasian	Non-EA	NM_000060.4	c.1413T>C	p.=	c.1413T>C	p.=	AR	T+T	homo		Biotinidase deficiency
**AP0511**	***BTD***	27657684	F202	P206	/	subject 1	Caucasian	Non-EA	NM_000060.4	c.511G>A	p.Ala171Thr	c.1330G>C	p.Asp444His	AR	M+M	compound I	Female	Biotinidase deficiency
**AP0512**	***BTD***	27657684	F203	P207	/	subject 3	Caucasian	Non-EA	NM_000060.4	c.511G>A	p.Ala171Thr	c.1330G>C	p.Asp444His	AR	M+M	compound I	Male	Biotinidase deficiency
**AP0513**	***BTD***	27657684	F204	P208	/	subject 4	Caucasian	Non-EA	NM_000060.4	c.755A>G	p.Asp252Gly	c.755A>G	p.Asp252Gly	AR	M+M	homo	Male	Biotinidase deficiency
**AP0514**	***BTD***	27657684	F205	P209	/	subject 5	Caucasian	Non-EA	NM_000060.4	c.511G>A	p.Ala171Thr	c.1330G>C	p.Asp444His	AR	M+M	compound I	Female	Biotinidase deficiency
**AP0515**	***BTD***	27657684	F206	P210	/	subject 6	Caucasian	Non-EA	NM_000060.4	c.755A>G	p.Asp252Gly	c.933delT	p.Ser311Argfs	AR	M+T	compound I	Female	Biotinidase deficiency
**AP0516**	***BTD***	27657684	F207	P211	/	subject 7	Caucasian	Non-EA	NM_000060.4	c.511G>A	p.Ala171Thr	c.1330G>C	p.Asp444His	AR	M+M	compound I	Female	Biotinidase deficiency
**AP0517**	***BTD***	27657684	F208	P212	/	subject 8	Caucasian	Non-EA	NM_000060.4	c.1612C>T	p.Arg538Cys	c.755A>G	p.Asp252Gly	AR	M+M	compound I	Male	Biotinidase deficiency
**AP0518**	***BTD***	27657684	F209	P213	/	subject 10	Caucasian	Non-EA	NM_000060.4	c.100G>A	p.Gly34Ser	c.485C>T	p.Ala162Val	AR	M+M	compound I	Female	Biotinidase deficiency
**AP0519**	***BTD***	27657684	F210	P214	/	subject 11	Caucasian	Non-EA	NM_000060.4	c.755A>G	p.Asp252Gly	c.1595C>T	p.Thr532Met	AR	M+M	compound I	Female	Biotinidase deficiency
**AP0520**	***BTD***	27657684	F211	P215	/	subject 13	Caucasian	Non-EA	NM_000060.4	c.511G>A	p.Ala171Thr	c.511G>A	p.Ala171Thr	AR	M+M	homo	Female	Biotinidase deficiency
**AP0521**	***BTD***	27657684	F212	P216	/	subject 14	Caucasian	Non-EA	NM_000060.4	c.1368A>C	p.Gln456His	c.98_104delin	p.Cys33Phefs*	AR	M+T	compound I	Female	Biotinidase deficiency
**AP0522**	***BTD***	27657684	F213	P217	/	subject 16	Caucasian	Non-EA	NM_000060.4	c.98_104delinsTCC	p.Cys33Phefs*36	c.511G>A	p.Ala171Thr	AR	M+T	compound I	Male	Biotinidase deficiency
**AP0523**	***BTD***	27657684	F214	P218	/	subject 17	Caucasian	Non-EA	NM_000060.4	c.1368A>C	p.Gln456His	c.1158G>A	p.Trp386*	AR	M+T	compound I	Female	Biotinidase deficiency
**AP0524**	***BTD***	27657684	F215	P219	/	subject 18	Caucasian	Non-EA	NM_000060.4	c.1368A>C	p.Gln456His	c.133G>A	p.Gly45Arg	AR	M+M	compound I	Male	Biotinidase deficiency
**AP0525**	***BTD***	27657684	F216	P220	/	subject 19	Caucasian	Non-EA	NM_000060.4	c.511G>A	p.Ala171Thr	c.1330G>C	p.Asp444His	AR	M+M	compound I	Male	Biotinidase deficiency
**AP0526**	***BTD***	27657684	F217	P221	/	subject 20	Caucasian	Non-EA	NM_000060.4	c.98_104delinsTCC	p.Cys33Phefs*36	c.1267T>C	p.Cys423Arg	AR	M+T	compound I	Male	Biotinidase deficiency
**AP0527**	***BTD***	27657684	F218	P222	/	subject 21	Caucasian	Non-EA	NM_000060.4	c.1368A>C	p.Gln456His	c.933delT	p.Ser311Argfs	AR	M+T	compound I	Female	Biotinidase deficiency
**AP0528**	***BTD***	27657684	F219	P223	/	subject 22	Caucasian	Non-EA	NM_000060.4	c.184G>T	p.Val62Leu	/	/	AD	M	compound I	Female	Biotinidase deficiency
**AP0529**	***BTD***	27657684	F220	P224	/	subject 23	Caucasian	Non-EA	NM_000060.4	c.1368A>C	p.Gln456His	c.1368A>C	p.Gln456His	AR	M+M	homo	Male	Biotinidase deficiency
**AP0530**	***BTD***	27657684	F221	P225	/	subject 24	Caucasian	Non-EA	NM_000060.4	c.701C>T	p.Thr234Ile	c.701C>T	p.Thr234Ile	AR	M+M	homo	Female	Biotinidase deficiency
**AP0531**	***BTD***	27657684	F222	P226	/	subject 25	Caucasian	Non-EA	NM_000060.4	c.1368A>C	p.Gln456His	c.98_104delin	p.Cys33Phefs*	AR	M+T	compound I	Male	Biotinidase deficiency
**AP0532**	***BTD***	27657684	F223	P227	/	subject 26	Caucasian	Non-EA	NM_000060.4	c.511G>A	p.Ala171Thr	c.511G>A	p.Ala171Thr	AR	M+M	homo	Female	Biotinidase deficiency
**AP0533**	***BTD***	27657684	F224	P228	/	subject 27	Caucasian	Non-EA	NM_000060.4	c.755A>G	p.Asp252Gly	c.933delT	p.Ser311Argfs	AR	M+T	compound I	Female	Biotinidase deficiency
**AP0534**	***BTD***	27657684	F225	P229	/	subject 28	Caucasian	Non-EA	NM_000060.4	c.511G>A	p.Ala171Thr	c.1330G>C	p.Asp444His	AR	M+M	compound I	Male	Biotinidase deficiency
**AP0535**	***BTD***	27657684	F226	P230	/	subject 29	Caucasian	Non-EA	NM_000060.4	c.1368A>C	p.Gln456His	c.933delT	p.Ser311Argfs	AR	M+T	compound I	Female	Biotinidase deficiency
**AP0536**	***BTD***	27657684	F227	P231	/	subject 30	Caucasian	Non-EA	NM_000060.4	c.511G>A	p.Ala171Thr	c.1330G>C	p.Asp444His	AR	M+M	compound I	Female	Biotinidase deficiency
**AP0537**	***BTD***	27657684	F228	P232	/	subject 31	Caucasian	Non-EA	NM_000060.4	c.755A>G	p.Asp252Gly	c.1368A>C	p.Gln456His	AR	M+M	compound I	Male	Biotinidase deficiency
**AP0538**	***BTD***	27657684	F229	P233	/	subject 32	Caucasian	Non-EA	NM_000060.4	c.511G>A	p.Ala171Thr	c.1330G>C	p.Asp444His	AR	M+M	compound I	Male	Biotinidase deficiency
**AP0539**	***BTD***	27657684	F230	P234	/	subject 33	Caucasian	Non-EA	NM_000060.4	c.1368A>C	p.Gln456His	c.1368A>C	p.Gln456His	AR	M+M	homo	Male	Biotinidase deficiency
**AP0540**	***BTD***	27657684	F231	P235	/	subject 34	Caucasian	Non-EA	NM_000060.4	c.511G>A	p.Ala171Thr	c.1330G>C	p.Asp444His	AR	M+M	compound I	Male	Biotinidase deficiency
**AP0541**	***BTD***	27657684	F232	P236	/	subject 35	Caucasian	Non-EA	NM_000060.4	c.511G>A	p.Ala171Thr	c.1330G>C	p.Asp444His	AR	M+M	compound I	Male	Biotinidase deficiency
**AP0542**	***BTD***	27657684	F233	P237	/	subject 37	Caucasian	Non-EA	NM_000060.4	c.98_104delinsTCC	p.Cys33Phefs*36	c.470G>A	p.Arg157His	AR	M+T	compound I	Male	Biotinidase deficiency
**AP0543**	***BTD***	27657684	F234	P238	/	subject 38	Caucasian	Non-EA	NM_000060.4	c.1368A>C	p.Gln456His	c.98_104delin	p.Cys33Phefs*	AR	M+T	compound I	Male	Biotinidase deficiency
**AP0544**	***BTD***	27657684	F235	P239	/	subject 39	Caucasian	Non-EA	NM_000060.4	c.1368A>C	p.Gln456His	c.511G>A	p.Ala171Thr	AR	M+M	compound I	Female	Biotinidase deficiency
**AP0545**	***BTD***	27657684	F236	P240	/	subject 41	Caucasian	Non-EA	NM_000060.4	c.1368A>C	p.Gln456His	c.470G>A	p.Arg157His	AR	M+M	compound I	Male	Biotinidase deficiency
**AP0546**	***BTD***	27657684	F237	P241	/	subject 42	Caucasian	Non-EA	NM_000060.4	c.755A>G	p.Asp252Gly	c.1368A>C	p.Gln456His	AR	M+M	compound I	Male	Biotinidase deficiency
**AP0547**	***BTD***	27657684	F238	P242	/	subject 43	Caucasian	Non-EA	NM_000060.4	c.511G>A	p.Ala171Thr	c.1330G>C	p.Asp444His	AR	M+M	compound I	Male	Biotinidase deficiency
**AP0548**	***BTD***	27657684	F239	P243	/	subject 44	Caucasian	Non-EA	NM_000060.4	c.341G>T	p.Gly114Val	c.701C>T	p.Thr234Ile	AR	M+M	compound I	Male	Biotinidase deficiency
**AP0549**	***BTD***	27329734	F240	P244	/	patient 5	Caucasian	Non-EA	NM_000060.4	c.1101C>T	p.=	/	/	AD	M	heter	/	Biotinidase deficiency
**AP0550**	***BTD***	27329734	F241	P245	/	patient 6	Caucasian	Non-EA	NM_000060.4	c.1330G>C	p.Asp444His	/	/	AD	M	heter	/	Biotinidase deficiency
**AP0551**	***BTD***	27329734	F242	P246	/	patient 7	Caucasian	Non-EA	NM_000060.4	c.1595C>T	p.Thr532Met	/	/	AD	M	heter	/	Biotinidase deficiency
**AP0552**	***BTD***	27329734	F243	P247	/	patient 9	Caucasian	Non-EA	NM_000060.4	c.1330G>C	p.Asp444His	/	/	AD	M	heter	/	Biotinidase deficiency
**AP0553**	***BTD***	27329734	F244	P248	/	patient 11	Caucasian	Non-EA	NM_000060.4	c.1330G>C	p.Asp444His	/	/	AD	M	heter	/	Biotinidase deficiency
**AP0554**	***BTD***	27329734	F245	P249	/	patient 13	Caucasian	Non-EA	NM_000060.4	c.1595C>T	p.Thr532Met	/	/	AD	M	heter	/	Biotinidase deficiency
**AP0555**	***BTD***	27329734	F246	P250	/	patient 14	Caucasian	Non-EA	NM_000060.4	c.133G>A	p.Gly45Arg	c.1368A>C	p.Gln456His	AR	M+M	compound I	/	Biotinidase deficiency
**AP0556**	***BTD***	27329734	F247	P251	/	patient 15	Caucasian	Non-EA	NM_000060.4	c.595G>A	p.Val199Met	/	/	AD	M	heter	/	Biotinidase deficiency
**AP0557**	***BTD***	27329734	F248	P252	/	patient 16	Caucasian	Non-EA	NM_000060.4	c.1330G>C	p.Asp444His	c.1330G>C	p.Asp444His	AR	M+M	homo	/	Biotinidase deficiency
**AP0558**	***BTD***	27329734	F249	P253	/	patient 17	Caucasian	Non-EA	NM_000060.4	c.1330G>C	p.Asp444His	c.1330G>C	p.Asp444His	AR	M+M	homo	/	Biotinidase deficiency
**AP0559**	***BTD***	27329734	F250	P254	/	patient 18	Caucasian	Non-EA	NM_000060.4	c.1330G>C	p.Asp444His	c.595G>A	p.Val199Met	AR	M+M	compound I	/	Biotinidase deficiency
**AP0560**	***BTD***	27329734	F251	P255	/	patient 19	Caucasian	Non-EA	NM_000060.4	c.968A>G	p.His323Arg	c.[261C>T;895	p.[= ;Ala299Pr	AR	M+M	compound I	/	Biotinidase deficiency
**AP0561**	***BTD***	27329734	F252	P256	/	patient 20	Caucasian	Non-EA	NM_000060.4	c.364A>G	p.Arg122Gly	c.1330G>C	p.Asp444His	AR	M+M	compound I	/	Biotinidase deficiency
**AP0562**	***BTD***	27329734	F253	P257	/	patient 21	Caucasian	Non-EA	NM_000060.4	c.1330G>C	p.Asp444His	c.1595C>T	p.Thr532Met	AR	M+M	compound I	/	Biotinidase deficiency
**AP0563**	***BTD***	27329734	F254	P258	/	patient 22	Caucasian	Non-EA	NM_000060.4	c.1330G>C	p.Asp444His	c.1330G>C	p.Asp444His	AR	M+M	homo	/	Biotinidase deficiency
**AP0564**	***BTD***	27329734	F255	P259	/	patient 23	Caucasian	Non-EA	NM_000060.4	c.1330G>C	p.Asp444His	c.1330G>C	p.Asp444His	AR	M+M	homo	/	Biotinidase deficiency
**AP0565**	***BTD***	27329734	F256	P260	/	patient 24	Caucasian	Non-EA	NM_000060.4	c.1330G>C	p.Asp444His	c.1368A>C	p.Gln456His	AR	M+M	compound I	/	Biotinidase deficiency
**AP0566**	***BTD***	27329734	F257	P261	/	patient 25	Caucasian	Non-EA	NM_000060.4	c.1330G>C	p.Asp444His	c.1368A>C	p.Gln456His	AR	M+M	compound I	/	Biotinidase deficiency
**AP0567**	***BTD***	27329734	F258	P262	/	patient 26	Caucasian	Non-EA	NM_000060.4	c.1330G>C	p.Asp444His	c.1368A>C	p.Gln456His	AR	M+M	compound I	/	Biotinidase deficiency
**AP0568**	***BTD***	27329734	F259	P263	/	patient 27	Caucasian	Non-EA	NM_000060.4	c.645C>T	p.=	c.1368A>C	p.Gln456His	AR	M+T	compound I	/	Biotinidase deficiency
**AP0569**	***BTD***	27329734	F260	P264	/	patient 28	Caucasian	Non-EA	NM_000060.4	c.631C>T	p.Arg211Cys	c.1330G>C	p.Asp444His	AR	M+M	compound I	/	Biotinidase deficiency
**AP0570**	***BTD***	27329734	F261	P265	/	patient 29	Caucasian	Non-EA	NM_000060.4	c.470G>A	p.Arg157His	c.143G>A	p.Gly480Glu	AR	M+M	compound I	/	Biotinidase deficiency
**AP0571**	***BTD***	27329734	F262	P266	/	patient 30	Caucasian	Non-EA	NM_000060.4	c.1330G>C	p.Asp444His	c.1595C>T	p.Thr532Met	AR	M+M	compound I	/	Biotinidase deficiency
**AP0572**	***BTD***	27329734	F263	P267	/	patient 31	Caucasian	Non-EA	NM_000060.4	c.1330G>C	p.Asp444His	c.1330G>C	p.Asp444His	AR	M+M	homo	/	Biotinidase deficiency
**AP0573**	***BTD***	27329734	F264	P268	/	patient 32	Caucasian	Non-EA	NM_000060.4	c.470G>A	p.Arg157His	c.1330G>C	p.Asp444His	AR	M+M	compound I	/	Biotinidase deficiency
**AP0574**	***BTD***	27329734	F265	P269	/	patient 33	Caucasian	Non-EA	NM_000060.4	c.1330G>C	p.Asp444His	c.1368A>C	p.Gln456His	AR	M+M	compound I	/	Biotinidase deficiency
**AP0575**	***BTD***	27329734	F266	P270	/	patient 34	Caucasian	Non-EA	NM_000060.4	c.1330G>C	p.Asp444His	c.1330G>C	p.Asp444His	AR	M+M	homo	/	Biotinidase deficiency
**AP0576**	***BTD***	27329734	F267	P271	/	patient 35	Caucasian	Non-EA	NM_000060.4	c.1368A>C	p.Gln456His	c.1330G>C	p.Asp444His	AR	M+M	compound I	/	Biotinidase deficiency
**AP0577**	***BTD***	27329734	F268	P272	/	patient 36	Caucasian	Non-EA	NM_000060.4	c.1330G>C	p.Asp444His	c.631C>T	p.Arg211Cys	AR	M+M	compound I	/	Biotinidase deficiency
**AP0578**	***BTD***	27329734	F269	P273	/	patient 37	Caucasian	Non-EA	NM_000060.4	c.1330G>C	p.Asp444His	c.1090dupA	p.Ile364fs	AR	M+T	compound I	/	Biotinidase deficiency
**AP0579**	***BTD***	27329734	F270	P274	/	patient 38	Caucasian	Non-EA	NM_000060.4	c.1330G>C	p.Asp444His	c.1330G>C	p.Asp444His	AR	M+M	homo	/	Biotinidase deficiency
**AP0580**	***BTD***	27329734	F271	P275	/	patient 39	Caucasian	Non-EA	NM_000060.4	c.1330G>C	p.Asp444His	c.1330G>C	p.Asp444His	AR	M+M	homo	/	Biotinidase deficiency
**AP0581**	***BTD***	27329734	F272	P276	/	patient 40	Caucasian	Non-EA	NM_000060.4	c.1330G>C	p.Asp444His	c.1368A>C	p.Gln456His	AR	M+M	compound I	/	Biotinidase deficiency
**AP0582**	***BTD***	27329734	F273	P277	/	patient 41	Caucasian	Non-EA	NM_000060.4	c.1330G>C	p.Asp444His	c.1368A>C	p.Gln456His	AR	M+M	compound I	/	Biotinidase deficiency
**AP0583**	***BTD***	27329734	F274	P278	/	patient 42	Caucasian	Non-EA	NM_000060.4	c.1330G>C	p.Asp444His	c.1595C>T	p.Thr532Met	AR	M+M	compound I	/	Biotinidase deficiency
**AP0584**	***BTD***	27329734	F275	P279	/	patient 43	Caucasian	Non-EA	NM_000060.4	c.1330G>C	p.Asp444His	c.1330G>C	p.Asp444His	AR	M+M	homo	/	Biotinidase deficiency
**AP0585**	***BTD***	27329734	F276	P280	/	patient 44	Caucasian	Non-EA	NM_000060.4	c.1330G>C	p.Asp444His	c.1612C>T	p.Arg538Cys	AR	M+M	compound I	/	Biotinidase deficiency
**AP0586**	***BTD***	27329734	F277	P281	/	patient 45	Caucasian	Non-EA	NM_000060.4	c.1330G>C	p.Asp444His	c.1368A>C	p.Gln456His	AR	M+M	compound I	/	Biotinidase deficiency
**AP0587**	***BTD***	27329734	F278	P282	/	patient 46	Caucasian	Non-EA	NM_000060.4	c.1330G>C	p.Asp444His	c.645C>T	p.=	AR	M+T	compound I	/	Biotinidase deficiency
**AP0588**	***BTD***	27329734	F279	P283	/	patient 47	Caucasian	Non-EA	NM_000060.4	c.1330G>C	p.Asp444His	c.1368A>C	p.Gln456His	AR	M+M	compound I	/	Biotinidase deficiency
**AP0589**	***BTD***	27329734	F280	P284	/	patient 48	Caucasian	Non-EA	NM_000060.4	c.1330G>C	p.Asp444His	c.1368A>C	p.Gln456His	AR	M+M	compound I	/	Biotinidase deficiency
**AP0590**	***BTD***	27329734	F281	P285	/	patient 49	Caucasian	Non-EA	NM_000060.4	c.1612C>T	p.Arg538Cys	c.459+4A>G	p.?	AR	M+T	compound I	/	Biotinidase deficiency
**AP0591**	***BTD***	27329734	F282	P286	/	patient 50	Caucasian	Non-EA	NM_000060.4	c.100G>A	p.Gly34Ser	c.1330G>C	p.Asp444His	AR	M+M	compound I	/	Biotinidase deficiency
**AP0592**	***BTD***	27329734	F283	P287	/	patient 51	Caucasian	Non-EA	NM_000060.4	c.1595C>T	p.Thr532Met	c.1330G>C	p.Asp444His	AR	M+M	compound I	/	Biotinidase deficiency
**AP0593**	***BTD***	27329734	F284	P288	/	patient 52	Caucasian	Non-EA	NM_000060.4	c.1330G>C	p.Asp444His	c.1368A>C	p.Gln456His	AR	M+M	compound I	/	Biotinidase deficiency
**AP0594**	***BTD***	27329734	F285	P289	/	patient 53	Caucasian	Non-EA	NM_000060.4	c.1330G>C	p.Asp444His	c.1595C>T	p.Thr532Met	AR	M+M	compound I	/	Biotinidase deficiency
**AP0595**	***BTD***	27329734	F286	P290	/	patient 54	Caucasian	Non-EA	NM_000060.4	c.98_104delinsTCC	p.Cys33Phefs*36	c.1330G>C	p.Asp444His	AR	M+T	compound I	/	Biotinidase deficiency
**AP0596**	***BTD***	27329734	F287	P291	/	patient 55	Caucasian	Non-EA	NM_000060.4	c.1525C>T	p.Pro509Ser	c.1595C>T	p.Thr532Met	AR	M+M	compound I	/	Biotinidase deficiency
**AP0597**	***BTD***	27329734	F288	P292	/	patient 56	Caucasian	Non-EA	NM_000060.4	c.1330G>C	p.Asp444His	c.1368A>C	p.Gln456His	AR	M+M	compound I	/	Biotinidase deficiency
**AP0598**	***BTD***	27329734	F289	P293	/	patient 57	Caucasian	Non-EA	NM_000060.4	c.470G>A	p.Arg157His	c.470G>A	p.Arg157His	AR	M+M	homo	/	Biotinidase deficiency
**AP0599**	***BTD***	27329734	F290	P294	/	patient 58	Caucasian	Non-EA	NM_000060.4	c.1368A>C	p.Gln456His	c.1368A>C	p.Gln456His	AR	M+M	homo	/	Biotinidase deficiency
**AP0600**	***BTD***	27329734	F291	P295	/	patient 59	Caucasian	Non-EA	NM_000060.4	c.1368A>C	p.Gln456His	c.1368A>C	p.Gln456His	AR	M+M	homo	/	Biotinidase deficiency
**AP0601**	***BTD***	27329734	F292	P296	/	patient 60	Caucasian	Non-EA	NM_000060.4	c.1252T>C	p.Cys418Arg	c.1595C>T	p.Thr532Met	AR	M+M	compound I	/	Biotinidase deficiency
**AP0602**	***BTD***	27329734	F293	P297	/	patient 61	Caucasian	Non-EA	NM_000060.4	c.1330G>C	p.Asp444His	c.511G>A	p.Ala171Thr	AR	M+M	compound I	/	Biotinidase deficiency
**AP0603**	***BTD***	27207447	F294	P298	/		Caucasian	Non-EA	NM_000060.4	c.643C>T	p.Leu215Phe	c.1612C>T	p.Arg538Cys	AR	M+M	compound I	Female	Biotinidase deficiency
**AP0604**	***BTD***	27014582	F295	P299	/		Caucasian	Non-EA	NM_000060.4	del exon2-4	p.?	/	/	AD	M	heter		Biotinidase deficiency
**AP0605**	***BTD***	26810761	F296	P300	/	1	Caucasian	Non-EA	NM_000060.4	c.192G>C	p.Glu64Asp	c.1330G>C	p.Asp444His	AR	M+M	compound I	/	Biotinidase deficiency
**AP0606**	***BTD***	26810761	F297	P301	/	2	Caucasian	Non-EA	NM_000060.4	c.245C>T	p.Ala82Val	c.1330G>C	p.Asp444His	AR	M+M	compound I	/	Biotinidase deficiency
**AP0607**	***BTD***	26810761	F298	P302	/	3	Caucasian	Non-EA	NM_000060.4	c.299C>T	p.Ala100Val	c.1330G>C	p.Asp444His	AR	M+M	compound I	/	Biotinidase deficiency
**AP0608**	***BTD***	26810761	F299	P303	/	4	Caucasian	Non-EA	NM_000060.4	c.321T>G	p.Ile107Met	c.1330G>C	p.Asp444His	AR	M+M	compound I	/	Biotinidase deficiency
**AP0609**	***BTD***	26810761	F300	P304	/	5	Caucasian	Non-EA	NM_000060.4	c.326dupT	p.Phe110Valfs*42	/	/	AD	M	heter	/	Biotinidase deficiency
**AP0610**	***BTD***	26810761	F301	P305	/	6	Caucasian	Non-EA	NM_000060.4	c.356A>G	p.Asn119Ser	c.1459delT	p.Trp487Glyfs*	AR	M+T	compound I	/	Biotinidase deficiency
**AP0611**	***BTD***	26810761	F302	P306	/	7	Caucasian	Non-EA	NM_000060.4	c.406delC	p.Gln136Argfs*23	c.1330G>C	p.Asp444His	AR	M+T	compound I	/	Biotinidase deficiency
**AP0612**	***BTD***	26810761	F303	P307	/	8	Caucasian	Non-EA	NM_000060.4	c.582C>G	p.Phe194Leu	c.1330G>C	p.Asp444His	AR	M+M	compound I	/	Biotinidase deficiency
**AP0613**	***BTD***	26810761	F304	P308	/	9	Caucasian	Non-EA	NM_000060.4	c.625C>T	p.Arg209Cys	c.1368A>C	p.Gln456His	AR	M+M	compound I	/	Biotinidase deficiency
**AP0614**	***BTD***	26810761	F305	P309	/	10	Caucasian	Non-EA	NM_000060.4	c.631C>T	p.Arg211Cys	c.1330G>C	p.Asp444His	AR	M+M	compound I	/	Biotinidase deficiency
**AP0615**	***BTD***	26810761	F306	P310	/	11	Caucasian	Non-EA	NM_000060.4	c.646T>A	p.Tyr216Asn	c.1330G>C	p.Asp444His	AR	M+M	compound I	/	Biotinidase deficiency
**AP0616**	***BTD***	26810761	F307	P311	/	12	Caucasian	Non-EA	NM_000060.4	c.664G>C	p.Asp222His	c.1330G>C	p.Asp444His	AR	M+M	compound I	/	Biotinidase deficiency
**AP0617**	***BTD***	26810761	F308	P312	/	13	Caucasian	Non-EA	NM_000060.4	c.692delC	p.Phe232Leufs*3	c.1330G>C	p.Asp444His	AR	M+T	compound I	/	Biotinidase deficiency
**AP0618**	***BTD***	26810761	F309	P313	/	14	Caucasian	Non-EA	NM_000060.4	c.695T>C	p.Phe232Ser	c.1330G>C	p.Asp444His	AR	M+M	compound I	/	Biotinidase deficiency
**AP0619**	***BTD***	26810761	F310	P314	/	15	Caucasian	Non-EA	NM_000060.4	c.709G>A	p.Ala237Thr	c.1330G>C	p.Asp444His	AR	M+M	compound I	/	Biotinidase deficiency
**AP0620**	***BTD***	26810761	F311	P315	/	16	Caucasian	Non-EA	NM_000060.4	c.743T>C	p.Ile248Thr	c.528G>T	p.Lys176Asn	AR	M+M	compound I	/	Biotinidase deficiency
**AP0621**	***BTD***	26810761	F312	P316	/	17	Caucasian	Non-EA	NM_000060.4	c.758C>T	p.Pro253Leu	c.1489C>T	p.Pro497Ser	AR	M+M	compound I	/	Biotinidase deficiency
**AP0622**	***BTD***	26810761	F313	P317	/	18	Caucasian	Non-EA	NM_000060.4	c.770T>A	p.Val257Asp	c.1330G>C	p.Asp444His	AR	M+M	compound I	/	Biotinidase deficiency
**AP0623**	***BTD***	26810761	F314	P318	/	19	Caucasian	Non-EA	NM_000060.4	c.783C>G	p.Tyr261*	c.1330G>C	p.Asp444His	AR	M+T	compound I	/	Biotinidase deficiency
**AP0624**	***BTD***	26810761	F315	P319	/	20	Caucasian	Non-EA	NM_000060.4	c.814T>G	p.Trp272Gly	c.1330G>C	p.Asp444His	AR	M+M	compound I	/	Biotinidase deficiency
**AP0625**	***BTD***	26810761	F316	P320	/	21	Caucasian	Non-EA	NM_000060.4	c.836T>A	p.Leu279*	c.310-15delT	p.?	AR	T+T	compound I	/	Biotinidase deficiency
**AP0626**	***BTD***	26810761	F317	P321	/	22	Caucasian	Non-EA	NM_000060.4	c.856A>G	p.Lys286Glu	c.1330G>C	p.Asp444His	AR	M+M	compound I	/	Biotinidase deficiency
**AP0627**	***BTD***	26810761	F318	P322	/	23	Caucasian	Non-EA	NM_000060.4	c.865G>C	p.Ala289Pro	c.1330G>C	p.Asp444His	AR	M+M	compound I	/	Biotinidase deficiency
**AP0628**	***BTD***	26810761	F319	P323	/	25	Caucasian	Non-EA	NM_000060.4	c.895G>C	p.Ala299Pro	c.1413T>C	p.Cys471Cys	AR	M+M	compound I	/	Biotinidase deficiency
**AP0629**	***BTD***	26810761	F320	P324	/	26	Caucasian	Non-EA	NM_000060.4	c.1001T>A	p.Ile334Asn	c.1330G>C	p.Asp444His	AR	M+M	compound I	/	Biotinidase deficiency
**AP0630**	***BTD***	26810761	F321	P325	/	27	Caucasian	Non-EA	NM_000060.4	c.1046A>C	p.Asn349Thr	c.1330G>C	p.Asp444His	AR	M+M	compound I	/	Biotinidase deficiency
**AP0631**	***BTD***	26810761	F322	P326	/	28	Caucasian	Non-EA	NM_000060.4	c.1096_1097dupTC	p.Gly367Glnfs*23	c.1330G>C	p.Asp444His	AR	M+T	compound I	/	Biotinidase deficiency
**AP0632**	***BTD***	26810761	F323	P327	/	29	Caucasian	Non-EA	NM_000060.4	c.1126C>T	p.Gln376*	c.1612C> T	p.Arg538Cys	AR	M+T	compound I	/	Biotinidase deficiency
**AP0633**	***BTD***	26810761	F324	P328	/	30	Caucasian	Non-EA	NM_000060.4	c.1241_1252del12bp	p.Tyr414_Val417d	c.1330G>C	p.Asp444His	AR	M+M	compound I	/	Biotinidase deficiency
**AP0634**	***BTD***	26810761	F325	P329	/	31	Caucasian	Non-EA	NM_000060.4	c.1252T>C	p.Cys418Arg	c.1330G>C	p.Asp444His	AR	M+M	compound I	/	Biotinidase deficiency
**AP0635**	***BTD***	26810761	F326	P330	/	32	Caucasian	Non-EA	NM_000060.4	c.1309C>G	p.Leu437Val	c.1330G>C	p.Asp444His	AR	M+M	compound I	/	Biotinidase deficiency
**AP0636**	***BTD***	26810761	F327	P331	/	33	Caucasian	Non-EA	NM_000060.4	c.1334G>A	p.Gly445Arg	c.1330G>C	p.Asp444His	AR	M+M	compound I	/	Biotinidase deficiency
**AP0637**	***BTD***	26810761	F328	P332	/	34	Caucasian	Non-EA	NM_000060.4	c.1352_1353delGC	p.Gly451Aspfs*32	c.1330G>C	p.Asp444His	AR	M+T	compound I	/	Biotinidase deficiency
**AP0638**	***BTD***	26810761	F329	P333	/	35	Caucasian	Non-EA	NM_000060.4	c.1432G>A	p.Ala478Thr	c.755A>G	p.Asp252Gly	AR	M+M	compound I	/	Biotinidase deficiency
**AP0639**	***BTD***	26810761	F330	P334	/	36	Caucasian	Non-EA	NM_000060.4	c.1455C>G	p.His485Gln	c.1330G>C	p.Asp444His	AR	M+M	compound I	/	Biotinidase deficiency
**AP0640**	***BTD***	26810761	F331	P335	/	37	Caucasian	Non-EA	NM_000060.4	c.1458delG	p.W487Gfs*14	c.278A>G	p.Tyr93Cys	AR	M+M	compound I	/	Biotinidase deficiency
**AP0641**	***BTD***	26810761	F332	P336	/	38	Caucasian	Non-EA	NM_000060.4	c.1459delT	p.W487Gfs*14	c.356A>G	p.Asn119Ser	AR	M+M	compound I	/	Biotinidase deficiency
**AP0642**	***BTD***	26810761	F333	P337	/	40	Caucasian	Non-EA	NM_000060.4	c.1526C>G	p.Pro509Arg	c.1330G>C	p.Asp444His	AR	M+M	compound I	/	Biotinidase deficiency
**AP0643**	***BTD***	26810761	F334	P338	/	41	Caucasian	Non-EA	NM_000060.4	c.1612C>A	p.Arg538Ser	c.1330G>C	p.Asp444His	AR	M+M	compound I	/	Biotinidase deficiency
**AP0644**	***BTD***	26810761	F335	P339	/	42	Caucasian	Non-EA	NM_000060.4	c.1613G>A	p.Arg538His	c.1062G>A	p.=	AR	M+T	compound I	/	Biotinidase deficiency
**AP0645**	***BTD***	26810761	F336	P340	/	43	Caucasian	Non-EA	NM_000060.4	c.1628A>T	p.Asp543Val	c.310-15delT	p.?	AR	M+T	compound I	/	Biotinidase deficiency
**AP0646**	***BTD***	26810761	F337	P341	/	44	Caucasian	Non-EA	NM_000060.4	c.1629C>A	p.Asp543Glu	c.1330G>C	p.Asp444His	AR	M+M	compound I	/	Biotinidase deficiency
**AP0647**	***BTD***	26656798	F338	P342	/		Caucasian	Non-EA	NM_000060.4	c.941A>G	p.His314Arg	/	/	AD	M	heter	/	Biotinidase deficiency
**AP0648**	***BTD***	26203071	F339	P343	/		Caucasian	Non-EA	NM_000060.4	c.1316C>A	p.Ala439Asp	c.1413T>C	p.Cys471Cys	AR	M+M	compound I	Male	Biotinidase deficiency
**AP0649**	***BTD***	25967232	F340	P344	/	child 1	Caucasian	Non-EA	NM_000060.4	c.1455C>G	p.His485Gln	c.1455C>G	p.His485Gln	AR	M+M	homo	/	Biotinidase deficiency
**AP0650**	***BTD***	25967232	F341	P345	/	child 2	Caucasian	Non-EA	NM_000060.4	c.842C>T	p.Ala281Val	c.1330G>C	p.Asp444His	AR	M+M	compound I	/	Biotinidase deficiency
**AP0651**	***BTD***	25967232	F342	P346	/	child 3	Caucasian	Non-EA	NM_000060.4	c.1330G>C	p.Asp444His	/	/	AD	M	heter	/	Biotinidase deficiency
**AP0652**	***BTD***	25967232	F343	P347	/	child 4	Caucasian	Non-EA	NM_000060.4	c.529G>A	p.Glu177Lys	c.1595C>T	p.Thr532Met	AR	M+M	compound I	/	Biotinidase deficiency
**AP0653**	***BTD***	25967232	F344	P348	/	child 5	Caucasian	Non-EA	NM_000060.4	c.1629C>A	p.Asp543Glu	/	/	AD	M	heter	/	Biotinidase deficiency
**AP0654**	***BTD***	25967232	F345	P349	/	child 6	Caucasian	Non-EA	NM_000060.4	c.1330G>C	p.Asp444His	c.1330G>C	p.Asp444His	AR	M+M	homo	/	Biotinidase deficiency
**AP0655**	***BTD***	25967232	F346	P350	/	child 7	Caucasian	Non-EA	NM_000060.4	c.1330G>C	p.Asp444His	c.1330G>C	p.Asp444His	AR	M+M	homo	/	Biotinidase deficiency
**AP0656**	***BTD***	25967232	F347	P351	/	child 8	Caucasian	Non-EA	NM_000060.4	c.1489C>T	p.Pro497Ser	c.1330G>C	p.Asp444His	AR	M+M	compound I	/	Biotinidase deficiency
**AP0657**	***BTD***	25795614	F348	P352	/		Caucasian	Non-EA	NM_000060.4	c.1330G>C	p.Asp444His	c.1475C>T	p.Thr492Ile	AR	M+M	compound I	/	Biotinidase deficiency
**AP0658**	***BTD***	25754625	F349	P353	/	FNA	Caucasian	Non-EA	NM_000060.4	c.731C>T	p.Thr244Ile	c.1330G>C	p.Asp444His	AR	M+M	compound I	/	Biotinidase deficiency
**AP0659**	***BTD***	25754625	F350	P354	/	YC	Caucasian	Non-EA	NM_000060.4	c.731C>T	p.Thr244Ile	c.1330G>C	p.Asp444His	AR	M+M	compound I	/	Biotinidase deficiency
**AP0660**	***BTD***	25754625	F351	P355	/	EAO	Caucasian	Non-EA	NM_000060.4	c.731C>T	p.Thr244Ile	c.1330G>C	p.Asp444His	AR	M+M	compound I	/	Biotinidase deficiency
**AP0661**	***BTD***	25754625	F352	P356	/	SBB	Caucasian	Non-EA	NM_000060.4	c.1320delG	p.Val442Serfs*59	c.1330G>C	p.Asp444His	AR	M+T	compound I	/	Biotinidase deficiency
**AP0662**	***BTD***	25754625	F353	P357	/	TD	Caucasian	Non-EA	NM_000060.4	c.1438G>A	p.Gly480Arg	c.1438G>A	p.Gly480Arg	AR	M+M	homo	/	Biotinidase deficiency
**AP0663**	***BTD***	25754625	F354	P358	/	DK	Caucasian	Non-EA	NM_000060.4	c.1212_1222del11	p.Val406Glyfs*35	c.1330G>C	p.Asp444His	AR	M+T	compound I	/	Biotinidase deficiency
**AP0664**	***BTD***	25754625	F355	P359	/	MK	Caucasian	Non-EA	NM_000060.4	c.1369G>C	p.Val457Leu	/	/	AD	M	heter	/	Biotinidase deficiency
**AP0665**	***BTD***	25754625	F356	P360	/	HK	Caucasian	Non-EA	NM_000060.4	c.428G>T	p.Cys143Phe	c.428G>T	p.Cys143Phe	AR	M+M	homo	/	Biotinidase deficiency
**AP0666**	***BTD***	25754625	F358	P362	/	AT	Caucasian	Non-EA	NM_000060.4	c.98_104delinsTCC	p.Cys33Phefs*36	c.98_104delin	p.Cys33Phefs*	AR	T+T	homo	/	Biotinidase deficiency
**AP0667**	***BTD***	25754625	F359	P363	/	ENK	Caucasian	Non-EA	NM_000060.4	c.98_104delinsTCC	p.Cys33Phefs*36	c.98_104delin	p.Cys33Phefs*	AR	T+T	homo	/	Biotinidase deficiency
**AP0668**	***BTD***	25754625	F360	P364	/	EFU	Caucasian	Non-EA	NM_000060.4	c.98_104delinsTCC	p.Cys33Phefs*36	c.98_104delin	p.Cys33Phefs*	AR	T+T	homo	/	Biotinidase deficiency
**AP0669**	***BTD***	25754625	F361	P365	/	HA	Caucasian	Non-EA	NM_000060.4	c.1368A>C	p.Gln456His	c.1368A>C	p.Gln456His	AR	M+M	homo	/	Biotinidase deficiency
**AP0670**	***BTD***	25754625	F362	P366	/	IK	Caucasian	Non-EA	NM_000060.4	c.98_104delinsTCC	p.Cys33Phefs*36	c.98_104delin	p.Cys33Phefs*	AR	T+T	homo	/	Biotinidase deficiency
**AP0671**	***BTD***	25754625	F363	P367	/	MEG	Caucasian	Non-EA	NM_000060.4	c.1595C>T	p.Thr532Met	c.1595C>T	p.Thr532Met	AR	M+M	homo	/	Biotinidase deficiency
**AP0672**	***BTD***	25754625	F364	P368	/	RK	Caucasian	Non-EA	NM_000060.4	c.98_104delinsTCC	p.Cys33Phefs*36	c.98_104delin	p.Cys33Phefs*	AR	T+T	homo	/	Biotinidase deficiency
**AP0673**	***BTD***	25754625	F365	P369	/	SC	Caucasian	Non-EA	NM_000060.4	c.171T>G	p.Tyr57*	c.98_104delin	p.Cys33Phefs*	AR	T+T	compound I	/	Biotinidase deficiency
**AP0674**	***BTD***	25174816	F366	P370	/	P986	Caucasian	Non-EA	NM_000060.4	c.159C>A	p.His53Gln	c.160G>T	p.Glu54*	AR	M+T	compound I	Male	Biotinidase deficiency
**AP0675**	***BTD***	25174816	F367	P371	/	P1020	Caucasian	Non-EA	NM_000060.4	c.283C>T	p.Gln95*	c.1330G>C	p.Asp444His	AR	M+T	compound I	Female	Biotinidase deficiency
**AP0676**	***BTD***	25174816	F368	P372	/	P1005	Caucasian	Non-EA	NM_000060.4	c.212T>C	p.Leu71Pro	c.99C>T	p.=	AR	M+T	compound I	Male	Biotinidase deficiency
**AP0677**	***BTD***	25174816	F369	P373	/	P949A	Caucasian	Non-EA	NM_000060.4	c.341G>T	p.Gly114Val	c.341G>T	p.Gly114Val	AR	M+M	homo	Male	Biotinidase deficiency
**AP0678**	***BTD***	25174816	F370	P374	/	P965	Caucasian	Non-EA	NM_000060.4	c.654G>C	p.Glu218Asp	c.1052del C	p.Thr351Lysfs	AR	M+T	compound I	Male	Biotinidase deficiency
**AP0679**	***BTD***	25174816	F371	P375	/	P1019	Caucasian	Non-EA	NM_000060.4	c.734G>A	p.Cys245Tyr	c.1595C>T	p.Thr532Met	AR	M+M	compound I	Male	Biotinidase deficiency
**AP0680**	***BTD***	25174816	F372	P376	/	P1021	Caucasian	Non-EA	NM_000060.4	c.757C>T	p.Pro253Ser	c.98_104delin	p.Cys33Phefs*	AR	M+T	compound I	Female	Biotinidase deficiency
**AP0681**	***BTD***	25174816	F373	P377	/	P981	Caucasian	Non-EA	NM_000060.4	c.1271G>A	p.Cys424Tyr	c.1271G>A	p.Cys424Tyr	AR	M+M	compound I	Male	Biotinidase deficiency
**AP0682**	***BTD***	25174816	F374	P378	/	P987	Caucasian	Non-EA	NM_000060.4	c.1531G>C	p.Q511E	c.511G>A	p.Ala171Thr	AR	M+M	compound I	Male	Biotinidase deficiency
**AP0683**	***BTD***	25174816	F375	P379	/	P980	Caucasian	Non-EA	NM_000060.4	c.133G>A	p.Gly45Arg	c.133G>A	p.Gly45Arg	AR	M+M	homo	Male	Biotinidase deficiency
**AP0684**	***BTD***	25174816	F376	P380	/	P906	Caucasian	Non-EA	NM_000060.4	c.235C>T	p.Arg79Cys	c.470G>A	p.Arg157His	AR	M+M	compound I	Male	Biotinidase deficiency
**AP0685**	***BTD***	25174816	F377	P381	/	P1002	Caucasian	Non-EA	NM_000060.4	c.641A>G	p.Asn214Ser	c.734G>A	p.Cys245Tyr	AR	M+M	compound I	Male	Biotinidase deficiency
**AP0686**	***BTD***	25174816	F378	P382	/	P1018	Caucasian	Non-EA	NM_000060.4	c.1171C>T	p.Pro391Ser	c.1330G>C	p.Asp444His	AR	M+M	compound I	Male	Biotinidase deficiency
**AP0687**	***BTD***	24797656	F379	P383	/	1	Caucasian	Non-EA	NM_000060.4	c.257T>G	p.Met86Arg	c.1368A>C	p.Gln456His	AR	M+M	compound I	Female	Biotinidase deficiency
**AP0688**	***BTD***	24797656	F380	P384	/	2	Caucasian	Non-EA	NM_000060.4	c.455C>G	p.Thr152Arg	c.1330G>C	p.Asp444His	AR	M+M	compound I	Female	Biotinidase deficiency
**AP0689**	***BTD***	24797656	F381	P385	/	3	Caucasian	Non-EA	NM_000060.4	c.626G>A	p.Arg209His	c.1368A>C	p.Gln456His	AR	M+M	compound I	Female	Biotinidase deficiency
**AP0690**	***BTD***	24797656	F382	P386	/	4	Caucasian	Non-EA	NM_000060.4	c.683A>G	p.Asp228Gly	c.1330G>C	p.Asp444His	AR	M+M	compound I	Male	Biotinidase deficiency
**AP0691**	***BTD***	24797656	F383	P387	/	5	Caucasian	Non-EA	NM_000060.4	c.701C>T	p.Thr234Ile	c.1330G>C	p.Asp444His	AR	M+M	compound I	Female	Biotinidase deficiency
**AP0692**	***BTD***	24797656	F384	P388	/	6	Caucasian	Non-EA	NM_000060.4	c.701C>T	p.Thr234Ile	c.701C>T	p.Thr234Ile	AR	M+M	homo	Female	Biotinidase deficiency
**AP0693**	***BTD***	24797656	F385	P389	/	7	Caucasian	Non-EA	NM_000060.4	c.898A>C	p.Asn300His	c.1330G>C	p.Asp444His	AR	M+M	compound I	Female	Biotinidase deficiency
**AP0694**	***BTD***	24797656	F386	P390	/	8	Caucasian	Non-EA	NM_000060.4	c.898A>C	p.Asn300His	c.1330G>C	p.Asp444His	AR	M+M	compound I	Male	Biotinidase deficiency
**AP0695**	***BTD***	24797656	F387	P391	/	9	Caucasian	Non-EA	NM_000060.4	c.1372dup	p.Cys458Leufs*2(	c.968A>G	p.His323Arg	AR	M+T	compound I	Female	Biotinidase deficiency
**AP0696**	***BTD***	24797656	F388	P392	/	10	Caucasian	Non-EA	NM_000060.4	c.310-15delT	p.?	c.626G>A	p.R209H	AR	M+M	compound I	Female	Biotinidase deficiency
**AP0697**	***BTD***	24525934	F389	P393	/		Caucasian	Non-EA	NM_000060.4	c.1330G>C	p.Asp444His	c.1207T>G	p.Phe403Val	AR	M+M	compound I	Male	Biotinidase deficiency
**AP0698**	***BTD***	23481307	F390	P394	BD-1	P1	Caucasian	Non-EA	NM_000060.4	c.631delC	p.Arg211Valfs*53	c.631delC	p.Arg211Valfs*	AR	T+T	homo	/	Biotinidase deficiency
**AP0699**	***BTD***	23481307	F390	P395	BD-1	P2	Caucasian	Non-EA	NM_000060.4	c.631delC	p.Arg211Valfs*53	c.631delC	p.Arg211Valfs*	AR	T+T	homo	/	Biotinidase deficiency
**AP0700**	***BTD***	23481307	F391	P396	BD-2	P3	Caucasian	Non-EA	NM_000060.4	c.1557T>G	p.Tyr519*	c.1557T>G	p.Tyr519*	AR	T+T	homo	/	Biotinidase deficiency
**AP0701**	***BTD***	23481307	F392	P397	BD-3	P4	Caucasian	Non-EA	NM_000060.4	c.321_24insTA	p.Ile108*	c.321_24insTA	p.Ile108*	AR	T+T	homo	/	Biotinidase deficiency
**AP0702**	***BTD***	23481307	F393	P398	BD-4	P5	Caucasian	Non-EA	NM_000060.4	c.631delC	p.Arg211Valfs*53	c.631delC	p.Arg211Valfs*	AR	T+T	homo	/	Biotinidase deficiency
**AP0703**	***BTD***	22698809	F394	P399	/	1	Caucasian	Non-EA	NM_000060.4	c.810T>C	p.=	c.811G>T	p.Ala271Ser	AR	M+T	compound I	Male	Biotinidase deficiency
**AP0704**	***BTD***	22698809	F395	P400	/	2	Caucasian	Non-EA	NM_000060.4	c.528G>T	p.Lys176Asn	c.528G>T	p.Lys176Asn	AR	M+M	homo	Male	Biotinidase deficiency
**AP0705**	***BTD***	22698809	F396	P401	/	3	Caucasian	Non-EA	NM_000060.4	c.528G>T	p.Lys176Asn	c.1368A>C	p.Gln456His	AR	M+M	compound I	Male	Biotinidase deficiency
**AP0706**	***BTD***	22698809	F397	P402	/	4	Caucasian	Non-EA	NM_000060.4	c.1368A>C	p.Gln456His	c.1629C>A	p.Asp543Glu	AR	M+M	compound I	Male	Biotinidase deficiency
**AP0707**	***BTD***	22698809	F398	P403	/	5	Caucasian	Non-EA	NM_000060.4	c.528G>T	p.Lys176Asn	c.528G>T	p.Lys176Asn	AR	M+M	homo	Male	Biotinidase deficiency
**AP0708**	***BTD***	22698809	F399	P404	/	6	Caucasian	Non-EA	NM_000060.4	c.504C>A	p.Phe168Leu	c.528G>T	p.Lys176Asn	AR	M+M	compound I	Female	Biotinidase deficiency
**AP0709**	***BTD***	22698809	F400	P405	/	7	Caucasian	Non-EA	NM_000060.4	c.528G>T	p.Lys176Asn	c.866C>T	p.Ala289Val	AR	M+M	compound I	Male	Biotinidase deficiency
**AP0710**	***BTD***	22698809	F401	P406	/	8	Caucasian	Non-EA	NM_000060.4	c.528G>T	p.Lys176Asn	c.528G>T	p.Lys176Asn	AR	M+M	homo	Female	Biotinidase deficiency
**AP0711**	***BTD***	22698809	F402	P407	/	9	Caucasian	Non-EA	NM_000060.4	c.528G>T	p.Lys176Asn	c.1207T>G	p.Phe403Val	AR	M+M	compound I	Male	Biotinidase deficiency
**AP0712**	***BTD***	22698809	F403	P408	/	10	Caucasian	Non-EA	NM_000060.4	c.814T>G	p.Trp272Gly	c.933delT	p.Ser311Argfs	AR	M+T	compound I	Female	Biotinidase deficiency
**AP0713**	***BTD***	22698809	F404	P409	/	11	Caucasian	Non-EA	NM_000060.4	c.528G>T	p.Lys176Asn	c.743T>C	p.Ile248Thr	AR	M+M	compound I	Male	Biotinidase deficiency
**AP0714**	***BTD***	22698809	F405	P410	/	12	Caucasian	Non-EA	NM_000060.4	c.202_205dupATCC	p.Leu69Hisfs*24	c.1595C>T	p.Thr532Met	AR	M+T	compound I	Male	Biotinidase deficiency
**AP0715**	***BTD***	22698809	F406	P411	/	13	Caucasian	Non-EA	NM_000060.4	c.528G>T	p.Lys176Asn	c.528G>T	p.Lys176Asn	AR	M+M	homo	Male	Biotinidase deficiency
**AP0716**	***BTD***	22698809	F407	P412	/	14	Caucasian	Non-EA	NM_000060.4	c.755A>G	p.Asp252Gly	c.511G>	p.Ala171Thr	AR	M+M	compound I	Female	Biotinidase deficiency
**AP0717**	***BTD***	22698809	F408	P413	/	15	Caucasian	Non-EA	NM_000060.4	c.1629C>A	p.Asp543Glu	c.587delC	p.Thr196Lysfs	AR	M+T	compound I	Male	Biotinidase deficiency
**AP0718**	***BTD***	22698809	F409	P414	/	24	Caucasian	Non-EA	NM_000060.4	c.184G>A	p.Val62Met	c.946C>A	p.Pro316Thr	AR	M+M	compound I	Female	Biotinidase deficiency
**AP0719**	***BTD***	22698809	F410	P415	/	16	Caucasian	Non-EA	NM_000060.4	c.1612C>A	p.Arg538Ser	/	/	AD	M	heter	Female	Biotinidase deficiency
**AP0720**	***BTD***	22698809	F411	P416	/	17	Caucasian	Non-EA	NM_000060.4	c.665A>G	p.Asp222Gly	c.1368A>C	p.Gln456His	AR	M+M	compound I	Female	Biotinidase deficiency
**AP0721**	***BTD***	22698809	F412	P417	/	19	Caucasian	Non-EA	NM_000060.4	c.470G>A	p.Arg157His	c.1010del	p.Val337Glyfs*	AR	M+T	compound I	Male	Biotinidase deficiency
**AP0722**	***BTD***	22698809	F413	P418	/	20	Caucasian	Non-EA	NM_000060.4	c.357_366delCTTTAC	p.Asn119Lysfs*37	c.470G>A	p.Arg157His	AR	M+T	compound I	Female	Biotinidase deficiency
**AP0723**	***BTD***	22698809	F414	P419	/	21	Caucasian	Non-EA	NM_000060.4	c.98_104delinsTCC	p.Cys33Phefs*36	c.470G>A	p.Arg157His	AR	M+T	compound I	Female	Biotinidase deficiency
**AP0724**	***BTD***	22698809	F415	P420	/	22	Caucasian	Non-EA	NM_000060.4	c.1612C>T	p.Arg538Cys	c.1612C>T	p.Arg538Cys	AR	M+M	compound I	Male	Biotinidase deficiency
**AP0725**	***BTD***	22698809	F416	P421	/	23	Caucasian	Non-EA	NM_000060.4	c.357_366delCTTTAC	p.Asn119Lysfs*37	c.470G>A	p.Arg157His	AR	M+T	compound I	Male	Biotinidase deficiency
**AP0726**	***BTD***	21907891	F417	P422	/		Caucasian	Non-EA	NM_000060.4	c.1339C>T	p.His447Tyr	c.1339C>T	p.His447Tyr	AR	M+M	homo	Male	Biotinidase deficiency
**AP0727**	***BTD***	21752405	F418	P423	/	1	Caucasian	Non-EA	NM_000060.4	c.1330G>C	p.Asp444His	c.511G>A	p.Ala171Thr	AR	M+M	compound I	/	Biotinidase deficiency
**AP0728**	***BTD***	21752405	F419	P424	/	2	Caucasian	Non-EA	NM_000060.4	c.1330G>C	p.Asp444His	c.511G>A	p.Ala171Thr	AR	M+M	compound I	/	Biotinidase deficiency
**AP0729**	***BTD***	21752405	F420	P425	/	3	Caucasian	Non-EA	NM_000060.4	c.1330G>C	p.Asp444His	c.511G>A	p.Ala171Thr	AR	M+M	compound I	/	Biotinidase deficiency
**AP0730**	***BTD***	21752405	F421	P426	/	4	Caucasian	Non-EA	NM_000060.4	c.631C>T	p.Arg211Cys	c.631C>T	p.Arg211Cys	AR	M+M	homo	/	Biotinidase deficiency
**AP0731**	***BTD***	21752405	F422	P427	/	6	Caucasian	Non-EA	NM_000060.4	c.1330G>C	p.Asp444His	c.511G>A	p.Ala171Thr	AR	M+M	compound I	/	Biotinidase deficiency
**AP0732**	***BTD***	21752405	F423	P428	/	7	Caucasian	Non-EA	NM_000060.4	c.1330G>C	p.Asp444His	c.511G>A	p.Ala171Thr	AR	M+M	compound I	/	Biotinidase deficiency
**AP0733**	***BTD***	21752405	F424	P429	/	8	Caucasian	Non-EA	NM_000060.4	c.1330G>C	p.Asp444His	?	/	AD?	M+?	heter	/	Biotinidase deficiency
**AP0734**	***BTD***	21752405	F425	P430	/	9	Caucasian	Non-EA	NM_000060.4	c.98_104delinsTCC	p.Cys33Phefs*36	?	?	AR?	?	compound I	/	Biotinidase deficiency
**AP0735**	***BTD***	21752405	F426	P431	/	10	Caucasian	Non-EA	NM_000060.4	c.1330G>C	p.Asp444His	c.1330G>C	p.Asp444His	AR	M+M	homo	/	Biotinidase deficiency
**AP0736**	***BTD***	21752405	F427	P432	/	11	Caucasian	Non-EA	NM_000060.4	c.1330G>C	p.Asp444His	c.1330G>C	p.Asp444His	AR	M+M	homo	/	Biotinidase deficiency
**AP0737**	***BTD***	21752405	F428	P433	/	12	Caucasian	Non-EA	NM_000060.4	c.1330G>C	p.Asp444His	c.1330G>C	p.Asp444His	AR	M+M	homo	/	Biotinidase deficiency
**AP0738**	***BTD***	21752405	F429	P434	/	13	Caucasian	Non-EA	NM_000060.4	c.1330G>C	p.Asp444His	c.1330G>C	p.Asp444His	AR	M+M	homo	/	Biotinidase deficiency
**AP0739**	***BTD***	21752405	F430	P435	/	14	Caucasian	Non-EA	NM_000060.4	c.1330G>C	p.Asp444His	c.1330G>C	p.Asp444His	AR	M+M	homo	/	Biotinidase deficiency
**AP0740**	***BTD***	21752405	F431	P436	/	15	Caucasian	Non-EA	NM_000060.4	c.1330G>C	p.Asp444His	c.1330G>C	p.Asp444His	AR	M+M	homo	/	Biotinidase deficiency
**AP0741**	***BTD***	21752405	F432	P437	/	16	Caucasian	Non-EA	NM_000060.4	c.1330G>C	p.Asp444His	c.1330G>C	p.Asp444His	AR	M+M	homo	/	Biotinidase deficiency
**AP0742**	***BTD***	21752405	F433	P438	/	17	Caucasian	Non-EA	NM_000060.4	c.1330G>C	p.Asp444His	c.1330G>C	p.Asp444His	AR	M+M	homo	/	Biotinidase deficiency
**AP0743**	***BTD***	21752405	F434	P439	/	18	Caucasian	Non-EA	NM_000060.4	c.1211C>T	p.Thr404Ile	/	/	AD	M	heter	/	Biotinidase deficiency
**AP0744**	***BTD***	21752405	F435	P440	/	19	Caucasian	Non-EA	NM_000060.4	c.98_104delinsTCC	p.Cys33Phefs*36	?	/	AD?	T+?	heter	/	Biotinidase deficiency
**AP0745**	***BTD***	21752405	F436	P441	/	20	Caucasian	Non-EA	NM_000060.4	c.100G>A	p.Gly34Ser	c.1394dupG	p.Leu466Serfs	AR	M+T	compound I	/	Biotinidase deficiency
**AP0746**	***BTD***	21752405	F437	P442	/	21	Caucasian	Non-EA	NM_000060.4	c.1595C>T	p.Thr532Met	c.1595C>T	p.Thr532Met	AR	M+M	homo	/	Biotinidase deficiency
**AP0747**	***BTD***	20224900	F438	P443	/	P01	Caucasian	Non-EA	NM_000060.4	c.1489C > T	p.Pro497Ser	c.248T>C	p.Leu83Ser	AR	M+M	compound I	/	Biotinidase deficiency
**AP0748**	***BTD***	20224900	F439	P444	/	P02	Caucasian	Non-EA	NM_000060.4	c.1595C>T	p.Thr532Met	c.1595C>T	p.Thr532Met	AR	M+M	homo	/	Biotinidase deficiency
**AP0749**	***BTD***	20224900	F440	P445	/	P04	Caucasian	Non-EA	NM_000060.4	c.1333G>A	p.Gly445Arg	c.1333G>A	p.Gly445Arg	AR	M+M	homo	/	Biotinidase deficiency
**AP0750**	***BTD***	20224900	F441	P446	/	P06	Caucasian	Non-EA	NM_000060.4	c.511G>A	p.Ala171Thr	c.764T >C	p.Ile255Thr	AR	M+M	compound I	/	Biotinidase deficiency
**AP0751**	***BTD***	20224900	F442	P447	/	P11	Caucasian	Non-EA	NM_000060.4	c.1595C>T	p.Thr532Met	c.1595C>T	p.Thr532Met	AR	M+M	homo	/	Biotinidase deficiency
**AP0752**	***BTD***	20224900	F443	P448	/	P13	Caucasian	Non-EA	NM_000060.4	c.1333G>A	p.Gly445Arg	c.470G>A	p.Arg157His	AR	M+M	compound I	/	Biotinidase deficiency
**AP0753**	***BTD***	20224900	F444	P449	/	P05	Caucasian	Non-EA	NM_000060.4	c.1330G>C	p.Asp444His	c.1595C>T	p.Thr532Met	AR	M+M	compound I	/	Biotinidase deficiency
**AP0754**	***BTD***	20224900	F445	P450	/	P07	Caucasian	Non-EA	NM_000060.4	c.605A>T	p.Asn202Ile	c.1489C>T	p.Pro497Ser	AR	M+M	compound I	/	Biotinidase deficiency
**AP0755**	***BTD***	20224900	F446	P451	/	P08	Caucasian	Non-EA	NM_000060.4	c.1330G>C	p.Asp444His	c.235C>T	p.Arg79Cys	AR	M+M	compound I	/	Biotinidase deficiency
**AP0756**	***BTD***	20224900	F447	P452	/	P09	Caucasian	Non-EA	NM_000060.4	c.1368A>C	p.Gln456His	c.1330G>C	p.Asp444His	AR	M+M	compound I	/	Biotinidase deficiency
**AP0757**	***BTD***	20224900	F448	P453	/	P10	Caucasian	Non-EA	NM_000060.4	c.1330G>C	p.Asp444His	c.1214T>C	p.Leu405Pro	AR	M+M	compound I	/	Biotinidase deficiency
**AP0758**	***BTD***	20224900	F449	P454	/	P12-1	Caucasian	Non-EA	NM_000060.4	c.469C>T	p.Arg157Cys	c.469C>T	p.Arg157Cys	AR	M+M	homo	/	Biotinidase deficiency
**AP0759**	***BTD***	20224900	F449	P455	/	P12-2	Caucasian	Non-EA	NM_000060.4	c.469C>T	p.Arg157Cys	c.469C>T	p.Arg157Cys	AR	M+M	homo	/	Biotinidase deficiency
**AP0760**	***BTD***	20083419	F450	P456	/	P1	Caucasian	Non-EA	NM_000060.4	c.1052delC	p.Thr351Lysfs*12	c.1052delC	p.Thr351Lysfs	AR	T+T	homo	/	Biotinidase deficiency
**AP0761**	***BTD***	20083419	F451	P457	/	P2	Caucasian	Non-EA	NM_000060.4	c.98_104delinsTCC	p.Cys33Phefs*36	c.98_104delin	p.Cys33Phefs*	AR	T+T	homo	/	Biotinidase deficiency
**AP0762**	***BTD***	20083419	F452	P458	/	P3	Caucasian	Non-EA	NM_000060.4	c.100G>A	p.Gly34Ser	c.341G>T	p.Gly114Val	AR	M+M	compound I	/	Biotinidase deficiency
**AP0763**	***BTD***	20083419	F453	P459	/	P4	Caucasian	Non-EA	NM_000060.4	c.98_104delinsTCC	p.Cys33Phefs*36	c.98_104delin	p.Cys33Phefs*	AR	T+T	homo	/	Biotinidase deficiency
**AP0764**	***BTD***	20083419	F454	P460	/	P5	Caucasian	Non-EA	NM_000060.4	c.794A>T	p.His265Leu	c.933T>G	p.Ser311Arg	AR	M+M	compound I	/	Biotinidase deficiency
**AP0765**	***BTD***	20083419	F455	P461	/	P6	Caucasian	Non-EA	NM_000060.4	c.1052delC	p.Thr351Lysfs*12	c.1052delC	p.Thr351Lysfs	AR	T+T	homo	/	Biotinidase deficiency
**AP0766**	***BTD***	20083419	F456	P462	/	P7	Caucasian	Non-EA	NM_000060.4	c.98_104delinsTCC	p.Cys33Phefs*36	c.1612C>T	p.Arg538Cys	AR	M+T	compound I	/	Biotinidase deficiency
**AP0767**	***BTD***	20083419	F457	P463	/	P8	Caucasian	Non-EA	NM_000060.4	c.1264_1265insC	p.Leu422Profs*23	c.1264dup	p.Leu422Profs	AR	T+T	compound I	/	Biotinidase deficiency
**AP0768**	***BTD***	20083419	F458	P464	/	P9	Caucasian	Non-EA	NM_000060.4	c.98_104delinsTCC	p.Cys33Phefs*36	c.98_104delin	p.Cys33Phefs*	AR	T+T	homo	/	Biotinidase deficiency
**AP0769**	***BTD***	20083419	F459	P465	/	P10	Caucasian	Non-EA	NM_000060.4	c.98_104delinsTCC	p.Cys33Phefs*36	c.98_104delin	p.Cys33Phefs*	AR	T+T	homo	/	Biotinidase deficiency
**AP0770**	***BTD***	20083419	F460	P466	/	P11	Caucasian	Non-EA	NM_000060.4	c.98_104delinsTCC	p.Cys33Phefs*36	c.98_104delin	p.Cys33Phefs*	AR	T+T	homo	/	Biotinidase deficiency
**AP0771**	***BTD***	20083419	F461	P467	/	P12	Caucasian	Non-EA	NM_000060.4	c.559C>T	p.Pro187Ser	c.559C>T	p.Pro187Ser	AR	M+M	homo	/	Biotinidase deficiency
**AP0772**	***BTD***	20083419	F462	P468	/	P13	Caucasian	Non-EA	NM_000060.4	c.98_104delinsTCC	p.Cys33Phefs*36	c.98_104delin	p.Cys33Phefs*	AR	T+T	homo	/	Biotinidase deficiency
**AP0773**	***BTD***	20083419	F463	P469	/	P14	Caucasian	Non-EA	NM_000060.4	c.1275T>G	p.Tyr425*	c.1275T>G	p.Tyr425*	AR	T+T	homo	/	Biotinidase deficiency
**AP0774**	***BTD***	20083419	F464	P470	/	P15	Caucasian	Non-EA	NM_000060.4	c.1275T>G	p.Tyr425*	c.1275T>G	p.Tyr425*	AR	T+T	homo	/	Biotinidase deficiency
**AP0775**	***BTD***	20083419	F465	P471	/	P16	Caucasian	Non-EA	NM_000060.4	c.1275T>G	p.Tyr425*	c.1275T>G	p.Tyr425*	AR	T+T	homo	/	Biotinidase deficiency
**AP0776**	***BTD***	20083419	F466	P472	/	P17	Caucasian	Non-EA	NM_000060.4	c.1275T>G	p.Tyr425*	c.1275T>G	p.Tyr425*	AR	T+T	homo	/	Biotinidase deficiency
**AP0777**	***BTD***	20083419	F467	P473	/	P18	Caucasian	Non-EA	NM_000060.4	c.1275T>G	p.Tyr425*	c.1275T>G	p.Tyr425*	AR	T+T	homo	/	Biotinidase deficiency
**AP0778**	***BTD***	20083419	F468	P474	/	P19	Caucasian	Non-EA	NM_000060.4	c.1275T>G	p.Tyr425*	c.1275T>G	p.Tyr425*	AR	T+T	homo	/	Biotinidase deficiency
**AP0779**	***BTD***	20083419	F469	P475	/	P20	Caucasian	Non-EA	NM_000060.4	c.1275T>G	p.Tyr425*	c.1275T>G	p.Tyr425*	AR	T+T	homo	/	Biotinidase deficiency
**AP0780**	***BTD***	20083419	F470	P476	/	P21	Caucasian	Non-EA	NM_000060.4	c.1275T>G	p.Tyr425*	c.1275T>G	p.Tyr425*	AR	T+T	homo	/	Biotinidase deficiency
**AP0781**	***BTD***	20083419	F471	P477	/	P22	Caucasian	Non-EA	NM_000060.4	c.212T>C	p.Leu71Pro	c.98_104delin	p.Cys33Phefs*	AR	M+T	compound I	/	Biotinidase deficiency
**AP0782**	***BTD***	20083419	F472	P478	/	P23	Caucasian	Non-EA	NM_000060.4	c.98_104delinsTCC	p.Cys33Phefs*36	c.98_104delin	p.Cys33Phefs*	AR	T+T	homo	/	Biotinidase deficiency
**AP0783**	***BTD***	19757147	F473	P479	/	1	Caucasian	Non-EA	NM_000060.4	c.424C>A	p.Pro142Thr	c.1489C>T	p.Pro497Ser	AR	M+M	compound I	/	Biotinidase deficiency
**AP0784**	***BTD***	19757147	F474	P480	/	2	Caucasian	Non-EA	NM_000060.4	c.424C>A	p.Pro142Thr	c.1489C>T	p.Pro497Ser	AR	M+M	compound I	/	Biotinidase deficiency
**AP0785**	***BTD***	19757147	F475	P481	/	3	Caucasian	Non-EA	NM_000060.4	c.1489C>T	p.Pro497Ser	c.1489C>T	p.Pro497Ser	AR	M+M	homo	/	Biotinidase deficiency
**AP0786**	***BTD***	19757147	F476	P482	/	4	Caucasian	Non-EA	NM_000060.4	c.424C>A	p.Pro142Thr	c.1489C>T	p.Pro497Ser	AR	M+M	compound I	/	Biotinidase deficiency
**AP0787**	***BTD***	19757147	F477	P483	/	5	Caucasian	Non-EA	NM_000060.4	c.1432G>C	p.Ala478Pro	c.1432G>C	p.Ala478Pro	AR	M+M	homo	/	Biotinidase deficiency
**AP0788**	***BTD***	18645204	F478	P484	/		Caucasian	Non-EA	NM_000060.4	c.1339C>T	p.His447Tyr	c.1339C>T	p.His447Tyr	AR	M+M	homo	/	Biotinidase deficiency
**AP0789**	***BTD***	17382128	F479	P485	/	16	Caucasian	Non-EA	NM_000060.4	c.98_104delinsTCC	p.Cys33Phefs*36	c.98_104delin	p.Cys33Phefs*	AR	T+T	homo	Female	Biotinidase deficiency
**AP0790**	***BTD***	17382128	F480	P486	/	18	Caucasian	Non-EA	NM_000060.4	c.171T>G	p.Tyr57*	c.171T>G	p.Tyr57*	AR	T+T	homo	Male	Biotinidase deficiency
**AP0791**	***BTD***	17382128	F481	P487	/	17	Caucasian	Non-EA	NM_000060.4	c.171T>G	p.Tyr57*	c.171T>G	p.Tyr57*	AR	T+T	homo	Male	Biotinidase deficiency
**AP0792**	***BTD***	17382128	F482	P488	/	19	Caucasian	Non-EA	NM_000060.4	c.171T>G	p.Tyr57*	c.171T>G	p.Tyr57*	AR	T+T	homo	Male	Biotinidase deficiency
**AP0793**	***BTD***	17382128	F483	P489	/	20	Caucasian	Non-EA	NM_000060.4	c.1612C>T	p.Arg538Cys	c.1612C>T	p.Arg538Cys	AR	M+M	compound I	Female	Biotinidase deficiency
**AP0794**	***BTD***	17382128	F484	P490	/	15	Caucasian	Non-EA	NM_000060.4	c.98_104delinsTCC	p.Cys33Phefs*36	c.98_104delin	p.Cys33Phefs*	AR	T+T	homo	Female	Biotinidase deficiency
**AP0795**	***BTD***	17382128	F485	P491	/	13	Caucasian	Non-EA	NM_000060.4	c.171T>G	p.Tyr57*	c.171T>G	p.Tyr57*	AR	T+T	homo	Female	Biotinidase deficiency
**AP0796**	***BTD***	17382128	F486	P492	/	12	Caucasian	Non-EA	NM_000060.4	c.98_104delinsTCC	p.Cys33Phefs*36	c.98_104delin	p.Cys33Phefs*	AR	T+T	homo	Female	Biotinidase deficiency
**AP0797**	***BTD***	17382128	F487	P493	/	11	Caucasian	Non-EA	NM_000060.4	c.98_104delinsTCC	p.Cys33Phefs*36	c.98_104delin	p.Cys33Phefs*	AR	T+T	homo	Female	Biotinidase deficiency
**AP0798**	***BTD***	17382128	F488	P494	/	10	Caucasian	Non-EA	NM_000060.4	c.1493insT	p.Leu498Phefs*1	c.1493insT	p.Leu498Phef	AR	T+T	homo	Male	Biotinidase deficiency
**AP0799**	***BTD***	17382128	F489	P495	/	6	Caucasian	Non-EA	NM_000060.4	c.235C>T	p.Arg79Cys	c.235C>T	p.Arg79Cys	AR	M+M	homo	Male	Biotinidase deficiency
**AP0800**	***BTD***	17382128	F490	P496	/	4	Caucasian	Non-EA	NM_000060.4	c.235C>T	p.Arg79Cys	c.235C>T	p.Arg79Cys	AR	M+M	homo	Male	Biotinidase deficiency
**AP0801**	***BTD***	17382128	F491	P497	/	9	Caucasian	Non-EA	NM_000060.4	c.643C>T	p.Leu215Phe	c.643C>T	p.Leu215Phe	AR	M+M	homo	Female	Biotinidase deficiency
**AP0802**	***BTD***	17382128	F492	P498	/	7	Caucasian	Non-EA	NM_000060.4	c.235C>T	p.Arg79Cys	c.235C>T	p.Arg79Cys	AR	M+M	homo	Male	Biotinidase deficiency
**AP0803**	***BTD***	17382128	F493	P499	/	8	Caucasian	Non-EA	NM_000060.4	c.587C>G	p.Thr196Arg	c.587C>G	p.Thr196Arg	AR	M+M	homo	Male	Biotinidase deficiency
**AP0804**	***BTD***	17382128	F494	P500	/	5	Caucasian	Non-EA	NM_000060.4	c.235C>T	p.Arg79Cys	c.235C>T	p.Arg79Cys	AR	M+M	homo	Female	Biotinidase deficiency
**AP0805**	***BTD***	17382128	F495	P501	/	1	Caucasian	Non-EA	NM_000060.4	c.98_104delinsTCC	p.Cys33Phefs*36	c.98_104delin	p.Cys33Phefs*	AR	T+T	homo	Female	Biotinidase deficiency
**AP0806**	***BTD***	17382128	F496	P502	/	2	Caucasian	Non-EA	NM_000060.4	c.98_104delinsTCC	p.Cys33Phefs*36	c.98_104delin	p.Cys33Phefs*	AR	T+T	homo	Female	Biotinidase deficiency
**AP0807**	***BTD***	17382128	F497	P503	/	3	Caucasian	Non-EA	NM_000060.4	c.1493insT	p.Leu498Phefs*1	c.1493insT	p.Leu498Phef	AR	T+T	homo	Male	Biotinidase deficiency
**AP0808**	***BTD***	17185019	F498	P504	/	05-01	Caucasian	Non-EA	NM_000060.4	c.1368A>C	p.Gln456His	c.1368A>C	p.Gln456His	AR	M+M	compound I	Male	Biotinidase deficiency
**AP0809**	***BTD***	17185019	F499	P505	/	06-01	Caucasian	Non-EA	NM_000060.4	c.652G>C	p.Glu218Gln	c.832C>G	p.Leu278Val	AR	M+M	compound I	/	Biotinidase deficiency
**AP0810**	***BTD***	17185019	F500	P506	/	07-01	Caucasian	Non-EA	NM_000060.4	c.334G>A	p.Glu112Lys	c.1368A>C	p.Gln456His	AR	M+M	compound I	/	Biotinidase deficiency
**AP0811**	***BTD***	17185019	F501	P507	/	19-01	Caucasian	Non-EA	NM_000060.4	c.933delT	p.Ser311Argfs*23	c.1368A>C	p.Gln456His	AR	M+T	compound I	/	Biotinidase deficiency
**AP0812**	***BTD***	17185019	F502	P508	/	28-01	Caucasian	Non-EA	NM_000060.4	c.334G>A	p.Glu112Lys	c.1368A>C	p.Gln456His	AR	M+M	compound I	/	Biotinidase deficiency
**AP0813**	***BTD***	17185019	F502	P509	/	28-02	Caucasian	Non-EA	NM_000060.4	c.334G>A	p.Glu112Lys	c.1612C>T	p.Arg538Cys	AR	M+M	compound I	/	Biotinidase deficiency
**AP0814**	***BTD***	17185019	F503	P510	/	30-01	Caucasian	Non-EA	NM_000060.4	c.1368A>C	p.Gln456His	c.1612C>T	p.Arg538Cys	AR	M+M	compound I	/	Biotinidase deficiency
**AP0815**	***BTD***	17185019	F504	P511	/	36-01	Caucasian	Non-EA	NM_000060.4	c.98_104delinsTCC	p.Cys33Phefs*36	c.212T>C	p.Leu71Pro	AR	M+T	compound I	/	Biotinidase deficiency
**AP0816**	***BTD***	17185019	F505	P512	/	37-01	Caucasian	Non-EA	NM_000060.4	c.184G>A	p.Val62Met	c.310-1G>T	p.?	AR	M+T	compound I	/	Biotinidase deficiency
**AP0817**	***BTD***	17185019	F506	P513	/	38-01	Caucasian	Non-EA	NM_000060.4	c.245C>A	p.Ala82Val	/	/	AD	M	heter	/	Biotinidase deficiency
**AP0818**	***BTD***	17185019	F507	P514	/	39-01	Caucasian	Non-EA	NM_000060.4	c.595G>A	p.Val199Met	/	/	AD	M	heter	/	Biotinidase deficiency
**AP0819**	***BTD***	17185019	F508	P515	/	01-02	Caucasian	Non-EA	NM_000060.4	c.[511G>A; 1330G>C]	p.Ala171Thr+p.As	c.1330G>C	p.Asp444His	AR	M+M	compound I	/	Biotinidase deficiency
**AP0820**	***BTD***	17185019	F508	P516	/	01-03	Caucasian	Non-EA	NM_000060.4	c.[511G>A; 1330G>C]	p.Ala171Thr+p.As	c.1330G>C	p.Asp444His	AR	M+M	compound I	/	Biotinidase deficiency
**AP0821**	***BTD***	17185019	F509	P517	/	10-01	Caucasian	Non-EA	NM_000060.4	c.[511G>A; 1330G>C]	p.Ala171Thr+p.As	c.1330G>C	p.Asp444His	AR	M+M	compound I	/	Biotinidase deficiency
**AP0822**	***BTD***	17185019	F510	P518	/	24-01	Caucasian	Non-EA	NM_000060.4	c.[511G>A; 1330G>C]	p.Ala171Thr+p.As	c.1330G>C	p.Asp444His	AR	M+M	compound I	/	Biotinidase deficiency
**AP0823**	***BTD***	17185019	F511	P519	/	27-01	Caucasian	Non-EA	NM_000060.4	c.[511G>A; 1330G>C]	p.Ala171Thr+p.As	c.1330G>C	p.Asp444His	AR	M+M	compound I	/	Biotinidase deficiency
**AP0824**	***BTD***	17185019	F512	P520	/	35-01	Caucasian	Non-EA	NM_000060.4	c.[511G>A; 1330G>C]	p.Ala171Thr+p.As	c.1330G>C	p.Asp444His	AR	M+M	compound I	/	Biotinidase deficiency
**AP0825**	***BTD***	17185019	F513	P521	/	44-01	Caucasian	Non-EA	NM_000060.4	c.[511G>A; 1330G>C]	p.Ala171Thr+p.As	c.1330G>C	p.Asp444His	AR	M+M	compound I	/	Biotinidase deficiency
**AP0826**	***BTD***	17185019	F514	P522	/	02-01	Caucasian	Non-EA	NM_000060.4	c.98_104delinsTCC	p.Cys33Phefs*36	c.1330G>C	p.Asp444His	AR	M+T	compound I	/	Biotinidase deficiency
**AP0827**	***BTD***	17185019	F515	P523	/	11-01	Caucasian	Non-EA	NM_000060.4	c.98_104delinsTCC	p.Cys33Phefs*36	c.1330G>C	p.Asp444His	AR	M+T	compound I	/	Biotinidase deficiency
**AP0828**	***BTD***	17185019	F516	P524	/	14-01	Caucasian	Non-EA	NM_000060.4	c.98_104delinsTCC	p.Cys33Phefs*36	c.1330G>C	p.Asp444His	AR	M+T	compound I	/	Biotinidase deficiency
**AP0829**	***BTD***	17185019	F517	P525	/	17-01	Caucasian	Non-EA	NM_000060.4	c.98_104delinsTCC	p.Cys33Phefs*36	c.1330G>C	p.Asp444His	AR	M+T	compound I	/	Biotinidase deficiency
**AP0830**	***BTD***	17185019	F517	P526	/	17-02	Caucasian	Non-EA	NM_000060.4	c.98_104delinsTCC	p.Cys33Phefs*36	c.1330G>C	p.Asp444His	AR	M+T	compound I	/	Biotinidase deficiency
**AP0831**	***BTD***	17185019	F518	P527	/	22-01	Caucasian	Non-EA	NM_000060.4	c.98_104delinsTCC	p.Cys33Phefs*36	c.1330G>C	p.Asp444His	AR	M+T	compound I	/	Biotinidase deficiency
**AP0832**	***BTD***	17185019	F519	P528	/	25-01	Caucasian	Non-EA	NM_000060.4	c.98_104delinsTCC	p.Cys33Phefs*36	c.1330G>C	p.Asp444His	AR	M+T	compound I	/	Biotinidase deficiency
**AP0833**	***BTD***	17185019	F520	P529	/	33-01	Caucasian	Non-EA	NM_000060.4	c.98_104delinsTCC	p.Cys33Phefs*36	c.1330G>C	p.Asp444His	AR	M+T	compound I	/	Biotinidase deficiency
**AP0834**	***BTD***	17185019	F521	P530	/	03-01	Caucasian	Non-EA	NM_000060.4	c.454A>C	p.Thr152Pro	c.1330G>C	p.Asp444His	AR	M+M	compound I	/	Biotinidase deficiency
**AP0835**	***BTD***	17185019	F522	P531	/	04-01	Caucasian	Non-EA	NM_000060.4	c.1368A>C	p.Gln456His	c.1330G>C	p.Asp444His	AR	M+M	compound I	/	Biotinidase deficiency
**AP0836**	***BTD***	17185019	F523	P532	/	09-01	Caucasian	Non-EA	NM_000060.4	c.1368A>C	p.Gln456His	c.1330G>C	p.Asp444His	AR	M+M	compound I	/	Biotinidase deficiency
**AP0837**	***BTD***	17185019	F524	P533	/	09-02	Caucasian	Non-EA	NM_000060.4	c.1368A>C	p.Gln456His	c.1330G>C	p.Asp444His	AR	M+M	compound I	/	Biotinidase deficiency
**AP0838**	***BTD***	17185019	F525	P534	/	13-01	Caucasian	Non-EA	NM_000060.4	c.1368A>C	p.Gln456His	c.1330G>C	p.Asp444His	AR	M+M	compound I	/	Biotinidase deficiency
**AP0839**	***BTD***	17185019	F525	P535	/	13-02	Caucasian	Non-EA	NM_000060.4	c.1368A>C	p.Gln456His	c.1330G>C	p.Asp444His	AR	M+M	compound I	/	Biotinidase deficiency
**AP0840**	***BTD***	17185019	F526	P536	/	15-01	Caucasian	Non-EA	NM_000060.4	c.1368A>C	p.Gln456His	c.1330G>C	p.Asp444His	AR	M+M	compound I	/	Biotinidase deficiency
**AP0841**	***BTD***	17185019	F527	P537	/	26-01	Caucasian	Non-EA	NM_000060.4	c.1368A>C	p.Gln456His	c.1330G>C	p.Asp444His	AR	M+M	compound I	/	Biotinidase deficiency
**AP0842**	***BTD***	17185019	F528	P538	/	31-01	Caucasian	Non-EA	NM_000060.4	c.1368A>C	p.Gln456His	c.1330G>C	p.Asp444His	AR	M+M	compound I	/	Biotinidase deficiency
**AP0843**	***BTD***	17185019	F529	P539	/	45-01	Caucasian	Non-EA	NM_000060.4	c.1368A>C	p.Gln456His	c.1330G>C	p.Asp444His	AR	M+M	compound I	/	Biotinidase deficiency
**AP0844**	***BTD***	17185019	F530	P540	/	12-01	Caucasian	Non-EA	NM_000060.4	c.1253G>C	p.Cys418Ser	c.1330G>C	p.Asp444His	AR	M+M	compound I	/	Biotinidase deficiency
**AP0845**	***BTD***	17185019	F531	P541	/	16-01	Caucasian	Non-EA	NM_000060.4	c.136G>T	p.Glu46*	c.1330G>C	p.Asp444His	AR	M+T	compound I	/	Biotinidase deficiency
**AP0846**	***BTD***	17185019	F532	P542	/	20-01	Caucasian	Non-EA	NM_000060.4	c.832C>G	p.Leu278Val	c.1330G>C	p.Asp444His	AR	M+M	compound I	/	Biotinidase deficiency
**AP0847**	***BTD***	17185019	F533	P543	/	41-01	Caucasian	Non-EA	NM_000060.4	c.832C>G	p.Leu278Val	c.1330G>C	p.Asp444His	AR	M+M	compound I	/	Biotinidase deficiency
**AP0848**	***BTD***	17185019	F534	P544	/	21-01	Caucasian	Non-EA	NM_000060.4	c.469C>T	p.Arg157Cys	c.1330G>C	p.Asp444His	AR	M+M	compound I	/	Biotinidase deficiency
**AP0849**	***BTD***	17185019	F534	P545	/	21-03	Caucasian	Non-EA	NM_000060.4	c.469C>T	p.Arg157Cys	c.1330G>C	p.Asp444His	AR	M+M	compound I	/	Biotinidase deficiency
**AP0850**	***BTD***	17185019	F535	P546	/	23-01	Caucasian	Non-EA	NM_000060.4	c.584A>G	p.Asn195Ser	c.1330G>C	p.Asp444His	AR	M+M	compound I	/	Biotinidase deficiency
**AP0851**	***BTD***	17185019	F535	P547	/	23-02	Caucasian	Non-EA	NM_000060.4	c.584A>G	p.Asn195Ser	c.1330G>C	p.Asp444His	AR	M+M	compound I	/	Biotinidase deficiency
**AP0852**	***BTD***	17185019	F536	P548	/	32-01	Caucasian	Non-EA	NM_000060.4	c.643C>T	p.Leu215Phe	c.1330G>C	p.Asp444His	AR	M+M	compound I	/	Biotinidase deficiency
**AP0853**	***BTD***	17185019	F537	P549	/	34-01	Caucasian	Non-EA	NM_000060.4	c.1612C>T	p.Arg538Cys	c.1330G>C	p.Asp444His	AR	M+M	compound I	/	Biotinidase deficiency
**AP0854**	***BTD***	17185019	F537	P550	/	34-02	Caucasian	Non-EA	NM_000060.4	c.1612C>T	p.Arg538Cys	c.1330G>C	p.Asp444His	AR	M+M	compound I	/	Biotinidase deficiency
**AP0855**	***BTD***	17185019	F538	P551	/	42-01	Caucasian	Non-EA	NM_000060.4	c.1612C>T	p.Arg538Cys	c.1330G>C	p.Asp444His	AR	M+M	compound I	/	Biotinidase deficiency
**AP0856**	***BTD***	17185019	F539	P552	/	43-01	Caucasian	Non-EA	NM_000060.4	c.933delT	p.Ser311Argfs*23	c.1330G>C	p.Asp444His	AR	M+T	compound I	/	Biotinidase deficiency
**AP0857**	***BTD***	15060693	F540	P553	/	P724	Caucasian	Non-EA	NM_000060.4	c.98_104delinsTCC	p.Cys33Phefs*36	c.235C>T	p.Arg79Cys	AR	M+T	compound I	/	Biotinidase deficiency
**AP0858**	***BTD***	15060693	F541	P554	/	P739	Caucasian	Non-EA	NM_000060.4	c.755A>G	p.Asp252Gly	c.755A>G	p.Asp252Gly	AR	M+M	homo	/	Biotinidase deficiency
**AP0859**	***BTD***	15060693	F542	P555	/	P835	Caucasian	Non-EA	NM_000060.4	c.595G>A	p.Val199Met	c.1314T>A	p.Tyr438*	AR	M+T	compound I	/	Biotinidase deficiency
**AP0860**	***BTD***	15060693	F543	P556	/	P815	Caucasian	Non-EA	NM_000060.4	c.98_104delinsTCC	p.Cys33Phefs*36	c.1330G>C	p.Asp444His	AR	M+T	compound I	/	Biotinidase deficiency
**AP0861**	***BTD***	15060693	F544	P557	/	P818	Caucasian	Non-EA	NM_000060.4	c.98_104delinsTCC	p.Cys33Phefs*36	c.1330G>C	p.Asp444His	AR	M+T	compound I	/	Biotinidase deficiency
**AP0862**	***BTD***	15060693	F545	P558	/	P850	Caucasian	Non-EA	NM_000060.4	c.98_104delinsTCC	p.Cys33Phefs*36	c.1330G>C	p.Asp444His	AR	M+T	compound I	/	Biotinidase deficiency
**AP0863**	***BTD***	15060693	F546	P559	/	P730	Caucasian	Non-EA	NM_000060.4	c.100G>A	p.Gly34Ser	c.1330G>C	p.Asp444His	AR	M+M	compound I	/	Biotinidase deficiency
**AP0864**	***BTD***	15060693	F547	P560	/	P715	Caucasian	Non-EA	NM_000060.4	c.184G>A	p.Val62Met	c.1330G>C	p.Asp444His	AR	M+M	compound I	/	Biotinidase deficiency
**AP0865**	***BTD***	15060693	F548	P561	/	P853	Caucasian	Non-EA	NM_000060.4	c.595G>A	p.Val199Met	c.1330G>C	p.Asp444His	AR	M+M	compound I	/	Biotinidase deficiency
**AP0866**	***BTD***	15060693	F549	P562	/	P736	Caucasian	Non-EA	NM_000060.4	c.755A>G	p.Asp252Gly	c.1330G>C	p.Asp444His	AR	M+M	compound I	/	Biotinidase deficiency
**AP0867**	***BTD***	15060693	F550	P563	/	P718	Caucasian	Non-EA	NM_000060.4	c.[511G>A; 1330G>C]	p.Ala171Thr + p.A	c.1330G>C	p.Asp444His	AR	M+M	compound I	/	Biotinidase deficiency
**AP0868**	***BTD***	15060693	F551	P564	/	P721	Caucasian	Non-EA	NM_000060.4	c.[511G>A; 1330G>C]	p.Ala171Thr + p.A	c.1330G>C	p.Asp444His	AR	M+M	compound I	/	Biotinidase deficiency
**AP0869**	***BTD***	15060693	F552	P565	/	P733	Caucasian	Non-EA	NM_000060.4	c.[511G>A; 1330G>C]	p.Ala171Thr + p.A	c.1330G>C	p.Asp444His	AR	M+M	compound I	/	Biotinidase deficiency
**AP0870**	***BTD***	15060693	F553	P566	/	P856	Caucasian	Non-EA	NM_000060.4	c.1330G>C	p.Asp444His	c.1330G>C	p.Asp444His	AR	M+M	homo	/	Biotinidase deficiency
**AP0871**	***BTD***	15060693	F554	P567	/	P811	Caucasian	Non-EA	NM_000060.4	c.133G>A	p.Gly45Arg	/	/	AD	M	heter	/	Biotinidase deficiency
**AP0872**	***BTD***	15060693	F555	P568	/	P824	Caucasian	Non-EA	NM_000060.4	c.236G>A	p.Arg79His	/	/	AD	M	heter	/	Biotinidase deficiency
**AP0873**	***BTD***	15060693	F556	P569	/	P821	Caucasian	Non-EA	NM_000060.4	c.1330G>C	p.Asp444His	/	/	AD	M	heter	/	Biotinidase deficiency
**AP0874**	***BTD***	15060693	F557	P570	/	P859	Caucasian	Non-EA	NM_000060.4	c.1330G>C	p.Asp444His	/	/	AD	M	heter	/	Biotinidase deficiency
**AP0875**	***BTD***	14707518	F558	P571	/	22	Caucasian	Non-EA	NM_000060.4	c.1368A>C	p.Gln456His	c.1368A>C	p.Gln456His	AR	M+M	compound I	Female	Biotinidase deficiency
**AP0876**	***BTD***	14707518	F559	P572	/	13	Caucasian	Non-EA	NM_000060.4	c.1595C>T	p.Thr532Met	c.1595C>T	p.Thr532Met	AR	M+M	homo	Male	Biotinidase deficiency
**AP0877**	***BTD***	14707518	F560	P573	/	3	Caucasian	Non-EA	NM_000060.4	c.1595C>T	p.Thr532Met	c.1595C>T	p.Thr532Met	AR	M+M	homo	Male	Biotinidase deficiency
**AP0878**	***BTD***	14707518	F561	P574	/	16	Caucasian	Non-EA	NM_000060.4	c.1368A>C	p.Gln456His	c.1330G>C	p.Asp444His	AR	M+M	compound I	Female	Biotinidase deficiency
**AP0879**	***BTD***	14707518	F562	P575	/	6	Caucasian	Non-EA	NM_000060.4	c.1595C>T	p.Thr532Met	c.1595C>T	p.Thr532Met	AR	M+M	homo	Male	Biotinidase deficiency
**AP0880**	***BTD***	14707518	F563	P576	/	5	Caucasian	Non-EA	NM_000060.4	c.1595C>T	p.Thr532Met	c.1595C>T	p.Thr532Met	AR	M+M	homo	Male	Biotinidase deficiency
**AP0881**	***BTD***	14707518	F564	P577	/	34	Caucasian	Non-EA	NM_000060.4	c.652G>C	p.Glu218Gln	c.832C>G	p.Leu278Val	AR	M+M	compound I	Female	Biotinidase deficiency
**AP0882**	***BTD***	14707518	F565	P578	/	53	Caucasian	Non-EA	NM_000060.4	c.933delT	p.Ser311Argfs*23	c.1368A>C	p.Gln456His	AR	M+T	compound I	Female	Biotinidase deficiency
**AP0883**	***BTD***	14707518	F566	P579	/	41	Caucasian	Non-EA	NM_000060.4	c.98_104delinsTCC	p.Cys33Phefs*36	c.236G>A+c.2	p.R79H+p.L71	AR	M+T	compound I	Male	Biotinidase deficiency
**AP0884**	***BTD***	14707518	F567	P580	/	84	Caucasian	Non-EA	NM_000060.4	c.532C>G	p.Pro178Ala	c.310G>T	p.Asp104Tyr	AR	M+M	compound I	Female	Biotinidase deficiency
**AP0885**	***BTD***	14707518	F568	P581	/	69	Caucasian	Non-EA	NM_000060.4	c.1612C>T	p.Arg538Cys	c.334G>A	p.Glu112Lys	AR	M+M	compound I	Male	Biotinidase deficiency
**AP0886**	***BTD***	14707518	F569	P582	/	14	Caucasian	Non-EA	NM_000060.4	c.1511T>A	p.M50K	/	/	AD	M	heter	Female	Biotinidase deficiency
**AP0887**	***BTD***	14707518	F570	P583	/	35	Caucasian	Non-EA	NM_000060.4	c.245C>A	p.Ala82Val	/	/	AD	M	heter	Male	Biotinidase deficiency
**AP0888**	***BTD***	14707518	F571	P584	/	2	Caucasian	Non-EA	NM_000060.4	c.1595C>T	p.Thr532Met	c.1330G>C	p.Asp444His	AR	M+M	compound I	Female	Biotinidase deficiency
**AP0889**	***BTD***	14707518	F572	P585	/	45	Caucasian	Non-EA	NM_000060.4	c.1358A>C	p.Tyr453Ser	c.1330G>C	p.Asp444His	AR	M+M	compound I	Male	Biotinidase deficiency
**AP0890**	***BTD***	14707518	F573	P586	/	58	Caucasian	Non-EA	NM_000060.4	c.98_104delinsTCC	p.Cys33Phefs*36	c.1330G>C	p.Asp444His	AR	M+T	compound I	Female	Biotinidase deficiency
**AP0891**	***BTD***	14707518	F574	P587	/	57	Caucasian	Non-EA	NM_000060.4	c.1253G>C	p.Cys418Ser	c.1330G>C	p.Asp444His	AR	M+M	compound I	Female	Biotinidase deficiency
**AP0892**	***BTD***	14707518	F575	P588	/	49	Caucasian	Non-EA	NM_000060.4	c.98_104delinsTCC	p.Cys33Phefs*36	c.1330G>C	p.Asp444His	AR	M+T	compound I	Female	Biotinidase deficiency
**AP0893**	***BTD***	14707518	F576	P589	/	65	Caucasian	Non-EA	NM_000060.4	c.511G>A	p.Ala171Thr	c.1330G>C	p.Asp444His	AR	M+M	compound I	Male	Biotinidase deficiency
**AP0894**	***BTD***	14707518	F577	P590	/	63	Caucasian	Non-EA	NM_000060.4	c.98_104delinsTCC	p.Cys33Phefs*36	c.1330G>C	p.Asp444His	AR	M+T	compound I	Female	Biotinidase deficiency
**AP0895**	***BTD***	12359137	F578	P591	/	900	Caucasian	Non-EA	NM_000060.4	c.393delC	p.Phe131Leufs*2	c.393delC	p.Phe131Leuf	AR	T+T	homo	/	Biotinidase deficiency
**AP0896**	***BTD***	12359137	F579	P592	/	901	Caucasian	Non-EA	NM_000060.4	c.393delC	p.Phe131Leufs*2	c.393delC	p.Phe131Leuf	AR	T+T	homo	/	Biotinidase deficiency
**AP0897**	***BTD***	12359137	F580	P593	/	931	Caucasian	Non-EA	NM_000060.4	c.393delC	p.Phe131Leufs*2	c.393delC	p.Phe131Leuf	AR	T+T	homo	/	Biotinidase deficiency
**AP0898**	***BTD***	12359137	F581	P594	/	472	Caucasian	Non-EA	NM_000060.4	c.1049delC	p.Ala350Glufs*13	c.1049delC	p.Ala350Glufs	AR	T+T	homo	/	Biotinidase deficiency
**AP0899**	***BTD***	12359137	F582	P595	/	445	Caucasian	Non-EA	NM_000060.4	c.1052delC	p.Thr351Lysfs*12	c.98_104delin	p.Cys33Phefs*	AR	T+T	compound I	/	Biotinidase deficiency
**AP0900**	***BTD***	12359137	F583	P596	/	687	Caucasian	Non-EA	NM_000060.4	c.1191-1192del	p.Glu397Aspfs*4	c.1330G>C	p.Asp444His	AR	M+T	compound I	/	Biotinidase deficiency
**AP0901**	***BTD***	12359137	F584	P597	/	624	Caucasian	Non-EA	NM_000060.4	c.1239delC	p.Tyr414Ilefs*87	c.1368A>C	p.Gln456His	AR	M+T	compound I	/	Biotinidase deficiency
**AP0902**	***BTD***	12359137	F585	P598	/	634	Caucasian	Non-EA	NM_000060.4	c.1239delC	p.Tyr414Ilefs*87	c.1368A>C	p.456Q>H	AR	M+T	compound I	/	Biotinidase deficiency
**AP0903**	***BTD***	12359137	F586	P599	/	652	Caucasian	Non-EA	NM_000060.4	c.1239delC	p.Tyr414Ilefs*87	c.1330G>C	p.Asp444His	AR	M+T	compound I	/	Biotinidase deficiency
**AP0904**	***BTD***	12359137	F587	P600	/	539	Caucasian	Non-EA	NM_000060.4	c.190G>A	p.Glu64Lys	c.98_104delin	p.Cys33Phefs*	AR	M+T	compound I	/	Biotinidase deficiency
**AP0905**	***BTD***	12359137	F588	P601	/	916	Caucasian	Non-EA	NM_000060.4	c.278A>G	p.Tyr93Cys	c.278A>G	p.Tyr93Cys	AR	M+M	homo	/	Biotinidase deficiency
**AP0906**	***BTD***	12359137	F589	P602	/	912	Caucasian	Non-EA	NM_000060.4	c.298G>A	p.Ala100Thr	c.1330G>C	p.Asp444His	AR	M+M	compound I	/	Biotinidase deficiency
**AP0907**	***BTD***	12359137	F590	P603	/	925	Caucasian	Non-EA	NM_000060.4	c.595G>A	p.Val199Met	c.1595C>T	p.Thr532Met	AR	M+M	compound I	/	Biotinidase deficiency
**AP0908**	***BTD***	12359137	F591	P604	/	853	Caucasian	Non-EA	NM_000060.4	c.595G>A	p.Val199Met	c.1330G>C	p.Asp444His	AR	M+M	compound I	/	Biotinidase deficiency
**AP0909**	***BTD***	12359137	F592	P605	/	530	Caucasian	Non-EA	NM_000060.4	c.794A>T	p.His265Leu	c.133G>A	p.Gly45Arg	AR	M+M	compound I	/	Biotinidase deficiency
**AP0910**	***BTD***	12359137	F593	P606	/	485	Caucasian	Non-EA	NM_000060.4	c.887T>G	p.Val296Gly	c.933delT	p.Ser311Argfs	AR	M+T	compound I	/	Biotinidase deficiency
**AP0911**	***BTD***	12359137	F594	P607	/	432	Caucasian	Non-EA	NM_000060.4	c.934G>A	p.Gly312Ser	c.933delT	p.Ser311Argfs	AR	M+T	compound I	/	Biotinidase deficiency
**AP0912**	***BTD***	12359137	F595	P608	/	421	Caucasian	Non-EA	NM_000060.4	c.1313A>G	p.Tyr438Cys	c.98_104delin	p.Cys33Phefs*	AR	M+T	compound I	/	Biotinidase deficiency
**AP0913**	***BTD***	12359137	F596	P609	/	649	Caucasian	Non-EA	NM_000060.4	c.1388G>A	p.Cys463Tyr	c.98_104delin	p.Cys33Phefs*	AR	M+T	compound I	/	Biotinidase deficiency
**AP0914**	***BTD***	12359137	F597	P610	/	481	Caucasian	Non-EA	NM_000060.4	c.1612C>T	p.Arg538Cys	c.1612C>T	p.Arg538Cys	AR	M+M	homo	/	Biotinidase deficiency
**AP0915**	***BTD***	12359137	F598	P611	/	482	Caucasian	Non-EA	NM_000060.4	c.1612C>T	p.Arg538Cys	c.1612C>T	p.Arg538Cys	AR	M+M	homo	/	Biotinidase deficiency
**AP0916**	***BTD***	12227467	F599	P612	/	1.1	Caucasian	Non-EA	NM_000060.4	c.1330G>C	p.Asp444His	c.511G>A+c.1	p.Ala171Thr +	AR	M+M	compound I	/	Biotinidase deficiency
**AP0917**	***BTD***	12227467	F599	P613	/	1.2	Caucasian	Non-EA	NM_000060.4	c.1330G>C	p.Asp444His	c.511G>A+c.1	p.Ala171Thr +	AR	M+M	compound I	/	Biotinidase deficiency
**AP0918**	***BTD***	12227467	F600	P614	/	2.1	Caucasian	Non-EA	NM_000060.4	c.1330G>C	p.Asp444His	c.1595C>T	p.Thr532Met	AR	M+M	compound I	/	Biotinidase deficiency
**AP0919**	***BTD***	12227467	F600	P615	/	2.2	Caucasian	Non-EA	NM_000060.4	c.1330G>C	p.Asp444His	c.1595C>T	p.Thr532Met	AR	M+M	compound I	/	Biotinidase deficiency
**AP0920**	***BTD***	12227467	F601	P616	/	3	Caucasian	Non-EA	NM_000060.4	c.1330G>C	p.Asp444His	c.1211C>T	p.Thr404Ile	AR	M+M	compound I	/	Biotinidase deficiency
**AP0921**	***BTD***	12227467	F602	P617	/	4	Caucasian	Non-EA	NM_000060.4	c.1330G>C	p.Asp444His	c.1211C>T	p.Thr404Ile	AR	M+M	compound I	/	Biotinidase deficiency
**AP0922**	***BTD***	11313766	F603	P618	/	1	Austrian	Non-EA	NM_000060.4	c.98_104delinsTCC	p.Cys33Phefs*36	c.98_104delin	p.Cys33Phefs*	AR	T+T	homo	/	Biotinidase deficiency
**AP0923**	***BTD***	11313766	F604	P619	/	2	Turkish	Non-EA	NM_000060.4	c.98_104delinsTCC	p.Cys33Phefs*36	c.98_104delin	p.Cys33Phefs*	AR	T+T	homo	/	Biotinidase deficiency
**AP0924**	***BTD***	11313766	F605	P620	/	3	Turkish	Non-EA	NM_000060.4	c.98_104delinsTCC	p.Cys33Phefs*36	c.470G>A	p.Arg157His	AR	M+T	compound I	/	Biotinidase deficiency
**AP0925**	***BTD***	11313766	F606	P621	/	4	Austrian	Non-EA	NM_000060.4	c.98_104delinsTCC	p.Cys33Phefs*36	c.1368A>C	p.Gln456His	AR	M+T	compound I	/	Biotinidase deficiency
**AP0926**	***BTD***	11313766	F607	P622	/	5	Austrian	Non-EA	NM_000060.4	c.1368A>C	p.Gln456His	c.1368A>C	p.Gln456His	AR	M+M	compound I	/	Biotinidase deficiency
**AP0927**	***BTD***	11313766	F608	P623	/	8	Turkish	Non-EA	NM_000060.4	c.1595C>T	p.Thr532Met	c.1595C>T	p.Thr532Met	AR	M+M	homo	/	Biotinidase deficiency
**AP0928**	***BTD***	11313766	F609	P624	/	35	Austrian	Non-EA	NM_000060.4	c.98_104delinsTCC	p.Cys33Phefs*36	c.184G>A	p.Val62Met	AR	M+T	compound I	/	Biotinidase deficiency
**AP0929**	***BTD***	11313766	F610	P625	/	9	Austrian	Non-EA	NM_000060.4	c.1368A>C	p.Gln456His	c.[511G>A; 13	p.Ala171Thr +	AR	M+M	compound I	/	Biotinidase deficiency
**AP0930**	***BTD***	11313766	F611	P626	/	10	Yugoslavian	Non-EA	NM_000060.4	c.1368A>C	p.Gln456His	c.1595C>T	p.Thr532Met	AR	M+M	compound I	/	Biotinidase deficiency
**AP0931**	***BTD***	11313766	F612	P627	/	12	Turkish	Non-EA	NM_000060.4	c.1595C>T	p.Thr532Met	c.1595C>T	p.Thr532Met	AR	M+M	homo	/	Biotinidase deficiency
**AP0932**	***BTD***	11313766	F613	P628	/	7	Turkish	Non-EA	NM_000060.4	c.1368A>C	p.Gln456His	c.1368A>C	p.Gln456His	AR	M+M	compound I	/	Biotinidase deficiency
**AP0933**	***BTD***	11313766	F614	P629	/	13	Turkish	Non-EA	NM_000060.4	c.470G>A	p.Arg157His	/	/	AD	M	heter	/	Biotinidase deficiency
**AP0934**	***BTD***	11313766	F615	P630	/	14	Yugoslavian	Non-EA	NM_000060.4	c.470G>A	p.Arg157His	/	/	AD	M	heter	/	Biotinidase deficiency
**AP0935**	***BTD***	11313766	F616	P631	/	15	Austrian	Non-EA	NM_000060.4	c.470G>A	p.Arg157His	/	/	AD	M	heter	/	Biotinidase deficiency
**AP0936**	***BTD***	11313766	F617	P632	/	16	Austrian	Non-EA	NM_000060.4	c.1595C>T	p.Thr532Met	c.1595C>T	p.Thr532Met	AR	M+M	homo	/	Biotinidase deficiency
**AP0937**	***BTD***	11313766	F618	P633	/	17	Turkish	Non-EA	NM_000060.4	c.184G>A	p.Val62Met	c.1268G>C	p.Cys423Trp	AR	M+M	compound I	/	Biotinidase deficiency
**AP0938**	***BTD***	11313766	F619	P634	/	18	Austrian	Non-EA	NM_000060.4	c.1368A>C	p.Gln456His	/	/	AD	M	heter	/	Biotinidase deficiency
**AP0939**	***BTD***	11313766	F620	P635	/	35	Austrian	Non-EA	NM_000060.4	c.1595C>T	p.Thr532Met	c.1627G>C	p.Asp543His	AR	M+M	compound I	/	Biotinidase deficiency
**AP0940**	***BTD***	11313766	F621	P636	/	19	Polish	Non-EA	NM_000060.4	c.1368A>C	p.Gln456His	/	/	AD	M	heter	/	Biotinidase deficiency
**AP0941**	***BTD***	11313766	F622	P637	/	20	Caucasian	Non-EA	NM_000060.4	c.1368A>C	p.Gln456His	/	/	AD	M	heter	/	Biotinidase deficiency
**AP0942**	***BTD***	11313766	F623	P638	/	21	Caucasian	Non-EA	NM_000060.4	c.98_104delinsTCC	p.Cys33Phefs*36	c.1330G>C	p.Asp444His	AR	M+T	compound I	/	Biotinidase deficiency
**AP0943**	***BTD***	10801053	F624	P639	/	238	Caucasian	Non-EA	NM_000060.4	c.171T>G	p.Tyr57*	c.171T>G	p.Tyr57*	AR	T+T	homo	/	Biotinidase deficiency
**AP0944**	***BTD***	10801053	F625	P640	/	241	Caucasian	Non-EA	NM_000060.4	c.98_104delinsTCC	p.Cys33Phefs*36	c.98_104delin	p.Cys33Phefs*	AR	T+T	homo	/	Biotinidase deficiency
**AP0945**	***BTD***	10801053	F626	P641	/	244	Caucasian	Non-EA	NM_000060.4	c.587C>G	p.Thr196Arg	c.587C>G	p.Thr196Arg	AR	M+M	homo	/	Biotinidase deficiency
**AP0946**	***BTD***	10801053	F627	P642	/	246	Caucasian	Non-EA	NM_000060.4	c.98_104delinsTCC	p.Cys33Phefs*36	c.98_104delin	p.Cys33Phefs*	AR	T+T	homo	/	Biotinidase deficiency
**AP0947**	***BTD***	10801053	F628	P643	/	266	Caucasian	Non-EA	NM_000060.4	c.235C>T	p.Arg79Cys	c.235C>T	p.Arg79Cys	AR	M+M	homo	/	Biotinidase deficiency
**AP0948**	***BTD***	10801053	F629	P644	/	271	Caucasian	Non-EA	NM_000060.4	c.98_104delinsTCC	p.Cys33Phefs*36	c.98_104delin	p.Cys33Phefs*	AR	T+T	homo	/	Biotinidase deficiency
**AP0949**	***BTD***	10801053	F630	P645	/	274	Caucasian	Non-EA	NM_000060.4	c.1612C>T	p.Arg538Cys	c.1612C>T	p.Arg538Cys	AR	M+M	compound I	/	Biotinidase deficiency
**AP0950**	***BTD***	10801053	F631	P646	/	342	Caucasian	Non-EA	NM_000060.4	c.98_104delinsTCC	p.Cys33Phefs*36	c.98_104delin	p.Cys33Phefs*	AR	T+T	homo	/	Biotinidase deficiency
**AP0951**	***BTD***	10801053	F632	P647	/	345	Caucasian	Non-EA	NM_000060.4	c.98_104delinsTCC	p.Cys33Phefs*36	c.98_104delin	p.Cys33Phefs*	AR	T+T	homo	/	Biotinidase deficiency
**AP0952**	***BTD***	10801053	F633	P648	/	354	Caucasian	Non-EA	NM_000060.4	c.98_104delinsTCC	p.Cys33Phefs*36	c.98_104delin	p.Cys33Phefs*	AR	T+T	homo	/	Biotinidase deficiency
**AP0953**	***BTD***	10801053	F634	P649	/	583	Caucasian	Non-EA	NM_000060.4	c.1368A>C	p.Gln456His	c.1368A>C	p.Gln456His	AR	M+M	compound I	/	Biotinidase deficiency
**AP0954**	***BTD***	10801053	F635	P650	/	540	Caucasian	Non-EA	NM_000060.4	c.235C>T	p.Arg79Cys	c.235C>T	p.Arg79Cys	AR	M+M	homo	/	Biotinidase deficiency
**AP0955**	***BTD***	10801053	F636	P651	/	563	Caucasian	Non-EA	NM_000060.4	c.1369G>A	p.Val457Met	c.1369G>A	p.Val457Met	AR	M+M	homo	/	Biotinidase deficiency
**AP0956**	***BTD***	10801053	F637	P652	/	267	Caucasian	Non-EA	NM_000060.4	c.235C>T	p.Arg79Cys	c.470G>A	p.Arg157His	AR	M+M	compound I	/	Biotinidase deficiency
**AP0957**	***BTD***	10801053	F638	P653	/	268	Caucasian	Non-EA	NM_000060.4	c.1595C>T	p.Thr532Met	c.1595C>T	p.Thr532Met	AR	M+M	homo	/	Biotinidase deficiency
**AP0958**	***BTD***	10801053	F639	P654	/	262	Caucasian	Non-EA	NM_000060.4	c.235C>T	p.Arg79Cys	c.235C>T	p.Arg79Cys	AR	M+M	homo	/	Biotinidase deficiency
**AP0959**	***BTD***	10801053	F640	P655	/	263	Caucasian	Non-EA	NM_000060.4	c.1595C>T	p.Thr532Met	c.557G>A	p.Cys186Tyr	AR	M+M	compound I	/	Biotinidase deficiency
**AP0960**	***BTD***	10801053	F641	P656	/	264	Caucasian	Non-EA	NM_000060.4	c.1595C>T	p.Thr532Met	c.1595C>T	p.Thr532Met	AR	M+M	homo	/	Biotinidase deficiency
**AP0961**	***BTD***	10801053	F641	P657	/	265	Caucasian	Non-EA	NM_000060.4	c.1595C>T	p.Thr532Met	/	/	AD	M	heter	/	Biotinidase deficiency
**AP0962**	***BTD***	10801053	F642	P658	/	269	Caucasian	Non-EA	NM_000060.4	c.98_104delinsTCC	p.Cys33Phefs*36	c.98_104delin	p.Cys33Phefs*	AR	T+T	homo	/	Biotinidase deficiency
**AP0963**	***BTD***	10801053	F643	P659	/	348	Caucasian	Non-EA	NM_000060.4	c.235C>T	p.Arg79Cys	c.235C>T	p.Arg79Cys	AR	M+M	homo	/	Biotinidase deficiency
**AP0964**	***BTD***	10801053	F644	P660	/	351	Caucasian	Non-EA	NM_000060.4	c.100G>A	p.Gly34Ser	c.1369G>A	p.Val457Met	AR	M+M	compound I	/	Biotinidase deficiency
**AP0965**	***BTD***	10801053	F645	P661	/	571	Caucasian	Non-EA	NM_000060.4	c.[511G>A; 1330G>C]	p.Ala171Thr+p.As	c.[511G>A; 13	p.Ala171Thr+	AR	M+M	homo	/	Biotinidase deficiency
**AP0966**	***BTD***	10801053	F646	P662	/	544	Caucasian	Non-EA	NM_000060.4	c.235C>T	p.Arg79Cys	c.235C>T	p.Arg79Cys	AR	M+M	homo	/	Biotinidase deficiency
**AP0967**	***BTD***	10801053	F647	P663	/	592	Caucasian	Non-EA	NM_000060.4	c.929G>A	p.Gly310Glu	c.929G>A	p.Gly310Glu	AR	M+M	homo	/	Biotinidase deficiency
**AP0968**	***BTD***	10801053	F648	P664	/	568	Caucasian	Non-EA	NM_000060.4	c.1595C>T	p.Thr532Met	c.1595C>T	p.Thr532Met	AR	M+M	homo	/	Biotinidase deficiency

**AP0969**	***BTD***	10801053	F648	P665	/	595	Caucasian	Non-EA	NM_000060.4	c.1368A>C	p.Gln456His	c.[511G>A; 13	p.Ala171Thr +	AR	M+M	compound I	/
**AP0970**	***BTD***	10801053	F649	P666	/	569	Caucasian	Non-EA	NM_000060.4	c.1595C>T	p.Thr532Met	c.1595C>T	p.Thr532Met	AR	M+M	homo	/
**AP0971**	***BTD***	10801053	F650	P667	/	547	Caucasian	Non-EA	NM_000060.4	c.[511G>A; 1330G>C]	p.Ala171Thr+p.Asp	c.[511G>A; 13	p.Ala171Thr+p	AR	M+M	homo	/
**AP0972**	***BTD***	10801053	F650	P668	/	548	Caucasian	Non-EA	NM_000060.4	c.[511G>A; 1330G>C]	p.Ala171Thr+p.Asp	c.[511G>A; 13	p.Ala171Thr+p	AR	M+M	homo	/
**AP0973**	***BTD***	9654207	F651	P669	/	285	Caucasian	Non-EA	NM_000060.4	c.1330G>C	p.Asp444His	c.511G>A+c.1	p.Ala171Thr+p	AR	M+M	compound I	/
**AP0974**	***BTD***	9654207	F652	P670	/	390	Caucasian	Non-EA	NM_000060.4	c.1330G>C	p.Asp444His	c.511G>A+c.1	p.Ala171Thr+p	AR	M+M	compound I	/
**AP0975**	***BTD***	9654207	F653	P671	/	398	Caucasian	Non-EA	NM_000060.4	c.1330G>C	p.Asp444His	c.511G>A+c.1	p.Ala171Thr+p	AR	M+M	compound I	/
**AP0976**	***BTD***	9654207	F654	P672	/	545	Caucasian	Non-EA	NM_000060.4	c.1330G>C	p.Asp444His	c.511G>A+c.1	p.Ala171Thr+p	AR	M+M	compound I	/
**AP0977**	***BTD***	9654207	F655	P673	/	53	Caucasian	Non-EA	NM_000060.4	c.1330G>C	p.Asp444His	c.1612C>T	p.Arg538Cys	AR	M+M	compound I	/
**AP0978**	***BTD***	9654207	F656	P674	/	212	Caucasian	Non-EA	NM_000060.4	c.1330G>C	p.Asp444His	c.1612C>T	p.Arg538Cys	AR	M+M	compound I	/
**AP0979**	***BTD***	9654207	F657	P675	/	325	Caucasian	Non-EA	NM_000060.4	c.1330G>C	p.Asp444His	c.1368A>C	p.Gln456His	AR	M+M	compound I	/
**AP0980**	***BTD***	9654207	F658	P676	/	384	Caucasian	Non-EA	NM_000060.4	c.1330G>C	p.Asp444His	c.1368A>C	p.Gln456His	AR	M+M	compound I	/
**AP0981**	***BTD***	9654207	F659	P677	/	408	Caucasian	Non-EA	NM_000060.4	c.1330G>C	p.Asp444His	c.1368A>C	p.Gln456His	AR	M+M	compound I	/
**AP0982**	***BTD***	9654207	F660	P678	/	448	Caucasian	Non-EA	NM_000060.4	c.1330G>C	p.Asp444His	c.1368A>C	p.Gln456His	AR	M+M	compound I	/
**AP0983**	***BTD***	9654207	F661	P679	/	519	Caucasian	Non-EA	NM_000060.4	c.1330G>C	p.Asp444His	c.1368A>C	p.Gln456His	AR	M+M	compound I	/
**AP0984**	***BTD***	9654207	F662	P680	/	185	Caucasian	Non-EA	NM_000060.4	c.1330G>C	p.Asp444His	c.98_104delin	p.Cys33Phefs*	AR	M+T	compound I	/
**AP0985**	***BTD***	9654207	F663	P681	/	410	Caucasian	Non-EA	NM_000060.4	c.1330G>C	p.Asp444His	c.98_104delin	p.Cys33Phefs*	AR	M+T	compound I	/
**AP0986**	***BTD***	9654207	F664	P682	/	556	Caucasian	Non-EA	NM_000060.4	c.1330G>C	p.Asp444His	c.98_104delin	p.Cys33Phefs*	AR	M+T	compound I	/
**AP0987**	***BTD***	9654207	F665	P683	/	334	Caucasian	Non-EA	NM_000060.4	c.1330G>C	p.Asp444His	c.382T>G	p.Phe128Val	AR	M+M	compound I	/
**AP0988**	***BTD***	9654207	F666	P684	/	375	Caucasian	Non-EA	NM_000060.4	c.1330G>C	p.Asp444His	c.933delT	p.Ser311Argfs	AR	M+T	compound I	/
**AP0989**	***BTD***	9654207	F667	P685	/	503	Caucasian	Non-EA	NM_000060.4	c.1330G>C	p.Asp444His	c.682G>T	p.Asp228Tyr	AR	M+M	compound I	/
**AP0990**	***BTD***	9654207	F668	P686	/	505	Caucasian	Non-EA	NM_000060.4	c.1330G>C	p.Asp444His	c.1595C>T	p.Thr532Met	AR	M+M	compound I	/
**AP0991**	***BTD***	9654207	F669	P687	/	143	Caucasian	Non-EA	NM_000060.4	c.1330G>C	p.Asp444His	c.1352G>A	p.Gly451Asp	AR	M+M	compound I	/
**AP0992**	***BTD***	9506660	F670	P688	/	P1	Caucasian	Non-EA	NM_000060.4	p.Val457Met	p.Val457Met	c.1369G>A	p.Val457Met	AR	M+M	compound I	Male
**AP0993**	***BTD***	9506660	F671	P689	/	P2	Caucasian	Non-EA	NM_000060.4	c.643C>T	p.Leu215Phe	c.1096T>C	p.Ser366Pro	AR	M+M	compound I	Male
**AP0994**	***BTD***	9506660	F672	P690	/	P3	Caucasian	Non-EA	NM_000060.4	c.1612C>T	p.Arg538Cys	c.1612C>T	p.Arg538Cys	AR	M+M	homo	Female
**AP0995**	***BTD***	9506660	F673	P691	/	P4	Caucasian	Non-EA	NM_000060.4	c.98_104delinsTCC	p.Cys33Phefs*36	c.643C>T	p.Leu215Phe	AR	M+T	compound I	Male
**AP0996**	***BTD***	9232193	F674	P692	/	P54	Caucasian	Non-EA	NM_000060.4	c.1368A>C	p.Gln456His	/	/	AD	M	heter	/
**AP0997**	***BTD***	9232193	F675	P693	/	P64	Caucasian	Non-EA	NM_000060.4	c.1368A>C	p.Gln456His	/	/	AD	M	heter	/
**AP0998**	***BTD***	9232193	F676	P694	/	P82	Caucasian	Non-EA	NM_000060.4	c.1368A>C	p.Gln456His	/	/	AD	M	heter	/
**AP0999**	***BTD***	9232193	F677	P695	/	P121	Caucasian	Non-EA	NM_000060.4	c.1368A>C	p.Gln456His	c.[511G>A; 13	p.Ala171Thr +	AR	M+M	compound I	/
**AP1000**	***BTD***	9232193	F678	P696	/	P122	Caucasian	Non-EA	NM_000060.4	c.1368A>C	p.Gln456His	c.1368A>C	p.Gln456His	AR	M+M	homo	/
**AP1001**	***BTD***	9232193	F679	P697	/	P133	Caucasian	Non-EA	NM_000060.4	c.1368A>C	p.Gln456His	c.[511G>A; 13	p.Ala171Thr +	AR	M+M	compound I	/
**AP1002**	***BTD***	9232193	F680	P698	/	P151	Caucasian	Non-EA	NM_000060.4	c.1368A>C	p.Gln456His	/	/	AD	M	heter	/
**AP1003**	***BTD***	9232193	F681	P699	/	P154	Caucasian	Non-EA	NM_000060.4	c.1368A>C	p.Gln456His	c.1612C>T	p.Arg538Cys	AR	M+M	compound I	/
**AP1004**	***BTD***	9232193	F681	P700	/	P155	Caucasian	Non-EA	NM_000060.4	c.1368A>C	p.Gln456His	c.1612C>T	p.Arg538Cys	AR	M+M	compound I	/
**AP1005**	***BTD***	9232193	F682	P701	/	P162	Italian	Non-EA	NM_000060.4	c.1368A>C	p.Gln456His	c.[511G>A; 13	p.Ala171Thr +	AR	M+M	compound I	/
**AP1006**	***BTD***	9232193	F683	P702	/	P164	Irish, Ital, Polish/Iris	Non-EA	NM_000060.4	c.1368A>C	p.Gln456His	c.[511G>A; 13	p.Ala171Thr +	AR	M+M	compound I	/
**AP1007**	***BTD***	9232193	F684	P703	/	P166	Ger, Irish, Eng, Nat	Non-EA	NM_000060.4	c.1368A>C	p.Gln456His	c.[511G>A; 13	p.Ala171Thr +	AR	M+M	compound I	/
**AP1008**	***BTD***	9232193	F684	P704	/	P167	Ger, Irish, Eng, Nat	Non-EA	NM_000060.4	c.1368A>C	p.Gln456His	c.[511G>A; 13	p.Ala171Thr +	AR	M+M	compound I	/
**AP1009**	***BTD***	9232193	F685	P705	/	P168	Ger, French, Dutch	Non-EA	NM_000060.4	c.1368A>C	p.Gln456His	c.[511G>A; 13	p.Ala171Thr +	AR	M+M	compound I	/
**AP1010**	***BTD***	9232193	F686	P706	/	P169	Caucasian	Non-EA	NM_000060.4	c.1368A>C	p.Gln456His	c.98_104delin	p.Cys33Phefs*	AR	M+T	compound I	/
**AP1011**	***BTD***	9232193	F687	P707	/	P206	Dutch	Non-EA	NM_000060.4	c.1368A>C	p.Gln456His	/	/	AD	M	heter	/
**AP1012**	***BTD***	9099842	F688	P708	/	8	Caucasian	Non-EA	NM_000060.4	c.1612C>T	p.Arg538Cys	/	/	AD	M	heter	/
**AP1013**	***BTD***	9099842	F689	P709	/	9	Caucasian	Non-EA	NM_000060.4	c.1612C>T	p.Arg538Cys	/	/	AD	M	heter	/
**AP1014**	***BTD***	9099842	F690	P710	/	13	Caucasian	Non-EA	NM_000060.4	c.1612C>T	p.Arg538Cys	/	/	AD	M	heter	/
**AP1015**	***BTD***	9099842	F691	P711	/	14	Caucasian	Non-EA	NM_000060.4	c.1612C>T	p.Arg538Cys	/	/	AD	M	heter	/
**AP1016**	***BTD***	9099842	F692	P712	/	42	Caucasian	Non-EA	NM_000060.4	c.1612C>T	p.Arg538Cys	/	/	AD	M	heter	/
**AP1017**	***BTD***	9099842	F693	P713	/	90	Caucasian	Non-EA	NM_000060.4	c.1612C>T	p.Arg538Cys	/	/	AD	M	heter	/
**AP1018**	***BTD***	9099842	F694	P714	/	190	Caucasian	Non-EA	NM_000060.4	c.1612C>T	p.Arg538Cys	/	/	AD	M	heter	/
**AP1019**	***BTD***	9099842	F695	P715	/	218	Caucasian	Non-EA	NM_000060.4	c.1612C>T	p.Arg538Cys	c.1612C>T	p.Arg538Cys	AR	M+M	homo	/
**AP1020**	***BTD***	9099842	F696	P716	/	253	Caucasian	Non-EA	NM_000060.4	c.1612C>T	p.Arg538Cys	c.1612C>T	p.Arg538Cys	AR	M+M	homo	/
**AP1021**	***BTD***	9099842	F697	P717	/	55	English, Irish*/? (A	Non-EA	NM_000060.4	c.1612C>T	p.Arg538Cys	c.98_104delin	p.Cys33Phefs*	AR	M+T	compound I	/
**AP1022**	***BTD***	9099842	F698	P718	/	86	Caucasian	Non-EA	NM_000060.4	c.1612C>T	p.Arg538Cys	c.98_104delin	p.Cys33Phefs*	AR	M+T	compound I	/
**AP1023**	***BTD***	9099842	F699	P719	/	161	French, German, E	Non-EA	NM_000060.4	c.1612C>T	p.Arg538Cys	c.98_104delin	p.Cys33Phefs*	AR	M+T	compound I	/
**AP1024**	***BTD***	9099842	F700	P720	/	172	German, Dutch/Sc	Non-EA	NM_000060.4	c.1612C>T	p.Arg538Cys	c.98_104delin	p.Cys33Phefs*	AR	M+T	compound I	/
**AP1025**	***BTD***	9099842	F701	P860	/	191	French, German/G	Non-EA	NM_000060.4	c.1612C>T	p.Arg538Cys	c.98_104delin	p.Cys33Phefs*	AR	M+T	compound I	/
**AP1026**	***BTD***	19728141	F702	P861	/		Chinese	EA	NM_00137065	c.1384delA	p.(Arg462Glyfs*3	c.1384delA	p.(Arg462Glyf	AR	M+T	homo	/
**AP1027**	***BTD***	19728141	F703	P862	/		Chinese	EA	NM_00137065	c.98_104delinsTCC	p.Cys33Phefs*36	c.1493_1494ir	p.(Leu498Phe	AR	T+T	compound I	/
**AP1028**	***BTD***	19728141	F704	P863	/		Chinese	EA	NM_00137065	c.1284C>A	p.(Tyr428*)	c.1369G>A	p.(Val457Met)	AR	M+T	compound I	/
**AP1029**	***BTD***	19728141	F705	P864	/		Chinese	EA	NM_00137065	c.1157G>A	p.(Trp386*)	c.1493_1494ir	p.(Leu498Phe	AR	T+T	compound I	/
**AP1030**	***C12orf65***	20598281	F001	P001	/	Patient 1	Japanese	EA	NM_152269.3	c.394C>T	p.Arg132*	c.394C>T	p.Arg132*	AR	T+T	homo	Female
**AP1031**	***C12orf65***	20598281	F002	P002	/	Patient 2	Japanese	EA	NM_152269.3	c.394C>T	p.Arg132*	c.394C>T	p.Arg132*	AR	T+T	homo	Male
**AP1032**	***C12orf65***	23188110	F003	P003	/	IV-6	Japanese	EA	NM_152269.3	c.415C>T	p.Gln139*	c.415C>T	p.Gln139*	AR	T+T	homo	Male
**AP1033**	***C12orf65***	23188110	F004	P004	/	IV-3	Japanese	EA	NM_152269.3	c.415C>T	p.Gln139*	c.415C>T	p.Gln139*	AR	T+T	homo	Male
**AP1034**	***C12orf65***	24080142	F005	P005	/	MR013-01	German	Non-EA	NM_152269.3	c.415C>T	p.Gln139*	c.415C>T	p.Gln139*	AR	T+T	homo	Female
**AP1035**	***C12orf65***	24080142	F005	P006	/	MR013-02	Indian	Non-EA	NM_152269.3	c.346delG	p.Val116*	c.346delG	p.Val116*	AR	T+T	homo	Male
**AP1036**	***C12orf65***	24080142	F005	P007	/	MR013-06	Indian	Non-EA	NM_152269.3	c.346delG	p.Val116*	c.346delG	p.Val116*	AR	T+T	homo	Female
**AP1037**	***C12orf65***	24198383	F006	P008	/	Case 1	Indian	Non-EA	NM_152269.3	c.346delG	p.Val116*	c.346delG	p.Val116*	AR	T+T	homo	Male
**AP1038**	***C12orf65***	24198383	F006	P009	/	Case 2	Indian	Non-EA	NM_152269.3	c.210delA	p.Gly72Alafs*13	c.96_99 dupA	p.Pro34IlefsX2	AR	T+T	homo	Male
**AP1039**	***C12orf65***	24198383	F006	P010	/	Case 3	Israel	Non-EA	NM_152269.3	c.210delA	p.Gly72Alafs*13	c.96_99 dupA	p.Pro34IlefsX2	AR	T+T	homo	Female
**AP1040**	***C12orf65***	24284555	F007	P011	/	Case 2	Israel	Non-EA	NM_152269.3	c.413_417 del	p.Lys138Argfs*17	c.413_417 del	p.Lys138Argfs	AR	T+T	homo	Male
**AP1041**	***C12orf65***	24284555	F007	P012	/	Case 1	Israel	Non-EA	NM_152269.3	c.413_417 del	p.Lys138Argfs*17	c.413_417 del	p.Lys138Argfs	AR	T+T	homo	Female
**AP1042**	***C12orf65***	24424123	F008	P013	Family A	IV-1	Israel	Non-EA	NM_152269.3	c.413_417 del	p.Lys138Argfs*17	c.413_417 del	p.Lys138Argfs	AR	T+T	homo	Male
**AP1043**	***C12orf65***	24424123	F008	P014	Family A	IV-2	Israel	Non-EA	NM_152269.3	c.413_417 del	p.Lys138Argfs*17	c.413_417 del	p.Lys138Argfs	AR	T+T	homo	Male
**AP1044**	***C12orf65***	24424123	F008	P015	Family A	IV-4	Israel	Non-EA	NM_152269.3	c.282+2T>A	p.?	c.282+2T>A	p.?	AR	T+T	homo	Male
**AP1045**	***C12orf65***	24424123	F008	P016	Family A	IV-3	Israel	Non-EA	NM_152269.3	c.282+2T>A	p.?	c.282+2T>A	p.?	AR	T+T	homo	Female
**AP1046**	***C12orf65***	24424123	F009	P017	Family B	V-1	Israeli	Non-EA	NM_152269.3	c.282+2T>A	p.?	c.282+2T>A	p.?	AR	T+T	homo	Female
**AP1047**	***C12orf65***	24424123	F009	P018	Family B	V-2	Indian	Non-EA	NM_152269.3	c.346delG	p.Val116*	c.346delG	p.Val116*	AR	T+T	homo	Male
**AP1048**	***C12orf65***	24424123	F009	P019	Family B	V-3	Indian	Non-EA	NM_152269.3	c.346delG	p.Val116*	c.346delG	p.Val116*	AR	T+T	homo	Female
**AP1049**	***C12orf65***	25995486	F010	P020	F1	II-3	Japanese	EA	NM_152269.3	c.7_8del	p.Thr3Argfs*54	c.394C>T	p.Arg132*	AR	T+T	compound I	Female
**AP1050**	***C12orf65***	25995486	F010	P021	F1	II-4	Asian	EA	NM_152269.3	c.7_8del	p.Thr3Argfs*54	c.394C>T	p.Arg132*	AR	T+T	compound I	Female
**AP1051**	***C12orf65***	27858754	F011	P022	/	/	Caucasian	Non-EA	NM_152269.3	c.207_220del	p.Pro70Asnfs*28	c.207_220del	p.Pro70Asnfs*	AR	T+T	compound I	/
**AP1052**	***C12orf65***	28639102	F012	P023	/	/	Asian	EA	NM_152269.3	c.394C>T	p.Arg132*	c.447_449del(	p.E150Kfs	AR	T+T	compound I	Male
**AP1053**	***C12orf65***	32478789	F013	P024	/	/	Irish	Non-EA	NM_152269.3	c.96_99dupATCC	p.Pro34Ilefs*25	c.96_99dupAT	p.Pro34Ilefs*2	AR	T+T	homo	Female
**AP1054**	***C12orf65***	31753091	F014	P025	/	/	Japanese	EA	NM_152269.3	c.96_99dupATCC	p.Pro34Ilefs*25	c.96_99dupAT	p.Pro34Ilefs*2	AR	T+T	homo	/
**AP1055**	***C12orf65***	25058219	F015	P026	/		Caucasian	Non-EA	NM_152269.3	c.96_99dupATCC	p.Pro34Ilefs*25	c.96_99dupAT	p.Pro34Ilefs*2	AR	T+T	homo	Female
**AP1056**	***C12orf65***	26380172	F016	P027	/	Patient 1	Englander	Non-EA	NM_152269.3	c.96_99dupATCC	p.Pro34Ilefs*25	c.96_99dupAT	p.Pro34Ilefs*2	AR	T+T	homo	Male
**AP1057**	***C12orf65***	26380172	F016	P028	/	Patient 2	Hungarian Roma e	Non-EA	NM_152269.3	c.282G>A	p.?	c.282G>A	p.?	AR	M+M	homo	Female
**AP1058**	***C12orf65***	26380172	F017	P029	/	Patient 3	Northern England	Non-EA	NM_152269.3	c.171_172delGA	p.Asn58*	c.171_172del(	p.Asn58*	AR	T+T	homo	/
**AP1059**	***C12orf65***	26380172	F018	P030	/	Patient 4	Hungarian Roma	Non-EA	NM_152269.3	c.127_146del	p.Met43Profs*8	c.127_146del	p.Met43Profs*i	AR	T+T	homo	Male
**AP1060**	***C12orf65***	28804760	F019	P031	/		Japanese	EA	NM_152269.3	c.171_172delGA	p.Asn58*	c.171_172del(	p.Asn58*	AR	T+T	homo	Female
**AP1061**	***C12orf65***	33600046	F020	P032	/		German	Non-EA	NM_152269.3	c.127_146del	p.Met43Profs*8	c.127_146del	p.Met43Profs*i	AR	T+T	homo	/
**AP1062**	***C12orf65***	28091420	F021		/		Chinese	EA	NM_152269.3	c.7_8del	p.Thr3Argfs*54	c.394C>T	p.Arg132*	AR	T+T	compound I	Female
**AP1063**	***C19ORF12***	21981780	F001	P001	28	II-1	Polish	Non-EA	NM_00103172	c.204_214del11	p.Gly69Argfs*10	c.204_214del1	p.Gly69Argfs*1	AR	T+T	homo	Male
**AP1064**	***C19ORF12***	21981780	F001	P002	28	II-4	Polish	Non-EA	NM_00103172	c.204_214del11	p.Gly69Argfs*10	c.204_214del1	p.Gly69Argfs*1	AR	T+T	homo	Male
**AP1065**	***C19ORF12***	21981780	F001	P003	28	II-5	Polish	Non-EA	NM_00103172	c.204_214del11	p.Gly69Argfs*10	c.204_214del1	p.Gly69Argfs*1	AR	T+T	homo	Male
**AP1066**	***C19ORF12***	21981780	F002	P004	4	II-1	Polish	Non-EA	NM_00103172	c.204_214del11	p.Gly69Argfs*10	c.204_214del1	p.Gly69Argfs*1	AR	T+T	homo	Female
**AP1067**	***C19ORF12***	21981780	F002	P005	4	II-2	Polish	Non-EA	NM_00103172	c.204_214del11	p.Gly69Argfs*10	c.204_214del1	p.Gly69Argfs*1	AR	T+T	homo	Female
**AP1068**	***C19ORF12***	21981780	F003	P006	21	II-1	Polish	Non-EA	NM_00103172	c.204_214del11	p.Gly69Argfs*10	c.204_214del1	p.Gly69Argfs*1	AR	T+T	homo	Male
**AP1069**	***C19ORF12***	21981780	F003	P007	21	II-2	Polish	Non-EA	NM_00103172	c.204_214del11	p.Gly69Argfs*10	c.204_214del1	p.Gly69Argfs*1	AR	T+T	homo	Female
**AP1070**	***C19ORF12***	21981780	F004	P008	37	II-1	Polish	Non-EA	NM_00103172	c.204_214del11	p.Gly69Argfs*10	c.204_214del1	p.Gly69Argfs*1	AR	T+T	homo	Male
**AP1071**	***C19ORF12***	21981780	F004	P009	37	II-2	Polish	Non-EA	NM_00103172	c.204_214del11	p.Gly69Argfs*10	c.204_214del1	p.Gly69Argfs*1	AR	T+T	homo	Female
**AP1072**	***C19ORF12***	21981780	F005	P010	2		Polish	Non-EA	NM_00103172	c.205G>A	p.Gly69Arg	/	/	AD	M	heter	Male
**AP1073**	***C19ORF12***	21981780	F006	P011	3		Polish	Non-EA	NM_00103172	c.204_214del11	p.Gly69Argfs*10	c.204_214del1	p.Gly69Argfs*1	AR	T+T	homo	Male
**AP1074**	***C19ORF12***	21981780	F007	P012	5		Polish	Non-EA	NM_00103172	c.204_214del11	p.Gly69Argfs*10	c.204_214del1	p.Gly69Argfs*1	AR	T+T	homo	Male
**AP1075**	***C19ORF12***	21981780	F008	P013	6		Polish	Non-EA	NM_00103172	c.157G>A	p.Gly53Arg	c.204_214del1	p.Gly69Argfs*1	AR	M+T	compound I	Male
**AP1076**	***C19ORF12***	21981780	F009	P014	12		Polish	Non-EA	NM_00103172	c.204_214del11	p.Gly69Argfs*10	c.204_214del1	p.Gly69Argfs*1	AR	T+T	homo	Female
**AP1077**	***C19ORF12***	21981780	F010	P015	20		Polish	Non-EA	NM_00103172	c.204_214del11	p.Gly69Argfs*10	c.204_214del1	p.Gly69Argfs*1	AR	T+T	homo	Male
**AP1078**	***C19ORF12***	21981780	F011	P016	22		Polish	Non-EA	NM_00103172	c.204_214del11	p.Gly69Argfs*10	c.204_214del1	p.Gly69Argfs*1	AR	T+T	homo	Male
**AP1079**	***C19ORF12***	21981780	F012	P017	25		Polish	Non-EA	NM_00103172	c.204_214del11	p.Gly69Argfs*10	c.204_214del1	p.Gly69Argfs*1	AR	T+T	homo	Male
**AP1080**	***C19ORF12***	21981780	F013	P018	27		Polish	Non-EA	NM_00103172	c.32C>T	p.Thr11Met	c.204_214del1	p.Gly69Argfs*1	AR	M+T	compound I	Male
**AP1081**	***C19ORF12***	21981780	F014	P019	32		Polish	Non-EA	NM_00103172	c.204_214del11	p.Gly69Argfs*10	c.204_214del1	p.Gly69Argfs*1	AR	T+T	homo	Male
**AP1082**	***C19ORF12***	21981780	F015	P020	33		Polish	Non-EA	NM_00103172	c.194G>A	p.Gly65Glu	c.204_214del1	p.Gly69Argfs*1	AR	M+T	compound I	Male
**AP1083**	***C19ORF12***	21981780	F016	P021	34		Polish	Non-EA	NM_00103172	c.204_214del11	p.Gly69Argfs*10	c.204_214del1	p.Gly69Argfs*1	AR	T+T	homo	Male
**AP1084**	***C19ORF12***	21981780	F017	P022	38		Polish	Non-EA	NM_00103172	c.204_214del11	p.Gly69Argfs*10	c.204_214del1	p.Gly69Argfs*1	AR	T+T	homo	Female
**AP1085**	***C19ORF12***	21981780	F018	P023	39		Polish	Non-EA	NM_00103172	c.204_214del11	p.Gly69Argfs*10	c.204_214del1	p.Gly69Argfs*1	AR	T+T	homo	Male
**AP1086**	***C19ORF12***	21981780	F019	P024	41		Polish	Non-EA	NM_00103172	c.32C>T	p.Thr11Met	/	/	AD	M	heter	Female
**AP1087**	***C19ORF12***	21981780	F020	P025	40		Polish	Non-EA	NM_00103172	c.205G>A	p.Gly69Arg	c.424A>G	p.Lys142Glu	AR	M+M	compound I	Male
**AP1088**	***C19ORF12***	22508347	F021	P026	/	patient 1	Turkish	Non-EA	NM_00103172	c.362T>A	p.Leu121Gln	c.362T>A	p.Leu121Gln	AR	M+M	homo	Male
**AP1089**	***C19ORF12***	22508347	F021	P027	/	patient 2	Turkish	Non-EA	NM_00103172	c.362T>A	p.Leu121Gln	c.362T>A	p.Leu121Gln	AR	M+M	homo	Male
**AP1090**	***C19ORF12***	22584950	F022	P028	/	patient 1	German	Non-EA	NM_00103172	c.197 199del	p.Gly66del	c.32C>T	p.Thr11Met	AR	M+M	compound I	Female
**AP1091**	***C19ORF12***	22584950	F022	P029	/	patient 2	German	Non-EA	NM_00103172	c.197 199del	p.Gly66del	c.32C>T	p.Thr11Met	AR	M+M	compound I	Male
**AP1092**	***C19ORF12***	22584950	F023	P030	/	patient 3	German	Non-EA	NM_00103172	c.197 199del	p.Gly66del	c.204_214del1	p.Gly69Argfs*1	AR	M+T	compound I	Female
**AP1093**	***C19ORF12***	22704260	F024	P031	/	case1	Italian	Non-EA	NM_00103172	c.287A>C	p.Gln96Pro	c.287A>C	p.Gln96Pro	AR	M+M	homo	Male
**AP1094**	***C19ORF12***	22704260	F025	P032	/	case2	Italian	Non-EA	NM_00103172	c.172G>A	p.Gly58Ser	c.172G>A	p.Gly58Ser	AR	M+M	homo	Female
**AP1095**	***C19ORF12***	22704260	F026	P033	/	case3	Italian	Non-EA	NM_00103172	c.297insGCT	p.L99fs102	/	/	AD	Truncation	heter	Female
**AP1096**	***C19ORF12***	23160001	F027	P034	/	PNK101-3	Iranian	Non-EA	NM_00103172	c.191insG	p.Ala67GlyfsX14	c.191insG	p.Ala67GlyfsX	AR	T+T	homo	Female
**AP1097**	***C19ORF12***	23160001	F027	P035	/	PNK101-4	Iranian	Non-EA	NM_00103172	c.191insG	p.Ala67GlyfsX14	c.191insG	p.Ala67GlyfsX	AR	T+T	homo	Female
**AP1098**	***C19ORF12***	23160001	F028	P036	/	PNK109-3	Iranian	Non-EA	NM_00103172	c.404insT	p.Met135IlefsX15	c.404insT	p.Met135IlefsX	AR	T+T	homo	Female
**AP1099**	***C19ORF12***	23160001	F029	P037	/	PNK103-3	Iranian	Non-EA	NM_00103172	c.32C>T	p.Thr11Met	c.32C>T	p.Thr11Met	AR	M+M	homo	Male
**AP1100**	***C19ORF12***	23160001	F030	P038	/	PNK102-3	Iranian	Non-EA	NM_00103172	c.32C>T	p.Thr11Met	c.32C>T	p.Thr11Met	AR	M+M	homo	Male
**AP1101**	***C19ORF12***	23269600/3108	F031	P039	/	18-306	Polish	Non-EA	NM_00103172	c.227_237del11	p.Met76Thrfs*3	/	/	AD	Truncation	heter	Male
**AP1102**	***C19ORF12***	23269600/3108	F032	P040	/	18-307	Polish	Non-EA	NM_00103172	c.227_237del11	p. Met76Thrfs*3	/	/	AD	Truncation	heter	Male
**AP1103**	***C19ORF12***	23269600/3108	F033	P041	/	18-403	Polish	Non-EA	NM_00103172	c.227_237del11	p. Met76Thrfs*3	/	/	AD	Truncation	heter	Male
**AP1104**	***C19ORF12***	23269600/3108	F034	P042	/	18-411	Polish	Non-EA	NM_00103172	c.227_237del11	p. Met76Thrfs*3	/	/	AD	Truncation	heter	Male
**AP1105**	***C19ORF12***	23269600	F035	P043	/	220	Polish	Non-EA	NM_00103172	c.278delC	p.Pro93Leufs*26	/	/	AD	Truncation	heter	/
**AP1106**	***C19ORF12***	23269600	F036	P044	/	227	Polish	Non-EA	NM_00103172	c.256C>T	p.Gln86*	/	/	AD	Truncation	heter	/
**AP1107**	***C19ORF12***	23269600	F037	P045	/	392	Polish	Non-EA	NM_00103172	c.278dupC	p.Pro93Profs*8	/	/	AD	Truncation	heter	/
**AP1108**	***C19ORF12***	23269600	F038	P046	/	437	Polish	Non-EA	NM_00103172	c.357dupG	p.Ala120Glyfs*32	/	/	AD	Truncation	heter	/
**AP1109**	***C19ORF12***	23269600	F039	P047	/	474	Polish	Non-EA	NM_00103172	c.279delT	p.Ala94Profs*25	/	/	AD	Truncation	heter	/
**AP1110**	***C19ORF12***	23269600	F040	P048	/	630	Polish	Non-EA	NM_00103172	c.300delT	p.Phe100Leufs*1	/	/	AD	Truncation	heter	/
**AP1111**	***C19ORF12***	23269600	F041	P049	/	655	Polish	Non-EA	NM_00103172	c.268G>T	p.Glu90*	/	/	AD	Truncation	heter	/
**AP1112**	***C19ORF12***	23269600	F042	P050	/	661	Polish	Non-EA	NM_00103172	c.279_282del TGCC	p.Ala94Serfs*24	/	/	AD	Truncation	heter	/
**AP1113**	***C19ORF12***	23269600	F043	P051	/	663	Polish	Non-EA	NM_00103172	c.349C>T	p.Gln117*	/	/	AD	Truncation	heter	/
**AP1114**	***C19ORF12***	23269600	F044	P052	/	691-1	Polish	Non-EA	NM_00103172	c.238C>T	p.Gln80*	/	/	AD	Truncation	heter	/
**AP1115**	***C19ORF12***	23269600	F044	P053	/	691-2	Polish	Non-EA	NM_00103172	c.238C>T	p.Gln80*	/	/	AD	Truncation	heter	/
**AP1116**	***C19ORF12***	23269600	F045	P054	/	698	Polish	Non-EA	NM_00103172	c.238C>T	p.Gln80*	/	/	AD	Truncation	heter	/
**AP1117**	***C19ORF12***	23269600	F046	P055	/	748-301	Polish	Non-EA	NM_00103172	c.336G>A	p.Trp112*	/	/	AD	Truncation	heter	/
**AP1118**	***C19ORF12***	23269600	F047	P056	/	748-402	Polish	Non-EA	NM_00103172	c.336G>A	p.Trp112*	/	/	AD	Truncation	heter	/
**AP1119**	***C19ORF12***	23269600	F048	P057	/	76	Polish	Non-EA	NM_00103172	c.116C>T	p.Ser39Phe	c.205G>A	p.Gly69Arg	AR	M+M	compound I	/
**AP1120**	***C19ORF12***	23269600	F049	P058	/	160	Polish	Non-EA	NM_00103172	c.204_211del11	p.Gly69Argfs*10	c.204_211del1	p.Gly69Argfs*1	AR	T+T	homo	/
**AP1121**	***C19ORF12***	23269600	F050	P059	/	165	Polish	Non-EA	NM_00103172	c.204_211del11	p.Gly69Argfs*10	c.157G>A	p.Gly53Arg	AR	T+M	compound I	/
**AP1122**	***C19ORF12***	23269600	F051	P060	/	195	Polish	Non-EA	NM_00103172	c.248C>T	p.Pro83Leu	c.400G>C	p.Ala134Pro	AR	M+M	compound I	/
**AP1123**	***C19ORF12***	23269600	F052	P061	/	196	Polish	Non-EA	NM_00103172	c.204_211del11	p.Gly69Argfs*10	c.294G>C	p.Arg98Ser	AR	T+M	compound I	/
**AP1124**	***C19ORF12***	23269600	F053	P062	/	248	Polish	Non-EA	NM_00103172	c.204_211del11	p.Gly69Argfs*10	c.205G>A	p.Gly69Arg	AR	T+M	compound I	/
**AP1125**	***C19ORF12***	23269600	F054	P063	/	251-1	Polish	Non-EA	NM_00103172	c.199del	p.Ala67Leufs*6	c.199del	p.Ala67Leufs*i	AR	T+T	homo	/
**AP1126**	***C19ORF12***	23269600	F054	P064	/	251-2	Polish	Non-EA	NM_00103172	c.199del	p.Ala67Leufs*6	c.199del	p.Ala67Leufs*i	AR	T+T	homo	/
**AP1127**	***C19ORF12***	23269600	F055	P065	/	281-1	Polish	Non-EA	NM_00103172	c.142G>C	p.Ala48Pro	c.194-2A>G	p.?	AR	M+T	compound I	/
**AP1128**	***C19ORF12***	23269600	F055	P066	/	281-2	Polish	Non-EA	NM_00103172	c.142G>C	p.Ala48Pro	c.194-2A>G	p.?	AR	M+T	compound I	/
**AP1129**	***C19ORF12***	23269600	F056	P067	/	334	Polish	Non-EA	NM_00103172	c.157G>A	p.Gly53Arg	c.205G>A	p.Gly69Arg	AR	M+M	compound I	/
**AP1130**	***C19ORF12***	23269600	F057	P068	/	341	Polish	Non-EA	NM_00103172	c.194G>A	p.Gly65Glu	c.194G>A	p.Gly65Glu	AR	M+M	homo	/
**AP1131**	***C19ORF12***	23269600	F058	P069	/	348	Polish	Non-EA	NM_00103172	c.194G>A	p.Gly65Glu	c.179C>T	p.Pro60Leu	AR	M+M	compound I	/
**AP1132**	***C19ORF12***	23269600	F059	P070	/	379	Polish	Non-EA	NM_00103172	c.194G>A	p.Gly65Glu	c.194G>A	p.Gly65Glu	AR	M+M	homo	/
**AP1133**	***C19ORF12***	23269600	F060	P071	/	396	Polish	Non-EA	NM_00103172	c.204_211del11	p.Gly69Argfs*10	c.204_211del1	p.Gly69Argfs*1	AR	T+T	homo	/
**AP1134**	***C19ORF12***	23269600	F061	P072	/	433	Polish	Non-EA	NM_00103172	c.194G>A	p.Gly65Glu	c.400G>C	p.Ala134Pro	AR	M+M	compound I	/
**AP1135**	***C19ORF12***	23269600	F062	P073	/	438	Polish	Non-EA	NM_00103172	c.204_211del11	p.Gly69Argfs*10	c.204_211del1	p.Gly69Argfs*1	AR	T+T	homo	/
**AP1136**	***C19ORF12***	23269600	F063	P074	/	440	Polish	Non-EA	NM_00103172	c.171_181del11	p.Gly58Argfs*10	c.171_181del1	p.Gly58Argfs*1	AR	T+T	homo	/
**AP1137**	***C19ORF12***	23269600	F064	P075	/	446-1	Polish	Non-EA	NM_00103172	c.194G>A	p.Gly65Glu	c.204_211del1	p.Gly69Argfs*1	AR	M+T	compound I	/
**AP1138**	***C19ORF12***	23269600	F064	P076	/	446-2	Polish	Non-EA	NM_00103172	c.194G>A	p.Gly65Glu	c.204_211del1	p.Gly69Argfs*1	AR	M+T	compound I	/
**AP1139**	***C19ORF12***	23269600	F065	P077	/	447	Polish	Non-EA	NM_00103172	c.94delA	p.Met32fs*	c.248C>T	p.Pro83Leu	AR	T+M	compound I	/
**AP1140**	***C19ORF12***	23269600	F066	P078	/	629	Polish	Non-EA	NM_00103172	c.205G>A	p.Gly69Arg	c.205G>A	p.Gly69Arg	AR	M+M	homo	/
**AP1141**	***C19ORF12***	23436634	F067	P079	/	MPAN-1	Russian	Non-EA	NM_00103172	c.32 C>T	p.Thr11Met	c.204_214del	p.Gly69Argfs*1	AR	M+T	compound I	Female
**AP1142**	***C19ORF12***	23436634	F068	P080	/	MPAN-2	Turkish	Non-EA	NM_00103172	c.53A>G	p.Asp18Gly	c.395 T>A	p.Leu132Gln	AR	M+M	compound I	Male
**AP1143**	***C19ORF12***	23436634	F069	P081	/	MPAN-3	Polish	Non-EA	NM_00103172	c.204_214del	p.Gly69Argfs*10	c.204_214del	p.Gly69Argfs*1	AR	T+T	homo	Male
**AP1144**	***C19ORF12***	23494994	F070	P082	/	/	Russian, and Czec	Non-EA	NM_00103172	c.204_214del11	p.Gly69Argfs*10	c.205G>A	p.Gly69Arg	AR	T+M	compound I	Male
**AP1145**	***C19ORF12***	23521069	F071	P083	/		Turkish	Non-EA	NM_00103172	c.177_178insG	p.Leu60Alafs10X	c.177_178ins(	p.Leu60Alafs1	AR	T+T	homo	Male
**AP1146**	***C19ORF12***	23857908	F072	P084	/		Milan	Non-EA	NM_00103172	c.187G>C	p.Ala63Pro	c.187G>C	p.Ala63Pro	AR	M+M	homo	Female
**AP1147**	***C19ORF12***	23857908	F072	P085	/		Milanese	Non-EA	NM_00103172	c.187G>C	p.Ala63Pro	c.187G>C	p.Ala63Pro	AR	M+M	homo	Female
**AP1148**	***C19ORF12***	23857908	F073	P086	/		Malian	Non-EA	NM_00103172	c.197 199del	p.Gly66del			AR	M+	compound I	Female
**AP1149**	***C19ORF12***	23857908	F074	P087	/		Brazilian	Non-EA	NM_00103172	c.187G>C	p.Ala63Pro	c.187G>C	p.Ala63Pro	AR	M+M	homo	Female
**AP1150**	***C19ORF12***	23857908	F074	P088	/		Brazilian	Non-EA	NM_00103172	c.187G>C	p.Ala63Pro	c.187G>C	p.Ala63Pro	AR	M+M	homo	Female
**AP1151**	***C19ORF12***	24361204	F075	P089	/	patient 1	Saudi	Non-EA	NM_00103172	c.157G>A	p.Gly53Arg	c.157G>A	p.Gly53Arg	AR	M+M	homo	Male
**AP1152**	***C19ORF12***	24361204	F075	P090	/	patient 2	Saudi	Non-EA	NM_00103172	c.157G>A	p.Gly53Arg	c.157G>A	p.Gly53Arg	AR	M+M	homo	Male
**AP1153**	***C19ORF12***	24361204	F075	P091	/	patient 3	Saudi	Non-EA	NM_00103172	c.157G>A	p.Gly53Arg	c.157G>A	p.Gly53Arg	AR	M+M	homo	Male
**AP1154**	***C19ORF12***	24636776	F076	P092	/		Polish	Non-EA	NM_00103172	c.32C > T	p.T11M	c.204_214del	p.Gly69Argfs*1	AR	M+T	compound I	Female
**AP1155**	***C19ORF12***	25592411	F077	P093	/	MAPAN-1	Turkish	Non-EA	NM_00103172	c.32C > T	p.Thr11Met	c.32C > T	p.Thr11Met	AR	M+M	homo	Female
**AP1156**	***C19ORF12***	25592411	F078	P094	/	MAPAN-2	Turkish	Non-EA	NM_00103172	c.32C > T	p.Thr11Met	c.32C > T	p.Thr11Met	AR	M+M	homo	Female
**AP1157**	***C19ORF12***	25592411	F079	P095	/	MAPAN-3	German	Non-EA	NM_00103172	c.205G>A	p.Gly69Arg	c.205G>A	p.Gly69Arg	AR	M+M	homo	Female
**AP1158**	***C19ORF12***	25962551	F080	P096	First Family	C19orf12-01	Italian	Non-EA	NM_00103172	c.436_437insG	p.Ala146Glyfs*6	c.436_437ins(	p.Ala146Glyfs*	AR	T+T	homo	Female
**AP1159**	***C19ORF12***	25962551	F081	P097	Second Fami	C19orf12-02	Italian	Non-EA	NM_00103172	c.287A > C	p.Gln96Pro	c.287A>C	p.Gln96Pro	AR	M+M	homo	Male
**AP1160**	***C19ORF12***	25962551	F082	P098	Third Family	C19orf12-03	French	Non-EA	NM_00103172	c.199del	p.Ala67Leufs*6	c.416A > G	p.Tyr139Cys	AR	T+M	compound I	Male
**AP1161**	***C19ORF12***	25962551	F083	P099	Fouth Family	C19orf12-04	French	Non-EA	NM_00103172	c.244A > T	p.Lys82*	/	/	AD	Truncation	heter	Female
**AP1162**	***C19ORF12***	25962551	F084	P100	Fifth Family	C19orf12- 05	French	Non-EA	NM_00103172	c.248C > T	p.Pro83Leu	c.400G > C	p.Ala134Pro	AR	M+M	compound I	Male
**AP1163**	***C19ORF12***	26187298	F085	P101	/	Case 1	Turkish	Non-EA	NM_00103172	c.248C > T	p.Pro83Leu	c.248C > T	p.Pro83Leu	AR	M+M	homo	Female
**AP1164**	***C19ORF12***	26187298	F085	P102	/	Case 2	Turkish	Non-EA	NM_00103172	c.248C > T	p.Pro83Leu	c.248C > T	p.Pro83Leu	AR	M+M	homo	Female
**AP1165**	***C19ORF12***	28042406	F086	P103	/		Omani	Non-EA	NM_00103172	ex. 2	/	ex. 2	/	AR	CNV+CNV	homo	Female
**AP1166**	***C19ORF12***	28832565	F087	P104	SR88		Caucasian	Non-EA	NM_00103172	c.157G>A	p.Gly53Arg	c.157G>A	p.Gly53Arg	AR	M+M	heter	/
**AP1167**	***C19ORF12***	29295770	F088	P105	/		Italian	Non-EA	NM_00103172	c.265_266delAT	p.M89Gfs*12	/	/	AD	Truncation	heter	Female
**AP1168**	***C19ORF12***	29915382	F089	P106	/	21	Caucasian	Non-EA	NM_00103172	c.187G>C	p.Ala63Pro	c.194-2del	p.?	AR	M+T	compound I	Female
**AP1169**	***C19ORF12***	30088953	F090	P107	/		Czech	Non-EA	NM_00103172	c.32C>T	p.Thr11Met	c.205G>A	p.Gly69Arg	AR	M+M	compound I	Male
**AP1170**	***C19ORF12***	30298423	F091	P109	/		Turkish	Non-EA	NM_00103172	c.171_181delCGGGG	p.Gly58Argfs*10	c.171_181del(	p.Gly58Argfs*1	AR	T+T	homo	Female
**AP1171**	***C19ORF12***	31607023	F092	P110	/	II-3	Chinese	EA	NM_00103172	c.52G>T	p.Asp18Tyr	c.52G>T	p.Asp18Tyr	AR	M+M	homo	Male
**AP1172**	***C19ORF12***	31607023	F092	P111	/	II-1	Chinese	EA	NM_00103172	c.52G>T	p.Asp18Tyr	c.52G>T	p.Asp18Tyr	AR	M+M	homo	Male
**AP1173**	***C19ORF12***	31607023	F092	P112	/	II-2	Chinese	EA	NM_00103172	c.52G>T	p.Asp18Tyr	c.52G>T	p.Asp18Tyr	AR	M+M	homo	Male
**AP1174**	***C19ORF12***	31744972	F093	P113	/		India	Non-EA	NM_00103172	c.151T>G	p.Phe51Val	c.151T>G	p.Phe51Val	AR	M+M	homo	Male
**AP1175**	***C19ORF12***	33134513	F094	P114	/		Hungarian	Non-EA	NM_00103172	c.335G>A	p.Trp112Ter	/	/	AD	Truncation	heter	/
**AP1176**	***C19ORF12***	33607528	F095	P115	/	1	Russian	Non-EA	NM_00103172	c.204_214del	p.Gly69Argfs*10	c.193+5G > A	p.Pro9GlyFs*1	AR	T+T	compound I	Female
**AP1177**	***C19ORF12***	33607528	F096	P116	/	2	Russian	Non-EA	NM_00103172	c.32C > T	p.Thr11Met	c.141_142del	p. Ala48Hisfs*:	AR	M+T	compound I	Female
**AP1178**	***C19ORF12***	33607528	F097	P117	/	3	Russian	Non-EA	NM_00103172	c.204_214del	p.Gly69Argfs*10	c.204_214del	p.Gly69Argfs*1	AR	T+T	homo	Female
**AP1179**	***C19ORF12***	33607528	F098	P118	/	4	Russian	Non-EA	NM_00103172	c.204_214del	p.Gly69Argfs*10	c.204_214del	p.Gly69Argfs*1	AR	T+T	homo	Male
**AP1180**	***C19ORF12***	33607528	F099	P119	/	5	Russian	Non-EA	NM_00103172	c.204_214del	p.Gly69Argfs*10	/	/	AD	Truncation	heter	Female
**AP1181**	***C19ORF12***	33607528	F100	P120	/	6	Russian	Non-EA	NM_00103172	c.205G>A	p.Gly69Arg	c.204_214del	p.Gly69Argfs*1	AR	M+T	compound I	Male
**AP1182**	***C19ORF12***	33607528	F101	P121	/	7	Russian	Non-EA	NM_00103172	c.205G > A	p.Gly69Arg	c.204_214del	p.Gly69Argfs*1	AR	M+T	compound I	Female
**AP1183**	***C19ORF12***	33607528	F102	P122	/	8	Russian	Non-EA	NM_00103172	c.204_214del	p.Gly69Argfs*10	c.204_214del	p.Gly69Argfs*1	AR	T+T	homo	Female
**AP1184**	***C19ORF12***	33607528	F103	P123	/	9	Russian	Non-EA	NM_00103172	c.205G > A	p.Gly69Arg	c.272T > A	p.Leu91Gln	AR	M+M	compound I	Female
**AP1185**	***C19ORF12***	33607528	F104	P124	/	10	Russian	Non-EA	NM_00103172	c.204_214del	p.Gly69Argfs*10	c.248C>T	p.Pro83;eu	AR	T+M	compound I	Female
**AP1186**	***C19ORF12***	33607528	F105	P125	/	11	Russian	Non-EA	NM_00103172	c.204_214del	p.Gly69Argfs*10	c.204_214del	p.Gly69Argfs*1	AR	T+T	homo	Female
**AP1187**	***C19ORF12***	33607528	F106	P126	/	12	Russian	Non-EA	NM_00103172	c.204_214del	p.Gly69Argfs*10	c.204_214del	p.Gly69Argfs*1	AR	T+T	homo	Male
**AP1188**	***C19ORF12***	33607528	F107	P127	/	13	Russian	Non-EA	NM_00103172	c.204_214del	p.Gly69Argfs*10	c.197G>A	p.Gly66Glu	AR	T+M	compound I	Female
**AP1189**	***C19ORF12***	33607528	F108	P128	/	14	Russian	Non-EA	NM_00103172	c.204_214del	p.Gly69Argfs*10	c.204_214del	p.Gly69Argfs*1	AR	T+T	homo	Male
**AP1190**	***C19ORF12***	33607528	F109	P129	/	15	Russian	Non-EA	NM_00103172	c.204_214del	p.Gly69Argfs*10	c.204_214del	p.Gly69Argfs*1	AR	T+T	homo	Female
**AP1191**	***C19ORF12***	33607528	F110	P130	/	16	Russian	Non-EA	NM_00103172	c.204_214del	p.Gly69Argfs*10	c.204_214del	p.Gly69Argfs*1	AR	T+T	homo	Male
**AP1192**	***C19ORF12***	33607528	F111	P131	/	17	Russian	Non-EA	NM_00103172	c.204_214del	p.Gly69Argfs*10	c.204_214del	p.Gly69Argfs*1	AR	T+T	homo	Male
**AP1193**	***CISD2***	33996696/2505	F001	P001	/		Caucasian	Non-EA	NM_00100838	ex. 2		ex. 2		AR	T+T	homo	Female
**AP1194**	***CISD2***	31600780	F002	P002	/		Turkish	Non-EA	NM_00100838	entire gene		entire gene		AR	T+T	homo	Female
**AP1195**	***CISD2***	31391115	F003	P003	/	II-1	Chinese	EA	NM_00100838	c.272_273del	p.Leu91fs	c.272_273del	p.Leu91fs	AR	T+T	homo	Female
**AP1196**	***CISD2***	31391115	F003	P004	/	II-2	Chinese	EA	NM_00100838	c.272_273del	p.Leu91fs	c.272_273del	p.Leu91fs	AR	T+T	homo	Female
**AP1197**	***CISD2***	28335035	F004	P005	/	V-3	Moroccan	Non-EA	NM_00100838	c.215A > G	p.Asn72Ser	c.215A > G	p.Asn72Ser	AR	M+M	homo	Male
**AP1198**	***CISD2***	28335035	F004	P006	/	V-1	Moroccan	Non-EA	NM_00100838	c.215A > G	p.Asn72Ser	c.215A > G	p.Asn72Ser	AR	M+M	homo	Female
**AP1199**	***CISD2***	29237418/2537	F005	P007	/		Italian	Non-EA	NM_00100838	c.103+1G>A	p.?	c.103+1G>A	p.?	AR	T+T	homo	Female
**AP1200**	***CISD2***	29237418/2537	F006	P008	/		Italian	Non-EA	NM_00100838	c.103+1G>A	p.?	c.103+1G>A	p.?	AR	T+T	homo	Female
**AP1201**	***CISD2***	17846994	F007	P009	WS2		Jordanian	Non-EA	NM_00100838	c.109G > C	p.Glu37Gln	c.109G > C	p.Glu37Gln	AR	M+M	homo	Male
**AP1202**	***CISD2***	17846994	F008	P010	WS3		Jordanian	Non-EA	NM_00100838	c.109G > C	p.Glu37Gln	c.109G > C	p.Glu37Gln	AR	M+M	homo	Female
**AP1203**	***CISD2***	17846994	F009	P011	WS4		Jordanian	Non-EA	NM_00100838	c.109G > C	p.Glu37Gln	c.109G > C	p.Glu37Gln	AR	M+M	homo	Female
**AP1204**	***DNAJC19***	16055927	F001	P001	1	6	Caucasian	Non-EA	NM_00119023	c.130-1G>C	p.?	c.130-1G>C	p.?	AR	T+T	homo	Male
**AP1205**	***DNAJC19***	16055927	F001	P002	1	1	Caucasian	Non-EA	NM_00119023	c.130-1G>C	p.?	c.130-1G>C	p.?	AR	T+T	homo	Male
**AP1206**	***DNAJC19***	16055927	F001	P003	1	5	Caucasian	Non-EA	NM_00119023	c.130-1G>C	p.?	c.130-1G>C	p.?	AR	T+T	homo	Male
**AP1207**	***DNAJC19***	16055927	F002	P004	2	22	Caucasian	Non-EA	NM_00119023	c.130-1G>C	p.?	c.130-1G>C	p.?	AR	T+T	homo	Female
**AP1208**	***DNAJC19***	16055927	F002	P005	2	21	Caucasian	Non-EA	NM_00119023	c.130-1G>C	p.?	c.130-1G>C	p.?	AR	T+T	homo	Female
**AP1209**	***DNAJC19***	16055927	F003	P006	3	7	Caucasian	Non-EA	NM_00119023	c.130-1G>C	p.?	c.130-1G>C	p.?	AR	T+T	homo	Male
**AP1210**	***DNAJC19***	16055927	F003	P007	3	8	Caucasian	Non-EA	NM_00119023	c.130-1G>C	p.?	c.130-1G>C	p.?	AR	T+T	homo	Male
**AP1211**	***DNAJC19***	16055927	F004	P008	4	19	Caucasian	Non-EA	NM_00119023	c.130-1G>C	p.?	c.130-1G>C	p.?	AR	T+T	homo	Male
**AP1212**	***DNAJC19***	16055927	F004	P009	4	16	Caucasian	Non-EA	NM_00119023	c.130-1G>C	p.?	c.130-1G>C	p.?	AR	T+T	homo	Male
**AP1213**	***DNAJC19***	16055927	F004	P010	4	20	Caucasian	Non-EA	NM_00119023	c.130-1G>C	p.?	c.130-1G>C	p.?	AR	T+T	homo	Female
**AP1214**	***DNAJC19***	16055927	F005	P011	5	10	Caucasian	Non-EA	NM_00119023	c.130-1G>C	p.?	c.130-1G>C	p.?	AR	T+T	homo	Male
**AP1215**	***DNAJC19***	16055927	F006	P012	6	23	Caucasian	Non-EA	NM_00119023	c.130-1G>C	p.?	c.130-1G>C	p.?	AR	T+T	homo	Female
**AP1216**	***DNAJC19***	16055927	F007	P013	7	11	Caucasian	Non-EA	NM_00119023	c.130-1G>C	p.?	c.130-1G>C	p.?	AR	T+T	homo	Male
**AP1217**	***DNAJC19***	16055927	F008	P014	8	9	Caucasian	Non-EA	NM_00119023	c.130-1G>C	p.?	c.130-1G>C	p.?	AR	T+T	homo	Female
**AP1218**	***DNAJC19***	16055927	F009	P015	9	24	Caucasian	Non-EA	NM_00119023	c.130-1G>C	p.?	c.130-1G>C	p.?	AR	T+T	homo	Female
**AP1219**	***DNAJC19***	16055927	F010	P016	10	12	Caucasian	Non-EA	NM_00119023	c.130-1G>C	p.?	c.130-1G>C	p.?	AR	T+T	homo	Male
**AP1220**	***DNAJC19***	16055927	F010	P017	10	13	Caucasian	Non-EA	NM_00119023	c.130-1G>C	p.?	c.130-1G>C	p.?	AR	T+T	homo	Female
**AP1221**	***DNAJC19***	16055927	F011	P018	11	25	Caucasian	Non-EA	NM_00119023	c.130-1G>C	p.?	c.130-1G>C	p.?	AR	T+T	homo	Male
**AP1222**	***DNAJC19***	22797137	F012	P019	/	patient 1	Finnish	Non-EA	NM_00119023	c.300delA	p.Ala100fsX11	c.300delA	p.Ala100fsX11	AR	T+T	homo	Male
**AP1223**	***DNAJC19***	22797137	F012	P020	/	patient 2	Finnish	Non-EA	NM_00119023	c.300delA	p.Ala100fsX11	c.300delA	p.Ala100fsX11	AR	T+T	homo	Male
**AP1224**	***DNAJC19***	22981120	F013	P021	/		American	Non-EA	NM_00119023	c.130-1G>C	p.?	c.130-1G>C	p.?	AR	T+T	homo	Male
**AP1225**	***DNAJC19***	27426421	F014	P022	/		Turkish	Non-EA	NM_00119023	c.280+1_280+5delGTA	p.?	c.280+1_280+	p.?	AR	T+T	homo	/
**AP1226**	***DNAJC19***	27928788	F015	P023	/		Turkish	Non-EA	NM_00119023	c.63delC	p.Tyr21*	c.63delC	p.Tyr21*	AR	T+T	homo	Female
**AP1227**	***DNAJC19***	28771251	F016	P024	/		Canadian	Non-EA	NM_00119023	c.280+1_280+5delGTA	p.?	c.280+1_280+	p.?	AR	T+T	homo	Female
**AP1228**	***DNAJC19***	29625556	F017	P025	/		French	Non-EA	NM_00119023	c.51delT	p.Phe17Leufs*10	c.51delT	p.Phe17Leufs*	AR	T+T	homo	Male
**AP1229**	***DNAJC30***	33465056	F001	P001	Family 1	1	German	Non-EA	NM_032317.3	c.152A>G	p.Tyr51Cys	c.152A>G	p.Tyr51Cys	AR	M+M	homozygou	Female
**AP1230**	***DNAJC30***	33465056	F002	P002	Family 2	2	Luxemburger	Non-EA	NM_032317.3	c.152A>G	p.Tyr51Cys	c.152A>G	p.Tyr51Cys	AR	M+M	homozygou	Male
**AP1231**	***DNAJC30***	33465056	F003	P003	Family 3	3-1	Polish	Non-EA	NM_032317.3	c.152A>G	p.Tyr51Cys	c.152A>G	p.Tyr51Cys	AR	M+M	homozygou	Male
**AP1232**	***DNAJC30***	33465056	F003	P004	Family 3	3-2	Polish	Non-EA	NM_032317.3	c.152A>G	p.Tyr51Cys	c.152A>G	p.Tyr51Cys	AR	M+M	homozygou	Male
**AP1233**	***DNAJC30***	33465056	F004	P005	Family 4	4	Romanian	Non-EA	NM_032317.3	c.152A>G	p.Tyr51Cys	c.152A>G	p.Tyr51Cys	AR	M+M	homozygou	Male
**AP1234**	***DNAJC30***	33465056	F005	P006	Family 5	5	Russian	Non-EA	NM_032317.3	c.152A>G	p.Tyr51Cys	c.152A>G	p.Tyr51Cys	AR	M+M	homozygou	Male
**AP1235**	***DNAJC30***	33465056	F006	P007	Family 6	6	Russian	Non-EA	NM_032317.3	c.152A>G	p.Tyr51Cys	c.152A>G	p.Tyr51Cys	AR	M+M	homozygou	Male
**AP1236**	***DNAJC30***	33465056	F007	P008	Family 7	7	Ukrainer	Non-EA	NM_032317.3	c.152A>G	p.Tyr51Cys	c.152A>G	p.Tyr51Cys	AR	M+M	homozygou	Male
**AP1237**	***DNAJC30***	33465056	F008	P009	Family 8	8	Russian	Non-EA	NM_032317.3	c.152A>G	p.Tyr51Cys	c.152A>G	p.Tyr51Cys	AR	M+M	homozygou	Male
**AP1238**	***DNAJC30***	33465056	F009	P010	Family 9	9	Russian	Non-EA	NM_032317.3	c.152A>G	p.Tyr51Cys	c.152A>G	p.Tyr51Cys	AR	M+M	homozygou	Male
**AP1239**	***DNAJC30***	33465056	F010	P011	Family 10	10	Russian	Non-EA	NM_032317.3	c.152A>G	p.Tyr51Cys	c.152A>G	p.Tyr51Cys	AR	M+M	homozygou	Male
**AP1240**	***DNAJC30***	33465056	F011	P012	Family 11	11	Russian	Non-EA	NM_032317.3	c.152A>G	p.Tyr51Cys	c.152A>G	p.Tyr51Cys	AR	M+M	homozygou	Male
**AP1241**	***DNAJC30***	33465056	F012	P013	Family 12	12	Russian	Non-EA	NM_032317.3	c.152A>G	p.Tyr51Cys	c.152A>G	p.Tyr51Cys	AR	M+M	homozygou	Male
**AP1242**	***DNAJC30***	33465056	F013	P014	Family 13	13	Russian	Non-EA	NM_032317.3	c.152A>G	p.Tyr51Cys	c.152A>G	p.Tyr51Cys	AR	M+M	homozygou	Male
**AP1243**	***DNAJC30***	33465056	F014	P015	Family 14	14	Russian	Non-EA	NM_032317.3	c.152A>G	p.Tyr51Cys	c.152A>G	p.Tyr51Cys	AR	M+M	homozygou	Male
**AP1244**	***DNAJC30***	33465056	F015	P016	Family 15	15-1	Russian	Non-EA	NM_032317.3	c.152A>G	p.Tyr51Cys	c.152A>G	p.Tyr51Cys	AR	M+M	homozygou	Male
**AP1245**	***DNAJC30***	33465056	F015	P017	Family 15	15-2	Russian	Non-EA	NM_032317.3	c.152A>G	p.Tyr51Cys	c.152A>G	p.Tyr51Cys	AR	M+M	homozygou	Female
**AP1246**	***DNAJC30***	33465056	F016	P018	Family 16	16	Russian	Non-EA	NM_032317.3	c.152A>G	p.Tyr51Cys	c.152A>G	p.Tyr51Cys	AR	M+M	homozygou	Female
**AP1247**	***DNAJC30***	33465056	F017	P019	Family 17	17	Russian	Non-EA	NM_032317.3	c.152A>G	p.Tyr51Cys	c.152A>G	p.Tyr51Cys	AR	M+M	homozygou	Male
**AP1248**	***DNAJC30***	33465056	F018	P020	Family 18	18-1	Tunisia	Non-EA	NM_032317.3	c.232C>T	p.Pro78Ser	c.232C>T	p.Pro78Ser	AR	M+M	homozygou	Male
**AP1249**	***DNAJC30***	33465056	F018	P021	Family 18	18-2	Tunisia	Non-EA	NM_032317.3	c.232C>T	p.Pro78Ser	c.232C>T	p.Pro78Ser	AR	M+M	homozygou	Male
**AP1250**	***DNAJC30***	33465056	F019	P022	Family 19	19	Canadian	Non-EA	NM_032317.3	c.152A>G	p.Tyr51Cys	c.152A>G	p.Tyr51Cys	AR	M+M	homozygou	Male
**AP1251**	***DNAJC30***	33465056	F020	P023	Family 20	20-1	Turkish	Non-EA	NM_032317.3	c.302T>A	p.Leu101Gln	c.302T>A	p.Leu101Gln	AR	M+M	homozygou	Male
**AP1252**	***DNAJC30***	33465056	F020	P024	Family 20	20-2	Russian	Non-EA	NM_032317.3	c.302T>A	p.Leu101Gln	c.302T>A	p.Leu101Gln	AR	M+M	homozygou	Male
**AP1253**	***DNAJC30***	33465056	F021	P025	Family 21	21	Russian	Non-EA	NM_032317.3	c.152A>G	p.Tyr51Cys	c.152A>G	p.Tyr51Cys	AR	M+M	homozygou	Male
**AP1254**	***DNAJC30***	33465056	F022	P026	Family 22	22	Russian	Non-EA	NM_032317.3	c.152A>G	p.Tyr51Cys	c.152A>G	p.Tyr51Cys	AR	M+M	homozygou	Male
**AP1255**	***DNAJC30***	33465056	F023	P027	Family 23	23	Russian	Non-EA	NM_032317.3	c.152A>G	p.Tyr51Cys	c.152A>G	p.Tyr51Cys	AR	M+M	homozygou	Male
**AP1256**	***DNAJC30***	33465056	F024	P028	Family 24	24	Russian	Non-EA	NM_032317.3	c.152A>G	p.Tyr51Cys	c.152A>G	p.Tyr51Cys	AR	M+M	homozygou	Male
**AP1257**	***DNAJC30***	33465056	F025	P029	Family 25	25	Russian	Non-EA	NM_032317.3	c.152A>G	p.Tyr51Cys	c.152A>G	p.Tyr51Cys	AR	M+M	homozygou	Male
**AP1258**	***DNAJC30***	33465056	F026	P030	Family 26	26	Russian	Non-EA	NM_032317.3	c.152A>G	p.Tyr51Cys	c.152A>G	p.Tyr51Cys	AR	M+M	homozygou	Male
**AP1259**	***DNAJC30***	33465056	F027	P031	Family 27	27	Russian	Non-EA	NM_032317.3	c.152A>G	p.Tyr51Cys	c.152A>G	p.Tyr51Cys	AR	M+M	homozygou	Male
**AP1260**	***DNAJC30***	33465056	F028	P032	Family 28	28	Ukrainer	Non-EA	NM_032317.3	c.152A>G	p.Tyr51Cys	c.152A>G	p.Tyr51Cys	AR	M+M	homozygou	Male
**AP1261**	***DNAJC30***	33465056	F029	P033	Family 29	29	Ukrainer	Non-EA	NM_032317.3	c.152A>G	p.Tyr51Cys	c.152A>G	p.Tyr51Cys	AR	M+M	homozygou	Male
**AP1262**	***DNAJC30***	35091433	F030	P034	/	LHON 59(1316	Central European	Non-EA	NM_032317.3	c.152A>G	p.Tyr51Cys	c.152A>G	p.Tyr51Cys	AR	M+M	homozygou	Male
**AP1263**	***DNAJC30***	35091433	F030	P035	/	LHON 59(1824	German	Non-EA	NM_032317.3	c.152A>G	p.Tyr51Cys	c.152A>G	p.Tyr51Cys	AR	M+M	homozygou	Male
**AP1264**	***DNAJC30***	35091433	F031	P036	/	LHON 84	Eastern European	Non-EA	NM_032317.3	c.152A>G	p.Tyr51Cys	c.152A>G	p.Tyr51Cys	AR	M+M	homozygou	Male
**AP1265**	***DNAJC30***	35091433	F032	P037	/	LHON 96	Caucasian	Non-EA	NM_032317.3	c.152A>G	p.Tyr51Cys	c.610G>T;	p.Glu204*	AR	M+T	Compd het	Male
**AP1266**	***DNAJC30***	35091433	F033	P038	/	LHON 210	Central European	Non-EA	NM_032317.3	c.152A>G	p.Tyr51Cys	c.152A>G	p.Tyr51Cys	AR	M+M	homozygou	Male
**AP1267**	***DNAJC30***	35091433	F034	P039	/	LHON 238	Central European	Non-EA	NM_032317.3	c.152A>G	p.Tyr51Cys	c.152A>G	p.Tyr51Cys	AR	M+M	homozygou	Female
**AP1268**	***DNAJC30***	35091433	F035	P040	/	LHON 246	Caucasian	Non-EA	NM_032317.3	c.152A>G	p.Tyr51Cys	c.152A>G	p.Tyr51Cys	AR	M+M	homozygou	Male
**AP1269**	***DNAJC30***	35091433	F036	P041	/	LHON 286	Caucasian	Non-EA	NM_032317.3	c.152A>G	p.Tyr51Cys	c.152A>G	p.Tyr51Cys	AR	M+M	homozygou	Female
**AP1270**	***DNAJC30***	35091433	F037	P042	/	LHON 347	Caucasian	Non-EA	NM_032317.3	c.152A>G	p.Tyr51Cys	c.152A>G	p.Tyr51Cys	AR	M+M	homozygou	Male
**AP1271**	***DNAJC30***	35091433	F038	P043	/	LHON 377	Caucasian	Non-EA	NM_032317.3	c.152A>G	p.Tyr51Cys	c.152A>G	p.Tyr51Cys	AR	M+M	homozygou	Male
**AP1272**	***DNAJC30***	35091433	F039	P044	/	LHON 380	Arabian	Non-EA	NM_032317.3	c.152A>G	p.Tyr51Cys	c.152A>G	p.Tyr51Cys	AR	M+M	homozygou	Male
**AP1273**	***DNAJC30***	35091433	F040	P045	/	LHON 466	Central European	Non-EA	NM_032317.3	c.152A>G	p.Tyr51Cys	c.152A>G	p.Tyr51Cys	AR	M+M	homozygou	Male
**AP1274**	***DNAJC30***	35091433	F041	P046	/	LHON 507	Central European	Non-EA	NM_032317.3	c.152A>G	p.Tyr51Cys	c.152A>G	p.Tyr51Cys	AR	M+M	homozygou	Female
**AP1275**	***DNAJC30***	35091433	F042	P047	/	LHON 526	Central European	Non-EA	NM_032317.3	c.152A>G	p.Tyr51Cys	c.152A>G	p.Tyr51Cys	AR	M+M	homozygou	Male
**AP1276**	***DNAJC30***	35091433	F043	P048	/	LHON 573	Central European	Non-EA	NM_032317.3	c.152A>G	p.Tyr51Cys	c.610G>T;	p.Glu204*	AR	M+T	Compd het	Male
**AP1277**	***DNAJC30***	35091433	F044	P049	/	LHON 582	Central European	Non-EA	NM_032317.3	c.152A>G	p.Tyr51Cys	c.152A>G	p.Tyr51Cys	AR	M+M	homozygou	Male
**AP1278**	***DNAJC30***	35091433	F045	P050	/	LHON 600	Central European	Non-EA	NM_032317.3	c.152A>G	p.Tyr51Cys	c.152A>G	p.Tyr51Cys	AR	M+M	homozygou	Male
**AP1279**	***DNAJC30***	35091433	F046	P051	/	LHON 606	Central European	Non-EA	NM_032317.3	c.152A>G	p.Tyr51Cys	c.152A>G	p.Tyr51Cys	AR	M+M	homozygou	Male
**AP1280**	***DNAJC30***	35091433	F047	P052	/	LHON 612	Central European	Non-EA	NM_032317.3	c.152A>G	p.Tyr51Cys	c.152A>G	p.Tyr51Cys	AR	M+M	homozygou	Male
**AP1281**	***DNAJC30***	35091433	F048	P053	/	LHON 749(120	Central European	Non-EA	NM_032317.3	c.152A>G	p.Tyr51Cys	c.152A>G	p.Tyr51Cys	AR	M+M	homozygou	Male
**AP1282**	***DNAJC30***	35091433	F048	P054	/	LHON 749(145	Central European	Non-EA	NM_032317.3	c.152A>G	p.Tyr51Cys	c.152A>G	p.Tyr51Cys	AR	M+M	homozygou	Male
**AP1283**	***DNAJC30***	35091433	F049	P055	/	LHON 760	Central European	Non-EA	NM_032317.3	c.152A>G	p.Tyr51Cys	c.152A>G	p.Tyr51Cys	AR	M+M	homozygou	Male
**AP1284**	***DNAJC30***	35091433	F050	P056	/	LHON 785	Central European	Non-EA	NM_032317.3	c.152A>G	p.Tyr51Cys	c.152A>G	p.Tyr51Cys	AR	M+M	homozygou	Female
**AP1285**	***DNAJC30***	35091433	F051	P057	/	LHON 895	Central European	Non-EA	NM_032317.3	c.152A>G	p.Tyr51Cys	c.152A>G	p.Tyr51Cys	AR	M+M	homozygou	Male
**AP1286**	***DNAJC30***	35091433	F052	P058	/	LHON 1076	Central European	Non-EA	NM_032317.3	c.152A>G	p.Tyr51Cys	c.152A>G	p.Tyr51Cys	AR	M+M	homozygou	Male
**AP1287**	***DNAJC30***	35091433	F053	P059	/	LHON 1088	Central European	Non-EA	NM_032317.3	c.152A>G	p.Tyr51Cys	c.152A>G	p.Tyr51Cys	AR	M+M	homozygou	Male
**AP1288**	***DNAJC30***	35091433	F054	P060	/	LHON 1089	Central European	Non-EA	NM_032317.3	c.152A>G	p.Tyr51Cys	c.152A>G	p.Tyr51Cys	AR	M+M	homozygou	Female
**AP1289**	***DNAJC30***	35091433	F055	P061	/	LHON 1129	Central European	Non-EA	NM_032317.3	c.152A>G	p.Tyr51Cys	c.152A>G	p.Tyr51Cys	AR	M+M	homozygou	Male
**AP1290**	***DNAJC30***	35091433	F056	P062	/	LHON 1149	Central European	Non-EA	NM_032317.3	c.152A>G	p.Tyr51Cys	c.610G>T;	p.Glu204*	AR	M+T	Compd het	Male
**AP1291**	***DNAJC30***	35091433	F057	P063	/	OAK 317	Central European	Non-EA	NM_032317.3	c.152A>G	p.Tyr51Cys	c.152A>G	p.Tyr51Cys	AR	M+M	homozygou	Male
**AP1292**	***DNAJC30***	35091433	F058	P064	/	OAK 559(1977	Turkish	Non-EA	NM_032317.3	c.230_232del;	p.His77del	c.230_232del;	p.His77del	AR	T+T	homozygou	Male
**AP1293**	***DNAJC30***	35091433	F058	P065	/	OAK 559(1977	Turkish	Non-EA	NM_032317.3	c.230_232del;	p.His77del	c.230_232del;	p.His77del	AR	T+T	homozygou	Male
**AP1294**	***DNAJC30***	35091433	F059	P066	/	OAK 627	Central European	Non-EA	NM_032317.3	c.152A>G	p.Tyr51Cys	c.152A>G	p.Tyr51Cys	AR	M+M	homozygou	Male
**AP1295**	***DNAJC30***	35091433	F060	P067	/	OAK715	Central European	Non-EA	NM_032317.3	c.152A>G	p.Tyr51Cys	c.152A>G	p.Tyr51Cys	AR	M+M	homozygou	Male
**AP1296**	***DNAJC30***	35091433	F061	P068	/	OAK767	Central European	Non-EA	NM_032317.3	c.152A>G	p.Tyr51Cys	c.152A>G	p.Tyr51Cys	AR	M+M	homozygou	Male
**AP1297**	***DNAJC30***	35148383	F062	P069	1	patient 1	Czech	Non-EA	NM_032317.3	c.152A>G	p.Tyr51Cys	c.152A>G	p.Tyr51Cys	AR	M+M	homozygou	Female
**AP1298**	***DNAJC30***	35148383	F063	P070	2	patient 2	Czech	Non-EA	NM_032317.3	c.152A>G	p.Tyr51Cys	c.152A>G	p.Tyr51Cys	AR	M+M	homozygou	Female
**AP1299**	***DNAJC30***	35148383	F064	P071	3	patient 3	Czech	Non-EA	NM_032317.3	c.152A>G	p.Tyr51Cys	c.152A>G	p.Tyr51Cys	AR	M+M	homozygou	Male
**AP1300**	***DNAJC30***	35148383	F065	P072	4		Czech	Non-EA	NM_032317.3	c.152A>G	p.Tyr51Cys	c.152A>G	p.Tyr51Cys	AR	M+M	homozygou	/
**AP1301**	***DNAJC30***	35148383	F066	P073	5		Czech	Non-EA	NM_032317.3	c.152A>G	p.Tyr51Cys	c.152A>G	p.Tyr51Cys	AR	M+M	homozygou	/
**AP1302**	***DNAJC30***	35148383	F067	P074	6		Czech	Non-EA	NM_032317.3	c.152A>G	p.Tyr51Cys	c.152A>G	p.Tyr51Cys	AR	M+M	homozygou	/
**AP1303**	***DNAJC30***	35148383	F068	P075	7		Czech	Non-EA	NM_032317.3	c.152A>G	p.Tyr51Cys	c.152A>G	p.Tyr51Cys	AR	M+M	homozygou	/
**AP1304**	***DNAJC30***	35148383	F069	P076	8		Czech	Non-EA	NM_032317.3	c.152A>G	p.Tyr51Cys	c.152A>G	p.Tyr51Cys	AR	M+M	homozygou	/
**AP1305**	***DNAJC30***	35148383	F070	P077	9		Czech	Non-EA	NM_032317.3	c.152A>G	p.Tyr51Cys	c.152A>G	p.Tyr51Cys	AR	M+M	homozygou	/
**AP1306**	***DNAJC30***	35148383	F071	P078	10		Czech	Non-EA	NM_032317.3	c.152A>G	p.Tyr51Cys	c.152A>G	p.Tyr51Cys	AR	M+M	homozygou	/
**AP1307**	***DNAJC30***	35148383	F072	P079	11		Czech	Non-EA	NM_032317.3	c.152A>G	p.Tyr51Cys	c.152A>G	p.Tyr51Cys	AR	M+M	homozygou	/
**AP1308**	***DNAJC30***	35148383	F073	P080	12		Czech	Non-EA	NM_032317.3	c.152A>G	p.Tyr51Cys	c.152A>G	p.Tyr51Cys	AR	M+M	homozygou	/
**AP1309**	***DNAJC30***	35148383	F074	P081	13		Czech	Non-EA	NM_032317.3	c.152A>G	p.Tyr51Cys	c.152A>G	p.Tyr51Cys	AR	M+M	homozygou	/
**AP1310**	***DNAJC30***	35148383	F075	P082	14		Czech	Non-EA	NM_032317.3	c.152A>G	p.Tyr51Cys	c.152A>G	p.Tyr51Cys	AR	M+M	homozygou	/
**AP1311**	***DNAJC30***	35148383	F076	P083	15		Swedish	Non-EA	NM_032317.3	c.152A>G	p.Tyr51Cys	c.152A>G	p.Tyr51Cys	AR	M+M	homozygou	/
**AP1312**	***DNAJC30***	35148383	F077	P084	16		Russian	Non-EA	NM_032317.3	c.152A>G	p.Tyr51Cys	c.152A>G	p.Tyr51Cys	AR	M+M	homozygou	/
**AP1313**	***DNAJC30***	35148383	F078	P085	17		Russian	Non-EA	NM_032317.3	c.152A>G	p.Tyr51Cys	c.152A>G	p.Tyr51Cys	AR	M+M	homozygou	/
**AP1314**	***DNAJC30***	35148383	F079	P086	18		Russian	Non-EA	NM_032317.3	c.152A>G	p.Tyr51Cys	c.152A>G	p.Tyr51Cys	AR	M+M	homozygou	/
**AP1315**	***DNAJC30***	35148383	F080	P087	19		Russian	Non-EA	NM_032317.3	c.152A>G	p.Tyr51Cys	c.152A>G	p.Tyr51Cys	AR	M+M	homozygou	/
**AP1316**	***DNAJC30***	35148383	F081	P088	20		Russian	Non-EA	NM_032317.3	c.152A>G	p.Tyr51Cys	c.152A>G	p.Tyr51Cys	AR	M+M	homozygou	/
**AP1317**	***DNAJC30***	35148383	F082	P089	21		Russian	Non-EA	NM_032317.3	c.152A>G	p.Tyr51Cys	c.152A>G	p.Tyr51Cys	AR	M+M	homozygou	/
**AP1318**	***DNAJC30***	35148383	F083	P090	22		American	Non-EA	NM_032317.3	c.152A>G	p.Tyr51Cys	c.152A>G	p.Tyr51Cys	AR	M+M	homozygou	/
**AP1319**	***DNAJC30***	35148383	F084	P091	23		American	Non-EA	NM_032317.3	c.152A>G	p.Tyr51Cys	c.152A>G	p.Tyr51Cys	AR	M+M	homozygou	/
**AP1320**	***DNAJC30***	35148383	F085	P092	24		Romanian	Non-EA	NM_032317.3	c.152A>G	p.Tyr51Cys	c.152A>G	p.Tyr51Cys	AR	M+M	homozygou	/
**AP1321**	***DNAJC30***	35148383	F086	P093	25		Polish	Non-EA	NM_032317.3	c.152A>G	p.Tyr51Cys	c.152A>G	p.Tyr51Cys	AR	M+M	homozygou	/
**AP1322**	***DNAJC30***	35148383	F087	P094	26		Turkish	Non-EA	NM_032317.3	c.152A>G	p.Tyr51Cys	c.152A>G	p.Tyr51Cys	AR	M+M	homozygou	/
**AP1323**	***DNAJC30***	35148383	F088	P095	/	/	Swedish	Non-EA	NM_032317.3	c.152A>G	p.Tyr51Cys	c.152A>G	p.Tyr51Cys	AR	M+M	homozygou	/
**AP1324**	***DNM1L***	17460227	F001	P001	/	patient1	Caucasian	Non-EA	NM_012062.4	c.1184C>A	p.Ala395>Asp	/	/	AD	M	heter	Female
**AP1325**	***DNM1L***	26604000	F002	P002	/		Caucasian	Non-EA	NM_012062.4	c.1085G>A	p.Gly362Ser	/	/	AD	M	heter	Male
**AP1326**	***DNM1L***	26825290	F003	P003	/	patient A	Filipino	Non-EA	NM_012062.4	c.261dup	p.Trp88Metfs*	c.385_386de	p.Glu129Lys*6	AR	T+T	compound I	Female
**AP1327**	***DNM1L***	26825290	F003	P004	/	patient B	Filipino	Non-EA	NM_012062.4	c.261dup	p.Trp88Metfs*	c.385_386de	p.Glu129Lys*6	AR	T+T	compound I	Male
**AP1328**	***DNM1L***	26931468	F004	P005	/		Caucasian	Non-EA	NM_012062.4	c.1048G>A	p.Gly350Arg	/	/	AD	M	heter	Male
**AP1329**	***DNM1L***	26992161	F005	P006	/	1	Moslem Arab	Non-EA	NM_012062.4	c.1084G>A	p.Gly362Ser	/	/	AD	M	heter	Male
**AP1330**	***DNM1L***	27145208	F006	P007	/	Proband 1	Caucasian	Non-EA	NM_012062.4	c.1207C>T	p.Arg403Cys	/	/	AD	M	heter	Male
**AP1331**	***DNM1L***	27145208	F007	P008	/	Proband 2	Caucasian	Non-EA	NM_012062.4	c.1207C>T	p.Arg403Cys	/	/	AD	M	heter	Male
**AP1332**	***DNM1L***	27301154	F008	P009	/	patient	Japanese	EA	NM_012062.4	c.1217T>C	p.Leu406Ser	/	/	AD	M	heter	Male
**AP1333**	***DNM1L***	27328748	F009	P010	/	II-2	Caucasian	Non-EA	NM_012062.4	c.346_347delGA	p.Glu116Lysfs*6	c.106A>G	p.Ser36Gly	AR	T+M	compound I	Male
**AP1334**	***DNM1L***	27328748	F009	P011	/	II-3	Caucasian	Non-EA	NM_012062.4	c.346_347delGA	p.Glu116Lysfs*6	c.106A>G	p.Ser36Gly	AR	T+M	compound I	Male
**AP1335**	***DNM1L***	28969390	F010	P012	Family 3	/	French	Non-EA	NM_012062.4	c.575C>A	p.Ala192Glu	/	/	AD	M	heter	Male
**AP1336**	***DNM1L***	28969390	F011	P013	Family 2	/	French	Non-EA	NM_012062.4	c.5A>C	p.Glu2Ala	/	/	AD	M	heter	Male
**AP1337**	***DNM1L***	28969390	F012	P014	Family 1	II:5	French	Non-EA	NM_012062.4	c.5A>C	p.Glu2Ala	/	/	AD	M	heter	Female
**AP1338**	***DNM1L***	28969390	F012	P015	Family 1	II:6	French	Non-EA	NM_012062.4	c.5A>C	p.Glu2Ala	/	/	AD	M	heter	Female
**AP1339**	***DNM1L***	28969390	F012	P016	Family 1	IV:4	French	Non-EA	NM_012062.4	c.5A>C	p.Glu2Ala	/	/	AD	M	heter	Female
**AP1340**	***DNM1L***	28969390	F012	P017	Family 1	IV:6	French	Non-EA	NM_012062.4	c.5A>C	p.Glu2Ala	/	/	AD	M	heter	Female
**AP1341**	***DNM1L***	29110115	F013	P018	/	patient	Canadian	Non-EA	NM_012062.4	c.305C>T	p.Tht115Met	c.305C>T	p.Tht115Met	AR	M	homo	Female
**AP1342**	***DNM1L***	29877124	F014	P019	/	patient	Caucasian	Non-EA	NM_012062.4	c.1207C>T	p.Arg403Cys	/	/	AD	M	heter	/
**AP1343**	***DNM1L***	30085106	F015	P020	/	patient 1	Caucasian	Non-EA	NM_012062.4	c.1292 G>A	p.Cys431Tyr	/	/	AD	M	heter	Female
**AP1344**	***DNM1L***	30085106	F016	P021	/	patient 2	Caucasian	Non-EA	NM_012062.4	c.1207C>T	p.Arg403Cys	/	/	AD	M	heter	Female
**AP1345**	***DNM1L***	30085106	F017	P022	/	patient 3	Caucasian	Non-EA	NM_012062.4	c.1207C>T	p.Arg403Cys	/	/	AD	M	heter	Male
**AP1346**	***DNM1L***	30085106	F018	P023	/	patient 4	Caucasian	Non-EA	NM_012062.4	c.95G>C	p.Glu32Ala	/	/	AD	M	heter	Female
**AP1347**	***DNM1L***	30085106	F019	P024	/	patient 5	Caucasian	Non-EA	NM_012062.4	c.1184C>G	p.Ala395Gly	/	/	AD	M	heter	Female
**AP1348**	***DNM1L***	30109270	F020	P025	/	case 1	Caucasian	Non-EA	NM_012062.4	c.1207C>T	p.Arg403Cys	/	/	AD	M	heter	/
**AP1349**	***DNM1L***	30109270	F021	P026	/	case 2	Caucasian	Non-EA	NM_012062.4	c.1207C>T	p.Arg403Cys	/	/	AD	M	heter	/
**AP1350**	***DNM1L***	30801875	F022	P027	/	Pt1	Italian	Non-EA	NM_012062.4	c.668G>T	p.Gly223Val	/	/	AD	M	heter	Female
**AP1351**	***DNM1L***	30801875	F023	P028	/	Pt2	Italian	Non-EA	NM_012062.4	c.1207C>T	p.Arg403Cys	/	/	AD	M	heter	Male
**AP1352**	***DNM1L***	30801875	F024	P029	/	Pt3	Italian	Non-EA	NM_012062.4	c.1109T>G	p.Phe370Cys	/	/	AD	M	heter	Female
**AP1353**	***DNM1L***	30801875	F025	P030	/	Pt4	Italian	Non-EA	NM_012062.4	c.1085G>A	p.Gly362Ser	c.1535T>C	p.Ile512Thr	AR	M	compound I	Male
**AP1354**	***DNM1L***	30801875	F026	P031	/	Pt5	Italian	Non-EA	NM_012062.4	c.1084G>A	p.Gly362Ser	/	/	AD	M	heter	Female
**AP1355**	***DNM1L***	30850373	F027	P032	/		Caucasian	Non-EA	NM_012062.4	c.2072A>G	p.Tyr691Cys	/	/	AD	M	heter	Female
**AP1356**	***DNM1L***	30952489	F028	P033	/	P13	Korean	Non-EA	NM_012062.4	c.1949T>G	p.Leu650Arg	/	/	AD	M	heter	/
**AP1357**	***DNM1L***	30952489	F029	P034	/	P14	Korean	Non-EA	NM_012062.4	c.1247T>C	p.Leu416Pro	/	/	AD	M	heter	/
**AP1358**	***DNM1L***	31587467	F030	P035	/		Caucasian	Non-EA	NM_012062.4	c.1228G>A	p.Glu410Lys	/	/	AD	M	heter	Female
**AP1359**	***DNM1L***	31868880	F031	P036	/	/	Caucasian	Non-EA	NM_012062.4	c.436G>A	p.D146N	/	/	AD	M	heter	Male
**AP1360**	***DNM1L***	33387674	F032	P037	/	II2	Caucasian	Non-EA	NM_012062.4	c.115A>G	p.Ser39Gly	/	/	AD	M	heter	Male
**AP1361**	***DNM1L***	33619735	F033	P038	/	patient 1	Caucasian	Non-EA	NM_012062.4	c.428C>G	p.Thr143Arg	/	/	AD	M	heter	/
**AP1362**	***DNM1L***	33619735	F034	P039	/	patient 2	Caucasian	Non-EA	NM_012062.4	c.1207C>T	p.Arg403Cys	/	/	AD	M	heter	/
**AP1363**	***DNM1L***	33718295	F035	P040	/		Caucasian	Non-EA	NM_012062.4	c.445 G>A	p.G149R	/	/	AD	M	heter	Male
**AP1364**	***DNM1L***	34307245	F036	P041	/	II-2	Chinese	EA	NM_012062.4	c.116 G>A	p.Ser39Asn	/	/	AD	M	heter	Male
**AP1365**	***DNM1L***	34573276	F037	P042	Family1	patient	Caucasian	Non-EA	NM_012062.4	c.1085G>A	p.Gly362Ser	c.1535T>C	p.Ile512Thr	AR	M	compound I	Male
**AP1366**	***DNM1L***	33233419	F038	P043	/	patient6	Caucasian	Non-EA	NM_012062.4	c.223A>G	p.Lys75Glu	/	/	AD	M	heter	Male
**AP1367**	***DNMT1***	33270637	F001	P001	/	11	Caucasian	Non-EA	NM_00113082	c.2858A>G	p.Asp953Gly	/	/	AD	M	heter	Male
**AP1368**	***DNMT1***	33098801	F002	P002	/	CB-DYS-103	Caucasian	Non-EA	NM_00113082	c.4250T>C	p.Met1417Thr	/	/	AD	M	heter	/
**AP1369**	***DNMT1***	32754641	F003	P003	/	CASE 1	Caucasian	Non-EA	NM_00113082	c.1528G>A	p.Glu510Lys	/	/	AD	M	heter	Male
**AP1370**	***DNMT1***	32754641	F004	P004	/	CASE 2	Caucasian	Non-EA	NM_00113082	c.4636C>G	p.Pro1546Ala	/	/	AD	M	heter	Male
**AP1371**	***DNMT1***	32754641	F005	P005	/	CASE 3	Caucasian	Non-EA	NM_00113082	c.1709C>T	p.Ala570Val	/	/	AD	M	heter	Male
**AP1372**	***DNMT1***	32754641	F006	P006	/	CASE 4	Caucasian	Non-EA	NM_00113082	c.1489A>C	p.Thr497Pro	/	/	AD	M	heter	Female
**AP1373**	***DNMT1***	32754641	F007	P007	/	CASE 5	Caucasian	Non-EA	NM_00113082	c.1531T>C	p.Tyr511His	/	/	AD	M	heter	Male
**AP1374**	***DNMT1***	31957642	F008	P008	/		Caucasian	Non-EA	NM_00113082	c.1723G>A	p.Glu575Lys	/	/	AD	M	heter	Female
**AP1375**	***DNMT1***	31807999	F009	P009	/		Caucasian	Non-EA	NM_00113082	c.1700T>C	p.Leu567Pro	/	/	AD	M	heter	Male
**AP1376**	***DNMT1***	31393079	F010	P010	/	VI	Caucasian	Non-EA	NM_00113082	c.1250C>T	p.Ala417Val	/	/	AD	M	heter	Male
**AP1377**	***DNMT1***	31217084	F011	P011	/	2	Caucasian	Non-EA	NM_00113082	c.3139A>T	p.Thr1047Ser	/	/	AD	M	heter	Male
**AP1378**	***DNMT1***	30911858	F012	P012	/		Caucasian	Non-EA	NM_00113082	c.1794C > T	p.Arg598Trp	/	/	AD	M	heter	Male
**AP1379**	***DNMT1***	30342480	F013	P013	/		Chinese	EA	NM_00113082	c.1618T>A	p.Tyr540Asn	/	/	AD	M	heter	Female
**AP1380**	***DNMT1***	27277422	F014	P014	/		Caucasian	Non-EA	NM_00113082	c.1635_1637delCAA	p.Asn545del	/	/	AD	M	heter	Male
**AP1381**	***DNMT1***	27217319	F015	P015	/		Caucasian	Non-EA	NM_00113082	c.2053G>A	p.Ala685Thr	/	/	AD	M	heter	Female
**AP1382**	***DNMT1***	25678562	F016	P017	Kindred-1	II-2	Caucasian	Non-EA	NM_00113082	c.1489A>C	p.Thr497Pro	/	/	AD	M	heter	Female
**AP1383**	***DNMT1***	25678562	F017	P018	Kindred-2	II-1	Caucasian	Non-EA	NM_00113082	c.1520C>T	p.Pro507Leu	/	/	AD	M	heter	Male
**AP1384**	***DNMT1***	25678562	F018	P019	Kindred-3	I-1	Caucasian	Non-EA	NM_00113082	c.1618T>A	p.Tyr540Asn	/	/	AD	M	heter	Male
**AP1385**	***DNMT1***	25678562	F018	P020	Kindred-3	II-1	Caucasian	Non-EA	NM_00113082	c.1618T>A	p.Tyr540Asn	/	/	AD	M	heter	Male
**AP1386**	***DNMT1***	25678562	F019	P021	Kindred-4	I-1	Caucasian	Non-EA	NM_00113082	c.1640T>A	p.Ile547Asn	/	/	AD	M	heter	Male
**AP1387**	***DNMT1***	25678562	F019	P022	Kindred-4	II-1	Caucasian	Non-EA	NM_00113082	c.1640T>A	p.Ile547Asn	/	/	AD	M	heter	Female
**AP1388**	***DNMT1***	25678562	F020	P023	Kindred-5	II-1	Caucasian	Non-EA	NM_00113082	c.1532A>G	p.Tyr511Cys	/	/	AD	M	heter	Male
**AP1389**	***DNMT1***	25678562	F021	P024	Kindred-6	I-1	Caucasian	Non-EA	NM_00113082	c.1106G>T	p.Cys369Phe	/	/	AD	M	heter	Female
**AP1390**	***DNMT1***	25678562	F021	P025	Kindred-6	II-1	Caucasian	Non-EA	NM_00113082	c.1106G>T	p.Cys369Phe	/	/	AD	M	heter	Female
**AP1391**	***DNMT1***	25678562	F021	P026	Kindred-6	II-2	Caucasian	Non-EA	NM_00113082	c.1106G>T	p.Cys369Phe	/	/	AD	M	heter	Male
**AP1392**	***DNMT1***	25678562	F021	P027	Kindred-6	III-2	Caucasian	Non-EA	NM_00113082	c.1106G>T	p.Cys369Phe	/	/	AD	M	heter	Male
**AP1393**	***DNMT1***	25678562	F021	P028	Kindred-6	III-3	Caucasian	Non-EA	NM_00113082	c.1106G>T	p.Cys369Phe	/	/	AD	M	heter	Male
**AP1394**	***DNMT1***	25678562	F021	P029	Kindred-6	IV-1	Caucasian	Non-EA	NM_00113082	c.1106G>T	p.Cys369Phe	/	/	AD	M	heter	Female
**AP1395**	***DNMT1***	25678562	F021	P030	Kindred-6	IV-4	Caucasian	Non-EA	NM_00113082	c.1532A>G	p.Tyr511Cys	/	/	AD	M	heter	Male
**AP1396**	***DNMT1***	25678562	F022	P031	Kindred-7	II-3	Caucasian	Non-EA	NM_00113082	c.1532A>G	p.Tyr511Cys	/	/	AD	M	heter	Female
**AP1397**	***DNMT1***	25678562	F022	P032	Kindred-7	II-6	Caucasian	Non-EA	NM_00113082	c.1532A>G	p.Tyr511Cys	/	/	AD	M	heter	Female
**AP1398**	***DNMT1***	25678562	F022	P033	Kindred-7	II-8	Caucasian	Non-EA	NM_00113082	c.1532A>G	p.Tyr511Cys	/	/	AD	M	heter	Female
**AP1399**	***DNMT1***	25678562	F022	P034	Kindred-7	II-9	Caucasian	Non-EA	NM_00113082	c.1532A>G	p.Tyr511Cys	/	/	AD	M	heter	Female
**AP1400**	***DNMT1***	25678562	F022	P035	Kindred-7	III-1	Caucasian	Non-EA	NM_00113082	c.1532A>G	p.Tyr511Cys	/	/	AD	M	heter	Female
**AP1401**	***DNMT1***	25678562	F022	P036	Kindred-7	III-2	Caucasian	Non-EA	NM_00113082	c.1532A>G	p.Tyr511Cys	/	/	AD	M	heter	Male
**AP1402**	***DNMT1***	25678562	F022	P037	Kindred-7	III-3	Caucasian	Non-EA	NM_00113082	c.1532A>G	p.Tyr511Cys	/	/	AD	M	heter	Female
**AP1403**	***DNMT1***	25678562	F022	P038	Kindred-7	III-5	Caucasian	Non-EA	NM_00113082	c.1532A>G	p.Tyr511Cys	/	/	AD	M	heter	Female
**AP1404**	***DNMT1***	25678562	F022	P039	Kindred-7	IV-3	Caucasian	Non-EA	NM_00113082	c.1532A>G	p.Tyr511Cys	/	/	AD	M	heter	Female
**AP1405**	***DNMT1***	25678562	F022	P040	Kindred-7	IV-4	Caucasian	Non-EA	NM_00113082	c.1532A>G	p.Tyr511Cys	/	/	AD	M	heter	Female
**AP1406**	***DNMT1***	25678562	F023	P041	Kindred-8	I-2	Caucasian	Non-EA	NM_00113082	c.1531T>C	p.Tyr511His	/	/	AD	M	heter	Male
**AP1407**	***DNMT1***	25678562	F023	P042	Kindred-8	I-3	Caucasian	Non-EA	NM_00113082	c.1531T>C	p.Tyr511His	/	/	AD	M	heter	Male
**AP1408**	***DNMT1***	25678562	F023	P043	Kindred-8	I-4	Caucasian	Non-EA	NM_00113082	c.1531T>C	p.Tyr511His	/	/	AD	M	heter	Male
**AP1409**	***DNMT1***	25678562	F023	P044	Kindred-8	II-2	Caucasian	Non-EA	NM_00113082	c.1531T>C	p.Tyr511His	/	/	AD	M	heter	Male
**AP1410**	***DNMT1***	25678562	F023	P045	Kindred-8	II-3	Caucasian	Non-EA	NM_00113082	c.1531T>C	p.Tyr511His	/	/	AD	M	heter	Male
**AP1411**	***DNMT1***	25678562	F023	P046	Kindred-8	II-4	Caucasian	Non-EA	NM_00113082	c.1531T>C	p.Tyr511His	/	/	AD	M	heter	Female
**AP1412**	***DNMT1***	25678562	F023	P047	Kindred-8	II-5	Caucasian	Non-EA	NM_00113082	c.1531T>C	p.Tyr511His	/	/	AD	M	heter	Male
**AP1413**	***DNMT1***	25678562	F023	P048	Kindred-8	II-6	Caucasian	Non-EA	NM_00113082	c.1531T>C	p.Tyr511His	/	/	AD	M	heter	Female
**AP1414**	***DNMT1***	25678562	F023	P049	Kindred-8	II-7	Caucasian	Non-EA	NM_00113082	c.1531T>C	p.Tyr511His	/	/	AD	M	heter	Male
**AP1415**	***DNMT1***	25678562	F023	P050	Kindred-8	II-8	Caucasian	Non-EA	NM_00113082	c.1531T>C	p.Tyr511His	/	/	AD	M	heter	Male
**AP1416**	***DNMT1***	25678562	F024	P051	Kindred-9	I-1	Caucasian	Non-EA	NM_00113082	c.1532A>G	p.Tyr511Cys	/	/	AD	M	heter	Male
**AP1417**	***DNMT1***	25678562	F024	P052	Kindred-9	II-3	Caucasian	Non-EA	NM_00113082	c.1532A>G	p.Tyr511Cys	/	/	AD	M	heter	Female
**AP1418**	***DNMT1***	25678562	F024	P053	Kindred-9	II-5	Caucasian	Non-EA	NM_00113082	c.1532A>G	p.Tyr511Cys	/	/	AD	M	heter	Female
**AP1419**	***DNMT1***	25678562	F024	P054	Kindred-9	III-1	Caucasian	Non-EA	NM_00113082	c.1532A>G	p.Tyr511Cys	/	/	AD	M	heter	Female
**AP1420**	***DNMT1***	25678562	F024	P055	Kindred-9	III-3	Caucasian	Non-EA	NM_00113082	c.1532A>G	p.Tyr511Cys	/	/	AD	M	heter	Female
**AP1421**	***DNMT1***	25678562	F024	P056	Kindred-9	III-4	Caucasian	Non-EA	NM_00113082	c.1532A>G	p.Tyr511Cys	/	/	AD	M	heter	Female
**AP1422**	***DNMT1***	25678562	F024	P057	Kindred-9	III-6	Caucasian	Non-EA	NM_00113082	c.1532A>G	p.Tyr511Cys	/	/	AD	M	heter	Male
**AP1423**	***DNMT1***	25678562	F024	P058	Kindred-9	III-7	Caucasian	Non-EA	NM_00113082	c.1532A>G	p.Tyr511Cys	/	/	AD	M	heter	Male
**AP1424**	***DNMT1***	25678562	F024	P059	Kindred-9	III-9	Caucasian	Non-EA	NM_00113082	c.1532A>G	p.Tyr511Cys	/	/	AD	M	heter	Male
**AP1425**	***DNMT1***	25678562	F024	P060	Kindred-9	IV-4	Caucasian	Non-EA	NM_00113082	c.1532A>G	p.Tyr511Cys	/	/	AD	M	heter	Male
**AP1426**	***DNMT1***	25678562	F024	P061	Kindred-9	IV-5	Caucasian	Non-EA	NM_00113082	c.1532A>G	p.Tyr511Cys	/	/	AD	M	heter	Female
**AP1427**	***DNMT1***	24727570	F025	P062	K1	II-1	Italian	Non-EA	NM_00113082	c.1709C>T	p.Ala570Val	/	/	AD	M	heter	/
**AP1428**	***DNMT1***	24727570	F026	P064	K2	III-1	Italian	Non-EA	NM_00113082	c.1814G>C	p.Gly605Ala	/	/	AD	M	heter	/
**AP1429**	***DNMT1***	24727570	F027	P066	K3	III-1	Italian	Non-EA	NM_00113082	c.1520C>G	p.Pro507Arg	/	/	AD	M	heter	/
**AP1430**	***DNMT1***	24727570	F027	P067	K3	III-2	Italian	Non-EA	NM_00113082	c.1520C>G	p.Pro507Arg	/	/	AD	M	heter	/
**AP1431**	***DNMT1***	24727570	F028	P068	K4	I-1	Italian	Non-EA	NM_00113082	c.1562_1564delAGA	p.Lys521del	/	/	AD	M	heter	/
**AP1432**	***DNMT1***	24709307	F029	P069	/	II-1	Caucasian	Non-EA	NM_00113082	c.1709C>T	p.Ala570Val	/	/	AD	M	heter	Male
**AP1433**	***DNMT1***	23904686	F030	P070	/		Brazilian	Non-EA	NM_00113082	c.1786T>C	p.Cys596Arg	/	/	AD	M	heter	Female
**AP1434**	***DNMT1***	23521649	F031	P071	/		Japanese	EA	NM_00113082	c.1706A>G	p.His569Arg	/	/	AD	M	heter	Male
**AP1435**	***DNMT1***	22328086	F032	P072	/	proband 1	Italian	Non-EA	NM_00113082	c.1709C>T	p.Ala570Val	/	/	AD	M	heter	Male
**AP1436**	***DNMT1***	22328086	F033	P073	/	US	Caucasian	Non-EA	NM_00113082	c.1709C>T	p.Ala570Val	/	/	AD	M	heter	Male
**AP1437**	***DNMT1***	22328086	F034	P074	/	Italian	Italian	Non-EA	NM_00113082	c.1814G>C	p.Gly605Ala	/	/	AD	M	heter	Male
**AP1438**	***DNMT1***	22328086	F035	P075	/	Swedish	Swedish	Non-EA	NM_00113082	c.1816G>T	p.Val606Phe	/	/	AD	M	heter	Male
**AP1439**	***DNMT1***	21532572	F036	P076	K1	V7	Caucasian	Non-EA	NM_00113082	c.1532A>G	p.Tyr511Cys	/	/	AD	M	heter	Female
**AP1440**	***DNMT1***	21532572	F036	P077	K1	V12	Caucasian	Non-EA	NM_00113082	c.1532A>G	p.Tyr511Cys	/	/	AD	M	heter	Male
**AP1441**	***DNMT1***	21532572	F036	P078	K1	V14	Caucasian	Non-EA	NM_00113082	c.1532A>G	p.Tyr511Cys	/	/	AD	M	heter	Female
**AP1442**	***DNMT1***	21532572	F036	P079	K1	V16	Caucasian	Non-EA	NM_00113082	c.1532A>G	p.Tyr511Cys	/	/	AD	M	heter	Female
**AP1443**	***DNMT1***	21532572	F036	P080	K1	VI-11	Caucasian	Non-EA	NM_00113082	c.1532A>G	p.Tyr511Cys	/	/	AD	M	heter	Female
**AP1444**	***DNMT1***	21532572	F036	P081	K1	VI-23	Caucasian	Non-EA	NM_00113082	c.1532A>G	p.Tyr511Cys	/	/	AD	M	heter	Male
**AP1445**	***DNMT1***	21532572	F036	P082	K1	VI-30	Caucasian	Non-EA	NM_00113082	c.1532A>G	p.Tyr511Cys	/	/	AD	M	heter	Male
**AP1446**	***DNMT1***	21532572	F036	P083	K1	VI-33	Caucasian	Non-EA	NM_00113082	c.1532A>G	p.Tyr511Cys	/	/	AD	M	heter	Male
**AP1447**	***DNMT1***	21532572	F036	P084	K1	VI-36	Caucasian	Non-EA	NM_00113082	c.1532A>G	p.Tyr511Cys	/	/	AD	M	heter	Male
**AP1448**	***DNMT1***	21532572	F036	P085	K1	VII13	Caucasian	Non-EA	NM_00113082	c.1532A>G	p.Tyr511Cys	/	/	AD	M	heter	Female
**AP1449**	***DNMT1***	21532572	F036	P086	K1	VII 15	Caucasian	Non-EA	NM_00113082	c.1532A>G	p.Tyr511Cys	/	/	AD	M	heter	Female
**AP1450**	***DNMT1***	21532572	F037	P087	K2	II-2	Caucasian	Non-EA	NM_00113082	c.1518_1520delTCCin	p.Asp506_Pro507	/	/	AD	M	heter	Female
**AP1451**	***DNMT1***	21532572	F037	P088	K2	III-1	Caucasian	Non-EA	NM_00113082	c.1518_1520delTCCin	p.Asp506_Pro507	/	/	AD	M	heter	Male
**AP1452**	***DNMT1***	21532572	F037	P089	K2	III-3	Caucasian	Non-EA	NM_00113082	c.1518_1520delTCCin	p.Asp506_Pro507	/	/	AD	M	heter	Female
**AP1453**	***DNMT1***	21532572	F038	P090	K3	IV-16	Caucasian	Non-EA	NM_00113082	c.1532A>G	p.Tyr511Cys	/	/	AD	M	heter	Female
**AP1454**	***DNMT1***	21532572	F038	P091	K3	IV-17	Caucasian	Non-EA	NM_00113082	c.1532A>G	p.Tyr511Cys	/	/	AD	M	heter	Male
**AP1455**	***DNMT1***	21532572	F038	P092	K3	V-1	Caucasian	Non-EA	NM_00113082	c.1532A>G	p.Tyr511Cys	/	/	AD	M	heter	Female
**AP1456**	***DNMT1***	21532572	F039	P093	K4	III-3	Caucasian	Non-EA	NM_00113082	c.1532A>G	p.Tyr511Cys	/	/	AD	M	heter	Male

**AP1457**	***FDXR***	28965846	F001	P001	F1	Individuals 1	Caucasian	Non-EA	NM_024417	c.916C>T	p.Arg306Cys	c.916C>T	p.Arg306Cys	AR	M+M	homo	Female
**AP1458**	***FDXR***	28965846	F001	P002	F1	Individuals 2	Caucasian	Non-EA	NM_024417	c.916C>T	p.Arg306Cys	c.916C>T	p.Arg306Cys	AR	M+M	homo	Female
**AP1459**	***FDXR***	28965846	F001	P003	F1	Individuals 3	Caucasian	Non-EA	NM_024417	c.916C>T	p.Arg306Cys	c.916C>T	p.Arg306Cys	AR	M+M	homo	Female
**AP1460**	***FDXR***	28965846	F001	P004	F1	Individuals 4	Caucasian	Non-EA	NM_024417	c.916C>T	p.Arg306Cys	c.916C>T	p.Arg306Cys	AR	M+M	homo	Female
**AP1461**	***FDXR***	28965846	F002	P005	F2	Individuals 5	Caucasian	Non-EA	NM_024417	c.916C>T	p.Arg306Cys	c.1255 C>T	p.Gln419*	AR	M+T	compound I	Male
**AP1462**	***FDXR***	28965846	F003	P006	F3	Individuals 6	Caucasian	Non-EA	NM_024417	c.724C>T	p.Arg242Trp	c.979C>A	p.Arg327Ser	AR	M+M	compound I	Male
**AP1463**	***FDXR***	28965846	F004	P007	F4	Individuals 7	Caucasian	Non-EA	NM_024417	c.643C>G	p.Leu215Val	c.1429 G>A	p.Glu477Lys	AR	M+M	compound I	Female
**AP1464**	***FDXR***	28965846	F004	P008	F4	Individuals 8	Caucasian	Non-EA	NM_024417	c.643C>G	p.Leu215Val	c.1429 G>A	p.Glu477Lys	AR	M+M	compound I	Female
**AP1465**	***FDXR***	29040572	F005	P009	1	II-3	Caucasian	Non-EA	NM_024417	c.1156C>T	p.(Arg386Trp)	c.1156C>T	p.(Arg386Trp)	AR	M+M	homo	Female
**AP1466**	***FDXR***	29040572	F005	P010	1	II-2	Caucasian	Non-EA	NM_024417	c.1156C>T	p.(Arg386Trp)	c.1156C>T	p.(Arg386Trp)	AR	M+M	homo	Male
**AP1467**	***FDXR***	29040572	F006	P011	2		Caucasian	Non-EA	NM_024417	c.151T>C	p.(Phe51Leu)	c.1309G>A	p.Gly437Ser	AR	M+M	compound I	Male
**AP1468**	***FDXR***	29040572	F007	P012	3		Caucasian	Non-EA	NM_024417	c.1156C>T	p.(Arg386Trp)	c.1156C>T	p.(Arg386Trp)	AR	M+M	homo	Male
**AP1469**	***FDXR***	29040572	F008	P013	4		Caucasian	Non-EA	NM_024417	c.1156C>T	p.(Arg386Trp)	c.1156C>T	p.(Arg386Trp)	AR	M+M	homo	Male
**AP1470**	***FDXR***	29040572	F009	P014	5		Caucasian	Non-EA	NM_024417	c.221C>T	p.(Pro74Leu)	c.1156C>T	p.(Arg386Trp)	AR	M+M	compound I	Female
**AP1471**	***FDXR***	29040572	F010	P015	6		Caucasian	Non-EA	NM_024417	c.427A>T	p.Ile143Phe	c.1058G>A	p.Cys353Tyr	AR	M+M	compound I	Female
**AP1472**	***FDXR***	29040572	F011	P016	7		Caucasian	Non-EA	NM_024417	c.613A>G	p.Thr205Ala	c.1309G>T	p.Gly437Cys	AR	M+M	compound I	Male
**AP1473**	***FDXR***	29040572	F012	P017	8		Caucasian	Non-EA	NM_024417	c.1102G>A	p.Asp368Asn	c.1102G>A	p.Asp368Asn	AR	M+M	homo	Male
**AP1474**	***FDXR***	29040572	F013	P018	9		Caucasian	Non-EA	NM_024417	c.472G>A	p.Val158Met	c.619A>T	p.Ile207Phe	AR	M+M	compound I	Female
**AP1475**	***FDXR***	29040572	F014	P019	10		Caucasian	Non-EA	NM_024417	c.925C>T	p.Arg309*	c.1208C>T	p.Pro403Leu	AR	M+T	compound I	Male
**AP1476**	***FDXR***	29040572	F015	P020	11		Caucasian	Non-EA	NM_024417	c.1156C>T	p.(Arg386Trp)	c.1156C>T	p.(Arg386Trp)	AR	M+M	homo	Male
**AP1477**	***FDXR***	29040572	F016	P021	12		Caucasian	Non-EA	NM_024417	c.472G>A	p.Val158Met	c.838A>T	p.(Thr280Ser)	AR	M+T	compound I	Male
**AP1478**	***FDXR***	29040572	F017	P022	13	II-2	Caucasian	Non-EA	NM_024417	c.1156C>T	p.(Arg386Trp)	c.1156C>T	p.(Arg386Trp)	AR	M+M	homo	Male
**AP1479**	***FDXR***	30250212	F018	P023	/	case 1	Caucasian	Non-EA	NM_024417	c.1A > G	p.M1?	c.463C>T	p.Arg155Trp	AR	M+T	compound I	Male
**AP1480**	***FDXR***	30250212	F019	P024	/	case 2	Caucasian	Non-EA	NM_024417	c.929delG	p.Ser310Thrfs*19	c.578G>A	p.Arg193His	AR	M+T	compound I	Female
**AP1481**	***FDXR***	33348459	F020	P025	F1	Subject 1	Caucasian	Non-EA	NM_024417	c.35C>G	p.Ser12Trp	c.683G>T	p.Arg228Leu	AR	M+M	compound I	Female
**AP1482**	***FDXR***	33348459	F021	P026	F2	Subject 2	Caucasian	Non-EA	NM_024417	c.576delTCTGGACGTC	p.Leu189_Ala192	c.332T>C	p.Val111Ala	AR	M+M	compound I	Male
**AP1483**	***FDXR***	33348459	F022	P027	F3	Subject 3.1	Caucasian	Non-EA	NM_024417	c.1343G>A	p.Arg448Gln	c.325G>A	p.Gly109Ser	AR	M+M	compound I	Female
**AP1484**	***FDXR***	33348459	F022	P028	F3	Subject 3.2	Caucasian	Non-EA	NM_024417	c.1343G>A	p.Arg448Gln	c.325G>A	p.Gly109Ser	AR	M+M	compound I	Male
**AP1485**	***FDXR***	33348459	F023	P029	F4	Subject 4	Caucasian	Non-EA	NM_024417	c.683G>T	p.Arg228Leu	c.1058G>A	p.Cys353Ty	AR	M+M	compound I	Male
**AP1486**	***FDXR***	33348459	F024	P030	F5	Subject 5	Caucasian	Non-EA	NM_024417	c.632C>T	p.Ala211Val	c.632C>T	p.Ala211Val	AR	M+M	homo	Female
**AP1487**	***FDXR***	33348459	F025	P031	F6	Subject 6	Caucasian	Non-EA	NM_024417	c.632C>T	p.Ala211Val	c.632C>T	p.Ala211Val	AR	M+M	homo	Female
**AP1488**	***FDXR***	33600046	F026	P032	/	11	Caucasian	Non-EA	NM_024417	c.463C>T	p.Arg155Trp	c.463C>T	p.Arg155Trp	AR	M+M	homo	/
**AP1489**	***FDXR***	33742450	F027	P033	/		Caucasian	Non-EA	NM_024417	c.250C>T	p.Pro84Ser	c.634G>C	p.Asp212His	AR	M+M	compound I	Male
**AP1490**	***FDXR***	33938912	F028	P034	Family 1		Caucasian	Non-EA	NM_024417	c.577C>T	p.Arg193Cys	c.1115C>A	p.Pro372His	AR	M+M	compound I	Male
**AP1491**	***FDXR***	33938912	F029	P035	Family 2		Caucasian	Non-EA	NM_024417	c. 925C>T	p.Arg309*	c.1115C>A	p.Pro372His	AR	M+T	compound I	Male
**AP1492**	***FDXR***	33938912	F029	P036	Family 2		Caucasian	Non-EA	NM_024417	c. 925C>T	p.Arg309*	c.1115C>A	p.Pro372His	AR	M+T	compound I	Male
**AP1493**	***FDXR***	33938912	F030	P037	Family 3		Caucasian	Non-EA	NM_024417	c.461C>T	p.Ala154Val	c.823C>T	p.Arg275Trp	AR	M+M	compound I	Male
**AP1494**	***FDXR***	33938912	F031	P038	Family 4		Caucasian	Non-EA	NM_024417	c.1115C>A	p.Pro372His	c.1279G>C	p.Ala427Pro	AR	M+M	compound I	Female
**AP1495**	***FDXR***	33938912	F032	P039	Family 5		Caucasian	Non-EA	NM_024417	c.614C>T	p.Thr205Met	c.823C>T	p.Arg275Trp	AR	M+M	compound I	Male
**AP1496**	***FDXR***	33938912	F033	P040	Family 6		Caucasian	Non-EA	NM_024417	c.1115C>A	p.Pro372His	chr17:748186	p.?	AR	M+M	compound I	Male
**AP1497**	***FDXR***	33938912	F034	P041	Family 7		Caucasian	Non-EA	NM_024417	c.1115C>A	p.Pro372His	c.1189G>A	p.Gly397Ser	AR	M+M	compound I	Male
**AP1498**	***FDXR***	33938912	F034	P041	Family 7		Caucasian	Non-EA	NM_024417	c.1115C>A	p.Pro372His	c.1189G>A	p.Gly397Ser	AR	M+M	compound I	Female
**AP1499**	***FDXR***	33938912	F035	P042	Family 8		Caucasian	Non-EA	NM_024417	c.724C>T	p.Arg242Trp	c.916C>T	p.Gly397Ser	AR	M+M	compound I	Female
**AP1500**	***FDXR***	34416374	F036	P043	/		Caucasian	Non-EA	NM_024417	c.752G>A	p.Thr251Met	c.499delG	p.Glu167Argfs	AR	M+M	compound I	/
**AP1501**	***FDXR***	37481223	F037	P044	SRF-F1		Chinese	EA	NM_024417	c.383C > T	p.Ala128Val	c.963delG	p.Arg322Glyfs	AR	M+T	compound I	Female
**AP1502**	***FDXR***	37481223	F038	P045	SRF-F2		Chinese	EA	NM_024417	c.940G>T	p.Val314Leu	c.394-11T>G	p.?	AR	M+T	compound I	Female
**AP1503**	***FDXR***	37481223	F039	P046	SRF-F3		Chinese	EA	NM_024417	c. 940G > T	p.Val314Leu	c.1052_1053d	p.Leu351Profs	AR	M+T	compound I	Female
**AP1504**	***FDXR***	37481223	F040	P047	SRF-F4		Chinese	EA	NM_024417	c.1002+1G > A	p.?	c.916C > T	p.Arg306Cys	AR	M+T	compound I	Female
**AP1505**	***FDXR***	37481223	F041	P048	SRF-F5		Chinese	EA	NM_024417	c.472G > A	p.Val158Met	c.577C > T	p.Arg193Cys	AR	M+M	compound I	Male
**AP1506**	***FXN***	32488064/8596	F001	P001	/	1	Caucasian	Non-EA	NM_000144.5	(GAA)n	p.?	GAAn	p.?	AR	CNV+CNV	compound I	Male
**AP1507**	***FXN***	32488064/8596	F002	P002	/	2	Caucasian	Non-EA	NM_000144.5	(GAA)n	p.?	GAAn	p.?	AR	CNV+CNV	compound I	Male
**AP1508**	***FXN***	32488064/8596	F003	P003	/	3	Caucasian	Non-EA	NM_000144.5	(GAA)n	p.?	GAAn	p.?	AR	CNV+CNV	compound I	Male
**AP1509**	***FXN***	32488064/8596	F004	P004	/	4	Caucasian	Non-EA	NM_000144.5	(GAA)n	p.?	GAAn	p.?	AR	CNV+CNV	compound I	Female
**AP1510**	***FXN***	32488064/8596	F005	P005	/	5	Caucasian	Non-EA	NM_000144.5	(GAA)n	p.?	GAAn	p.?	AR	CNV+CNV	compound I	Male
**AP1511**	***FXN***	32488064/8596	F006	P006	/	6	Caucasian	Non-EA	NM_000144.5	(GAA)n	p.?	GAAn	p.?	AR	CNV+CNV	compound I	Female
**AP1512**	***FXN***	32488064/8596	F007	P007	/	7	Caucasian	Non-EA	NM_000144.5	(GAA)n	p.?	GAAn	p.?	AR	CNV+CNV	compound I	Female
**AP1513**	***FXN***	32488064/8596	F008	P008	/	8	Caucasian	Non-EA	NM_000144.5	(GAA)n	p.?	GAAn	p.?	AR	CNV+CNV	compound I	Male
**AP1514**	***FXN***	32488064/8596	F009	P009	/	9	Caucasian	Non-EA	NM_000144.5	(GAA)n	p.?	GAAn	p.?	AR	CNV+CNV	compound I	Female
**AP1515**	***FXN***	32488064/8596	F010	P010	/	10	Caucasian	Non-EA	NM_000144.5	(GAA)n	p.?	GAAn	p.?	AR	CNV+CNV	compound I	Male
**AP1516**	***FXN***	32488064/8596	F011	P011	/	11	Caucasian	Non-EA	NM_000144.5	(GAA)n	p.?	GAAn	p.?	AR	CNV+CNV	compound I	Male
**AP1517**	***FXN***	32488064/8596	F012	P012	/	12	Caucasian	Non-EA	NM_000144.5	(GAA)n	p.?	GAAn	p.?	AR	CNV+CNV	compound I	Female
**AP1518**	***FXN***	32488064/8596	F013	P013	/	13	Caucasian	Non-EA	NM_000144.5	(GAA)n	p.?	GAAn	p.?	AR	CNV+CNV	compound I	Male
**AP1519**	***FXN***	32488064/8596	F014	P014	/	14	Caucasian	Non-EA	NM_000144.5	(GAA)n	p.?	GAAn	p.?	AR	CNV+CNV	compound I	Male
**AP1520**	***FXN***	32488064/8596	F015	P015	/	15	Caucasian	Non-EA	NM_000144.5	(GAA)n	p.?	GAAn	p.?	AR	CNV+CNV	compound I	Male
**AP1521**	***FXN***	32488064/8596	F016	P016	/	16	Caucasian	Non-EA	NM_000144.5	(GAA)n	p.?	GAAn	p.?	AR	CNV+CNV	compound I	Male
**AP1522**	***FXN***	32488064/8596	F017	P017	/	17	Caucasian	Non-EA	NM_000144.5	(GAA)n	p.?	GAAn	p.?	AR	CNV+CNV	compound I	Female
**AP1523**	***FXN***	32488064/8596	F018	P018	/	18	Caucasian	Non-EA	NM_000144.5	(GAA)n	p.?	GAAn	p.?	AR	CNV+CNV	compound I	Male
**AP1524**	***FXN***	32488064/8596	F019	P019	/	20	Caucasian	Non-EA	NM_000144.5	(GAA)n	p.?	GAAn	p.?	AR	CNV+CNV	compound I	Female
**AP1525**	***FXN***	32488064/8596	F020	P020	/	21	Caucasian	Non-EA	NM_000144.5	(GAA)n	p.?	GAAn	p.?	AR	CNV+CNV	compound I	Male
**AP1526**	***FXN***	32488064/8596	F021	P021	/	22	Caucasian	Non-EA	NM_000144.5	(GAA)n	p.?	GAAn	p.?	AR	CNV+CNV	compound I	Female
**AP1527**	***FXN***	32488064/8596	F022	P022	/	23	Caucasian	Non-EA	NM_000144.5	(GAA)n	p.?	GAAn	p.?	AR	CNV+CNV	compound I	Male
**AP1528**	***FXN***	32488064/8596	F023	P023	/	24	Caucasian	Non-EA	NM_000144.5	(GAA)n	p.?	GAAn	p.?	AR	CNV+CNV	compound I	Female
**AP1529**	***FXN***	32488064/8596	F024	P024	/	25	Caucasian	Non-EA	NM_000144.5	(GAA)n	p.?	GAAn	p.?	AR	CNV+CNV	compound I	Male
**AP1530**	***FXN***	32488064/8596	F025	P025	/	26	Caucasian	Non-EA	NM_000144.5	(GAA)n	p.?	GAAn	p.?	AR	CNV+CNV	compound I	Female
**AP1531**	***FXN***	32488064/8596	F026	P026	/	27	Caucasian	Non-EA	NM_000144.5	(GAA)n	p.?	GAAn	p.?	AR	CNV+CNV	compound I	Male
**AP1532**	***FXN***	32488064/8596	F027	P027	/	28	Caucasian	Non-EA	NM_000144.5	(GAA)n	p.?	GAAn	p.?	AR	CNV+CNV	compound I	Female
**AP1533**	***FXN***	32488064/8596	F028	P028	/	29	Caucasian	Non-EA	NM_000144.5	(GAA)n	p.?	GAAn	p.?	AR	CNV+CNV	compound I	Female
**AP1534**	***FXN***	32488064/8596	F029	P029	/	30	Caucasian	Non-EA	NM_000144.5	(GAA)n	p.?	GAAn	p.?	AR	CNV+CNV	compound I	Female
**AP1535**	***FXN***	32488064/8596	F030	P030	/	31	Caucasian	Non-EA	NM_000144.5	(GAA)n	p.?	GAAn	p.?	AR	CNV+CNV	compound I	Male
**AP1536**	***FXN***	32488064/8596	F031	P031	/	32	Caucasian	Non-EA	NM_000144.5	(GAA)n	p.?	GAAn	p.?	AR	CNV+CNV	compound I	Female
**AP1537**	***FXN***	32488064/8596	F032	P032	/	33	Caucasian	Non-EA	NM_000144.5	(GAA)n	p.?	GAAn	p.?	AR	CNV+CNV	compound I	Male
**AP1538**	***FXN***	32488064/8596	F033	P033	/	34	Caucasian	Non-EA	NM_000144.5	(GAA)n	p.?	GAAn	p.?	AR	CNV+CNV	compound I	Male
**AP1539**	***FXN***	32488064/8596	F034	P034	/	35	Caucasian	Non-EA	NM_000144.5	(GAA)n	p.?	GAAn	p.?	AR	CNV+CNV	compound I	Female
**AP1540**	***FXN***	32488064/8596	F035	P035	/	36	Caucasian	Non-EA	NM_000144.5	(GAA)n	p.?	GAAn	p.?	AR	CNV+CNV	compound I	Male
**AP1541**	***FXN***	31243663	F036	P036	/	P1-UNICAMP	Caucasian	Non-EA	NM_000144.5	c.157delC	p.Arg53Alafs*23	/	/	AD	Truncation	heter	/
**AP1542**	***FXN***	31243663	F037	P037	/	P2-UFRGS	Caucasian	Non-EA	NM_000144.5	c.157delC	p.Arg53Alafs*23	/	/	AD	Truncation	heter	/
**AP1543**	***FXN***	31243663	F038	P038	/	P3-USP-RP	Caucasian	Non-EA	NM_000144.5	c.482+1G>T	p.?	/	/	AD	Truncation	heter	/
**AP1544**	***FXN***	30159187	F039	P039	/		Caucasian	Non-EA	NM_000144.5	(GAA)n	p.?	c.317delT	p.Leu106*	AR	CNV+T	compound I	Male
**AP1545**	***FXN***	30159187	F039	P040	/		Caucasian	Non-EA	NM_000144.5	(GAA)n	p.?	c.317delT	p.Leu106*	AR	CNV+T	compound I	Male
**AP1546**	***FXN***	30159187	F040	P041	/		Caucasian	Non-EA	NM_000144.5	(GAA)n	p.?	c.464G>A	p.Trp155*	AR	CNV+T	compound I	Male
**AP1547**	***FXN***	30159187	F041	P042	/		Caucasian	Non-EA	NM_000144.5	(GAA)n	p.?	c.354C>G	p.Tyr118*	AR	CNV+T	compound I	Male
**AP1548**	***FXN***	30159187	F042	P043	/		Caucasian	Non-EA	NM_000144.5	(GAA)n	p.?	c.464G>A	p.Trp155*	AR	CNV+T	compound I	Male
**AP1549**	***FXN***	32488064	F043	P044	/	19	Caucasian	Non-EA	NM_000144.5	(GAA)n	p.?	GAAn	p.?	AR	CNV+CNV	compound I	/
**AP1550**	***FXN***	26635519	F044	P045	/		Caucasian	Non-EA	NM_000144.5	(GAA)n	p.?	c.410G > T	p.Gly137Val	AR	CNV+M	compound I	/
**AP1551**	***FXN***	26338206	F045	P046	/	1-1	Caucasian	Non-EA	NM_000144.5	(GAA)n	p.?	GAAn	p.?	AR	CNV+CNV	compound I	Female
**AP1552**	***FXN***	26338206	F046	P047	/	2-1	Caucasian	Non-EA	NM_000144.5	(GAA)n	p.?	GAAn	p.?	AR	CNV+CNV	compound I	Male
**AP1553**	***FXN***	26338206	F047	P048	/	3-1	Caucasian	Non-EA	NM_000144.5	(GAA)n	p.?	GAAn	p.?	AR	CNV+CNV	compound I	Female
**AP1554**	***FXN***	26338206	F048	P049	/	4-1	Caucasian	Non-EA	NM_000144.5	(GAA)n	p.?	GAAn	p.?	AR	CNV+CNV	compound I	Male
**AP1555**	***FXN***	26338206	F048	P050	/	4-2	Caucasian	Non-EA	NM_000144.5	(GAA)n	p.?	GAAn	p.?	AR	CNV+CNV	compound I	Male
**AP1556**	***FXN***	26338206	F049	P051	/	5-1	Caucasian	Non-EA	NM_000144.5	(GAA)n	p.?	GAAn	p.?	AR	CNV+CNV	compound I	Male
**AP1557**	***FXN***	26338206	F050	P052	/	6-1	Caucasian	Non-EA	NM_000144.5	(GAA)n	p.?	GAAn	p.?	AR	CNV+CNV	compound I	Female
**AP1558**	***FXN***	26338206	F051	P053	/	7-1	Caucasian	Non-EA	NM_000144.5	(GAA)n	p.?	GAAn	p.?	AR	CNV+CNV	compound I	Male
**AP1559**	***FXN***	26338206	F052	P054	/	8-1	Caucasian	Non-EA	NM_000144.5	(GAA)n	p.?	GAAn	p.?	AR	CNV+CNV	compound I	Female
**AP1560**	***FXN***	26338206	F053	P055	/	9-1	Caucasian	Non-EA	NM_000144.5	(GAA)n	p.?	GAAn	p.?	AR	CNV+CNV	compound I	Male
**AP1561**	***FXN***	26338206	F054	P056	/	10-1	Caucasian	Non-EA	NM_000144.5	(GAA)n	p.?	GAAn	p.?	AR	CNV+CNV	compound I	Male
**AP1562**	***FXN***	26338206	F055	P057	/	11-1	Caucasian	Non-EA	NM_000144.5	(GAA)n	p.?	GAAn	p.?	AR	CNV+CNV	compound I	Male
**AP1563**	***FXN***	26338206	F056	P058	/	12-1	Caucasian	Non-EA	NM_000144.5	(GAA)n	p.?	GAAn	p.?	AR	CNV+CNV	compound I	Male
**AP1564**	***FXN***	26338206	F057	P059	/	13-1	Caucasian	Non-EA	NM_000144.5	(GAA)n	p.?	GAAn	p.?	AR	CNV+CNV	compound I	Female
**AP1565**	***FXN***	26338206	F057	P060	/	13-2	Caucasian	Non-EA	NM_000144.5	(GAA)n	p.?	GAAn	p.?	AR	CNV+CNV	compound I	Female
**AP1566**	***FXN***	26338206	F058	P061	/	14-1	Caucasian	Non-EA	NM_000144.5	(GAA)n	p.?	GAAn	p.?	AR	CNV+CNV	compound I	Female
**AP1567**	***FXN***	26338206	F058	P062	/	14-2	Caucasian	Non-EA	NM_000144.5	(GAA)n	p.?	GAAn	p.?	AR	CNV+CNV	compound I	Female
**AP1568**	***FXN***	26338206	F059	P063	/	15-1	Caucasian	Non-EA	NM_000144.5	(GAA)n	p.?	GAAn	p.?	AR	CNV+CNV	compound I	Male
**AP1569**	***FXN***	26338206	F060	P064	/	16-1	Caucasian	Non-EA	NM_000144.5	(GAA)n	p.?	GAAn	p.?	AR	CNV+CNV	compound I	Male
**AP1570**	***FXN***	26338206	F061	P065	/	17-1	Caucasian	Non-EA	NM_000144.5	(GAA)n	p.?	GAAn	p.?	AR	CNV+CNV	compound I	Female
**AP1571**	***FXN***	26338206	F062	P066	/	18-1	Caucasian	Non-EA	NM_000144.5	(GAA)n	p.?	GAAn	p.?	AR	CNV+CNV	compound I	Male
**AP1572**	***FXN***	26338206	F063	P067	/	19-1	Caucasian	Non-EA	NM_000144.5	(GAA)n	p.?	GAAn	p.?	AR	CNV+CNV	compound I	Female
**AP1573**	***FXN***	26338206	F064	P068	/	20-1	Caucasian	Non-EA	NM_000144.5	(GAA)n	p.?	GAAn	p.?	AR	CNV+CNV	compound I	Female
**AP1574**	***FXN***	26338206	F065	P069	/	21-1	Caucasian	Non-EA	NM_000144.5	(GAA)n	p.?	GAAn	p.?	AR	CNV+CNV	compound I	Male
**AP1575**	***FXN***	26338206	F066	P070	/	22-1	Caucasian	Non-EA	NM_000144.5	(GAA)n	p.?	c.467T>C		AR	CNV+M	compound I	Male
**AP1576**	***FXN***	26338206	F067	P071	/	23-1	Caucasian	Non-EA	NM_000144.5	(GAA)n	p.?	g.120032_12	p.?	AR	CNV+CNV	compound I	Female
**AP1577**	***FXN***	25149925	F068	P073	/		Caucasian	Non-EA	NM_000144.5	c.317T>C	p.Leu106Ser	/		AD	M	compound I	/
**AP1578**	***FXN***	24816001	F069	P074	/	Pt1	Caucasian	Non-EA	NM_000144.5	(GAA)n	p.?	GAAn	p.?	AR	CNV+CNV	compound I	/
**AP1579**	***FXN***	24816001	F070	P075	/	Pt2	Caucasian	Non-EA	NM_000144.5	(GAA)n	p.?	GAAn	p.?	AR	CNV+CNV	compound I	/
**AP1580**	***FXN***	24816001	F071	P076	/	Pt3	Caucasian	Non-EA	NM_000144.5	(GAA)n	p.?	GAAn	p.?	AR	CNV+CNV	compound I	/
**AP1581**	***FXN***	24816001	F072	P077	/	Pt4	Caucasian	Non-EA	NM_000144.5	(GAA)n	p.?	GAAn	p.?	AR	CNV+CNV	compound I	/
**AP1582**	***FXN***	24816001	F073	P078	/	Pt5	Caucasian	Non-EA	NM_000144.5	(GAA)n	p.?	GAAn	p.?	AR	CNV+CNV	compound I	/
**AP1583**	***FXN***	24816001	F074	P079	/	Pt6	Caucasian	Non-EA	NM_000144.5	(GAA)n	p.?	GAAn		AR	CNV+CNV	compound I	/
**AP1584**	***FXN***	24816001	F075	P080	/	Pt7	Caucasian	Non-EA	NM_000144.5	(GAA)n	p.?	c.493C>T	p.Arg165Pro	AR	CNV+M	compound I	/
**AP1585**	***FXN***	24816001	F076	P081	/	Pt8	Caucasian	Non-EA	NM_000144.5	(GAA)n	p.?	c.493C>T	p.Arg165Pro	AR	CNV+M	compound I	/
**AP1586**	***FXN***	24816001	F076	P082	/	Pt9	Caucasian	Non-EA	NM_000144.5	(GAA)n	p.?	c.493C>T	p.Arg165Pro	AR	CNV+M	compound I	/
**AP1587**	***FXN***	24665325	F077	P083	/		Caucasian	Non-EA	NM_000144.5	(GAA)n	p.?	c.241T>G	p.Ser81Ala	AR	CNV+M	compound I	Male
**AP1588**	***FXN***	24665325	F078	P084	/		Caucasian	Non-EA	NM_000144.5	(GAA)n	p.?	c.574A>T	p.Ser192Cys	AR	CNV+M	compound I	Female
**AP1589**	***FXN***	24665325	F079	P085	/		Caucasian	Non-EA	NM_000144.5	(GAA)n	p.?	c.367T>G	p.Tyr186Asp	AR	CNV+M	compound I	Female
**AP1590**	***FXN***	23196337	F080	P086	/		Caucasian	Non-EA	NM_000144.5	(GAA)n	p.?	c.572delC	p.Thr191Ilefs*	AR	CNV+T	compound I	Female
**AP1591**	***FXN***	21830088	F081	P087	/	Sibling 1	Caucasian	Non-EA	NM_000144.5	(GAA)n	p.?	c.371_376del	p.Asp124_Ser	AR	CNV+T	compound I	Male
**AP1592**	***FXN***	21830088	F081	P088	/	Sibling 2	Caucasian	Non-EA	NM_000144.5	(GAA)n	p.?	c.371_376del	p.Asp124_Ser	AR	CNV+T	compound I	Female
**AP1593**	***FXN***	20675166	F082	P089	/	P.MUT.001	Caucasian	Non-EA	NM_000144.5	(GAA)n	p.?	c.460A>T	p.Ile154Phe	AR	CNV+M	compound I	Male
**AP1594**	***FXN***	20675166	F083	P090	/	P.MUT.002	Caucasian	Non-EA	NM_000144.5	(GAA)n	p.?	c.493C>T	p.Arg165Cys	AR	CNV+M	compound I	Male
**AP1595**	***FXN***	20675166	F084	P091	/	P.MUT.003	Caucasian	Non-EA	NM_000144.5	(GAA)n	p.?	c.463T>C	p.Trp155Arg	AR	CNV+M	compound I	Female
**AP1596**	***FXN***	20675166	F085	P092	/	P.MUT.004	Caucasian	Non-EA	NM_000144.5	(GAA)n	p.?	c.317T>C	p.Leu106Ser	AR	CNV+M	compound I	Male
**AP1597**	***FXN***	20675166	F086	P093	/	P.MUT.005	Caucasian	Non-EA	NM_000144.5	(GAA)n	p.?	c.389G>T	p.Gly130Val	AR	CNV+M	compound I	Male
**AP1598**	***FXN***	20675166	F087	P094	/	P.MUT.006	Caucasian	Non-EA	NM_000144.5	(GAA)n	p.?	c.389G>T	p.Gly130Val	AR	CNV+M	compound I	Male
**AP1599**	***FXN***	20675166	F088	P095	/	P.MUT.007	Caucasian	Non-EA	NM_000144.5	(GAA)n	p.?	c.389G>T	p.Gly130Val	AR	CNV+M	compound I	Female
**AP1600**	***FXN***	20675166	F089	P096	/	P.MUT.008	Caucasian	Non-EA	NM_000144.5	(GAA)n	p.?	c.483-1G>T	p.?	AR	CNV+T	compound I	Female
**AP1601**	***FXN***	20151403	F090	P097	/	Patient 4	Caucasian	Non-EA	NM_000144.5	c.264-1G>A	p.?	/	/	AD	T	heter	/
**AP1602**	***FXN***	19182486	F091	P098	/	*Patient 1 (IV-3)*	Caucasian	Non-EA	NM_000144.5	c.165+1G>A	p.?	/	/	AD	T	heter	Male
**AP1603**	***FXN***	19182486	F091	P099	/	*Patient 2 (IV-2)*	Caucasian	Non-EA	NM_000144.5	c.165+1G>A	p.?	/	/	AD	T	heter	Female
**AP1604**	***FXN***	18931386	F092	P100	1		Caucasian	Non-EA	NM_000144.5	(GAA)n	p.?	GAAn	p.?	AR	CNV+CNV	compound I	/
**AP1605**	***FXN***	18931386	F093	P101	2		Caucasian	Non-EA	NM_000144.5	(GAA)n	p.?	GAAn	p.?	AR	CNV+CNV	compound I	/
**AP1606**	***FXN***	18931386	F094	P102	3		Caucasian	Non-EA	NM_000144.5	(GAA)n	p.?	GAAn	p.?	AR	CNV+CNV	compound I	/
**AP1607**	***FXN***	18931386	F095	P103	4		Caucasian	Non-EA	NM_000144.5	(GAA)n	p.?	GAAn	p.?	AR	CNV+CNV	compound I	/
**AP1608**	***FXN***	18931386	F096	P104	5		Caucasian	Non-EA	NM_000144.5	(GAA)n	p.?	GAAn	p.?	AR	CNV+CNV	compound I	/
**AP1609**	***FXN***	18931386	F097	P105	6		Caucasian	Non-EA	NM_000144.5	(GAA)n	p.?	GAAn	p.?	AR	CNV+CNV	compound I	/
**AP1610**	***FXN***	18931386	F098	P106	7		Caucasian	Non-EA	NM_000144.5	(GAA)n	p.?	GAAn	p.?	AR	CNV+CNV	compound I	/
**AP1611**	***FXN***	18931386	F099	P107	8		Caucasian	Non-EA	NM_000144.5	(GAA)n	p.?	GAAn	p.?	AR	CNV+CNV	compound I	/
**AP1612**	***FXN***	18931386	F100	P108	9		Caucasian	Non-EA	NM_000144.5	(GAA)n	p.?	GAAn	p.?	AR	CNV+CNV	compound I	/
**AP1613**	***FXN***	18931386	F101	P109	10		Caucasian	Non-EA	NM_000144.5	(GAA)n	p.?	GAAn	p.?	AR	CNV+CNV	compound I	/
**AP1614**	***FXN***	18931386	F102	P110	11		Caucasian	Non-EA	NM_000144.5	(GAA)n	p.?	GAAn	p.?	AR	CNV+CNV	compound I	/
**AP1615**	***FXN***	18931386	F103	P111	12		Caucasian	Non-EA	NM_000144.5	(GAA)n	p.?	GAAn	p.?	AR	CNV+CNV	compound I	/
**AP1616**	***FXN***	18931386	F104	P112	13		Caucasian	Non-EA	NM_000144.5	(GAA)n	p.?	GAAn	p.?	AR	CNV+CNV	compound I	/
**AP1617**	***FXN***	18931386	F105	P113	14		Caucasian	Non-EA	NM_000144.5	(GAA)n	p.?	GAAn	p.?	AR	CNV+CNV	compound I	/
**AP1618**	***FXN***	18931386	F106	P114	15		Caucasian	Non-EA	NM_000144.5	(GAA)n	p.?	GAAn	p.?	AR	CNV+CNV	compound I	/
**AP1619**	***FXN***	18931386	F107	P115	16		Caucasian	Non-EA	NM_000144.5	(GAA)n	p.?	GAAn	p.?	AR	CNV+CNV	compound I	/
**AP1620**	***FXN***	18931386	F108	P116	17		Caucasian	Non-EA	NM_000144.5	(GAA)n	p.?	GAAn	p.?	AR	CNV+CNV	compound I	/
**AP1621**	***FXN***	18931386	F109	P117	18		Caucasian	Non-EA	NM_000144.5	(GAA)n	p.?	GAAn	p.?	AR	CNV+CNV	compound I	/
**AP1622**	***FXN***	18931386	F110	P118	19		Caucasian	Non-EA	NM_000144.5	(GAA)n	p.?	GAAn	p.?	AR	CNV+CNV	compound I	/
**AP1623**	***FXN***	18931386	F111	P119	20		Caucasian	Non-EA	NM_000144.5	(GAA)n	p.?	c.157delC	p.?	AR	CNV+T	compound I	/
**AP1624**	***FXN***	18931386	F112	P120	21		Caucasian	Non-EA	NM_000144.5	(GAA)n	p.?	c.381_384del	p.?	AR	CNV+T	compound I	/
**AP1625**	***FXN***	17703324	F113	P121	F1	P785	Caucasian	Non-EA	NM_000144.5	(GAA)n	p.?	c.100delG	p.Ala34Profs*	AR	CNV+T	compound I	Male
**AP1626**	***FXN***	17703324	F114	P122	F2	P840	Caucasian	Non-EA	NM_000144.5	(GAA)n	p.?	c.104delC	p.Pro35Hisfs*	AR	CNV+T	compound I	Female
**AP1627**	***FXN***	17703324	F115	P123	F3	P550	Caucasian	Non-EA	NM_000144.5	(GAA)n	p.?	c.157delC	p.Arg53Alafs*	AR	CNV+T	compound I	Male
**AP1628**	***FXN***	17703324	F116	P124	F4	P1250	Caucasian	Non-EA	NM_000144.5	(GAA)n	p.?	c.157delC	p.Arg53Alafs*	AR	CNV+T	compound I	Female
**AP1629**	***FXN***	17703324	F117	P125	F5	P1278	Caucasian	Non-EA	NM_000144.5	(GAA)n	p.?	c.157delC	p.Arg53Alafs*	AR	CNV+T	compound I	Female
**AP1630**	***FXN***	17703324	F118	P126	F6	P1649	Caucasian	Non-EA	NM_000144.5	(GAA)n	p.?	c.157delC	p.Arg53Alafs*	AR	CNV+T	compound I	Female
**AP1631**	***FXN***	17703324	F119	P127	F7	P585	Caucasian	Non-EA	NM_000144.5	(GAA)n	p.?	c.354C>G	p.Tyr118*	AR	CNV+T	compound I	Male
**AP1632**	***FXN***	17703324	F120	P128	F8	P469	Caucasian	Non-EA	NM_000144.5	(GAA)n	p.?	c.460A>T	p.Ile154Phe	AR	CNV+M	compound I	Male
**AP1633**	***FXN***	17703324	F121	P129	F9	P525	Caucasian	Non-EA	NM_000144.5	(GAA)n	p.?	c.517T>G	p.Trp173Gly	AR	CNV+M	compound I	Female
**AP1634**	***FXN***	17703324	F122	P130	F10	P127	Caucasian	Non-EA	NM_000144.5	(GAA)n	p.?	c.517T>G	p.Trp173Gly	AR	CNV+M	compound I	Male
**AP1635**	***FXN***	17703324	F122	P131	F10	P614	Caucasian	Non-EA	NM_000144.5	(GAA)n	p.?	c.517T>G	p.Trp173Gly	AR	CNV+M	compound I	Male
**AP1636**	***FXN***	17703324	F122	P132	F10	P857	Caucasian	Non-EA	NM_000144.5	(GAA)n	p.?	c.517T>G	p.Trp173Gly	AR	CNV+M	compound I	Female
**AP1637**	***FXN***	15376485	F123	P133	/	Patient 3:1	Caucasian	Non-EA	NM_000144.5	(GAA)n	p.?	c.11_12delTC	p.Leu4Argfs*8	AR	CNV+T	compound I	Male
**AP1638**	***FXN***	15376485	F123	P134	/	Patient 3:3	Caucasian	Non-EA	NM_000144.5	(GAA)n	p.?	c.11_12delTC	p.Leu4Argfs*8	AR	CNV+T	compound I	Female
**AP1639**	***FXN***	15340363	F124	P135	/	P1	Caucasian	Non-EA	NM_000144.5	(GAA)n	p.?	g.120032_122	p.?	AR	CNV+CNV	compound I	Female
**AP1640**	***FXN***	15340363	F125	P136	/	P2	Caucasian	Non-EA	NM_000144.5	(GAA)n	p.?	c.438C>G	p.Asn146Lys	AR	CNV+M	compound I	Female
**AP1641**	***FXN***	15340363	F126	P137	/	P3	Caucasian	Non-EA	NM_000144.5	(GAA)n	p.?	c.557T>G	p.Leu186Arg	AR	CNV+M	compound I	Male
**AP1642**	***FXN***	12112211	F127	P138	/		Caucasian	Non-EA	NM_000144.5	(GAA)n	p.?	GAAn	p.?	AR	CNV+CNV	compound I	Male
**AP1643**	***FXN***	11843702	F128	P139	/	Case 1	Caucasian	Non-EA	NM_000144.5	(GAA)n	p.?	c.389G>T	p.Gly130Val	AR	CNV+M	compound I	Male
**AP1644**	***FXN***	11843702	F129	P140	/	Case 2	Caucasian	Non-EA	NM_000144.5	(GAA)n	p.?	c.389G>T	p.Gly130Val	AR	CNV+M	compound I	Female
**AP1645**	***FXN***	10982187	F130	P141	/	96-230	Caucasian	Non-EA	NM_000144.5	(GAA)n	p.?	c.464G>A	p.Trp155*	AR	CNV+M	compound I	Female
**AP1646**	***FXN***	10982187	F131	P142	/	AF559	Caucasian	Non-EA	NM_000144.5	(GAA)n	p.?	c.296_297ins	p.Glu100Argfs	AR	CNV+T	compound I	Male
**AP1647**	***FXN***	10982187	F132	P143	/	AF214	Caucasian	Non-EA	NM_000144.5	(GAA)n	p.?	c.118delC	p.Arg40Valfs*	AR	CNV+T	compound I	/
**AP1648**	***FXN***	10982187	F133	P144	/	AF3	Caucasian	Non-EA	NM_000144.5	(GAA)n	p.?	c.385-2A>G	p.?	AR	CNV+T	compound I	/
**AP1649**	***FXN***	10766903	F134	P145	/	Patient 1	Caucasian	Non-EA	NM_000144.5		p.?	c.493C>T	p.Arg165Cys	AR	CNV+M	compound I	Male
**AP1650**	***FXN***	10766903	F135	P146	/	Patient 2	Caucasian	Non-EA	NM_000144.5		p.?	c.165+5G>C	p.?	AR	CNV+T	compound I	Male
**AP1651**	***FXN***	10732799	F136	P147	/	Patient 1	Caucasian	Non-EA	NM_000144.5	(GAA)n	p.?	c.482+2T>G	p.?	AR	CNV+T	compound I	Male
**AP1652**	***FXN***	10732799	F137	P148	/	Patient 2	Caucasian	Non-EA	NM_000144.5	(GAA)n	p.?	c.545T>A	p.Leu182His	AR	CNV+M	compound I	Male
**AP1653**	***FXN***	10732799	F138	P149	/	Patient 3	Caucasian	Non-EA	NM_000144.5	(GAA)n	p.?	c.493C>T	p.Arg165Cys	AR	CNV+M	compound I	Male
**AP1654**	***FXN***	10732799	F139	P150	/	Patient 4	Caucasian	Non-EA	NM_000144.5	(GAA)n	p.?	c.389G>T	p.Gly130Val	AR	CNV+M	compound I	Male
**AP1655**	***FXN***	10732799	F140	P151	/	Patient 5	Caucasian	Non-EA	NM_000144.5	(GAA)n	p.?	c.389G>T	Gly130Val	AR	CNV+M	compound I	Male
**AP1656**	***FXN***	10399865	F141	P152	1	1	Caucasian	Non-EA	NM_000144.5	(GAA)n	p.?	GAAn	p.?	AR	CNV+CNV	compound I	Female
**AP1657**	***FXN***	10399865	F142	P153	2	2	Caucasian	Non-EA	NM_000144.5	(GAA)n	p.?	GAAn	p.?	AR	CNV+CNV	compound I	Male
**AP1658**	***FXN***	10399865	F143	P154	3	3	Caucasian	Non-EA	NM_000144.5	(GAA)n	p.?	GAAn	p.?	AR	CNV+CNV	compound I	Female
**AP1659**	***FXN***	10399865	F144	P155	4	4	Caucasian	Non-EA	NM_000144.5	(GAA)n	p.?	GAAn	p.?	AR	CNV+CNV	compound I	Female
**AP1660**	***FXN***	10399865	F145	P156	5	5	Caucasian	Non-EA	NM_000144.5	(GAA)n	p.?	GAAn	p.?	AR	CNV+CNV	compound I	Female
**AP1661**	***FXN***	10399865	F145	P157	5	6	Caucasian	Non-EA	NM_000144.5	(GAA)n	p.?	GAAn	p.?	AR	CNV+CNV	compound I	Male
**AP1662**	***FXN***	10399865	F145	P158	5	7	Caucasian	Non-EA	NM_000144.5	(GAA)n	p.?	GAAn	p.?	AR	CNV+CNV	compound I	Male
**AP1663**	***FXN***	10399865	F146	P159	6	8	Caucasian	Non-EA	NM_000144.5	(GAA)n	p.?	GAAn	p.?	AR	CNV+CNV	compound I	Male
**AP1664**	***FXN***	10399865	F147	P160	7	9	Caucasian	Non-EA	NM_000144.5	(GAA)n	p.?	GAAn	p.?	AR	CNV+CNV	compound I	Male
**AP1665**	***FXN***	10399865	F148	P161	8	10	Caucasian	Non-EA	NM_000144.5	(GAA)n	p.?	GAAn	p.?	AR	CNV+CNV	compound I	Female
**AP1666**	***FXN***	10399865	F148	P162	8	11	Caucasian	Non-EA	NM_000144.5	(GAA)n	p.?	GAAn	p.?	AR	CNV+CNV	compound I	Male
**AP1667**	***FXN***	9989622	F149	P163	/	1	Caucasian	Non-EA	NM_000144.5	(GAA)n	p.?	c.1A>C	p.M1?	AR	CNV+T	compound I	Female
**AP1668**	***FXN***	9989622	F150	P164	/	2	Caucasian	Non-EA	NM_000144.5	(GAA)n	p.?	c.2T>C	p.M1?	AR	CNV+T	compound I	Male
**AP1669**	***FXN***	9989622	F151	P165	/	3	Caucasian	Non-EA	NM_000144.5	(GAA)n	p.?	c.3G>T	p.M1?	AR	CNV+T	compound I	Male
**AP1670**	***FXN***	9989622	F152	P166	/	4	Caucasian	Non-EA	NM_000144.5	(GAA)n	p.?	c.157delC	p.Arg53Alafs*	AR	CNV+T	compound I	Female
**AP1671**	***FXN***	9989622	F153	P167	/	5	Caucasian	Non-EA	NM_000144.5	(GAA)n	p.?	c.157dupC	p.Arg53Profs*	AR	CNV+T	compound I	Female
**AP1672**	***FXN***	9989622	F154	P168	/	6a	Caucasian	Non-EA	NM_000144.5	(GAA)n	p.?	c.317T>G	p.Leu106*	AR	CNV+T	compound I	Female
**AP1673**	***FXN***	9989622	F154	P169	/	6b	Caucasian	Non-EA	NM_000144.5	(GAA)n	p.?	c.317T>G	p.Leu106*	AR	CNV+T	compound I	Male
**AP1674**	***FXN***	9989622	F155	P170	/	7	Caucasian	Non-EA	NM_000144.5	(GAA)n	p.?	c.482+3delA	p.?	AR	CNV+T	compound I	Male
**AP1675**	***FXN***	9989622	F156	P171	/	8	Caucasian	Non-EA	NM_000144.5	(GAA)n	p.?	c.364G>T	p.Asp122Tyr	AR	CNV+M	compound I	Female
**AP1676**	***FXN***	9989622	F157	P172	/	9a	Caucasian	Non-EA	NM_000144.5	(GAA)n	p.?	c.389G>T	p.Gly130Val	AR	CNV+M	compound I	Male
**AP1677**	***FXN***	9989622	F157	P173	/	9b	Caucasian	Non-EA	NM_000144.5	(GAA)n	p.?	c.389G>T	p.Gly130Val	AR	CNV+M	compound I	Female
**AP1678**	***FXN***	9989622	F158	P174	/	10a	Caucasian	Non-EA	NM_000144.5	(GAA)n	p.?	c.389G>T	p.Gly130Val	AR	CNV+M	compound I	Female
**AP1679**	***FXN***	9989622	F158	P175	/	10b	Caucasian	Non-EA	NM_000144.5	(GAA)n	p.?	c.389G>T	p.Gly130Val	AR	CNV+M	compound I	Male
**AP1680**	***FXN***	9989622	F158	P176	/	10c	Caucasian	Non-EA	NM_000144.5	(GAA)n	p.?	c.389G>T	p.Gly130Val	AR	CNV+M	compound I	Female
**AP1681**	***FXN***	9989622	F159	P177	/	11	Caucasian	Non-EA	NM_000144.5	(GAA)n	p.?	c.389G>T	p.Gly130Val	AR	CNV+M	compound I	Female
**AP1682**	***FXN***	9989622	F160	P178	/	12a	Caucasian	Non-EA	NM_000144.5	(GAA)n	p.?	c.460A>T	p.Ile154Phe	AR	CNV+M	compound I	Female
**AP1683**	***FXN***	9989622	F160	P179	/	12b	Caucasian	Non-EA	NM_000144.5	(GAA)n	p.?	c.460A>T	p.Ile154Phe	AR	CNV+M	compound I	Female
**AP1684**	***FXN***	9989622	F161	P180	/	13	Caucasian	Non-EA	NM_000144.5	(GAA)n	p.?	c.460A>T	p.Ile154Phe	AR	CNV+M	compound I	Female
**AP1685**	***FXN***	9989622	F162	P181	/	14a	Caucasian	Non-EA	NM_000144.5	(GAA)n	p.?	c.460A>T	p.Ile154Phe	AR	CNV+M	compound I	Female
**AP1686**	***FXN***	9989622	F162	P182	/	14b	Caucasian	Non-EA	NM_000144.5	(GAA)n	p.?	c.460A>T	p.Ile154Phe	AR	CNV+M	compound I	Male
**AP1687**	***FXN***	9989622	F163	P183	/	15	Caucasian	Non-EA	NM_000144.5	(GAA)n	p.?	c.467T>C	p.Leu156Pro	AR	CNV+M	compound I	Female
**AP1688**	***FXN***	9989622	F164	P184	/	16	Caucasian	Non-EA	NM_000144.5	(GAA)n	p.?	c.517T>G	p.Trp173Gly	AR	CNV+M	compound I	Female
**AP1689**	***FXN***	9989622	F165	P185	/	17	Caucasian	Non-EA	NM_000144.5	(GAA)n	p.?	c.517T>G	p.Trp173Gly	AR	CNV+M	compound I	Female
**AP1690**	***FXN***	9989622	F166	P186	/	18	Caucasian	Non-EA	NM_000144.5	(GAA)n	p.?	c.544C>T	p.Leu182Phe	AR	CNV+M	compound I	Male
**AP1691**	***FXN***	9989622	F167	P187	/	19	Caucasian	Non-EA	NM_000144.5	(GAA)n	p.?	c.548A>G	p.His183Arg	AR	CNV+M	compound I	Female
**AP1692**	***FXN***	9539351	F168	P188	/	Case 1	Caucasian	Non-EA	NM_000144.5	(GAA)n	p.?	GAAn	p.?	AR	CNV+CNV	homo	Male
**AP1693**	***FXN***	9539351	F169	P189	/	Case 2	Caucasian	Non-EA	NM_000144.5	(GAA)n	p.?	GAAn	p.?	AR	CNV+CNV	homo	Male
**AP1694**	***IBA57***	34709542	F001	P001	/	Patient 1	Chinese	EA	NM_00101086	c.189delC	p.Asp65Thrfs*7	c.286T>C	p.Tyr96His	AR	M+T	compound I	Male
**AP1695**	***IBA57***	34709542	F001	P002	/	Patient 2	Chinese	EA	NM_00101086	c.580A>G	p.Met194Val	c.286T>C	p.Tyr96His	AR	M+M	compound I	Female
**AP1696**	***IBA57***	33890810	F002	P003	/		Japanese	EA	NM_00101086	c.49_67dup	p.Leu23Profs*61	c.310G>T	p.Gly104Cys	AR	M+T	compound I	Female
**AP1697**	***IBA57***	32348839	F003	P004	/		Chinese	EA	NM_00101086	c.697C > T	p.Arg233*	c.286T>C	p.Tyr96His	AR	M+T	compound I	/
**AP1698**	***IBA57***	32348839	F004	P005	/		Caucasian	Non-EA	NM_00101086	c.588_589del	p.Arg197Alafs*6	c.286T>C	p.Tyr96His	AR	M+T	compound I	/
**AP1699**	***IBA57***	32180488	F005	P006	/		Caucasian	Non-EA	NM_00101086	c.738C>g	p.Asn246Lys	c.802C>T	p.Arg268Cys	AR	M+M	compound I	/
**AP1700**	***IBA57***	30770271	F006	P007	/	Case 1	Chinese	EA	NM_00101086	c.286T>C	p.Tyr96His	c.697C>T	p.Arg233*	AR	M+T	compound I	Male
**AP1701**	***IBA57***	30770271	F007	P008	/	Case 2	Chinese	EA	NM_00101086	c.286T>C	p.Tyr96His	c.316A>G	p.Thr106Ala	AR	M+M	compound I	Female
**AP1702**	***IBA57***	30770271	F008	P009	/	Case 3	Chinese	EA	NM_00101086	c.286T>C	p.Tyr96His	c.316A>G	p.Thr106Ala	AR	M+M	compound I	Female
**AP1703**	***IBA57***	30770271	F009	P010	/	Case 4	Chinese	EA	NM_00101086	c.286T>C	p.Tyr96His	c.697C>T	p.Arg233*	AR	M+T	compound I	Male
**AP1704**	***IBA57***	30770271	F010	P011	/	Case 5	Chinese	EA	NM_00101086	c.22C>T	p.Arg8*	c.286T>C	p.Tyr96His	AR	M+T	compound I	Female
**AP1705**	***IBA57***	30770271	F011	P012	/	Case 6	Chinese	EA	NM_00101086	c.286T>C	p.Tyr96His	c.307C>T	p.Gln103*	AR	M+T	compound I	Female
**AP1706**	***IBA57***	30770271	F012	P013	/	Case 7	Chinese	EA	NM_00101086	c.286T>C	p.Tyr96His	c.307C>T	p.Gln103*	AR	M+T	compound I	Male
**AP1707**	***IBA57***	30770271	F013	P014	/	Case 8	Chinese	EA	NM_00101086	c.286T>C	p.Tyr96His	c.754G>T	p.Gly252Cys	AR	M+M	compound I	Male
**AP1708**	***IBA57***	30770271	F014	P015	/	Case 9	Chinese	EA	NM_00101086	c.286T>C	p.Tyr96His	c.697C>T	p.Arg233*	AR	M+T	compound I	Male
**AP1709**	***IBA57***	30770271	F015	P016	/	Case 10	Chinese	EA	NM_00101086	c.701A>G	p.Asp234Gly	c.782T>C	p.Ile261Thr	AR	M+M	compound I	Female
**AP1710**	***IBA57***	30770271	F016	P017	/	Case 11	Chinese	EA	NM_00101086	c.286T>C	p.Tyr96His	c.697C>T	p.Arg233*	AR	M+T	compound I	Female
**AP1711**	***IBA57***	30770271	F017	P018	/	Case 12	Chinese	EA	NM_00101086	c.286T>C	p.Tyr96His	c.188G>A	p.Gly63Asp	AR	M+M	compound I	Female
**AP1712**	***IBA57***	30770271	F018	P019	/	Case 13	Chinese	EA	NM_00101086	c.286T>C	p.Tyr96His	c.1053G>A	p.Trp351*	AR	M+T	compound I	Male
**AP1713**	***IBA57***	30770271	F019	P020	/	Case 14	Chinese	EA	NM_00101086	c.286T>C	p.Tyr96His	c.588_589del	p.Arg197Alafs	AR	M+T	compound I	Female
**AP1714**	***IBA57***	30770271	F020	P021	/	Case 15	Chinese	EA	NM_00101086	c.286T>C	p.Tyr96His	c.522_523del	p.Ser174Serfs	AR	M+T	compound I	Male
**AP1715**	***IBA57***	30770271	F021	P022	/	Case 16	Chinese	EA	NM_00101086	c.307C>T	p.Gln103*	c.236C>T	p.Pro79Leu	AR	M+T	compound I	Female
**AP1716**	***IBA57***	30770271	F022	P023	/	Case 17	Chinese	EA	NM_00101086	c.286T>C	p.Tyr96His	c.188G>A	p.Gly630Asp	AR	M+M	compound I	Female
**AP1717**	***IBA57***	30258207	F023	P024	family 1	II-1	Sepharadi Jewish	Non-EA	NM_00101086	c.335T>C	p.Leu112Ser	c.588dup	p.Arg197Alafs	AR	M+T	compound I	Male
**AP1718**	***IBA57***	30258207	F023	P025	family 1	II-3	Sepharadi Jewish	Non-EA	NM_00101086	c.335T>C	p.Leu112Ser	c.588dup	p.Arg197Alafs	AR	M+T	compound I	Male
**AP1719**	***IBA57***	30258207	F024	P026	family 2	II-1	Caucasian	Non-EA	NM_00101086	c.386A>T	p.Asp129Val	c.731A>C	p.Glu244Ala	AR	M+M	compound I	Male
**AP1720**	***IBA57***	29353736	F025	P027	/	MLE13	Caucasian	Non-EA	NM_00101086	c.738C>G	p. Asn246Lys	c.802C>T	p. Arg268Cys	AR	M+M	compound I	Male
**AP1721**	***IBA57***	29353736	F026	P028	/	MLE14	Caucasian	Non-EA	NM_00101086	c.706C>T	p. Pro236Ser	c.656A>G	p.Tyr219Cys	AR	M+M	compound I	Male
**AP1722**	***IBA57***	28913435	F027	P029	/	P1	Caucasian	Non-EA	NM_00101086	c.323A>C	p.Tyr108Ser	c.940C>T	p.Gln314*	AR	M+T	compound I	Female
**AP1723**	***IBA57***	28913435	F027	P030	/	P2	Caucasian	Non-EA	NM_00101086	c.323A>C	p.Tyr108Ser	c.940C>T	p.Gln314*	AR	M+T	compound I	Female
**AP1724**	***IBA57***	28913435	F028	P031	/	P3	Caucasian	Non-EA	NM_00101086	c.323A>C	p.Tyr108Ser	c.150C>A	p.Cys50*	AR	M+T	compound I	Female
**AP1725**	***IBA57***	28803783	F029	P032	/	P9	Caucasian	Non-EA	NM_00101086	c.335T>G	p.Leu112Trp	c.437G>C	p.Arg146Pro	AR	M+M	compound I	Male
**AP1726**	***IBA57***	28803783	F030	P033	/	P10	Caucasian	Non-EA	NM_00101086	c.316A>G	p.Thr106Ala	c.738C>G	p.Asn246Lys	AR	M+M	compound I	Female
**AP1727**	***IBA57***	28671726	F031	P034	/	Case 1	Caucasian	Non-EA	NM_00101086	c.286T>C	p.Tyr96His	c.316A>G	p.Thr106Ala	AR	M+M	compound I	/
**AP1728**	***IBA57***	28671726	F032	P035	/	Case 2	Caucasian	Non-EA	NM_00101086	c.286T>C	p.Tyr96His	c.316A>G	p.Thr106Ala	AR	M+M	compound I	/
**AP1729**	***IBA57***	28671726	F033	P036	/	Case 3	Caucasian	Non-EA	NM_00101086	c.701A>G	p.Asp234Gly	c.782T>C	p.Ile261Thr	AR	M+M	compound I	/
**AP1730**	***IBA57***	28671726	F034	P037	/	Case 4	Caucasian	Non-EA	NM_00101086	c.286T>C	p.Tyr96His	c.697C>T	p.Arg233*	AR	M+T	compound I	/
**AP1731**	***IBA57***	28671726	F035	P038	/	Case 5	Caucasian	Non-EA	NM_00101086	c.286T>C	p.Tyr96His	c.697C>T	p.Arg233*	AR	M+T	compound I	/
**AP1732**	***IBA57***	28671726	F036	P039	/	Case 6	Caucasian	Non-EA	NM_00101086	c.286T>C	p.Tyr96His	c.188G>A	p.Gly63Asp	AR	M+M	compound I	/
**AP1733**	***IBA57***	28671726	F037	P040	/	Case 7	Caucasian	Non-EA	NM_00101086	c.286T>C	p.Tyr96His	c.307C>T	p.Gln103*	AR	M+T	compound I	/
**AP1734**	***IBA57***	28671726	F038	P041	/	Case 8	Caucasian	Non-EA	NM_00101086	c.286T>C	p.Tyr96His	c.307C>T	p.Gln103*	AR	M+T	compound I	/
**AP1735**	***IBA57***	28671726	F039	P042	/	Case 9	Caucasian	Non-EA	NM_00101086	c.286T>C	p.Tyr96His	c.697C>T	p.Arg233*	AR	M+T	compound I	/
**AP1736**	***IBA57***	28671726	F040	P043	/	Case 10	Caucasian	Non-EA	NM_00101086	c.286T>C	p.Tyr96His	c.754G>T	p.Gly252Cys	AR	M+M	compound I	/
**AP1737**	***IBA57***	28671726	F041	P044	/	Case 11	Caucasian	Non-EA	NM_00101086	c.286T>C	p.Tyr96His	c.22C>T	p.Arg8*	AR	M+T	compound I	/
**AP1738**	***IBA57***	27785568	F042	P045	/	P1	Caucasian	Non-EA	NM_00101086	c.586T>G	p.Trp196Gly	c.686C>T	p.Pro229Leu	AR	M+M	compound I	Female
**AP1739**	***IBA57***	27785568	F043	P046	/	P2	Caucasian	Non-EA	NM_00101086	c.87ins_GCCCAAGGT	p.R30Afs*46	c.313C>T	p.Arg105Trp	AR	M+T	compound I	Male
**AP1740**	***IBA57***	27785568	F044	P047	/	P3	Caucasian	Non-EA	NM_00101086	c.706C>T	p. Pro236Ser	c.706C>T	p. Pro236Ser	AR	M+M	homo	Female
**AP1741**	***IBA57***	27785568	F045	P048	/	P4	Caucasian	Non-EA	NM_00101086	c.316A>G	p.Thr106Ala	c.757G>C	p.Val253Leu	AR	M+M	compound I	Female
**AP1742**	***IBA57***	25971455	F046	P049	/		Caucasian	Non-EA	NM_00101086	c.436C>T	p.Arg146Trp	c.436C>T	p.Arg146Trp	AR	M+M	homo	Male
**AP1743**	***IBA57***	25609768	F047	P050	/	III-1	Caucasian	Non-EA	NM_00101086	c.678A>G	p.?	c.678A>G	p.?	AR	T+T	homo	Female
**AP1744**	***IBA57***	25609768	F047	P051	/	III-5	Caucasian	Non-EA	NM_00101086	c.678A>G	p.?	c.678A>G	p.?	AR	T+T	homo	Male
**AP1745**	***IBA57***	25609768	F047	P052	/	III-6	Caucasian	Non-EA	NM_00101086	c.678A>G	p.?	c.678A>G	p.?	AR	T+T	homo	Male
**AP1746**	***IBA57***	25609768	F047	P053	/	IV-1	Caucasian	Non-EA	NM_00101086	c.678A>G	p.?	c.678A>G	p.?	AR	T+T	homo	Male
**AP1747**	***IBA57***	25609768	F047	P054	/	IV-2	Caucasian	Non-EA	NM_00101086	c.678A>G	p.?	c.678A>G	p.?	AR	T+T	homo	Female
**AP1748**	***IBA57***	25609768	F047	P055	/	IV-3	Caucasian	Non-EA	NM_00101086	c.678A>G	p.?	c.678A>G	p.?	AR	T+T	homo	Male
**AP1749**	***IBA57***	25609768	F047	P056	/	IV-4	Caucasian	Non-EA	NM_00101086	c.678A>G	p.?	c.678A>G	p.?	AR	T+T	homo	Female
**AP1750**	***IBA57***	25609768	F047	P057	/	IV-7	Caucasian	Non-EA	NM_00101086	c.678A>G	p.?	c.678A>G	p.?	AR	T+T	homo	Male
**AP1751**	***IBA57***	25609768	F047	P058	/	IV-8	Caucasian	Non-EA	NM_00101086	c.678A>G	p.?	c.678A>G	p.?	AR	T+T	homo	Female
**AP1752**	***IBA57***	25609768	F047	P059	/	IV-10	Caucasian	Non-EA	NM_00101086	c.678A>G	p.?	c.678A>G	p.?	AR	T+T	homo	Male
**AP1753**	***IBA57***	25609768	F047	P060	/	V-2	Caucasian	Non-EA	NM_00101086	c.678A>G	p.?	c.678A>G	p.?	AR	T+T	homo	Male
**AP1754**	***IBA57***	23462291	F048	P061	/		Caucasian	Non-EA	NM_00101086	c.941A>C	p.Gln314Pro	c.941A>C	p.Gln314Pro	AR	M+M	homo	Male
**AP1755**	***IBA57***	23462291	F048	P062	/		Caucasian	Non-EA	NM_00101086	c.941A>C	p.Gln314Pro	c.941A>C	p.Gln314Pro	AR	M+M	homo	Female
**AP1756**	***MCAT***	33918393	F001	P001	/	II-1	French	Non-EA		c.1039G>A	p.Glu347Lys	c.424-2A>G	p.?	AR	M+T	Compd het	Female
**AP1757**	***MCAT***	31915829	F002	P002	/	V1	Japanese	EA		c.242T>G	p.Leu81Arg	c.634C>T	p.Arg212Trp	AR	M+M	Compd het	Male
**AP1758**	***MCAT***	31915829	F002	P003	/	V2	Japanese	EA		c.242T>G	p.Leu81Arg	c.634C>T	p.Arg212Trp	AR	M+M	Compd het	Male
**AP1759**	***MECR***	27817865	F001	P001	Family A	1	Ashkenazi Jewish	Non-EA		c.695G>A	p.Gly232Glu	c.855T>G	p.Tyr285*	AR	M+T	Compound	Male
**AP1760**	***MECR***	27817865	F002	P002	Family B	2	Ashkenazi Jewish	Non-EA		c.695G>A	p.Gly232Glu	c.830+2insT	p.?	AR	M+T	Compound	Male
**AP1761**	***MECR***	27817865	F003	P003	Family C	3	Ashkenazi Jewish	Non-EA		c.695G>A	p.Gly232Glu	c.830+2insT	p.?	AR	M+T	Compound	Male
**AP1762**	***MECR***	27817865	F003	P004	Family C	4	Ashkenazi Jewish	Non-EA		c.695G>A	p.Gly232Glu	c.830+2insT	p.?	AR	M+T	Compound	Male
**AP1763**	***MECR***	27817865	F004	P005	Family D	5	Tunisian	Non-EA		c.854A>G	p.Tyr285Cys	c.854A>G	p.Tyr285Cys	AR	M+M	Homo	Female
**AP1764**	***MECR***	27817865	F005	P006	Family E	6	Anglo-Saxon	Non-EA		c.772C>T	p.Arg258Trp	c.247_250del	p.Asn83Hisfs*	AR	M+T	Compound	Male
**AP1765**	***MECR***	27817865	F005	P007	Family E	7	Anglo-Saxon	Non-EA		c.772C>T	p.Arg258Trp	c.247_250del	p. Asn83Hisfs*	AR	M+T	Compound	Male
**AP1766**	***MECR***	33401012	F006	P009	Family G	9	Chinese	EA		c.910G>T	p.Asp304Tyr	c.910G>T	p.Asp304Tyr	AR	M+M	Homo	Male
**AP1767**	***MECR***	32313153	F007	P010	/	6	Caucasian	Non-EA		c.247_250del	p.Asn83Hisfs*4	c.772C>T	p.Arg258Trp	AR	M+T	Compound	/
**AP1768**	***MECR***	31137067	F008	P011	/	/	Turkish	Non-EA		c.772C>T	p.Arg258Trp	c.1009C>T	p.Arg337*	AR	M+T	Compound	/
**AP1769**	***MECR***	33098801	F009	P012	/	IS-DYS-434	Caucasian	Non-EA		c.772C>T	p.Arg258Trp	c.553delG	p.Asp185Ilefs*	AR	M+T	Compound	/
**AP1770**	***MECR***	32445240	F010	P013	/	34	Caucasian	Non-EA		c.695G>A	p.Gly232Glu	c.830+2insT	p.?	AR	M+T	Compound	/
**AP1771**	***MECR***	37734847	F011	P014	Family I	II:1	Italian	Non-EA		c.772C>T	p.Arg258Trp	c.772C>T	p.Arg258Trp	AR	M+M	Homo	/
**AP1772**	***MECR***	37734847	F011	P015	Family I	II:2	Italian	Non-EA		c.772C>T	p.Arg258Trp	c.772C>T	p.Arg258Trp	AR	M+M	Homo	/
**AP1773**	***MFN2***	16437557	F001	P001	CMT-5	III:1	Africa American	Non-EA	NM_014874.3	c.280C>T	p.Arg94Trp	/	/	AD	M	heter	/
**AP1774**	***MFN2***	16437557	F002	P002	CMT-5	III:1	Africa American	Non-EA	NM_014874.3	c.280C>T	p.Arg94Trp	/	/	AD	M	heter	/
**AP1775**	***MFN2***	16437557	F003	P003	CMT-52		European	Non-EA	NM_014874.3	c.617C>T	p.Thr206Ile	/	/	AD	M	heter	/
**AP1776**	***MFN2***	16437557	F004	P004	CMT-52		European	Non-EA	NM_014874.3	c.617C>T	p.Thr206Ile	/	/	AD	M	heter	/
**AP1777**	***MFN2***	16437557	F005	P005	CMT-273		European	Non-EA	NM_014874.3	c.827A>G	p.Gln276Arg	/	/	AD	M	heter	/
**AP1778**	***MFN2***	16437557	F006	P006	CMT-506		European	Non-EA	NM_014874.3	c.1081C>T	p.His361Tyr	/	/	AD	M	heter	/
**AP1779**	***MFN2***	16437557	F007	P007	CMT-11		European	Non-EA	NM_014874.3	c.1090C>T	p.Arg364Trp	/	/	AD	M	heter	/
**AP1780**	***MFN2***	16437557	F008	P008	CMT-11		European	Non-EA	NM_014874.3	c.1090C>T	p.Arg364Trp	/	/	AD	M	heter	/
**AP1781**	***MFN2***	16437557	F009	P009	CMT-11		European	Non-EA	NM_014874.3	c.1090C>T	p.Arg364Trp	/	/	AD	M	heter	/
**AP1782**	***MFN2***	16437557	F010	P010	DUK2158		European	Non-EA	NM_014874.3	c.1252C>T	p.Arg418*	/	/	AD	M	heter	/
**AP1783**	***MFN2***	16762064	F011	P011	/	1	German	Non-EA	NM_014874.3	c.380G>T	p.Gla127Val	/	/	AD	M	compound I	Female
**AP1784**	***MFN2***	16762064	F012	P012	/	2	German	Non-EA	NM_014874.3	c.1128G>A	p.Met376Ile	/	/	AD	M	compound I	Female
**AP1785**	***MFN2***	16762064	F013	P013	/	3	German	Non-EA	NM_014874.3	c.1040A>T	p.Glu347Val	/	/	AD	M	compound I	Male
**AP1786**	***MFN2***	16762064	F014	P014	/	4	German	Non-EA	NM_014874.3	c.1403G>A	p.Arg468His	/	/	AD	M	compound I	Female
**AP1787**	***MFN2***	16762064	F015	P015	/	5	German	Non-EA	NM_014874.3	c.2113G>A	p.Val705Ile	/	/	AD	M	compound I	Female
**AP1788**	***MFN2***	16762064	F016	P016	/	6	German	Non-EA	NM_014874.3	c.2258dup	p.Gln754Alafs*9	/	/	AD	M	compound I	Male
**AP1789**	***MFN2***	16835246/248	F017	P017	FC48		Korean	EA	NM_014874.3	c.380G>A	p.Gly127Asp	/	/	AD	M	heter	/
**AP1790**	***MFN2***	16835246/248	F018	P018	FC111		Korean	EA	NM_014874.3	c.494A>G	p.His165Arg	/	/	AD	M	heter	/
**AP1791**	***MFN2***	16835246/248	F019	P019	FC52		Korean	EA	NM_014874.3	c.787T>C	p.Ser263Pro	/	/	AD	M	heter	/
**AP1792**	***MFN2***	16835246/248	F020	P020	FC188		Korean	EA	NM_014874.3	c.1085C>T	p.Thr362Met	/	/	AD	M	heter	/
**AP1793**	***MFN2***	16835246/248	F021	P021	FC70		Korean	EA	NM_014874.3	c.1127T>C	p.Met376Thr	/	/	AD	M	heter	/
**AP1794**	***MFN2***	17309650	F022	P022	/	3	Korean	EA	NM_014874.3	c.1048T>C	p.Ser350Pro	/	/	AD	M	compound I	Male
**AP1795**	***MFN2***	17309650	F023	P023	/	4	Korean	EA	NM_014874.3	c.787T>C	p.Ser263Pro	/	/	AD	M	compound I	Male
**AP1796**	***MFN2***	17309650	F024	P024	/	10	Korean	EA	NM_014874.3	c.494A>G	p.His165Arg	/	/	AD	M	compound I	Female
**AP1797**	***MFN2***	17309650	F025	P025	/	11	Korean	EA	NM_014874.3	c.280C>T	p.Arg94Trp	/	/	AD	M	compound I	Female
**AP1798**	***MFN2***	18425620	F026	P026	Family1	patient 1	German	Non-EA	NM_014874.3	c.310 C>T	p.Arg104Trp	/	/	AD	M	heter	Male
**AP1799**	***MFN2***	18425620	F027	P027	Family4	patient 4	German	Non-EA	NM_014874.3	c.310 C>T	p.Arg104Trp	/	/	AD	M	heter	Male
**AP1800**	***MFN2***	18946002	F028	P028	Family 1	III-2	German	Non-EA	NM_014874.3	c.310 C>T	p.Arg104Trp	/	/	AD	M	heter	Male
**AP1801**	***MFN2***	18996695	F029	P029	Family 2		Spanish	Non-EA	NM_014874.3	c.280C>G	p.Arg94Gly	/	/	AD	M	heter	Male
**AP1802**	***MFN2***	20008656	F030	P030	/	1	Caucasian	Non-EA	NM_014874.3	c.280C>T	p.Arg94Trp	/	/	AD	M	compound I	Female
**AP1803**	***MFN2***	20008656	F031	P031	/	2	Caucasian	Non-EA	NM_014874.3	c.280C>T	p.Arg94Trp	/	/	AD	M	compound I	Female
**AP1804**	***MFN2***	20008656	F032	P032	/	3	Caucasian	Non-EA	NM_014874.3	c.310 C>T	p.Arg104Trp			AR	M	compound I	Female
**AP1805**	***MFN2***	20008656	F033	P033	/	4	Caucasian	Non-EA	NM_014874.3	c.310 C>T	p.Arg104Trp	/	/	AD	M	compound I	Female
**AP1806**	***MFN2***	20008656	F034	P034	/	5	Caucasian	Non-EA	NM_014874.3	c.310 C>T	p.Arg104Trp	/	/	AD	M	compound I	Female
**AP1807**	***MFN2***	20008656	F035	P035	/	6	Caucasian	Non-EA	NM_014874.3	c.322G>A	p.Gly108Arg	c.2119C>T	p.Arg707Trp	AR	M+M	compound I	Male
**AP1808**	***MFN2***	20008656	F036	P036	/	7	Caucasian	Non-EA	NM_014874.3	c.322G>A	p.Gly108Arg	c.2119C>T	p.Arg707Trp	AR	M+M	compound I	Female
**AP1809**	***MFN2***	20008656	F037	P037	/	8	Caucasian	Non-EA	NM_014874.3	c.322G>A	p.Gly108Arg	c.2119C>T	p.Arg707Trp	AR	M+M	compound I	Male
**AP1810**	***MFN2***	20008656	F038	P038	/	9	Caucasian	Non-EA	NM_014874.3	c.383A>G	p.His128Arg	/	/	AD	M	compound I	Female
**AP1811**	***MFN2***	20008656	F039	P039	/	10	Caucasian	Non-EA	NM_014874.3	c.467G>T	p.Ser156Ile	/	/	AD	M	compound I	Female
**AP1812**	***MFN2***	20008656	F040	P040	/	11	Caucasian	Non-EA	NM_014874.3	c.491C>T	p.Ala164Val	c.1085C>G	p.Thr362Met	AR	M+M	compound I	Female
**AP1813**	***MFN2***	20008656	F041	P041	/	12	Caucasian	Non-EA	NM_014874.3	c.640G>A	p.Asp214Asn		p.C390R	AR	M	compound I	Male
**AP1814**	***MFN2***	20008656	F042	P042	/	13	Caucasian	Non-EA	NM_014874.3	c.707C>T	p.Thr236Met	/	/	AD	M	compound I	Female
**AP1815**	***MFN2***	20008656	F043	P043	/	14	Caucasian	Non-EA	NM_014874.3	c.730G>A	p.Val244Met	/	/	AD	M	compound I	Male
**AP1816**	***MFN2***	20008656	F044	P044	/	15	Caucasian	Non-EA	NM_014874.3	c.827A>G	p.Gln276Arg	/	/	AD	M	compound I	Male
**AP1817**	***MFN2***	20008656	F045	P045	/	16	Caucasian	Non-EA	NM_014874.3	c.829C>T	p.His277Tyr	/	/	AD	M	compound I	Male
**AP1818**	***MFN2***	20008656	F046	P046	/	17	Caucasian	Non-EA	NM_014874.3	c.1091G>C	p.Arg364Pro	/	/	AD	M	compound I	Female
**AP1819**	***MFN2***	20008656	F047	P047	/	18	Caucasian	Non-EA	NM_014874.3	c.1091G>C	p.Arg364Pro	/	/	AD	M	compound I	Female
**AP1820**	***MFN2***	20008656	F048	P048	/	19	Caucasian	Non-EA	NM_014874.3	c.1091G>A	p.Arg364Gln	/	/	AD	M	compound I	Female
**AP1821**	***MFN2***	20008656	F049	P049	/	20	Caucasian	Non-EA	NM_014874.3	c.1994T>C	p.Phe665Ser	/	/	AD	M	compound I	Female
**AP1822**	***MFN2***	20008656	F050	P050	/	21	Caucasian	Non-EA	NM_014874.3	c.2220G>C	p.Trp740Cys	/	/	AD	M	compound I	Female
**AP1823**	***MFN2***	20008656	F051	P051	/	22	Caucasian	Non-EA	NM_014874.3	c.2220G>C	p.Trp740Cys	/	/	AD	M	compound I	Female
**AP1824**	***MFN2***	20008656	F052	P052	/	23	Caucasian	Non-EA	NM_014874.3	c.2234T>C	p.Leu745Pro	/	/	AD	M	compound I	Male
**AP1825**	***MFN2***	20008656	F053	P053	/	24	Caucasian	Non-EA	NM_014874.3	c.2234T>C	p.Leu745Pro	/	/	AD	M	compound I	Female
**AP1826**	***MFN2***	20008656	F054	P054	/	25	Caucasian	Non-EA	NM_014874.3	c.2240T>C	p.Met747Thr	/	/	AD	M	compound I	Female
**AP1827**	***MFN2***	20350294	F055	P055	1	II-2	Norwegian	Non-EA	NM_014874.3	c.280C>T	p.Arg94Trp	/	/	AD	M	compound I	Female
**AP1828**	***MFN2***	20350294	F055	P056	1	III-1	Norwegian	Non-EA	NM_014874.3	c.280C>T	p.Arg94Trp	/	/	AD	M	compound I	Female
**AP1829**	***MFN2***	20350294	F056	P057	2	II-1	Norwegian	Non-EA	NM_014874.3	c.281G>A	p.Arg94Gln	/	/	AD	M	compound I	Female
**AP1830**	***MFN2***	20350294	F057	P058	3	II-1	Norwegian	Non-EA	NM_014874.3	c.1403G>A	p.Arg468His	/	/	AD	M	compound I	Male
**AP1831**	***MFN2***	20350294	F058	P059	4	II-1	Norwegian	Non-EA	NM_014874.3	c.1709A>G	p.Asn570Ser	/	/	AD	M	compound I	Male
**AP1832**	***MFN2***	20350294	F059	P060	5	III-9	Norwegian	Non-EA	NM_014874.3	c.2113G>A	p.Val705Ile	/	/	AD	M	compound I	Female
**AP1833**	***MFN2***	20350294	F060	P061	6	II-3	Norwegian	Non-EA	NM_014874.3	c.2113G>A	p.Val705Ile	/	/	AD	M	compound I	Male
**AP1834**	***MFN2***	20350294	F061	P062	7	III-3	Norwegian	Non-EA	NM_014874.3	c.2119C>T	p.Arg707Trp	/	/	AD	M	compound I	Male
**AP1835**	***MFN2***	20350294	F062	P063	8	III-2	Norwegian	Non-EA	NM_014874.3	c.2146G>A	p.Ala716Thr	/	/	AD	M	compound I	Male
**AP1836**	***MFN2***	20350294	F063	P064	8	III-4	Norwegian	Non-EA	NM_014874.3	c.2146G>A	p.Ala716Thr	/	/	AD	M	compound I	Male
**AP1837**	***MFN2***	20587496	F064	P065	DUKE 2158		Korean	EA	NM_014874.3	c.1252C>T	p.Arg418*	/	/	AD	M	heter	/
**AP1838**	***MFN2***	20587496/248	F065	P066	FC1	II-3	Korean	EA	NM_014874.3	c.1090C>T	p.Arg364Trp	/	/	AD	M	heter	Female
**AP1839**	***MFN2***	20587496/248	F065	P067	FC1	III-1	Caucasian	Non-EA	NM_014874.3	c.1090C>T	p.Arg364Trp	/	/	AD	M	heter	/
**AP1840**	***MFN2***	20587496/248	F065	P068	FC1	III-2	Caucasian	Non-EA	NM_014874.3	c.1090C>T	p.Arg364Trp	/	/	AD	M	heter	/
**AP1841**	***MFN2***	20587496/248	F066	P069	FC6	5	Korean	EA	NM_014874.3	c.1090C>T	p.Arg364Trp	/	/	AD	M	heter	/
**AP1842**	***MFN2***	20587496/248	F066	P070	FC6	4	Korean	EA	NM_014874.3	c.1090C>T	p.Arg364Trp	/	/	AD	M	heter	/
**AP1843**	***MFN2***	20587496/248	F067	P071	FC337		Korean	EA	NM_014874.3	c.1090C>T	p.Arg364Trp	/	/	AD	M	heter	/
**AP1844**	***MFN2***	20587496/248	F067	P072	FC337		Korean	EA	NM_014874.3	c.1090C>T	p.Arg364Trp	/	/	AD	M	heter	/
**AP1845**	***MFN2***	21149811	F068	P073	24		Caucasian	Non-EA	NM_014874.3	c.2140>G	p.Ile714Val	/	/	AD	M	compound I	Female
**AP1846**	***MFN2***	21149811	F069	P074	25		Caucasian	Non-EA	NM_014874.3	c.2213C>T	p.Ala738Val	/	/	AD	M	compound I	Female
**AP1847**	***MFN2***	21601224	F070	P075	INDEX 1	III-6	Chinese	EA	NM_014874.3	c.280C>T	p.Arg94Trp	/	/	AD	M	compound I	Male
**AP1848**	***MFN2***	21601224	F070	P076	INDEX 1	II-2	Chinese	EA	NM_014874.3	c.280C>T	p.Arg94Trp	/	/	AD	M	compound I	Male
**AP1849**	***MFN2***	21601224	F070	P077	INDEX 1	III-2	Chinese	EA	NM_014874.3	c.280C>T	p.Arg94Trp	/	/	AD	M	compound I	Female
**AP1850**	***MFN2***	21601224	F070	P078	INDEX 1	IV-3	Chinese	EA	NM_014874.3	c.280C>T	p.Arg94Trp	/	/	AD	M	compound I	Female
**AP1851**	***MFN2***	21601224	F070	P079	INDEX 1	IV-6	Chinese	EA	NM_014874.3	c.280C>T	p.Arg94Trp	/	/	AD	M	compound I	Female
**AP1852**	***MFN2***	21601224	F071	P080	INDEX 2		Chinese	EA	NM_014874.3	c.280C>T	p.Arg94Trp	/	/	AD	M	compound I	Male
**AP1853**	***MFN2***	21601224	F071	P081	INDEX 2		Chinese	EA	NM_014874.3	c.280C>T	p.Arg94Trp	/	/	AD	M	compound I	Male
**AP1854**	***MFN2***	21601224	F072	P082	INDEX 3		Chinese	EA	NM_014874.3	c.311G>A	p.Arg104Gln	/	/	AD	M	compound I	Male
**AP1855**	***MFN2***	21601224	F073	P083	INDEX 4		Chinese	EA	NM_014874.3	c.311G>A	p.Arg104Gln	/	/	AD	M	compound I	Male
**AP1856**	***MFN2***	21601224	F074	P084	INDEX 5		Chinese	EA	NM_014874.3	c.311G>A	p.Arg104Gln	/	/	AD	M	compound I	Female
**AP1857**	***MFN2***	21601224	F075	P085	INDEX 6		Chinese	EA	NM_014874.3	c.310 C>T	p.Arg104Trp	/	/	AD	M	compound I	Female
**AP1858**	***MFN2***	21601224	F076	P086	INDEX 7		Chinese	EA	NM_014874.3	c.314C>T	p.Thr105Met	/	/	AD	M	compound I	Male
**AP1859**	***MFN2***	21601224	F077	P087	INDEX 8		Chinese	EA	NM_014874.3	c.614T>C	p.Val205Ala	/	/	AD	M	compound I	Male
**AP1860**	***MFN2***	21601224	F078	P088	INDEX 9		Chinese	EA	NM_014874.3	c.718T>G	p.Phe240Val	/	/	AD	M	compound I	Male
**AP1861**	***MFN2***	21601224	F079	P089	INDEX 10		Chinese	EA	NM_014874.3	c.718T>G	p.Phe240Val	/	/	AD	M	compound I	Male
**AP1862**	***MFN2***	21601224	F079	P090	INDEX 10		Chinese	EA	NM_014874.3	c.1090C>T	p.Arg364Trp	/	/	AD	M	compound I	Female
**AP1863**	***MFN2***	21601224	F080	P091	INDEX 11		Chinese	EA	NM_014874.3	c.1090C>T	p.Arg364Trp	/	/	AD	M	compound I	Male
**AP1864**	***MFN2***	21601224	F080	P092	INDEX 11		Chinese	EA	NM_014874.3	c.1090C>T	p.Arg364Trp	/	/	AD	M	compound I	Female
**AP1865**	***MFN2***	21601224	F081	P093	INDEX 12		Chinese	EA	NM_014874.3	c.2218T>C	p.Trp740Arg	/	/	AD	M	compound I	Female
**AP1866**	***MFN2***	21601224	F081	P094	INDEX 12		Chinese	EA	NM_014874.3	c.2218T>C	p.Trp740Arg	/	/	AD	M	compound I	Male
**AP1867**	***MFN2***	21715711	F082	P095	Family 2	II-1	Caucasian	Non-EA	NM_014874.3	c.922G>T	p.Glu308*	c.1556G>C	p.Arg519Pro	AR	M+T	heter	/
**AP1868**	***MFN2***	21715711	F083	P096	Family 3	II-2	Caucasian	Non-EA	NM_014874.3	c.1085C>T	p.Thr362Met	c.113_115del	p.Lys38del	AR	M+M	heter	/
**AP1869**	***MFN2***	21715711	F083	P097	Family 3	II-1	Caucasian	Non-EA	NM_014874.3	c.1085C>T	p.Thr362Met	c.113_115del	p.Lys38del	AR	M+M	heter	/
**AP1870**	***MFN2***	21987543	F084	P098	Family 1	VI-3	Caucasian	Non-EA	NM_014874.3	c.436C>T	p.Leu146Phe	/	/	AD	M	heter	/
**AP1871**	***MFN2***	21987543	F084	P099	Family 1	VI-7	Caucasian	Non-EA	NM_014874.3	c.436C>T	p.Leu146Phe	/	/	AD	M	heter	/
**AP1872**	***MFN2***	21987543	F084	P100	Family 1	VI-8	Caucasian	Non-EA	NM_014874.3	c.436C>T	p.Leu146Phe	/	/	AD	M	heter	/
**AP1873**	***MFN2***	21987543	F084	P101	Family 1	VI-9	Caucasian	Non-EA	NM_014874.3	c.436C>T	p.L146P	/	/	AD	M	heter	/
**AP1874**	***MFN2***	21987543	F084	P102	Family 1	VI-10	Caucasian	Non-EA	NM_014874.3	c.436C>T	p.L146P	/	/	AD	M	heter	/
**AP1875**	***MFN2***	21987543	F084	P103	Family 1	V-3	Caucasian	Non-EA	NM_014874.3	c.436C>T	p.Leu146Phe	/	/	AD	M	heter	/
**AP1876**	***MFN2***	21987543	F084	P104	Family 1	V-7	Caucasian	Non-EA	NM_014874.3	c.436C>T	p.Leu146Phe	/	/	AD	M	heter	/
**AP1877**	***MFN2***	21987543	F084	P105	Family 1	V-17	Caucasian	Non-EA	NM_014874.3	c.436C>T	p.Leu146Phe	/	/	AD	M	heter	/
**AP1878**	***MFN2***	21987543	F084	P106	Family 1	V-18	Caucasian	Non-EA	NM_014874.3	c.436C>T	p.Leu146Phe	/	/	AD	M	heter	/
**AP1879**	***MFN2***	21987543	F084	P107	Family 1	IV-8	Caucasian	Non-EA	NM_014874.3	c.436C>T	p.Leu146Phe	/	/	AD	M	heter	/
**AP1880**	***MFN2***	22189565	F084	P108	Family 1	I-1	Tunisian	Non-EA	NM_014874.3	c.629A>T	p.Asp210Val	/	/	AD	M	heter	/
**AP1881**	***MFN2***	22189565	F084	P109	Family 1	II-4	Tunisian	Non-EA	NM_014874.3	c.629A>T	p.Asp210Val	/	/	AD	M	heter	/
**AP1882**	***MFN2***	22189565	F084	P110	Family 1	II-5	Tunisian	Non-EA	NM_014874.3	c.629A>T	p.Asp210Val	/	/	AD	M	heter	/
**AP1883**	***MFN2***	22189565	F084	P111	Family 1	II-7	Tunisian	Non-EA	NM_014874.3	c.629A>T	p.Asp210Val	/	/	AD	M	heter	/
**AP1884**	***MFN2***	22189565	F084	P112	Family 1	II-8	Tunisian	Non-EA	NM_014874.3	c.629A>T	p.Asp210Val	/	/	AD	M	heter	/
**AP1885**	***MFN2***	22189565	F084	P113	Family 1	II-9	Tunisian	Non-EA	NM_014874.3	c.629A>T	p.Asp210Val	/	/	AD	M	heter	/
**AP1886**	***MFN2***	22189565	F084	P114	Family 1	II-11	Tunisian	Non-EA	NM_014874.3	c.629A>T	p.Asp210Val	/	/	AD	M	heter	/
**AP1887**	***MFN2***	22189565	F084	P115	Family 1	II-12	Tunisian	Non-EA	NM_014874.3	c.629A>T	p.Asp210Val	/	/	AD	M	heter	/
**AP1888**	***MFN2***	22189565	F084	P116	Family 1	III-7	Tunisian	Non-EA	NM_014874.3	c.629A>T	p.Asp210Val	/	/	AD	M	heter	/
**AP1889**	***MFN2***	22189565	F084	P117	Family 1	III-14	Tunisian	Non-EA	NM_014874.3	c.629A>T	p.Asp210Val	/	/	AD	M	heter	/
**AP1890**	***MFN2***	22189565	F084	P118	Family 1	III-15	Tunisian	Non-EA	NM_014874.3	c.629A>T	p.Asp210Val	/	/	AD	M	heter	/
**AP1891**	***MFN2***	22189565	F084	P119	Family 1	III-16	Tunisian	Non-EA	NM_014874.3	c.629A>T	p.Asp210Val	/	/	AD	M	heter	/
**AP1892**	***MFN2***	22206013	F085	P120	/	10	Taiwanese	EA	NM_014874.3	c.475-1G>T	p.?	/	/	AD	M	compound I	/
**AP1893**	***MFN2***	22206013	F086	P121	/	11	Taiwanese	EA	NM_014874.3	c.697C>G	p.L233V	/	/	AD	M	compound I	/
**AP1894**	***MFN2***	22206013	F087	P122	/	12	Taiwanese	EA	NM_014874.3	c.1090C>T	p.Arg364Trp	/	/	AD	M	compound I	/
**AP1895**	***MFN2***	22206013	F088	P123	/	13	Taiwanese	EA	NM_014874.3	c.1090C>T	p.Arg364Trp	/	/	AD	M	compound I	/
**AP1896**	***MFN2***	22206013	F089	P124	/	14	Taiwanese	EA	NM_014874.3	c.2230_2231delinsAT	p.Glu744Met	/	/	AD	M	compound I	/
**AP1897**	***MFN2***	19889647	F090	P125	MFN 06-7		Spanish	Non-EA	NM_014874.3	c.280C>T	p.Arg94Trp	/	/	AD	M	compound I	/
**AP1898**	***MFN2***	19889647	F091	P126	MFN 06-15		Spanish	Non-EA	NM_014874.3	c.281G>A	p.Arg94Gln	/	/	AD	M	compound I	/
**AP1899**	***MFN2***	19889647	F092	P127	CMT II-2		Spanish	Non-EA	NM_014874.3	c.609T>G	p.Ile203Met	/	/	AD	M	compound I	/
**AP1900**	***MFN2***	19889647	F093	P128	CMT 560		Spanish	Non-EA	NM_014874.3	c.752C>T	p.Asn252Lys	/	/	AD	M	compound I	/
**AP1901**	***MFN2***	19889647	F094	P129	CMT II-4		Spanish	Non-EA	NM_014874.3	c.828G>C	p.Gln276His	/	/	AD	M	compound I	/
**AP1902**	***MFN2***	19889647	F095	P130	CMT1344		Spanish	Non-EA	NM_014874.3	c.892G>A	p.Gly298Arg	/	/	AD	M	compound I	/
**AP1903**	***MFN2***	19889647	F096	P131	CMT 364		Spanish	Non-EA	NM_014874.3	c.1127T>C	p.Met376Val	/	/	AD	M	compound I	/
**AP1904**	***MFN2***	19889647	F097	P132	MFN 06-6		Spanish	Non-EA	NM_014874.3	c.1091G>A	p.Arg364Gln	/	/	AD	M	compound I	/
**AP1905**	***MFN2***	19889647	F098	P133	MFN 06-16		Spanish	Non-EA	NM_014874.3	c.1403G>A	p.Arg468His	/	/	AD	M	compound I	/
**AP1906**	***MFN2***	19889647	F099	P134	CMT 1045		Spanish	Non-EA	NM_014874.3	c.1403G>A	p.Arg468His	/	/	AD	M	compound I	/
**AP1907**	***MFN2***	19889647	F100	P135	CMT 1088		Spanish	Non-EA	NM_014874.3	c.1403G>A	p.Arg468His	/	/	AD	M	compound I	/
**AP1908**	***MFN2***	19889647	F101	P136	CMT II-21		Spanish	Non-EA	NM_014874.3	c.1403G>A	p.Arg468His	/	/	AD	M	compound I	/
**AP1909**	***MFN2***	19889647	F102	P137	MFN 06-9		Spanish	Non-EA	NM_014874.3	c.1403G>A	p.Arg468His	/	/	AD	M	compound I	/
**AP1910**	***MFN2***	24078732	F103	P138	/		Caucasian	Non-EA	NM_014874.3	c.306dupT	p.G103WfsX41	/	/	AD	M	compound I	/
**AP1911**	***MFN2***	24078732	F104	P139	/		Caucasian	Non-EA	NM_014874.3	c.752C>T	p.Pro251Leu	/	/	AD	M	compound I	/
**AP1912**	***MFN2***	24311535/2486	F105	P140	FC81	9	Korean	EA	NM_014874.3	c.494A>G	p.His165Arg	/	/	AD	M	compound I	/
**AP1913**	***MFN2***	24311535/2486	F106	P141	FC280	16	Korean	EA	NM_014874.3	c.839 G>A	p.Arg280His	/	/	AD	M	compound I	/
**AP1914**	***MFN2***	24311535/2486	F107	P142	FC337	17	Korean	EA	NM_014874.3	c.1090C>T	p.Arg364Trp	/	/	AD	M	compound I	/
**AP1915**	***MFN2***	24311535/2486	F108	P143	FC337	18	Korean	EA	NM_014874.3	c.310C>T	p.Arg104Trp	/	/	AD	M	compound I	/
**AP1916**	***MFN2***	24311535	F109	P144	/	19	Korean	EA	NM_014874.3	c.312-1G>C	p.?	/	/	AD	M	compound I	/
**AP1917**	***MFN2***	24311535	F110	P145	/	20	Korean	EA	NM_014874.3	c.1252C>T	p.Arg418*	/	/	AD	M	compound I	/
**AP1918**	***MFN2***	24311535	F111	P146	/	21	Korean	EA	NM_014874.3			/	/	AD	M	compound I	/
**AP1919**	***MFN2***	24444136	F112	P147	1		Norwegian	Non-EA	NM_014874.3	c.250A>G	p.Lys84Glu	/	/	AD	M	compound I	/
**AP1920**	***MFN2***	24444136	F113	P148	2		Norwegian	Non-EA	NM_014874.3	c.1709A>G	p.Asn570Ser	/	/	AD	M	compound I	/
**AP1921**	***MFN2***	24444136	F114	P149	3		Norwegian	Non-EA	NM_014874.3	c.2146_2148dup	p.Ala716dup	/	/	AD	M	compound I	/
**AP1922**	***MFN2***	24444136	F115	P150	4		Norwegian	Non-EA	NM_014874.3	c.692C>T	p.Ser231Phe	/	/	AD	M	compound I	/
**AP1923**	***MFN2***	24530046	F116	P151	/	P1	Finnish	Non-EA	NM_014874.3	c.708G>A	p.=	/	/	AD	M	compound I	Male
**AP1924**	***MFN2***	24530046	F116	P152	/	P2	Finnish	Non-EA	NM_014874.3	c.708G>A	p.=	/	/	AD	M	compound I	Male
**AP1925**	***MFN2***	24627108	F117	P153	/	HMSN-R6	Caucasian	Non-EA	NM_014874.3	c.776G>A	p.Arg259His	/	/	AD	M	compound I	Female
**AP1926**	***MFN2***	24803844	F118	P154	/		Polish	Non-EA	NM_014874.3	c.311+1G>T	p.?	/	/	AD	M	compound I	Male
**AP1927**	***MFN2***	24819634	F119	P155	/	9837	Italian	Non-EA	NM_014874.3	c.984_986delAGA	p.Glu329del	/	/	AD	M	heter	/
**AP1928**	***MFN2***	24819634	F120	P156	/	9989	Italian	Non-EA	NM_014874.3	c.1090C>T	p.Arg364Trp	/	/	AD	M	heter	/
**AP1929**	***MFN2***	24863639	F121	P157	FC34		Korean	EA	NM_014874.3	c.275T>C	p.Leu92Pro	/	/	AD	M	heter	/
**AP1930**	***MFN2***	24863639	F122	P158	FC25		Korean	EA	NM_014874.3	c.280C>T	p.Arg94Trp	/	/	AD	M	heter	/
**AP1931**	***MFN2***	24863639	F123	P159	FC113		Korean	EA	NM_014874.3	c.280C>T	p.Arg94Trp	/	/	AD	M	heter	/
**AP1932**	***MFN2***	24863639	F124	P160	FC250		Korean	EA	NM_014874.3	c.280C>T	p.Arg94Trp	/	/	AD	M	heter	/
**AP1933**	***MFN2***	24863639	F125	P161	FC450		Korean	EA	NM_014874.3	c.280C>T	p.Arg94Trp	/	/	AD	M	heter	/
**AP1934**	***MFN2***	24863639	F126	P162	FC514		Korean	EA	NM_014874.3	c.280C>T	p.Arg94Trp	/	/	AD	M	heter	/
**AP1935**	***MFN2***	24863639	F127	P163	FC356		Korean	EA	NM_014874.3	c.310 C>T	p.Arg104Trp	/	/	AD	M	heter	/
**AP1936**	***MFN2***	24863639	F128	P164	FC600		Korean	EA	NM_014874.3	c.617C>T	p.Thr206Ile	/	/	AD	M	heter	/
**AP1937**	***MFN2***	24863639	F129	P165	FC431		Korean	EA	NM_014874.3	c.280C>T	p.Arg94Trp	/	/	AD	M	heter	/
**AP1938**	***MFN2***	24863639	F130	P166	FC228		Korean	EA	NM_014874.3	c.1367C> T	p.Pro456Leu	/	/	AD	M	heter	/
**AP1939**	***MFN2***	24863639	F131	P167	FC365		Korean	EA	NM_014874.3	c.1525G> C	p.Val509Leu	/	/	AD	M	heter	/
**AP1940**	***MFN2***	24863639	F132	P168	FC184		Korean	EA	NM_014874.3	c.1930_1932 delCTC	p.Leu644del	/	/	AD	M	heter	/
**AP1941**	***MFN2***	24863639	F133	P169	FC368		Korean	EA	NM_014874.3	c.2230G>A	p.Glu744Lys	/	/	AD	M	heter	/
**AP1942**	***MFN2***	24863639	F134	P170	FC477		Korean	EA	NM_014874.3	c.292A>C	p.Lys98Gln	/	/	AD	M	heter	/
**AP1943**	***MFN2***	24863639	F135	P171	FC135		Korean	EA	NM_014874.3	c.314C>T	p.Thr105Met	/	/	AD	M	heter	/
**AP1944**	***MFN2***	24863639	F136	P172	FC364		Korean	EA	NM_014874.3	c.314C>T	p.Thr105Met	/	/	AD	M	heter	/

**AP1945**	***MFN2***	24863639	F137	P173	FC600		Korean	EA	NM_014874.3	c.617C>T	p.Thr206Ile	/	/	AD	M	heter
**AP1946**	***MFN2***	24863639	F138	P174	FC169		Korean	EA	NM_014874.3	c.839G>A	p.Arg280His	/	/	AD	M	heter
**AP1947**	***MFN2***	24863639	F139	P175	FC527		Korean	EA	NM_014874.3	c.839G>A	p.Arg280His	/	/	AD	M	heter
**AP1948**	***MFN2***	24863639	F140	P176	FC257		Korean	EA	NM_014874.3	c. 1048T>C	p.Ser350Pro	/	/	AD	M	heter
**AP1949**	***MFN2***	24863639	F141	P177	FC259		Korean	EA	NM_014874.3	c.1253G>A	p.Arg418Gln	/	/	AD	M	heter
**AP1950**	***MFN2***	24863639	F142	P178	FC6		Korean	EA	NM_014874.3	c.1090C>T	p.Arg364Trp	/	/	AD	M	heter
**AP1951**	***MFN2***	24863639	F143	P179	FC55		Korean	EA	NM_014874.3	c.1090C>T	p.Arg364Trp	/	/	AD	M	heter
**AP1952**	***MFN2***	24863639	F144	P180	FC87		Korean	EA	NM_014874.3	c.1090C>T	p.Arg364Trp	/	/	AD	M	heter
**AP1953**	***MFN2***	24863639	F145	P181	FC256		Korean	EA	NM_014874.3	c.1090C>T	p.Arg364Trp	/	/	AD	M	heter
**AP1954**	***MFN2***	24863639	F146	P182	FC337		Korean	EA	NM_014874.3	c.1090C>T	p.Arg364Trp	/	/	AD	M	heter
**AP1955**	***MFN2***	26114802/2171	F147	P183	1	II-2	Caucasian	Non-EA	NM_014874.3	Del ex 7-8	p.?		p.Phe216Ser	AR	M+T	compound
**AP1956**	***MFN2***	26114802/2171	F147	P184	1	II-1	Caucasian	Non-EA	NM_014874.3	Del ex 7-8	p.?		p.Phe216Ser	AR	M+T	compound
**AP1957**	***MFN2***	26114802	F148	P185	2	III-2	Caucasian	Non-EA	NM_014874.3	Del ex 7-8	p.?		p.Thr362Met	AR	M+T	compound
**AP1958**	***MFN2***	26114802	F149	P186	3	II-1	Caucasian	Non-EA	NM_014874.3	Del ex 7-8	p.?		p.Thr362Met	AR	M+T	compound
**AP1959**	***MFN2***	26114802	F150	P187	4	II-2	Caucasian	Non-EA	NM_014874.3	Del ex 7-8	p.?	c.2119C>T	p.Arg707Trp	AR	M+T	compound
**AP1960**	***MFN2***	26306937	F151	P188	/		Caucasian	Non-EA	NM_014874.3	c.748C>T	p.Arg250Trp	c.1426C>T	p.Arg476*	AR	M+T	compound
**AP1961**	***MFN2***	26382835	F152	P189	/	IV6	Chinese	EA	NM_014874.3	c.280C>T	p.Arg94Trp	/	/	AD	M	heter
**AP1962**	***MFN2***	26382835	F152	P190	/	IV3	Chinese	EA	NM_014874.3	c.280C>T	p.Arg94Trp	/	/	AD	M	heter
**AP1963**	***MFN2***	26382835	F152	P191	/	III2	Chinese	EA	NM_014874.3	c.280C>T	p.Arg94Trp	/	/	AD	M	heter
**AP1964**	***MFN2***	26382835	F152	P192	/	II2	Chinese	EA	NM_014874.3	c.280C>T	p.Arg94Trp	/	/	AD	M	heter
**AP1965**	***MFN2***	26382835	F152	P193	/	Index 1(III6)	Chinese	EA	NM_014874.3	c.280C>T	p.Arg94Trp	/	/	AD	M	heter
**AP1966**	***MFN2***	26382835	F152	P194	/	III2	Chinese	EA	NM_014874.3	c.280C>T	p.Arg94Trp	/	/	AD	M	heter
**AP1967**	***MFN2***	26382835	F153	P195	/	Index 2 (II4)	Chinese	EA	NM_014874.3	c.280C>T	p.Arg94Trp	/	/	AD	M	heter
**AP1968**	***MFN2***	26382835	F153	P196	/	Index3	Chinese	EA	NM_014874.3	c.281G>A	p.Arg94Gln	/	/	AD	M	heter
**AP1969**	***MFN2***	26382835	F154	P197	/	Index4	Chinese	EA	NM_014874.3	c.281G>A	p.Arg94Gln	/	/	AD	M	heter
**AP1970**	***MFN2***	26382835	F155	P198	/	Index5	Chinese	EA	NM_014874.3	c.281G>A	p.Arg94Gln	/	/	AD	M	heter
**AP1971**	***MFN2***	26382835	F156	P199	/	Index6	Chinese	EA	NM_014874.3	c.310 C>T	p.Arg104Trp	/	/	AD	M	heter
**AP1972**	***MFN2***	26382835	F157	P200	/	Index7	Chinese	EA	NM_014874.3	c.310 C>T	p.Arg104Trp	/	/	AD	M	heter
**AP1973**	***MFN2***	26382835	F158	P201	/	Index8	Chinese	EA	NM_014874.3	c.314C>T	p.Thr105Met	/	/	AD	M	heter
**AP1974**	***MFN2***	26382835	F159	P202	/	Index9	Chinese	EA	NM_014874.3	c.614T>C	p.Val205Ala	/	/	AD	M	heter
**AP1975**	***MFN2***	26382835	F160	P203	/	Index10	Chinese	EA	NM_014874.3	c.718T>G	p.Phe240Val	/	/	AD	M	heter
**AP1976**	***MFN2***	26382835	F161	P204	/	Index11	Chinese	EA	NM_014874.3	c.1090C>T	p.Arg364Trp	/	/	AD	M	heter
**AP1977**	***MFN2***	26382835	F162	P205	/	Index12	Chinese	EA	NM_014874.3	c.2219G>C	p.Trp740Ser	/	/	AD	M	heter
**AP1978**	***MFN2***	26686600	F163	P206	/	A-II-3	Caucasian	Non-EA	NM_014874.3	c.311G > A	p.Arg104Gln	/	/	AD	M	heter
**AP1979**	***MFN2***	26686600	F164	P207	/	B-II-1	Caucasian	Non-EA	NM_014874.3	c.280C>T	p.Arg94Trp	/	/	AD	M	heter
**AP1980**	***MFN2***	26686600	F165	P208	/	C-III-1	Caucasian	Non-EA	NM_014874.3	c.748C > T	p.Arg250Trp	/	/	AD	M	heter
**AP1981**	***MFN2***	26686600	F166	P209	/	C-III-2	Caucasian	Non-EA	NM_014874.3	c.748C > T	p.Arg250Trp	/	/	AD	M	heter
**AP1982**	***MFN2***	26686600	F167	P210	/	D-II-1	Caucasian	Non-EA	NM_014874.3	c.1987 >T	p.Arg663Cys	/	/	AD	M	heter
**AP1983**	***MFN2***	26686600	F168	P211	/	E-II-1	Caucasian	Non-EA	NM_014874.3	c.280C>T	p.Arg94Trp	/	/	AD	M	heter
**AP1984**	***MFN2***	26801520	F169	P212	/	Index1 III-1	Chinese	EA	NM_014874.3	c.385A>G	p.Thr129Ala	/	/	AD	M	heter
**AP1985**	***MFN2***	26801520	F169	P213	/	Index1 II-2	Chinese	EA	NM_014874.3	c.385A>G	p.Thr129Ala	/	/	AD	M	heter
**AP1986**	***MFN2***	26801520	F169	P214	/	Index1 II-4	Chinese	EA	NM_014874.3	c.385A>G	p.Thr129Ala	/	/	AD	M	heter
**AP1987**	***MFN2***	26801520	F170	P215	/	Index2	Chinese	EA	NM_014874.3	c.746C>T	p.Ser249Phe	/	/	AD	M	heter
**AP1988**	***MFN2***	26801520	F171	P216	/	Index3	Chinese	EA	NM_014874.3	c.1100A>C	p.Gln367Pro	/	/	AD	M	heter
**AP1989**	***MFN2***	26801520	F172	P217	/	Index4 II-2	Chinese	EA	NM_014874.3	c.2021A>T	p.Gln674Leu	/	/	AD	M	heter
**AP1990**	***MFN2***	26801520	F172	P218	/	Index4 III-2	Chinese	EA	NM_014874.3	c.2021A>T	p.Gln674Leu	/	/	AD	M	heter
**AP1991**	***MFN2***	26801520	F172	P219	/	Index4 III-3	Chinese	EA	NM_014874.3	c.2021A>T	p.Gln674Leu	/	/	AD	M	heter
**AP1992**	***MFN2***	26801520	F173	P220	/	Index5	Chinese	EA	NM_014874.3	c.280C>T	p.Arg94Trp	/	/	AD	M	heter
**AP1993**	***MFN2***	26801520	F174	P221	/	Index6 III-1	Chinese	EA	NM_014874.3	c.280C>T	p.Arg94Trp	/	/	AD	M	heter
**AP1994**	***MFN2***	26801520	F174	P222	/	Index6 IV-1	Chinese	EA	NM_014874.3	c.280C>T	p.Arg94Trp	/	/	AD	M	heter
**AP1995**	***MFN2***	26801520	F174	P223	/	Index6 IV-2	Chinese	EA	NM_014874.3	c.280C>T	p.Arg94Trp	/	/	AD	M	heter
**AP1996**	***MFN2***	26801520	F175	P224	/	Index7	Chinese	EA	NM_014874.3	c.281G>A	p.Arg94Gln	/	/	AD	M	heter
**AP1997**	***MFN2***	26801520	F176	P225	/	Index8	Chinese	EA	NM_014874.3	c.314C>T	p.Thr105Met	/	/	AD	M	heter
**AP1998**	***MFN2***	26801520	F177	P226	/	Index9 II-1	Chinese	EA	NM_014874.3	c.395G>A	p.Cys132Tyr	/	/	AD	M	heter
**AP1999**	***MFN2***	26801520	F177	P227	/	Index9 I-2	Chinese	EA	NM_014874.3	c.395G>A	p.Cys132Tyr	/	/	AD	M	heter
**AP2000**	***MFN2***	26801520	F178	P228	/	Index10 III-1	Chinese	EA	NM_014874.3	c.1126A>G	p.Met376Val	/	/	AD	M	heter
**AP2001**	***MFN2***	26801520	F178	P229	/	Index10 II-1	Chinese	EA	NM_014874.3	c.1126A>G	p.Met376Val	/	/	AD	M	heter
**AP2002**	***MFN2***	26801520	F179	P230	/	Index11 II-1	Chinese	EA	NM_014874.3	c.2251C>T	p.Gln751*	/	/	AD	M	heter
**AP2003**	***MFN2***	26801520	F179	P231	/	Index11 I-2	Chinese	EA	NM_014874.3	c.2251C>T	p.Gln751*	/	/	AD	M	heter
**AP2004**	***MFN2***	26801520	F180	P232	/	Index12	Chinese	EA	NM_014874.3	c.358A>G	p.Lys120Glu	/	/	AD	M	heter
**AP2005**	***MFN2***	26801520	F181	P233	/	Inedx13	Chinese	EA	NM_014874.3	c.650G>T	p.Cys217Phe	/	/	AD	M	heter
**AP2006**	***MFN2***	26801520	F182	P234	/	Index14	Chinese	EA	NM_014874.3	c.919A>G	p.Lys307Glu	/	/	AD	M	heter
**AP2007**	***MFN2***	26801520	F183	P235	/	Index15	Chinese	EA	NM_014874.3	c.1067C>G	p.T356S	/	/	AD	M	heter
**AP2008**	***MFN2***	26955893	F184	P236	/		Caucasian	Non-EA	NM_014874.3	c.392A>G	p.Asn131Ser	c.2054_2069	p.Ser685Argf	AR	M+T	compound
**AP2009**	***MFN2***	26956144	F185	P237	/		Chinese	EA	NM_014874.3	c.730G>C	p.Val244Leu	/	/	AD	M	heter
**AP2010**	***MFN2***	26989944	F186	P238	/		Caucasian	Non-EA	NM_014874.3	c.776G>A	p.Arg259His	/	/	AD	M	heter
**AP2011**	***MFN2***	26989944	F187	P239	/		Caucasian	Non-EA	NM_014874.3	c.301 302delinsTT	p.Ala100Phe	/	/	AD	M	heter
**AP2012**	***MFN2***	26989944	F188	P240	/		Caucasian	Non-EA	NM_014874.3	c.839G>A	p.Arg280His	/	/	AD	M	heter
**AP2013**	***MFN2***	26989944	F189	P241	/		Caucasian	Non-EA	NM_014874.3	c.2240T>C	p.Met747Thr	/	/	AD	M	heter
**AP2014**	***MFN2***	26989944	F190	P242	/		Caucasian	Non-EA	NM_014874.3	c.2240T>C	p.Met747Thr	/	/	AD	M	heter
**AP2015**	***MFN2***	26989944	F191	P243	/		Caucasian	Non-EA	NM_014874.3	c.2240T>C	p.Met747Thr	/	/	AD	M	heter
**AP2016**	***MFN2***	26989944	F192	P244	/		Caucasian	Non-EA	NM_014874.3	c.2213C>T	p.Ala738Val	/	/	AD	M	heter
**AP2017**	***MFN2***	26989944	F193	P245	/		Caucasian	Non-EA	NM_014874.3	c.281G>A	p.Arg94Gln	/	/	AD	M	heter
**AP2018**	***MFN2***	27027447	F194	P246	/	case 2	Chinese	EA	NM_014874.3	c.379G>A	p.Gly127Ser	/	/	AD	M	heter
**AP2019**	***MFN2***	27027447	F195	P247	/	case 5	Chinese	EA	NM_014874.3	c.839G>A	p.Arg280His	/	/	AD	M	heter
**AP2020**	***MFN2***	27549087	F196	P248	/		Caucasian	Non-EA	NM_014874.3	c.280C>T	p.Arg94Trp	/	/	AD	M	heter
**AP2021**	***MFN2***	27549087	F197	P249	/		Caucasian	Non-EA	NM_014874.3	c.436 C>T	p.Leu146Phe	/	/	AD	M	heter
**AP2022**	***MFN2***	27549087	F198	P250	/		Caucasian	Non-EA	NM_014874.3	c.493C>T	p.His165Tyr	/	/	AD	M	heter
**AP2023**	***MFN2***	27549087	F199	P251	/		Caucasian	Non-EA	NM_014874.3	c.701T>A	p.Met234Lys	/	/	AD	M	heter
**AP2024**	***MFN2***	27549087	F200	P252	/		Caucasian	Non-EA	NM_014874.3	c.839G>A	p.Arg280His	/	/	AD	M	heter
**AP2025**	***MFN2***	27549087	F201	P253	/		Caucasian	Non-EA	NM_014874.3	c.880C>T	p.Arg294*	/	/	AD	M	heter
**AP2026**	***MFN2***	27549087	F202	P254	/		Caucasian	Non-EA	NM_014874.3	c.1081C>T	p.His361Tyr	/	/	AD	M	heter
**AP2027**	***MFN2***	27549087	F203	P255	/		Caucasian	Non-EA	NM_014874.3	c.1090C>T	p.Arg364Trp	/	/	AD	M	heter
**AP2028**	***MFN2***	27549087	F204	P256	/		Caucasian	Non-EA	NM_014874.3	c.1574A>G	p.Asn525Ser	/	/	AD	M	heter
**AP2029**	***MFN2***	27706887	F205	P257	/		Caucasian	Non-EA	NM_014874.3	c.239G>T	p.Gly80Val	c.311G>A	p.Arg104Gln	AR	M+M	compound
**AP2030**	***MFN2***	27862672	F206	P258	/	Pt26	Chinese	EA	NM_014874.3	c.497C>T	p.Ala166Val	/	/	AD	M	heter
**AP2031**	***MFN2***	27862672	F207	P259	/	Pt25	Chinese	EA	NM_014874.3	c.748C>T	p.Arg250Trp	/	/	AD	M	heter
**AP2032**	***MFN2***	27862672	F208	P260	/	Pt11	Chinese	EA	NM_014874.3	c.281G>A	p.Arg94Gln	/	/	AD	M	heter
**AP2033**	***MFN2***	28076385	F209	P261	/		Caucasian	Non-EA	NM_014874.3	c.820C>T	p.Arg274Trp	/	/	AD	M	heter
**AP2034**	***MFN2***	28215760	F210	P262	/	Patient 1	Italian	Non-EA	NM_014874.3	c.385A>C	p.Thr129Pro	c.2258dupT	p.Q754Afs*9	AR	M+T	compound
**AP2035**	***MFN2***	28215760	F211	P263	/	Patient 2	Italian	Non-EA	NM_014874.3	c.582C>G	p.Asp194Glu	c.974delG	p.G325Afs*9	AR	M+T	compound
**AP2036**	***MFN2***	28708278	F212	P264	/	3	Canadian	Non-EA	NM_014874.3	c.828_830dup	p.Gln276dup	/	/	AD	M	heter
**AP2037**	***MFN2***	28902413	F213	P265	/		Germany	Non-EA	NM_014874.3	c.1090C>T	p.Arg364Trp	/	/	AD	M	heter
**AP2038**	***MFN2***	28902413	F214	P266	/		Germany	Non-EA	NM_014874.3	c.1403G>A	p.Arg468His	/	/	AD	M	heter
**AP2039**	***MFN2***	28902413	F215	P267	/		Germany	Non-EA	NM_014874.3	c.281G>A	p.Arg94Gln	/	/	AD	M	heter
**AP2040**	***MFN2***	28902413	F216	P268	/		Germany	Non-EA	NM_014874.3	c.368C>T	p.Pro123Leu	/	/	AD	M	heter
**AP2041**	***MFN2***	28902413	F217	P269	/		Germany	Non-EA	NM_014874.3	c.313A>G	p.Thr105Ala	/	/	AD	M	heter
**AP2042**	***MFN2***	28902413	F218	P270	/		Germany	Non-EA	NM_014874.3	c.839G>A	p.Arg280His	/	/	AD	M	heter
**AP2043**	***MFN2***	28902413	F219	P271	/		Germany	Non-EA	NM_014874.3	c.1067C>A	p.Thr356Asn	/	/	AD	M	heter
**AP2044**	***MFN2***	29215088	F220	P272	/	patient 1	Japanese	EA	NM_014874.3	c.1894C>T	p.Arg632Trp	c.1894C>T	p.Arg632Trp	AR	M+M	homo
**AP2045**	***MFN2***	29215088	F220	P273	/	patient 2	Japanese	EA	NM_014874.3	c.1894C>T	p.Arg632Trp	c.1894C>T	p.Arg632Trp	AR	M+M	homo
**AP2046**	***MFN2***	29341354	F221	P274	/	I-3	Caucasian	Non-EA	NM_014874.3	c.2222T>G	p.Leu741Trp	/	/	AD	M	heter
**AP2047**	***MFN2***	29341354	F221	P275	/	I-9	Caucasian	Non-EA	NM_014874.3	c.2222T>G	p.Leu741Trp	/	/	AD	M	heter
**AP2048**	***MFN2***	29341354	F221	P276	/	II-3	Caucasian	Non-EA	NM_014874.3	c.2222T>G	p.Leu741Trp	/	/	AD	M	heter
**AP2049**	***MFN2***	29341354	F221	P277	/	II-4	Caucasian	Non-EA	NM_014874.3	c.2222T>G	p.Leu741Trp	/	/	AD	M	heter
**AP2050**	***MFN2***	29361379	F222	P278	/		Caucasian	Non-EA	NM_014874.3	c.526G>A	p.Gly176Ser	c.526G>A	p.Gly176Ser	AR	M+M	homo
**AP2051**	***MFN2***	29748043	F223	P279	/		Caucasian	Non-EA	NM_014874.3	c.526G>A	p.Gly176Ser	c.526G>A	p.Gly176Ser	AR	M+M	homo
**AP2052**	***MFN2***	29858556	F224	P280	/	28	Caucasian	Non-EA	NM_014874.3	c.404G>A	p.Arg135Gln	c.404G>A	p.Arg135Gln	AR	M+M	homo
**AP2053**	***MFN2***	29858556	F225	P281	/	29	Caucasian	Non-EA	NM_014874.3	c.1085C>T]	p.Thr362Met	c.1085C>T	p.Thr362Met	AR	M+M	compound
**AP2054**	***MFN2***	29858556	F226	P282	/	30	Caucasian	Non-EA	NM_014874.3	c.154G>A	p.Glu52Lys	c.154G>A	p.Glu52Lys	AR	M+M	homo
**AP2055**	***MFN2***	29858556	F227	P283	/	35	Caucasian	Non-EA	NM_014874.3	c.310C>T	p.Arg104Trp	/	/	AD	M	heter
**AP2056**	***MFN2***	30369951	F228	P284	/		Caucasian	Non-EA	NM_014874.3	c.1058C>T	p.Ala353Val	/	/	AD	M	heter
**AP2057**	***MFN2***	30373780	F229	P285	/	42	Caucasian	Non-EA	NM_014874.3	c.311G>T	p.Arg104Leu	/	/	AD	M	heter
**AP2058**	***MFN2***	30373780	F230	P286	/	26	Caucasian	Non-EA	NM_014874.3	c.1127T>G	p.Met376Arg	/	/	AD	M	heter
**AP2059**	***MFN2***	30373780	F231	P287	/	46	Caucasian	Non-EA	NM_014874.3	c.1714C>T	p.Gln572Glu	c.1928T>C	p.Leu643Pro	AR	M+M	compound
**AP2060**	***MFN2***	30564185	F232	P288	/	Pt68	Italian	Non-EA	NM_014874.3	c.2183A>G	p.Gln728Arg	/	/	AD	M	heter
**AP2061**	***MFN2***	30642740	F233	P289	/	III-2	Caucasian	Non-EA	NM_014874.3	c.283A>G	p.Arg95Gly	/	/	AD	M	heter
**AP2062**	***MFN2***	30642740	F233	P290	/	IV-1	Caucasian	Non-EA	NM_014874.3	c.283A>G	p.Arg95Gly	/	/	AD	M	heter
**AP2063**	***MFN2***	30642740	F233	P291	/	III-6	Caucasian	Non-EA	NM_014874.3	c.283A>G	p.Arg95Gly	/	/	AD	M	heter
**AP2064**	***MFN2***	30642740	F233	P292	/	II-1	Caucasian	Non-EA	NM_014874.3	c.283A>G	p.Arg95Gly	/	/	AD	M	heter
**AP2065**	***MFN2***	30642740	F233	P293	/	II-4	Caucasian	Non-EA	NM_014874.3	c.283A>G	p.Arg95Gly	/	/	AD	M	heter
**AP2066**	***MFN2***	30724636	F234	P294	/	14	Caucasian	Non-EA	NM_014874.3	c.406G>T	p.Val136Leu	c.406G>T	p.Val136Leu	AR	M+M	homo
**AP2067**	***MFN2***	31127728	F235	P295	/	Patient 1	Caucasian	Non-EA	NM_014874.3	c.2222T>G	p.Leu741Trp	/	/	AD	M	heter
**AP2068**	***MFN2***	31127728	F235	P296	/	Patient 2	Caucasian	Non-EA	NM_014874.3	c.2222T>G	p.Leu741Trp	/	/	AD	M	heter
**AP2069**	***MFN2***	31127728	F235	P297	/	Patient 3	Caucasian	Non-EA	NM_014874.3	c.2222T>G	p.Leu741Trp	/	/	AD	M	heter
**AP2070**	***MFN2***	31372974	F236	P298	/		Chinese	EA	NM_014874.3	c.284G>C	p.Arg95Thr	/	/	AD	M	heter
**AP2071**	***MFN2***	31407473	F237	P299	/		Caucasian	Non-EA	NM_014874.3	c.1252C>T	p.Arg418*	/	/	AD	M	heter
**AP2072**	***MFN2***	31453851	F238	P300	/		Saudi Arabian	Non-EA	NM_014874.3	c.58C>T	p. His20Tyr	c.2218T>A	p. Trp740Arg	AR	M+M	compound
**AP2073**	***MFN2***	31827005	F239	P301	/	5	Caucasian	Non-EA	NM_014874.3	c.436C>T	p.Leu146Phe	/	/	AD	M	heter
**AP2074**	***MFN2***	31827005	F240	P302	/	12	Caucasian	Non-EA	NM_014874.3	c. 449G>T	p.Gly150Val	del exon 7-8	p.?	AR	M+T	compound
**AP2075**	***MFN2***	31827005	F241	P303	/	18	Caucasian	Non-EA	NM_014874.3	c.2219G>C	p.Trp740Ser	/	/	AD	M	heter
**AP2076**	***MFN2***	31827005	F242	P304	/	42	Caucasian	Non-EA	NM_014874.3	c.1091 G>A	p.Arg364Gln	/	/	AD	M	heter
**AP2077**	***MFN2***	31827005	F243	P305	/	59	Caucasian	Non-EA	NM_014874.3	c.311G>T	p.Arg104Leu	/	/	AD	M	heter
**AP2078**	***MFN2***	31827005	F244	P306	/	60	Caucasian	Non-EA	NM_014874.3	c.839G>A	p.Arg280His	/	/	AD	M	heter
**AP2079**	***MFN2***	31827005	F245	P307	/	63	Caucasian	Non-EA	NM_014874.3	c.2219G>C	p.Trp740Ser	/	/	AD	M	heter
**AP2080**	***MFN2***	31827005	F246	P308	/	181	Caucasian	Non-EA	NM_014874.3	c.749G>A	p.Arg250Gln	c.1085C>G	p.Thr362Arg	AR	M+M	compound
**AP2081**	***MFN2***	31832804	F247	P309	/	P1	Caucasian	Non-EA	NM_014874.3	c.134A>G	p.Gln45Arg	/	/	AD	M	heter
**AP2082**	***MFN2***	31832804	F248	P310	/	P2	Caucasian	Non-EA	NM_014874.3	c.281G>A	p.Arg94Gln	/	/	AD	M	heter
**AP2083**	***MFN2***	31832804	F249	P311	/	P3	Caucasian	Non-EA	NM_014874.3	c.280C>T	p.Arg94Trp	/	/	AD	M	heter
**AP2084**	***MFN2***	31832804	F250	P312	/	P4	Caucasian	Non-EA	NM_014874.3	c.280C>T	p.Arg94Trp	/	/	AD	M	heter
**AP2085**	***MFN2***	31832804	F251	P313	/	P5	Caucasian	Non-EA	NM_014874.3	c.280C>T	p.Arg94Trp	/	/	AD	M	heter
**AP2086**	***MFN2***	31832804	F252	P314	/	P6	Caucasian	Non-EA	NM_014874.3	c.280C>T	p.Arg94Trp	/	/	AD	M	heter
**AP2087**	***MFN2***	31832804	F253	P315	/	P7	Caucasian	Non-EA	NM_014874.3	c.614T>C	p.Val205Ala	/	/	AD	M	heter
**AP2088**	***MFN2***	31832804	F254	P316	/	P8	Caucasian	Non-EA	NM_014874.3	c.776G>A	p.Arg259His	/	/	AD	M	heter
**AP2089**	***MFN2***	31832804	F255	P317	/	P9	Caucasian	Non-EA	NM_014874.3	c.827A>G	p.Gln276Arg	/	/	AD	M	heter
**AP2090**	***MFN2***	31832804	F256	P318	/	P10	Caucasian	Non-EA	NM_014874.3	c.827A>G	p.Gln276Arg	/	/	AD	M	heter
**AP2091**	***MFN2***	31832804	F257	P319	/	P11	Caucasian	Non-EA	NM_014874.3	c.839G>A	p.Arg280His	/	/	AD	M	heter
**AP2092**	***MFN2***	31832804	F258	P320	/	P12	Caucasian	Non-EA	NM_014874.3	c.839G>A	p.Arg280His	/	/	AD	M	heter
**AP2093**	***MFN2***	31832804	F259	P321	/	P13	Caucasian	Non-EA	NM_014874.3	c.1090C>T	p.Arg364Trp	/	/	AD	M	heter
**AP2094**	***MFN2***	31832804	F260	P322	/	P14	Caucasian	Non-EA	NM_014874.3	c.1090C>T	p.Arg364Trp	/	/	AD	M	heter
**AP2095**	***MFN2***	31832804	F261	P323	/	P15	Caucasian	Non-EA	NM_014874.3	c.776G>A	p.Arg259His	/	/	AD	M	heter
**AP2096**	***MFN2***	32214227	F262	P324	TR44A		Caucasian	Non-EA	NM_014874.3	c.1963A>G	p. Lys655Glu	c.1963A>G	p. Lys655Glu	AR	M+M	homo
**AP2097**	***MFN2***	32522799	F263	P325	/		India	Non-EA	NM_014874.3	c.775C>T	p.Arg259Cys	/	/	AD	M	heter
**AP2098**	***MFN2***	33067402	F264	P326	/		Caucasian	Non-EA	NM_014874.3	c.775C>T	p.Arg259Cys	/	/	AD	M	heter
**AP2099**	***MFN2***	33067402	F265	P327	/		Caucasian	Non-EA	NM_014874.3	c.280C>G	p.Arg94Gly	/	/	AD	M	heter
**AP2100**	***MFN2***	26686600/2218	F266	P328	/	F-III-2	Tunisian	Non-EA	NM_014874.3	c.629A > T	p.Asp210Val	/	/	AD	M	heter
**AP2101**	***MFN2***	26686600/2218	F267	P329	/	F-III-3	Tunisian	Non-EA	NM_014874.3	c.629A > T	p.Asp210Val	/	/	AD	M	heter
**AP2102**	***MFN2***	26686600/2218	F268	P330	/	F-III-4	Tunisian	Non-EA	NM_014874.3	c.629A > T	p.Asp210Val	/	/	AD	M	heter
**AP2103**	***MFN2***	26686600/2218	F269	P331	/	F-III-5	Tunisian	Non-EA	NM_014874.3	c.629A > T	p.Asp210Val	/	/	AD	M	heter
**AP2104**	***MFN2***	26686600/2218	F270	P332	/	F-III-6	Tunisian	Non-EA	NM_014874.3	c.629A > T	p.Asp210Val	/	/	AD	M	heter
**AP2105**	***MFN2***	29906321/2866	F271	P333	Family 1		Japanese	EA	NM_014874.3	c.154G>A	p.Glu52Lys	/	/	AD	M	heter
**AP2106**	***MFN2***	29906321/2866	F272	P334	Family 6		Japanese	EA	NM_014874.3	c.313A>T	p.Thr105Ser	/	/	AD	M	heter
**AP2107**	***MFN2***	29906321/2866	F273	P335	/		Japanese	EA	NM_014874.3	c.313A>T	p.Thr105Ser	/	/	AD	M	heter
**AP2108**	***MFN2***	29906321/2866	F274	P336	/		Japanese	EA	NM_014874.3	c.326A>G	p.Lys109Arg	/	/	AD	M	heter
**AP2109**	***MFN2***	29906321/2866	F275	P337	/		Japanese	EA	NM_014874.3	c.382C>T	p.His128Tyr	/	/	AD	M	heter
**AP2110**	***MFN2***	29906321/2866	F276	P338	/		Japanese	EA	NM_014874.3	c.382C>T	p.His128Tyr	/	/	AD	M	heter
**AP2111**	***MFN2***	29906321/2866	F277	P339	/		Japanese	EA	NM_014874.3	c.389C>T	p.Thr130Ile	/	/	AD	M	heter
**AP2112**	***MFN2***	29906321/2866	F278	P340	/		Japanese	EA	NM_014874.3	c.658G>A	p.Ala220Thr	/	/	AD	M	heter
**AP2113**	***MFN2***	29906321/2866	F279	P341	/		Japanese	EA	NM_014874.3	c.733A>C	p.Ser245Ar	/	/	AD	M	heter
**AP2114**	***MFN2***	29906321/2866	F280	P342	/		Japanese	EA	NM_014874.3	c.733A>C	p.Ser245Ar	/	/	AD	M	heter
**AP2115**	***MFN2***	29906321/2866	F281	P343	/		Japanese	EA	NM_014874.3	c.1078C>G	p.Gln360Glu	/	/	AD	M	heter
**AP2116**	***MFN2***	29906321/2866	F282	P344	/		Japanese	EA	NM_014874.3	c.1147_1149delGCT	p.Ala383del	/	/	AD	M	heter
**AP2117**	***MFN2***	29906321/2866	F283	P345	Family 3		Japanese	EA	NM_014874.3	c.1427G>C	p.Arg476Pro	/	/	AD	M	heter
**AP2118**	***MFN2***	29906321/2866	F284	P346	/		Japanese	EA	NM_014874.3	c.1636T>G	p.Ser546Ala	/	/	AD	M	heter
**AP2119**	***MFN2***	29906321/2866	F285	P347	/		Japanese	EA	NM_014874.3	c.2075T>G	p.Leu692Arg	/	/	AD	M	heter
**AP2120**	***MFN2***	29906321/2866	F286	P348	Family 4		Japanese	EA	NM_014874.3	c.2222T>C	p.Leu741Ser	/	/	AD	M	heter
**AP2121**	***MFN2***	29906321/2866	F287	P349	Family 5		Japanese	EA	NM_014874.3	c.2240T>G	p.Met747Arg	/	/	AD	M	heter
**AP2122**	***MFN2***	29906321/2866	F288	P350	/		Japanese	EA	NM_014874.3	c.2240T>G	p.Met747Arg	/	/	AD	M	heter
**AP2123**	***MFN2***	29906321/2866	F289	P351	/		Japanese	EA	NM_014874.3	c.2240T>G	p.Met747Arg	/	/	AD	M	heter
**AP2124**	***MFN2***	29906321/2866	F290	P352	/		Japanese	EA	NM_014874.3	c.2243_2244insT	p.Thr749Hisfs*14	/	/	AD	M	heter
**AP2125**	***MFN2***	29906321/2866	F291	P353	/		Japanese	EA	NM_014874.3	c.281G>A	p.Arg94Gln	/	/	AD	M	heter
**AP2126**	***MFN2***	29906321/2866	F292	P354	/		Japanese	EA	NM_014874.3	c.281G>A	p.Arg94Gln	/	/	AD	M	heter
**AP2127**	***MFN2***	29906321/2866	F293	P355	/		Japanese	EA	NM_014874.3	c.281G>A	p.Arg94Gln	/	/	AD	M	heter
**AP2128**	***MFN2***	29906321/2866	F294	P356	/		Japanese	EA	NM_014874.3	c.281G>A	p.Arg94Gln	/	/	AD	M	heter
**AP2129**	***MFN2***	29906321/2866	F295	P357	/		Japanese	EA	NM_014874.3	c.281G>A	p.Arg94Gln	/	/	AD	M	heter
**AP2130**	***MFN2***	29906321/2866	F296	P358	/		Japanese	EA	NM_014874.3	c.280C>T	p.Arg94Trp	/	/	AD	M	heter
**AP2131**	***MFN2***	29906321/2866	F297	P359	/		Japanese	EA	NM_014874.3	c.280C>T	p.Arg94Trp	/	/	AD	M	heter
**AP2132**	***MFN2***	29906321/2866	F298	P360	/		Japanese	EA	NM_014874.3	c.280C>T	p.Arg94Trp	/	/	AD	M	heter
**AP2133**	***MFN2***	29906321/2866	F299	P361	/		Japanese	EA	NM_014874.3	c.280C>T	p.Arg94Trp	/	/	AD	M	heter
**AP2134**	***MFN2***	29906321/2866	F300	P362	/		Japanese	EA	NM_014874.3	c.280C>T	p.Arg94Trp	/	/	AD	M	heter
**AP2135**	***MFN2***	29906321/2866	F301	P363	/		Japanese	EA	NM_014874.3	c.280C>T	p.Arg94Trp	/	/	AD	M	heter
**AP2136**	***MFN2***	29906321/2866	F302	P364	/		Japanese	EA	NM_014874.3	c.280C>G	p.Arg94Gly	/	/	AD	M	heter
**AP2137**	***MFN2***	29906321/2866	F303	P365	/		Japanese	EA	NM_014874.3	c.310 C>T	p.Arg104Trp	/	/	AD	M	heter
**AP2138**	***MFN2***	29906321/2866	F304	P366	/		Japanese	EA	NM_014874.3	c.310 C>T	p.Arg104Trp	/	/	AD	M	heter
**AP2139**	***MFN2***	29906321/2866	F305	P367	/		Japanese	EA	NM_014874.3	c.310 C>T	p.Arg104Trp	/	/	AD	M	heter
**AP2140**	***MFN2***	29906321/2866	F306	P368	/		Japanese	EA	NM_014874.3	c.311G>T	p.Arg104Leu	/	/	AD	M	heter
**AP2141**	***MFN2***	29906321/2866	F307	P369	/		Japanese	EA	NM_014874.3	c.314C>T	p.Thr105Met	PMP22	/	AD	digenic	heter
**AP2142**	***MFN2***	29906321/2866	F308	P370	/		Japanese	EA	NM_014874.3	c.314C>T	p.Thr105Met	/	/	AD	M	heter
**AP2143**	***MFN2***	29906321/2866	F309	P371	/		Japanese	EA	NM_014874.3	c.383A>G	p.His128Arg	/	/	AD	M	heter
**AP2144**	***MFN2***	29906321/2866	F310	P372	/		Japanese	EA	NM_014874.3	c.494A>G	p.His165Arg	/	/	AD	M	heter
**AP2145**	***MFN2***	29906321/2866	F311	P373	/		Japanese	EA	NM_014874.3	c.694A>G	p.Thr232Ala	/	/	AD	M	heter
**AP2146**	***MFN2***	29906321/2866	F312	P374	/		Japanese	EA	NM_014874.3	c.746C>T	p.Ser249Phe	/	/	AD	M	heter
**AP2147**	***MFN2***	29906321/2866	F313	P375	/		Japanese	EA	NM_014874.3	c.775C>T	p.Arg259Cys	/	/	AD	M	heter
**AP2148**	***MFN2***	29906321/2866	F314	P376	/		Japanese	EA	NM_014874.3	c.775C>T	p.Arg259Cys	/	/	AD	M	heter
**AP2149**	***MFN2***	29906321/2866	F315	P377	/		Japanese	EA	NM_014874.3	c.775C>T	p.Arg259Cys	/	/	AD	M	heter
**AP2150**	***MFN2***	29906321/2866	F316	P378	/		Japanese	EA	NM_014874.3	c.775C>T	p.Arg259Cys	/	/	AD	M	heter
**AP2151**	***MFN2***	29906321/2866	F317	P379	/		Japanese	EA	NM_014874.3	c.775C>T	p.Arg259Cys	/	/	AD	M	heter
**AP2152**	***MFN2***	29906321/2866	F318	P380	/		Japanese	EA	NM_014874.3	c.776G>A	p.Arg259His	/	/	AD	M	heter
**AP2153**	***MFN2***	29906321/2866	F319	P381	/		Japanese	EA	NM_014874.3	c.820C>T	p.Arg274Trp	/	/	AD	M	heter
**AP2154**	***MFN2***	29906321/2866	F320	P382	/		Japanese	EA	NM_014874.3	c.839G>A	p.Arg280His	/	/	AD	M	heter
**AP2155**	***MFN2***	29906321/2866	F321	P383	/		Japanese	EA	NM_014874.3	c.839G>A	p.Arg280His	/	/	AD	M	heter
**AP2156**	***MFN2***	29906321/2866	F322	P384	/		Japanese	EA	NM_014874.3	c.839G>A	p.Arg280His	/	/	AD	M	heter
**AP2157**	***MFN2***	29906321/2866	F323	P385	/		Japanese	EA	NM_014874.3	c.839G>A	p.Arg280His	c. 748C>T	p.Arg250Trp	AR	M+M	compound
**AP2158**	***MFN2***	29906321/2866	F324	P386	/		Japanese	EA	NM_014874.3	c.851T>A	p.Phe284Tyr	/	/	AD	M	heter
**AP2159**	***MFN2***	29906321/2866	F325	P387	/		Japanese	EA	NM_014874.3	c.919A>G	p.Lys307Glu	/	/	AD	M	heter
**AP2160**	***MFN2***	29906321/2866	F326	P388	/		Japanese	EA	NM_014874.3	c.707C>T	p.Thr236Met	/	/	AD	M	heter
**AP2161**	***MFN2***	29906321/2866	F327	P389	/		Japanese	EA	NM_014874.3	c.1085C>G	p.Thr362Arg	/	/	AD	M	heter
**AP2162**	***MFN2***	29906321/2866	F328	P390	/		Japanese	EA	NM_014874.3	c.1090C>T	p.Arg364Trp	/	/	AD	M	heter
**AP2163**	***MFN2***	29906321/2866	F329	P391	/		Japanese	EA	NM_014874.3	c.1090C>T	p.Arg364Trp	/	/	AD	M	heter
**AP2164**	***MFN2***	29906321/2866	F330	P392	/		Japanese	EA	NM_014874.3	c.1090C>T	p.Arg364Trp	/	/	AD	M	heter
**AP2165**	***MFN2***	29906321/2866	F331	P393	/		Japanese	EA	NM_014874.3	c.1090C>T	p.Arg364Trp	/	/	AD	M	heter
**AP2166**	***MFN2***	29906321/2866	F332	P394	/		Japanese	EA	NM_014874.3	c.1090C>T	p.Arg364Trp	/	/	AD	M	heter
**AP2167**	***MFN2***	29906321/2866	F333	P395	/		Japanese	EA	NM_014874.3	c.1090C>T	p.Arg364Trp	/	/	AD	M	heter
**AP2168**	***MFN2***	29906321/2866	F334	P396	/		Japanese	EA	NM_014874.3	c.1090C>T	p.Arg364Trp	/	/	AD	M	heter
**AP2169**	***MFN2***	29906321/2866	F335	P397	/		Japanese	EA	NM_014874.3	c.1090C>T	p.Arg364Trp	/	/	AD	M	heter
**AP2170**	***MFN2***	29906321/2866	F336	P398	/		Japanese	EA	NM_014874.3	c.1090C>T	p.Arg364Trp	/	/	AD	M	heter
**AP2171**	***MFN2***	29906321/2866	F337	P399	/		Japanese	EA	NM_014874.3	c.1128G>A	p.Met376Ile	/	/	AD	M	heter
**AP2172**	***MFN2***	29906321/2866	F338	P400	/		Japanese	EA	NM_014874.3	c.1128G>A	p.Met376Ile	/	/	AD	M	heter
**AP2173**	***MFN2***	29906321/2866	F339	P401	/		Japanese	EA	NM_014874.3	c.1128G>A	p.Met376Ile	/	/	AD	M	heter
**AP2174**	***MFN2***	29906321/2866	F340	P402	/		Japanese	EA	NM_014874.3	c.1126A>G	p.Met376Val	/	/	AD	M	heter
**AP2175**	***MFN2***	29906321/2866	F341	P403	/		Japanese	EA	NM_014874.3	c.1126A>G	p.Met376Val	/	/	AD	M	heter
**AP2176**	***MFN2***	29906321/2866	F342	P404	/		Japanese	EA	NM_014874.3	c.1132T>C	p.Ser378Pro	/	/	AD	M	heter
**AP2177**	***MFN2***	29906321/2866	F343	P405	/		Japanese	EA	NM_014874.3	c.1252C>T	p.Arg418*	/	/	AD	M	heter
**AP2178**	***MFN2***	29906321/2866	F344	P406	/		Japanese	EA	NM_014874.3	c.1930_1932delCTC	p.Leu644del	/	/	AD	M	heter
**AP2179**	***MFN2***	29906321/2866	F345	P407	/		Japanese	EA	NM_014874.3	c.2116A>C	p.Thr706Pro	/	/	AD	M	heter
**AP2180**	***MFN2***	29906321/2866	F346	P408	/		Japanese	EA	NM_014874.3	c.2129T>C	p.Leu710Pro	/	/	AD	M	heter
**AP2181**	***MFN2***	29906321/2866	F347	P409	/		Japanese	EA	NM_014874.3	c.2129T>C	p.Leu710Pro	/	/	AD	M	heter
**AP2182**	***MFN2***	29906321/2866	F348	P410	/		Japanese	EA	NM_014874.3	c.2220G>C	p.Trp740Cys	/	/	AD	M	heter
**AP2183**	***MFN2***	29906321/2866	F349	P411	/		Japanese	EA	NM_014874.3	c.475-1G>C	p.?	/	/	AD	M	heter
**AP2184**	***MFN2***	26382835	F350	P412	/	Index10 (II2)	Chinese	EA	NM_014874.3	c.718T>G	p.Phe240Val			AD	M	heter
**AP2185**	***MFN2***	26382835	F351	P413	/	Index11 (II5)	Chinese	EA	NM_014874.3	c.1090C>T	p.Arg364Trp			AD	M	heter
**AP2186**	***MFN2***	26382835	F352	P414	/	Index12 (II2)	Chinese	EA	NM_014874.3	c.2219G>C	p.Trp740Ser			AD	M	heter
**AP2187**	***MFN2***	24863639	F353	P415	*FC135*		Korean	EA	NM_014874.3	c.314C>T	p.Thr105Met			AD	M	heter
**AP2188**	***MFN2***	24819634	F354	P416	/	7333	Italian	Non-EA	NM_014874.3	c.1085C>T	p.Thr362Met			AD	M	heter
**AP2189**	***MFN2***	24819634	F355	P417	/	9040	Italian	Non-EA	NM_014874.3	c.281G>C	p.Arg94Prp	c.113_115del/	p.Lys38del	AR	?	compound
**AP2190**	***MFN2***	24819634	F356	P418	/	9629	Italian	Non-EA	NM_014874.3	c.494A>G	p.His165Arg			AD	M	heter
**AP2191**	***MFN2***	24819634	F357	P419	/	10095	Italian	Non-EA	NM_014874.3	c.864G>C	p.Gln288Asp	/	/	AD	M	heter
**AP2192**	***MFN2***	24819634	F358	P420	/	5364	Italian	Non-EA	NM_014874.3	c.1148>T	p.Ala383Val	/	/	AD	M	heter
**AP2193**	***MFN2***	24819634	F359	P421	/	9132	Italian	Non-EA	NM_014874.3	c.1148>T	p.Ala383Val	/	/	AD	M	heter
**AP2194**	***MFN2***	24819634	F360	P422	/	7284	Italian	Non-EA	NM_014874.3	c.1392+2T>C	p.?	/	/	AD	M	heter
**AP2195**	***MFN2***	24819634	F361	P423	/	10350	Italian	Non-EA	NM_014874.3	c.2116A>C	p.Thr706Pro	/	/	AD	M	heter
**AP2196**	***MFN2***	22762946	F363	P424	/	P1	Chinese	EA	NM_014874.3	c.280C>T	p.Arg94Trp	/	/	AD	M	heter
**AP2197**	***MFN2***	22762946	F364	P424	/	P2	Chinese	EA	NM_014874.3	c.1090C>T	p.Arg364Trp	/	/	AD	M	heter
**AP2198**	***MFN2***	22762946	F365	P424	/	P3	Chinese	EA	NM_014874.3	c.2220G>C	p.Trp740Cys	/	/	AD	M	heter
**AP2199**	***MFN2***	24819634	F362	P424	/	7440	Italian	Non-EA	NM_014874.3	c.2213C>T	p.Ala738Val	/	/	AD	M	heter
**AP2200**	***MIEF1***	33632269	F001	P001	/	1	Maghrebi	Non-EA	NM_00130456	c.718C>T	p.Tyr240Asn	/	/	AD	M	heter
**AP2201**	***MIEF1***	33632269	F002	P002	/	2	Egyptian	Non-EA	NM_00130456	c.436T>A	p.Arg146Trp	/	/	AD	M	heter
**AP2202**	***MIEF1***	29177109	F003	P003	1201		European	Non-EA	NM_00130456	c.505C>T	p.Arg169Trp	/	/	AD	M	heter
**AP2203**	***MIEF1***	33077954	F004	P004	KCHYD187-2		European	Non-EA	NM_00130456	c.184C>T	p.Arg62Cys	/	/	AD	M	heter
**AP2204**	***MTFMT***	32577402	F001	P001	/	/	Hutterite	Non-EA	NM_139242.4	c.994C>T	p.Arg332*	/	/	AD	Truncation	heter
**AP2205**	***MTFMT***	32133637	F002	P002	/	/	Caucasian	Non-EA	NM_139242.4	c.994C>T	p.Arg332*	c.434T>G	p.Val145Gly	AR	M+T	compound
**AP2206**	***MTFMT***	31501239	F003	P003	/	/	Caucasian	Non-EA	NM_139242.4	c.479T>C	p.Ile160Thr	c.1094G>A	p.Cys365Tyr	AR	M+M	compound
**AP2207**	***MTFMT***	30911575	F004	P004	/	1	British	Non-EA	NM_139242.4	c.626C>T	p.Ser209Leu	c.626C>T	p.Ser209Leu	AR	M+M	homo
**AP2208**	***MTFMT***	30911575	F005	P005	/	2	British	Non-EA	NM_139242.4	c.626C>T	p.Ser209Leu	c.626C>T	p.Ser209Leu	AR	M+M	homo
**AP2209**	***MTFMT***	30911575	F006	P006	/	3	British	Non-EA	NM_139242.4	c.626C>T	p.Ser209Leu	c.1100_1101d	p.Phe367Serfs	AR	M+T	compound
**AP2210**	***MTFMT***	30911575	F007	P007	/	4	British	Non-EA	NM_139242.4	c.626C>T	p.Ser209Leu	c.626C>T	p.Ser209Leu	AR	M+M	homo
**AP2211**	***MTFMT***	30911575	F008	P008	/	5	Australian	Non-EA	NM_139242.4	c.626C>T	p.Ser209Leu	c.626C>T	p.Ser209Leu	AR	M+M	homo
**AP2212**	***MTFMT***	30911575	F009	P009	/	6	Polish	Non-EA	NM_139242.4	c.994C>T	p.Arg332*	c.994C>T	p.Arg332*	AR	T+T	homo
**AP2213**	***MTFMT***	30911575	F010	P010	/	7	Polish	Non-EA	NM_139242.4	c.626C>T	p.Ser209Leu	c.994C>T	p.Arg332*	AR	M+T	compound
**AP2214**	***MTFMT***	30911575	F011	P011	/	8	Polish	Non-EA	NM_139242.4	c.626C>T	p.Ser209Leu	c.994C>T	p.Arg332*	AR	M+T	compound
**AP2215**	***MTFMT***	30911575	F012	P012	/	9	Polish	Non-EA	NM_139242.4	c.626C>T	p.Ser209Leu	c.994C>T	p.Arg332*	AR	M+T	compound
**AP2216**	***MTFMT***	30911575	F013	P013	/	10	American	Non-EA	NM_139242.4	c.626C>T	p.Ser209Leu	c.994C>T	p.Arg332*	AR	M+T	compound
**AP2217**	***MTFMT***	30911575	F014	P014	/	11	Netherlanderen	Non-EA	NM_139242.4	c.626C>T	p.Ser209Leu	c.994C>T	p.Arg332*	AR	M+T	compound
**AP2218**	***MTFMT***	30911575	F015	P015	/	12	Netherlanderen	Non-EA	NM_139242.4	c.626C>T	p.Ser209Leu	c.160G>A	p.Gly54Ser	AR	M+M	compound
**AP2219**	***MTFMT***	30911575	F016	P016	/	13	Netherlanderen	Non-EA	NM_139242.4	c.626C>T	p.Ser209Leu	c.160G>A	p.Gly54Ser	AR	M+M	compound
**AP2220**	***MTFMT***	30911575	F017	P017	/	14	Netherlanderen	Non-EA	NM_139242.4	c.626C>T	p.Ser209Leu	c.160G>A	p.Gly54Ser	AR	M+M	compound
**AP2221**	***MTFMT***	30369941	F018	P018	/	/	Caucasian	Non-EA	NM_139242.4	c.160G>A	p.Gly54Ser	c.626C>T	p.Ser209Leu	AR	M+M	compound
**AP2222**	***MTFMT***	28058511	F019	P019	/	P1	Canadian	Non-EA	NM_139242.4	c.626C>T	p.Ser209Leu	c.176C>T	p.Ala59Val	AR	M+M	compound
**AP2223**	***MTFMT***	27393152	F020	P020	/	/	Arabian	Non-EA	NM_139242.4	c.1116delT	p.Pro373Glnfs*19	c.1116delT	p.Pro373Glnfs	AR	T+T	homo
**AP2224**	***MTFMT***	25911677	F021	P021	/	P1	Canadian	Non-EA	NM_139242.4	c.626C>T	p.Ser209Leu	c.994C>T	p.Arg332*	AR	M+T	compound
**AP2225**	***MTFMT***	25911677	F022	P022	/	P2	Canadian	Non-EA	NM_139242.4	c. 242T>C	p.Leu81Pro	c.994C>T	p.Arg332*	AR	M+T	compound
**AP2226**	***MTFMT***	25911677	F023	P023	/	P3	Canadian	Non-EA	NM_139242.4	c.219_222del	p.Glu74Lysfs*3	c. 626C>T	p.Arg181Serfs	AR	T+T	compound
**AP2227**	***MTFMT***	25058219	F024	P024	/	18	Caucasian	Non-EA	NM_139242.4	c.452C>T	p.Pro151Leu	c.994C>T	p.Arg332*	AR	M+T	compound
**AP2228**	***MTFMT***	25058219	F025	P025	/	19	Caucasian	Non-EA	NM_139242.4	c.626C>T	p.Ser209Leu	c.1100_1101d	p.Phe367Serfs	AR	M+T	compound
**AP2229**	***MTFMT***	25044680	F026	P026	/	/	Arabian	Non-EA	NM_139242.4	c.17G>C	p.Arg6Pro	c.199 0del	p.Thr664Argfs	AR	M+T	compound
**AP2230**	***MTFMT***	24461907	F027	P027	/	33009	German	Non-EA	NM_139242.4	c.146_153del	p.Arg49Leufs*58	c. 626C>T	p.Arg181Serfs	AR	T+T	compound
**AP2231**	***MTFMT***	24461907	F028	P028	/	33467	German	Non-EA	NM_139242.4	c.146_153del	p.Arg49Leufs*58	c. 626C>T	p.Arg181Serfs	AR	T+T	compound
**AP2232**	***MTFMT***	24461907	F029	P029	/	49728	German	Non-EA	NM_139242.4	c. 626C>T	p.Arg181Serfs*5	c.878G>A	p.Ser293Asn	AR	M+T	compound
**AP2233**	***MTFMT***	24461907	F030	P030	/	52181	German	Non-EA	NM_139242.4	c.219_222del	p.Glu74Lysfs*3	c. 626C>T	p.Arg181Serfs	AR	T+T	compound
**AP2234**	***MTFMT***	24461907	F031	P031	/	54502	German	Non-EA	NM_139242.4	c.994C>T	p.Arg332*	c. 626C>T	p.Arg181Serfs	AR	T+T	compound
**AP2235**	***MTFMT***	24461907	F032	P032	/	56713	German	Non-EA	NM_139242.4	c.73C> T	p.Gln25*	c. 626C>T	p.Arg181Serfs	AR	T+T	compound
**AP2236**	***MTFMT***	24461907	F033	P033	/	56902	German	Non-EA	NM_139242.4	c.452C>T	p.Pro151Leu	c.994C>T	p.Arg332*	AR	M+T	compound
**AP2237**	***MTFMT***	24461907	F034	P034	/	73922	German	Non-EA	NM_139242.4	c. 626C>T	p.Arg181Serfs*5	c.452C>T	p.Pro151Leu	AR	M+T	compound
**AP2238**	***MTFMT***	24461907	F035	P035	/	8432723	German	Non-EA	NM_139242.4	c. 626C>T	p.Arg181Serfs*5	c.766C>T	p.Gln256*	AR	T+T	compound
**AP2239**	***MTFMT***	24215330	F036	P036	/	/	Caucasian	Non-EA	NM_139242.4	c. 626C>T	p.Arg181Serfs*5	c.998G>C	p.Ser333*	AR	T+T	compound
**AP2240**	***MTFMT***	23499752	F037	P037	/	P1	Caucasian	Non-EA	NM_139242.4	c.452C>T	p.Pro151Leu	c.994C>T	p.Arg332*	AR	M+T	compound
**AP2241**	***MTFMT***	23499752	F038	P038	/	P2	Caucasian	Non-EA	NM_139242.4	c.452C>T	p.Pro151Leu	c.994C>T	p.Arg332*	AR	M+T	compound
**AP2242**	***MTFMT***	23499752	F039	P039	/	/	Caucasian	Non-EA	NM_139242.4	c.452C>T	p.Pro151Leu	c.994C>T	p.Arg332*	AR	M+T	compound
**AP2243**	***MTFMT***	22499348	F040	P040	MITO049	44409	Caucasian	Non-EA	NM_139242.4	c.994C>T	p.Arg332*	c. 626C>T	p.Arg181Serfs	AR	T+T	compound
**AP2244**	***MTFMT***	22499348	F041	P041	MITO061	51506	Caucasian	Non-EA	NM_139242.4	c. 626C>T	p.Arg181Serfs*5	c. 626C>T	p.Arg181Serfs	AR	T+T	homo
**AP2245**	***MTFMT***	21907147	F042	P042	/	P1	Caucasian	Non-EA	NM_139242.4	c. 626C>T	p.Arg181Serfs*5	c.382C>T	p.Arg128*	AR	T+T	compound
**AP2246**	***MTFMT***	21907147	F043	P043	/	P2	Caucasian	Non-EA	NM_139242.4	c. 626C>T	p.Arg181Serfs*5	c.374C>T	p.Ser125Leu	AR	M+T	compound
**AP2247**	***MTPAP***	31779033	F001	P001	family F664	P1	European	Non-EA	NM_018109.4	c.1283T > C	p.Ile428Thr	c.1567C > T	p.Arg523Trp	AR	M+M	compound
**AP2248**	***MTPAP***	31779033	F001	P002	family F664	P2	European	Non-EA	NM_018109.4	c.1283T > C	p.Ile428Thr	c.1567C > T	p.Arg523Trp	AR	M+M	homo
**AP2249**	***MTPAP***	31779033	F002	P003	family F432	P3	Pakistani	Non-EA	NM_018109.4	c.1153A > T	p.Ile385Phe	c.1153A > T	p.Ile385Phe	AR	M+M	homo
**AP2250**	***MTPAP***	20970105	F003	P004	/	IX-2	Order Amish	Non-EA	NM_018109.4	c.1432A>G	p.Asn478Asp	c.1432A>G	p.Asn478Asp	AR	M+M	homo
**AP2251**	***MTPAP***	20970105	F3	P005	/	IX-3	Order Amish	Non-EA	NM_018109.4	c.1432A>G	p.Asn478Asp	c.1432A>G	p.Asn478Asp	AR	M+M	homo
**AP2252**	***MTPAP***	20970105	F003	P006	/	IX-4	Order Amish	Non-EA	NM_018109.4	c.1432A>G	p.Asn478Asp	c.1432A>G	p.Asn478Asp	AR	M+M	homo
**AP2253**	***MTPAP***	20970105	F003	P007	/	IX-5	Order Amish	Non-EA	NM_018109.4	c.1432A>G	p.Asn478Asp	c.1432A>G	p.Asn478Asp	AR	M+M	homo
**AP2254**	***MTPAP***	20970105	F003	P008	/	IX-6	Order Amish	Non-EA	NM_018109.4	c.1432A>G	p.Asn478Asp	c.1432A>G	p.Asn478Asp	AR	M+M	homo
**AP2255**	***MTPAP***	20970105	F003	P009	/	IX-7	Order Amish	Non-EA	NM_018109.4	c.1432A>G	p.Asn478Asp	c.1432A>G	p.Asn478Asp	AR	M+M	homo
**AP2256**	***MTPAP***	24651433	F004	P010	/		Amish	Non-EA	NM_018109.4	c.1432A>G	p.Asn478Asp	c.1432A>G	p.Asn478Asp	AR	M+M	homo
**AP2257**	***MTPAP***	25008111	F005	P011	/	P1	Amish	Non-EA	NM_018109.4	c.1432A>G	p.Asn478Asp	c.1432A>G	p.Asn478Asp	AR	M+M	homo
**AP2258**	***MTPAP***	25008111	F006	P012	/	P2	Amish	Non-EA	NM_018109.4	c.1432A>G	p.Asn478Asp	c.1432A>G	p.Asn478Asp	AR	M+M	homo
**AP2259**	***NBAS***	34490615	F001	P001	/	HH_NEO049	German	Non-EA	NM_015909.3	c.1528C>T	p.Arg510*	c.5741G>A	p.Arg1914His	AR	M+T	compound
**AP2260**	***NBAS***	34490615	F002	P002	/	MITO_HH006	German	Non-EA	NM_015909.3	c.767G>A	p.Cys256Tyr	c.2012T>G	p.Phe671Cys	AR	M+M	compound
**AP2261**	***NBAS***	34480364	F003	P003	/	151	Caucasian	Non-EA	NM_015909.3	c.2167C>T	p.Gln723*	/	/	AD	Truncation	heter
**AP2262**	***NBAS***	34427841	F004	P004	/	/	Polish	Non-EA	NM_015909.3	c.2809C>G	p.Pro937Ala	c.2809C>G	p.Pro937Ala	AR	M+M	homo
**AP2263**	***NBAS***	34396667	F005	P005	/	1	Turkish	Non-EA	NM_015909.3	c.3602A>C	p.Gln1201Pro	c.3602A>C	p.Gln1201Pro	AR	M+M	homo
**AP2264**	***NBAS***	34396667	F005	P006	/	2	Turkish	Non-EA	NM_015909.3	c.3602A>C	p.Gln1201Pro	c.3602A>C	p.Gln1201Pro	AR	M+M	homo
**AP2265**	***NBAS***	34396667	F006	P007	/	3	Turkish	Non-EA	NM_015909.3	c.1556T>A	p.Val519Glu	c.1556T>A	p.Val519Glu	AR	M+M	homo
**AP2266**	***NBAS***	34396667	F007	P008	/	4	Turkish	Non-EA	NM_015909.3	c.307G>A	p.Ala103Thro	c.5119T>A	p.Phe1707Ile	AR	M+M	compound
**AP2267**	***NBAS***	34396667	F008	P009	/	5	Turkish	Non-EA	NM_015909.3	c.1628_1629insA	p.Ser544ValfsTer	c.1226C>T	p.Ala409Val	AR	M+M	compound
**AP2268**	***NBAS***	34298581	F009	P010	/	P20	Chinese	EA	NM_015909.3	c.2630G>T	p.Gly877Val	c.4288C>T	p.Gln1430*	AR	M+M	compound
**AP2269**	***NBAS***	34288298	F010	P011	/	/	Caucasian	Non-EA	NM_015909.3	c.1549C>T	p.Arg517Cys	c.4646T>C	p.Leu1549Pro	AR	M+M	compound
**AP2270**	***NBAS***	34110364	F011	P012	/	/	Korean	EA	NM_015909.3	c.3493del	p.Val1165Serfs*3	c.5740C>T	p.Arg1914Cys	AR	M+T	compound
**AP2271**	***NBAS***	34023347	F012	P013	/	pat 3	Caucasian	Non-EA	NM_015909.3	c.4731_4733dup	p.Tyr1578dup	/	/	AD	M	heter
**AP2272**	***NBAS***	34023347	F013	P014	/	pat 9	Caucasian	Non-EA	NM_015909.3	c.exons 17-19 dup	p.?	/	/	AD	Truncation	heter
**AP2273**	***NBAS***	34023347	F014	P015	/	pat 12	Caucasian	Non-EA	NM_015909.3	c.1702G>A	p.Val568Ile	c.191G>A	p.Arg64His	AR	M+M	compound
**AP2274**	***NBAS***	33707149	F015	P016	/	/	Spanish	Non-EA	NM_015909.3	c.1265T>C	p.Leu422Pro	c.1549C>T	p.Arg517Cys	AR	M+M	compound
**AP2275**	***NBAS***	33520894	F016	P017	/	/	Chinese	EA	NM_015909.3	c.3596G> A	p.Cys1199Tyr	ex.9del	p.?	AR	M+M	compound
**AP2276**	***NBAS***	33270637	F017	P018	/	6	Caucasian	Non-EA	NM_015909.3	c.6311G>A	p.Arg2104Gln	c.1118T>C	p.Leu373Pro	AR	M+M	compound
**AP2277**	***NBAS***	33042920	F018	P019	/	patient 1	Russian	Non-EA	NM_015909.3	c.5741G>A	p.Arg1914His	ex. 35-47 del	p.?	AR	M+CNV	compound
**AP2278**	***NBAS***	33042920	F018	P020	/	patient 2	Russian	Non-EA	NM_015909.3	c.5741G>A	p.Arg1914His	ex. 35-47 del	p.?	AR	M+CNV	compound
**AP2279**	***NBAS***	33042920	F019	P021	/	patient 3	Russian	Non-EA	NM_015909.3	c.5741G>A	p.Arg1914His	c.1628_1629i	p.Ser544fs	AR	M+T	compound
**AP2280**	***NBAS***	32957979	F020	P022	/	/	Chinese	EA	NM_015909.3	c.3386C>T	p.Ser1129Phe	c.1A>C	p.Met1Leu	AR	M+M	compound
**AP2281**	***NBAS***	32812336	F021	P023	/	P1	Chinese	EA	NM_015909.3	c.426C>G	p.Tyr142Ter	c.3596G>A	p.Cys1199Tyr	AR	M+M	compound
**AP2282**	***NBAS***	32812336	F022	P024	/	P2	Chinese	EA	NM_015909.3	c.501delT	p.Phe167LeufsTe	c.2570C>T	p.Ala857Val	AR	M+T	compound
**AP2283**	***NBAS***	32812336	F023	P025	/	P3	Chinese	EA	NM_015909.3	c.648-1G>A	p.?	c.3596G>A	p.Cys1199Tyr	AR	M+T	compound
**AP2284**	***NBAS***	32812336	F024	P026	/	P4	Chinese	EA	NM_015909.3	c.1018G>C	p.Gly340Arg	c.5740C>T	p.Arg1914Cys	AR	M+M	compound
**AP2285**	***NBAS***	32812336	F025	P027	/	P5	Chinese	EA	NM_015909.3	c.1018G>C	p.His855_Gln859	c.3596G>A	p.Cys1199Tyr	AR	M+T	compound
**AP2286**	***NBAS***	32812336	F025	P028	/	P6	Chinese	EA	NM_015909.3	c.2563_c.2577+5del	p.His855_Gln859	c.3596G>A	p.Cys1199Tyr	AR	M+T	compound
**AP2287**	***NBAS***	32812336	F026	P029	/	P7	Chinese	EA	NM_015909.3	c.3284G>A	p.Trp1095*	c.3596G>A	p.Cys1199Tyr	AR	M+T	compound
**AP2288**	***NBAS***	32812336	F027	P030	/	P8	Chinese	EA	NM_015909.3	c.3432_3435dupCAGT	p.Ala1146GlnfsTe	c.6859G>T	p.Asp2287Tyr	AR	M+T	compound
**AP2289**	***NBAS***	32812336	F028	P031	/	P9	Chinese	EA	NM_015909.3	c.2423+404G>A	p.?	c.2822G>A	p.Arg941His	AR	M+T	compound
**AP2290**	***NBAS***	32812336	F029	P032	/	P10	Chinese	EA	NM_015909.3	c.680_690dupACTGTT	p.Phe231ThrfsTer	c.1361C>G	p.Pro454Arg	AR	M+T	compound
**AP2291**	***NBAS***	32812336	F030	P033	/	P11	Chinese	EA	NM_015909.3	c.1028G>A	p.Ser343Asn	c.5741G>A	p.Arg1914His	AR	M+M	compound
**AP2292**	***NBAS***	32812336	F031	P034	/	P12	Chinese	EA	NM_015909.3	c.1177_1182delinsAG	p.Val393ArgfsTer	c.5752A>C	p.Thr1918Pro	AR	M+T	compound
**AP2293**	***NBAS***	32812336	F032	P035	/	P13	Chinese	EA	NM_015909.3	c.2239T>C	p.Tyr747Asp	c.7003C>T	p.Arg2335Trp	AR	M+M	compound
**AP2294**	***NBAS***	32812336	F033	P036	/	P14	Chinese	EA	NM_015909.3	c.3115C>T	p.Gln1039*	c.7041_7043d	p.2346delLeu	AR	M+M	compound
**AP2295**	***NBAS***	32768688	F034	P037	/		Caucasian	Non-EA	NM_015909.3	c.2411A > G	p.Glu804Gly	c.1450C > T	p.Arg484*	AR	M+T	compound
**AP2296**	***NBAS***	32297715	F035	P038	/		Caucasian	Non-EA	NM_015909.3	c.17C>A	p.Ser6*	c.5741G>A	p.Arg1914His	AR	M+T	compound
**AP2297**	***NBAS***	32146038	F036	P039	/	p2	Caucasian	Non-EA	NM_015909.3	c.2822G>A	p.Arg1914His	c.2822G>A	p.Arg1914His	AR	M+M	homo
**AP2298**	***NBAS***	32146038	F037	P040	/	p3	Caucasian	Non-EA	NM_015909.3	c.2708T>G	p.Leu903Arg	c.2708T>G	p.Leu903Arg	AR	M+M	homo
**AP2299**	***NBAS***	31889758	F038	P041	/		Caucasian	Non-EA	NM_015909.3	c.761G>A	p.Gly254Asp	c.761G>A	p.Gly254Asp	AR	M+M	homo
**AP2300**	***NBAS***	31761904	F039	P042	/	46	Caucasian	Non-EA	NM_015909.3	c.284C>T	p.Ala95Val	c.2802G>A	p.Trp934*	AR	M+T	compound
**AP2301**	***NBAS***	31761904	F040	P043	/	82	Caucasian	Non-EA	NM_015909.3	c.812T>C	p.Leu271Pro	c.812T>C	p.Leu271Pro	AR	M+M	homo
**AP2302**	***NBAS***	31761904	F041	P044	/	7	Caucasian	Non-EA	NM_015909.3	c.1241C>T	p.Ser414Phe	c.2950delA	p.Ile984Leufs*	AR	M+T	compound
**AP2303**	***NBAS***	31761904	F042	P045	/	8	Caucasian	Non-EA	NM_015909.3	c.1241C>T	p.Ser414Phe	c.[6236+1_62	p.Glu2080*	AR	M+T	compound
**AP2304**	***NBAS***	31761904	F043	P046	/	13	Caucasian	Non-EA	NM_015909.3	c.1549C>T	p.Arg517Cys	c.5041_5048d	p.Ser1681Glnf	AR	M+T	compound
**AP2305**	***NBAS***	31761904	F044	P047	/	12	Caucasian	Non-EA	NM_015909.3	c.1550G>A	p.Arg517His	c.6805G>T	p.Glu2269*	AR	M+T	compound
**AP2306**	***NBAS***	31761904	F045	P048	/	64	Caucasian	Non-EA	NM_015909.3	c.1550G>A	p.Arg517His	c.6805G>T	p.Glu2269*	AR	M+T	compound
**AP2307**	***NBAS***	31761904	F046	P049	/	51	Caucasian	Non-EA	NM_015909.3	c.2191A>C	p.Thr731Pro	c.2191A>C	p.Thr731Pro	AR	M+M	homo
**AP2308**	***NBAS***	31761904	F047	P050	/	55	Caucasian	Non-EA	NM_015909.3	c.2330C>A	p.Pro777His	c.2330C>A	p.Pro777His	AR	M+M	homo
**AP2309**	***NBAS***	31761904	F048	P051	/	56	Caucasian	Non-EA	NM_015909.3	c.2330C>A	p.Pro777His	c.2330C>A	p.Pro777His	AR	M+M	homo
**AP2310**	***NBAS***	31761904	F049	P052	/	68	Caucasian	Non-EA	NM_015909.3	c.2809C>G	p.Pro937Ala	c.[5138 + 1_51	p.?	AR	M+T	compound
**AP2311**	***NBAS***	31761904	F050	P053	/	51	Caucasian	Non-EA	NM_015909.3	c.2819A>C	p.His940Pro	c.2819A>C	p.His940Pro	AR	M+M	homo
**AP2312**	***NBAS***	31761904	F051	P054	/	9	Caucasian	Non-EA	NM_015909.3	c.2951T>G	p.Ile984Ser	c.2827G>T	p.Glu943*	AR	M+T	compound
**AP2313**	***NBAS***	31761904	F052	P055	/	69	Caucasian	Non-EA	NM_015909.3	c.3164T>C	p.Leu1055Pro	c.[5027 + 1_5	p.Glu1676Asp	AR	M+T	compound
**AP2314**	***NBAS***	31761904	F053	P056	/	84	Caucasian	Non-EA	NM_015909.3	c.3363A>G	p.Ile1121Met	c.513+2T>C	p.?	AR	M+T	compound
**AP2315**	***NBAS***	31761904	F054	P057	/	58	Caucasian	Non-EA	NM_015909.3	c.3386C>T	p.Ser1129Phe	c.3386C>T	p.Ser1129Phe	AR	M+M	homo
**AP2316**	***NBAS***	31761904	F055	P058	/	59	Caucasian	Non-EA	NM_015909.3	c.3386C>T	p.Ser1129Phe	c.3386C>T	p.Ser1129Phe	AR	M+M	homo
**AP2317**	***NBAS***	31761904	F056	P059	/	60	Caucasian	Non-EA	NM_015909.3	c.3534C>A	p.Ser1178Arg	c.1342-6A>G	p.?	AR	M+T	compound
**AP2318**	***NBAS***	31761904	F057	P060	/	53	Caucasian	Non-EA	NM_015909.3	c.3602A>C	p.Gln1201Pro	c.3602A>C	p.Gln1201Pro	AR	M+M	homo
**AP2319**	***NBAS***	31761904	F058	P061	/	50	Caucasian	Non-EA	NM_015909.3	c.5741G>A	p.Arg1914His	c.1528C>T	p.Arg510*	AR	M+T	compound
**AP2320**	***NBAS***	31761904	F059	P062	/	62	Caucasian	Non-EA	NM_015909.3	c.5740C>G	p.Arg1914Gly	c.6877delC	p.Leu2293Cys	AR	M+T	compound
**AP2321**	***NBAS***	31761904	F060	P063	/	14	Caucasian	Non-EA	NM_015909.3	c.5761G>C	p.Ala1921Pro	c.686dupT	p.Ser230Glnfs	AR	M+T	compound
**AP2322**	***NBAS***	31761904	F061	P064	/	11	Caucasian	Non-EA	NM_015909.3	c.6840G>T	p.?	c.6840G>T	p.?	AR	T+T	homo
**AP2323**	***NBAS***	31761904	F062	P065	/	57	Caucasian	Non-EA	NM_015909.3	c.6966_6969delinsTC	p.Gln2322Hisfs*1	c.6966_6969	p.Gln2322Hisf	AR	T+T	homo
**AP2324**	***NBAS***	31761904	F063	P066	/	61	Caucasian	Non-EA	NM_015909.3	c.767G>A	pCys256Tyr	c.2012T>G	pPhe671Cys	AR	M+M	compound
**AP2325**	***NBAS***	31761904	F064	P067	/	83	Caucasian	Non-EA	NM_015909.3	c.2535G>T	pTrp845Cys	c.5761G>C	pAla1921Pro	AR	M+M	compound
**AP2326**	***NBAS***	31507590	F065	P068	/		Pakistani	Non-EA	NM_015909.3	c.1948C> T	p.Pro650Ser	c.1948C>T	p.Pro650Ser	AR	M+M	homo
**AP2327**	***NBAS***	31216810	F066	P069	/		Chinese	EA	NM_015909.3	c.7041_7043del	p.2347_2348del	c.3759delC	p.T1254Hfs*16	AR	M+T	compound
**AP2328**	***NBAS***	31015584	F067	P070	/	patient 1	Turkish	Non-EA	NM_015909.3	c.5741G>A	pArg1914His	c.405G>A	pTrp135*	AR	M+T	compound
**AP2329**	***NBAS***	31015584	F068	P071	/	patient 2	Caucasian	Non-EA	NM_015909.3	c.2950delA	pIle984Leufs*8	c.3158A>G	pHis1053Arg	AR	M+T	compound
**AP2330**	***NBAS***	31015584	F069	P072	/	patient 3	Caucasian	Non-EA	NM_015909.3	c.5741G>A	pArg1914His	c.6564dupT	pGlu2189*	AR	M+T	compound
**AP2331**	***NBAS***	30825388	F070	P074	/		Caucasian	Non-EA	NM_015909.3	c.1501C>T	p.R501*	c.6840G>A	p.?	AR	T+T	compound
**AP2332**	***NBAS***	30825388	F071	P075	/		Italian	Non-EA	NM_015909.3	c.686_687insT	p.S230Qfs*4	c.6840G>A	p.?	AR	T+T	compound
**AP2333**	***NBAS***	30622725	F072	P076	/		Japanese	EA	NM_015909.3	c.1018G>C	p.Gly340Arg	c.2674G>T	p.Val892Phe	AR	M+M	compound
**AP2334**	***NBAS***	30592236	F073	P077	/		Caucasian	Non-EA	NM_015909.3	c.5752A>C	p.Thr1918Pro	c.500-501delT	p.Phe167Cysf	AR	M+T	compound
**AP2335**	***NBAS***	30558828	F074	P078	/		Caucasian	Non-EA	NM_015909.3	c.2617C>T	p.Arg873Trp	c.4475A>G	p.Tyr1492Cys	AR	M+M	compound
**AP2336**	***NBAS***	30369941	F075	P079	/		Caucasian	Non-EA	NM_015909.3	c.1556T>A	p.Val519Gln	c.1556T>A	p.Val519Gln	AR	M+M	homo
**AP2337**	***NBAS***	29929043	F076	P080	/	The younger si	Italian	Non-EA	NM_015909.3	c.6237-3C>G	p.?	c.6237-3C>G	p.?	AR	T+T	homo
**AP2338**	***NBAS***	29575310	F077	P081	/		Korean	EA	NM_015909.3	c.1339G>A	p.Glu447Lys	c.5454_5455d	p.Leu1818fsX	AR	p.?	compound
**AP2339**	***NBAS***	29262476	F078	P082	/		Chinese	EA	NM_015909.3	c.5741C>T	p.Arg1914His	c.6496_6497i	p.S2166Ffs*2	AR	p.?	compound
**AP2340**	***NBAS***	28946922	F079	P083	/		Italian	Non-EA	NM_015909.3	c.809G > C	p.Gly270Ala	c.2926del	p.Ser976Profs	AR	M+T	compound
**AP2341**	***NBAS***	28629372	F080	P084	/	1	Chinese	EA	NM_015909.3	c.6611_6612insCA	p. Met2204Ilefs*3	c.3596G>A	p.Cys1199Tyr	AR	M+T	compound
**AP2342**	***NBAS***	28629372	F081	P085	/	2	Chinese	EA	NM_015909.3	c.3596G>A	p.Cys1199Tyr	c.586C>T	p.Gln196*	AR	M+T	compound
**AP2343**	***NBAS***	28629372	F082	P086	/	3	Chinese	EA	NM_015909.3	c.5389+1G>T	p.?	c.2407G>A	p.Glu803Lys	AR	M+T	compound
**AP2344**	***NBAS***	29207168	F083	P089	/		Chinese	EA	NM_015909.3	c.3596G>A	p.Cys1199Tyr	c.209+1G>A	/	AR	M+T	compound
**AP2345**	***NBAS***	28576691	F084	P090	/	patient 1	Caucasian	Non-EA	NM_015909.3	c.680A>C	p.His227Pro	c.1749G>A	p.Trp583*	AR	M+T	compound
**AP2346**	***NBAS***	28576691	F085	P091	/	patient 2	Caucasian	Non-EA	NM_015909.3	c.680A>C	p.His227Pro	c.1749G>A	p.Trp583*	AR	M+T	compound
**AP2347**	***NBAS***	28115293	F086	P092	/		Korean	EA	NM_015909.3	c. 5741G>A	pArg1914His	c. 5741G>A	pArg1914His	AR	M+M	homo
**AP2348**	***NBAS***	28115293	F086	P093	/		Korean	EA	NM_015909.3	c. 5741G>A	pArg1914His	c. 5741G>A	pArg1914His	AR	M+M	homo
**AP2349**	***NBAS***	28031453	F087	P094	/	1	Caucasian	Non-EA	NM_015909.3	c.2827G>T	p.Glu943*	c.5741G>A	p.Arg1914His	AR	M+T	compound
**AP2350**	***NBAS***	27789416	F088	P095	/	patient 1	North European	Non-EA	NM_015909.3	c. 5741G>A	pArg1914His	c.3010C>T	pArg1004*	AR	M+T	compound
**AP2351**	***NBAS***	27789416	F089	P096	/	patient 2	Northern-Spanish	Non-EA	NM_015909.3	c. 5741G>A	pArg1914His	c.2032C>T	pGlu678*	AR	M+T	compound
**AP2352**	***NBAS***	26578240	F090	P097	/	II-1	Caucasian	Non-EA	NM_015909.3	c.409C>T	p.Arg137Trp	c.409C>T	p.Arg137Trp	AR	M+M	homo
**AP2353**	***NBAS***	26578240	F090	P098	/	II-2	Caucasian	Non-EA	NM_015909.3	c.409C>T	p.Arg137Trp	c.409C>T	p.Arg137Trp	AR	M+M	homo
**AP2354**	***NBAS***	26578240	F090	P099	/	II-3	Caucasian	Non-EA	NM_015909.3	c.409C>T	p.Arg137Trp	c.409C>T	p.Arg137Trp	AR	M+M	homo
**AP2355**	***NBAS***	26541327	F091	P111	FXI	Patient 12	Caucasian	Non-EA	NM_015909.3	c.2827G>T	p.Glu943*	exons 39–40 d	p.Val2145_Glu	AR	M+T	compound
**AP2356**	***NBAS***	26541327	F092	P112	FXII	Patient 13	Caucasian	Non-EA	NM_015909.3	c.1278A>C	p.Cys426Trp	exons 49–50 d	p.Val1528Glyf	AR	M+T	compound
**AP2357**	***NBAS***	26541327	F093	P113	FXIII	Patient 14	Caucasian	Non-EA	NM_015909.3	c.173-2A>G	p.?	c.3363A>G	p.Ile1121Met	AR	M+T	compound
**AP2358**	***NBAS***	26286438	F094	P116	/	Patient 1	Swiss	Non-EA	NM_015909.3	c.284C>T	p.Ala95Val	c.850A>T	p.Lys284*	AR	M+T	compound
**AP2359**	***NBAS***	26286438	F095	P117	/	Patient 2	German	Non-EA	NM_015909.3	c.409C>T	p.Arg137Trp	c.1186T>A	p.Trp396Arg	AR	M+M	compound
**AP2360**	***NBAS***	26073778	F096	P118	F1	II-1	German	Non-EA	NM_015909.3	c.558_560del	p.Ile187del	c.686dup	p.Ser230Glnfs	AR	M+T	compound
**AP2361**	***NBAS***	26073778	F097	P119	F2	II-1	German	Non-EA	NM_015909.3	c.2708T>G	p.Leu903Arg	c.2708T>G	p.Leu903Arg	AR	M+M	homo
**AP2362**	***NBAS***	26073778	F098	P120	F3	II-1	German	Non-EA	NM_015909.3	c.603_605del	p.Leu202del	c.3164T>C	p.Leu1055Pro	AR	M+M	compound
**AP2363**	***NBAS***	26073778	F099	P121	F4	II-3	German	Non-EA	NM_015909.3	c.2708T>G	p.Leu903Arg	c.2827G>T	p.Glu943*	AR	M+T	compound
**AP2364**	***NBAS***	26073778	F100	P122	F5	II-2	German	Non-EA	NM_015909.3	c.3010C>T	p.Arg1004*	c.3164T>C	p.Leu1055Pro	AR	M+T	compound
**AP2365**	***NBAS***	26073778	F101	P123	F6	II-1	German	Non-EA	NM_015909.3	c.1533_1545del	p.Ile512Thrfs*4	c.2951T>G	p.Ile984Ser	AR	M+T	compound
**AP2366**	***NBAS***	26073778	F102	P124	F7	II-1	German	Non-EA	NM_015909.3	c.1042C>T	p.Arg137Trp	c.2203-3C>G	p.?	AR	M+T	compound
**AP2367**	***NBAS***	26073778	F103	P125	F7	II-2	German	Non-EA	NM_015909.3	c.1042C>T	p.Arg137Trp	c.2203-3C>G	p.?	AR	M+T	compound
**AP2368**	***NBAS***	26073778	F104	P126	F8	II-2	German	Non-EA	NM_015909.3	c.1187G>A	p.Trp396*	c.2330C>A	p.Pro777His	AR	M+T	compound
**AP2369**	***NBAS***	26073778	F105	P127	F9	II-2	German	Non-EA	NM_015909.3	c.118-2A>G	p.?	c.2524G>T	p.Val842Phe	AR	M+T	compound
**AP2370**	***NBAS***	26073778	F106	P128	F10	II-1	German	Non-EA	NM_015909.3	c.686dup	p.Ser230Glnfs*4	c.3164T>C	p.Leu1055Pro	AR	M+T	compound
**AP2371**	***NDUFA10***	35379322	F001	P001	/	R51757	Italian	Non-EA	NM_004544.3	c.-99_-75del	p.?	c.-99_-75del	p.?	AR	T+T	homo
**AP2372**	***NDUFA10***	35379322	F001	P002	/	R21147	Caucasian	Non-EA	NM_004544.3	c.-99_-75del	p.?	c.-99_-75del	p.?	AR	T+T	homo
**AP2373**	***NDUFA10***	28247337	F003	P003	/		Italian	Non-EA	NM_004544.3	c.296G>A	p.Gly99Glu	c.296G>A	p.Gly99Glu	AR	M+M	homo
**AP2374**	***NDUFA10***	26741492	F004	P004	/		Japanese	EA	NM_004544.3	c.383_384insTAA	p.Asn127_Ser128	c.881T>C	p.Leu294Pro	AR	M+T	compound
**AP2375**	***NDUFA10***	22200994	F005	P005	/		Caucasian	Non-EA	NM_004544.3	c.296G>A	p.Gly99Glu	c.296G>A	p.Gly99Glu	AR	M+M	homo
**AP2376**	***NDUFA10***	21150889	F006	P005	/		Caucasian	Non-EA	NM_004544.3	c.1A>G	p.Met1?	c.425A>G	p.Gln142Arg	AR	M+T	compound
**AP2377**	***NDUFA10***	35094435	F007	P006	/		Chinese	EA	NM_004544.3	c.764T>A	p.Val255Asp	c.311del	p.(Leu104Prof	AR	M+T	compound
**AP2378**	***NDUFAF4***	28853723	F003	P004	/		Pakistani	Non-EA		c.7G>C	p.Ala3Pro	c.7G>C	p.Ala3Pro	AR	M+M	homo
**AP2379**	***NDUFAF2***	34234304	F001	P001	/	ATX787	Caucasian	Non-EA		c.221G>A	p.Trp74*	c.221G>A	p.Trp74*	AR	T+T	Homo
**AP2380**	***NDUFAF2***	31130284	F002	P002	/	18-2409	Caucasian	Non-EA		c.139C>T	p.Arg47*	c.139C>T	p.Arg47*	AR	T+T	Homo
**AP2381**	***NDUFAF2***	30634555	F003	P003	/	/	Caucasian	Non-EA		deletion		deletion		AR	CNV+CNV	Homo
**AP2382**	***NDUFAF2***	26795593	F004	P004	/	/	Caucasian	Non-EA		c.139C>T	p.Arg47*	c.139C>T	p.Arg47*	AR	T+T	Homo
**AP2383**	***NDUFAF2***	25590979	F005	P005	/	/	Caucasian	Non-EA		c.1A>T	p.Met1?	c.1A>T	p.Met1?	AR	T+T	Homo
**AP2384**	***NDUFAF2***	22644603	F006	P006	/	/	Lebanese	Non-EA		deletion		deletion		AR	CNV+CNV	Homo
**AP2385**	***NDUFAF2***	20571988	F007	P007	/	/	Australian	Non-EA		c.9G>A	p.Trp3*	c.9G>A	p.Trp3*	AR	T+T	Homo
**AP2386**	***NDUFAF2***	19384974	F008	P009	/	/	Caucasian	Non-EA		c.114C>G	p.Tyr38*	c.114C>G	p.Tyr38*	AR	T+T	Homo
**AP2387**	***NDUFAF2***	16200211	F009	P010	/	/	Caucasian	Non-EA		c.139C>T	p.Arg47*	c.139C>T	p.Arg47*	AR	T+T	Homo
**AP2388**	***NDUFAF2***	18180188	F010	P011	/	Patient 1	Arab-Moslem	Non-EA		c.1A>T	p.Met1?	c.1A>T	p.Met1?	AR	T+T	Homo
**AP2389**	***NDUFAF2***	18180188	F011	P012	/	Patient 2	Caucasian	Non-EA		c.1A>T	p.Met1?	c.1A>T	p.Met1?	AR	T+T	Homo
**AP2390**	***NDUFAF2***	20818383	F012	P013	/	DT67	Caucasian	Non-EA		c.103delA	p.Ile35serfsX17	c.103delA	p.Ile35serfsX1	AR	T+T	Homo
**AP2391**	***NDUFAF2***	27344355	F013	P014	/	patient 3	Caucasian	Non-EA		deletion		deletion		AR	CNV+CNV	Homo
**AP2392**	***NDUFAF4***	18179882	F001	P001	/	F511	Arab- Muslim	Non-EA		c.194T>C	p.Leu65Pro	c.194T>C	p.Leu65Pro	AR	M+M	homo
**AP2393**	***NDUFAF4***	18179882	F001	P002	/	F334	Arab- Muslim	Non-EA		c.194T>C	p.Leu65Pro	c.194T>C	p.Leu65Pro	AR	M+M	homo
**AP2394**	***NDUFAF4***	22200994	F002	P003	/	33006	European	Non-EA		c.23G>A	p.Gly8Asp	c.23G>A	p.Gly8Asp	AR	M+M	homo
**AP2395**	***NDUFAF4***	30369941	F004	P005	/		Caucasian	Non-EA		c.23G>A	p.Gly8Asp	c.23G>A	p.Gly8Asp	AR	M+M	homo
**AP2396**	***NDUFAF4***	32949790	F005	P006	/		Caucasian	Non-EA		c.558G>T	p.Glu160*	c.558G>T	p.Glu160*	AR	T+T	homo
**AP2397**	***NDUFAF4***	32949790	F006	P007	/		Caucasian	Non-EA		c.558G>T	p.Glu160*	c.558G>T	p.Glu160*	AR	T+T	homo
**AP2398**	***NDUFAF5***	35598585	F001	P001	/	/	Caucasian	Non-EA	NM_024120.4	c.155A>C	p.Lys52Thr	c.752T>G;	p.Met251Arg	AR	M+M	Compd he[illegible]
**AP2399**	***NDUFAF5***	35379322	F002	P002	/	R33391	Caucasian	Non-EA	NM_024120.4	c.605dup	p.Leu203Valfs*22	c.223 - 907A>	p.?	AR	M+T	Compd he[illegible]
**AP2400**	***NDUFAF5***	35094435	F003	P003	/	BLGP0026	Chinese	EA	NM_024120.4	c.425A>C	p.Glu142Ala	c.425A>C	p.Glu142Ala	AR	M+M	Homo
**AP2401**	***NDUFAF5***	35094435	F004	P004	/	BLGP0070	Chinese	EA	NM_024120.4	c.212T>C	p.Leu71Pro	c.698G>T	p.Gly233Val	AR	M+M	Compd he[illegible]
**AP2402**	***NDUFAF5***	35094435	F005	P005	/	BLGP0141	Chinese	EA	NM_024120.4	c.836T>G	p.Met279Arg	c.1019C>T	p.Pro340Leu	AR	M+M	Compd he[illegible]
**AP2403**	***NDUFAF5***	35094435	F006	P006	/	BLGP0142	Chinese	EA	NM_024120.4	c.163C>A	p.Gln55Lys	c.425A>C	p.Glu142Ala	AR	M+M	Compd he[illegible]
**AP2404**	***NDUFAF5***	34988976	F007	P007	/	P8	Caucasian	Non-EA	NM_024120.4	c.477A>C	p.Leu159Phe	c.477A>C	p.Leu159Phe	AR	M+M	Homo
**AP2405**	***NDUFAF5***	34964562	F008	P008	/	/	Caucasian	Non-EA	NM_024120.4	c.357C>G	p.Asp119Glu	c.611C>T	p.A204V	AR	M+M	Compd he[illegible]
**AP2406**	***NDUFAF5***	34797029	F009	P009	/	/	Africa American	Non-EA	NM_024120.4	c.144C>A	p.Asp48Glu	c. 480-3T>G	p.?	AR	M+T	Compd he[illegible]
**AP2407**	***NDUFAF5***	34177781	F010	P010	/	II-2	Chinese	EA	NM_024120.4	c.425A>C	p.Glu142Ala	c.836T > G	p.Met279Arg	AR	M+M	Compd he[illegible]
**AP2408**	***NDUFAF5***	34177781	F010	P011	/	II-1	Chinese	EA	NM_024120.4	c.425A>C	p.Glu142Ala	c.836T > G	p.Met279Arg	AR	M+M	Compd he[illegible]
**AP2409**	***NDUFAF5***	34177781	F010	P012	/	II-3	Chinese	EA	NM_024120.4	c.425A>C	p.Glu142Ala	c.836T > G	p.Met279Arg	AR	M+M	Compd he[illegible]
**AP2410**	***NDUFAF5***	32918965	F011	P013	/	/	Caucasian	Non-EA	NM_024120.4	c.24G>A	p.Trp8*	c.827G>A	p.Arg276Gln	AR	M+T	Compd he[illegible]
**AP2411**	***NDUFAF5***	32348839	F012	P014	/	55	Chinese	EA	NM_024120.4	c.562A>G	p.Met188Val	c.519+4A > G	p.?	AR	M+T	Compd he[illegible]
**AP2412**	***NDUFAF5***	30473481	F013	P015	/	patient 1	Chinese	EA	NM_024120.4	c.155A>C	p.Lys52ThrLys52	c.836T>G	p.Met279Arg	AR	M+M	Compd he[illegible]
**AP2413**	***NDUFAF5***	30473481	F014	P016	/	patient 2	Chinese	EA	NM_024120.4	c.836T>G	p.Met279ArgMet2	c.836T>G	p.Met279Arg	AR	M+M	Homo
**AP2414**	***NDUFAF5***	30473481	F015	P017	/	patient 3	Caucasian	Non-EA	NM_024120.4	c.327G>C	p.Lys109AsnLys1	c.223-907A>C	p.?	AR	M+T	Compd he[illegible]
**AP2415**	***NDUFAF5***	30473481	F016	P018	/	patient 4	Ashkenazi Jewish	Non-EA	NM_024120.4	c.327G > C	p.Lys109Asn	c.749G > T	p.Gly250Val	AR	M+T	Compd he[illegible]
**AP2416**	***NDUFAF5***	30225196	F018	P020	/	V:3	Caucasian	Non-EA	NM_024120.4	c.230C > T	p.Thr77IleThr77I	c.230C > T	p.Thr77Ile	AR	M+M	Compd he[illegible]
**AP2417**	***NDUFAF5***	30225196	F018	P021	/	V:4	Caucasian	Non-EA	NM_024120.4	c.230C > T	p.Thr77IleThr77I	c.230C > T	p.Thr77Ile	AR	M+T	Compd he[illegible]
**AP2418**	***NDUFAF5***	29581464	F019	P022	/	P_16	Chinese	EA	NM_024120.4	c.836T>G	p.Met279ArgMet2	c.145C>G	p.Arg49Gly	AR	M+T	Compd he[illegible]
**AP2419**	***NDUFAF5***	26275793	F020	P023	/	6	Caucasian	Non-EA	NM_024120.4	c.29T>A	p.Leu10*	c.782T>G	p.Met261Arg	AR	M+M	Homo
**AP2420**	***NDUFAF5***	24215330	F021	P024	/	/	Caucasian	Non-EA	NM_024120.4	c.164A>G	p.Gln55ArgGln55	c.327+3A>G	p.?	AR	M+M	Homo
**AP2421**	***NDUFAF5***	21607760	F022	P025	Family I	1	Ashkenazi Jewish	Non-EA	NM_024120.4	c.749G>T	p.Gly250Val	c.749G>T	p.Gly250Val	AR	M+M	Homo
**AP2422**	***NDUFAF5***	21607760	F022	P026	Family I	2	Ashkenazi Jewish	Non-EA	NM_024120.4	c.749G>T	p.Gly250Val	c.749G>T	p.Gly250Val	AR	M+M	Homo
**AP2423**	***NDUFAF5***	21607760	F023	P027	Family II	3	Ashkenazi Jewish	Non-EA	NM_024120.4	c.749G>T	p.Gly250Val	c.749G>T	p.Gly250Val	AR	M+M	Homo
**AP2424**	***NDUFAF5***	21607760	F023	P028	Family II	4	Ashkenazi Jewish	Non-EA	NM_024120.4	c.749G>T	p.Gly250Val	c.749G>T	p.Gly250Val	AR	M+M	Homo
**AP2425**	***NDUFAF5***	21607760	F023	P029	Family II	5	Ashkenazi Jewish	Non-EA	NM_024120.4	c.749G>T	p.Gly250Val	c.749G>T	p.Gly250Val	AR	M+M	Homo
**AP2426**	***NDUFAF5***	19542079	F024	P030	/	IV:7	Moroccan	Non-EA	NM_024120.4	c.477A>C	p.Leu159Phe	c.477A>C	p.Leu159Phe	AR	M+M	Homo
**AP2427**	***NDUFAF5***	19542079	F024	P031	/	IV:9	Moroccan	Non-EA	NM_024120.4	c.477A>C	p.Leu159Phe	c.477A>C	p.Leu159Phe	AR	M+M	homo
**AP2428**	***NDUFAF5***	18940309	F025	P032	/	/	Egyptian	Non-EA	NM_024120.4	c.686T>C	p.Leu229Pro	c.686T>C	p.Leu229Pro	AR	M+M	homo
**AP2429**	***NDUFB11***	34490615	F001	P001	/	Paed292	Caucasian	Non-EA	/	c.389G>A	p.Arg130His	/	/	AD	M	hemi
**AP2430**	***NDUFB11***	30423443	F002	P002	/	1	Caucasian	Non-EA	/	c.254G>A	p.Trp85*	/	/	AD	Truncation	hemi
**AP2431**	***NDUFB11***	30423443	F003	P003	/	2	Caucasian	Non-EA	/	c.262C>T	p.Arg88*	/	/	AD	Truncation	hemi
**AP2432**	***NDUFB11***	30423443	F004	P004	/	3	Caucasian	Non-EA	/	c.262C>T	p.Arg88*	/	/	AD	Truncation	hemi

AP2433	NDUFB11	30423443	F005	P005	/	4	Caucasian	Non-EA	/	c.276_278delCTT	p.Phe93del	/	/	AD	M	homozygo	Male	Mitochondrial disorders	/	/	/	/	/	/	/	/	/	/	/	/	/	Fetal tachycardia	/	/	/	/	/	9.00	YES	/	/	/	/	/	/	/
AP2434	NDUFB11	30423443	F006	P006	/	5	Caucasian	Non-EA	/	c.276_278delCTT	p.Phe93del	/	/	AD	M	homozygo	Male	Mitochondrial disorders	/	/	/	/	/	/	/	/	/	/	/	/	/	/	/	/	/	/	/	2.00	YES	/	/	/	/	/	/	/
AP2435	NDUFB11	30423443	F007	P007	/	6	Caucasian	Non-EA	/	c.276_278delCTT	p.Phe93del	/	/	AD	M	homozygo	Male	Mitochondrial disorders	/	/	/	/	/	/	/	/	/	/	/	Myopathy HP:00(	/	/	/	/	/	/	/	23.00	YES	/	/	/	/	/	/	/
AP2436	NDUFB11	30423443	F008	P008	/	7	Caucasian	Non-EA	/	c.276_278delCTT	p.Phe93del	/	/	AD	M	homozygo	Male	Mitochondrial disorders	/	/	/	Optic atrophy HP	/	/	/	/	/	/	/	/	/	/	/	/	/	/	/	20.00	YES	/	/	/	/	/	/	/
AP2437	NDUFB11	30423443	F009	P009	/	8	Caucasian	Non-EA	/	c.276_278delCTT	p.Phe93del	/	/	AD	M	homozygo	Male	Mitochondrial disorders	/	/	/	Optic atrophy HP	/	/	/	/	/	/	/	/	/	/	/	/	/	/	/	16.00	YES	/	/	/	/	/	/	/
AP2438	NDUFB11	30423443	F010	P010	/	9	Caucasian	Non-EA	/	c.276_278delCTT	p.Phe93del	/	/	AD	M	homozygo	Male	Mitochondrial disorders	/	/	/	/	/	/	/	/	/	/	/	/	/	/	/	/	/	/	/	8.00	YES	/	/	/	/	/	/	/
AP2439	NDUFB11	30423443	F011	P011	/	10	Caucasian	Non-EA	/	c.286T>C	p.Ser96Pro	/	/	AD	M	homozygo	Male	Mitochondrial disorders	/	/	/	Optic atrophy HP	/	/	/	/	/	/	/	/	/	Cardiomyopathy	/	/	/	/	/	15.00	YES	/	/	/	/	/	/	/
AP2440	NDUFB11	30423443	F012	P012	/	11	Caucasian	Non-EA	/	c.324T>G	p.Tyr108*	/	/	AD	Truncation	hemi	Female	Mitochondrial disorders	/	/	/	/	/	/	/	/	/	/	/	/	/	Cardiomyopathy	/	/	/	/	/	6.00	YES	/	/	/	/	/	/	/
AP2441	NDUFB11	30423443	F013	P013	/	12	Caucasian	Non-EA	/	c.328C>T	p.Pro110Ser	/	/	AD	M	homozygo	Male	Mitochondrial disorders	/	/	/	/	/	/	/	/	/	/	/	/	/	Cardiomyopathy	/	/	/	/	/	3.00	YES	/	/	/	/	/	/	/
AP2442	NDUFB11	30423443	F014	P014	/	13	Caucasian	Non-EA	/	c.361G>A	p.Glu121Lys	/	/	AD	M	homozygo	Male	Mitochondrial disorders	/	/	/	/	/	/	/	/	/	/	/	/	/	Cardiomyopathy	/	/	/	/	/	0.00	YES	/	/	/	/	/	/	/
AP2443	NDUFB11	30423443	F015	P015	/	14	Caucasian	Non-EA	/	c.402delG	p.Arg134Serfs*3	/	/	AD	Truncation	hemi	Female	Mitochondrial disorders	/	/	/	/	/	/	Nystagmus HP:0(	/	/	/	/	/	/	Cardiomyopathy	/	/	/	/	/	5.00	YES	/	/	/	/	/	/	/
AP2444	NDUFB11	30423443	F016	P016	/	15	Caucasian	Non-EA	/	c.402delG	p.Arg134Serfs*3	/	/	AD	Truncation	hemi	Female	Mitochondrial disorders	/	/	/	/	/	/	/	/	/	/	/	/	/	Cardiomyopathy	/	/	/	/	/	0.00	NO	/	/	/	/	/	/	/
AP2445	NDUFB11	28050600	F017	P017	/		Caucasian	Non-EA	/	c.262C>T	p.Arg88*	/	/	AD	Truncation	hemi	Female	Mitochondrial disorders	/	/	/	/	/	/	/	/	/	/	/	/	/	Supraventricular	/	/	/	/	/	/	/	/	/	/	/	/	/	/
AP2446	NDUFB11	27488349	F018	P018	/	05-II-1	Caucasian	Non-EA	/	c.276_278delCTT	p.Phe93del	/	/	AD	M	homozygo	Male	Sideroblastic anemia	/	/	/	/	/	/	/	/	/	/	/	/	/	/	/	/	/	/	/	/	/	/	/	/	/	/	/	/
AP2447	NDUFB11	27488349	F019	P019	/	400-II-2	Caucasian	Non-EA	/	c.276_278delCTT	p.Phe93del	/	/	AD	M	hemi	Female	Sideroblastic anemia	/	/	/	/	/	/	/	/	/	/	/	/	/	/	/	/	/	/	/	/	/	/	/	/	/	/	/	/
AP2448	NDUFB11	27488349	F020	P020	/	400-III-2	Caucasian	Non-EA	/	c.276_278delCTT	p.Phe93del	/	/	AD	M	homozygo	Male	Sideroblastic anemia	/	/	/	/	/	/	/	/	/	/	/	Myopathy HP:00(	/	/	/	/	/	/	/	/	/	/	/	/	/	/	/	/
AP2449	NDUFB11	27488349	F021	P021	/	400-III-3	Caucasian	Non-EA	/	c.276_278delCTT	p.Phe93del	/	/	AD	M	hemi	Female	Sideroblastic anemia	/	/	/	/	/	/	/	/	/	/	/	/	/	/	/	/	/	/	/	/	/	/	/	/	/	/	/	/
AP2450	NDUFB11	27488349	F022	P022	/	M-II-1	Caucasian	Non-EA	/	c.276_278delCTT	p.Phe93del	/	/	AD	M	homozygo	Male	Sideroblastic anemia	/	/	/	Optic atrophy HP	/	/	/	/	/	/	/	/	/	/	/	/	/	/	/	/	/	/	/	/	/	/	/	/
AP2451	NDUFB11	27488349	F023	P023	/	M-II-2	Caucasian	Non-EA	/	c.276_278delCTT	p.Phe93del	/	/	AD	M	homozygo	Male	Sideroblastic anemia	/	/	/	Optic atrophy HP	/	/	/	/	/	/	/	/	/	/	/	/	/	/	/	/	/	/	/	/	/	/	/	/
AP2452	NDUFB11	27488349	F024	P024	/	D-I-1	Caucasian	Non-EA	/	c.276_278delCTT	p.Phe93del	/	/	AD	M	homozygo	Male	Sideroblastic anemia	/	/	/	/	/	/	/	/	/	/	/	Myopathy HP:00(	/	/	/	/	/	/	/	/	/	/	/	/	/	/	/	/
AP2453	NDUFB11	27102574	F025	P025	/	/	Caucasian	Non-EA	/	c.276_278delCTT	p.Phe93del	/	/	AD	M	homozygo	Male	Complex I deficiency	/	/	/	/	/	/	/	/	/	/	/	/	/	Fetal tachycardia	/	/	/	/	/	/	/	/	/	/	/	/	/	/
AP2454	NDUFB11	26741492	F026	P026	/	Pt067	Caucasian	Non-EA	/	c.361G>A	p.E121K	/	/	AD	M	homozygo	Male	Mitochondrial disorders	/	/	/	/	/	/	/	/	/	/	/	/	/	/	/	/	/	/	/	/	/	/	/	/	/	/	/	/
AP2455	NDUFB11	25921236	F027	P027	S	/	Caucasian	Non-EA	/	c.324T>G	p.Tyr108*	/	/	AD	Truncation	hemi	Female	Cardiomyopathy	/	/	/	/	/	/	/	/	/	/	/	/	/	Cardiomyopathy	/	/	/	/	/	/	/	/	/	/	/	/	/	/
AP2456	NDUFB11	25921236	F028	P028	/	/	Caucasian	Non-EA	/	c.254G>A	p.Trp85*	/	/	AD	Truncation	hemi	Female	Cardiomyopathy	/	/	/	/	/	/	/	/	/	/	/	/	/	Cardiomyopathy	/	/	/	/	/	/	/	/	/	/	/	/	/	/
AP2457	NDUFB11	25772934	F029	P029	/	Subject 1	Caucasian	Non-EA	/	c.262C>T	p.Arg88*	/	/	AD	Truncation	hemi	Female	Complex I deficiency	/	/	/	/	/	/	/	/	/	/	/	Hypotonia HP:00	/	Cardiomyopathy	/	/	/	/	/	/	/	/	/	/	/	/	/	/
AP2458	NDUFB11	25772934	F030	P030	/	Subject 2	Caucasian	Non-EA	/	c.402delG	p.Arg134Serfs*3	/	/	AD	Truncation	hemi	Female	Complex I deficiency	/	/	Neurodevelopme	/	/	/	Nystagmus HP:0(	/	/	/	/	Hypotonia HP:00	/	Cardiomyopathy	/	/	/	/	/	/	/	/	/	/	/	/	/	/
AP2459	NDUFS1	21458341	F001	P001	/	patinet 1	Caucasian	Non-EA	NM_005006	c.497G>A	p.Gly166Glu	c.683T>C	p.Val228Ala	AR	M+M	compound I	Female	Mitochondrial complex I deficiency	0.42	0.75	Neurodevelopme	Optic atrophy HP	/	/	/	/	/	/	/	/	/	/	/	/	/	/	/	3.83	NO	/	/	/	/	/	/	/
AP2460	NDUFS1	21458341	F002	P002	/	patient 2	Caucasian	Non-EA	NM_005006	c.683T>C	p.Val228Ala	c.755A>G	p.Asp252Gly	AR	M+M	compound I	Male	Mitochondrial complex I deficiency	/	0.50	Neurodevelopme	/	/	/	/	/	/	/	/	Hypotonia HP:00	/	/	/	/	/	/	/	1.58	NO	/	/	/	/	/	/	/
AP2461	NDUFS1	21458341	F003	P003	/	patinet 3	Caucasian	Non-EA	NM_005006	c.1291C>G	p.Leu431Val	/	/	AD	M	heter	Male	Mitochondrial complex I deficiency	/	0.10	Neurodevelopme	/	Rod-cone dystrop	/	/	/	/	/	/	Hypotonia HP:00	/	Cardiomyopathy	/	/	/	/	/	/	/	/	/	/	/	/	/	/
AP2462	NDUFS1	21203893	F004	P004	/	patient 1	Caucasian	Non-EA	NM_005006	c.1783A>G	p.Thr595Ala	c.1783A>G	p.Thr595Ala	AR	M+M	homo	Female	Mitochondrial complex I deficiency	0.40	1.00	Neurodevelopme	/	/	/	Nystagmus HP:0(	/	/	/	/	Dystonia HP:000	/	/	/	/	/	/	/	4.00	yes	/	/	/	/	/	/	/
AP2463	NDUFS1	21203893	F004	P005	/	patient 2	Caucasian	Non-EA	NM_005006	c.1783A>G	p.Thr595Ala	c.1783A>G	p.Thr595Ala	AR	M+M	homo	Male	Mitochondrial complex I deficiency	/	0.67	Neurodevelopme	/	/	/	/	/	/	/	/	Hypotonia HP:00	/	/	/	/	/	/	/	2.50	yes	/	/	/	/	/	/	/
AP2464	NDUFS1	20382551	F005	P006	/	patinet 1	Caucasian	Non-EA	NM_005006	c.1669C>T	p.Arg557*	c.1855G>A	p.Asp619Asn	AR	M+M	compound I	Female	Mitochondrial complex I deficiency	/	0.67	Neurodevelopme	/	/	/	/	/	/	/	/	/	/	/	/	/	/	/	/	12.00	NO	/	/	/	/	/	/	/
AP2465	NDUFS1	20382551	F006	P007	/	patient 2	Caucasian	Non-EA	NM_005006	c.1222C>T	p.Arg408Cys	c.1222C>T	p.Arg408Cys	AR	M+M	homo	Male	Mitochondrial complex I deficiency	/	0.00	Neurodevelopme	/	/	/	/	/	/	/	/	Hypotonia HP:00	/	/	/	/	/	/	/	0.67	NO	/	/	/	/	/	/	/
AP2466	NDUFS1	20382551	F007	P008	/	patinet 3	Caucasian	Non-EA	NM_005006	c.631_622delGAA	p.211delGlu	c.683T>C	p.Val228Ala	AR	M+T	compound I	Female	Mitochondrial complex I deficiency	0.42	1.00	Neurodevelopme	/	/	/	Nystagmus HP:0(	/	/	/	/	/	/	/	/	/	/	/	/	2.00	NO	/	/	/	/	/	/	/
AP2467	NDUFS1	19167255	F008	P009	/	patinet 3	Caucasian	Non-EA	NM_005006	c.683T>C	p.Val228Ala	c.755A>G	p.Asp252Gly	AR	M+M	compound I	/	leukoencephalopathy	/	/	leukoencephalop	/	/	/	/	/	/	/	/	/	/	/	/	/	/	/	/	/	/	/	/	/	/	/	/	/
AP2468	NDUFS1	15824269	F009	P010	/		Spanish	Non-EA	NM_005006	c.691C>G	p.Leu231Val	c.691C>G	p.Leu231Val	AR	M+M	homo	/	Mitochondrial Complex I Deficienc	0.71	0.71	Brain magnetic re	/	/	/	Nystagmus HP:0(	/	/	/	/	Hypotonia HP:00	/	/	/	/	/	/	/	1.17	NO	/	/	/	/	/	/	/
AP2469	NDUFS1	11349233	F010	P011	/	patient 4	Caucasian	Non-EA	NM_005006	c.664_666del	p.Ile223del	c.755A>G	p.Asp252Gly	AR	M+T	compound I	Male	Mitochondrial Complex I Deficienc	0.58	0.33	Neurodevelopme	Optic atrophy HP	/	/	Nystagmus HP:0(	/	/	/	/	Hypotonia HP:00	/	/	/	/	/	/	/	0.83	NO	/	/	/	/	/	/	/
AP2470	NDUFS1	11349233	F011	P012	/	patient 5	Caucasian	Non-EA	NM_005006	c.721C>T	p.Arg241Trp	c.1669C>T	p.Arg557*	AR	M+T	compound I	Female	Mitochondrial Complex I Deficienc	/	0.17	Neurodevelopme	/	/	/	/	/	/	/	Hearing impairme	Hypotonia HP:00	/	/	/	/	/	/	/	0.42	NO	/	/	/	/	/	/	/
AP2471	NDUFS1	11349233	F012	P013	/	patient6	Portuguese	Non-EA	NM_005006	c.2119A>G	p.M707V	c.2119A>G	p.Met707Val	AR	M+M	homo	Male	Mitochondrial Complex I Deficienc	/	0.00	Neurodevelopme	/	/	/	/	/	/	/	Hearing impairme	Hypotonia HP:00	/	/	/	/	/	/	/	0.42	NO	/	/	/	/	/	/	/
AP2472	NDUFS2	28031252	F001	P001	/	II2	French	Non-EA	NM_00116615	c.158A>G	p.Tyr53Cys	c.923A>G	p.Tyr308Cys	AR	M+M	compound I	Male	LHON-like optic neuropathy	6.00	/	/	LHON-like neuro	/	/	/	/	/	/	/	/	/	/	/	/	/	/	/	26.00	YES	26.00	0.10	0.10	Non-haemorrhag	/	/	/
AP2473	NDUFS2	28031252	F001	P002	/	II3	French	Non-EA	NM_00116615	c. 158A>G	p.Tyr53Cys	c.923A>G	p.Tyr308Cys	AR	M+M	compound I	Female	LHON-like optic neuropathy	22.00	/	/	LHON-like neuro	/	/	/	/	/	/	/	/	/	/	/	/	/	/	/	22.00	YES	22.00	0.25	0.71	Papillary pseudo	/	/	/
AP2474	NDUFS2	28031252	F001	P003	/	II4	French	Non-EA	NM_00116615	c. 158A>G	p.Tyr53Cys	c.923A>G	p.Tyr308Cys	AR	M+M	compound I	Male	LHON-like optic neuropathy	7.00	/	/	LHON-like neuro	/	/	/	/	/	/	/	/	/	/	/	/	/	/	/	20.00	YES	20.00	0.71	0.10	Non-haemorrhag	/	/	/
AP2475	NDUFS2	20819849	F002	P004	/	patinet 3	Caucasian	Non-EA	NM_00116615	c.875T>C	p.Met292Thr	c.353G>A	p.Arg118Gln	AR	M+M	compound I	/	Mitochondrial leukoencephalopat	/	0.27	Neurodevelopme	/	/	/	Nystagmus HP:0(	/	/	/	/	Hypotonia HP:00	/	/	/	/	/	/	/	1.83	NO	/	/	/	/	/	/	/
AP2476	NDUFS2	20819849	F003	P005	/	Patient 19	Caucasian	Non-EA	NM_00116615	c.875T>C	p.Met292Thr	c.1328T>A	p.Met443Lys	AR	M+M	compound I	/	Mitochondrial leukoencephalopat	/	6.00	Seizure HP:0001	/	/	/	/	/	/	/	/	Hypotonia HP:00	/	/	/	/	/	/	/	9.00	YES	/	/	/	/	/	/	/
AP2477	NDUFS2	20819849	F004	P006	/	Patient 22	Caucasian	Non-EA	NM_00116615	c.875T>C	p.Met292Thr	c.442G>A	p.Glu148Lys	AR	M	compound I	/	Mitochondrial leukoencephalopat	/	1.00	Neurodevelopme	/	/	/	/	/	/	/	/	Hypotonia HP:00	/	/	/	/	/	/	/	7.50	YES	/	/	/	/	/	/	/
AP2478	NDUFS2	20819849	F005	P007	/	Patient 27	Caucasian	Non-EA	NM_00116615	c.998G>A	p.Arg333Gln	c.413G>A	p.Arg138Gln	AR	M+M	compound I	/	Mitochondrial leukoencephalopat	/	0.00	Neurodevelopme	/	/	/	/	/	/	/	/	/	/	Hypertrophic carc	Respiratory arres	/	/	/	/	0.29	NO	/	/	/	/	/	/	/

[illegible]

**AP2892**	***OPA1***	20157015	F234	P315	CN-1	35.00	Caucasian	Non-EA	NM_015560.1	c.1334G>A	p.Arg445His	/	/	AD	M	heter	Female	Dominant optic atrophy	/	/	/	Optic atrophy HP	/	/	/	/	/	/	/	/	/	/	/	/	/	/	/	46.00	YES	/	/	/	/	/	/	/
**AP2893**	***OPA1***	20157015	F234	P316	CN-1	36.00	Caucasian	Non-EA	NM_015560.1	c.1334G>A	p.Arg445His	/	/	AD	M	heter	Female	Dominant optic atrophy	/	/	/	Optic atrophy HP	/	/	/	/	/	/	/	/	/	/	/	/	/	/	/	22.00	YES	/	/	/	/	/	/	/
**AP2905**	***OPA1***	20157015	F239	P328	ES-1	49.00	Caucasian	Non-EA	NM_015560.1	c.1334G>A	p.Arg445His		/	AD	M	heter	Female	Dominant optic atrophy	/	/	/	Optic atrophy HP	/	/	Progressive exte	/	/	/	/	/	/	/	/	/	/	/	/	30.00	YES	/	/	/	/	/	/	/
**AP2906**	***OPA1***	20157015	F239	P329	ES-1	48.00	Caucasian	Non-EA	NM_015560.1	c.1334G>A	p.Arg445His		/	AD	M	heter	Female	Dominant optic atrophy	/	/	/	Optic atrophy HP	/	/	/	/	/	/	/	Myopathy HP:00	/	/	/	/	/	/	/	57.00	YES	/	/	/	DPOD	/	/	/
**AP2909**	***OPA1***	20157015	F242	P332	FR-2	52.00	Caucasian	Non-EA	NM_015560.1	c.1334G>A	p.Arg445His		/	AD	M	heter	Female	Dominant optic atrophy plus	/	/	Ataxia HP:00012	Optic atrophy HP	/	/	/	/	/	/	/	Myopathy HP:00	/	/	/	/	/	/	/	35.00	YES	/	/	/	/	/	/	/
**AP2910**	***OPA1***	20157015	F243	P333	FR-3	53.00	Caucasian	Non-EA	NM_015560.1	c.1334G>A	p.Arg445His		/	AD	M	heter	Male	Dominant optic atrophy	/	/	/	Optic atrophy HP	/	/	/	/	/	/	/	/	/	/	/	/	/	/	/	14.00	YES	/	/	/	/	/	/	/
**AP2913**	***OPA1***	20157015	F246	P336	FR-6	56.00	Caucasian	Non-EA	NM_015560.1	c.1334G>A	p.Arg445His		/	AD	M	heter	Female	Dominant optic atrophy	/	/	/	Optic atrophy HP	/	/	/	/	/	/	/	/	/	/	/	/	/	/	/	12.00	YES	/	/	/	/	/	/	/
**AP2914**	***OPA1***	20157015	F247	P337	FR-7	57.00	Caucasian	Non-EA	NM_015560.1	c.1334G>A	p.Arg445His		/	AD	M	heter	Male	Dominant optic atrophy	/	/	/	Optic atrophy HP	/	/	/	/	/	/	/	/	/	/	/	/	/	/	/	20.00	YES	/	/	/	/	/	/	/
**AP2915**	***OPA1***	20157015	F248	P338	FR-8	58.00	Caucasian	Non-EA	NM_015560.1	c.1334G>A	p.Arg445His		/	AD	M	heter	Female	Dominant optic atrophy plus	/	/	Ataxia HP:00012	Optic atrophy HP	/	/	/	/	/	/	/	/	/	/	/	/	/	/	/	38.00	YES	/	/	/	/	/	/	/
**AP2943**	***OPA1***	20157015	F261	P366	US-1	89.00	Caucasian	Non-EA	NM_015560.1	c.1334G>A	p.Arg445His		/	AD	M	heter	Female	Dominant optic atrophy	/	/	/	Optic atrophy HP	/	/	Progressive exte	/	/	/	/	/	/	/	/	/	/	/	/	64.00	YES	/	/	/	/	/	/	/
**AP2944**	***OPA1***	20157015	F261	P367	US-1	90.00	Caucasian	Non-EA	NM_015560.1	c.1334G>A	p.Arg445His		/	AD	M	heter	Male	Dominant optic atrophy	/	/	/	Optic atrophy HP	/	/	Progressive exte	/	/	/	/	/	/	/	/	/	/	/	/	56.00	YES	/	/	/	/	/	/	/
**AP2945**	***OPA1***	20157015	F261	P368	US-1	91.00	Caucasian	Non-EA	NM_015560.1	c.1334G>A	p.Arg445His		/	AD	M	heter	Female	Dominant optic atrophy	/	/	/	Optic atrophy HP	/	/	Progressive exte	/	/	/	/	/	/	/	/	/	/	/	/	77.00	YES	/	/	/	/	/	/	/
**AP2946**	***OPA1***	20157015	F261	P369	US-1	92.00	Caucasian	Non-EA	NM_015560.1	c.1334G>A	p.Arg445His		/	AD	M	heter	Male	Dominant optic atrophy	/	/	/	Optic atrophy HP	/	/	Progressive exte	/	/	/	/	/	/	/	/	/	/	/	/	64.00	YES	/	/	/	/	/	/	/
**AP2947**	***OPA1***	20157015	F261	P370	US-1	93.00	Caucasian	Non-EA	NM_015560.1	c.1334G>A	p.Arg445His		/	AD	M	heter	Male	Dominant optic atrophy	/	/	/	Optic atrophy HP	/	/	Progressive exte	/	/	/	/	/	/	/	/	/	/	/	/	49.00	YES	/	/	/	/	/	/	/
**AP2948**	***OPA1***	20157015	F261	P371	US-1	94.00	Caucasian	Non-EA	NM_015560.1	c.1334G>A	p.Arg445His		/	AD	M	heter	Female	Dominant optic atrophy	/	/	/	Optic atrophy HP	/	/	Progressive exte	/	/	/	/	/	/	/	/	/	/	/	/	45.00	YES	/	/	/	/	/	/	/
**AP2949**	***OPA1***	20157015	F261	P372	US-1	95.00	Caucasian	Non-EA	NM_015560.1	c.1334G>A	p.Arg445His		/	AD	M	heter	Male	Dominant optic atrophy	/	/	/	Optic atrophy HP	/	/	Progressive exte	/	/	/	/	/	/	/	/	/	/	/	/	38.00	YES	/	/	/	/	/	/	/
**AP2950**	***OPA1***	20157015	F261	P373	US-1	96.00	Caucasian	Non-EA	NM_015560.1	c.1334G>A	p.Arg445His		/	AD	M	heter	Male	Dominant optic atrophy	/	/	/	Optic atrophy HP	/	/	Progressive exte	/	/	/	/	/	/	/	/	/	/	/	/	42.00	YES	/	/	/	/	/	/	/
**AP2951**	***OPA1***	20157015	F261	P374	US-1	97.00	Caucasian	Non-EA	NM_015560.1	c.1334G>A	p.Arg445His		/	AD	M	heter	Female	Dominant optic atrophy	/	/	/	Optic atrophy HP	/	/	Progressive exte	/	/	/	/	/	/	/	/	/	/	/	/	43.00	YES	/	/	/	/	/	/	/
**AP2952**	***OPA1***	20157015	F261	P375	US-1	98.00	Caucasian	Non-EA	NM_015560.1	c.1334G>A	p.Arg445His		/	AD	M	heter	Female	Dominant optic atrophy	/	/	/	Optic atrophy HP	/	/	Progressive exte	/	/	/	/	/	/	/	/	/	/	/	/	51.00	YES	/	/	/	/	/	/	/
**AP2953**	***OPA1***	20157015	F261	P376	US-1	99.00	Caucasian	Non-EA	NM_015560.1	c.1334G>A	p.Arg445His		/	AD	M	heter	Female	Dominant optic atrophy	/	/	/	Optic atrophy HP	/	/	Progressive exte	/	/	/	/	/	/	/	/	/	/	/	/	26.00	YES	/	/	/	/	/	/	/
**AP2954**	***OPA1***	20157015	F261	P377	US-1	100.00	Caucasian	Non-EA	NM_015560.1	c.1334G>A	p.Arg445His		/	AD	M	heter	Female	Dominant optic atrophy	/	/	/	Optic atrophy HP	/	/	/	/	/	/	/	/	/	/	/	/	/	/	/	19.00	YES	/	/	/	/	/	/	/
**AP2955**	***OPA1***	20157015	F261	P378	US-1	87.00	Caucasian	Non-EA	NM_015560.1	c.1334G>A	p.Arg445His		/	AD	M	heter	Female	Dominant optic atrophy	/	/	/	Optic atrophy HP	/	/	/	/	/	/	/	/	/	/	/	/	/	/	/	71.00	YES	/	/	/	/	/	/	/
**AP2956**	***OPA1***	20157015	F261	P379	US-1	88.00	Caucasian	Non-EA	NM_015560.1	c.1334G>A	p.Arg445His		/	AD	M	heter	Female	Dominant optic atrophy	/	/	/	Optic atrophy HP	/	/	Progressive exte	/	/	/	/	/	/	/	/	/	/	/	/	68.00	YES	/	/	/	/	/	/	/
**AP3021**	***OPA1***	22197506	F323	P444	Family 2	AUNT	Italian	Non-EA	NM_015560.1	c.985-2A>G	p.?	/	/	AD	truncation	heter	Male	Dominant optic atrophy	6	/	/	Optic atrophy HP	/	/	/	/	/	/	/	/	/	/	/	/	/	/	/	15	YES	41	0.20	0.2	/	/	/	/
**AP3022**	***OPA1***	22382025	F324	P445	I-2	/	Chinese	EA	NM_015560.1	c.1198C>G	p.Pro400Ala	/	/	AD	M	heter	Female	Dominant optic atrophy	20	/	/	Optic atrophy HP	/	/	/	/	/	/	/	/	/	/	/	/	/	/	/	74	YES	74	0.01	0.01	/	/	/	/
**AP3024**	***OPA1***	22382025	F324	P447	II-6	/	Chinese	EA	NM_015560.1	c.1198C>G	p.Pro400Ala	/	/	AD	M	heter	Female	Dominant optic atrophy	8	/	/	Optic atrophy HP	/	/	/	/	/	/	/	/	/	/	/	/	/	/	/	45	YES	45	0.02	0.03	/	/	/	/
**AP3025**	***OPA1***	22382025	F324	P448	II-9	/	Chinese	EA	NM_015560.1	c.1198C>G	p.Pro400Ala	/	/	AD	M	heter	Male	Dominant optic atrophy	12	/	/	Optic atrophy HP	/	/	/	/	/	/	/	/	/	/	/	/	/	/	/	41	YES	41	0.04	0.04	/	/	/	/
**AP3026**	***OPA1***	22382025	F324	P449	II-11	/	Chinese	EA	NM_015560.1	c.1198C>G	p.Pro400Ala	/	/	AD	M	heter	Male	Dominant optic atrophy	18	/	/	Optic atrophy HP	/	/	/	/	/	Color vision defe	/	/	/	/	/	/	/	/	/	39	YES	39	0.04	0.04	/	/	/	/
**AP3027**	***OPA1***	22382025	F324	P450	II-15	/	Chinese	EA	NM_015560.1	c.1198C>G	p.Pro400Ala	/	/	AD	M	heter	Male	Dominant optic atrophy	5	/	/	Optic atrophy HP	/	/	/	/	/	/	/	/	/	/	/	/	/	/	/	34	YES	34	0.01	0.01	/	/	/	/
**AP3028**	***OPA1***	22382025	F324	P451	II-17	/	Chinese	EA	NM_015560.1	c.1198C>G	p.Pro400Ala	/	/	AD	M	heter	Male	Dominant optic atrophy	20	/	/	Optic atrophy HP	/	/	/	/	/	/	/	/	/	/	/	/	/	/	/	32	YES	32	0.12	0.06	/	/	/	/
**AP3056**	***OPA1***	23384603	F337	P479	Family XII	XII-1	Caucasian	Non-EA	NM_015560.1	c.1334G>A	p.Arg445His	/	/	AD	M	heter	Male	Dominant optic atrophy plus	13.00	/	Ataxia HP:00012	Optic atrophy HP	/	/	/	/	/	/	/	/	/	/	/	/	/	/	/	60.00	YES	60.00	0.05	0.05	/	/	/	/
**AP3029**	***OPA1***	22382025	F324	P452	III-10	/	Chinese	EA	NM_015560.1	c.1198C>G	p.Pro400Ala	/	/	AD	M	heter	Female	Dominant optic atrophy	10	/	/	Optic atrophy HP	/	/	/	/	/	/	/	/	/	/	/	/	/	/	/	26	YES	26	0.02	0.01	/	/	/	/
**AP3058**	***OPA1***	23384603	F337	P481	Family XII	XII-3	Caucasian	Non-EA	NM_015560.1	c.1334G>A	p.Arg445His	/	/	AD	M	heter	Female	Dominant optic atrophy plus	/	/	Ataxia HP:00012	Optic atrophy HP	/	/	/	/	/	/	/	/	/	/	/	/	/	/	/	29.00	YES	29.00	0.25	0.25	/	/	/	/
**AP3030**	***OPA1***	22382025	F324	P453	III-11	/	Chinese	EA	NM_015560.1	c.1198C>G	p.Pro400Ala	/	/	AD	M	heter	Female	Dominant optic atrophy	14	/	/	Optic atrophy HP	/	/	/	/	/	/	/	/	/	/	/	/	/	/	/	24	YES	24	0.10	0.10	/	/	/	/
**AP3044**	***OPA1***	23384603	F328	P467	Family III	III-1	Caucasian	Non-EA	NM_015560.1	c.1067_1068dup	p.Ala357Leufs*4	/	/	AD	truncation	heter	Male	Dominant optic atrophy	early adulthoo	/	/	Optic atrophy HP	/	/	/	/	/	/	/	/	/	/	/	/	/	/	/	39.00	YES	39.00	0.40	0.40	/	/	/	/
**AP3050**	***OPA1***	23384603	F332	P473	Family VII	VII-1	Caucasian	Non-EA	NM_015560.1	c.1334G>A	p.Arg445His	/	/	AD	M	heter	Female	Dominant optic atrophy	6.00	/	/	Optic atrophy HP	/	/	/	/	/	/	/	/	/	/	/	/	/	/	/	37.00	YES	37.00	0.10	0.10	/	/	/	/
**AP3051**	***OPA1***	23384603	F333	P474	Family VIII	VIII-1	Caucasian	Non-EA	NM_015560.1	c.1334G>A	p.Arg445His	/	/	AD	M	heter	Female	Dominant optic atrophy	13.00	/	/	Optic atrophy HP	/	/	/	/	/	/	/	/	/	/	/	/	/	/	/	57.00	YES	/	/	/	/	/	/	/
**AP3052**	***OPA1***	23384603	F333	P475	Family VIII	VIII-2	Caucasian	Non-EA	NM_015560.1	c.1334G>A	p.Arg445His	/	/	AD	M	heter	Female	Dominant optic atrophy	9.00	/	/	Optic atrophy HP	/	/	/	/	/	/	/	/	/	/	/	/	/	/	/	30.00	YES	/	/	/	/	/	/	/
**AP3053**	***OPA1***	23384603	F334	P476	Family IX	IX-1	Caucasian	Non-EA	NM_015560.1	c.1334G>A	p.Arg445His	/	/	AD	M	heter	Female	Dominant optic atrophy	7.00	/	/	Optic atrophy HP	/	/	/	/	/	/	/	/	/	/	/	/	/	/	/	12.00	YES	12.00	0.40	0.40	/	/	/	/
**AP3388**	***OPA1***	28926202	F629	P811	13	/	Caucasian	Non-EA	NM_015560.1	c.1334G>A	p.Arg445His	/	/	AD	M	heter	/	Dominant optic atrophy	/	/	/	Optic atrophy HP	/	/	/	/	/	/	/	/	/	/	/	/	/	/	/	/	/	/	/	/	/	/	/	/
**AP3405**	***OPA1***	29688962	F646	P828	/	/	Caucasian	Non-EA	NM_015560.1	c.1334G>A	p.Arg445His	/	/	AD	M	heter	/	Hearing impairment	/	/	/	/	/	/	/	/	/	/	Hearing impairme	/	/	/	/	/	/	/	/	/	/	/	/	/	/	/	/	/
[illegible]																																														

**AP2730**	***OPA1***	16513463	F121	P153	543	II-2	Japanese	EA	NM_015560.1	c.2708_2711del	p.Val903Glyfs*3		/	AD	truncation	heter	Female	Dominant optic atrophy	/	/	/	Optic atrophy HP:	/	/	/	/	/	Color vision defe	/	/	/	/	/	/	/	/	/	33.00	YES	/	0.3	0.3	/	/	/	/
**AP3458**	***OPA1***	32025183	F682	P881	OA08	II-1	Chinese	EA	NM_015560.1	c.1847+2T>G	p.?	/	/	AD	truncation	heter	Male	Dominant optic atrophy	5.00	/	/	Optic atrophy HP:	/	/	/	/	/	/	/	/	/	/	/	/	/	/	/	9.00	YES	9.00	0.50	0.4	/	/	/	/
**AP2755**	***OPA1***	17251483	F137	P178	42	3.00	Japanese	EA	NM_015560.1	c.2708_2711del	p.Val903Glyfs*3	/	/	AD	truncation	heter	Female	Dominant optic atrophy	/	/	/	Optic atrophy HP:	/	/	/	/	/	/	/	/	/	/	/	/	/	/	/	27.00	YES	27.00	0.40	0.40	/	/	/	/
**AP2770**	***OPA1***	17306754	F152	P193	VIC9	/	Caucasian	Non-EA	NM_015560.1	c.2708_2711del	p.Val903Glyfs*3		/	AD	truncation	heter	/	Dominant optic atrophy	/	/	/	Optic atrophy HP:	/	/	/	/	/	/	/	/	/	/	/	/	/	/	/	/	/	/	/	/	/	/	/	/
**AP2771**	***OPA1***	17306754	F153	P194	TAS4	/	Caucasian	Non-EA	NM_015560.1	c.2708_2711del	p.Val903Glyfs*3		/	AD	truncation	heter	/	Dominant optic atrophy	/	/	/	Optic atrophy HP:	/	/	/	/	/	/	/	/	/	/	/	/	/	/	/	/	/	/	/	/	/	/	/	/
**AP2772**	***OPA1***	17306754	F154	P195	NSW6	/	Caucasian	Non-EA	NM_015560.1	c.2708_2711del	p.Val903Glyfs*3		/	AD	truncation	heter	/	Dominant optic atrophy	/	/	/	Optic atrophy HP:	/	/	/	/	/	/	/	/	/	/	/	/	/	/	/	/	/	/	/	/	/	/	/	/
**AP2812**	***OPA1***	17722006	F194	P235	/	OAK52	Caucasian	Non-EA	NM_015560.1	c.2708_2711del	p.Val903Glyfs*3		/	AD	truncation	heter	/	Dominant optic atrophy	/	/	/	Optic atrophy HP:	/	/	/	/	/	/	/	/	/	/	/	/	/	/	/	/	/	/	/	/	/	/	/	/
**AP2813**	***OPA1***	17722006	F195	P236	/	OAK123	Caucasian	Non-EA	NM_015560.1	c.2708_2711del	p.Val903Glyfs*3		/	AD	truncation	heter	/	Dominant optic atrophy	/	/	/	Optic atrophy HP:	/	/	/	/	/	/	/	/	/	/	/	/	/	/	/	/	/	/	/	/	/	/	/	/
**AP2827**	***OPA1***	18653586	F202	P250	V9	/	Caucasian	Non-EA	NM_015560.1	c.2708delTTAG	p.Val903Glyfs*3		/	AD	truncation	heter	/	Dominant optic atrophy	/	/	/	Optic atrophy HP:	/	/	/	/	/	/	/	/	/	/	/	/	/	/	/	/	/	/	/	/	/	/	/	/
**AP2830**	***OPA1***	18653586	F205	P253	T4	/	Caucasian	Non-EA	NM_015560.1	c.2708delTTAG	p.Val903Glyfs*3		/	AD	truncation	heter	/	Dominant optic atrophy	/	/	/	Optic atrophy HP:	/	/	/	/	/	/	/	/	/	/	/	/	/	/	/	/	/	/	/	/	/	/	/	/
**AP2867**	***OPA1***	20157015	F223	P290	UK-7	10.00	British	Non-EA	NM_015560.1	c.2708_2711del	p.Val903Glyfs*3	/	/	AD	truncation	heter	Female	Dominant optic atrophy	/	/	/	Optic atrophy HP:	/	/	Progressive exte	/	/	/	/	/	/	/	/	/	/	/	/	46.00	YES	/	/	/	/	/	/	/
**AP3463**	***OPA1***	32025183	F686	P886	OA12	I-1	Chinese	EA	NM_015560.1	c.871-1G>C	p.?	/	/	AD	truncation	heter	Male	Dominant optic atrophy	8.00	/	/	Optic atrophy HP:	/	/	/	/	/	/	/	/	/	/	/	/	/	/	/	30.00	YES	30.00	0.30	0.3	/	/	/	/
**AP3464**	***OPA1***	32025183	F686	P887	OA12	II-1	Chinese	EA	NM_015560.1	c.871-1G>C	p.?	/	/	AD	truncation	heter	Male	Dominant optic atrophy	4.00	/	/	Optic atrophy HP:	/	/	/	/	/	/	/	/	/	/	/	/	/	/	/	6.00	YES	6.00	0.30	0.3	/	/	/	/
**AP3447**	***OPA1***	32025183	F671	P870	OA06	II-1	Chinese	EA	NM_015560.1	c.869G>A	p.Arg290Gln	/	/	AD	M	heter	Female	Dominant optic atrophy	11.00	/	/	Optic atrophy HP:	/	/	/	/	/	/	/	/	/	/	/	/	/	/	/	14.00	YES	14.00	0.20	0.1	/	/	/	/
**AP3455**	***OPA1***	32025183	F679	P878	OA06	I-1	Chinese	EA	NM_015560.1	c.869G>A	p.Arg290Gln	/	/	AD	M	heter	Female	Dominant optic atrophy	12.00	/	/	Optic atrophy HP:	/	/	/	/	/	/	/	/	/	/	/	/	/	/	/	42.00	YES	42.00	0.50	0.4	/	/	/	/
**AP3461**	***OPA1***	32025183	F685	P884	OA11	II-1	Chinese	EA	NM_015560.1	c.112C>T	p.Arg38*	/	/	AD	M	heter	Female	Dominant optic atrophy	12.00	/	/	Optic atrophy HP:	/	/	/	/	/	/	/	/	/	/	/	/	/	/	/	43.00	YES	43.00	0.10	0.1	/	/	/	/
**AP3462**	***OPA1***	32025183	F685	P885	OA11	II-2	Chinese	EA	NM_015560.1	c.112C>T	p.Arg38*	/	/	AD	M	heter	Female	Dominant optic atrophy	10.00	/	/	Optic atrophy HP:	/	/	/	/	/	/	/	/	/	/	/	/	/	/	/	42.00	YES	42.00	0.10	0.1	/	/	/	/
**AP3067**	***OPA1***	23388408	F340	P490	2	/	Caucasian	Non-EA	NM_015560.1	c.2708_2711del	p.Val903Glyfs*3	/	/	AD	truncation	heter	Male	Dominant optic atrophy	/	/	/	Optic atrophy HP:	/	/	/	/	/	Color vision defe	/	/	/	/	/	/	/	/	/	/	/	/	/	/	/	/	/	/
**AP3070**	***OPA1***	23388408	F343	P493	5	/	Caucasian	Non-EA	NM_015560.1	c.2708_2711del	p.Val903Glyfs*3	/	/	AD	truncation	heter	Female	Dominant optic atrophy	/	/	/	Optic atrophy HP:	/	/	/	/	/	Color vision defe	/	/	/	/	/	/	/	/	/	/	/	/	/	/	/	/	/	/
**AP3071**	***OPA1***	23388408	F344	P494	6	/	Caucasian	Non-EA	NM_015560.1	c.2708_2711del	p.Val903Glyfs*3	/	/	AD	truncation	heter	Male	Dominant optic atrophy	/	/	/	Optic atrophy HP:	/	/	/	/	/	Color vision defe	/	/	/	/	/	/	/	/	/	/	/	/	/	/	/	/	/	/
**AP3072**	***OPA1***	23388408	F345	P495	7	/	Caucasian	Non-EA	NM_015560.1	c.2708_2711del	p.Val903Glyfs*3	/	/	AD	truncation	heter	Female	Dominant optic atrophy	/	/	/	Optic atrophy HP:	/	/	/	/	/	Color vision defe	/	/	/	/	/	/	/	/	/	/	/	/	/	/	/	/	/	/
**AP3452**	***OPA1***	32025183	F676	P875	OA03	II-1	Chinese	EA	NM_015560.1	c.1334G>A	p.Arg445His	/	/	AD	M	heter	Male	Dominant optic atrophy	3.00	/	/	Optic atrophy HP:	/	/	/	/	/	/	/	/	/	/	/	/	/	/	/	8.00	YES	8.00	0.20	0.3	/	/	/	/
**AP3460**	***OPA1***	32025183	F684	P883	OA10	II-2	Chinese	EA	NM_015560.1	c.1334G>A	p.Arg445His	/	/	AD	M	heter	Male	Dominant optic atrophy	7.00	/	/	Optic atrophy HP:	/	/	/	/	/	/	/	/	/	/	/	/	/	/	/	9.00	YES	9.00	0.20	0.2	/	/	/	/
**AP3465**	***OPA1***	32025183	F687	P888	OA13	I-2	Chinese	EA	NM_015560.1	c.1961A>G	p.Asp654Gly	/	/	AD	M	heter	Female	Dominant optic atrophy	6.00	/	/	Optic atrophy HP:	/	/	/	/	/	/	/	/	/	/	/	/	/	/	/	31.00	YES	31.00	0.40	0.4	/	/	/	/
**AP3089**	***OPA1***	23401657	F362	P512	Ie2062M	/	Chinese	EA	NM_015560.1	c.2708_2711del	p.Val903Glyfs*3	/	/	AD	truncation	heter	/	Dominant optic atrophy	/	/	/	Optic atrophy HP:	/	/	/	/	/	/	/	/	/	/	/	/	/	/	/	49.00	YES	49.00	0.70	0.70	/	/	/	/
**AP3095**	***OPA1***	24024178	F368	P518	6	/	Caucasian	Non-EA	NM_015560.1	c.2708_2711del	p.Val903Glyfs*3	/	/	AD	truncation	heter	Male	Dominant optic atrophy	/	/	/	Optic atrophy HP:	/	/	/	/	/	/	/	/	/	/	/	/	/	/	/	43.00	YES	43.00	0.16	0.20	DPOD	/	/	/
**AP3115**	***OPA1***	24907432	F385	P538	Family1	/	Caucasian	Non-EA	NM_015560.1	c.2708_2711del	p.Val903Glyfs*3	/	/	AD	truncation	heter	/	Dominant optic atrophy	/	/	/	Optic atrophy HP:	/	/	/	/	/	/	/	/	/	/	/	/	/	/	/	/	/	/	/	/	/	/	/	/
**AP3116**	***OPA1***	24907432	F386	P539	Family2	/	Caucasian	Non-EA	NM_015560.1	c.2708_2711del	p.Val903Glyfs*3	/	/	AD	truncation	heter	/	Dominant optic atrophy	/	/	/	Optic atrophy HP:	/	/	/	/	/	/	/	/	/	/	/	/	/	/	/	/	/	/	/	/	/	/	/	/
**AP3117**	***OPA1***	24907432	F387	P540	Family3	/	Caucasian	Non-EA	NM_015560.1	c.2708_2711del	p.Val903Glyfs*3	/	/	AD	truncation	heter	/	Dominant optic atrophy	/	/	/	Optic atrophy HP:	/	/	/	/	/	/	/	/	/	/	/	/	/	/	/	/	/	/	/	/	/	/	/	/
**AP3118**	***OPA1***	24907432	F388	P541	Family4	/	Caucasian	Non-EA	NM_015560.1	c.2708_2711del	p.Val903Glyfs*3	/	/	AD	truncation	heter	/	Dominant optic atrophy	/	/	/	Optic atrophy HP:	/	/	/	/	/	/	/	/	/	/	/	/	/	/	/	/	/	/	/	/	/	/	/	/
**AP3119**	***OPA1***	24907432	F389	P542	Family5	/	Caucasian	Non-EA	NM_015560.1	c.2708_2711del	p.Val903Glyfs*3	/	/	AD	truncation	heter	/	Dominant optic atrophy	/	/	/	Optic atrophy HP:	/	/	/	/	/	/	/	/	/	/	/	/	/	/	/	/	/	/	/	/	/	/	/	/
**AP3189**	***OPA1***	25205859	F457	P612	/	A684	Chinese	EA	NM_015560.1	c.2708_2711del	p.Val903Glyfs*3	/		AD	truncation	heter	Female	Dominant optic atrophy	/	/	/	Optic atrophy HP:	/	/	/	/	/	/	/	/	/	/	/	/	/	/	/	16	YES	16	0.08	0.10	/	/	/	/
**AP3190**	***OPA1***	25205859	F458	P613	/	A690	Chinese	EA	NM_015560.1	c.2708_2711del	p.Val903Glyfs*3	/		AD	truncation	heter	Male	Dominant optic atrophy	/	/	/	Optic atrophy HP:	/	/	/	/	/	/	/	/	/	/	/	/	/	/	/	20	YES	20	0.25	0.25	/	/	/	/
**AP3191**	***OPA1***	25205859	F459	P614	/	076014	Chinese	EA	NM_015560.1	c.2708_2711del	p.Val903Glyfs*3	/		AD	truncation	heter	Male	Dominant optic atrophy	/	/	/	Optic atrophy HP:	/	/	/	/	/	/	/	/	/	/	/	/	/	/	/	17	YES	17	0.60	0.30	/	/	/	/
**AP3466**	***OPA1***	32025183	F687	P889	OA13	II-1	Chinese	EA	NM_015560.1	c.1961A>G	p.Asp654Gly	/	/	AD	M	heter	Male	Dominant optic atrophy	3.00	/	/	Optic atrophy HP:	/	/	/	/	/	/	/	/	/	/	/	/	/	/	/	7.00	YES	7.00	0.30	0.3	/	/	/	/
**AP3451**	***OPA1***	32025183	F675	P874	OA02	II-2	Chinese	EA	NM_015560.1	c.193C>G	p.Leu65Val	/	/	AD	M	heter	Male	Dominant optic atrophy	0.00	/	/	Optic atrophy HP:	/	/	/	/	/	/	/	/	/	/	/	/	/	/	/	30.00	YES	30.00	0.25	0.02	/	/	/	/
**AP3467**	***OPA1***	32025183	F688	P890	OA14	I-2	Chinese	EA	NM_015560.1	c.639_640del	p.Lys214Asnfs*2	/		AD	truncation	heter	Female	Dominant optic atrophy	6.00	/	/	Optic atrophy HP:	/	/	/	/	/	/	/	/	/	/	/	/	/	/	/	28.00	YES	28.00	0.30	0.4	/	/	/	/
**AP3468**	***OPA1***	32025183	F688	P891	OA14	II-1	Chinese	EA	NM_015560.1	c.639_640del	p.Lys214Asnfs*2	/		AD	truncation	heter	Male	Dominant optic atrophy	3.00	/	/	Optic atrophy HP:	/	/	/	/	/	/	/	/	/	/	/	/	/	/	/	6.00	YES	6.00	0.25	0.3	/	/	/	/
**AP3456**	***OPA1***	32025183	F680	P879	OA07	II-2	Chinese	EA	NM_015560.1	c.822_823del	p.Ser276*	/		AD	truncation	heter	Male	Dominant optic atrophy	4.00	/	/	Optic atrophy HP:	/	/	/	/	/	/	/	/	/	/	/	/	/	/	/	9.00	YES	9.00	0.40	0.3	/	/	/	/
**AP3454**	***OPA1***	32025183	F678	P877	OA05	II-1	Chinese	EA	NM_015560.1	c.906dupT	p.Ser303*	/	/	AD	M	heter	Male	Dominant optic atrophy	6.00	/	/	Optic atrophy HP:	/	/	/	/	/	/	/	/	/	/	/	/	/	/	/	10.00	YES	10.00	0.40	0.5	/	/	/	/
**AP3453**	***OPA1***	32025183	F677	P876	OA04	II-2	Chinese	EA	NM_015560.1	c.1635C>A	p.Ser545Arg	/	/	AD	M	heter	Male	Dominant optic atrophy	6.00	/	/	Optic atrophy HP:	/	/	/	/	/	/	/	/	/	/	/	/	/	/	/	10.00	YES	10.00	0.50	0.5	/	/	/	/
**AP3311**	***OPA1***	27860320	F560	P734	/	39	Caucasian	Non-EA	NM_015560.1	c.2708_2711del	p.Val903Glyfs*3	/	/	AD	truncation	heter	/	Dominant optic atrophy	/	/	/	Optic atrophy HP:	/	/	/	/	/	/	/	/	/	/	/	/	/	/	/	/	/	/	/	/	/	/	/	/
**AP3312**	***OPA1***	27860320	F561	P735	/	40	Caucasian	Non-EA	NM_015560.1	c.2708_2711del	p.Val903Glyfs*3	/	/	AD	truncation	heter	/	Dominant optic atrophy	/	/	/	Optic atrophy HP:	/	/	/	/	/	/	/	/	/	/	/	/	/	/	/	/	/	/	/	/	/	/	/	/
**AP3313**	***OPA1***	27860320	F562	P736	/	41	Caucasian	Non-EA	NM_015560.1	c.2708_2711del	p.Val903Glyfs*3	/	/	AD	truncation	heter	/	Dominant optic atrophy	/	/	/	Optic atrophy HP:	/	/	/	/	/	/	/	/	/	/	/	/	/	/	/	/	/	/	/	/	/	/	/	/
**AP3314**	***OPA1***	27860320	F563	P737	/	42	Caucasian	Non-EA	NM_015560.1	c.2708_2711del	p.Val903Glyfs*3	/	/	AD	truncation	heter	/	Dominant optic atrophy	/	/	/	Optic atrophy HP:	/	/	/	/	/	/	/	/	/	/	/	/	/	/	/	/	/	/	/	/	/	/	/	/
**AP3315**	***OPA1***	27860320	F564	P738	/	43	Caucasian	Non-EA	NM_015560.1	c.2708_2711del	p.Val903Glyfs*3	/	/	AD	truncation	heter	/	Dominant optic atrophy	/	/	/	Optic atrophy HP:	/	/	/	/	/	/	/	/	/	/	/	/	/	/	/	/	/	/	/	/	/	/	/	/
**AP3332**	***OPA1***	27974645	F581	P755	Family 1	/	Caucasian	Non-EA	NM_015560.1	c.2708_2711del	p.Val903Glyfs*3	c.661G>A	p.Asp221Asn	AR	M+T	compound h	Male	Dominant optic atrophy	2.00	Not mention	Ataxia HP:00012	Optic atrophy HP:	/	Ptosis HP:00005	/	/	/	/	Hearing impairm	/	/	/	/	/	/	/	/	17.00	YES	17.00	CF	HM	/	DT	DT	/
**AP3333**	***OPA1***	27974645	F581	P756	Family 1	/	Caucasian	Non-EA	NM_015560.1	c.2708_2711del	p.Val903Glyfs*3	/	/	AD	truncation	heter	Male	Dominant optic atrophy	/	/	/	Optic atrophy HP:	/	/	/	/	/	/	/	/	/	/	/	/	/	/	/	67.00	YES				/	/	/	/
**AP3376**	***OPA1***	28926202	F617	P799	1	/	Caucasian	Non-EA	NM_015560.1	c.2708_2711del	p.Val903Glyfs*3		/	AD	M	heter	/	Dominant optic atrophy	/	/	/	Optic atrophy HP:	/	/	/	/	/	/	/	/	/	/	/	/	/	/	/	/	/	/	/	/	/	/	/	/
**AP3377**	***OPA1***	28926202	F618	P800	2	/	Caucasian	Non-EA	NM_015560.1	c.2708_2711del	p.Val903Glyfs*3		/	AD	truncation	heter	/	Dominant optic atrophy	/	/	/	Optic atrophy HP:	/	/	/	/	/	/	/	/	/	/	/	/	/	/	/	/	/	/	/	/	/	/	/	/
**AP3378**	***OPA1***	28926202	F619	P801	3	/	Caucasian	Non-EA	NM_015560.1	c.2708_2711del	p.Val903Glyfs*3		/	AD	truncation	heter	/	Dominant optic atrophy	/	/	/	Optic atrophy HP:	/	/	/	/	/	/	/	/	/	/	/	/	/	/	/	/	/	/	/	/	/	/	/	/
**AP3400**	***OPA1***	29111013	F641	P823	/	/	Caucasian	Non-EA	NM_015560.1	c.2708_2711del	p.Val903Glyfs*3	/	/	AD	truncation	heter	/	Dominant optic atrophy	/	/	/	Optic atrophy HP:	/	/	/	/	/	/	/	/	/	/	/	/	/	/	/	/	/	/	/	/	/	/	/	/
**AP3401**	***OPA1***	29111013	F642	P824	/	/	Caucasian	Non-EA	NM_015560.1	c.2708_2711del	p.Val903Glyfs*3	/	/	AD	truncation	heter	/	Dominant optic atrophy	/	/	/	Optic atrophy HP:	/	/	/	/	/	/	Hearing impairm	/	/	/	/	/	/	/	/	/	/	/	/	/	/	/	/	/
**AP3448**	***OPA1***	32025183	F672	P871	OA01	II-3	Chinese	EA	NM_015560.1	c.803A>G	p.Tyr268Cys	/		AD	M	heter	Male	Dominant optic atrophy	3.00	/	/	Optic atrophy HP:	/	/	/	/	/	/	/	/	/	/	/	/	/	/	/	60.00	YES	60.00	0.02	0.01	/	/	/	/
**AP3449**	***OPA1***	32025183	F673	P872	OA01	III-1	Chinese	EA	NM_015560.1	c.803A>G	p.Tyr268Cys	/		AD	M	heter	Female	Dominant optic atrophy	8.00	/	/	Optic atrophy HP:	/	/	/	/	/	/	/	/	/	/	/	/	/	/	/	38.00	YES	38.00	0.10	0.1	/	/	/	/
**AP2934**	***OPA1***	20157015	F256	P357	IT-5	78.00	Caucasian	Non-EA	NM_015560.1	c.2729T>A	p.Val910Asp		/	AD	M	heter	Female	Dominant optic atrophy	/	/	/	Optic atrophy HP:	/	/	/	/	/	/	/	/	/	/	/	/	/	/	/	59.00	YES				/	/	/	/
**AP2935**	***OPA1***	20157015	F256	P358	IT-5	79.00	Caucasian	Non-EA	NM_015560.1	c.2729T>A	p.Val910Asp		/	AD	M	heter	Male	Dominant optic atrophy	/	/	/	Optic atrophy HP:	/	/	/	/	/	/	/	Myopathy HP:00(	/	/	/	/	/	/	/	53.00	YES	/	/	/	/	/	/	/
[illegible]																																														

**AP3897**	***POLG***	21880868	F282	P286	/	II-15	Caucasian	Non-EA	NM_002693.2	c.2243G>C	p.Trp748Ser	c.2243G>C	p.Trp748Ser	AR	M+M	homo	/	POLG-related disease	/	/	Ataxia HP:00012	/	/	Ptosis HP:00005	Progressive exte	/	/	/
**AP3898**	***POLG***	21880868	F283	P287	/	II-16	Caucasian	Non-EA	NM_002693.2	c.2243G>C	p.Trp748Ser	c.2674_2675ir	p.Asp892Glyfs	AR	M+T	compound I	/	POLG-related disease	/	/	/	/	/	/	/	/	/	/
**AP3899**	***POLG***	21880868	F284	P288	/	II-17	Caucasian	Non-EA	NM_002693.2	c.911T>G	p.Leu304Arg	c.911T>G	p.Leu304Arg	AR	M+M	homo	/	POLG-related disease	/	/	Peripheral neuro	/	/	Ptosis HP:00005	Progressive exte	/	/	/
**AP3900**	***POLG***	21880868	F285	P289	/	II-18	Caucasian	Non-EA	NM_002693.2	c.2243G>C	p.Trp748Ser	c.2542G>A	p.Gly848Ser	AR	M+M	compound I	/	POLG-related disease	/	/	Seizure HP:0001	/	/	/	/	/	/	/
**AP3901**	***POLG***	21880868	F286	P290	/	II-19	Caucasian	Non-EA	NM_002693.2	c.2243G>C	p.Trp748Ser	c.1345C>T	p.Gln449*	AR	M+T	compound I	/	POLG-related disease	/	/	Seizure HP:0001	/	/	/	/	/	/	/
**AP3902**	***POLG***	21880868	F287	P291	/	II-20	Caucasian	Non-EA	NM_002693.2	c.1399G>A	p.Ala467Thr	/	/	AD	M	heter	/	POLG-related disease	/	/	Ataxia HP:00012	/	/	Ptosis HP:00005	Progressive exte	/	/	/
**AP3903**	***POLG***	21880868	F288	P292	/	II-21	Caucasian	Non-EA	NM_002693.2	c.2243G>C	p.Trp748Ser	c.2542G>A	p.Gly848Ser	AR	M+M	compound I	/	POLG-related disease	/	/	Seizure HP:0001	/	/	/	/	/	/	/
**AP3904**	***POLG***	21880868	F289	P293	/	II-22	Caucasian	Non-EA	NM_002693.2	c.1399G>A	p.Ala467Thr	c.3149delA	p.Lys1050Argf	AR	M+T	compound I	/	POLG-related disease	/	/	Neurodevelopme	/	/	/	/	/	/	/
**AP3905**	***POLG***	21880868	F290	P294	/	II-23	Caucasian	Non-EA	NM_002693.2	c.1399G>A	p.Ala467Thr	c.1399G>A	p.Ala467Thr	AR	M+M	homo	/	POLG-related disease	/	/	Ataxia HP:00012	/	/	Ptosis HP:00005	Progressive exte	/	/	/
**AP3906**	***POLG***	21880868	F291	P295	/	II-24	Caucasian	Non-EA	NM_002693.2	c.911T>G	p.Leu304Arg	c.911T>G	p.Leu304Arg	AR	M+M	homo	/	POLG-related disease	/	/	Neurodevelopme	/	/	/	/	/	/	/
**AP3907**	***POLG***	21880868	F292	P296	/	II-25	Caucasian	Non-EA	NM_002693.2	c.2243G>C	p.Trp748Ser	c.2542G>A	p.Gly848Ser	AR	M+M	compound I	/	POLG-related disease	/	/	/	/	/	/	/	/	/	/
**AP3908**	***POLG***	21880868	F293	P297	/	II-26	Caucasian	Non-EA	NM_002693.2	c.3286C>T	p.Arg1096Cys	c.3286C>T	p.Arg1096Cys	AR	M+M	homo	/	POLG-related disease	/	/	Seizure HP:0001	/	/	/	/	/	/	/
**AP3909**	***POLG***	21880868	F294	P298	/	II-27	Caucasian	Non-EA	NM_002693.2	c.2243G>C	p.Trp748Ser	c.2542G>A	p.Gly848Ser	AR	M+M	compound I	/	POLG-related disease	/	/	Abnormality of th	/	/	/	/	/	/	/
**AP3910**	***POLG***	21880868	F295	P299	/	II-28	Caucasian	Non-EA	NM_002693.2	c.911T>G	p.Leu304Arg	c.428C>T	p.Ala143Val	AR	M+M	compound I	/	POLG-related disease	/	/	Abnormality of th	/	/	/	Progressive exte	/	/	/
**AP3911**	***POLG***	21880868	F296	P300	/	II-29	Caucasian	Non-EA	NM_002693.2	c.2542G>A	p.Gly848Ser	c.2542G>A	p.Gly848Ser	AR	M+M	homo	/	POLG-related disease	/	/	Seizure HP:0001	/	/	/	/	/	/	/
**AP3912**	***POLG***	21880868	F297	P301	/	II-30	Caucasian	Non-EA	NM_002693.2	c.911T>G	p.Leu304Arg	c.911T>G	p.Leu304Arg	AR	M+M	homo	/	POLG-related disease	/	/	Ataxia HP:00012	/	/	Ptosis HP:00005	Progressive exte	/	/	/
**AP3913**	***POLG***	21880868	F298	P302	/	II-31	Caucasian	Non-EA	NM_002693.2	c.3286C>T	p.Arg1096Cys	c.3286C>T	p.Arg1096Cys	AR	M+M	homo	/	POLG-related disease	/	/	Ataxia HP:00012	/	/	/	/	/	/	/
**AP3914**	***POLG***	21880868	F299	P303	/	II-32	Caucasian	Non-EA	NM_002693.2	c.1399G>A	p.Ala467Thr	c.2243G>C	p.Trp748Ser	AR	M+M	compound I	/	POLG-related disease	/	/	Ataxia HP:00012	/	/	/	/	/	/	/
**AP3915**	***POLG***	21880868	F300	P304	/	II-33	Caucasian	Non-EA	NM_002693.2	c.2243G>C	p.Trp748Ser	c.2857C>T	p.Arg953Cys	AR	M+M	compound I	/	POLG-related disease	/	/	Ataxia HP:00012	/	/	Ptosis HP:00005	Progressive exte	/	/	/
**AP3916**	***POLG***	21880868	F301	P305	/	II-34	Caucasian	Non-EA	NM_002693.2	c.2542G>A	p.Gly848Ser	c.3286C>T	p.Arg1096Cys	AR	M+M	compound I	/	POLG-related disease	/	/	Ataxia HP:00012	/	/	Ptosis HP:00005	/	/	/	/
**AP3917**	***POLG***	21880868	F302	P306	/	II-35	Caucasian	Non-EA	NM_002693.2	c.1399G>A	p.Ala467Thr	c.1399G>A	p.Ala467Thr	AR	M+M	homo	/	POLG-related disease	/	/	Ataxia HP:00012	/	/	/	/	/	/	/
**AP3918**	***POLG***	21880868	F303	P307	/	II-36	Caucasian	Non-EA	NM_002693.2	c.1399G>A	p.Ala467Thr	c.1433+1G>A	p.?	AR	M+T	compound I	/	POLG-related disease	/	/	Ataxia HP:00012	/	/	Ptosis HP:00005	/	/	/	/
**AP3919**	***POLG***	21880868	F304	P308	/	II-37	Caucasian	Non-EA	NM_002693.2	c.1399G>A	p.Ala467Thr	c.1399G>A	p.Ala467Thr	AR	M+M	homo	/	POLG-related disease	/	/	Ataxia HP:00012	/	/	/	/	/	/	/
**AP3920**	***POLG***	21880868	F305	P309	/	II-38	Caucasian	Non-EA	NM_002693.2	c.1399G>A	p.Ala467Thr	c.1399G>A	p.Ala467Thr	AR	M+M	homo	/	POLG-related disease	/	/	Ataxia HP:00012	/	/	/	/	/	/	/
**AP3921**	***POLG***	21880868	F306	P310	/	II-39	Caucasian	Non-EA	NM_002693.2	c.752C>T	p.Thr251Ile	c.2794C>T	p.His932Tyr	AR	M+M	compound I	/	POLG-related disease	/	/	Ataxia HP:00012	/	/	Ptosis HP:00005	Progressive exte	/	/	/
**AP3922**	***POLG***	21880868	F307	P311	/	II-40	Caucasian	Non-EA	NM_002693.2	c.911T>G	p.Leu304Arg	c.911T>G	p.Leu304Arg	AR	M+M	homo	/	POLG-related disease	/	/	Ataxia HP:00012	/	/	Ptosis HP:00005	/	/	/	/
**AP3923**	***POLG***	21880868	F308	P312	/	II-41	Caucasian	Non-EA	NM_002693.2	c.2542G>A	p.Gly848Ser	c.3218C>T	p.Pro1073Leu	AR	M+M	compound I	/	POLG-related disease	/	/	Ataxia HP:00012	/	/	/	/	/	/	/
**AP3924**	***POLG***	21880868	F309	P313	/	II-42	Caucasian	Non-EA	NM_002693.2	c.3286C>T	p.Arg1096Cys	c.3286C>T	p.Arg1096Cys	AR	M+M	homo	/	POLG-related disease	/	/	Ataxia HP:00012	/	/	/	/	/	/	/
**AP3925**	***POLG***	21880868	F310	P314	/	II-43	Caucasian	Non-EA	NM_002693.2	c.752C>T	p.Thr251Ile	c.2794C>T	p.His932Tyr	AR	M+M	compound I	/	POLG-related disease	/	/	Ataxia HP:00012	/	/	Ptosis HP:00005	/	/	/	/
**AP3926**	***POLG***	21880868	F311	P315	/	II-44	Caucasian	Non-EA	NM_002693.2	c.752C>T	p.Thr251Ile	c.1760C>T	p.Pro587Leu	AR	M+M	compound I	/	POLG-related disease	/	/	Ataxia HP:00012	/	/	Ptosis HP:00005	/	/	/	/
**AP3927**	***POLG***	21880868	F312	P316	/	II-45	Caucasian	Non-EA	NM_002693.2	c.2243G>C	p.Trp748Ser	c.2740A>C	p.Thr914Pro	AR	M+M	compound I	/	POLG-related disease	/	/	Ataxia HP:00012	/	/	/	/	/	/	/
**AP3928**	***POLG***	21880868	F313	P317	/	II-46	Caucasian	Non-EA	NM_002693.2	c.2209G>C	p.Gly737Arg	c.1276G/A	p.Gly426Ser	AR	M+M	compound I	/	POLG-related disease	/	/	Ataxia HP:00012	/	/	/	/	/	/	/
**AP3929**	***POLG***	21880868	F314	P318	/	II-47	Caucasian	Non-EA	NM_002693.2	c.1399G>A	p.Ala467Thr	c.1399G>A	p.Ala467Thr	AR	M+M	homo	/	POLG-related disease	/	/	Ataxia HP:00012	/	/	/	Progressive exte	/	/	/
**AP3930**	***POLG***	21880868	F315	P319	/	II-48	Caucasian	Non-EA	NM_002693.2	c.2542G>A	p.Gly848Ser	c.428C>T	p.Ala143Val	AR	M+M	compound I	/	POLG-related disease	/	/	Ataxia HP:00012	/	/	/	/	/	/	/
**AP3931**	***POLG***	21880868	F316	P320	/	II-49	Caucasian	Non-EA	NM_002693.2	c.2542G>A	p.Gly848Ser	c.428C>T	p.Ala143Val	AR	M+M	compound I	/	POLG-related disease	/	/	Ataxia HP:00012	/	/	/	/	/	/	/
**AP3932**	***POLG***	21880868	F317	P321	/	II-50	Caucasian	Non-EA	NM_002693.2	c.1399G>A	p.Ala467Thr	c.3338T>C	p.Leu1113Pro	AR	M+M	compound I	/	POLG-related disease	/	/	Ataxia HP:00012	/	/	/	/	/	/	/
**AP3933**	***POLG***	21880868	F318	P322	/	II-51	Caucasian	Non-EA	NM_002693.2	c.2246T>C	p.Phe749Ser	c.3104+3A>T	p.?	AR	M+T	compound I	/	POLG-related disease	/	/	Ataxia HP:00012	/	/	Ptosis HP:00005	Progressive exte	/	/	/
**AP3934**	***POLG***	21880868	F319	P323	/	II-52	Caucasian	Non-EA	NM_002693.2	c.1399G>A	p.Ala467Thr	c.2740A>C	p.Thr914Pro	AR	M+M	compound I	/	POLG-related disease	/	/	Ataxia HP:00012	/	/	/	/	/	/	/
**AP3935**	***POLG***	21880868	F320	P324	/	II-53	Caucasian	Non-EA	NM_002693.2	c.2542G>A	p.Gly848Ser	c.3104+3A>T	p.?	AR	M+T	compound I	/	POLG-related disease	/	/	Ataxia HP:00012	/	Rod-cone dystro	Ptosis HP:00005	Progressive exte	/	/	/
**AP3936**	***POLG***	21880868	F321	P325	/	II-54	Caucasian	Non-EA	NM_002693.2	c.911T>G	p.Leu304Arg	c.911T>G	p.Leu304Arg	AR	M+M	homo	/	POLG-related disease	/	/	Ataxia HP:00012	/	/	Ptosis HP:00005	Progressive exte	/	Cataract HP:000	/
**AP3937**	***POLG***	21880868	F322	P326	/	II-55	Caucasian	Non-EA	NM_002693.2	c.915C>G	p.Ser305Arg	c.1399G>A	p.Ala467Thr	AR	M+M	compound I	/	POLG-related disease	/	/	Ataxia HP:00012	/	/	/	/	/	/	/
**AP3938**	***POLG***	21880868	F323	P327	/	II-56	Caucasian	Non-EA	NM_002693.2	c.1399G>A	p.Ala467Thr	c.2740A>C	p.Thr914Pro	AR	M+M	compound I	/	POLG-related disease	/	/	Ataxia HP:00012	/	/	/	/	/	/	/
**AP3939**	***POLG***	21880868	F324	P328	/	II-57	Caucasian	Non-EA	NM_002693.2	c.752C>T	p.Thr251Ile	c.3104+3A>T	p.?	AR	M+T	compound I	/	POLG-related disease	/	/	Ataxia HP:00012	/	/	Ptosis HP:00005	External ophthalr	/	/	/
**AP3940**	***POLG***	21880868	F325	P329	/	II-58	Caucasian	Non-EA	NM_002693.2	c.1399G>A	p.Ala467Thr	c.2246T>C	p.Phe749Ser	AR	M+M	compound I	/	POLG-related disease	/	/	Ataxia HP:00012	/	/	/	/	/	/	/
**AP3941**	***POLG***	21880868	F326	P330	/	II-59	Caucasian	Non-EA	NM_002693.2	c.2243G>C	p.Trp748Ser	c.2740A>C	p.Thr914Pro	AR	M+M	compound I	/	POLG-related disease	/	/	Ataxia HP:00012	/	/	/	/	/	/	/
**AP3942**	***POLG***	21880868	F327	P331	/	II-60	Caucasian	Non-EA	NM_002693.2	c.1399G>A	p.Ala467Thr	c.3285C>G	p.Ser1095Arg	AR	M+M	compound I	/	POLG-related disease	/	/	Ataxia HP:00012	/	/	/	/	/	/	/
**AP3943**	***POLG***	21880868	F328	P332	/	II-61	Caucasian	Non-EA	NM_002693.2	c.1399G>A	p.Ala467Thr	c.2740A>C	p.Thr914Pro	AR	M+M	compound I	/	POLG-related disease	/	/	Ataxia HP:00012	/	/	/	/	/	/	/
**AP3944**	***POLG***	21880868	F329	P333	/	II-62	Caucasian	Non-EA	NM_002693.2	c.1880G>A	p.Arg627Gln	c.2554C>T	p.Arg852Cys	AR	M+M	compound I	/	POLG-related disease	/	/	Ataxia HP:00012	/	/	/	/	/	/	/
**AP3945**	***POLG***	21880868	F330	P334	/	II-63	Caucasian	Non-EA	NM_002693.2	c.1399G>A	p.Ala467Thr	c.2740A>C	p.Thr914Pro	AR	M+M	compound I	/	POLG-related disease	/	/	Ataxia HP:00012	/	/	/	/	/	/	/
**AP3946**	***POLG***	21880868	F331	P335	/	II-64	Caucasian	Non-EA	NM_002693.2	c.1399G>A	p.Ala467Thr	c.1399G>A	p.Ala467Thr	AR	M+M	homo	/	POLG-related disease	/	/	Ataxia HP:00012	/	/	/	/	/	/	/
**AP3947**	***POLG***	21880868	F332	P336	/	II-65	Caucasian	Non-EA	NM_002693.2	c.1399G>A	p.Ala467Thr	c.3067C>T	p.Gln1023*	AR	M+T	compound I	/	POLG-related disease	/	/	Ataxia HP:00012	/	/	/	/	/	/	/
**AP3948**	***POLG***	21880868	F333	P337	/	II-66	Caucasian	Non-EA	NM_002693.2	c.2542G>A	p.Gly848Ser	c.428C>T	p.Ala143Val	AR	M+M	compound I	/	POLG-related disease	/	/	Ataxia HP:00012	/	/	/	/	/	/	/
**AP3949**	***POLG***	21880868	F334	P338	/	II-67	Caucasian	Non-EA	NM_002693.2	c.2542G>A	p.Gly848Ser	c.428C>T	p.Ala143Val	AR	M+M	compound I	/	POLG-related disease	/	/	Ataxia HP:00012	/	/	/	/	/	/	/
**AP3950**	***POLG***	21880868	F335	P339	/	II-68	Caucasian	Non-EA	NM_002693.2	c.1399G>A	p.Ala467Thr	c.2243G>C	p.Trp748Ser	AR	M+M	compound I	/	POLG-related disease	/	/	Ataxia HP:00012	/	/	/	/	/	/	/
**AP3951**	***POLG***	21880868	F336	P340	/	II-69	Caucasian	Non-EA	NM_002693.2	c.1399G>A	p.Ala467Thr	c.428C>T	p.Ala143Val	AR	M+M	compound I	/	POLG-related disease	/	/	Ataxia HP:00012	/	/	/	/	/	/	/
**AP3952**	***POLG***	21880868	F337	P341	/	II-70	Caucasian	Non-EA	NM_002693.2	c.911T>G	p.Leu304Arg	c.1399G>A	p.Ala467Thr	AR	M+M	compound I	/	POLG-related disease	/	/	Ataxia HP:00012	/	/	/	/	/	/	/
**AP3953**	***POLG***	21880868	F338	P342	/	II-71	Caucasian	Non-EA	NM_002693.2	c.1399G>A	p.Ala467Thr	c.2419C>T	p.Arg807Cys	AR	M+M	compound I	/	POLG-related disease	/	/	Ataxia HP:00012	/	/	/	/	/	/	/
**AP3954**	***POLG***	21880868	F339	P343	/	II-72	Caucasian	Non-EA	NM_002693.2	c.1399G>A	p.Ala467Thr	c.2740A>C	p.Thr914Pro	AR	M+M	compound I	/	POLG-related disease	/	/	Ataxia HP:00012	/	/	/	/	/	/	/
**AP3955**	***POLG***	21880868	F340	P344	/	II-73	Caucasian	Non-EA	NM_002693.2	c.1399G>A	p.Ala467Thr	c.2246T>C	p.Phe749Ser	AR	M+M	compound I	/	POLG-related disease	/	/	Ataxia HP:00012	/	/	/	/	/	/	/
**AP3956**	***POLG***	21880868	F341	P345	/	II-74	Caucasian	Non-EA	NM_002693.2	c.1399G>A	p.Ala467Thr	c.2262C>G	p.His754Gln	AR	M+M	compound I	/	POLG-related disease	/	/	Ataxia HP:00012	/	/	/	/	/	/	/
**AP3957**	***POLG***	21880868	F342	P346	/	II-75	Caucasian	Non-EA	NM_002693.2	c.1399G>A	p.Ala467Thr	c.2420G>A	p.Arg807His	AR	M+M	compound I	/	POLG-related disease	/	/	Ataxia HP:00012	/	/	/	/	/	/	/
**AP3958**	***POLG***	21880868	F343	P347	/	II-76	Caucasian	Non-EA	NM_002693.2	c.2243G>C	p.Trp748Ser	c.2391_2393d	p.Met797del	AR	M+M	compound I	/	POLG-related disease	/	/	Ataxia HP:00012	/	/	/	/	/	/	/
**AP3959**	***POLG***	21880868	F344	P348	/	II-77	Caucasian	Non-EA	NM_002693.2	c.2243G>C	p.Trp748Ser	c.2663G>A	p.Gly888Asp	AR	M+M	compound I	/	POLG-related disease	/	/	Ataxia HP:00012	/	/	/	/	/	/	/
**AP3960**	***POLG***	21880868	F345	P349	/	II-78	Caucasian	Non-EA	NM_002693.2	c.2243G>C	p.Trp748Ser	c.2555G>A	p.Arg852His	AR	M+M	compound I	/	POLG-related disease	/	/	Ataxia HP:00012	/	/	/	/	/	/	/
**AP3961**	***POLG***	21880868	F346	P350	/	II-79	Caucasian	Non-EA	NM_002693.2	c.2870C>T	p.Ala957Val	c.3229T>G	p.Cys1077Gly	AR	M+M	compound I	/	POLG-related disease	/	/	Ataxia HP:00012	/	/	/	/	/	/	/
**AP3962**	***POLG***	21880868	F347	P351	/	II-80	Caucasian	Non-EA	NM_002693.2	c.2209G>C	p.Gly737Arg	c.2563G>T	p.Val855Leu	AR	M+M	compound I	/	POLG-related disease	/	/	Ataxia HP:00012	/	/	/	/	/	/	/
**AP3963**	***POLG***	21880868	F348	P352	/	II-81	Caucasian	Non-EA	NM_002693.2	c.2209G>C	p.Gly737Arg	c.2870C>T	p.Ala957Val	AR	M+M	compound I	/	POLG-related disease	/	/	Ataxia HP:00012	/	/	/	/	/	/	/

[illegible]

AP4385	SDHA	28546994	F057	P068	/	/	Caucasian	Non-EA	NM_004168	c.1338delA	p.His447Metfs*23	/	/	AD	T	heter	Female	Cancer	/
AP4386	SDHA	28546994	F058	P069	/	/	Caucasian	Non-EA	NM_004168	c.1468G>T	p.Glu490Ter	/	/	AD	M	heter	Male	Cancer	/
AP4387	SDHA	28546994	F059	P070	/	/	Caucasian	Non-EA	NM_004168	c.1753C>T	p.Arg585Trp	/	/	AD	M	heter	Female	Cancer	/
AP4388	SDHA	28546994	F060	P071	/	/	Caucasian	Non-EA	NM_004168	c.1765C>T	p.Arg589Trp	/	/	AD	M	heter	Female	Cancer	/
AP4389	SDHA	28546994	F061	P072	/	/	Caucasian	Non-EA	NM_004168	c.1909-2A>G	p.?	/	/	AD	T	heter	Female	Cancer	/
AP4390	SDHA	28500238	F062	P073	/	1	Caucasian	Non-EA	NM_004168	c.91C>T	p.Arg31*	/	/	AD	T	heter	/	Paraganglioma	/
AP4391	SDHA	28500238	F063	P074	/	2	Caucasian	Non-EA	NM_004168	c.91C>T	p.Arg31*	/	/	AD	T	heter	/	Paraganglioma	/
AP4392	SDHA	28500238	F064	P075	/	3	Caucasian	Non-EA	NM_004168	c.1338delA	p.His447Metfs*23	/	/	AD	T	heter	/	Paraganglioma	/
AP4393	SDHA	28500238	F065	P076	/	4	Caucasian	Non-EA	NM_004168	c.1338delA	p.His447Metfs*23	/	/	AD	T	heter	/	Paraganglioma	/
AP4394	SDHA	28500238	F066	P077	/	5	Caucasian	Non-EA	NM_004168	c.1338delA	p.His447Metfs*23	/	/	AD	T	heter	/	Paraganglioma	/
AP4395	SDHA	28500238	F067	P078	/	6	Caucasian	Non-EA	NM_004168	c.1338delA	p.His447Metfs*23	/	/	AD	T	heter	/	Paraganglioma	/
AP4396	SDHA	28500238	F068	P079	/	7	Caucasian	Non-EA	NM_004168	c.1753C>T	p.Arg585Trp	/	/	AD	M	heter	/	Paraganglioma	/
AP4397	SDHA	28500238	F069	P080	/	8	Caucasian	Non-EA	NM_004168	c.923C>T	p.Thr308Met	/	/	AD	M	heter	/	Paraganglioma	/
AP4398	SDHA	28500238	F070	P081	/	9	Caucasian	Non-EA	NM_004168	c.91C>T	p.Arg31*	/	/	AD	T	heter	/	Paraganglioma	/
AP4399	SDHA	28500238	F071	P082	/	10	Caucasian	Non-EA	NM_004168	c.91C>T	p.Arg31*	/	/	AD	T	heter	/	Paraganglioma	/
AP4400	SDHA	28500238	F072	P083	/	11	Caucasian	Non-EA	NM_004168	c.91C>T	p.Arg31*	/	/	AD	T	heter	/	Paraganglioma	/
AP4401	SDHA	28384794	F073	P084	/	/	Caucasian	Non-EA	NM_004168	c.1A>C	p.Met1?	/	/	AD	T	heter	Male	tumour	/
AP4402	SDHA	28384794	F074	P085	/	/	Caucasian	Non-EA	NM_004168	c.1A>T	p.Met1?	/	/	AD	T	heter	Male	tumour	/
AP4403	SDHA	28384794	F075	P086	/	/	Caucasian	Non-EA	NM_004168	c.2T>G	p.?	/	/	AD	M	heter	Female	tumour	/
AP4404	SDHA	28384794	F076	P087	/	/	Caucasian	Non-EA	NM_004168	c.3G>C	p.?	/	/	AD	M	heter	Male	tumour	/
AP4405	SDHA	28384794	F077	P088	/	/	Caucasian	Non-EA	NM_004168	c.91C>T	p.Arg31*	/	/	AD	T	heter	/	tumour	/
AP4406	SDHA	28384794	F078	P089	/	/	Caucasian	Non-EA	NM_004168	c.223C>T	p.Arg75*	/	/	AD	T	heter	/	tumour	/
AP4407	SDHA	28384794	F079	P090	/	/	Caucasian	Non-EA	NM_004168	c.296A>G	p.His99Arg	/	/	AD	M	heter	Female	Pheochromocytoma and Paragang	/
AP4408	SDHA	28384794	F080	P091	/	/	Caucasian	Non-EA	NM_004168	c.457-1G>A	p.?	/	/	AD	M	heter	Female	Pheochromocytoma and Paragang	/
AP4409	SDHA	28384794	F081	P092	/	/	Caucasian	Non-EA	NM_004168	c.566G>A	p.Cys189Tyr	/	/	AD	M	heter	Male	Pheochromocytoma and Paragang	/
AP4410	SDHA	28384794	F082	P093	/	/	Caucasian	Non-EA	NM_004168	c.622T>C	p.Ser208Pro	/	/	AD	M	heter	Male	Pheochromocytoma and Paragang	/
AP4411	SDHA	28384794	F083	P094	/	/	Caucasian	Non-EA	NM_004168	c.629G>A	p.Arg210Gln	/	/	AD	M	heter	Male	Pheochromocytoma and Paragang	/
AP4412	SDHA	28384794	F084	P095	/	/	Caucasian	Non-EA	NM_004168	c.778G>A	p.Gly260Arg	/	/	AD	M	heter	Male	Pheochromocytoma and Paragang	/
AP4413	SDHA	28384794	F085	P096	/	/	Caucasian	Non-EA	NM_004168	c.820G>A	p.Gly274Ser	/	/	AD	M	heter	Male	Pheochromocytoma and Paragang	/
AP4414	SDHA	28384794	F086	P097	/	/	Caucasian	Non-EA	NM_004168	c.830C>T	p.Thr277Met	/	/	AD	M	heter	Female	Pheochromocytoma and Paragang	/
AP4415	SDHA	28384794	F087	P098	/	/	Caucasian	Non-EA	NM_004168	c.940G>A	p.Glu314Lys	/	/	AD	M	heter	Female	Pheochromocytoma and Paragang	/
AP4416	SDHA	28384794	F088	P099	/	/	Caucasian	Non-EA	NM_004168	c.1115C>G	p.Pro372Arg	/	/	AD	M	heter	Male	Pheochromocytoma and Paragang	/
AP4417	SDHA	28384794	F089	P100	/	/	Caucasian	Non-EA	NM_004168	c.1177G>A	p.Pro372Arg	/	/	AD	M	heter	Female	Pheochromocytoma and Paragang	/
AP4418	SDHA	28384794	F090	P101	/	/	Caucasian	Non-EA	NM_004168	c.1283_1298del	p.Gln428Profs*37	/	/	AD	T	heter	Male	Pheochromocytoma and Paragang	/
AP4419	SDHA	28384794	F091	P102	/	/	Caucasian	Non-EA	NM_004168	c.1316G>A	p.Gly439Glu	/	/	AD	M	heter	Female	Pheochromocytoma and Paragang	/
AP4420	SDHA	28384794	F092	P103	/	/	Caucasian	Non-EA	NM_004168	c.1334C>T	p.Ser445Leu	/	/	AD	M	heter	Female	Pheochromocytoma and Paragang	/
AP4421	SDHA	28384794	F093	P104	/	/	Caucasian	Non-EA	NM_004168	c.1340A>G	p.His447Arg	/	/	AD	M	heter	Male	Pheochromocytoma and Paragang	/
AP4422	SDHA	28384794	F094	P105	/	/	Caucasian	Non-EA	NM_004168	c.1361C>A	p.Ala454Glu	/	/	AD	M	heter	Male	Pheochromocytoma and Paragang	/
AP4423	SDHA	28384794	F095	P106	/	/	Caucasian	Non-EA	NM_004168	c.1432_1432+1del	p.?	/	/	AD	T	heter	Male	Pheochromocytoma and Paragang	/
AP4424	SDHA	28384794	F096	P107	/	/	Caucasian	Non-EA	NM_004168	c.1766G>A	p.Arg589Gln	/	/	AD	M	heter	Female	Pheochromocytoma and Paragang	/
AP4425	SDHA	28384794	F097	P108	/	/	Caucasian	Non-EA	NM_004168	c.1799G>A	p.Arg600Gln	/	/	AD	M	heter	/	Pheochromocytoma and Paragang	/
AP4426	SDHA	28384794	F098	P109	/	/	Caucasian	Non-EA	NM_004168	c.1979C>G	p.Ala660Gly	/	/	AD	M	heter	Male	Pheochromocytoma and Paragang	/
AP4427	SDHA	27683074	F099	P110	/	II-5	Caucasian	Non-EA	NM_004168	c.1351C>T	p.Arg451Cys	/	/	AD	M	heter	Male	Complex II Deficiency	/
AP4428	SDHA	27683074	F099	P111	/	III-1	Caucasian	Non-EA	NM_004168	c.1351C>T	p.Arg451Cys	/	/	AD	M	heter	Female	Complex II Deficiency	/
AP4429	SDHA	27683074	F099	P112	/	III-2	Caucasian	Non-EA	NM_004168	c.1351C>T	p.Arg451Cys	/	/	AD	M	heter	Male	Complex II Deficiency	/
AP4430	SDHA	26642834	F100	P113	/	8	Caucasian	Non-EA	NM_004168	c.1523C>T	p.Thr508Ile	c.1526C>T	p.Ser509Leu	AR	M+M	compound I	/	Leukoencephalopathy	/
AP4431	SDHA	26642834	F101	P114	/	14	Caucasian	Non-EA	NM_004168	c.771-772delGA	p.Gly258LeufsX6	c.1065-1260de	p.Gly356Serfs	AR	T+T	compound I	/	Leukoencephalopathy	/
AP4432	SDHA	26334176	F102	P115	/	/	Caucasian	Non-EA	NM_004168	c.1A>C	p.Met1Val	/	/	AD	M	heter	/	Leigh syndrome	/
AP4433	SDHA	26269449	F103	P116	/	424 (G)	Caucasian	Non-EA	NM_004168	c.1754G>A	p.Arg585Gln	/	/	AD	M	heter	/	Pheochromocytoma and Paragang	/
AP4434	SDHA	26269449	F104	P117	/	510 (G),	Caucasian	Non-EA	NM_004168	c.457-1G>A	p.?	/	/	AD	M	heter	/	Pheochromocytoma and Paragang	/
AP4435	SDHA	26269449	F105	P118	/	449 (G)	Caucasian	Non-EA	NM_004168	c.457-1G>A	p.?	/	/	AD	M	heter	/	Pheochromocytoma and Paragang	/
AP4436	SDHA	25595276	F106	P119	/	case 3	Caucasian	Non-EA	NM_004168	c.394T>C	p.Trp132Arg	/	/	AD	M	heter	/	Thyroid paraganglioma	/
AP4437	SDHA	25595276	F106	P119	/	case 4	Caucasian	Non-EA	NM_004168	c.394T>C	p.Trp132Arg	/	/	AD	M	heter	/	Thyroid paraganglioma	/
AP4438	SDHA	24781757	F107	P120	/	p1	Caucasian	Non-EA	NM_004168	c.356G>A	p.Trp119*	c.248C>T	p.Ala83_Gln1(	AR	M+T	compound I	/	Mitochondrial disorgers	/
AP4439	SDHA	24781757	F108	P121	/	p2	Caucasian	Non-EA	NM_004168	c.91C>T	p.Arg31*	c.565T>G	p.Cys189Gly	AR	M+T	compound I	/	Mitochondrial disorgers	/
AP4440	SDHA	24781757	F109	P122	/	p3	Caucasian	Non-EA	NM_004168	c.1065-3C>A	p.?	c.1065-3C>A	p.?	AR	T+T	homo	/	Mitochondrial disorgers	/
AP4441	SDHA	24781757	F110	P123	/	p4	Caucasian	Non-EA	NM_004168	c.64-2A>G	p.?	c.64-2A>G	p.?	AR	T+T	homo	/	Mitochondrial disorgers	/
AP4442	SDHA	24694336	F111	P124	/	/	Caucasian	Non-EA	NM_004168	c.629G>A	p.Arg210Gln	/	/	AD	M	heter	/	Pheochromocytoma and Paragang	/
AP4443	SDHA	23849264	F112	P125	/	/	Caucasian	Non-EA	NM_004168	c.117del	p.Asn40Thrfs*18	c.220_221insT	p.Leu74Phefs*	AR	T+T	compound I	Male	Encephalopathy	/
AP4444	SDHA	23750034	F113	P126	/	/	Caucasian	Non-EA	NM_004168	c.223C>T	p.Arg75*	/	/	AD	T	heter	Female	Paraganglioma	/
AP4445	SDHA	23666964	F114	P127	/	/	Caucasian	Non-EA	NM_004168	c.1338delA	p.His447Metfs*23	/	/	AD	M	heter	/	Pheochromocytoma and Paragang	/
AP4446	SDHA	23666964	F115	P128	/	/	Caucasian	Non-EA	NM_004168	c.136A>G	p.Lys46Glu	/	/	AD	M	heter	/	Pheochromocytoma and Paragang	/
AP4447	SDHA	23666964	F116	P129	/	/	Caucasian	Non-EA	NM_004168	c.91C>T	p.Arg31*	/	/	AD	T	heter	/	Pheochromocytoma and Paragang	/
AP4448	SDHA	23666964	F117	P130	/	/	Caucasian	Non-EA	NM_004168	c.91C>T	p.Arg31*	/	/	AD	T	heter	/	Pheochromocytoma and Paragang	/
AP4449	SDHA	23666964	F118	P131	/	/	Caucasian	Non-EA	NM_004168	c.549C>T	p.=	/	/	AD	T	heter	/	Pheochromocytoma and Paragang	/
AP4450	SDHA	23666964	F119	P132	/	/	Caucasian	Non-EA	NM_004168	c.822C>T	p.=	/	/	AD	T	heter	/	Pheochromocytoma and Paragang	/
AP4451	SDHA	23666964	F120	P133	/	/	Caucasian	Non-EA	NM_004168	c.1002G>A	p.?	/	/	AD	T	heter	/	Pheochromocytoma and Paragang	/
AP4452	SDHA	23666964	F121	P134	/	/	Caucasian	Non-EA	NM_004168	c.923C>T	p.Thr308Met	/	/	AD	M	heter	/	Pheochromocytoma and Paragang	/
AP4453	SDHA	23666964	F122	P135	/	/	Caucasian	Non-EA	NM_004168	c.1273G>A	p.Val425Met	/	/	AD	M	heter	/	Pheochromocytoma and Paragang	/
AP4454	SDHA	23666964	F123	P136	/	/	Caucasian	Non-EA	NM_004168	c.1623G>A	p.=	/	/	AD	T	heter	/	Pheochromocytoma and Paragang	/
AP4455	SDHA	23666964	F124	P137	/	/	Caucasian	Non-EA	NM_004168	c.1753C>T	p.Arg585Trp	/	/	AD	M	heter	/	Pheochromocytoma and Paragang	/
AP4456	SDHA	23666964	F125	P138	/	/	Caucasian	Non-EA	NM_004168	c.1776T>C	p.Arg589Gln	/	/	AD	M	heter	/	Pheochromocytoma and Paragang	/
AP4457	SDHA	23633203	F126	P139	/	/	Caucasian	Non-EA	NM_004168	c.1873C>T	p.His625Tyr	c.1865G>A	p.Trp622*	AR	M+T	compound I	/	Paraganglioma	/
AP4458	SDHA	23282968	F127	P140	/	case 1	Caucasian	Non-EA	NM_004168	c.91C>T	p.Arg31*	/	/	AD	T	heter	/	Gastrointestinal Stromal Tumors	/
AP4459	SDHA	23282968	F128	P141	/	case 2	Caucasian	Non-EA	NM_004168	c.91C>T	p.Arg31*	/	/	AD	T	heter	/	Gastrointestinal Stromal Tumors	/
AP4460	SDHA	23282968	F129	P142	/	case 3	Caucasian	Non-EA	NM_004168	c.91C>T	p.Arg31*	/	/	AD	T	heter	/	Gastrointestinal Stromal Tumors	/
AP4461	SDHA	23282968	F130	P143	/	case 4	Caucasian	Non-EA	NM_004168	c.767C>T	p.Thr256Ile	/	/	AD	M	heter	/	Gastrointestinal Stromal Tumors	/
AP4462	SDHA	23282968	F131	P144	/	case 5	Caucasian	Non-EA	NM_004168	c.1334C>T	p.Ser445Leu	/	/	AD	M	heter	/	Gastrointestinal Stromal Tumors	/
AP4463	SDHA	23282968	F132	P145	/	case 6	Caucasian	Non-EA	NM_004168	c.1794G>C	p.K598N	/	/	AD	M	heter	/	Gastrointestinal Stromal Tumors	/
AP4464	SDHA	23282968	F133	P146	/	case 7	Caucasian	Non-EA	NM_004168	c.1795-1G>T	p.?	/	/	AD	T	heter	/	Gastrointestinal Stromal Tumors	/
AP4465	SDHA	23109135	F134	P147	/	case 1	Caucasian	Non-EA	NM_004168	c.818C>T	p.Thr273Ile	c.1357G > A	p.Gly453Arg	AR	M+M	compound I	/	Gastrointestinal Stromal Tumors	/
AP4466	SDHA	23109135	F135	P148	/	case 2	Caucasian	Non-EA	NM_004168	c.457-2_c457delAGC	p.Leu153Lysfs*71	/	/	AD	T	heter	/	Gastrointestinal Stromal Tumors	/
AP4467	SDHA	23109135	F136	P149	/	case 3	Caucasian	Non-EA	NM_004168	c.91C>T	p.Arg31*	/	/	AD	T	heter	/	Gastrointestinal Stromal Tumors	/
AP4468	SDHA	23109135	F137	P150	/	case 4	Caucasian	Non-EA	NM_004168	c.91C>T	p.Arg31*	/	/	AD	T	heter	/	Gastrointestinal Stromal Tumors	/
AP4469	SDHA	23109135	F138	P151	/	case 5	Caucasian	Non-EA	NM_004168	c.1471G>T	p.Glu491*	/	/	AD	T	heter	/	Gastrointestinal Stromal Tumors	/
AP4470	SDHA	22974104	F139	P152	/	case 1	Caucasian	Non-EA	NM_004168	c.91C>T	p.Arg31*	/	/	AD	T	heter	Male	Gastrointestinal Stromal Tumors	/
AP4471	SDHA	22974104	F140	P153	/	case 2	Caucasian	Non-EA	NM_004168	c.91C>T	p.Arg31*	/	/	AD	T	heter	Female	Gastrointestinal Stromal Tumors	/
AP4472	SDHA	22974104	F141	P154	/	/	Caucasian	Non-EA	NM_004168	c.91C>T	p.Arg31*	/	/	AD	T	heter	/	Gastrointestinal Stromal Tumors	/
AP4473	SDHA	22974104	F142	P155	/	/	Caucasian	Non-EA	NM_004168	c.91C>T	p.Arg31*	/	/	AD	T	heter	/	Gastrointestinal Stromal Tumors	/
AP4474	SDHA	22974104	F143	P156	/	/	Caucasian	Non-EA	NM_004168	c.91C>T	p.Arg31*	/	/	AD	T	heter	/	Gastrointestinal Stromal Tumors	/
AP4475	SDHA	22974104	F144	P157	/	/	Caucasian	Non-EA	NM_004168	c.91C>T	p.Arg31*	/	/	AD	T	heter	/	Gastrointestinal Stromal Tumors	/
AP4476	SDHA	22972948	F145	P158	/	Patient 1	Caucasian	Non-EA	NM_004168	c.1523C>T	p.Thr508Ile	c.1526C>T	p.Ser509Leu	AR	M+M	compound I	/		/
AP4477	SDHA	22955521	F146	P159	/	Case 1	Caucasian	Non-EA	NM_004168	c.553C>T	p.Gln185*	c.1043-1055de	p.?	AR	M+T	compound I	Male	Gastrointestinal Stromal Tumors	/
AP4478	SDHA	22955521	F147	P160	/	Case 2	Caucasian	Non-EA	NM_004168	c.1534C>T	p.Arg512*	/	/	AD	T	heter	Female	Gastrointestinal Stromal Tumors	/
AP4479	SDHA	22955521	F148	P161	/	Case 3	Caucasian	Non-EA	NM_004168	c.688delG	p.Asp223fs	/	/	AD	T	heter	Female	Gastrointestinal Stromal Tumors	/
AP4480	SDHA	22955521	F149	P162	/	Case 4	Caucasian	Non-EA	NM_004168	c.91C>T	p.Arg31*	/	/	AD	T	heter	Male	Gastrointestinal Stromal Tumors	/
AP4481	SDHA	22955521	F150	P163	/	Case 5	Caucasian	Non-EA	NM_004168	c.985C>T	p.Arg329*	/	/	AD	T	heter	Male	Gastrointestinal Stromal Tumors	/
AP4482	SDHA	22955521	F151	P164	/	Case 6	Caucasian	Non-EA	NM_004168	c.1765C>T	p.Arg589Trp	/	/	AD	M	heter	Male	Gastrointestinal Stromal Tumors	/
AP4483	SDHA	22955521	F152	P165	/	Case 7	Caucasian	Non-EA	NM_004168	c.91C>T	p.Arg31*	/	/	AD	T	heter	Female	Gastrointestinal Stromal Tumors	/
AP4484	SDHA	22955521	F153	P166	/	Case 8	Caucasian	Non-EA	NM_004168	c.91C>T	p.Arg31*	c.985C>T	p.Arg329*	AR	T+T	compound I	Female	Gastrointestinal Stromal Tumors	/
AP4485	SDHA	21753896	F154	P167	/	Case 9	Caucasian	Non-EA	NM_004168	c.91C>T	p.Arg31*	/	/	AD	T	heter	/	Gastrointestinal Stromal Tumors	/
AP4486	SDHA	21753896	F155	P168	/	/	Caucasian	Non-EA	NM_004168	c.91C>T	p.Arg31*	/	/	AD	T	heter	/	Gastrointestinal Stromal Tumors	/
AP4487	SDHA	21753896	F156	P169	/	/	Caucasian	Non-EA	NM_004168	c.91C>T	p.Arg31*	/	/	AD	T	heter	/	Gastrointestinal Stromal Tumors	/
AP4488	SDHA	21753896	F157	P170	/	/	Caucasian	Non-EA	NM_004168	c.91C>T	p.Arg31*	/	/	AD	T	heter	/	Gastrointestinal Stromal Tumors	/
AP4489	SDHA	21753896	F158	P171	/	/	Caucasian	Non-EA	NM_004168	c.91C>T	p.Arg31*	/	/	AD	T	heter	/	Gastrointestinal Stromal Tumors	/
AP4490	SDHA	21753896	F159	P172	/	/	Caucasian	Non-EA	NM_004168	c.1753C>T	p.Arg585Trp	/	/	AD	M	heter	/	Gastrointestinal Stromal Tumors	/
AP4491	SDHA	20551992	F160	P173	A	A6	Bedouin	Non-EA	NM_004168	c.1664G>A	p.Gly555Glu	/	/	AD	M	heter	Female	Cardiomyopathy	/
AP4492	SDHA	20551992	F160	P174	A	A7	Bedouin	Non-EA	NM_004168	c.1664G>A	p.Gly555Glu	/	/	AD	M	heter	Male	Cardiomyopathy	/
AP4493	SDHA	20551992	F160	P175	A	A8	Bedouin	Non-EA	NM_004168	c.1664G>A	p.Gly555Glu	/	/	AD	M	heter	Female	Cardiomyopathy	/
AP4494	SDHA	20551992	F160	P176	A	A9	Bedouin	Non-EA	NM_004168	c.1664G>A	p.Gly555Glu	/	/	AD	M	heter	Male	Cardiomyopathy	/
AP4495	SDHA	20551992	F160	P177	A	A10	Bedouin	Non-EA	NM_004168	c.1664G>A	p.Gly555Glu	/	/	AD	M	heter	Female	Cardiomyopathy	/
AP4496	SDHA	20551992	F160	P178	A	A11	Bedouin	Non-EA	NM_004168	c.1664G>A	p.Gly555Glu	/	/	AD	M	heter	Female	Cardiomyopathy	/
AP4497	SDHA	20551992	F160	P179	A	A12	Bedouin	Non-EA	NM_004168	c.1664G>A	p.Gly555Glu	/	/	AD	M	heter	Male	Cardiomyopathy	/
AP4498	SDHA	20551992	F160	P180	A	A13	Bedouin	Non-EA	NM_004168	c.1664G>A	p.Gly555Glu	/	/	AD	M	heter	Male	Cardiomyopathy	/
AP4499	SDHA	20551992	F161	P181	B	B1	Bedouin	Non-EA	NM_004168	c.1664G>A	p.Gly555Glu	/	/	AD	M	heter	Male	Cardiomyopathy	/
AP4500	SDHA	20551992	F161	P182	B	B2	Bedouin	Non-EA	NM_004168	c.1664G>A	p.Gly555Glu	/	/	AD	M	heter	Female	Cardiomyopathy	/
AP4501	SDHA	20551992	F161	P183	B	B3	Bedouin	Non-EA	NM_004168	c.1664G>A	p.Gly555Glu	/	/	AD	M	heter	Female	Cardiomyopathy	/
AP4502	SDHA	20551992	F161	P184	B	B4	Bedouin	Non-EA	NM_004168	c.1664G>A	p.Gly555Glu	/	/	AD	M	heter	Female	Cardiomyopathy	/
AP4503	SDHA	20551992	F161	P185	B	B5	Bedouin	Non-EA	NM_004168	c.1664G>A	p.Gly555Glu	/	/	AD	M	heter	Male	Cardiomyopathy	/
AP4504	SDHA	20551992	F162	P186	C	C1	Bedouin	Non-EA	NM_004168	c.1664G>A	p.Gly555Glu	/	/	AD	M	heter	Male	Cardiomyopathy	/
AP4505	SDHA	20551992	F163	P187	D	D1	Bedouin	Non-EA	NM_004168	c.1664G>A	p.Gly555Glu	/	/	AD	M	heter	Male	Cardiomyopathy	/
AP4506	SDHA	16361598	F165	P189	/	/	Caucasian	Non-EA	NM_004168	c.356G>A	p.Trp119*	c.248C>T	p.Ala83Val	AR	M	compound I	/	Leigh syndrome	/
AP4507	SDHA	12794685	F166	P190	/	/	Caucasian	Non-EA	NM_004168	c1664G>A	p.Gly555Glu	/	/	AD	M	heter	/	Complex II Deficiency	/
AP4508	SDHA	10976639/8967	F167	P191	/	/	Caucasian	Non-EA	NM_004168	c.1351C>T	p.Arg451Cys	/	/	AD	M	heter	Female	/	/
AP4509	SDHA	10976639/8967	F168	P192	/	/	Caucasian	Non-EA	NM_004168	c.1351C>T	p.Arg451Cys	/	/	AR	M	heter	Female	/	/
AP4510	SDHA	10746566	F169	P193	/	/	Caucasian	Non-EA	NM_004168	c.1571C>T	p.Ala524Val	c.1A>C	p.Met1?	AR	M+T	compound I	/	Complex II Deficiency	/
AP4511	SLC25A46	35012485	F001	P001	/	proband	Caucasian	Non-EA	NM_138773	c.385-1G>A	p.?	c.385-1G>A	p.?	AR	T+T	homo	Male	Pontocerebellar hypoplasia	/
AP4512	SLC25A46	35012485	F001	P002	/	/	Caucasian	Non-EA	NM_138773	c.385-1G>A	p.?	c.385-1G>A	p.?	AR	T+T	homo	Male	Pontocerebellar hypoplasia	/
AP4513	SLC25A46	33369814	F002	P003	/	F31/	Caucasian	Non-EA	NM_138773	c.106G>A	p.Gly36Arg	c.106G>A	p.Gly36Arg	AR	M+M	homo	/	Distal hereditary motor neuropathy	/
AP4514	SLC25A46	33144682	F003	P004	/	VLC68	Caucasian	Non-EA	NM_138773	c.468_471dup	p.Ala158*	c.731T>C	p.Ile244Thr	AR	T+M	compound I	/	Distal hereditary motor neuropathy	/
AP4515	SLC25A46	32259769	F004	P005	/	Tn-1	Caucasian	Non-EA	NM_138773	c.410A>G	p.His137Arg	c.1198_1199ir	p.A401Sfs*17	AR	M+T	compound I	Female	Parkinson's Disease and optic atro	16.00
AP4516	SLC25A46	32259769	F005	P006	/	Ps1	Caucasian	Non-EA	NM_138773	c.527C>T	p.Aal176Val	c.767A>G	p.Lys256Arg	AR	M+M	compound I	Female	Parkinson's Disease and optic atro	52.00
AP4517	SLC25A46	31607746	F006	P007	/	3	Caucasian	Non-EA	NM_138773	c.385-852_385-739del	p.?	c.992T>C	p.Leu331Pro	AR	M+T	compound I	Male	neurodevelopmental delay	/
AP4518	SLC25A46	30178502	F007	P008	/	/	Turkish	Non-EA	NM_138773	c.770G>A	p.Arg257Gln	c.770G > A	p.Arg257Gln	AR	M+M	homo	/	neurodegenerative disorders with	0.67
AP4519	SLC25A46	28653766	F008	P009	Family 1	/	German	Non-EA	NM_138773	c.736A>T	p.Arg246*	c.736A>T	p.Arg246*	AR	T+T	homo	Male	Pontocerebellar hypoplasia	/
AP4520	SLC25A46	28653766	F008	P010	Family 1	/	German	Non-EA	NM_138773	c.736A>T	p.Arg246*	c.736A>T	p.Arg246*	AR	T+T	homo	Female	Pontocerebellar hypoplasia	/
AP4521	SLC25A46	28653766	F009	P011	Family 2	/	Italian	Non-EA	NM_138773	c.42C>G	p.[Y14*]	c.462+ 1G>A	?	AR	T+T	compound I	Male	Pontocerebellar hypoplasia	/
AP4522	SLC25A46	28653766	F009	P012	Family 2	/	Italian	Non-EA	NM_138773	c.42C>G	p.[Y14*]	c.462+ 1G>A	?	AR	T+T	compound I	Female	Pontocerebellar hypoplasia	/
AP4523	SLC25A46	28637197	F010	P013	/	/	Dutch	Non-EA	NM_138773	c.691C>T	p.Arg231*	del exon 3	p.?	AR	T+T	compound I	/	Pontocerebellar hypoplasia	/
AP4524	SLC25A46	28558379	F011	P014	family G	G2	Turkish	Non-EA	NM_138773	c.1018C>T	p.Arg340Cys	c.1018C>T	p.Arg340Cys	AR	M+M	homo	Male	Cerebellar Ataxia	10.00
AP4525	SLC25A46	28558379	F011	P015	family G	G3	Turkish	Non-EA	NM_138773	c.1018C>T	p.Arg340Cys	c.1018C>T	p.Arg340Cys	AR	M+M	homo	Male	Cerebellar Ataxia	6.00
AP4526	SLC25A46	28558379	F012	P016	families AAR	1	Algerian	Non-EA	NM_138773	c.479G>C	p.Trp160Ser	c.479G>C	p.Trp160Ser	AR	M+M	homo	Male	Cerebellar Ataxia	2.00
AP4527	SLC25A46	28558379	F013	P017	families AAR	/	Algerian	Non-EA	NM_138773	c.479G>C	p.Trp160Ser	c.479G>C	p.Trp160Ser	AR	M+M	homo	Male	Cerebellar Ataxia	/
AP4528	SLC25A46	28369803	F014	P018	/	/	Saudi	Non-EA	NM_138773	c.1018C>T	p.Arg340Cys	c.1018C>T	p.Arg340Cys	AR	M+M	homo	Female	Optic atrophy and progressive limb	/
AP4529	SLC25A46	27543974	F015	P019	/	/	Caucasian	Non-EA	NM_138773	c.1022T>C	p.Leu341Pro	c.1022T>C	p.Leu341Pro	AR	M+M	homo	Male	Pontocerebellar hypoplasia	/
AP4530	SLC25A46	27543974	F016	P020	/	/	Caucasian	Non-EA	NM_138773	del exon 1	p.?	del exon 1	p.?	AR	CNV+cnv	homo	Female	Pontocerebellar hypoplasia	/
AP4531	SLC25A46	27430653	F017	P021	/	/	Pakistan	Non-EA	NM_138773	c.413T>G	p.Leu138Arg	c.413T > G	p.Leu138Arg	AR	M+M	homo	Male	Progressive Myoclonic Ataxia With	/
AP4532	SLC25A46	27390132	F018	P022	/	/	French Canadian	Non-EA	NM_138773	c.425 > T	p.Thr142Ile	c.425C > T	p.Thr142Ile	AR	M+M	homo	Male	Leigh syndrome	/
AP4533	SLC25A46	26951855	F019	P023	/	/	Moroccan	Non-EA	NM_138773	c.283+3G>T	p.Ser32Thrfs*4	c.283+3G>T	p.Ser32Thrfs*4	AR	T+T	compound I	Female	Optic atrophy plus	0.00
AP4534	SLC25A46	26168012	F020	P024	UK	II-2	British	Non-EA	NM_138773	c.165_166insC	p.His56fs*94	c.746G>A	p.Gly249Asp	AR	M+T	compound I	Female	Optic atrophy plus	8.00
AP4535	SLC25A46	26168012	F020	P025	UK	II-1	British	Non-EA	NM_138773	c.165_166insC	p.His56fs*94	c.746G>A	p.Gly249Asp	AR	M+T	compound I	Female	Optic atrophy plus	5.00
AP4536	SLC25A46	26168012	F021	P026	IT	II-3	Italian	Non-EA	NM_138773	c.1018C>T	p.Arg340Cys	c.1018C>T	p.Arg340Cys	AR	M+M	homo	Male	Optic atrophy plus	2.00
AP4537	SLC25A46	26168012	F022	P027	PL	II-5	Palestine	Non-EA	NM_138773	c.1005A>T	p.Glu335Asn	c.1005A>T	p.Glu335Asn	AR	M+M	homo	Female	Optic atrophy plus	1.83
AP4538	SLC25A46	26168012	F023	P028	US	/	Caucasian	Non-EA	NM_138773	c.882_885dup TTAC	p.Asn296fs*297	c.998C>T	p.Pro333Leu	AR	M+T	compound I	/		0.00
AP4539	SLC52A2	33713422	F001	P001	L-950124	/	Iranian	Non-EA	NM_024531.4	c.973T>G	p.Cys325Gly	c.973T>G	p.Cys325Gly	AR	M+M	homo	/	Hearing loss	/
AP4540	SLC52A2	32827528	F002	P002	/	/	Caucasian	Non-EA	NM_024531.4	c.696_703del	p.Leu233Glyfs	c.1250T>C	p.Leu417Pro	AR	?	compound I	Male	/	/
AP4541	SLC52A2	31152317	F003	P003	/	/	Caucasian	Non-EA	NM_024531.4	c.167C>T	p.Ala56Val	c.593G>A	p.Trp198*	AR	M+T	compound I	/	Hearing loss	/
AP4542	SLC52A2	31064337	F004	P004	/	/	Chinese	EA	NM_024531.4	c.1328G>A	p.Cys443Tyr	c.1022_1023i	p.Leu341Profs	AR	M+T	compound I	Female	Brown-Vialetto-Van Laere syndron	/
AP4543	SLC52A2	29961509	F005	P005	/	individual 1	Caucasian	Non-EA	NM_024531.4	c.505C>T	p.Arg169Cys	c.505C>T	p.Arg169Cys	AR	M+M	homo	Male	Brown-Vialetto-Van Laere syndron	/
AP4544	SLC52A2	29961509	F005	P006	/	individual 2	Caucasian	Non-EA	NM_024531.4	c.505C>T	p.Arg169Cys	c.505C>T	p.Arg169Cys	AR	M+M	homo	Male	Brown-Vialetto-Van Laere syndron	/
AP4545	SLC52A2	29915382	F006	P007	/	/	Caucasian	Non-EA	NM_024531.4	c.505C>T	p.Arg169Cys	c.505C>T	p.Arg169Cys	AR	M+M	homo	Female	Ataxia	/
AP4546	SLC52A2	29913018	F007	P008	/	NLDT9	Caucasian	Non-EA	NM_024531.4	c.401C>T	p.Pro134Leu	c.401C>T	p.Pro134Leu	AR	M+M	homo	Female	Movement Disorders	/
AP4547	SLC52A2	29913018	F008	P009	/	WHA9	Caucasian	Non-EA	NM_024531.4	c.916G>A	p.Gly306Arg	c.916G>A	p.Gly306Arg	AR	M+M	homo	Female	Movement Disorders	/
AP4548	SLC52A2	29858556	F009	P010	/	6	Caucasian	Non-EA	NM_024531.4	c.-110-1G>A	p.?	c.297G>C	p.Trp99Cys	AR	M+T	compound I	/	Muscle atrophy	/
AP4549	SLC52A2	29287867	F010	P011	/	II-2	Caucasian	Non-EA	NM_024531.4	c.973T>G	p.Cys325Gly	c.973T>G	p.Cys325Gly	AR	M+M	homo	Male	Ataxia	/
AP4550	SLC52A2	29287867	F010	P012	/	II-3	Caucasian	Non-EA	NM_024531.4	c.973T>G	p.Cys325Gly	c.973T>G	p.Cys325Gly	AR	M+M	homo	Male	Ataxia	/
AP4551	SLC52A2	29287867	F010	P013	/	II-4	Caucasian	Non-EA	NM_024531.4	c.973T>G	p.Cys325Gly	c.973T>G	p.Cys325Gly	AR	M+M	homo	Male	Ataxia	/
AP4552	SLC52A2	29193829	F016	P014	/	patient 1	Caucasian	Non-EA	NM_024531.4	c.917G>A	p.Gly306Glu	c.917G>A	p.Gly306Glu	AR	M+M	homo	Male	Neurodevelopmental delay	/
AP4553	SLC52A2	29053833	F017	P015	/	AP1	Caucasian	Non-EA	NM_024531.4	c.935T>C	p.Leu312Pro	c.935T>C	p.Leu312Pro	AR	M+M	homo	Female	Sensorimotor neuropathy	0.6
AP4554	SLC52A2	29053833	F018	P016	/	AP2	Caucasian	Non-EA	NM_024531.4	c.383C>T	p.Ser128Leu	c.1088C>T	p.Pro363Leu	AR	M+M	compound I	Female	Sensorimotor neuropathy	/
AP4555	SLC52A2	29053833	F019	P017	/	AP6	Caucasian	Non-EA	NM_024531.4	c.1016T>C	p.Leu339Pro	c.935T>C	p.Leu312Pro	AR	M+M	compound I	Male	Sensorimotor neuropathy	/
AP4556	SLC52A2	29053833	F020	P018	/	AM3	Caucasian	Non-EA	NM_024531.4	c.231G>A	p.Glu77Lys	c.865C>T	p.Ala288Val	AR	M+M	compound I	Female	Sensorimotor neuropathy	/
AP4557	SLC52A2	29053833	F021	P019	/	AM5	Caucasian	Non-EA	NM_024531.4	c.1327T>C	p.Cys443Arg	c.1327T>C	p.Cys443Arg	AR	M+M	homo	Male	Sensorimotor neuropathy	/
AP4558	SLC52A2	29053833	F022	P020	/	AM6	Caucasian	Non-EA	NM_024531.4	c.1327T>C	p.Cys443Arg	c.1327T>C	p.Cys443Arg	AR	M+M	homo	Female	Sensorimotor neuropathy	/
AP4559	SLC52A2	28251916	F023	P021	F25	P49	Caucasian	Non-EA	NM_024531.4	c.916G>A	p.Gly306Arg	c.1016T>C	p.Leu339Pro	AR	M+M	compound I	Female	Ataxia	/
AP4560	SLC52A2	28251916	F024	P022	F32	P56	Caucasian	Non-EA	NM_024531.4	c.819C>T	p.Met273Ile	c.1371C>G	p.Phe457Leu	AR	M+M	compound I	Male	Ataxia	/
AP4561	SLC52A2	27702554	F025	P023	/	/	Caucasian	Non-EA	NM_024531.4	c.421C>A	p.Pro141Thr	c.421C>A	p.Pro141Thr	AR	M+M	homo	Male	Brown-Vialetto-Van Laere syndron	/
AP4562	SLC52A2	27148561	F026	P024	/	/	Caucasian	Non-EA	NM_024531.4	c.808C>T	p.Gln270*	c.1016T>C	p.Leu339Pro	AR	M+M	compound I	Female	Neurodevelopmental delay	/
AP4563	SLC52A2	26846157	F027	P025	/	/	Caucasian	Non-EA	NM_024531.4	c.1327T>C	p.Cys443Arg	c.1327T>C	p.Cys443Arg	AR	M+M	homo	Female	Neurodevelopmental delay	/
AP4564	SLC52A2	26669662	F028	P026	family A	uncle	Caucasian	Non-EA	NM_024531.4	c.916G>A	p.Gly306Arg	c.916G>A	p.Gly306Arg	AR	M+M	homo	Male	Ataxia	/
AP4565	SLC52A2	26669662	F028	P027	family A	niece	Caucasian	Non-EA	NM_024531.4	c.916G>A	p.Gly306Arg	c.916G>A	p.Gly306Arg	AR	M+M	homo	Female	Ataxia	/
AP4566	SLC52A2	26669662	F029	P028	family B	/	Caucasian	Non-EA	NM_024531.4	c.401C>T	p.Pro134Leu	c.401C>T	p.Pro134Leu	AR	M+M	homo	Female	Ataxia	/
AP4567	SLC52A2	26669662	F029	P029	family B	/	Caucasian	Non-EA	NM_024531.4	c.401C>T	p.Pro134Leu	c.401C>T	p.Pro134Leu	AR	M+M	homo	Female	Ataxia	/
AP4568	SLC52A2	25356970	F030	P030	/	/	Caucasian	Non-EA	NM_024531.4	c.652G>C	p.Ser218Leu		p.Leu339Pro	AR	M+M	homo	/	Ataxia	/
AP4569	SLC52A2	25133958	F031	P031	/	/	Caucasian	Non-EA	NM_024531.4	c.916G>A	p.Gly306Arg	c.916G>A	p.Gly306Arg	AR	M+M	homo	/	Ataxia	/
AP4570	SLC52A2	24616084	F032	P032	/	/	Caucasian	Non-EA	NM_024531.4	c.916G>A	p.Gly306Arg	c.916G>A	p.Gly306Arg	AR	M+M	homo	/	Ataxia	/
AP4571	SLC52A2	24616084	F033	P033	/	/	Caucasian	Non-EA	NM_024531.4	c.916G>A	p.Gly306Arg	c.916G>A	p.Gly306Arg	AR	M+M	homo	/	Ataxia	10
AP4572	SLC52A2	24616084	F034	P034	/	/	Caucasian	Non-EA	NM_024531.4	c.916G>A	p.Gly306Arg	c.916G>A	p.Gly306Arg	AR	M+M	homo	/	Ataxia	/
AP4573	SLC52A2	24616084	F035	P035	/	/	Caucasian	Non-EA	NM_024531.4	c.916G>A	p.Gly306Arg	c.916G>A	p.Gly306Arg	AR	M+M	homo	/	Ataxia	7
AP4574	SLC52A2	24616084	F036	P036	/	/	Caucasian	Non-EA	NM_024531.4	c.916G>A	p.Gly306Arg	c.916G>A	p.Gly306Arg	AR	M+M	homo	/	Ataxia	/
AP4575	SLC52A2	24253200	F037	P037	/	E1	Caucasian	Non-EA	NM_024531.4	c.916G>A	p.Gly306Arg	c.916G>A	p.Gly306Arg	AR	M+M	homo	Female	Childhood neuronopathy	/
AP4576	SLC52A2	24253200	F038	P038	/	E2	Caucasian	Non-EA	NM_024531.4	c.92G>C	p.Trp31Ser	c.935T>C	p.Leu312Pro	AR	M+M	compound I	Female	Childhood neuronopathy	/
AP4577	SLC52A2	24253200	F039	P039	/	E3	Caucasian	Non-EA	NM_024531.4	c.700C>T	p.Gln234*	c.1258G>A	p.Ala420Thr	AR	M+T	compound I	Female	Childhood neuronopathy	/
AP4578	SLC52A2	24253200	F040	P040	/	E4	Caucasian	Non-EA	NM_024531.4	c.916G>A	p.Gly306Arg	c.1016T>C	p.Leu339Pro	AR	M+M	compound I	Female	Childhood neuronopathy	/
AP4579	SLC52A2	24253200	F041	P041	/	E5	Caucasian	Non-EA	NM_024531.4	c.916G>A	p.Gly306Arg	c.1016T>C	p.Leu339Pro	AR	M+M	compound I	Female	Childhood neuronopathy	/
AP4580	SLC52A2	24253200	F042	P042	/	E6	Caucasian	Non-EA	NM_024531.4	c.935T>C	p.Leu312Pro	c.1016T>C	p.Leu339Pro	AR	M+M	compound I	Female	Childhood neuronopathy	/
AP4581	SLC52A2	24253200	F043	P043	/	E7	Caucasian	Non-EA	NM_024531.4	c.916G>A	p.Gly306Arg	c.1258G>A	p.Ala420Thr	AR	M+M	compound I	Male	Childhood neuronopathy	/
AP4582	SLC52A2	24253200	F044	P044	Family 1	A1	Caucasian	Non-EA	NM_024531.4	c.916G>A	p.Gly306Arg	c.916G>A	p.Gly306Arg	AR	M+M	homo	Female	Childhood neuronopathy	/
AP4583	SLC52A2	24253200	F044	P045	Family 1	A2	Caucasian	Non-EA	NM_024531.4	c.916G>A	p.Gly306Arg	c.916G>A	p.Gly306Arg	AR	M+M	homo	Female	Childhood neuronopathy	/
AP4584	SLC52A2	24253200	F045	P046	Family 2	A3	Caucasian	Non-EA	NM_024531.4	c.916G>A	p.Gly306Arg	c.1016T>C	p.Leu339Pro	AR	M+M	compound I	Female	Childhood neuronopathy	/
AP4585	SLC52A2	24253200	F045	P047	Family 2	A4	Caucasian	Non-EA	NM_024531.4	c.916G>A	p.Gly306Arg	c.1016T>C	p.Leu339Pro	AR	M+M	compound I	Female	Childhood neuronopathy	/
AP4586	SLC52A2	24253200	F046	P048	Family 3	A5	Caucasian	Non-EA	NM_024531.4	c.916G>A	p.Gly306Arg	c.916G>A	p.Gly306Arg	AR	M+M	homo	Male	Childhood neuronopathy	/
AP4587	SLC52A2	24253200	F046	P049	Family 3	A6	Caucasian	Non-EA	NM_024531.4	c.916G>A	p.Gly306Arg	c.916G>A	p.Gly306Arg	AR	M+M	homo	Male	Childhood neuronopathy	/
AP4588	SLC52A2	24253200	F046	P050	Family 3	A7	Caucasian	Non-EA	NM_024531.4	c.851C>A	p.Ala284Asp	c.916G>A	p.Gly306Arg	AR	M+M	compound I	Male	Childhood neuronopathy	/
AP4589	SLC52A2	24253200	F047	P051	Family 4	U1	Caucasian	Non-EA	NM_024531.4	c.851C>A	p.Ala284Asp	c.916G>A	p.Gly306Arg	AR	M+M	compound I	Female	Childhood neuronopathy	/
AP4590	SLC52A2	24253200	F047	P052	Family 4	U2	Caucasian	Non-EA	NM_024531.4	c.851C>A	p.Ala284Asp	c.916G>A	p.Gly306Arg	AR	M+M	compound I	Male	Childhood neuronopathy	/
AP4591	SLC52A2	24253200	F048	P053	/	I1	Caucasian	Non-EA	NM_024531.4	c.914A>G	p.Tyr305Cys	c.916G>A	p.Gly306Arg	AR	M+M	compound I	Male	Childhood neuronopathy	/
AP4592	SLC52A2	24253200	F049	P054	/	L1	Caucasian	Non-EA	NM_024531.4	c.916G>A	p.Gly306Arg	c.916G>A	p.Gly306Arg	AR	M+M	homo	Male	Childhood neuronopathy	/
AP4593	SLC52A2	23243084	F050	P055	/	/	Caucasian	Non-EA	NM_024531.4	c.155C>T	p.Ser52Phe	c.1255G>A	p.Gly419Ser	AR	M+M	compound I	Male	Brown-Vialetto-Van Laere syndron	/
AP4594	SLC52A2	22864630	F051	P056	/	/	Caucasian	Non-EA	NM_024531.4	c.368T>C	p.Leu123Pro	c.1016T>C	p.Leu339Pro	AR	M+M	compound I	Female	Brown-Vialetto-Van Laere syndron	3
AP4595	SLC52A2	22740598	F052	P057	/	/	Caucasian	Non-EA	NM_024531.4	c.916G>A	p.Gly306Arg	c.916G>A	p.Gly306Arg	AR	M+M	homo	/	Motor neuron disease	/
AP4596	SPG7	18563470	F001	P001	/	II-2	Caucasian	Non-EA	NM_003119.3	c.1047insC	p.Gly352Argfs*44	c.1047insC	p.Gly352Argfs	AR	T+T	homo	Female	Spastic paraplegia	/
AP4597	SPG7	18563470	F001	P002	/	II-4	Caucasian	Non-EA	NM_003119.3	c.1047insC	p.Gly352Argfs*44	c.1047insC	p.Gly352Argfs	AR	T+T	homo	Male	Spastic paraplegia	/
AP4598	SPG7	18563470	F001	P003	/	II-5	Caucasian	Non-EA	NM_003119.3	c.1047insC	p.Gly352Argfs*44	c.1047insC	p.Gly352Argfs	AR	T+T	homo	Male	Spastic paraplegia	/
AP4599	SPG7	18799786	F002	P004	/	patient 1	Caucasian	Non-EA	NM_003119.3	c.1454_1462del	p.Arg485_Glu48	c.1529C>T	p.Ala510Val	AR	M+M	compound I	Female	Spastic paraplegia	/
AP4600	SPG7	18799786	F003	P005	/	patient 2	Caucasian	Non-EA	NM_003119.3	c.1454_1462del	p.Arg485_Glu48	c.1529C>T	p.Ala510Val	AR	M+M	compound I	Female	Spastic paraplegia	/
AP4601	SPG7	18799786	F004	P006	/	patient 3	Caucasian	Non-EA	NM_003119.3	c.1454_1462del	p.Arg485_Glu48	c.1529C>T	p.Ala510Val	AR	M+M	compound I	Male	Spastic paraplegia	/
AP4602	SPG7	18799786	F005	P007	/	patient 4	Caucasian	Non-EA	NM_003119.3	c.1454_1462del	p.Arg485_Glu48	c.1454_1462d	p.Arg485_Gl	AR	M+M	homo	Male	Spastic paraplegia	/
AP4603	SPG7	18799786	F006	P008	/	patient 5	Caucasian	Non-EA	NM_003119.3	c.1529C>T	p.Ala510Val	c.2014G>A	p.Gly672Arg	AR	M+M	compound I	Female	Spastic paraplegia	/
AP4604	SPG7	18799786	F007	P009	/	patient 6	Caucasian	Non-EA	NM_003119.3	c.1454_1462del	p.Arg485_Glu48	c.2228T>C	p.Ile743Thr	AR	M+M	compound I	Male	Spastic paraplegia	/
AP4605	SPG7	18799786	F008	P010	/	patient 7	Caucasian	Non-EA	NM_003119.3	c.1045G>A	p.Gly349Ser	c.2069C>T	p.Pro690Leu	AR	M+M	compound I	Female	Spastic paraplegia	/
AP4606	SPG7	22571692	F009	P011	/	HSP36	Spanish	Non-EA	NM_003119.3	c.1061G>C	p.Gly354Ala	c.1715C>T	p.Ala572Val	AR	M+M	compound I	/	Spastic paraplegia	/
AP4607	SPG7	22571692	F010	P012	/	HSP40	Spanish	Non-EA	NM_003119.3	c.1987A>T	p.Lys663*	/	/	AD	Truncation	heter	/	Spastic paraplegia	/
AP4608	SPG7	22571692	F011	P013	/	HSP48	Spanish	Non-EA	NM_003119.3	c.661A>T	p.Lys221*	/	/	AD	Truncation	heter	/	Spastic paraplegia	/
AP4609	SPG7	22571692	F012	P014	/	HSP173	Spanish	Non-EA	NM_003119.3	c.1198C>T	p.Arg400Trp	/	/	AD	M	heter	/	Spastic paraplegia	/
AP4610	SPG7	22571692	F013	P015	/	HSP3244	Spanish	Non-EA	NM_003119.3	c.1044_1045insGCT	p.Leu348_Gly349	/	/	AD	M	heter	/	Spastic paraplegia	/
AP4611	SPG7	22571692	F014	P016	/	HSP260	Spanish	Non-EA	NM_003119.3	c.1645G>A	p.Val549Met	/	/	AD	M	heter	/	Spastic paraplegia	/
AP4612	SPG7	22571692	F015	P017	/	HSP296	Spanish	Non-EA	NM_003119.3	c.233T>A	p.Leu78*	/	/	AD	Truncation	heter	/	Spastic paraplegia	/
AP4613	SPG7	22571692	F016	P018	/	HSP312	Spanish	Non-EA	NM_003119.3	c.773_774delTG	p.Val258Glyfs*30	/	/	AD	Truncation	heter	/	Spastic paraplegia	/
AP4614	SPG7	22571692	F017	P019	/	HSP370	Spanish	Non-EA	NM_003119.3	c.1-?_757+?del	p.Met1_Asn253del	/	/	AD	Truncation	heter	/	Spastic paraplegia	/
AP4615	SPG7	22571692	F018	P020	/	HSP416	Spanish	Non-EA	NM_003119.3	c.376G>C	p.Glu126Argfs*5	/	/	AD	Truncation	heter	/	Spastic paraplegia	/
AP4616	SPG7	22571692	F019	P021	/	HSP240	Spanish	Non-EA	NM_003119.3	c.376G>C	p.Glu126Argfs*5	c.1529C>T	p.Ala510Val	AR	M+T	compound I	/	Spastic paraplegia	/
AP4617	SPG7	22571692	F020	P022	/	HSP365	Spanish	Non-EA	NM_003119.3	c.1982T>C	p.Met661Thr	c.1529C>T	p.Ala510Val	AR	M+M	compound I	/	Spastic paraplegia	/
AP4618	SPG7	22571692	F021	P023	/	HSP402	Spanish	Non-EA	NM_003119.3	c.184-?_1324+?del	p.Ser62_Asp441de	c.1529C>T	p.Ala510Val	AR	M+T	compound I	/	Spastic paraplegia	/
AP4619	SPG7	22571692	F022	P024	/	HSP503	Spanish	Non-EA	NM_003119.3	c.1777A>T	p.Lys593*	c.1529C>T	p.Ala510Val	AR	M+T	compound I	/	Spastic paraplegia	/
AP4620	SPG7	22964162	F027	P029	Family 1	1	Dutch	Non-EA	NM_003119.3	c.1409G>A	p.Arg470Gln	c.1409G>A	p.Arg470Gln	AR	M+M	homo	Female	Spastic paraplegia	/
AP4621	SPG7	22964162	F027	P030	Family 1	2	Dutch	Non-EA	NM_003119.3	c.1409G>A	p.Arg470Gln	c.1409G>A	p.Arg470Gln	AR	M+M	homo	Female	Spastic paraplegia	/
AP4622	SPG7	22964162	F028	P031	Family 2	3	Dutch	Non-EA	NM_003119.3	c.1454_1462delGGCGI	p.Arg485_Glu487	c.1454_1462d	p.Arg485_Glu	AR	M+M	homo	Female	Spastic paraplegia	/
AP4623	SPG7	22964162	F028	P032	Family 2	4	Dutch	Non-EA	NM_003119.3	c.1454_1462delGGCGI	p.Arg485_Glu487	c.1454_1462d	p.Arg485_Glu	AR	M+M	homo	Female	Spastic paraplegia	/
AP4624	SPG7	22964162	F029	P033	Family 3	5	Dutch	Non-EA	NM_003119.3	c.1454_1462delGGCGI	p.Arg485_Glu487	c.1454_1462d	p.Arg485_Glu	AR	M+M	homo	Male	Spastic paraplegia	/
AP4625	SPG7	22964162	F029	P034	Family 3	6	Dutch	Non-EA	NM_003119.3	c.1454_1462delGGCGI	p.Arg485_Glu487	c.1454_1462d	p.Arg485_Glu	AR	M+M	homo	Male	Spastic paraplegia	/
AP4626	SPG7	22964162	F030	P035	Family 4	7	Dutch	Non-EA	NM_003119.3	c.1454_1462delGGCGI	p.Arg485_Glu487	c.1672A>T	p.Lys558*	AR	M+T	compound I	Female	Spastic paraplegia	/
AP4627	SPG7	22964162	F030	P036	Family 4	8	Dutch	Non-EA	NM_003119.3	c.1454_1462delGGCGI	p.Arg485_Glu487	c.1672A>T	p.Lys558*	AR	M+T	compound I	Male	Spastic paraplegia	/
AP4628	SPG7	22964162	F031	P037	Family 5	9	Dutch	Non-EA	NM_003119.3	c.1454_1462delGGCGI	p.Arg485_Glu487	c.2115_2131d	p.Leu706fs	AR	M+T	compound I	Female	Spastic paraplegia	/
AP4629	SPG7	22964162	F031	P038	Family 5	10	Dutch	Non-EA	NM_003119.3	c.1454_1462delGGCGI	p.Arg485_Glu487	c.2115_2131d	p.Leu706fs	AR	M+T	compound I	Male	Spastic paraplegia	/
AP4630	SPG7	22964162	F032	P039	Family 6	11	Dutch	Non-EA	NM_003119.3	c.1529C>T	p.Ala510Val	c.1454_1462d	p.Arg485_Glu	AR	M+M	compound I	Male	Spastic paraplegia	/
AP4631	SPG7	22964162	F032	P040	Family 6	12	Dutch	Non-EA	NM_003119.3	c.1529C>T	p.Ala510Val	c.1454_1462d	p.Arg485_Glu	AR	M+M	compound I	Male	Spastic paraplegia	/
AP4632	SPG7	22964162	F033	P041	Family 7	13	Dutch	Non-EA	NM_003119.3	c.1529C>T	p.Ala510Val	c.1529C>T	p.Ala510Val	AR	M+M	homo	Male	Spastic paraplegia	/
AP4633	SPG7	22964162	F033	P042	Family 7	14	Dutch	Non-EA	NM_003119.3	c.1529C>T	p.Ala510Val	c.1529C>T	p.Ala510Val	AR	M+M	homo	Male	Spastic paraplegia	/
AP4634	SPG7	22964162	F034	P043	Family 8	15	Dutch	Non-EA	NM_003119.3	c.1529C>T	p.Ala510Val	c.1937-2del	p.?	AR	M+T	compound I	Female	Spastic paraplegia	/
AP4635	SPG7	22964162	F034	P044	Family 8	16	Dutch	Non-EA	NM_003119.3	c.1529C>T	p.Ala510Val	c.1937-2del	p.?	AR	M+T	compound I	Female	Spastic paraplegia	/
AP4636	SPG7	22964162	F035	P045	Family 9	17	Dutch	Non-EA	NM_003119.3	c.1529C>T	p.Ala510Val	c.861+2dup	p.?	AR	M+T	compound I	Female	Spastic paraplegia	/
AP4637	SPG7	22964162	F035	P046	Family 9	18	Dutch	Non-EA	NM_003119.3	c.1529C>T	p.Ala510Val	c.861+2dup	p.?	AR	M+T	compound I	Male	Spastic paraplegia	/
AP4638	SPG7	22964162	F036	P047	Family 10	19	Dutch	Non-EA	NM_003119.3	c.861dup	p.Asn288*	c.2228T>C	p.Ile743Thr	AR	M+T	compound I	Male	Spastic paraplegia	/
AP4639	SPG7	22964162	F036	P048	Family 10	20	Dutch	Non-EA	NM_003119.3	c.861dup	p.Asn288*	c.2228T>C	p.Ile743Thr	AR	M+T	compound I	Female	Spastic paraplegia	/
AP4640	SPG7	22964162	F037	P049	Family 11	21	Dutch	Non-EA	NM_003119.3	c.861dup	p.Asn288*	c.861dup	p.Asn288*	AR	T+T	homo	Female	Spastic paraplegia	/
AP4641	SPG7	22964162	F037	P050	Family 11	22	Dutch	Non-EA	NM_003119.3	c.861dup	p.Asn288*	c.861dup	p.Asn288*	AR	T+T	homo	Female	Spastic paraplegia	/
AP4642	SPG7	22964162	F038	P051	Family 12	23	Dutch	Non-EA	NM_003119.3	c.1529C>T	p.Ala510Val	c.1045G>A	p.Gly349Ser	AR	M+M	compound I	Male	Spastic paraplegia	/
AP4643	SPG7	22964162	F038	P052	Family 12	24	Dutch	Non-EA	NM_003119.3	c.1529C>T	p.Ala510Val	c.1529C>T	p.Ala510Val	AR	M+M	homo	Male	Spastic paraplegia	/
AP4644	SPG7	22964162	F039	P053	Family 13	25	Dutch	Non-EA	NM_003119.3	c.1529C>T	p.Ala510Val	c.1529C>T	p.Ala510Val	AR	M+M	homo	Male	Spastic paraplegia	/
AP4645	SPG7	22964162	F039	P054	Family 13	26	Dutch	Non-EA	NM_003119.3	c.1529C>T	p.Ala510Val	c.1529C>T	p.Ala510Val	AR	M+M	homo	Female	Spastic paraplegia	/
AP4646	SPG7	22964162	F039	P055	Family 13	27	Dutch	Non-EA	NM_003119.3	c.1529C>T	p.Ala510Val	c.1529C>T	p.Ala510Val	AR	M+M	homo	Female	Spastic paraplegia	/
AP4647	SPG7	22964162	F040	P056	/	28	Dutch	Non-EA	NM_003119.3	c.1045G>A	p.Gly349Ser	c.2069C>T	p.Pro690Leu	AR	M+M	compound I	Female	Spastic paraplegia	/
AP4648	SPG7	22964162	F041	P057	/	29	Dutch	Non-EA	NM_003119.3	c.1045G>A	p.Gly349Ser	c.2090A>C	p.Gln697Pro	AR	M+M	compound I	Male	Spastic paraplegia	/
AP4649	SPG7	22964162	F042	P058	/	30	Dutch	Non-EA	NM_003119.3	c.1045G>A	p.Gly349Ser	c.2115_2131d	p.Leu706fs	AR	M+T	compound I	Female	Spastic paraplegia	/
AP4650	SPG7	22964162	F043	P059	/	31	Dutch	Non-EA	NM_003119.3	c.1147G>T	p.Gly383*	c.1822C>T	p.Gln608*	AR	T+T	compound I	Female	Spastic paraplegia	/
AP4651	SPG7	22964162	F044	P060	/	32	Dutch	Non-EA	NM_003119.3	c.1454_1462delGGCGI	p.Arg485_Glu487	c.2228T>C	p.Ile743Thr	AR	M+M	compound I	Male	Spastic paraplegia	/
AP4652	SPG7	22964162	F045	P061	/	33	Dutch	Non-EA	NM_003119.3	c.1454_1462delGGCGI	p.Arg485_Glu487del			AD	M	heter	Male	Spastic paraplegia	/
AP4653	SPG7	22964162	F046	P062	/	34	Dutch	Non-EA	NM_003119.3	c.1529C>T	p.Ala510Val	c.1454_1462d	p.Arg485_Glu	AR	M+M	compound I	Female	Spastic paraplegia	/
AP4654	SPG7	22964162	F047	P063	/	35	Dutch	Non-EA	NM_003119.3	c.1529C>T	p.Ala510Val	c.1454_1462d	p.Arg485_Glu	AR	M+M	compound I	Male	Spastic paraplegia	/
AP4655	SPG7	22964162	F048	P064	/	36	Dutch	Non-EA	NM_003119.3	c.1529C>T	p.Ala510Val	c.1454_1462d	p.Arg485_Glu	AR	M+M	compound I	Female	Spastic paraplegia	/
AP4656	SPG7	22964162	F049	P065	/	37	Dutch	Non-EA	NM_003119.3	c.1529C>T	p.Ala510Val	c.1454_1462d	p.Arg485_Glu	AR	M+M	compound I	Male	Spastic paraplegia	/
AP4657	SPG7	22964162	F050	P066	/	38	Dutch	Non-EA	NM_003119.3	c.1529C>T	p.Ala510Val	c.1454_1462d	p.Arg485_Glu	AR	M+M	compound I	Male	Spastic paraplegia	/
AP4658	SPG7	22964162	F051	P067	/	39	Dutch	Non-EA	NM_003119.3	c.1529C>T	p.Ala510Val	c.1454_1462d	p.Arg485_Glu	AR	M+M	compound I	Female	Spastic paraplegia	/
AP4659	SPG7	22964162	F052	P068	/	40	Dutch	Non-EA	NM_003119.3	c.1529C>T	p.Ala510Val	c.1529C>T	p.Ala510Val	AR	M+M	homo	Male	Spastic paraplegia	/
AP4660	SPG7	22964162	F053	P069	/	41	Dutch	Non-EA	NM_003119.3	c.1529C>T	p.Ala510Val	c.1529C>T	p.Ala510Val	AR	M+M	homo	Female	Spastic paraplegia	/
AP4661	SPG7	22964162	F054	P070	/	42	Dutch	Non-EA	NM_003119.3	c.1529C>T	p.Ala510Val	c.1529C>T	p.Ala510Val	AR	M+M	homo	Male	Spastic paraplegia	/
AP4662	SPG7	22964162	F055	P071	/	43	Dutch	Non-EA	NM_003119.3	c.1529C>T	p.Ala510Val	c.1529C>T	p.Ala510Val	AR	M+M	homo	Male	Spastic paraplegia	/
AP4663	SPG7	22964162	F056	P072	/	44	Dutch	Non-EA	NM_003119.3	c.1529C>T	p.Ala510Val	c.2014G>A	p.Gly672Arg	AR	M+M	compound I	Female	Spastic paraplegia	/
AP4664	SPG7	22964162	F057	P073	/	45	Dutch	Non-EA	NM_003119.3	c.1529C>T	p.Ala510Val	c.2115_2131d	p.Leu706fs	AR	M+T	compound I	Male	Spastic paraplegia	/
AP4665	SPG7	22964162	F058	P074	/	46	Dutch	Non-EA	NM_003119.3	c.1529C>T	p.Ala510Val	c.2115_2131d	p.Leu706fs	AR	M+T	compound I	Female	Spastic paraplegia	/
AP4666	SPG7	22964162	F059	P075	/	47	Dutch	Non-EA	NM_003119.3	c.1529C>T	p.Ala510Val	c.2115_2131d	p.Leu706fs	AR	M+T	compound I	Male	Spastic paraplegia	/
AP4667	SPG7	22964162	F060	P076	/	48	Dutch	Non-EA	NM_003119.3	c.1529C>T	p.Ala510Val	c.759-?_2181+?del	p.?	AR	M+T	compound I	Male	Spastic paraplegia	/
AP4668	SPG7	22964162	F061	P077	/	49	Dutch	Non-EA	NM_003119.3	c.1529C>T	p.Ala510Val	c.759-?_2181+?del	p.?	AR	M+T	compound I	Female	Spastic paraplegia	/
AP4669	SPG7	22964162	F062	P078	/	50	Dutch	Non-EA	NM_003119.3	c.1672A>T	p.Lys558*	c.1672A>T	p.Lys558*	AR	T+T	compound I	Male	Spastic paraplegia	/
AP4670	SPG7	22964162	F063	P079	/	51	Dutch	Non-EA	NM_003119.3	c.1894G>A	p.Gly632Arg	c.1984delinsT	p.Val662fs	AR	M+T	compound I	Male	Spastic paraplegia	/
AP4671	SPG7	22964162	F064	P080	/	52	Dutch	Non-EA	NM_003119.3	c.2115_2131del	p.Leu706fs	c.2115_2131d	p.Leu706fs	AR	T+T	homo	Female	Spastic paraplegia	/
AP4672	SPG7	22964162	F065	P081	/	53	Dutch	Non-EA	NM_003119.3	c.2115_2131del	p.Leu706fs	c.2115_2131d	p.Leu706fs	AR	T+T	homo	Female	Spastic paraplegia	/
AP4673	SPG7	22964162	F066	P082	/	54	Dutch	Non-EA	NM_003119.3	c.3G>A	p.?			AD	M	heter	Female	Spastic paraplegia	/
AP4674	SPG7	22964162	F067	P083	/	55	Dutch	Non-EA	NM_003119.3	c.618+11_618+68del	p.?			AD	Truncation	heter	Male	Spastic paraplegia	/
AP4675	SPG7	22964162	F068	P084	/	56	Dutch	Non-EA	NM_003119.3	c.1045G>A	p.Gly349Ser			AD	M	heter	Male	Spastic paraplegia	/
AP4676	SPG7	22964162	F069	P085	/	57	Dutch	Non-EA	NM_003119.3	c.1529C>T	p.Ala510Val	c.1045G>A	p.Gly349Ser	AR	M+M	compound I	Male	Spastic paraplegia	/
AP4677	SPG7	22964162	F070	P086	/	58	Dutch	Non-EA	NM_003119.3	c.1529C>T	p.Ala510Val	c.1045G>A	p.Gly349Ser	AR	M+M	compound I	Male	Spastic paraplegia	/
AP4678	SPG7	22964162	F071	P087	/	59	Dutch	Non-EA	NM_003119.3	c.1529C>T	p.Ala510Val	c.1053dup	p.Gly352Argfs	AR	M+T	compound I	Female	Spastic paraplegia	/
AP4679	SPG7	22964162	F072	P088	/	60	Dutch	Non-EA	NM_003119.3	c.1529C>T	p.Ala510Val	c.1454_1462d	p.Arg485_Glu	AR	M+M	compound I	Male	Spastic paraplegia	/
AP4680	SPG7	23065789	F073	P089	FSP-554	5	French	Non-EA	NM_003119.3	c.1454_1462delGGCGI	p.Arg485_Glu487	c.1454_1462d	p.Arg485_Glu	AR	M+M	homo	Female	Spastic paraplegia	/
AP4681	SPG7	23065789	F074	P090	SAL-640	6	French	Non-EA	NM_003119.3	c.1454_1462delGGCGI	p.Arg485_Glu487	c.1529C>T	p.Ala510Val	AR	M+M	compound I	Male	Spastic paraplegia	/
AP4682	SPG7	23065789	F075	P091	FSP-171	7	French	Non-EA	NM_003119.3	c.1A>T	p.Met1?	c.850_851deli	p.Phe284Profs	AR	M+T	compound I	Male	Spastic paraplegia	/
AP4683	SPG7	23065789	F076	P092	AAD-08	8	French	Non-EA	NM_003119.3	c.1454_1462delGGCGI	p.Arg485_Glu487	c.1529C>T	p.Ala510Val	AR	M+M	compound I	Female	Spastic paraplegia	/
AP4684	SPG7	23065789	F077	P093	FSP-159	9	French	Non-EA	NM_003119.3	c.233T>A	p.Leu78*	c.1454_1462d	p.Arg485_Glu	AR	M+T	compound I	Male	Spastic paraplegia	/
AP4685	SPG7	23065789	F078	P094	/	10	French	Non-EA	NM_003119.3	c.1-?_286+?	p.0 ?	c.1529C>T	p.Ala510Val	AR	M+T	compound I	Female	Spastic paraplegia	/
AP4686	SPG7	23065789	F079	P095	FSP-1099	11	French	Non-EA	NM_003119.3	c.233T>A	p.Leu78*	c.233T>A	p.Leu78*	AR	T+T	homo	Female	Spastic paraplegia	/
AP4687	SPG7	23065789	F080	P096	399-598	12	Moroccan	Non-EA	NM_003119.3	c.618+3G>C	p.?	c.2240T>C	p.Ile747Thr	AR	M+T	compound I	Male	Spastic paraplegia	/
AP4688	SPG7	23065789	F081	P097	FSP-796	13	French	Non-EA	NM_003119.3	c.1454_1462delGGCGI	p.Arg485_Glu487	c.1529C>T	p.Ala510Val	AR	M+M	compound I	Male	Spastic paraplegia	/
AP4689	SPG7	23065789	F082	P098	FSP-476	14	French	Non-EA	NM_003119.3	c.1049_1077del	p.Pro350GlnfsX3	c.1529C>T	p.Ala510Val	AR	M+T	compound I	Female	Spastic paraplegia	/
AP4690	SPG7	23065789	F083	P099	FSP-659	15	French	Non-EA	NM_003119.3	c.2216dupA	p.Asp739LysfsX3	c.2216dupA	p.Asp739Lysfs	AR	T+T	homo	Male	Spastic paraplegia	/
AP4691	SPG7	23065789	F084	P100	FSP-859	16	French	Non-EA	NM_003119.3	c.1053dup	p.Gly352ArgfsX44	c.1529C>T	p.Ala510Val	AR	M+T	compound I	Male	Spastic paraplegia	/
AP4692	SPG7	23065789	F085	P101	/	17	French	Non-EA	NM_003119.3	c.376 + 1G>T	p. ?	c.1045G>A	p.Gly349Ser	AR	M+T	compound I	Female	Spastic paraplegia	/
AP4693	SPG7	23065789	F086	P102	/	18	French	Non-EA	NM_003119.3	c.1529C>T	p.Ala510Val	c.1715C>T	p.Ala572Val	AR	M+M	compound I	Female	Spastic paraplegia	/
AP4694	SPG7	23065789	F087	P103	/	19	French	Non-EA	NM_003119.3	c.1529C>T	p.Ala510Val	c.2249C>T	p.Pro750Leu	AR	M+M	compound I	Male	Spastic paraplegia	/
AP4695	SPG7	23065789	F088	P104	/	20	British	Non-EA	NM_003119.3	c.1053dup	p.Gly352ArgfsX44	c.1529C>T	p.Ala510Val	AR	M+T	compound I	Female	Spastic paraplegia	/
AP4696	SPG7	23065789	F089	P105	N 08 0132	21	French	Non-EA	NM_003119.3	c.1529C>T	p.Ala510Val	c.2249C>T	p.Pro750Leu	AR	M+M	compound I	Female	Spastic paraplegia	/
AP4697	SPG7	23065789	F090	P106	N 10 0935	22	French	Non-EA	NM_003119.3	c.1529C>T	p.Ala510Val	c.1369C>T	p.Arg457*	AR	M+T	compound I	Male	Spastic paraplegia	/
AP4698	SPG7	23065789	F091	P107	N 10 0094	23	French	Non-EA	NM_003119.3	c.861dup	p.Asn288*	c.1529C>T	p.Ala510Val	AR	M+T	compound I	Female	Spastic paraplegia	/
AP4699	SPG7	23268439	F095	P111	/	Individual 1	British	Non-EA	NM_003119.3	c.1529C>T	p.Ala510Val	c.1529C>T	p.Ala510Val	AR	M+M	homo	/	Spastic paraplegia	/
AP4700	SPG7	23268439	F096	P112	/	Individual 2	British	Non-EA	NM_003119.3	c.1529C>T	p.Ala510Val	c.1046insC	p.Gly352fs*44	AR	M+T	compound I	/	Spastic paraplegia	/
AP4701	SPG7	23268439	F097	P113	/	Individual 3	British	Non-EA	NM_003119.3	c.1529C>T	p.Ala510Val	c.1454_1462d	p.Arg485_Glu	AR	M+M	compound I	/	Spastic paraplegia	/
AP4702	SPG7	23268439	F098	P114	/	Individual 4	British	Non-EA	NM_003119.3	c.1796G>T	p. Arg599Leu			AD	M	heter	/	Spastic paraplegia	/
AP4703	SPG7	23268439	F099	P115	Family 1	1	British	Non-EA	NM_003119.3	c.1529C>T	p.Ala510Val	c.1529C>T	p.Ala510Val	AR	M+M	homo	/	Spastic paraplegia	/
AP4704	SPG7	23268439	F099	P116	Family 1	2	British	Non-EA	NM_003119.3	c.1529C>T	p.Ala510Val	c.1529C>T	p.Ala510Val	AR	M+M	homo	/	Spastic paraplegia	/
AP4705	SPG7	23268439	F099	P117	Family 1	3	British	Non-EA	NM_003119.3	c.1529C>T	p.Ala510Val	c.1529C>T	p.Ala510Val	AR	M+M	homo	/	Spastic paraplegia	/
AP4706	SPG7	23268439	F100	P118	Family 2	Father	British	Non-EA	NM_003119.3	c.1045G>A	p.Gly349Ser	c.1529C>T	p.Ala510Val	AR	M+M	compound I	/	Spastic paraplegia	/
AP4707	SPG7	23268439	F100	P119	Family 2	Son	British	Non-EA	NM_003119.3	c.1529C>T	p.Ala510Val	c.1529C>T	p.Ala510Val	AR	M+M	homo	/	Spastic paraplegia	/
AP4708	SPG7	23733235	F101	P120	2		Caucasian	Non-EA	NM_003119.3	c.415C > T	p.Arg139*	c.1053dupC	p.Gly352Argfs	AR	T+T	compound I	/	Spastic paraplegia	/
AP4709	SPG7	23733235	F102	P121	11		Caucasian	Non-EA	NM_003119.3	c.1529C>T	p.Ala510Val	c.1529C>T	p.Ala510Val	AR	M+M	homo	/	Spastic paraplegia	/
AP4710	SPG7	23733235	F103	P122	13		Caucasian	Non-EA	NM_003119.3	c.1529C>T	p.Ala510Val	c.861+6T>C	p.?	AR	M+T	compound I	/	Spastic paraplegia	/
AP4711	SPG7	23733235	F104	P123	24		Caucasian	Non-EA	NM_003119.3	c.1045G>A	p.Gly349Ser	c.1996G>C	p.Gly666Arg	AR	M+M	compound I	/	Spastic paraplegia	/
AP4712	SPG7	23733235	F105	P124	27		Caucasian	Non-EA	NM_003119.3	c.759-1G>T	?	c.1450-1_1457	p.?	AR	T+T	compound I	/	Spastic paraplegia	/
AP4713	SPG7	23733235	F106	P125	30		Caucasian	Non-EA	NM_003119.3	c.1529C>T	p.Ala510Val	c.4delG	p.Ala2fsX64	AR	M+T	compound I	/	Spastic paraplegia	/
AP4714	SPG7	23733235	F107	P126	31		Caucasian	Non-EA	NM_003119.3	del exon 6		del exon 6	p.?	AR	CNV+CNV	homo	/	Spastic paraplegia	/
AP4715	SPG7	23733235	F108	P127	44		Caucasian	Non-EA	NM_003119.3	del exon 6		del exon 6	p.?	AR	CNV+CNV	homo	/	Spastic paraplegia	/
AP4716	SPG7	23733235	F109	P128	45		Caucasian	Non-EA	NM_003119.3	c.1996G>C	p.Gly666Arg			AD	M	heter	/	Spastic paraplegia	/
AP4717	SPG7	23733235	F110	P129	24		Caucasian	Non-EA	NM_003119.3	c.1045G>A	p.Gly349Ser	c.1996G>C	p.Gly666Arg	AR	M+M	compound I	/	Spastic paraplegia	/
AP4718	SPG7	23812641	F111	P130	/	L-6655	European	Non-EA	NM_003119.3	c.1045G>A	p.Gly349Ser	c.1454_1462d	p.Arg485_Glu	AR	M+M	compound I	Male	Spastic paraplegia	/
AP4719	SPG7	23812641	F112	P131	/	L-6709	European	Non-EA	NM_003119.3	c.1529C>T	p.Ala510Val	c.1454_1462d	p.Arg485_Glu	AR	M+M	compound I	Male	Spastic paraplegia	/
AP4720	SPG7	23812641	F113	P132	/	P26108	European	Non-EA	NM_003119.3	c.1529C>T	p.Ala510Val	c.1454_1462d	p.Arg485_Glu	AR	M+M	compound I	Male	Spastic paraplegia	/
AP4721	SPG7	23812641	F114	P133	/	L-7632'	European	Non-EA	NM_003119.3	c.1529C>T	p.Ala510Val	c.1727C>G	p.Ser576Trp	AR	M+M	compound I	Male	Spastic paraplegia	/
AP4722	SPG7	24466038	F115	P134	/	Patient AIV-5	Norwegian	Non-EA	NM_003119.3	c.2102A>C	p.His701Pro	c.2102A>C	p. His701Pro	AR	M+M	homo	Female	Spastic paraplegia	/
AP4723	SPG7	24466038	F115	P135	/	Patient AIV-2	Norwegian	Non-EA	NM_003119.3	c.2102A>C	p.His701Pro	c.2102A>C	p. His701Pro	AR	M+M	homo	Male	Spastic paraplegia	/
AP4724	SPG7	24466038	F116	P136	/	Patient BIII-2	Norwegian	Non-EA	NM_003119.3	c.2102A>C	p.His701Pro	c.1454_1462d	p.Arg485_Glu	AR	M+M	compound I	Female	Spastic paraplegia	/
AP4725	SPG7	24466038	F116	P137	/	Patient BIII-5	Norwegian	Non-EA	NM_003119.3	c.2102A>C	p.His701Pro	c.1454_1462d	p.Arg485_Glu	AR	M+M	compound I	Male	Spastic paraplegia	/
AP4726	SPG7	24727571	F117	P138	/	1	Caucasian	Non-EA	NM_003119.3	c.861dup	p.Asn288*	c.1672A>T	p.Lys558*	AR	T+T	compound I	Male	Spastic paraplegia	/
AP4727	SPG7	24727571	F118	P139	/	2	Caucasian	Non-EA	NM_003119.3	c.1192C>T	p.Arg398*	c.1529C>T	p.Ala510Val	AR	M+T	compound I	Male	Spastic paraplegia	/
AP4728	SPG7	24727571	F119	P140	/	3	Caucasian	Non-EA	NM_003119.3	c.1529C>T	p.Ala510Val	c.1672A>T	p.Lys558*	AR	M+T	compound I	Male	Spastic paraplegia	/
AP4729	SPG7	24727571	F120	P141	/	4	Caucasian	Non-EA	NM_003119.3	c.2221G>A	p.Glu741Lys	c.2224G>A	p.Asp742Asn	AR	M+M	compound I	Female	Spastic paraplegia	/
AP4730	SPG7	24727571	F121	P142	/	5	Caucasian	Non-EA	NM_003119.3	c.1053dup	p.Gly352 Argfs*44	c.1529C>T	p.Ala510Val	AR	M+T	compound I	Male	Spastic paraplegia	/
AP4731	SPG7	24727571	F122	P143	/	6	Caucasian	Non-EA	NM_003119.3	c.1454_1462del	p.Arg485_Glu487	c.2228T>C	p.Ile743Thr	AR	M+M	compound I	Male	Spastic paraplegia	/
AP4732	SPG7	24727571	F123	P144	/	7	Caucasian	Non-EA	NM_003119.3	c.233T>A	p.Leu78*	c.1529C>T	p.Ala510Val	AR	M+T	compound I	Female	Spastic paraplegia	/
AP4733	SPG7	24727571	F124	P145	/	8	Caucasian	Non-EA	NM_003119.3	c.1192C>T	p.Arg398*	c.1529C>T	p.Ala510Val	AR	M+T	compound I	Male	Spastic paraplegia	/
AP4734	SPG7	24727571	F125	P146	/	9	Caucasian	Non-EA	NM_003119.3	c.1046insC	p.Gly352fs*44	c.1529C>T	p.Ala510Val	AR	M+T	compound I	Male	Spastic paraplegia	/
AP4735	SPG7	24727571	F126	P147	/	10	Caucasian	Non-EA	NM_003119.3	c.184-3C>T	p.?	c.1457G>A	p.Arg486Gln	AR	M+T	compound I	Female	Spastic paraplegia	/
AP4736	SPG7	24727571	F127	P148	/	11	Caucasian	Non-EA	NM_003119.3	c.1529C>T	p.Ala510Val	c.1529C>T	p.Ala510Val	AR	M+M	homo	Male	Spastic paraplegia	/
AP4737	SPG7	24727571	F128	P149	/	12	Caucasian	Non-EA	NM_003119.3	c.1529C>T	p.Ala510Val	c.1529C>T	p.Ala510Val	AR	M+M	homo	Female	Spastic paraplegia	/
AP4738	SPG7	24727571	F129	P150	/	13	Caucasian	Non-EA	NM_003119.3	c.1529C>T	p.Ala510Val	c.1529C>T	p.Ala510Val	AR	M+M	homo	Male	Spastic paraplegia	/
AP4739	SPG7	24727571	F130	P151	/	14	Caucasian	Non-EA	NM_003119.3	c.1067C>T	p.Thr356Met	c.1067C>T	p.Thr356Met	AR	M+M	homo	Male	Spastic paraplegia	/
AP4740	SPG7	24727571	F131	P152	/	15	Caucasian	Non-EA	NM_003119.3	c.233T>A	p.Leu78*	c.233T>A	p.Leu78*	AR	T+T	homo	Female	Spastic paraplegia	/
AP4741	SPG7	24731568	F132	P153	/	14	Italian	Non-EA	NM_003119.3	del exons 13–17		c.1529C>T	p.Ala510Val	AR	M+CNV	compound I	Male	Spastic paraplegia	/
AP4742	SPG7	24731568	F133	P154	/	15	Italian	Non-EA	NM_003119.3	c.1529C>T	p.Ala510Val	c.2216dup	p.Asn739Lysfs	AR	M+T	compound I	Female	Spastic paraplegia	/
AP4743	SPG7	24731568	F134	P155	/	16	Italian	Non-EA	NM_003119.3	c.233T>A	p.Leu78*	c.233T>A	p.Leu78*	AR	T+T	homo	Male	Spastic paraplegia	/
AP4744	SPG7	24731568	F135	P156	/	17	Italian	Non-EA	NM_003119.3	c.1617delC	p.Ser539 *	c.1617delC	p.Ser539 *	AR	T+T	homo	Male	Spastic paraplegia	/
AP4745	SPG7	25497598	F136	P157	/	P9	European	Non-EA	NM_003119.3	c.1529C>T	p.Ala510Val	c.1715C>T	p.Ala572Val	AR	M+M	compound I	Male	Spastic paraplegia	/
AP4746	SPG7	25497598	F136	P158	/	P10	European	Non-EA	NM_003119.3	c.1529C>T	p.Ala510Val	c.1715C>T	p.Ala572Val	AR	M+M	compound I	Female	Spastic paraplegia	/
AP4747	SPG7	25681447	F137	P159	/	1	Caucasian	Non-EA	NM_003119.3	c.1529C>T	p.Ala510Val	c.1715C>T	p.Ala572Val	AR	M+M	compound I	/	Spastic paraplegia	/
AP4748	SPG7	25681447	F137	P160	/	2	Caucasian	Non-EA	NM_003119.3	c.1529C>T	p.Ala510Val	c.1715C>T	p.Ala572Val	AR	M+M	compound I	/	Spastic paraplegia	/
AP4749	SPG7	25681447	F138	P161	/	3	Caucasian	Non-EA	NM_003119.3	c.1529C>T	p.Ala510Val	c.1192C>T	p.Arg398*	AR	M+T	compound I	/	Spastic paraplegia	/
AP4750	SPG7	25681447	F138	P162	/	4	Caucasian	Non-EA	NM_003119.3	c.1529C>T	p.Ala510Val	c.1192C>T	p.Arg398*	AR	M+T	compound I	/	Spastic paraplegia	/
AP4751	SPG7	25681447	F139	P163	/	5	Caucasian	Non-EA	NM_003119.3	c.1529C>T	p.Ala510Val	c.233T>A	p.Leu78*	AR	M+T	compound I	/	Spastic paraplegia	/
AP4752	SPG7	25681447	F140	P164	/	6	Caucasian	Non-EA	NM_003119.3	c.1529C>T	p.Ala510Val	c.1715C>T	p.Ala572Val	AR	M+M	compound I	/	Spastic paraplegia	/
AP4753	SPG7	25681447	F141	P165	/	7	Caucasian	Non-EA	NM_003119.3	c.1529C>T	p.Ala510Val	c.1715C>T	p.Ala572Val	AR	M+M	compound I	/	Spastic paraplegia	/
AP4754	SPG7	25681447	F142	P166	/	8	Caucasian	Non-EA	NM_003119.3	c.1529C>T	p.Ala510Val	c.1529C>T	p.Ala510Val	AR	M+M	homo	/	Spastic paraplegia	/
AP4755	SPG7	25681447	F143	P167	/	9	Caucasian	Non-EA	NM_003119.3	c.1529C>T	p.Ala510Val	c.2228T>C	p.Ile743Thr	AR	M+M	compound I	/	Spastic paraplegia	/
AP4756	SPG7	25681447	F144	P168	/	10	Caucasian	Non-EA	NM_003119.3	c.1529C>T	p.Ala510Val	c.1529C>T	p.Ala510Val	AR	M+M	homo	/	Spastic paraplegia	/
AP4757	SPG7	25681447	F145	P169	/	11	Caucasian	Non-EA	NM_003119.3	c.1529C>T	p.Ala510Val	c.1053dup	p.Gly352Argfs	AR	M+T	compound I	/	Spastic paraplegia	/
AP4758	SPG7	25681447	F146	P170	/	12	Caucasian	Non-EA	NM_003119.3	c.1529C>T	p.Ala510Val	c.1225_1229d	p.Glu409Argfs	AR	M+T	compound I	/	Spastic paraplegia	/
AP4759	SPG7	25681447	F147	P171	/	13	Caucasian	Non-EA	NM_003119.3	c.1529C>T	p.Ala510Val	c.1529C>T	p.Ala510Val	AR	M+M	homo	/	Spastic paraplegia	/
AP4760	SPG7	25681447	F148	P172	/	14	Caucasian	Non-EA	NM_003119.3	c.1529C>T	p.Ala510Val	c.1529C>T	p.Ala510Val	AR	M+M	homo	/	Spastic paraplegia	/
AP4761	SPG7	25681447	F149	P173	/	15	Caucasian	Non-EA	NM_003119.3	c.1529C>T	p.Ala510Val	c.1715C>T	p.Ala572Val	AR	M+M	compound I	/	Spastic paraplegia	/
AP4762	SPG7	25681447	F150	P174	/	16	Caucasian	Non-EA	NM_003119.3	c.1529C>T	p.Ala510Val	c.1053dup	p.Gly352Argfs	AR	M+T	compound I	/	Spastic paraplegia	/
AP4763	SPG7	25681447	F151	P175	/	17	Caucasian	Non-EA	NM_003119.3	c.1529C>T	p.Ala510Val	c.1053dup	p.Gly352Argfs	AR	M+T	compound I	/	Spastic paraplegia	/
AP4764	SPG7	25976027	F152	P176	/	1	Caucasian	Non-EA	NM_003119.3	c.1529C>T	p.Ala510Val	c.1053dupC	p.Gly352Argfs	AR	M+T	compound I	Female	Spastic paraplegia	/
AP4765	SPG7	25976027	F153	P177	/	2	Caucasian	Non-EA	NM_003119.3	c.1529C>T	p.Ala510Val	c.233T>A	p.Leu78*	AR	M+T	compound I	Female	Spastic paraplegia	/
AP4766	SPG7	26260707	F154	P178	/	V.2	Ioannina (Western	Non-EA	NM_003119.3	c.27_51del	p.Ala10Valfs*48	c.27_51del	p.Ala10Valfs*4	AR	T+T	homo	/	Spastic paraplegia	/
AP4767	SPG7	26260707	F154	P179	/	V.3	Ioannina (Western	Non-EA	NM_003119.3	c.27_51del	p.Ala10Valfs*48	c.27_51del	p.Ala10Valfs*4	AR	T+T	homo	/	Spastic paraplegia	/
AP4768	SPG7	26260707	F154	P180	/	V.4	Ioannina (Western	Non-EA	NM_003119.3	c.27_51del	p.Ala10Valfs*48	c.27_51del	p.Ala10Valfs*4	AR	T+T	homo	/	Spastic paraplegia	/
AP4769	SPG7	26626314	F155	P181	A	1	French Canadian	Non-EA	NM_003119.3	c.1529C>T	p.Ala510Val	c.2249C>T	p.Pro750Leu	AR	M+M	compound I	/	Spastic paraplegia	/
AP4770	SPG7	26626314	F155	P182	A	2	French Canadian	Non-EA	NM_003119.3	c.1529C>T	p.Ala510Val	c.2249C>T	p.Pro750Leu	AR	M+M	compound I	/	Spastic paraplegia	/
AP4771	SPG7	26626314	F155	P183	A	3	French Canadian	Non-EA	NM_003119.3	c.1529C>T	p.Ala510Val	c.2249C>T	p.Pro750Leu	AR	M+M	compound I	/	Spastic paraplegia	/
AP4772	SPG7	26626314	F155	P184	A	4	French Canadian	Non-EA	NM_003119.3	c.1529C>T	p.Ala510Val	c.2249C>T	p.Pro750Leu	AR	M+M	compound I	/	Spastic paraplegia	/
AP4773	SPG7	26626314	F156	P185	B	5	Caucasian	Non-EA	NM_003119.3	c.1529C>T	p.Ala510Val	c.1715C>T	p.Ala572Val	AR	M+M	compound I	/	Spastic paraplegia	/
AP4774	SPG7	26626314	F156	P186	B	6	Caucasian	Non-EA	NM_003119.3	c.1529C>T	p.Ala510Val	c.1715C>T	p.Ala572Val	AR	M+M	compound I	/	Spastic paraplegia	/
AP4775	SPG7	26626314	F156	P187	B	7	Caucasian	Non-EA	NM_003119.3	c.1529C>T	p.Ala510Val	c.1715C>T	p.Ala572Val	AR	M+M	compound I	/	Spastic paraplegia	/
AP4776	SPG7	26626314	F156	P188	B	8	Caucasian	Non-EA	NM_003119.3	c.1529C>T	p.Ala510Val	c.1715C>T	p.Ala572Val	AR	M+M	compound I	/	Spastic paraplegia	/
AP4777	SPG7	26626314	F157	P189	C	9	Caucasian	Non-EA	NM_003119.3	c.1529C>T	p.Ala510Val	c.988−1G>A	p.?	AR	M+T	compound I	/	Spastic paraplegia	/
AP4778	SPG7	26626314	F158	P190	D	10	Caucasian	Non-EA	NM_003119.3	c.1529C>T	p.Ala510Val	c.1715C>T	p.Ala572Val	AR	M+M	compound I	/	Spastic paraplegia	/
AP4779	SPG7	26626314	F159	P191	E	11	Caucasian	Non-EA	NM_003119.3	c.1045G>A	p.Gly349Ser	c.2249C>T	p.Pro750Leu	AR	M+M	compound I	/	Spastic paraplegia	/
AP4780	SPG7	26626314	F159	P192	E	12	Caucasian	Non-EA	NM_003119.3	c.1045G>A	p.Gly349Ser	c.2249C>T	p.Pro750Leu	AR	M+M	compound I	/	Spastic paraplegia	/
AP4781	SPG7	26626314	F160	P193	F	13	Caucasian	Non-EA	NM_003119.3	c.1045G>A	p.Gly349Ser	c.2249C>T	p.Pro750Leu	AR	M+M	compound I	/	Spastic paraplegia	/
AP4782	SPG7	26626314	F161	P194	G	14	Caucasian	Non-EA	NM_003119.3	c.1529C>T	p.Ala510Val	c.233T>A	p.Leu78*	AR	M+T	compound I	/	Spastic paraplegia	/
AP4783	SPG7	26626314	F162	P195	H	15	Caucasian	Non-EA	NM_003119.3	c.1529C>T	p.Ala510Val	c.1529C>T	p.Ala510Val	AR	M+M	homo	/	Spastic paraplegia	/
AP4784	SPG7	26626314	F163	P196	I	16	Caucasian	Non-EA	NM_003119.3	c.988−1G>A	p.?	c.1715C>T	p.Ala572Val	AR	M+T	compound I	/	Spastic paraplegia	/
AP4785	SPG7	26626314	F163	P197	I	17	Caucasian	Non-EA	NM_003119.3	c.988−1G>A	p.?	c.1715C>T	p.Ala572Val	AR	M+T	compound I	/	Spastic paraplegia	/
AP4786	SPG7	26626314	F164	P198	J	18	Caucasian	Non-EA	NM_003119.3	c.233T>A	p.Leu78*	c.988−1G>A	p.?	AR	T+T	compound I	/	Spastic paraplegia	/
AP4787	SPG7	26626314	F165	P199	K	19	Caucasian	Non-EA	NM_003119.3	c.988−1G>A	p.?	c.988−1G>A	p.?	AR	T+T	homo	/	Spastic paraplegia	/
AP4788	SPG7	26626314	F165	P200	K	20	Caucasian	Non-EA	NM_003119.3	c.988−1G>A	p.?	c.988−1G>A	p.?	AR	T+T	homo	/	Spastic paraplegia	/
AP4789	SPG7	26626314	F166	P201	K	21	Caucasian	Non-EA	NM_003119.3	c.988−1G>A	p.?	c.988−1G>A	p.?	AR	T+T	homo	/	Spastic paraplegia	/
AP4790	SPG7	26626314	F167	P202	M	22	Caucasian	Non-EA	NM_003119.3	c.473_474del	p.Leu158GlnfsTer	c.988−1G>A	p.?	AR	T+T	compound I	/	Spastic paraplegia	/
AP4791	SPG7	26756429	F168	P203	A	AIV-2	Norwegian	Non-EA	NM_003119.3	c.2102A>C	p.His701Pro	c.2102A>C	p. His701Pro	AR	M+M	homo	/	Spastic paraplegia	/
AP4792	SPG7	26756429	F168	P204	A	AIV-4	Norwegian	Non-EA	NM_003119.3	c.2102A>C	p.His701Pro	c.2102A>C	p. His701Pro	AR	M+M	homo	/	Spastic paraplegia	/
AP4793	SPG7	26756429	F169	P205	B	BIII-2	Norwegian	Non-EA	NM_003119.3	c.2102A>C	p.His701Pro	c.1454_1462d	p.Arg485_Glu	AR	M+M	compound I	/	Spastic paraplegia	/
AP4794	SPG7	26756429	F169	P206	B	BIII-4	Norwegian	Non-EA	NM_003119.3	c.2102A>C	p.His701Pro	c.1454_1462d	p.Arg485_Glu	AR	M+M	compound I	/	Spastic paraplegia	/
AP4795	SPG7	26756429	F170	P207	C		Norwegian	Non-EA	NM_003119.3	c.1053dup	p.G352Rfs*44	c.1053dup	p.Gly352Argfs	AR	T+T	homo	/	Spastic paraplegia	/
AP4796	SPG7	26756429	F171	P208	D	DIII-3	Norwegian	Non-EA	NM_003119.3	c.2102A>C	p.His701Pro	c.1529C>T	p.Ala510Val	AR	M+M	compound I	/	Spastic paraplegia	/
AP4797	SPG7	26756429	F171	P209	D	DIII-4	Norwegian	Non-EA	NM_003119.3	c.2102A>C	p.His701Pro	c.1529C>T	p.Ala510Val	AR	M+M	compound I	/	Spastic paraplegia	/
AP4798	SPG7	26756429	F172	P210	E	EIII-1	Norwegian	Non-EA	NM_003119.3	c.2102A>C	p.His701Pro	c.1529C>T	p.Ala510Val	AR	M+M	compound I	/	Spastic paraplegia	/
AP4799	SPG7	26756429	F172	P211	E	EIII-2	Norwegian	Non-EA	NM_003119.3	c.2102A>C	p.His701Pro	c.1529C>T	p.Ala510Val	AR	M+M	compound I	/	Spastic paraplegia	/
AP4800	SPG7	26756429	F173	P212	F	FIII-1	Norwegian	Non-EA	NM_003119.3	c.1672A>T	p.Lys558*	c.1529C>T	p.Ala510Val	AR	M+T	compound I	/	Spastic paraplegia	/
AP4801	SPG7	26756429	F173	P213	F	FIII-2	Norwegian	Non-EA	NM_003119.3	c.1672A>T	p.Lys558*	c.1529C>T	p.Ala510Val	AR	M+T	compound I	/	Spastic paraplegia	/
AP4802	SPG7	27077743	F174	P214	/	#43	Caucasian	Non-EA	NM_003119.3	c.637C>T	p.Arg213*	c.1231G>A	p.Asp412Asn	AR	T+T	compound I	/	Spastic paraplegia	/
AP4803	SPG7	27077743	F175	P215	/	#57	Caucasian	Non-EA	NM_003119.3	c.1529C>T	p.Ala510Val	c.1529C>T	p.Ala510Val	AR	M+M	homo	/	Spastic paraplegia	/
AP4804	SPG7	27077743	F176	P216	/	#58	Caucasian	Non-EA	NM_003119.3	c.1529C>T	p.Ala510Val	c.1529C>T	p.Ala510Val	AR	M+M	homo	/	Spastic paraplegia	/
AP4805	SPG7	27077743	F177	P217	/	#59	Caucasian	Non-EA	NM_003119.3	c.233T>A	p.Leu78*	c.233T>A	p.Leu78*	AR	T+T	homo	/	Spastic paraplegia	/
AP4806	SPG7	27077743	F178	P218	/		Caucasian	Non-EA	NM_003119.3					AD	Truncation	homo	/	Spastic paraplegia	/
AP4807	SPG7	27084228	F179	P219	/	13	Hungary	Non-EA	NM_003119.3	c.233T>A	p.Leu78*	/	/	AD	Truncation	heter	/	Spastic paraplegia	/
AP4808	SPG7	27084228	F180	P220	/	14	Hungary	Non-EA	NM_003119.3	c.1529C>T	p.Ala510Val	c.233T>A	p.Leu78*	AR	M+T	compound I	/	Spastic paraplegia	/
AP4809	SPG7	27084228	F181	P221	/	15	Hungary	Non-EA	NM_003119.3	c.233T>A	p.Leu78*	/	/	AD	Truncation	heter	/	Spastic paraplegia	/
AP4810	SPG7	27084228	F182	P222	/	16	Hungary	Non-EA	NM_003119.3	c.233T>A	p.Leu78*	/	/	AD	Truncation	heter	/	Spastic paraplegia	/
AP4811	SPG7	27084228	F183	P223	/	17	Hungary	Non-EA	NM_003119.3	c.1529C>T	p.Ala510Val	c.1031G>A	p.Gly344Asp	AR	M+M	compound I	/	Spastic paraplegia	/
AP4812	SPG7	27123479	F184	P224	/	II-1	Caucasian	Non-EA	NM_003119.3	c.2228T>C	p.Ile743Thr	c.2084T>C	p.Leu695Pro	AR	M+M	compound I	Male	Spastic paraplegia	/
AP4813	SPG7	27123479	F184	P225	/	II-3	Caucasian	Non-EA	NM_003119.3	c.2228T>C	p.Ile743Thr	c.2084T>C	p.Leu695Pro	AR	M+M	compound I	Male	Spastic paraplegia	/
AP4814	SPG7	27123479	F184	P226	/	II-4	Caucasian	Non-EA	NM_003119.3	c.2228T>C	p.Ile743Thr	c.2084T>C	p.Leu695Pro	AR	M+M	compound I	Male	Spastic paraplegia	/
AP4815	SPG7	27123479	F184	P227	/	II-6	Caucasian	Non-EA	NM_003119.3	c.2228T>C	p.Ile743Thr	c.2084T>C	p.Leu695Pro	AR	M+M	compound I	Male	Spastic paraplegia	/
AP4816	SPG7	27123479	F184	P228	/	II-7	Caucasian	Non-EA	NM_003119.3	c.2228T>C	p.Ile743Thr	c.2084T>C	p.Leu695Pro	AR	M+M	compound I	Male	Spastic paraplegia	/
AP4817	SPG7	27165006	F185	P229	/	1	Caucasian	Non-EA	NM_003119.3	c.1454_1462del	p.Arg485_Glu487	c.1529C>T	p.Ala510Val	AR	M+M	compound I	Female	Spastic paraplegia	/
AP4818	SPG7	27165006	F186	P230	/	2	Caucasian	Non-EA	NM_003119.3	c.1454_1462del	p.Arg485_Glu487	c.1529C>T	p.Ala510Val	AR	M+M	compound I	Male	Spastic paraplegia	/
AP4819	SPG7	27165006	F187	P231	/	3	Caucasian	Non-EA	NM_003119.3	c.1529C>T	p.Ala510Val	c.1756G>T	p.Glu586*	AR	M+T	compound I	Male	Spastic paraplegia	/
AP4820	SPG7	27212339	F188	P232	/	35	Kenyan	Non-EA	NM_003119.3	c.233T>A	p.Leu78*	c.233T>A	p.Leu78*	AR	T+T	homo	Male	Spastic paraplegia	/
AP4821	SPG7	27212339	F189	P233	/	37	British	Non-EA	NM_003119.3	c.1672A>T	p.Lys558*	c.1672A>T	p.Lys558*	AR	T+T	homo	Male	Spastic paraplegia	/
AP4822	SPG7	27212339	F190	P234	/	38	British	Non-EA	NM_003119.3	c.1523G>A	p.Arg508His	c.1529C>T	p.Ala510Val	AR	M+M	compound I	Male	Spastic paraplegia	/
AP4823	SPG7	27212339	F191	P235	/	39	British	Non-EA	NM_003119.3	c.1450_1457de	p.E484Gfs*4	c.1450_1457d	p.E484Gfs*4	AR	T+T	homo	Female	Spastic paraplegia	/
AP4824	SPG7	27212339	F192	P236	/	44	British	Non-EA	NM_003119.3	c.1529C>T	p.Ala510Val	c.2102A>C	p. His701Pro	AR	M+M	compound I	Female	Spastic paraplegia	/
AP4825	SPG7	27260292	F193	P237	/	5	Caucasian	Non-EA	NM_003119.3	c.397C>T	p.Arg133Trp	/	/	AD	M	heter	Male	Spastic paraplegia	/
AP4826	SPG7	27790088	F194	P238	/	#385	German	Non-EA	NM_003119.3	c.637C>T	p.Arg213*	/	/	AD	Truncation	heter	Female	Spastic paraplegia	/
AP4827	SPG7	27790088	F195	P239	/	#41	German	Non-EA	NM_003119.3	c.2228T>C	p.Ile743Thr	/	/	AD	M	heter	Male	Spastic paraplegia	/
AP4828	SPG7	27790088	F196	P240	/	#19	German	Non-EA	NM_003119.3	c.1045G>A	p.Gly349Ser	/	/	AD	M	heter	Male	Spastic paraplegia	/
AP4829	SPG7	27790088	F197	P241	/	#28	German	Non-EA	NM_003119.3	c.1045G>A	p.Gly349Ser	/	/	AD	M	heter	Female	Spastic paraplegia	/
AP4830	SPG7	28362824	F198	P242	HCT-048		Norwegian	Non-EA	NM_003119.3	c.233T>A	p.Leu78*	c.233T>A	p.Leu78*	AR	T+T	homo	Male	Spastic paraplegia	/
AP4831	SPG7	28362824	F199	P243	HCT-033		Norwegian	Non-EA	NM_003119.3	c.1529C>T	p.Ala510Val	c.1672A>T	p.Lys558*	AR	M+T	compound I	Male	Spastic paraplegia	/
AP4832	SPG7	28362824	F200	P244	HCT-112		Norwegian	Non-EA	NM_003119.3	c.1529C>T	p.Ala510Val	c.2102A>C	p. His701Pro	AR	M+M	compound I	Male	Spastic paraplegia	/
AP4833	SPG7	28362824	F201	P245	HCT-116		Norwegian	Non-EA	NM_003119.3	c.1529C>T	p.Ala510Val	c.2102A>C	p. His701Pro	AR	M+M	compound I	Female	Spastic paraplegia	/
AP4834	SPG7	28444220	F202	P246	AAD-785-9		Caucasian	Non-EA	NM_003119.3	c.1529C>T	p.Ala510Val	c.858dupT	p.Ala286fs	AR	M+T	compound I	/	Spastic paraplegia	/
AP4835	SPG7	28444220	F203	P247	AAD-248-12		Caucasian	Non-EA	NM_003119.3	c.958G>T	p.Glu320*	c.1047dupC	p.G349fs	AR	T+T	compound I	/	Spastic paraplegia	/
AP4836	SPG7	28444220	F204	P248	AAD-420-1		Caucasian	Non-EA	NM_003119.3	c.1529C>T	p.Ala510Val	c.1519C>T	p.Gln507*	AR	M+T	compound I	/	Spastic paraplegia	/
AP4837	SPG7	28444220	F205	P249	AAR-541-13		Caucasian	Non-EA	NM_003119.3	c.1529C>T	p.Ala510Val	c.1519C>T	p.Gln507*	AR	M+T	compound I	/	Spastic paraplegia	/
AP4838	SPG7	28444220	F206	P250	AAD-951-11		Caucasian	Non-EA	NM_003119.3	c.1529C>T	p.Ala510Val	c.1529C>T	p.Ala510Val	AR	M+M	homo	/	Spastic paraplegia	/
AP4839	SPG7	28444220	F207	P251	AAD-847-18		Caucasian	Non-EA	NM_003119.3	c.1529C>T	p.Ala510Val	c.1529C>T	p.Ala510Val	AR	M+M	homo	/	Spastic paraplegia	/
AP4840	SPG7	28444220	F208	P252	AAD-1033-1		Caucasian	Non-EA	NM_003119.3	c.1529C>T	p.Ala510Val	c.1529C>T	p.Ala510Val	AR	M+M	homo	/	Spastic paraplegia	/
AP4841	SPG7	28444220	F209	P253	SAL-399-1026		Caucasian	Non-EA	NM_003119.3	c.1529C>T	p.Ala510Val	c.1529C>T	p.Ala510Val	AR	M+M	homo	/	Spastic paraplegia	/
AP4842	SPG7	28444220	F210	P254	AAD-458-5		Caucasian	Non-EA	NM_003119.3	c.1529C>T	p.Ala510Val	c.2249C>T	p.Pro750Leu	AR	M+M	compound I	/	Spastic paraplegia	/
AP4843	SPG7	28832565	F211	P255	SR75		Portuguese	Non-EA	NM_003119.3	c.1454_1462del	p.Arg485_Glu487	c.1454_1462d	p.Arg485_Glu	AR	M+M	homo	Male	Spastic paraplegia	/
AP4844	SPG7	28832565	F212	P256	S26		Portuguese	Non-EA	NM_003119.3	c.1447C>T	p.Gln483*	c.1447C>T	p.Gln483*	AR	T+T	homo	Male	Spastic paraplegia	/
AP4845	SPG7	28832565	F213	P257	CI3		Portuguese	Non-EA	NM_003119.3	c.1529C>T	p.Ala510Val	c.376G>C	p.Glu126Gln	AR	M+M	compound I	Male	Spastic paraplegia	/
AP4846	SPG7	29026558	F214	P258	/		Caucasian	Non-EA	NM_003119.3	c.1529C>T	p.Ala510Val	c.1454_1462d	p.Arg485_Glu	AR	M+M	compound I	Male	Spastic paraplegia	/
AP4847	SPG7	29246610	F215	P259	21	HSP41	Caucasian	Non-EA	NM_003119.3	c.1450-1_1457delGGA	p.Glu484Glyfs*4	c.2014G > A	p.Gly672Arg	AR	M+T	compound I	/	Spastic paraplegia	/
AP4848	SPG7	29246610	F216	P260	22	HSP42	Caucasian	Non-EA	NM_003119.3	c.1450-1_1457delGGA	p.Glu484Glyfs*4	c.2014G > A	p.Gly672Arg	AR	M+T	compound I	/	Spastic paraplegia	/
AP4849	SPG7	29246610	F217	P261	22	HSP43	Caucasian	Non-EA	NM_003119.3	c.1450-1_1457delGGA	p.Glu484Glyfs*4	c.2014G > A	p.Gly672Arg	AR	M+T	compound I	/	Spastic paraplegia	/
AP4850	SPG7	29246610	F218	P262	22	HSP44	Caucasian	Non-EA	NM_003119.3	c.1450-1_1457delGGA	p.Glu484Glyfs*4	c.2014G > A	p.Gly672Arg	AR	M+T	compound I	/	Spastic paraplegia	/
AP4851	SPG7	29482223	F219	P263	/	AAR-40-9	Caucasian	Non-EA	NM_003119.3	c.1529C>T	p.Ala510Val	c.1529C>T	p.Ala510Val	AR	M+M	homo	/	Spastic paraplegia	/
AP4852	SPG7	29482223	F220	P264	/	AAD-81-8	Caucasian	Non-EA	NM_003119.3	c.1529C>T	p.Ala510Val	c.1747T>C	p.Trp583Arg	AR	M+M	compound I	/	Spastic paraplegia	/
AP4853	SPG7	29482223	F221	P265	/	AAR-331-6	Caucasian	Non-EA	NM_003119.3	c.1529C>T	p.Ala510Val	c.1529C>T	p.Ala510Val	AR	M+M	homo	/	Spastic paraplegia	/
AP4854	SPG7	29482223	F222	P266	/	AAR-339-1	Caucasian	Non-EA	NM_003119.3	c.1529C>T	p.Ala510Val	c.1529C>T	p.Ala510Val	AR	M+M	homo	/	Spastic paraplegia	/
AP4855	SPG7	29482223	F223	P267	/	SAL-399-45	Caucasian	Non-EA	NM_003119.3	c.1529C>T	p.Ala510Val	c.233T>A	p.Leu78*	AR	M+T	compound I	/	Spastic paraplegia	/
AP4856	SPG7	29482223	F224	P268	/	SAL-399-216	Caucasian	Non-EA	NM_003119.3	c.1529C>T	p.Ala510Val	c.2084T>C	p.Leu695Pro	AR	M+M	compound I	/	Spastic paraplegia	/
AP4857	SPG7	29482223	F225	P269	/	SAL-399-225	Caucasian	Non-EA	NM_003119.3	c.1529C>T	p.Ala510Val	c.1894G>A	p.Gly632Arg	AR	M+M	compound I	/	Spastic paraplegia	/
AP4858	SPG7	29482223	F226	P270	/	SAL-399-327	Caucasian	Non-EA	NM_003119.3	c.1529C>T	p.Ala510Val	c.233T>A	p.Leu78*	AR	M+T	compound I	/	Spastic paraplegia	/
AP4859	SPG7	29482223	F227	P271	/	SAL-399-473	Caucasian	Non-EA	NM_003119.3	c.1529C>T	p.Ala510Val	c.86G>A	p.Trp29*	AR	M+T	compound I	/	Spastic paraplegia	/
AP4860	SPG7	29482223	F228	P272	/	SAL-399-566	Caucasian	Non-EA	NM_003119.3	c.1529C>T	p.Ala510Val	c.2228T>C	p.Ile743Thr	AR	M+M	compound I	/	Spastic paraplegia	/
AP4861	SPG7	29482223	F229	P273	/	SAL-399-961	Caucasian	Non-EA	NM_003119.3	c.1529C>T	p.Ala510Val	c.1972G>A	p.Ala658Thr	AR	M+M	compound I	/	Spastic paraplegia	/
AP4862	SPG7	29482223	F230	P274	/	SAL-541-9	Caucasian	Non-EA	NM_003119.3	c.1519C>T	p.Gln507*	c.1529C>T	p.Ala510Val	AR	M+T	compound I	/	Spastic paraplegia	/
AP4863	SPG7	29482223	F231	P275	/	AAD-847-18	Caucasian	Non-EA	NM_003119.3	c.1529C>T	p.Ala510Val	c.1529C>T	p.Ala510Val	AR	M+M	homo	/	Spastic paraplegia	/
AP4864	SPG7	29482223	F232	P276	/	AAR-628-1	Caucasian	Non-EA	NM_003119.3	c.1408C>T	p.Arg70*	c.1715C>T	p.Ala572Val	AR	M+T	compound I	/	Spastic paraplegia	/
AP4865	SPG7	29913018	F233	P277	/	ATX167	Caucasian	Non-EA	NM_003119.3	c.1749G>C	p.Trp583Cys	c.1529C>T	p.Ala510Val	AR	M+M	compound I	/	Spastic paraplegia	/
AP4866	SPG7	29915382	F234	P278	/	59	Caucasian	Non-EA	NM_003119.3	c.1529C>T	p.Ala510Val	.2181+5G>A	p.?	AR	M+T	compound I	Male	Spastic paraplegia	/
AP4867	SPG7	29915382	F235	P279	/	60	Caucasian	Non-EA	NM_003119.3	c.1529C>T	p.Ala510Val	c.1529C>T	p.Ala510Val	AR	M+M	homo	Male	Spastic paraplegia	/
AP4868	SPG7	29915382	F236	P280	/	61	Caucasian	Non-EA	NM_003119.3	c.1904C>T	p.Ser635Le	c.2254C>G	p.His752Asp	AR	M+M	compound I	Female	Spastic paraplegia	/
AP4869	SPG7	29915382	F237	P281	/	62	Caucasian	Non-EA	NM_003119.3	c.861dup	p.Asn288*	c.1529C>T	p.Ala510Val	AR	M+T	compound I	Female	Spastic paraplegia	/
AP4870	SPG7	29915382	F238	P282	/	63	Caucasian	Non-EA	NM_003119.3	Exon 6 deletion		Exon 6 deletio	p.?	AR	CNV+CNV	homo	Male	Spastic paraplegia	/
AP4871	SPG7	29915382	F239	P283	/	64	Caucasian	Non-EA	NM_003119.3	c.1529C>T	p.Ala510Val	c.1529C>T	p.Ala510Val	AR	M+M	homo	Male	Spastic paraplegia	/
AP4872	SPG7	29915382	F240	P284	/	65	Caucasian	Non-EA	NM_003119.3	c.1454_1462del	p. Arg485_Glu487	c.1033G>C	p.Ala345Pro	AR	M+M	compound I	Male	Spastic paraplegia	/

**AP4873**	*SPG7*	29915382	F241	P285	/	66	Caucasian	Non-EA	NM_003119.3	c.988−1G>A	p.?	c.1529C>T	p.Ala510Val	AR	M+T	compound I	Female
**AP4874**	*SPG7*	30098094	F242	P286	A	ATA-1	Italian	Non-EA	NM_003119.3	c.1529C>T	p.Ala510Val	c.1529C>T	p.Ala510Val	AR	M+M	homo	Male
**AP4875**	*SPG7*	30098094	F242	P287	A	ATA-2	Italian	Non-EA	NM_003119.3	c.1529C>T	p.Ala510Val	c.1529C>T	p.Ala510Val	AR	M+M	homo	Male
**AP4876**	*SPG7*	30098094	F243	P288	B	ATA-3	Italian	Non-EA	NM_003119.3	c.1529C>T	p.Ala510Val	c.1529C>T	p.Ala510Val	AR	M+M	homo	Male
**AP4877**	*SPG7*	30098094	F244	P289	C	ATA-4	Italian	Non-EA	NM_003119.3	c.1529C>T	p.Ala510Val	c.1529C>T	p.Ala510Val	AR	M+M	homo	Male
**AP4878**	*SPG7*	30098094	F245	P290	D	ATA-5	Italian	Non-EA	NM_003119.3	c.1529C>T	p.Ala510Val	c.1529C>T	p.Ala510Val	AR	M+M	homo	Male
**AP4879**	*SPG7*	30098094	F246	P291	E	ATA-6	Italian	Non-EA	NM_003119.3	c.1529C>T	p.Ala510Val	c.1529C>T	p.Ala510Val	AR	M+M	homo	Male
**AP4880**	*SPG7*	30098094	F247	P292	F	ATA-7	Italian	Non-EA	NM_003119.3	c.1529C>T	p.Ala510Val	c.1529C>T	p.Ala510Val	AR	M+M	homo	Male
**AP4881**	*SPG7*	30098094	F248	P293	F	ATA-8	Italian	Non-EA	NM_003119.3	c.1529C>T	p.Ala510Val	c.1529C>T	p.Ala510Val	AR	M+M	homo	Female
**AP4882**	*SPG7*	30098094	F249	P294	G	ATA-9	Italian	Non-EA	NM_003119.3	c.1529C>T	p.Ala510Val	c.1529C>T	p.Ala510Val	AR	M+M	homo	Male
**AP4883**	*SPG7*	30098094	F250	P295	H	ATA-10	Italian	Non-EA	NM_003119.3	c.1529C>T	p.Ala510Val	c.1529C>T	p.Ala510Val	AR	M+M	homo	Male
**AP4884**	*SPG7*	30098094	F251	P296	I	ATA-11	Italian	Non-EA	NM_003119.3	c.1529C>T	p.Ala510Val	c.1617delC	p.V540Cfs*52	AR	M+T	compound I	Female
**AP4885**	*SPG7*	30098094	F252	P297	J	ATA-12	Italian	Non-EA	NM_003119.3	c.1529C>T	p.Ala510Val	c.2228T>C	p.Ile743Thr	AR	M+M	compound I	Male
**AP4886**	*SPG7*	30098094	F253	P298	K	ATA-13	Italian	Non-EA	NM_003119.3	c.1529C>T	p.Ala510Val	c.2102A>C	p. His701Pro	AR	M+M	compound I	Male
**AP4887**	*SPG7*	30098094	F254	P299	L	ATA-14	Italian	Non-EA	NM_003119.3	c.1529C>T	p.Ala510Val	c.287-1G>C	p.?	AR	M+T	compound I	Male
**AP4888**	*SPG7*	30098094	F255	P300	M	ATA-15	Italian	Non-EA	NM_003119.3	c.1529C>T	p.Ala510Val	c.1553-	p.?	AR	M+T	compound I	Male
**AP4889**	*SPG7*	30098094	F256	P301	N	ATA-16	Italian	Non-EA	NM_003119.3	c.1529C>T	p.Ala510Val	c.1972G>A	p.Ala658Thr	AR	M+M	compound I	Female
**AP4890**	*SPG7*	30098094	F257	P302	O	ATA-17	Italian	Non-EA	NM_003119.3	c.1529C>T	p.Ala510Val	c.1972G>A	p.Ala658Thr	AR	M+M	compound I	Female
**AP4891**	*SPG7*	30098094	F258	P303	P	ATA-18	Italian	Non-EA	NM_003119.3	c.1529C>T	p.Ala510Val	c.773_774del	p.Val258Glyfs*	AR	M+T	compound I	Female
**AP4892**	*SPG7*	30098094	F258	P304	P	ATA-19	Italian	Non-EA	NM_003119.3	c.1529C>T	p.Ala510Val	c.773_774del	p.Val258Glyfs*	AR	M+T	compound I	Female
**AP4893**	*SPG7*	30098094	F259	P305	Q	ATA-20	Italian	Non-EA	NM_003119.3	c.1529C>T	p.Ala510Val	c.1369C>T	p.Arg457*	AR	M+T	compound I	Female
**AP4894**	*SPG7*	30098094	F259	P306	Q	ATA-21	Italian	Non-EA	NM_003119.3	c.1529C>T	p.Ala510Val	c.1369C>T	p.Arg457*	AR	M+T	compound I	Male
**AP4895**	*SPG7*	30098094	F260	P307	R	ATA-22	Italian	Non-EA	NM_003119.3	c.1529C>T	p.Ala510Val	c.1A>G	p.Met1Val	AR	M+M	compound I	Female
**AP4896**	*SPG7*	30098094	F261	P308	S	ATA-23	Italian	Non-EA	NM_003119.3	c.1529C>T	p.Ala510Val	c.1877T>C	p.Met626Thr	AR	M+M	compound I	Female
**AP4897**	*SPG7*	30098094	F262	P309	T	ATA-24	Italian	Non-EA	NM_003119.3	c.1529C>T	p.Ala510Val	c.637C>T	p.Arg213*	AR	M+T	compound I	Male
**AP4898**	*SPG7*	30098094	F263	P310	U	ATA-25	Italian	Non-EA	NM_003119.3	c.1529C>T	p.Ala510Val	c.2181G>A	p.?	AR	M+T	compound I	Female
**AP4899**	*SPG7*	30369941	F264	P311	/		Caucasian	Non-EA	NM_003119.3	c.861dup	p.Asn288*	c.861dup	p.Asn288*	AR	T+T	homo	Male
**AP4900**	*SPG7*	30369941	F265	P312	/		Caucasian	Non-EA	NM_003119.3	c.1529C>T	p.Ala510Val	c.2090A>C	p.Gln697Pro	AR	M+M	compound I	Male
**AP4901**	*SPG7*	30497413	F265	P313	/	II-1	Chinese	EA	NM_003119.3	c.1150_1150-1insCTA(	p. ?	c.2062_2063d	p.Arg688*	AR	T+T	compound I	/
**AP4902**	*SPG7*	30497413	F265	P314	/	II-3	Chinese	EA	NM_003119.3	c.1150_1150-1insCTA(	p. ?	c.2062_2063d	p.Arg688*	AR	T+T	compound I	/
**AP4903**	*SPG7*	30497413	F265	P315	/	II-4	Chinese	EA	NM_003119.3	c.1150_1150-1insCTA(	p. ?	c.2062_2063d	p.Arg688*	AR	T+T	compound I	/
**AP4904**	*SPG7*	30533525	F266	P316	1	/	Englander	Non-EA	NM_003119.3	c.1529C>T	p.Ala510Val	c.1529C>T	p.Ala510Val	AR	M+M	homo	Male
**AP4905**	*SPG7*	30533525	F267	P317	2	/	Englander	Non-EA	NM_003119.3	c.1529C>T	p.Ala510Val	c.1937-2A>G	p.?	AR	M+T	compound I	Male
**AP4906**	*SPG7*	30533525	F268	P318	3	/	Englander	Non-EA	NM_003119.3	c.1529C>T	p.Ala510Val	c.1529C>T	p.Ala510Val	AR	M+M	homo	Male
**AP4907**	*SPG7*	30533525	F269	P319	4	/	Englander	Non-EA	NM_003119.3	c.1529C>T	p.Ala510Val	c.1454_1462(	p.Arg485_Glu	AR	M+M	compound I	Male
**AP4908**	*SPG7*	30533525	F270	P320	5	/	Englander	Non-EA	NM_003119.3	c.1529C>T	p.Ala510Val	c.1529C>T	p.Ala510Val	AR	M+M	homo	Male
**AP4909**	*SPG7*	30533525	F271	P321	6	/	Englander	Non-EA	NM_003119.3	c.1529C>T	p.Ala510Val	c.1045 G>A	.Gly349Ser	AR	M+M	compound I	Male
**AP4910**	*SPG7*	30533525	F272	P322	7	/	Englander	Non-EA	NM_003119.3	c.1529C>T	p.Ala510Val	c.1529C>T	p.Ala510Val	AR	M+M	homo	Male
**AP4911**	*SPG7*	30533525	F273	P323	8	/	Englander	Non-EA	NM_003119.3	c.1529C>T	p.Ala510Val	c.1045 G>A	.Gly349Ser	AR	M+M	compound I	Male
**AP4912**	*SPG7*	30533525	F274	P324	9	/	Englander	Non-EA	NM_003119.3	c.1529C>T	p.Ala510Val	c.1904C>T	p.Ser635Leu	AR	M+M	compound I	Female
**AP4913**	*SPG7*	30533525	F275	P325	10	/	Englander	Non-EA	NM_003119.3	c.1529C>T	p.Ala510Val	c.1454_1462(	p.Arg485_Glu	AR	M+M	compound I	Male
**AP4914**	*SPG7*	30533525	F276	P326	11	/	Englander	Non-EA	NM_003119.3	c.1529C>T	p.Ala510Val	c.1192C>T	p.Arg398*	AR	M+T	compound I	Male
**AP4915**	*SPG7*	30533525	F277	P327	12	/	Englander	Non-EA	NM_003119.3	c.1529C>T	p.Ala510Val	c.1529C>T	p.Ala510Val	AR	M+M	homo	Male
**AP4916**	*SPG7*	30533525	F278	P328	13	/	Englander	Non-EA	NM_003119.3	c.1529C>T	p.Ala510Val	c.1529C>T	p.Ala510Val	AR	M+M	homo	Male
**AP4917**	*SPG7*	30533525	F279	P329	14	/	Englander	Non-EA	NM_003119.3	c.233T>A	p.Leu78*	c.233T>A	p.Leu78*	AR	T+T	homo	Male
**AP4918**	*SPG7*	30533525	F280	P330	15	/	Englander	Non-EA	NM_003119.3	c.1529C>T	p.Ala510Val	c.861+2dupT	p.?	AR	M+T	compound I	Male

[illegible]

**AP5361**	***WFS1***	29529044	F133	P286	Family #15	15-1	Japanese	EA	NM_006005.3	c.2507A>C	p.Lys836Thr	/	/	AD	M	heter	Female	Non-syndromic hearing loss	/	/	/	/	/	/
**AP5362**	***WFS1***	29529044	F133	P287	Family #15	15-2	Japanese	EA	NM_006005.3	c.2507A>C	p.Lys836Thr	/	/	AD	M	heter	Male	Non-syndromic hearing loss	/	/	/	/	/	/
**AP5363**	***WFS1***	29529044	F134	P288	Family #16	16-1	Japanese	EA	NM_006005.3	c.2507A>C	p.Lys836Thr	/	/	AD	M	heter	Male	Non-syndromic hearing loss	/	/	/	/	/	/
**AP5364**	***WFS1***	29529044	F134	P289	Family #16	16-2	Japanese	EA	NM_006005.3	c.2507A>C	p.Lys836Thr	/	/	AD	M	heter	Female	Non-syndromic hearing loss	/	/	/	/	/	/
**AP5365**	***WFS1***	29529044	F135	P290	Family #17	17-1	Japanese	EA	NM_006005.3	c.2508G>C	p.Lys836Asn	/	/	AD	M	heter	Male	Non-syndromic hearing loss	/	/	/	/	/	/
**AP5366**	***WFS1***	29529044	F135	P291	Family #17	17-2	Japanese	EA	NM_006005.3	c.2508G>C	p.Lys836Asn	/	/	AD	M	heter	Female	Non-syndromic hearing loss	/	/	/	/	/	/
**AP5367**	***WFS1***	29529044	F135	P292	Family #17	17-3	Japanese	EA	NM_006005.3	c.2508G>C	p.Lys836Asn	/	/	AD	M	heter	Female	Non-syndromic hearing loss	/	/	/	/	/	/
**AP5368**	***WFS1***	29529044	F136	P293	Family #18	18-1	Japanese	EA	NM_006005.3	c.2590G>A	p.Glu864Lys	/	/	AD	M	heter	Female	Wolfram Syndrome	22.00	/	/	/	/	/
**AP5369**	***WFS1***	29529044	F136	P294	Family #18	18-2	Japanese	EA	NM_006005.3	c.2590G>A	p.Glu864Lys	/	/	AD	M	heter	Female	Non-syndromic hearing loss	/	/	/	/	/	/
**AP5370**	***WFS1***	29529044	F137	P295	Family #19	19-1	Japanese	EA	NM_006005.3	c.2590G>A	p.Glu864Lys	/	/	AD	M	heter	Female	Non-syndromic hearing loss	/	/	/	/	/	/
**AP5371**	***WFS1***	29529044	F137	P296	Family #19	19-2	Japanese	EA	NM_006005.3	c.2590G>A	p.Glu864Lys	/	/	AD	M	heter	Male	Non-syndromic hearing loss	/	/	/	/	/	/
**AP5372**	***WFS1***	29483894	F138	P297	/	Case 3	Chinese	EA	NM_006005.3	c.453_460delCAGAAG	p.Asp151Glufs*93	c.1300_1302d	p.Val434del	AR	M+T	compound	Male	Wolfram Syndrome	5.00	/	/	Optic atrophy HP	/	/
**AP5373**	***WFS1***	29447883	F139	P298	Family 18	VI:2	Chinese	EA	NM_006005.3	c.2389G>A	p.Asp797Asn	/	/	AD	M	heter	Male	Non-syndromic hearing loss	/	/	/	/	/	/
**AP5374**	***WFS1***	29447883	F139	P299	Family 18	V:4	Chinese	EA	NM_006005.3	c.2389G>A	p.Asp797Asn	/	/	/	M	heter	Male	Non-syndromic hearing loss	/	/	/	/	/	/
**AP5375**	***WFS1***	29447883	F139	P300	Family 18	V:29	Chinese	EA	NM_006005.3	c.2389G>A	p.Asp797Asn	/	/	/	M	heter	Male	Non-syndromic hearing loss	/	/	/	/	/	/
**AP5376**	***WFS1***	29447883	F140	P301	Family 21	IV:1	Chinese	EA	NM_006005.3	c.2389G>A	p.Asp797Asn	/	/	AD	M	heter	Male	Non-syndromic hearing loss	/	/	/	/	/	/
**AP5377**	***WFS1***	29447883	F140	P302	Family 21	IV:39	Chinese	EA	NM_006005.3	c.2389G>A	p.Asp797Asn	/	/	/	M	heter	Male	Non-syndromic hearing loss	/	/	/	/	/	/
**AP5378**	***WFS1***	29447883	F140	P303	Family 21	V:11	Chinese	EA	NM_006005.3	c.2389G>A	p.Asp797Asn	/	/	/	M	heter	Male	Non-syndromic hearing loss	/	/	/	/	/	/
**AP5379**	***WFS1***	29348930	F141	P304	/	/	Chinese	EA	NM_006005.3	c.1726G>A	p.Gly576Ser	/	/	AD	M	heter	/	Hereditary Optic Neuropathy	/	/	/	Optic atrophy HP	/	/
**AP5380**	***WFS1***	29348930	F142	P305	/	/	Chinese	EA	NM_006005.3	c.1726G>A	p.Gly576Ser	/	/	AD	M	heter	/	Hereditary Optic Neuropathy	/	/	/	Optic atrophy HP	/	/
**AP5381**	***WFS1***	29258540	F143	P306	/	30-12	Korean	EA	NM_006005.3	c.2419A>C	p.Ser807Arg	/	/	AD	M	heter	/	low-	/	/	/	/	/	/
**AP5382**	***WFS1***	29258540	F143	P307	/	30-22	Korean	EA	NM_006005.3	c.2419A>C	p.Ser807Arg	/	/	AD	M	heter	/	low-	/	/	/	/	/	/
**AP5383**	***WFS1***	29183106	F144	P308	/	10	Caucasian	Non-EA	NM_006005.3	c.1385_1393del	p.Glu462_Thr464	c.1385_1393d	p.Glu462_Thr	AR	M+M	homo	Female	Optic atrophy	/	/	/	Optic atrophy HP	/	/
**AP5384**	***WFS1***	29183106	F145	P309	/	11	Caucasian	Non-EA	NM_006005.3	c.529C>T	p.Arg177Cys	c.529C>T	p.Arg177Cys	AR	M+M	homo	Female	Optic atrophy	/	/	/	Optic atrophy HP	/	/
**AP5385**	***WFS1***	29183106	F146	P310	/	12	Caucasian	Non-EA	NM_006005.3	c.1230_1233delCTCT	p.Val412Serfs*29	c.1230_1233d	p.Val412Serfs	AR	T+T	homo	Male	Hydrocephalus, primary hypothyro	/	/	/	/	/	/
**AP5386**	***WFS1***	29183106	F147	P311	/	13	Caucasian	Non-EA	NM_006005.3	c.776delT	p.Ile259Thrfs*28	c.776delT	p.Ile259Thrfs*	AR	T+T	homo	Male	Optic atrophy+Diabetes mellitus	/	/	/	Optic atrophy HP	/	/
**AP5387**	***WFS1***	28993341	F148	P312	/	P5	Caucasian	Non-EA	NM_006005.3	c.2129C>G	p.Thr710Ser	/	/	AD	M	heter	Male	Multigenerational Diabetes Mellitu	/	/	/	/	/	/
**AP5388**	***WFS1***	28974383	F149	P313	/	II-6	Finnish	Non-EA	NM_006005.3	c.1582T>C	p.Tyr528His	/	/	AD	M	heter	Female	Hearing impairment	/	/	/	/	/	/
**AP5389**	***WFS1***	28802351	F150	P314	Family XH-0	II-2	Chinese	EA	NM_006005.3	c.2591A>G	p.Glu864Gly	/	/	AD	M	heter	Female	Non-syndromic hearing loss	/	/	/	/	/	/
**AP5390**	***WFS1***	28802351	F150	P315	Family XH-0	II-4	Chinese	EA	NM_006005.3	c.2591A>G	p.Glu864Gly	/	/	AD	M	heter	Female	Non-syndromic hearing loss	/	/	/	/	/	/
**AP5391**	***WFS1***	28802351	F150	P316	Family XH-0	III-4	Chinese	EA	NM_006005.3	c.2591A>G	p.Glu864Gly	/	/	AD	M	heter	Female	Non-syndromic hearing loss	/	/	/	/	/	/
**AP5392**	***WFS1***	28802351	F150	P317	Family XH-0	III-7	Chinese	EA	NM_006005.3	c.2591A>G	p.Glu864Gly	/	/	AD	M	heter	Male	Non-syndromic hearing loss	/	/	/	/	/	/
**AP5393**	***WFS1***	28802351	F150	P318	Family XH-0	III-9	Chinese	EA	NM_006005.3	c.2591A>G	p.Glu864Gly	/	/	AD	M	heter	Female	Non-syndromic hearing loss	/	/	/	/	/	/
**AP5394**	***WFS1***	28802351	F150	P319	Family XH-0	IV-3	Chinese	EA	NM_006005.3	c.2591A>G	p.Glu864Gly	/	/	AD	M	heter	Male	Non-syndromic hearing loss	/	/	/	/	/	/
**AP5395**	***WFS1***	28802351	F150	P320	Family XH-0	IV-7	Chinese	EA	NM_006005.3	c.2591A>G	p.Glu864Gly	/	/	AD	M	heter	Male	Non-syndromic hearing loss	/	/	/	/	/	/
**AP5396**	***WFS1***	28802351	F150	P321	Family XH-0	IV-15	Chinese	EA	NM_006005.3	c.2591A>G	p.Glu864Gly	/	/	AD	M	heter	Male	Non-syndromic hearing loss	/	/	/	/	/	/
**AP5397**	***WFS1***	28802351	F150	P322	Family XH-0	III-11	Chinese	EA	NM_006005.3	c.2591A>G	p.Glu864Gly	/	/	AD	M	heter	Female	Non-syndromic hearing loss	/	/	/	/	/	/
**AP5398**	***WFS1***	28468959	F151	P323	/	1	Caucasian	Non-EA	NM_006005.3	c.2425G>A	p.Glu809Lys	/	/	AD	M	heter	Male	Wolfram Syndrome	0.00	/	/	Optic atrophy HP	/	/
**AP5399**	***WFS1***	28468959	F152	P324	/	2	Caucasian	Non-EA	NM_006005.3	c.2425G>A	p.Glu809Lys	/	/	AD	M	heter	Female	Wolfram Syndrome	0.00	/	/	Optic atrophy HP	/	/
**AP5400**	***WFS1***	28468959	F153	P325	/	3	Caucasian	Non-EA	NM_006005.3	c.2425G>A	p.Glu809Lys	/	/	AD	M	heter	Male	Wolfram Syndrome	0.00	/	/	Optic atrophy HP	/	/
**AP5401**	***WFS1***	28468959	F154	P326	/	4	Caucasian	Non-EA	NM_006005.3	c.2489A>C	p.Glu830Ala	/	/	AD	M	heter	Female	Wolfram Syndrome	0.00	/	/	Optic atrophy HP	/	/
[illegible]	[illegible]	[illegible]	[illegible]	[illegible]	[illegible]	[illegible]	[illegible]	[illegible]	[illegible]	[illegible]	[illegible]	[illegible]	[illegible]	[illegible]	[illegible]	[illegible]	[illegible]	[illegible]	[illegible]	[illegible]	[illegible]	[illegible]	[illegible]	[illegible]

**AP5849**	***WFS1***	18806274	F518	P806	LIB1	4	Lebanese	Non-EA	NM_006005.3	c.1382C>G	p.Thr461Ser	c.2524C>T	p.Leu842Phe	AR	M+M	compound I	/
**AP5850**	***WFS1***	18806274	F519	P807	LIB2	1	Lebanese	Non-EA	NM_006005.3	c.2119G>A	p.Val707Ile	c.2649delC	p.Phe884Serfs	AR	M+T	compound I	/
**AP5851**	***WFS1***	18806274	F519	P808	LIB2	2	Lebanese	Non-EA	NM_006005.3	c.2119G>A	p.Val707Ile	c.2649delC	p.Phe884Serfs	AR	M+T	compound I	/
**AP5852**	***WFS1***	18806274	F520	P809	LIB3	1	Lebanese	Non-EA	NM_006005.3	c.1936_1943delTTCTG	p.Phe646Valfs*63	c.1936_1943d	p.Phe646Valfs	AR	T+T	homo	/
**AP5853**	***WFS1***	18806274	F520	P810	LIB3	2	Lebanese	Non-EA	NM_006005.3	c.1936_1943delTTCTG	p.Phe646Valfs*63	c.1936_1943d	p.Phe646Valfs	AR	T+T	homo	/
**AP5854**	***WFS1***	18806274	F521	P811	LIB4	1	Lebanese	Non-EA	NM_006005.3	c.320G>A	p.Gly107Glu	c.320G>A	p.Gly107Glu	AR	M+M	homo	/
**AP5855**	***WFS1***	18806274	F522	P812	LIB5	1	Lebanese	Non-EA	NM_006005.3	c.2119G>A	p.Val707Ile	c.2649delC	p.Phe884Serfs	AR	M+T	compound I	/
**AP5856**	***WFS1***	18806274	F522	P813	LIB5	2	Lebanese	Non-EA	NM_006005.3	c.2119G>A	p.Val707Ile	c.2649delC	p.Phe884Serfs	AR	M+T	compound I	/
**AP5857**	***WFS1***	18806274	F523	P814	LIB6	1	Lebanese	Non-EA	NM_006005.3	c.2467_2469delATC	p.Ile823del	c.2467_2469d	p.Ile823del	AR	M+M	homo	/
**AP5858**	***WFS1***	18806274	F523	P815	LIB6	2	Lebanese	Non-EA	NM_006005.3	c.2467_2469delATC	p.Ile823del	c.2467_2469d	p.Ile823del	AR	M+M	homo	/
**AP5859**	***WFS1***	18806274	F523	P816	LIB6	3	Lebanese	Non-EA	NM_006005.3	c.2467_2469delATC	p.Ile823del	c.2467_2469d	p.Ile823del	AR	M+M	homo	/
**AP5860**	***WFS1***	18806274	F524	P817	LIB7	1	Lebanese	Non-EA	NM_006005.3	c.1936_1943delTTCTG	p.Phe646Valfs*63	c.1936_1943d	p.Phe646Valfs	AR	T+T	homo	/
**AP5861**	***WFS1***	18806274	F524	P818	LIB7	2	Lebanese	Non-EA	NM_006005.3	c.1936_1943delTTCTG	p.Phe646Valfs*63	c.1936_1943d	p.Phe646Valfs	AR	T+T	homo	/
**AP5862**	***WFS1***	18806274	F525	P819	LIB8	1	Lebanese	Non-EA	NM_006005.3	c.1382C>G	p.Thr461Ser	c.2524C>T	p.Leu842Phe	AR	M+M	compound I	/
**AP5863**	***WFS1***	18806274	F526	P820	LIB9	1	Lebanese	Non-EA	NM_006005.3	c.2119G>A	p.Val707Ile	c.2649delC	p.Phe884Serfs	AR	M+T	compound I	/
**AP5864**	***WFS1***	18806274	F526	P821	LIB9	2	Lebanese	Non-EA	NM_006005.3	c.2119G>A	p.Val707Ile	c.2649delC	p.Phe884Serfs	AR	M+T	compound I	/
**AP5865**	***WFS1***	18806274	F527	P822	LIB10	1	Lebanese	Non-EA	NM_006005.3	c.2119G>A	p.Val707Ile	c.2649delC	p.Phe884Serfs	AR	M+T	compound I	/
**AP5866**	***WFS1***	18806274	F528	P823	LIB11	1	Lebanese	Non-EA	NM_006005.3	c.320G>A	p.Gly107Glu	c.320G>A	p.Gly107Glu	AR	M+M	homo	/
**AP5867**	***WFS1***	18806274	F529	P824	LIB12	1	Lebanese	Non-EA	NM_006005.3	c.2068T>G	p.Cys690Gly	c.2068T>G	p.Cys690Gly	AR	M+M	homo	/
**AP5868**	***WFS1***	18806274	F530	P825	LIB13	1	Lebanese	Non-EA	NM_006005.3	c.1582T>G	p.Tyr528Asp	c.1582T>G	p.Tyr528Asp	AR	M+M	homo	/
**AP5869**	***WFS1***	18806274	F531	P826	LIB14	1	Lebanese	Non-EA	NM_006005.3	c.2119G>A	p.Val707Ile	c.2649delC	p.Phe884Serfs	AR	M+T	compound I	/
**AP5870**	***WFS1***	18806274	F532	P827	LIB14	2	Lebanese	Non-EA	NM_006005.3	c.2119G>A	p.Val707Ile	c.2649delC	p.Phe884Serfs	AR	M+T	compound I	/
**AP5871**	***WFS1***	18806274	F533	P828	LIB15	1	Lebanese	Non-EA	NM_006005.3	c.2119G>A	p.Val707Ile	c.2649delC	p.Phe884Serfs	AR	M+T	compound I	/
**AP5872**	***WFS1***	18806274	F534	P829	LIB16	1	Lebanese	Non-EA	NM_006005.3	c.2119G>A	p.Val707Ile	c.2649delC	p.Phe884Serfs	AR	M+T	compound I	/
**AP5873**	***WFS1***	18806274	F535	P830	LIB17	1	Lebanese	Non-EA	NM_006005.3	c.2119G>A	p.Val707Ile	c.2649delC	p.Phe884Serfs	AR	M+T	compound I	/
**AP5874**	***WFS1***	18806274	F536	P831	LIB18	1	Lebanese	Non-EA	NM_006005.3	c.2119G>A	p.Val707Ile	c.2649delC	p.Phe884Serfs	AR	M+T	compound I	/
**AP5875**	***WFS1***	18806274	F536	P832	LIB18	2	Lebanese	Non-EA	NM_006005.3	c.2119G>A	p.Val707Ile	c.2649delC	p.Phe884Serfs	AR	M+T	compound I	/
**AP5876**	***WFS1***	18806274	F536	P833	LIB18	3	Lebanese	Non-EA	NM_006005.3	c.2119G>A	p.Val707Ile	c.2649delC	p.Phe884Serfs	AR	M+T	compound I	/
**AP5877**	***WFS1***	18806274	F537	P834	LIB19	1	Lebanese	Non-EA	NM_006005.3	c.2119G>A	p.Val707Ile	c.2649delC	p.Phe884Serfs	AR	M+T	compound I	/
**AP5878**	***WFS1***	18806274	F537	P835	LIB19	2	Lebanese	Non-EA	NM_006005.3	c.2119G>A	p.Val707Ile	c.2649delC	p.Phe884Serfs	AR	M+T	compound I	/
**AP5879**	***WFS1***	18806274	F537	P836	LIB19	3	Lebanese	Non-EA	NM_006005.3	c.2119G>A	p.Val707Ile	c.2649delC	p.Phe884Serfs	AR	M+T	compound I	/
**AP5880**	***WFS1***	18806274	F538	P837	LIB20	1	Lebanese	Non-EA	NM_006005.3	c.2119G>A	p.Val707Ile	c.2649delC	p.Phe884Serfs	AR	M+T	compound I	/
**AP5881**	***WFS1***	18806274	F538	P838	LIB20	2	Lebanese	Non-EA	NM_006005.3	c.2119G>A	p.Val707Ile	c.2649delC	p.Phe884Serfs	AR	M+T	compound I	/
**AP5882**	***WFS1***	18806274	F539	P839	LIB21	1	Lebanese	Non-EA	NM_006005.3	c.2119G>A	p.Val707Ile	c.2649delC	p.Phe884Serfs	AR	M+T	compound I	/
**AP5883**	***WFS1***	18806274	F540	P840	LIB22	1	Lebanese	Non-EA	NM_006005.3	c.2119G>A	p.Val707Ile	c.2649delC	p.Phe884Serfs	AR	M+T	compound I	/
**AP5884**	***WFS1***	18688868	F541	P841	/	IV:4	Caucasian	Non-EA	NM_006005.3	c.2576G>A	p.Arg859Gln	/	/	AD	M	heter	/
**AP5885**	***WFS1***	18660851	F542	P842	/	Case 1	Mexican	Non-EA	NM_006005.3	c.1354del16	p.Pro451fsX515	c.1354del16	p.Pro451fsX51	AR	T+T	homo	Male
**AP5886**	***WFS1***	18660851	F542	P843	/	Case 2	Mexican	Non-EA	NM_006005.3	c.1354del16	p.Pro451fsX515	c.1354del16	p.Pro451fsX51	AR	T+T	homo	Male
**AP5887**	***WFS1***	18660851	F542	P844	/	Case 3	Mexican	Non-EA	NM_006005.3	c.1354del16	p.Pro451fsX515	c.1354del16	p.Pro451fsX51	AR	T+T	homo	Male
**AP5888**	***WFS1***	18660851	F542	P845	/	Case 4	Mexican	Non-EA	NM_006005.3	c.1354del16	p.Pro451fsX515	c.1354del16	p.Pro451fsX51	AR	T+T	homo	Male
**AP5889**	***WFS1***	18566338	F543	P846	/	Case 1	Italian	Non-EA	NM_006005.3	c.1328G>T	p.Ser443Ile	IVS6+16G>A	#N/A	AR	M+T	compound I	Male
**AP5890**	***WFS1***	18566338	F544	P847	/	Case 2	Italian	Non-EA	NM_006005.3	c.1362_1377del	p.Tyr454*	c.1362_1377d	p.Tyr454*	AR	T+T	homo	Female
**AP5891**	***WFS1***	18566338	F545	P848	/	Case 3	Italian	Non-EA	NM_006005.3	c.1362_1377del	p.Tyr454*	c.1362_1377d	p.Tyr454*	AR	T+T	homo	Male
**AP5892**	***WFS1***	18566338	F546	P849	/	Case 4	Italian	Non-EA	NM_006005.3	c.1230_1233delCTCT	p.Val412Serfs*29	c.1230_1233d	p.Val412Serfs	AR	T+T	homo	Female
**AP5893**	***WFS1***	18566338	F547	P850	/	Case 5	Italian	Non-EA	NM_006005.3	c.1346C>T	p.Thr449Ile	c.1346C>T	p.Thr449Ile	AR	M+M	homo	Female
**AP5894**	***WFS1***	18544103	F548	P851	/	II-2	Caucasian	Non-EA	NM_006005.3	c.2590G>A	p.Glu864Lys	/	/	AD	M	heter	/
**AP6049**	***ZNHIT3***	28335020	F009	P011	Family w	w4	Finnish	Non-EA	NM_004773.3	c.92C>T	p.Ser31Leu	c.92C>T	p.Ser31Leu	AR	M+M	homo	/
**AP6050**	***ZNHIT3***	28335020	F010	P012	/		Finnish	Non-EA	NM_004773.3	c.92C>T	p.Ser31Leu	c.92C>T	p.Ser31Leu	AR	M+M	homo	/
**AP6051**	***ZNHIT3***	28335020	F011	P013	/		Finnish	Non-EA	NM_004773.3	c.92C>T	p.Ser31Leu	c.92C>T	p.Ser31Leu	AR	M+M	homo	/
**AP6052**	***ZNHIT3***	28335020	F012	P014	/		Finnish	Non-EA	NM_004773.3	c.92C>T	p.Ser31Leu	c.92C>T	p.Ser31Leu	AR	M+M	homo	/
**AP6053**	***ZNHIT3***	28335020	F013	P015	/		Finnish	Non-EA	NM_004773.3	c.92C>T	p.Ser31Leu	c.92C>T	p.Ser31Leu	AR	M+M	homo	/
**AP6054**	***ZNHIT3***	28335020	F014	P016	/		Finnish	Non-EA	NM_004773.3	c.92C>T	p.Ser31Leu	c.92C>T	p.Ser31Leu	AR	M+M	homo	/
**AP6055**	***ZNHIT3***	28335020	F015	P017	/		Finnish	Non-EA	NM_004773.3	c.92C>T	p.Ser31Leu	c.92C>T	p.Ser31Leu	AR	M+M	homo	/
**AP6056**	***ZNHIT3***	28335020	F016	P018	/		Finnish	Non-EA	NM_004773.3	c.92C>T	p.Ser31Leu	c.92C>T	p.Ser31Leu	AR	M+M	homo	/
**AP6057**	***ZNHIT3***	28335020	F017	P019	/		Finnish	Non-EA	NM_004773.3	c.92C>T	p.Ser31Leu	c.92C>T	p.Ser31Leu	AR	M+M	homo	/
**AP6058**	***ZNHIT3***	28335020	F018	P020	/		Finnish	Non-EA	NM_004773.3	c.92C>T	p.Ser31Leu	c.92C>T	p.Ser31Leu	AR	M+M	homo	/
**AP6059**	***ZNHIT3***	28335020	F019	P021	/		Finnish	Non-EA	NM_004773.3	c.92C>T	p.Ser31Leu	c.92C>T	p.Ser31Leu	AR	M+M	homo	/
**AP6060**	***ZNHIT3***	28335020	F020	P022	/		Finnish	Non-EA	NM_004773.3	c.92C>T	p.Ser31Leu	c.92C>T	p.Ser31Leu	AR	M+M	homo	/
**AP6061**	***ZNHIT3***	28335020	F021	P023	/		Finnish	Non-EA	NM_004773.3	c.92C>T	p.Ser31Leu	c.92C>T	p.Ser31Leu	AR	M+M	homo	/
**AP6062**	***ZNHIT3***	28335020	F022	P024	/		Finnish	Non-EA	NM_004773.3	c.92C>T	p.Ser31Leu	c.92C>T	p.Ser31Leu	AR	M+M	homo	/
**AP6063**	***ZNHIT3***	28335020	F023	P025	/		Finnish	Non-EA	NM_004773.3	c.92C>T	p.Ser31Leu	c.92C>T	p.Ser31Leu	AR	M+M	homo	/
**AP6064**	***ZNHIT3***	28335020	F024	P026	/		Finnish	Non-EA	NM_004773.3	c.92C>T	p.Ser31Leu	c.92C>T	p.Ser31Leu	AR	M+M	homo	/
**AP6065**	***ZNHIT3***	28335020	F025	P027	/		Finnish	Non-EA	NM_004773.3	c.92C>T	p.Ser31Leu	c.92C>T	p.Ser31Leu	AR	M+M	homo	/
**AP6066**	***ZNHIT3***	28335020	F026	P028	/		Finnish	Non-EA	NM_004773.3	c.92C>T	p.Ser31Leu	c.92C>T	p.Ser31Leu	AR	M+M	homo	/
**AP6067**	***ZNHIT3***	28335020	F027	P029	/		Finnish	Non-EA	NM_004773.3	c.92C>T	p.Ser31Leu	c.92C>T	p.Ser31Leu	AR	M+M	homo	/
**AP6068**	***ZNHIT3***	31048081	F028	P030	/		Caucasian	Non-EA	NM_004773.3	c.92C>T	p.Ser31Leu	c.41G > T	p.Cys14Phe	AR	M+M	compound I	Female
**AP6069**	***ZNHIT3***	34489640	F029	P031	/		Chinese	EA	NM_004773.3	c.1A>T	p.Met1Leu	c.184G>A	p.Val62Ile	AR	M+M	compound I	/

Note: AD= Autosomal dominant; AR= Autosomal recessive; EA= East-Asians; M= Missense; Non-EA= Non East-Asians; T= Truncation;
