# Supplementary material for: Clinical and genetic landscape of optic atrophy in 826 families: insights from 50 nuclear genes
Source: Brain. 2024 Oct 18;148(5):1604–20. doi: 10.1093/brain/awae324 (PMC12073998; doi:10.1093/brain/awae324)
Supplement: awae324_Supplementary_Data [file awae324_supplementary_data.zip › brain-2024-01629-File016.pdf]

Source data for Cox regression

ID	Age at initi	Fundus Gr	Genotype	BCVA	Event
ACO2-P001	42	OA	M+M	0.8	0
ACO2-P002	10	OA+RD	M+M	LP	1
ACO2-P003	16	OA	M+M	0.1	0
ACO2-P004	4	OA+RD	M+M	TL	1
ACO2-P005	12	OA	M+T	0.4	0
ACO2-P006	7	OA	M+M	0.6	0
ACO2-P007	6	OA	M+M	0.2	0
ACO2-P008	3	OA+RD	M+M	0.1	0
ACO2-P009	8	OA+RD	M+M	0.02	1
AFG3L2-P0	45	OA	Missense	0.15	0
AFG3L2-P0	37	OA	Missense	0.05	0
AFG3L2-P0	4	OA	Missense	FC	1
AFG3L2-P0	60	OA	Missense	0.15	0
AFG3L2-P0	6	OA	Missense	0.12	0
AFG3L2-P0	5	OA	Missense	0.1	0
AFG3L2-P0	8	OA	Missense	0.1	0
ATAD3A-P0	13	OA	Missense	0.5	0
BTD-P019	23	OA	M+T	0.1	0
C12ORF65	5	OA+RD	T+T	0.1	0
FDXR-P021	0.5	OA+RD	M+M	NLP	1
FDXR-P022	0.8	OA+RD	M+M	NLP	1
FDXR-P023	2	OA+RD	M+M	NLP	1
FDXR-P024	1	OA+RD	M+M	NLP	1
FDXR-P025	6	OA+RD	M+M	0.05	0
FDXR-P026	16	OA+RD	M+M	0.1	0
FDXR-P027	19	OA+RD	M+M	HM	1
FDXR-P028	12	OA+RD	M+M	0.3	0
FDXR-P029	6	OA+RD	M+M	0.03	1
FDXR-P030	4	OA+RD	M+M	0.25	0
FDXR-P031	7	OA+RD	M+M	0.04	1
FDXR-P032	5	OA+RD	M+M	0.08	0
IBA57-P033	14	OA	M+M	0.1	0
IBA57-P034	6.5	OA	M+T	0.12	0
MCAT-P035	10	LHON	M+T	0.2	0
NBAS-P036	6	OA+RD	M+T	0.05	0
NBAS-P037	10	OA+RD	M+T	0.1	0
NDUFA10-I	6	LHON	M+M	0.2	0
NDUFAF5-I	15	LHON	M+M	0.01	1
NDUFAF5-I	15	LHON	M+M	0.04	1
NDUFS1-P	5	LHON	M+M	1	0
NDUFS1-P	29	LHON	M+T	0.05	0
NR2F1-P04	6	OA	Truncation	0.32	0
NR2F1-P04	15	OA	Truncation	0.8	0

NR2F1-P04	7	OA	Truncation	0.1	0
OPA1-P046	10	OA	Missense	0.1	0
OPA1-P047	16	OA	Truncation	0.2	0
OPA1-P048	6	OA	Truncation	0.4	0
OPA1-P049	24	OA	Truncation	0.3	0
OPA1-P050	7	OA	Truncation	0.1	0
OPA1-P051	25	OA	Truncation	0.3	0
OPA1-P052	27	OA	Missense	0.25	0
OPA1-P053	27	OA	Truncation	0.3	0
OPA1-P054	10	OA	Truncation	0.3	0
OPA1-P055	33	OA	Truncation	0.25	0
OPA1-P056	5	OA	Missense	0.1	0
OPA1-P057	10	OA	Truncation	0.4	0
OPA1-P059	6	OA	Truncation	0.02	1
OPA1-P060	9	OA	Missense	0.1	0
OPA1-P061	4	OA	Truncation	0.03	1
OPA1-P062	5	OA	Missense	0.3	0
OPA1-P063	10	OA	Truncation	0.2	0
OPA1-P065	28	OA	Truncation	0.06	0
OPA1-P066	5	OA	Missense	0.2	0
OPA1-P067	11	OA	Missense	0.2	0
OPA1-P068	23	OA	Missense	0.08	0
OPA1-P069	13	OA	Truncation	0.2	0
OPA1-P070	6	OA	Missense	0.5	0
OPA1-P071	12	OA	Truncation	0.5	0
OPA1-P072	27	OA	Missense	0.16	0
OPA1-P073	9	OA	Missense	0.25	0
OPA1-P074	6	OA	Truncation	0.2	0
OPA1-P075	8	OA	Truncation	0.3	0
OPA1-P076	8	OA	Truncation	0.3	0
OPA1-P077	7	OA	Missense	0.6	0
OPA1-P078	28	OA	Missense	0.07	0
OPA1-P079	21	OA	Truncation	0.07	0
OPA1-P080	7	OA	M+T	0.15	0
OPA1-P081	9	OA	Truncation	0.25	0
OPA1-P082	19	OA	M+T	0.06	0
OPA1-P083	5	OA	Missense	0.1	0
OPA1-P084	19	OA	Missense	0.01	1
OPA1-P085	7	OA	Missense	0.8	0
OPA1-P087	6	OA	Truncation	0.12	0
OPA1-P088	7	OA	Truncation	0.4	0
OPA1-P089	33	OA	Truncation	0.2	0
OPA1-P090	12	OA	Missense	0.25	0
OPA1-P091	13	OA	Truncation	0.12	0
OPA1-P092	10	OA	Missense	0.2	0

OPA1-P093	5	OA	Missense	0.07	0
OPA1-P094	18	OA	Missense	0.1	0
OPA1-P095	5	OA	Missense	0.05	0
OPA1-P096	6	OA	Truncation	0.2	0
OPA1-P097	10	OA	Truncation	0.15	0
OPA1-P098	30	OA	Truncation	0.3	0
OPA1-P099	16	OA	Missense	0.05	0
OPA1-P100	7	OA	Truncation	0.4	0
OPA1-P101	24	OA	Truncation	0.2	0
OPA1-P102	6	OA	Missense	0.2	0
OPA1-P103	14	OA	Missense	0.1	0
OPA1-P104	29	OA	Truncation	0.5	0
OPA1-P105	30	OA	Truncation	0.3	0
OPA1-P106	11	OA	Truncation	0.3	0
OPA1-P107	17	OA	Truncation	0.05	0
OPA1-P108	36	OA	Truncation	0.3	0
OPA1-P109	30	OA	Truncation	0.16	0
OPA1-P110	18	OA	Missense	0.05	0
OPA1-P111	18	OA	Truncation	0.1	0
OPA1-P112	10	OA	Truncation	0.3	0
OPA1-P113	17	OA	Missense	0.1	0
OPA1-P114	7	OA	Truncation	0.25	0
OPA1-P115	18	OA	Missense	0.03	1
OPA1-P116	30	OA	Missense	0.06	0
OPA1-P117	26	OA	Missense	0.06	0
OPA1-P118	39	OA	Missense	0.07	0
OPA1-P120	4	OA	Truncation	0.2	0
OPA1-P121	6	OA	Truncation	0.1	0
OPA1-P122	23	OA	Truncation	0.12	0
OPA1-P123	28	OA	Missense	0.2	0
OPA1-P124	23	OA	M+M	FC	1
OPA1-P125	5	OA	M+T	FC	1
OPA1-P126	9	OA	Truncation	0.25	0
OPA1-P127	7	OA	Truncation	0.05	0
OPA1-P128	37	OA	Missense	FC	1
OPA1-P129	5	OA	Missense	0.15	0
OPA1-P130	5	OA	Missense	0.1	0
OPA1-P131	19	OA	Missense	0.4	0
OPA1-P132	15	OA	Missense	0.15	0
OPA3-P133	6	OA+RD	Missense	0.07	0
OPA3-P134	31	OA	Missense	0.05	0
OPA3-P135	8	OA	Truncation	0.12	0
RTN4IP1-P	7	OA+RD	M+M	0.2	0
SDHA-P138	6	OA	M+M	0.05	0
SLC25A46-	7	OA	M+M	0.16	0

SLC25A46-	6	OA	M+M	0.1	0
SSBP1-P14	31	OA+RD	Missense	0.12	0
SSBP1-P14	6	OA+RD	Missense	0.1	0
SSBP1-P14	5	OA+RD	Missense	0.05	0
SSBP1-P14	4	OA+RD	Missense	0.05	0
SSBP1-P14	5	OA+RD	Missense	0.04	1
SSBP1-P14	4	OA+RD	Missense	0.04	1
TMEM126A	11	OA	T+T	0.1	0
UCHL1-P1	29	OA	Truncation	0.12	0
WFS1-P14	20	OA	M+T	0.1	0
WFS1-P15	9	OA	M+T	0.6	0
WFS1-P15	21	OA	T+T	0.04	1
WFS1-P15	28	OA	M+T	0.04	1
WFS1-P15	23	OA	M+M	0.2	0
WFS1-P15	55	OA	Missense	0.05	0
WFS1-P15	14	OA	M+T	0.2	0
WFS1-P15	13	OA	M+T	0.5	0
WFS1-P15	10	OA	M+M	0.4	0
WFS1-P15	18	OA	T+T	LP	1
WFS1-P15	10	OA	M+T	0.25	0
WFS1-P16	33	OA	M+T	0.2	0
WFS1-P16	28	OA	M+M	0.12	0
WFS1-P16	6	OA	M+T	0.2	0
WFS1-P16	23	OA	M+M	0.2	0
WFS1-P16	4	OA	Missense	0.6	0
