# Supplementary material for: Clinical and genetic landscape of optic atrophy in 826 families: insights from 50 nuclear genes
Source: Brain. 2024 Oct 18;148(5):1604–20. doi: 10.1093/brain/awae324 (PMC12073998; doi:10.1093/brain/awae324)
Supplement: awae324_Supplementary_Data [file awae324_supplementary_data.zip › brain-2024-01629-File017.pdf]

Source data for OCT analysis

GENE	Transcript. No	Family ID	Patient ID	Patients ID in family	OCT (RNFL thickness)							
					OD				OS			
					T	I	S	N	T	I	S	N
ACO2	NM_001098	F001	P001	II-1	26	84	53	12	42	78	87	38
ACO2	NM_001098	F003	P003	II-1	27	52	79	18	29	56	76	18
ACO2	NM_001098	F005	P005	II-1	46	57	76	67	47	53	91	55
ACO2	NM_001098	F006	P006	II-1	48	125	111	66	42	115	108	80
ACO2	NM_001098	F007	P007	II-1	49	94	116	65	48	89	118	87
AFG3L2	NM_006796	F014	P015	II-1	42	60	69	53	40	58	67	54
AFG3L2	NM_006796	F015	P016	II-1	46	75	85	56	41	78	93	60
AFG3L2	NM_006796	F016	P017	II-1	22	55	74	35	38	51	79	34
AFG3L2	NM_006796	F009	P010	II-1	45	122	125	72	44	132	122	71
ATAD3A	NM_001170	F017	P018	II-1	38	94	133	65	34	106	132	74
BTD	NM_001370	F018	P019	II-1	29	67	82	49	31	77	81	49
IBA57	NM_001010	F033	P040	II-1	24	97	94	52	24	95	110	49
MCAT	NM_014507	F034	P041	II-1	43	110	143	66	37	124	160	78
NDUFA10	NM_004544	F036	P044	II-1	50	58	56	57	46	94	114	51
NDUFAF5	NM_024120	F037	P047	II-1	49	144	175	94	74	175	165	62
NDUFAF5	NM_024120	F037	P047	II-2	38	109	114	61	83	164	145	65
NDUFS1	NM_005006	F038	P048	II-1	30	91	103	67	92	159	123	66
NDUFS1	NM_005006	F039	P050	II-1	71	116	164	79	67	126	145	53
NR2F1	NM_005654	F040	P052	II-1	53	83	96	39	60	59	97	34
NR2F1	NM_005654	F041	P053	II-1	46	106	94	59	44	93	96	55
OPA1	NM_015560	F043	P055	II-1	46	73	64	50	29	66	95	46
OPA1	NM_015560	F044	P056	II-1	46	70	85	61	45	70	94	60
OPA1	NM_015560	F045	P059	II-1	52	83	124	82	36	89	134	85
OPA1	NM_015560	F048	P062	II-1	46	118	116	75	49	117	125	73
OPA1	NM_015560	F049	P063	II-1	40	120	125	77	50	109	129	77
OPA1	NM_015560	F050	P064	II-1	57	88	91	70	38	78	99	60
OPA1	NM_015560	F052	P066	II-1	40	89	99	75	38	79	83	65
OPA1	NM_015560	F053	P067	II-1	30	56	60	32	21	50	61	33
OPA1	NM_015560	F054	P069	II-1	28	80	106	53	32	75	122	52
OPA1	NM_015560	F056	P071	II-1	/	/	/	/	23	61	64	26
OPA1	NM_015560	F057	P072	II-1	26	43	48	25	24	38	36	24
OPA1	NM_015560	F058	P073	II-1	17	37	48	29	29	40	54	27
OPA1	NM_015560	F060	P075	II-1	46	72	75	68	39	88	91	67
OPA1	NM_015560	F061	P076	II-1	36	57	57	57	23	50	74	39
OPA1	NM_015560	F061	P077	I-2	22	70	72	78	18	74	86	57
OPA1	NM_015560	F064	P080	II-1	52	72	78	50	56	82	84	51
OPA1	NM_015560	F065	P081	II-1	21	51	103	50	21	57	93	40
OPA1	NM_015560	F066	P082	II-1	52	59	52	39	37	52	66	41
OPA1	NM_015560	F068	P084	II-1	38	84	78	59	38	69	76	53
OPA1	NM_015560	F070	P086	II-1	26	60	94	52	53	59	95	53
OPA1	NM_015560	F071	P087	II-1	n.d	n.d	n.d	n.d	n.d	n.d	n.d	n.d
OPA1	NM_015560	F072	P088	II-1	43	65	109	63	47	80	114	58
OPA1	NM_015560	F073	P090	I-2	29	74	74	38	24	73	77	37
OPA1	NM_015560	F076	P093	II-1	45	71	92	59	49	74	103	60
OPA1	NM_015560	F077	P094	II-1	53	57	63	53	48	56	63	50
OPA1	NM_015560	F078	P095	II-1	n.d	n.d	n.d	n.d	29	78	88	44
OPA1	NM_015560	F079	P096	II-1	57	65	54	57	54	69	64	57
OPA1	NM_015560	F083	P100	II-1	34	116	127	79	38	115	134	77
OPA1	NM_015560	F092	P109	II-1	36	87	131	52	28	84	136	63
OPA1	NM_015560	F093	P110	II-1	25	71	100	48	26	98	71	46
OPA1	NM_015560	F094	P111	II-1	39	71	84	64	43	65	78	63
OPA1	NM_015560	F095	P112	II-1	40	82	103	53	27	92	107	53
OPA1	NM_015560	F096	P113	II-1	55	91	107	63	40	93	118	61
OPA1	NM_015560	F097	P114	II-1	24	86	110	55	40	75	119	58
OPA1	NM_015560	F099	P116	II-1	45	98	131	91	47	94	133	81
OPA1	NM_015560	F100	P117	II-1	26	58	117	48	25	61	112	43
OPA1	NM_015560	F102	P119	II-1	44	88	102	79	49	81	109	72
OPA1	NM_015560	F104	P121	II-1	50	82	104	66	53	80	104	70
OPA1	NM_015560	F106	P123	II-1	46	121	160	66	53	110	142	63
OPA1	NM_015560	F107	P124	II-1	31	63	79	47	56	65	91	41
OPA1	NM_015560	F108	P125	II-1	24	72	100	45	27	68	107	37
OPA1	NM_015560	F109	P126	II-1	43	53	50	54	51	46	48	59
OPA1	NM_015560	F110	P127	II-1	43	46	45	37	45	45	50	40
OPA1	NM_015560	F114	P131	II-1	27	67	111	55	30	54	110	60
OPA1	NM_015560	F115	P132	II-1	43	68	76	62	41	66	68	59
OPA1	NM_015560	F116	P133	II-1	56	82	91	61	41	74	96	54
OPA1	NM_015560	F117	P134	II-1	22	28	28	24	16	22	27	23
OPA1	NM_015560	F119	P136	II-1	43	83	124	51	38	81	109	47
OPA1	NM_015560	F122	P140	II-1	65	88	77	52	46	68	103	49
OPA1	NM_015560	F124	P143	II-1	44	80	80	57	48	78	86	52
OPA1	NM_015560	F063	P079	II-1	27	93	89	52	25	84	95	47
OPA1	NM_015560	F074	P091	II-1	42	65	82	49	50	73	76	54
OPA1	NM_015560	F075	P092	II-1	39	94	110	61	42	95	111	57
OPA3	NM_025136	F126	P145	II-1	39	69	87	46	47	78	94	44
RTN4IP1	NM_032730	F129	P148	II-1	19	86	77	49	26	88	83	35
SLC25A46	NM_138773	F132	P151	II-1	42	110	102	53	36	91	101	38
SSBP1	NM_003143	F133	P152	I-2	37	52	46	62	37	45	46	54
SSBP1	NM_003143	F133	P153	II-1	46	67	57	38	22	65	79	44
SSBP1	NM_003143	F133	P155	II-2	19	98	94	48	26	99	77	48
SSBP1	NM_003143	F135	P157	II-1	26	74	42	34	26	52	39	26
TMEM126A	NM_032273	F136	P159	II-1	30	87	107	71	39	74	100	67
WFS1	NM_006005	F139	P163	II-1	29	58	71	34	32	60	75	35
WFS1	NM_006005	F142	P166	II-1	30	72	92	49	28	76	87	49
WFS1	NM_006005	F144	P168	II-1	25	60	68	37	23	58	77	45
WFS1	NM_006005	F145	P164	II-1	31	76	75	46	36	66	83	45
WFS1	NM_006005	F146	P156	II-1	42	54	66	57	34	53	67	51
WFS1	NM_006005	F147	P157	II-1	63	66	60	53	56	62	56	53
WFS1	NM_006005	F148	P158	II-1	37	70	65	42	35	66	76	44
WFS1	NM_006005	F149	P159	II-1	33	83	73	16	26	66	78	12
WFS1	NM_006005	F152	P162	II-1	50	50	66	55	53	64	63	56
WFS1	NM_006005	F153	P161	II-1	45	55	66	51	45	61	66	52
WFS1	NM_006005	F154	P165	II-1	54	128	142	52	46	102	120	65

Note = I, Inferior; N, Nasal; S, Superior; T, Temporal;
